# Tumor-derived exosomal CCT6A serves as a matchmaker introducing chemokines to tumor-associated macrophages in pancreatic ductal adenocarcinoma

**DOI:** 10.1038/s41419-025-07720-y

**Published:** 2025-05-15

**Authors:** Tianyin Ma, Wing-Wa Guo, Minghe Zhang, Wenzhi He, Cairang Dongzhi, Xiangdong Gongye, Peng Xia, Yibo Chai, Zhang Chen, Yimin Zhu, Chengming Qu, Jie Liu, Zhiyong Yang, Weijie Ma, Ming Tian, Yufeng Yuan

**Affiliations:** 1 Department of Hepatobiliary & Pancreatic Surgery, Zhongnan Hospital of Wuhan University, Wuhan, PR China; 2 Clinical Medicine Research Center for Minimally Invasive Procedure of Hepatobiliary & Pancreatic Diseases of Hubei Province, Hubei, PR China; 3 Department of Chemistry and Molecular Biology, Sahlgrenska Akademin, Göteborg Universitet, Gothenburg, Vastra Gotalands Sweden; 4 Department of Chemistry, The University of Chicago, Chicago, IL USA; 5 Taikang Center for Life and Medical Sciences of Wuhan University, Hubei, PR China

**Keywords:** Cancer, Cell biology

## Abstract

M2-polarized tumor-associated macrophages (TAMs) are a key factor contributing to the poor prognosis of pancreatic ductal adenocarcinoma (PDAC). While various factors within the tumor microenvironment (TME) drive their formation, the role of PDAC-derived exosomes in this process remains unclear. We aim to clarify the regulatory impacts of tumor-derived exosomes to TAMs. After the intratumoral injection to subcutaneous tumor of C57BL/6 mice, we demonstrated PDAC-derived exosomes exacerbate PDAC progression, accompanied with upregulated M2 phenotype of TAMs and unaffected proliferation signatures. Through intratumoral injection model and multi-Omics analyses, we identified CCT6A as a novel tumor-derived exosomal protein, bridging TAMs M2 polarization and PDAC prognosis. Co-culture with exosomes derived from CCT6A^high^ PDAC leads to greater M2 phenotype of TAMs via PI3K-AKT signaling. According to proteomics data, chemokines’ abundance reduces over tenfold once exosomal CCT6A absence, including CXCL1, CXCL3, CCL20 and CCL5, whose interaction with CCT6A in PDAC cells was confirmed by interactomics data. Moreover, we found silencing CCT6A abrogated the antagonism effects of CD47 antibody immunotherapy. Our findings implied that the subunit of the T-complex protein Ring Complex (TRiC) CCT6A serves as a matchmaker during exosome-mediated chemokines transfer from PDAC to TAMs. Silencing CCT6A effectively sensitized PDAC to CD47 antibody immunotherapy in vivo.

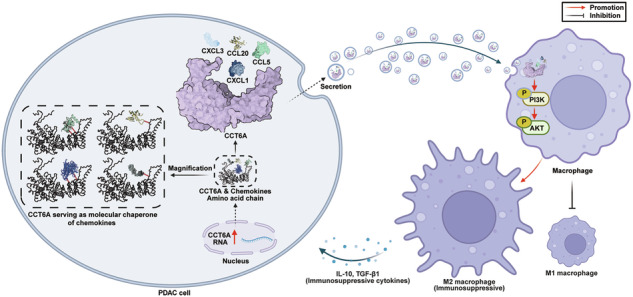

## Introduction

Pancreatic ductal adenocarcinoma (PDAC) persists as one of the world’s most formidable and lethal malignancies, with a 5-year survival rate below 10% [1]. By 2030, PDAC is predicted to rise to the second leading death cause for cancer [2]. PDAC is usually diagnosed in an advanced stage; thus, only 15–20% of patients can benefit from curative surgery [3]. For the vast majority, targeted and immune therapies, to which PDAC is refractory, remains the last treatment option.

PDAC is characterized by a complex and immunosuppressive tumor microenvironment (TME). This inherent property significantly contributes to the limited efficacy of various therapies. There are mounting of myeloid-derived suppressor cells, T cells, tumor correlated fibroblasts and macrophages, in its microenvironment. Among which, M2 macrophages and M2 macrophages-associated signaling pathways functioned as pivotal players in suppressing adaptive immunity, facilitating angiogenesis, and accelerating tumor growth [4, 5]. Therefore, it is urgent to find effective strategies to study pancreatic TME.

Previous study highlighted macrophages were activated into the M2 phenotype to promote the epithelial-mesenchymal transition, invasion, and migration of pancreatic tumor cells [6]. Additionally, high levels of CD163^+^ M2 macrophages infiltration were predicted to be worse prognosis [7]. The causes of M2-polarized tumor-associated macrophages (TAMs) are multifactorial. The elevated cytokines secreted by PDAC cells induce the polarization of tumor-associated macrophages towards M2 phenotype [8–11].

Exosomes mediate the intercellular material delivery and information communication, emerging as lipid double-coated extracellular vesicles [12]. Exosomes derived from PDAC are intricately linked to the TME [13]. However, the impacts of PDAC-derived exosomes on TAMs M2 polarization and the resultant mechanism remain largely unknown. Thus, further investigation may benefit for the protracted management for PDAC patients. For this reason, we sought to study the role of exosomes in PDAC.

Here we report that PDAC-derived exosomes induce M2 polarization of macrophages and increase the tumor burden in vitro and in vivo. To resolve the underlying mechanism, we performed proteomics analysis to PDAC-derived exosomes and focused on the candidates associated with PDAC burden and M2 macrophage status. We identified CCT6A as a potentially pivotal exosomal molecule, which is one of the subunits of TRiC (T-complex protein Ring Complex, responsible for the folding and maturation of many proteins). Transcriptomic and comparative proteomic analyses revealed that PDAC-derived exosomal CCT6A activates the PI3K-AKT pathway in macrophages, driving M2 polarization through increased exosomal chemokine levels. Interactomics data confirmed the interaction between CCT6A with these chemokines in PDAC cells. We further demonstrated that highly CCT6A-expressed PDAC-derived exosomes counteract the efficacy of anti-CD47 immune therapy. These results provide useful mechanistic information for the understanding of PDAC and the development of personalized therapies for metastatic PDAC patients.

## Materials and methods

### Exosome isolation and identification

Exosomes were successfully isolation and identified via diverse techniques [14], such as transmission electron microscopy (TEM), cryo-electron microscopy (Cryo-EM), nanoparticle tracking analysis (NTA), atomic force microscopy (AFM), dynamic light scattering (DLS), and resonance pulse sizing (RPS). Each specific method is described in the Materials and methods of Supplementary Materials.

### Syngeneic mouse model

All animal-related protocols were approved by the Institutional Animal Care and Use Committee (IACUC) of the Wuhan University Center for Animal Experiments (WP20230660). Six-week-old female C57BL/6 mice were purchased from the Laboratory Animal Center of Wuhan University and housed in a specific-pathogen-free (SPF) environment. The environment was maintained on a 12/12 h day/night cycle. Mice with tumor rupture were excluded from the study midway through the experiment. The exclusion criteria were pre-established.

For intratumoral injection experiments, the animals were randomized into Exo and Saline groups and blinding of the investigator was not performed. Each group consisted of 10 mice. Seven days after establishing the syngeneic mouse model, based on the NTA results, exosomes (1 × 10^10^ particles per 100 μL) were injected every three days into the subcutaneous tumors of the mice in the Exo group, for a total of four injections. Fresh exosomes from the same extraction batch were used in this assay to ensure consistency. Mice in the Saline group received an equal volume of saline injected into the subcutaneous tumor.

For CD47 nanobody injection model, the animals were randomized into sh-NC+Saline, sh-NC+anti-CD47, sh-CCT6A+Saline and sh-CCT6A+anti-CD47 groups and blinding of the investigator was not performed. Each group consisted of 10 mice. Seven days after establishing the syngeneic mouse model, anti-CD47 nanobody (200 μg per mouse) and an equal volume of saline were administered via tail vein injection. The anti-CD47 nanobody was a gift provided by the Shenzhen Clinical Research Center for Respirology [15].

The syngeneic mouse model was established via the subcutaneous injection of murine KPC tumor cells (4 × 10^6^ cells per 30 μL of saline). The mice were euthanized either when signs of discomfort were observed or when the tumor reached the maximum allowed volume of 2000 mm³. Subcutaneous tumors were collected and weighed after 18 days.

### Bioinformatics analysis

Detailed procedures for transcriptome library construction, RNA sequencing, data quality control, and alignment processing are provided in the Materials and methods of Supplementary Materials. Immune cell infiltration in each tumor sample from the five datasets was analyzed using the “CIBERSORT” and “TIMER” algorithms with the R package IOBR. Detailed procedures for public datasets analysis and immune infiltration analysis are also provided in Supplementary Materials. The sample processing for liquid chromatography and protein identification for 4D-DIA quantitative proteomics are also described in the Materials and methods of Supplementary Materials. GSVA SCORES were calculated difference with limma R package. Immune cells infiltration abundance difference was calculated by Wilcoxon rank-sum test. Differentially expressed proteins were identified based on the criteria *p* ≤ 0.05 and FC ≥ 1.5 or F ≤ 0.6667. Enrichment analysis was performed using clusterProfiler. Further details are available in Supplementary Materials.

### Patient sample collection

Human pancreatic tumor samples were obtained from 33 patients with a histologically confirmed status. These patients underwent surgical resection at the Department of Hepatobiliary & Pancreatic Surgery, Zhongnan Hospital of Wuhan University, between 2020 and 2023 and did not receive neoadjuvant chemotherapy. The criteria were pre-established. Before participating, all patients were required to provide written informed consent. The procedures for sample collection and processing were carried out in accordance with the guidelines defined in the Declaration of Helsinki, with the approval of the Medical Ethical Committee of Zhongnan Hospital of Wuhan University (2024096K).

### IHC staining

Paraffin-embedded tissue was sectioned, and the sections were deparaffinized and rehydrated with xylene and ethanol on the basis of previous studies [16, 17]. For antigen retrieval, the slides were placed in Tris-EDTA buffer (pH 9.0) and heated for 15 min. Subsequently, the slides were treated with 3% hydrogen peroxide solution for 10 min. After three washes with TBST, the slides were blocked with 10% rabbit serum for 1 h. The slides were incubated with primary antibodies for 24 h at 4 °C and then with HRP-conjugated secondary antibodies for 60 min. The slides were washed and incubated with DAB chromogenic solution to achieve the desired staining intensity. Dehydrated slides (70% ethanol‒xylene substitute) were air dried and mounted.

### Scoring of IHC staining

A semiquantitative scoring system was employed, considering both the staining intensity (0 to 4: 0 = no staining, 1 = very faint staining, 2 = weak staining, 3 = moderate staining, 4 = strong staining) and the proportion of stained cells (1 to 4: 1 ≤ 10%, 2 = 10–50%, 3 = 50–80%, 4 = ≥ 80%). The final score, calculated by multiplying the staining intensity and proportion values, ranged from 0 to 16 and was categorized into nine levels (1, 2, 3, 4, 6, 8, 9, 12, and 16) for analysis. CCT6A protein expression was considered high if the final score was ≥6.

### Immunoelectron microscopy

Immunoelectron microscopy was used to assess protein expression on exosomes. For immunogold labeling, exosomes in Saline at a concentration of 1 × 10^9^ particles/mL were placed onto glow-discharged copper grids, and the grids were then blocked and incubated with a rabbit anti-human polyclonal antibody (CCT6A, Proteintech Group, 19793-1-AP) that specifically recognizes the CCT6A protein. This antibody has been previously validated in western blot and IHC experiments. Next, the grids were incubated with anti-rabbit secondary antibodies conjugated to very small gold particles, either 4 nm (Jackson ImmunoResearch, 111-185-144) or 10 nm in diameter (Solarbio, K1034G-G35). After each staining step, the grids were thoroughly rinsed five times with saline and ten times with ultrapure water (ddH_2_O). Finally, they were stained with 2% uranyl acetate for contrast enhancement. The prepared samples were examined under a powerful transmission electron microscope (Thermo Fisher Scientific, Talos L120C G2).

### Exosome uptake assay

DiR dye (dlmbiotech) was added to the purified exosome suspension to confirm whether exosomes can be internalized by macrophages. The mixture was incubated overnight at 4 °C in a dark environment and was then centrifuged at 100,000 × *g* for 1.5 h to remove excess dye. The resulting exosomes were subsequently resuspended in saline. Macrophage nuclei were subsequently stained with DAPI, and exosome uptake was finally observed with a fluorescence confocal microscope (Leica TCS SP8 X).

### Enzyme-linked immunosorbent assay (ELISA)

An ELISA kit (Multi Sciences) was used to assess IL-10, TGF-β1, and TNF-α expression. The experiment was conducted in accordance with the protocol provided by the kit. The harvested cell culture supernatant was added to a precoated enzyme plate as previously described [18, 19]. A stop solution was added before the absorbance was measured with a microplate reader.

### Flow cytometry

Viable cells were collected before being suspended in saline. The cells were labeled with fluorescently labeled anti-CD11b and anti-CD163 or anti-CD86 antibodies. These cells were centrifuged to remove the supernatant following a 30-min incubation in the dark. The cells were resuspended in saline before analysis via flow cytometry (Beckman Coulter, USA).

### Co-IP

Cells were lysed using a buffer containing 1% NP-40 (Thermo Fisher Scientific), 20 mmol/L Tris-HCl (pH 7.4; Solarbio), 5 mmol/L sodium pyrophosphate (Solarbio), and a complete protease inhibitor cocktail (Sigma-Aldrich). The lysates were incubated with 2 μg of control IgG (Abclonal) or specific antibody and 40 μL of A/G agarose beads, followed by overnight rotation at 4 °C to promote antibody binding. The antibody-bound A/G sepharose beads were subsequently washed five times with 1 mL of lysis buffer. The beads were then boiled at 96 °C for 5 min to elute the bound proteins. After centrifugation at 10,000 rpm for 1 min, the supernatant was collected. The samples were immediately subjected to SDS-PAGE and western blotting for subsequent analysis [20, 21].

### GST pulldown

GST-tagged fusion proteins were first expressed in *Escherichia coli* and purified using Pierce™ GST Spin Purification Kit (Thermo Fisher Scientific). Purified proteins were incubated with the target protein in binding buffer (containing Tris-HCl, NaCl, and a protease inhibitor cocktail) at 4 °C for 2–4 h with gentle rotation. After incubation, the beads were washed extensively with binding buffer to remove unbound proteins. Bound proteins were eluted using glutathione elution buffer and subsequently analyzed by SDS-PAGE, followed by western blotting and CBB staining for detection of interactions.

### Molecular docking

The targeted proteins’ sequences were searched in the NCBI database and then submitted to AlphaFold3 for structure prediction. The docking process was performed using software AutoDock Vina, with the binding pocket defined based on known active sites (A domain). To perform protein-protein docking, the structures of the interacting proteins were submitted to the HDOCK server. The three-dimensional structure of the protein-protein complex was then visualized using PyMOL, highlighting key interaction interfaces involved in binding.

### Statistical analysis

The data were statistically analyzed via SPSS 22.0 and GraphPad Prism 9.0. Sample size was determined using https://powerandsamplesize.com/. Data are presented as mean ± standard deviation (SD). For comparisons between two groups, Student’s *t*-test was applied. Survival analysis was conducted using the Kaplan-Meier method, with differences assessed via the log-rank test. Multiple group comparisons were performed using one-way ANOVA to compare means across multiple groups and two-way ANOVA to evaluate the main effects and interactions between two independent factors. When ANOVA results indicated statistical significance, post hoc tests were conducted. Specifically, Dunnett’s test was applied for multiple experimental groups versus a single control group to determine which groups showed significant differences compared to the control, while Tukey’s test was used for pairwise comparisons among all groups. For nonparametric analyses, the Wilcoxon rank-sum test was applied. Simple linear regression and the Pearson correlation coefficient were used to assess relationships between variables. All data met the assumptions of the relevant tests, and variances were comparable between the groups undergoing statistical comparisons. All cellular experiments were independently repeated at least three times, and all animal experiments were performed with at least six independent biological replicates to ensure reproducibility and reliability. All statistical analyses were conducted using two-sided tests, and statistical significance was set as follows: n.s. no significant, *p ≤ 0.05, **p ≤ 0.01, ***p ≤ 0.001.

## Results

### PDAC-derived exosomes promote macrophage M2 polarization and drive tumor progression

**Fig. 1:**
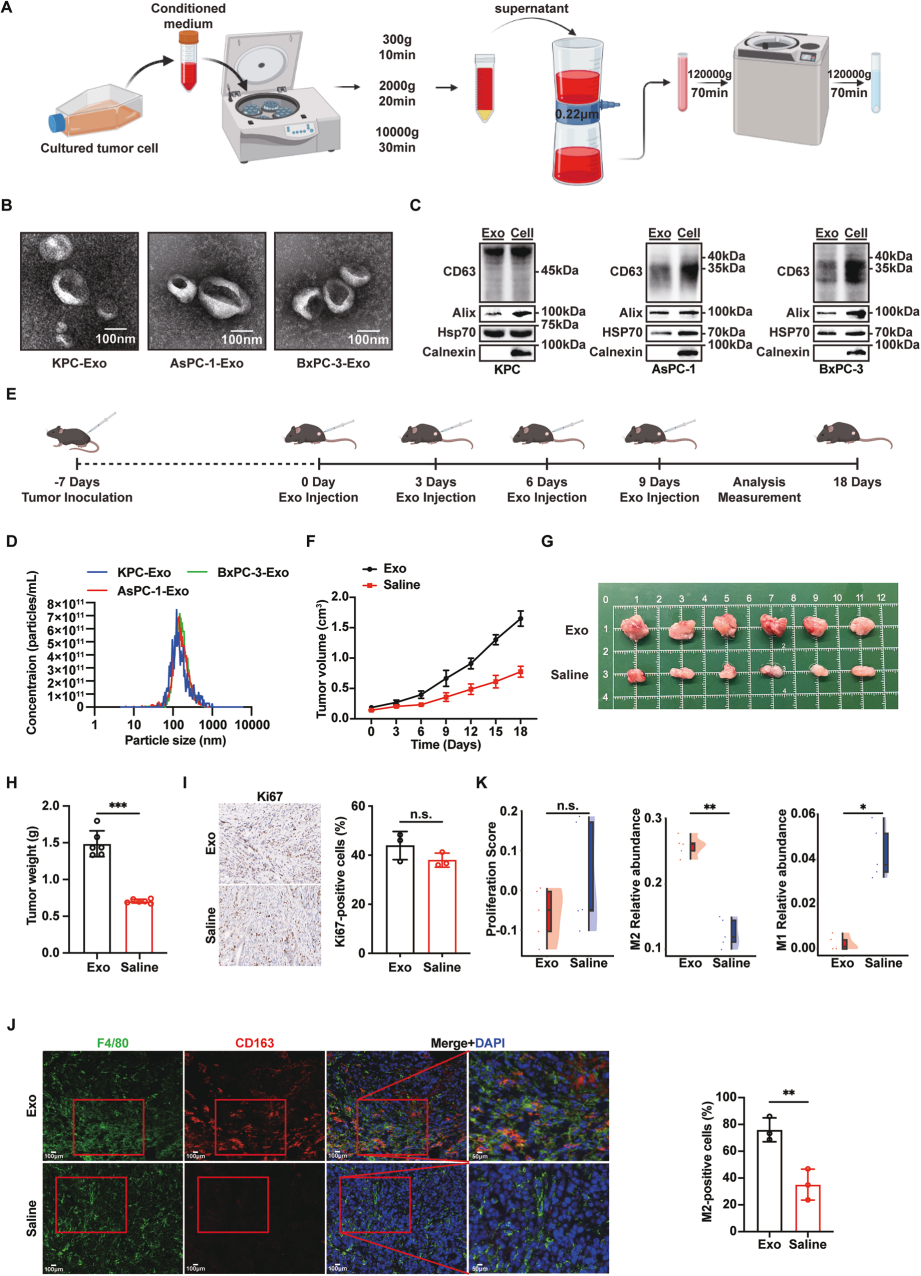
PDAC-derived exosomes induce TAMs towards M2 polarization and accelerate tumor progression consequently. **A** Schematic diagrams of the exosome isolation procedures. Created with BioRender.com. **B** Representative TEM images of exosomes isolated from KPC (KPC-Exo, left), AsPC-1 (AsPC-1-Exo, middle), and BxPC-3 (BxPC-3-Exo, right) cells. Scale bar, 100 nm. **C** Analysis of the expression of specific exosomal biomarkers in exosomes isolated from KPC, AsPC-1, and BxPC-3 cells via western blotting. **D** Size distribution of exosomal particles in exosomes isolated from KPC, AsPC-1, and BxPC-3 cells, as measured by NTA. **E** Schematic diagrams of evaluating PDAC-derived exosomes’ impacts on PDAC tumor in C57BL/6 mice. Created with BioRender.com. The exosome concentration for injection was diluted based on the NTA results. Each mouse in the exosome group was injected with 100 µL exosome solution per dose, containing 1×10¹⁰ particles of exosomes, with a total of 4 injections. (*n* = 6). **F** Tumor volume curves. Black represents intratumoral injection of exosomes (Exo), and red represents intratumoral injection of saline (Saline). (*n* = 6). **G** Representative images of subcutaneous tumor tissue. (*n* = 6). **H** Tumor weights of subcutaneous tumors. (*n* = 6). t-test analysis. **I** Representative Ki-67 staining IHC images (left) and positive cell count statistics (right) of subcutaneous tumor tissue. Scale bar, 100 μm for full images; 50 μm for inset images. (*n* = 3). *t*-test analysis. **J** Representative IF images (left) and positive cell (F4/80^+^CD163^+^) count statistics (right) of subcutaneous tumor tissue. Scale bar, 100μm for full images; 50μm for inset images. (*n* = 3). *t*-test analysis. **K** Analysis of proliferation (left) and M2/M1 macrophage infiltration (right) in tumors. (*n* = 5). Wilcoxon rank-sum test analysis. Data presented as mean ± SD. n.s. no significant, *p ≤ 0.05, **p ≤ 0.01, ***p ≤ 0.001.

To investigate the impact of PDAC cell-derived exosomes on macrophage polarization, we differentiated human THP-1 cells into M0 macrophages and indirectly co-cultured them with the highly metastatic PDAC cell line AsPC-1 or the poorly metastatic BxPC-3 in a Transwell chamber (Fig. S1A). Exosomes were isolated as described in Fig. 1A, and their quality was confirmed through TEM, western blotting, and NTA (Fig. 1B–D). Following co-culture, RT-qPCR and immunofluorescence (IF) analyses revealed a significant upregulation of M2 macrophage-associated markers, while M1 marker expression was slightly reduced (Fig. S1B–E). Notably, these effects were abolished upon treatment with GW4869, an inhibitor of exosome secretion (Fig. S1B–E). These results provide preliminary evidence that PDAC cells can promote M2 macrophage polarization via exosome secretion.

The results prompted us to further investigate the role of tumor-derived exosomes in TME, we then performed intratumoral injections of purified exosomes into subcutaneous tumors (Fig. 1E). Exosome injection significantly accelerated tumor growth compared to saline controls, as demonstrated by tumor volume measurements and gross morphology (Fig. 1F–H). Exosome treatment did not significantly enhance tumor cell proliferation or induce cell death, but it did lead to increased infiltration of M2 macrophages, as confirmed by immunohistochemistry (IHC) and IF analyses (Fig. 1I, J). Furthermore, transcriptomic analysis of subcutaneous tumors indicated no change in the proliferation rate but a notable increase in the proportion of M2 macrophages (Figs. 1K, S1F–G and S2A–C). Collectively, these findings suggest that PDAC-derived exosomes mediate M2 macrophage polarization, thereby contributing to tumor progression.

### Multi-Omics analyses identify PDAC cell-secreted exosomal CCT6A as the driver of macrophage M2 polarization and PDAC progression

**Fig. 2:**
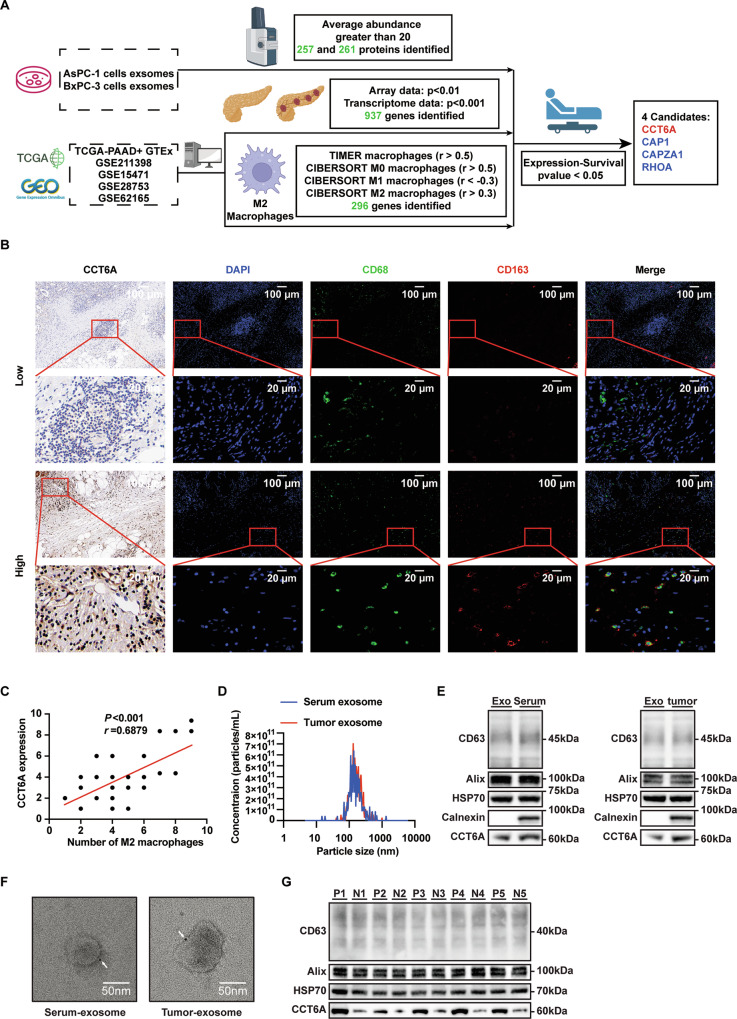
Multi-omics analyses identify exosomal CCT6A as a critical contributor to TAM M2 polarization. **A** Schematic diagram of experimental design assessing the potential PDAC-derived M2 TAM-induced exosomal proteins using proteomic detection and public datasets, which identifying CCT6A, CAP1, CAPZA1 and RHOA as the promising contributors. Created with BioRender.com. **B** IHC analysis of CCT6A and IF staining of CD68 (green) and CD163 (red) in tumor tissue sections from 33 PDAC patients. Scale bar, 100 μm. **C** Scatter diagram of the tumoral CCT6A expression levels and M2 macrophage infiltration scores in 33 PDAC patients. Simple linear regression and the Pearson correlation coefficient analysis. **D** Size distribution of human PDAC-derived exosomal particles, as measured by NTA. Blue represents exosomes derived from serum, and red represents exosomes derived from tumor. **E** Analysis of the expression of specific exosomal biomarkers and CCT6A in exosomes isolated from serum (left) and tumor (right) via western blotting. **F** Immunoelectron microscopy analysis of CCT6A in exosomes isolated from serum (left) and tumor (right). Scale bar, 50 nm. **G** Comparison of serum-originated exosomal CCT6A levels by western blotting. ‘P’ represents exosomes derived from serum of PDAC patients, while ‘N’ represents exosomes derived from serum of healthy individuals. (*n* = 5). n.s. no significant, *p ≤ 0.05, **p ≤ 0.01, ***p ≤ 0.001.

We employed proteomic analysis to identify key proteins within the exosomes derived from the PDAC cells. To ensure the reliability of our results, we performed detailed characterization analyses of the purified exosomes (including DLS, RPS, Cryo-EM and AFM), which demonstrated their purity (Fig. S3A–E). Among the 265 identified proteins, we narrowed down the focus to four candidates whose aberrant upregulation is associated with poor prognosis and enhanced M2 macrophage polarization in PDAC (Figs. 2A, S3F–G and S4A–D). Indeed, the oncogenic roles of CAP1, CAPZA1 and RHOA were well investigated [22–24]. Therefore, we selected CCT6A for further investigation.

To verify the relationship between tumoral CCT6A expression and TAM status, we collected tumor samples from 33 PDAC patients. Pathological staining revealed that CCT6A expression was positively correlated with histological differentiation and tumor stage (Table 1). Moreover, combined IHC analysis of TAM infiltration markers confirmed a positive correlation between CCT6A expression and the M2 macrophage phenotype (Fig. 2B, C).

**Table 1:** Summary descriptive table by groups of ‘CCT6A’.

	Low	High	Overall *p-value*
	*N* = 25	*N* = 8	
Age:			1.000
≤60	13 (52.0%)	4 (50.0%)	
>60	12 (48.0%)	4 (50.0%)	
Sex:			0.420
Female	8 (32.0%)	4 (50.0%)	
Male	17 (68.0%)	4 (50.0%)	
Smoking:			1.000
Never	18 (72.0%)	6 (75.0%)	
Ever	7 (28.0%)	2 (25.0%)	
Drinking:			1.000
Never	23 (92.0%)	8 (100%)	
Ever	2 (8.00%)	0 (0.00%)	
Histologic differentiation:			0.036
Low	5 (20.0%)	5 (62.5%)	
Moderate and high	20 (80.0%)	3 (37.5%)	
Tumor stage:			0.010
T1 + T2	22 (88.0%)	3 (37.5%)	
T3 + T4	3 (12.0%)	5 (62.5%)	
Nodal stage:			0.574
N0	3 (12.0%)	2 (25.0%)	
N1-2	22 (88.0%)	6 (75.0%)	
Metastatic stage:			1.000
MX/M0	24 (96.0%)	8 (100%)	
M1	1 (4.00%)	0 (0.00%)	

Additionally, exosomes isolated from the serum and pancreatic tissue of PDAC patients were examined. Using NTA, western blotting, and immunoelectron microscopy, we confirmed the presence of CCT6A in PDAC-derived exosomes (Figs. 2D–F and S5A, B). Notably, exosomal CCT6A levels were significantly elevated in the serum of PDAC patients (Fig. 2G). Collectively, these findings suggest that exosomal CCT6A in PDAC may contribute to M2 macrophage polarization, thereby promoting tumor progression.

### Macrophages that take up exosomal CCT6A undergo phenotypic alterations

We next explored the effects of PDAC-derived exosomal CCT6A on TAMs. The successful silencing of CCT6A was confirmed by western blotting analysis following the infection of PDAC cells with shRNA-expressing lentivirus (Fig. S6A). Exosomes isolated from these transfected cells were subsequently used to directly treat macrophages. The results of NTA and the endocytosis rate measurement of DiR-labeled PDAC-derived exosomes, indicated that CCT6A silencing did not affect the exosomes size and its exosome uptake by TAMs (Fig. S6B–D).

**Fig. 3:**
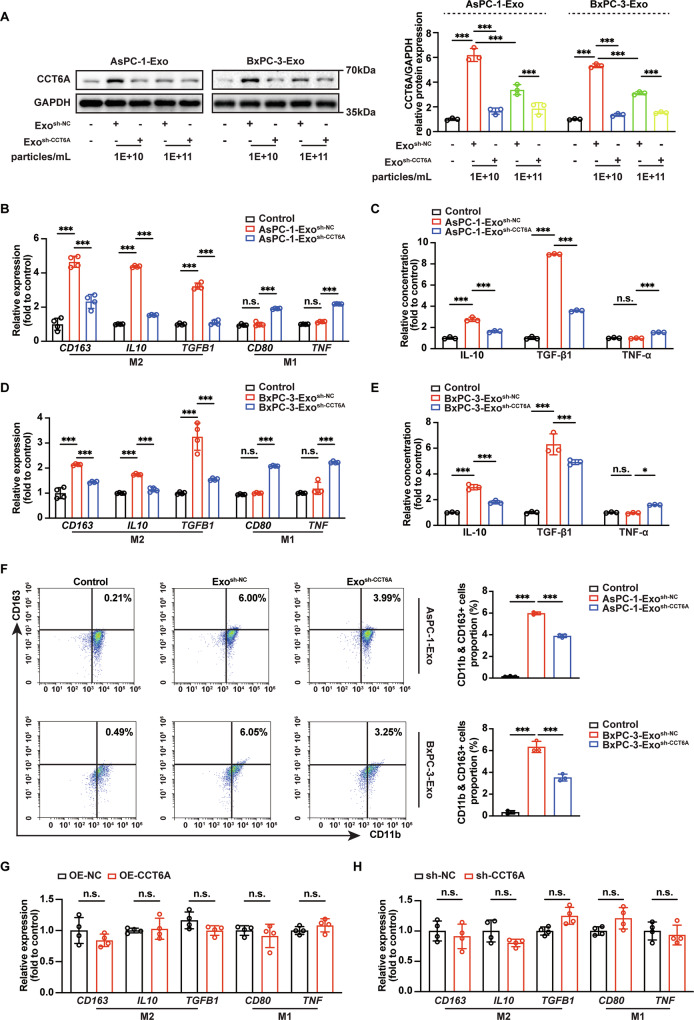
Uptake of exosomal CCT6A induces M2 polarization non-directly in macrophages. **A** Exosomes at different concentrations were directly added to the macrophage culture medium, resulting in final exosome concentrations of 1 × 10¹⁰ particles/mL and 1 × 10¹¹ particles/mL. Analysis of CCT6A levels in PDAC-derived exosomes-treated macrophages through western blotting (left). Quantification (right) of CCT6A/GAPDH level in macrophages treated with the exosomes from CCT6A-silenced cells (Exo^sh-CCT6A^) versus from control cells (Exo^sh-NC^). (*n* = 3). Two-way ANOVA analysis. **B** RT-qPCR of M2 and M1 markers in macrophages treated with an equal volume of saline without exosomes (Control), AsPC-1-Exo^sh-NC^ or AsPC-1-Exo^sh-CCT6A^. (*n* = 3). Two-way ANOVA analysis. **C** ELISA analysis for IL-10, TGF-β1, and TNF-α in macrophages treated with different AsPC-1-Exo groups. (*n* = 4). Two-way ANOVA analysis. **D** RT-qPCR of M2 and M1 markers in macrophages treated with Control, BxPC-3-Exo^sh-NC^ or BxPC-3-Exo^sh-CCT6A^. *n* = 4. Two-way ANOVA analysis. **E** ELISA analysis for IL-10, TGF-β1, and TNF-α in macrophages treated with different BxPC-3-Exo groups. (*n* = 3). Two-way ANOVA analysis. **F** Flow cytometry analysis of M2-type (CD11b^+^CD163^+^) macrophages’ distribution after exosomes treatment (left). Quantification (right) of M2-type macrophages. (*n* = 3). One-way ANOVA analysis. RT-qPCR of M2 and M1 markers in macrophages treated with CCT6A-expressing plasmid (**G**) or CCT6A shRNA-expressing (**H**). (*n* = 4). Two-way ANOVA analysis. Data presented as mean ± SD. n.s., no significant, *p ≤ 0.05, **p ≤ 0.01, ***p ≤ 0.001.

Following 24 h of exosome incubation, CCT6A levels significantly elevated in macrophages treated with the exosomes from control cells (Exo^sh-NC^) at a concentration of 1 × 10^10^ particles/mL, while slightly increased with the exosomes from CCT6A-silenced cells (Exo^sh-CCT6A^) (Fig. 3A). Treated with exosomes of 1×10^10^ particles/mL, macrophages were analyzed using RT-qPCR, ELISA, and flow cytometry. As CCT6A levels in PDAC-derived exosomes decreased, there was a corresponding downregulation of M2 macrophage markers and an upregulation of M1 markers in the treated macrophages (Figs. 3B–F and S6E). However, transfection of macrophages with CCT6A-expressing or CCT6A shRNA-expressing plasmid did not directly induce significant polarization towards either the M2 or M1 phenotype (Fig. 3G–H). Collectively, these findings suggest that exosomal CCT6A can modulate macrophage phenotypes, albeit in an indirect manner.

### Uptake of CCT6A-containing exosomes activates the PI3K-AKT pathway to drive alterations in the macrophage phenotype

**Fig. 4:**
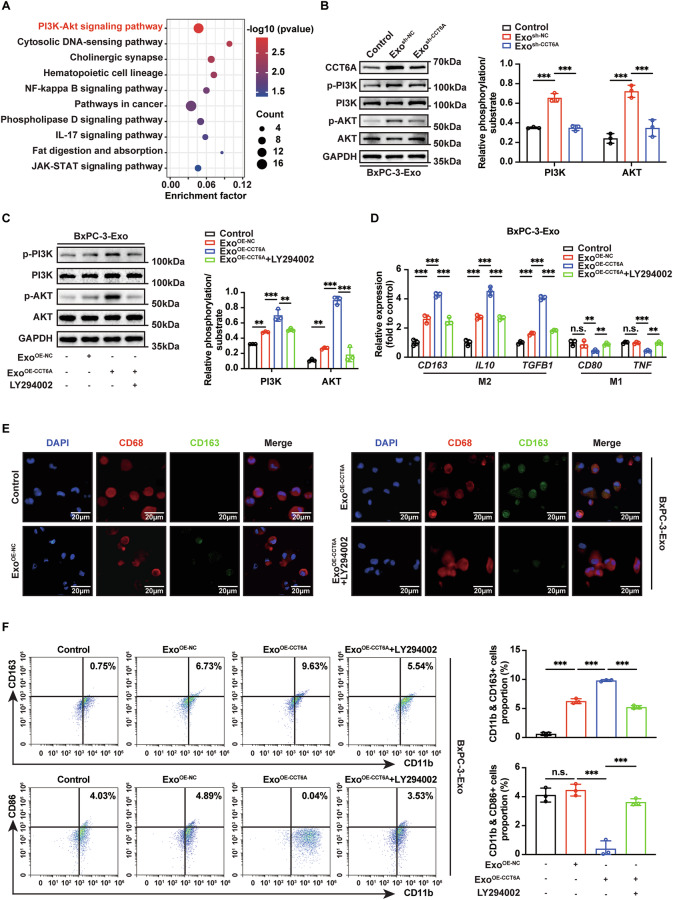
Uptake of exosomal CCT6A induces M2 polarization via activating PI3K-AKT signaling pathway. **A** KEGG enrichment analysis of DEGs between Exo^sh-NC^ and Exo^sh-CCT6A^ group. **B** Detection of PI3K-AKT signaling in Control (an equal volume of saline without exosomes), Exo^sh-NC^, and Exo^sh-CCT6A^ treated macrophages via western blotting (left). Quantification (right) of p-PI3K/PI3K and p-AKT/AKT levels. (*n* = 3). Two-way ANOVA analysis. **C** Detection of PI3K-AKT signaling in Control, BxPC-3-Exo from control cells (BxPC-3-Exo^OE-NC^), BxPC-3-Exo isolated from CCT6A-overexpressing cells (BxPC-3-Exo^OE-CCT6A^), or BxPC-3-Exo^OE-CCT6A^ with PI3K inhibitor LY294002 administration (BxPC-3-Exo^OE-CCT6A^ + LY294002) treated macrophages via western blotting (left). Quantification (right) of p-PI3K/PI3K and p-AKT/AKT levels. (*n* = 3). Two-way ANOVA analysis. **D** RT-qPCR of M2 and M1 markers in macrophages treated with different BxPC-3-Exo groups. (*n* = 3). Two-way ANOVA analysis. **E** Representative images of IF staining for CD68 (red) and CD163 (green) in macrophages treated with different BxPC-3-Exo groups. Scale bar, 20 μm. **F** Flow cytometry analysis for distribution of M2-type (CD11b^+^CD163^+^) and M1-type (CD11b^+^CD86^+^) macrophages after different BxPC-3-Exo treatments (left). Quantification (right) of M2-type and M1-type macrophages. *n* = 3. One-way ANOVA analysis. Data presented as mean ± SD. n.s. no significant, *p ≤ 0.05, **p ≤ 0.01, ***p ≤ 0.001.

To elucidate the mechanisms by which exosomal CCT6A influences macrophage phenotypes, we performed transcriptome profiling on exosome-treated macrophages. KEGG pathway analysis of DEGs revealed a significant enrichment of the PI3K-AKT signaling pathway (Figs. 4A, and S7A, B), a pathway known for its critical role in regulating macrophage polarization and shaping the tumor immune microenvironment [25]. Western blotting analysis further showed that the Exo^sh-NC^ group exhibited significantly increased phosphorylation of PI3K and AKT compared to the Saline and Exo^sh-CCT6A^ groups (Fig. 4B). These results suggest that exosomal CCT6A may regulate the PI3K-AKT signaling pathway in macrophages.

To confirm whether exosomal CCT6A induces M2 macrophage polarization via the PI3K-AKT pathway, we used the PI3K inhibitor LY294002. PDAC cells were transfected with a CCT6A-expressing vector or an empty vector as control (Fig. S8A). In line with the observation that LY294002 treatment effectively inhibited exosomal CCT6A-induced phosphorylation of PI3K and AKT (Fig. 4C), the results from RT-qPCR, IF, and flow cytometry demonstrated that the M2-promoting effects of exosomes with elevated CCT6A expression were completely abrogated following LY294002 administration (Figs. 4D–F and S8B–D). These findings strongly suggest that exosomal CCT6A promotes M2-like phenotypic alterations in macrophages by modulating the PI3K-AKT signaling pathway.

### Exosomal CCT6A activates the PI3K/AKT pathway in a chemokine-dependent manner and alters the macrophage phenotype

**Fig. 5:**
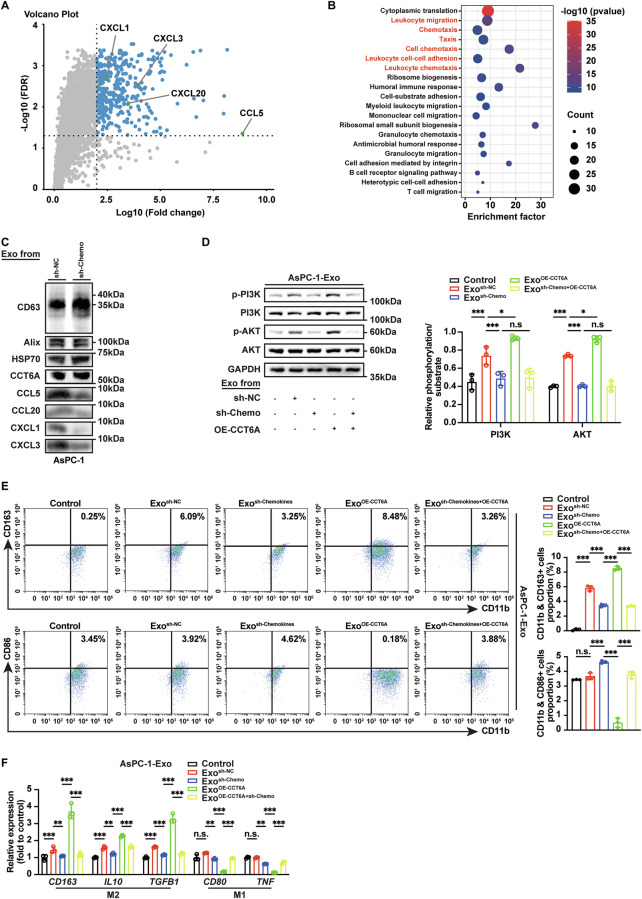
Exosomal CCT6A induces M2 polarization though increasing exosomal chemokines levels. **A** Volcano plot of DEPs between Exo^sh-NC^ and Exo^sh-CCT6A^ groups. **B** KEGG enrichment analysis of DEPs between Exo^sh-NC^ and Exo^sh-CCT6A^ group. **C** Validation of the exosomal chemokines knockdown efficacy in AsPC-1-Exo (AsPC-1-Exo^sh-Chemo^) via western blotting. **D** Detection of PI3K-AKT signaling in Control (an equal volume of saline without exosomes), AsPC-1-Exo^sh-NC^, AsPC-1-Exo^sh-Chemo^, AsPC-1-Exo^OE-CCT6A^, or exosomes isolated from AsPC-1 cells with downregulated chemokines and overexpressed CCT6A (AsPC-1-Exo^sh-Chemo+OE-CCT6A^) treated macrophages via western blotting (left). Quantification (right) of p-PI3K/PI3K and p-AKT/AKT levels. (*n* = 3). Two-way ANOVA analysis. **E** Flow cytometry analysis for distribution of M2-type (CD11b^+^CD163^+^) and M1-type (CD11b^+^CD86^+^) macrophages after different AsPC-1-Exo treatments (left). Quantification (right) of M2-type and M1-type macrophages. (*n* = 3). One-way ANOVA analysis. **F** RT-qPCR of M2 and M1 markers in macrophages treated with different AsPC-1-Exo groups. (*n* = 3). Two-way ANOVA analysis. Data presented as mean ± SD. n.s. no significant, *p ≤ 0.05, **p ≤ 0.01, ***p ≤ 0.001.

Collectively, we hypothesized that the elevated levels of exosomal CCT6A may induce M2 polarization in macrophages, via modulating exosomal compositions. To address this, 4D-DIA proteomic analysis of Exo^sh-NC^ and Exo^sh-CCT6A^ revealed chemokines (CXCL1, CXCL3, CCL20, CCL5) and their related pathways were notably enriched, among the most significantly downregulated proteins in Exo^sh-CCT6A^ (Figs. 5A, B, and S9A–C). These chemokines were well-documented for their roles in activating the PI3K-AKT pathway and subsequently inducing M2-like phenotypes in macrophages [26, 27].

Western blotting results showed a marked reduction in these chemokine levels within Exo^sh-CCT6A^ exosomes (Fig. S10A). Conversely, when we downregulated CXCL1, CXCL3, CCL20, and CCL5 expression in PDAC cells (sh-Chemo) using shRNA, chemokine levels in the exosomes decreased without affecting CCT6A levels, indicating that changes in chemokine expression do not influence exosomal CCT6A levels (Fig. 5C). Although changes in CCT6A affect other TRiC subunits (Fig. S10B), the levels of chemokines in exosomes with low expression of CCT3 or CCT8 (subunits adjacent to CCT6A) were not affected (Fig. S10C–E). Endogenous co-immunoprecipitation (Co-IP) showed that CCT6A interacts with neighboring subunits in exosomes (Fig. S10F). These results suggest that while CCT6A is not alone in exosomes, it has a unique role in regulating chemokines compared to other subunits. Moreover, when chemokine levels in exosomes were effectively reduced, exosomes derived from CCT6A-overexpressing PDAC cells lost their ability to promote PI3K signaling and M2 polarization, as shown in rescue experiments (Fig. 5D–F).

Given that CCL5 exhibited the most significant changes and has been directly reported to activate the PI3K-AKT pathway in regulating TAMs, we conducted additional rescue experiments to explore its specific function. The results demonstrated that CCL5 overexpression restored the ability of Exo^sh-CCT6A^ to promote PI3K signaling and M2 polarization in macrophages (Fig. S10G–I).

These findings strongly suggest that exosomal CCT6A modulates macrophage phenotype by upregulating exosomal chemokine levels, thereby activating the PI3K/AKT signaling pathway.

### CCT6A serves as a matchmaker to introduce the chemokines from PDAC cells to TAMs

**Fig. 6:**
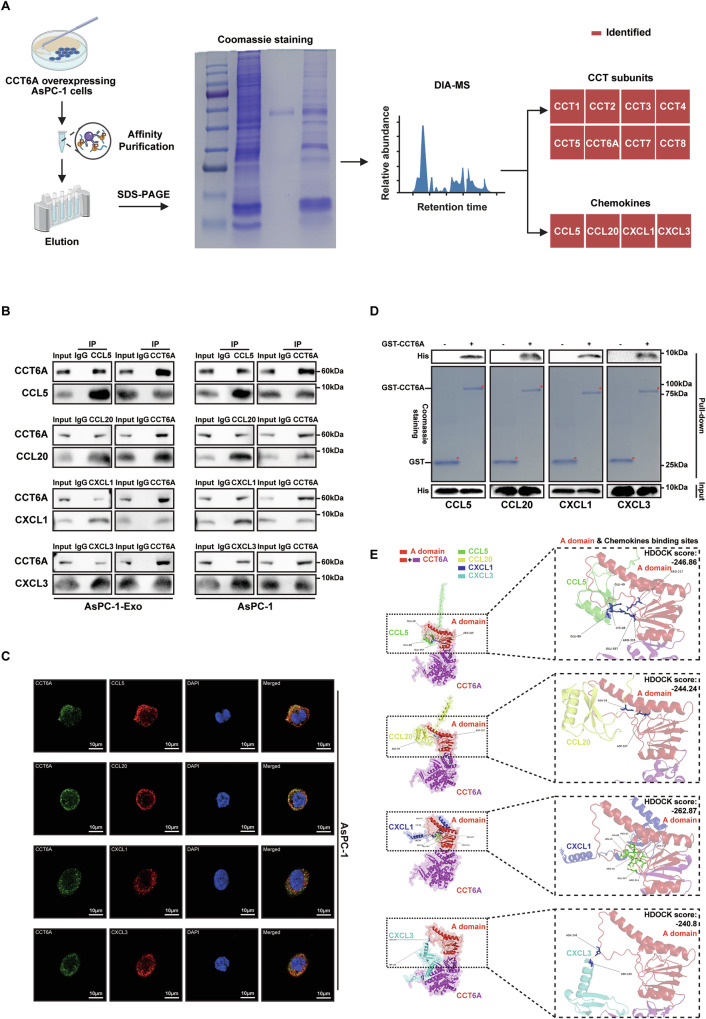
CCT6A mediates the interaction between chemokines from PDAC cells and TAMs. **A** Schematic diagram of experimental design utilizing interactome analysis to identify proteins potentially binding to CCT6A. Created with BioRender.com. **B** Detection of endogenous interaction between CCT6A and chemokines in AsPC-1-Exo (left) and AsPC-1 cells (right) via Co-IP and western blotting. **C** Representative Confocal micrographs of CCT6A (green) and chemokines (red) in AsPC-1 cells. Scale bar, 10 μm. **D** GST pulldown assays performed by western blotting or Coomassie Brilliant Blue (CBB) staining to detect direct binding of CCT6A and chemokines in vitro. **E** Molecular docking and HDOCK scores of CCT6A with four chemokines.

CCT6A is a critical subunit of the TRiC complex, responsible for the folding and maturation of approximately 10% of newly synthesized proteins within the cell. Given that different TRiC subunits exhibit substrate specificity, we hypothesize that CCT6A may act as the specific binding subunit for these chemokines, thereby facilitating their incorporation into exosomes. To test this hypothesis, we conducted interactome analysis to identify proteins interacting with CCT6A. Among the identified binding partners, eight CCT subunits were detected, confirming the reliability of the interactome (Fig. 6A). Notably, all decreased exosomal chemokines were identified as interacting proteins (Fig. 6A). Endogenous Co-IP further supported the binding of these chemokines with CCT6A in both PDAC cells and its-derived exosomes (Fig. 6B). Immunofluorescence staining demonstrated the colocalization of endogenous CCT6A and chemokines within PDAC cells (Fig. 6C). Additionally, GST pulldown assays revealed a direct interaction between CCT6A and the four chemokines (Fig. 6D). Additionally, molecular docking analysis revealed that these chemokines exhibit a high affinity for the substrate-binding interface of CCT6A (Hdock score < -240) (Fig. 6E). Among them, CCL5, which showed the most significant reduction in Exo^sh-CCT6A^, demonstrated the strongest binding capacity to the substrate-binding interface of the CCT6A subunit (Fig. S11A). Correspondingly, the GST pulldown assay results further demonstrated that other TRiC subunits did not directly interact with CCL5 (Fig. S11B). In conclusion, we hypothesize that CCT6A functions as a chaperone to these chemokines at an early stage of their synthesis, as a matchmaker facilitating their incorporation into PDAC-derived exosomes. Upon uptake by TAMs, the exosomal chemokines subsequently activate their M2 polarization.

### CCT6A silencing promotes the therapeutic effect of an anti-CD47 nanobody on PDAC tumors and alters the TAM phenotype

**Fig. 7:**
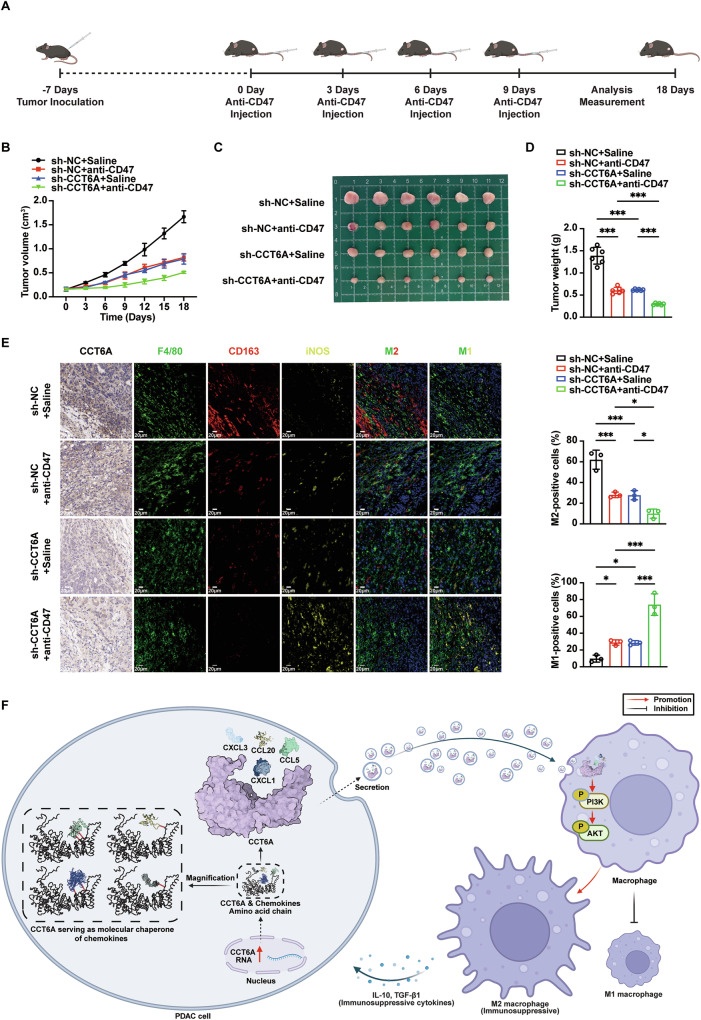
Tumoral CCT6A levels as a prognostic indicator for anti-CD47 antibody immunotherapy in PDAC. **A** Schematic diagram showing the tail vein injection of anti-CD47 nanobody into mice after subcutaneous injection of CCT6A-silenced or control KPC cells. (*n* = 6). Created with BioRender.com. **B** Tumor volume curve. (*n* = 6). Black: subcutaneous injection of control KPC cells and tail vein injection of saline (sh-NC + Saline), red: subcutaneous injection of control KPC cells and tail vein injection of anti-CD47 nanobody (sh-NC + anti-CD47), blue: subcutaneous injection of CCT6A-silenced KPC cells and tail vein injection of saline (sh-CCT6A + Saline), and green: subcutaneous injection of CCT6A-silenced KPC cells and tail vein injection of anti-CD47 nanobody (sh-CCT6A + anti-CD47). (*n* = 6). General images (**C**) and tumor weights (**D**) of subcutaneous tumors. (*n* = 6). **E** IHC analysis of CCT6A and tyramide signal amplification (TSA) -multiplex IF (mIF) staining (left) of F4/80 (green), CD163 (red) and iNOS (yellow) in the tumor sections. Scale bar, 20 μm. M2 (F4/80^+^CD163^+^) and M1 (F4/80^+^iNOS^+^) -positive cell count statistics (right) of the tumor sections. (*n* = 3). **F** Graphical abstract. Created with BioRender.com. Data presented as mean ± SD. n.s. no significant, *p ≤ 0.05, **p ≤ 0.01, ***p ≤ 0.001. One-way ANOVA analysis.

Eventually, a mouse subcutaneous tumor model was established to assess whether our findings could inform immunotherapy strategies (Fig. 7A). Mouse pancreatic cancer cells (KPC cells) were stably transfected with either CCT6A-targeting shRNA or scramble shRNA (Fig. S12A). An anti-CD47 nanobody, previously developed in our lab [15], was utilized for treatment. Notably, both the tumor growth curve and final tumor weight measurements demonstrated that the anti-CD47 nanobody achieved a more effective therapeutic outcome in tumors with CCT6A knockdown, compared to the scramble control group (Fig. 7B–D). Additionally, comprehensive analysis of pathological staining revealed that anti-CD47 nanobody therapy more effectively reduced the infiltration of F4/80^+^CD163^+^ TAMs and increased the infiltration of F4/80^+^CD86^+^ TAMs in pancreatic cancer with CCT6A knockdown (Fig. 7E). These results suggest that our findings may provide valuable insights for guiding the application of immunotherapy in PDAC treatment.

## Discussion

In this study, we found the PDAC-derived exosomes enhance PDAC progression via inducing M2 polarization of macrophages, other than accelerating tumor proliferation. Through proteomics analysis of PDAC-derived exosomes, we identified CCT6A as a key exosomal protein, linking PDAC burden and M2 macrophage phenotype together. Exosomes derived from CCT6A^high^ PDAC cells induced the greater M2 phenotype in macrophages. Transcriptomic analysis and rescue experiments revealed that exosomal CCT6A activated the PI3K-AKT signaling pathway and therefore led to M2-towards polarization. The top decreased proteins in CCT6A-silenced PDAC-derived exosomes were primarily concluded into chemokine. Interactomics data supports CCT6A binds with these chemokines in PDAC cells. Moreover, we demonstrated that PDAC-derived exosomes expression counteracted the efficacy of anti-CD47 immune therapy, which was cancelled by CCT6A silencing. Since CCT6A is the subunit of TRiC chaperone complex, we proposed the PDAC-derived exosomal CCT6A serves as matchmaker, exporting PDAC cells-originated chemokines to cause TAMs M2 polarization. These mechanistic insights contribute to a better understanding of PDAC and the therapeutic strategies development for PDAC patients.

In PDAC TME, macrophages play a central role, of which M2-polarized macrophages populate the most [28]. Many studies have confirmed that M2-polarized macrophages are closely related to the progression, metastasis, and drug resistance of PDAC. For example, M2-polarized macrophage-derived exosomes promote angiogenesis and growth of PDAC [29, 30]. Moreover, single-cell sequencing analysis showed that M2-polarized TAMs are the major source of immunosuppressive CD86 and PD-L1 in PDAC, which infers that M2-polarized macrophages are responsible for the effector T cells exhaustion in PDAC TME [31]. The PDAC cells can stimulate M2-polarized macrophages via the paracrine communication [11]. Exosomes mediate the material transfer and information communication between cells, as lipid double-coated extracellular vesicles containing proteins, nucleic acids and metabolites [32]. Our study revealed that PDAC-derived exosomal CCT6A functions as matchmaker to efficiently introduce chemokines into TAMs, which leads to increased M2-polarized macrophages.

The importance of exosomes in the TME was well recognized. They serve as crucial factors in intercellular communication, promoting treatment resistance within TME [33, 34]. Thus, exosome-based treatment displayed potential prospect in alleviating immunosuppressive TME. For instance, blocking the secretory transmission of tumor exosomes leads to the loss of exosome-delivered signaling molecules, which results in the reversal of the adverse reprogramming of tumor-associated stromal cells [35]. Furthermore, our studies previously demonstrated engineered exosomes’ potential for drug delivery, which effectively reverses the M2-polarized macrophages to M1-polarized phenotype and thereby shows anti-tumor therapeutic efficacy [36]. In the current research, we demonstrated silencing CCT6A abrogated the resistance of PDAC-derived exosomes to anti-CD47 antibody immunotherapy. Exosomal CCT6A may be a promising target to improve the immunosuppressive TME of PDAC.

The CCT chaperones are cytoprotective, but their abnormality may cause cancer [37]. For example, CCT2, CCT3, CCT4, and CCT5 were increased in breast cancer cells and positively correlated with tumor progression [38]. Another in silico study demonstrated a direct correlation between the increase in tumoral transcriptional levels of all CCT subunits, except CCT6B, with bad prognosis [39]. Moreover, studies with glioblastomas demonstrated CCT6A, CCT1, CCT2, and CCT7 subunits increased in the tumor tissue and tumor-derived extracellular vesicles, particularly CCT6A [40]. In our findings, CCT6A was the sole TRiC subunit, whose pathogenic increase in the PDAC tissue and PDAC-derived exosomes reinforces the immunosuppressive TME.

Exosomes are critical in PDAC TME. However, most investigations about exosomes-mediated material transport and intercellular communication focused on the microRNA and lncRNA, rarely proteins [41–43]. Plenty of studies have shown that TAMs-derived exosomes cause PDAC cells reprogramming and subsequent tumor progression [29, 30]. However, little is known about the impacts of PDAC-derived exosomes on TAMs. In this study, we clarified the pathogenic roles of exosomes from PDAC in activating M2 TAMs for the first time.

This study has limitations that must be acknowledged. Additional studies are required to reveal the specific role of exosomal CCT6A in the modulation of the PI3K/AKT pathway. In addition, there is a need for in-depth research on the mechanism underlying combined treatment involving CCT6A silencing and anti-CD47 nanobodies.

## Supplementary information

clean version of the supplementary materials

Original western blots

## Data Availability

All data are available in the main text or the Supplementary materials. All RNA-seq and proteomics datasets have been made publicly accessible in open repositories. RNA-seq data are deposited on the National Center for Biotechnology Information (NCBI); accession numbers GSE278339 and GSE278909. The mass spectrometry proteomics data are stored with the ProteomeXchange Consortium via the PRIDE partner repository, with the dataset identifier PXD056403 and PXD057101. The public datasets utilized in this study include TCGA-PAAD, GSE211398, GSE15471, GSE28753, and GSE62165.
