# Supplementary material for: Tumor-derived exosomal CCT6A serves as a matchmaker introducing chemokines to tumor-associated macrophages in pancreatic ductal adenocarcinoma
Source: Cell Death Dis. 2025 May 15;16(1):382. doi: 10.1038/s41419-025-07720-y (PMC12081750; doi:10.1038/s41419-025-07720-y)
Supplement: Supplementary file 1 — clean version of the supplementary materials [file 41419_2025_7720_MOESM1_ESM.docx]

Supplementary Materials for

**Tumor-Derived Exosomal CCT6A Serves as a Matchmaker Introducing Chemokines to Tumor-Associated Macrophages in Pancreatic Ductal Adenocarcinoma**

Tianyin Ma *et al.*

*Corresponding author. Email: yangzhiyong@whu.edu.cn (Z.Y.);

mawj1990@whu.edu.cn (W.M.);

dr.med.mingtian@whu.edu.cn (M.T.);

yuanyf1971@whu.edu.cn (Y.Y.)

**This PDF file includes:**

Materials and Methods

Figs. S1 to S12

Tables S1

Materials and Methods

Cell culture and transfection

Human PDAC cell lines (AsPC-1; CFPAC-1; BxPC-3; Capan-1 and Capan-2) and the human monocyte line THP-1 were purchased from Procell Life Science & Technology Co., Ltd. The mouse pancreatic cell line KPC was a gift from the Shenzhen Clinical Research Centre for Respirology. Capan-2 cells were cultured in McCoy’s 5A medium supplemented with 10% FBS. CFPAC-1 and Capan-1 cells were grown in Iscove's modified Dulbecco’s medium (IMDM) supplemented with 10% FBS. BxPC-3 and AsPC-1 cells were grown in RPMI 1640 medium supplemented with exosome-depleted 10% FBS (XP Biomed). KPC cells were cultured in DMEM supplemented with 10% FBS. THP-1 cells were grown in RPMI 1640 medium supplemented with 0.05mM mercaptoethanol and 10% FBS. All these cell lines have recently been authenticated via Short Tandem Repeat (STR) profiling to confirm their origin and genetic consistency. They were cultured and maintained at 37°C in a 5% CO₂ atmosphere and were regularly tested negative for mycoplasma contamination. THP-1 cells were stimulated with phorbol-12-myristate-13-acetate (PMA) (100ng/mL, MedChemExpress) for M0 macrophages differentiation. M0 macrophages co-cultured with PDAC cells or exosomes were treated with exosome secretion inhibitor GW4869 (20μM, MedChemExpress) or PI3K inhibitor LY294002 (10μM, MedChemExpress) for 24h. After incubation for the specified time, cells or exosomes from each group were collected for further testing. For transfection of cells with shRNAs or overexpression plasmids, GeneMute (SignaGen Laboratories) was used following the manufacturer's instructions. Stably transfected cells were selected with phleomycin. Except for the shRNAs targeting CCT6A, CCT3, and CCT8, which were designed (**Table S1**) and cloned into plasmids, the remaining shRNAs and overexpression plasmids were obtained from Miaoling Biotech.

Transwell-based indirect co-culture

THP-1 cell-derived M0 macrophages were co-cultured with PDAC cell lines (AsPC-1 and BxPC-3) in Transwell chambers (0.4μm, Corning). The PDAC cells were seeded in the upper chamber, and the macrophages were seeded in the lower chamber. The ratio of macrophage to PDAC cells was 1:1, with the following specific cell numbers: the experiment was conducted using a 12-well plate, with both macrophages and PDAC cells seeded at 150,000 cells per well, with a cell concentration of 150,000 cells/mL. After 48 hours of co-culture, the subsequent experiments were performed.

Antibodies and fluorescent antibodies

Primary antibodies specific for CD80 (66406-1-Ig), CD163 (16646-1-AP), GAPDH (10494-1-AP), CCT6A (19793-1-AP), CCT3 (10571-1-AP), CCT8 (12263-1-AP), Alix (12422-1-AP), CD63 (25682-1-AP), Hsp70 (25405-1-AP), Calnexin (10427-2-AP), PI3K (20584-1-AP), AKT (10176-2-AP), p-AKT (66444-1-Ig), CCL5 (12000-1-AP), CCL20 (26527-1-AP), CXCL1 (12335-1-AP), CXCL3 (11221-1-AP), His (HRP-66005) and the secondary antibodies (SA00001-1& SA00001-2) were obtained from Proteintech Group. The anti-p-PI3K antibody (bs-6417R) was purchased from Bioss. Anti-Ki67 (GB121141-100), anti-F4/80 (GB113373-100) and anti-iNOS (GB115703-100) were purchased by Servicebio. The fluorescent primary antibodies were anti-CD11b (VdoBiotech), anti-CD163 (Proteintech, CL488-65169) and anti-CD86 (Proteintech, APC-65165). The fluorescent secondary antibodies were Cy3-conjugated (Proteintech, SA00009-1& SA00009-2) and FITC-conjugated (Proteintech, SA00003-1& SA00003-2).

Western blotting analysis

Total protein was extracted in RIPA buffer, and the total protein concentration was measured via a BCA assay. Next, the extracted proteins were separated via 10% SDS‒PAGE and transferred onto a PVDF membrane (Millipore). This membrane was blocked with 5% nonfat dry milk for 2 hours, followed by overnight incubation with primary antibodies at 4°C. Secondary antibodies were added to the membrane after washing. Uncropped Protein bands were visualized with ECL detection reagents (GE Healthcare), and band intensities were quantified via ImageJ software. For the detection of specific exosomal biomarkers, exosomes were randomly selected from different batches that had been previously extracted and stored at -80°C.

Exosome isolation

Differential centrifugation was carried out to isolate exosomes from cell culture media. Initially, the mixture was subjected to 10min of centrifugation at 300g, followed by 20min of centrifugation at 2000g to remove larger pieces of cellular debris. The macrovesicles were then isolated by centrifugation for 30min at 10,000g. The supernatants were filtered through a 0.22μm filter. Further purification was achieved by centrifuging the filtered supernatant for 70min at 120,000g. Finally, the resultant pellet was resuspended in saline and subjected to an additional 70min of centrifugation at 120,000g for further purification. The pelleted exosomes were resuspended in saline and stored at -80°C for subsequent applications. All centrifugation procedures were conducted at 4°C. Exosome quantification was conducted with the Micro BCA Protein Assay Kit (Thermo Fisher Scientific). In this study, neither shRNA nor overexpression plasmids were directly transfected into exosomes. Exo^sh^ or Exo^OE^ were derived from PDAC cell lines treated with shRNA or overexpression plasmids, and both were extracted following the above methods.

Transmission electron microscopy (TEM)

The TEM was utilized to characterize the exosomes. Purified exosomes, suspended in PBS, were added onto copper grids, stained with a 2% uranyl acetate solution and dried under an infrared heat lamp. They were then analyzed using a TEM (Thermo Fisher Scientific, Talos L120C G2, USA).

Cryo-electron microscope (Cryo-EM)

Exosomes, which were solubilized in 20μL PBS, were applied to a glow-discharged copper grid. The grid was then blotted for 3 seconds with a force of 3 maintained in the chamber humidity of 100% at 8℃. The prepared grid was immediately emersed in liquid ethane, cooled by liquid nitrogen using a Vitrobot, FEI. Images were acquired using the Glacios 200kV transmission electron microscope (Thermo Fisher Scientific, Glacios, USA) equipped with a Falcon 4 direct electron detector.

Atomic force microscopy (AFM)

To examine the morphological characteristics of exosomes, samples were diluted at a 1:1000 ratio in deionized water and applied to freshly cleaved mica sheets. After rinsing with deionized water, the specimens were gently dried using a nitrogen stream. Surface morphology analysis was conducted using an atomic force microscope (Shimadzu, SPM-9700HT, JPN) in tapping mode. The analysis employed a silicon probe (OMCL-AC240TS-R3, OLYMPUS, resonant frequency: 70kHz, spring constant: k~1.7 N/m).

Dynamic Light Scattering (DLS)

The DLS technology was employed to evaluate the Brownian motion velocity of particles to determine their hydrodynamic diameters. To examine the initial particle size and potential distribution of exosomes based on their Brownian motion, exosomes were diluted with deionized water, and their particle size and potential were determined using the Zetasizer Nano ZSP (Malvern, UK).

Nanoparticle tracking analysis (NTA)

Exosome enumeration and size measurements were performed using NTA with the ZetaView Particle Metrix instrument (PMX-120, Germany). Prior to usage, the instrument was calibrated with 100nm polystyrene beads (Thermo Fisher Scientific, Fremont, CA). The NTA software was employed to determine the concentration of nanoparticles (particles/mL). To streamline the process, the batch processing feature in the NTA software was utilized for measuring individual samples.

Resistive pulse sensing (RPS)

To precisely assess the exosome particle size distribution and potential, measurements were conducted using the NanoCoulter counter, based on the Beckman Coulter principle. The NanoCoulter counter (Resun Technology, Co., Ltd., Shenzhen) had been found to be as effective as an electron microscope, equipped with a custom chip tailored to the specific measuring range required for analyte detection.

RNA isolation and analysis of gene expression via RT-qPCR

Total RNA was isolated with TRIzol reagent (Takara) and then transcribed into cDNA with an enzyme kit (Vazyme). RT-qPCR was conducted on a LightCycler96 Real-time PCR System (Roche) with 2 × ChamQ Universal SYBR qPCR Master Mix (Vazyme). **Table S1** shows the sequences of all the primers used for PCR.

IF

Cells were cultured and allowed to spread on slides before fixation with 4% paraformaldehyde (PFA) for 30min. After permeabilization for 10min with 0.1% Triton X-100, blocking was performed for 20min with NP-40 in saline. Slides were incubated overnight at 4°C with primary antibodies, followed by saline washing and a 1-hour incubation with fluorescent secondary antibodies.

RNA-seq analysis

Total RNA samples were obtained and mRNAs with poly (A) tails were enriched using oligo (dT) beads. The mRNA was then fragmented, and first- and second-strand cDNA were synthesized using random primers. The cDNA underwent end-repair, 3’ adenylation, and adaptor ligation, followed by PCR amplification and purification. Library quality was assessed, circularized, and amplified to form DNA nanoballs (DNBs). Sequencing was performed on the DNBSEQ platform using DNBSEQ Technology.

Raw data with adapter sequences or low-quality sequences were filtered using SOAPnuke developed by BGI with the following parameters: “-n 0.001 -l 20 -q 0.4 --adaMR 0.25 --polyX 50 --minReadLen 150”. Filtering steps included removing reads matching over 25.0% of adapter sequences, reads shorter than 150 bp, reads with over 0.1% N content, reads with polyX lengths exceeding 50 bp, and reads with over 40.0% of bases having a quality value below 20. Clean reads were obtained with the quality value system set to Phred+33. The cleaned reads were then aligned to the reference genome GRCm39 using the STAR v2.7.11b aligner with default parameters. Gene counts were extracted from STAR ReadsPerGene.out.tab files.

Public datasets analysis

To identify upregulated genes in PDAC tumors, we used five public PDAC datasets: TCGA-PAAD, GSE211398, GSE15471, GSE28753, and GSE62165. The TCGA-PAAD dataset was merged with the GTEx normal pancreas dataset. Each dataset was individually analyzed to identify upregulated genes in tumor samples compared to paratumor or normal pancreas samples using the limma R package. The p-value cutoff was set to 0.01 for array data and 0.001 for transcriptome data. After analyzing each dataset, we intersected the upregulated genes and identified 937 upregulated genes.

Immune Infiltration Analysis

Immune cell infiltration in each tumor sample from the aforementioned five datasets was analyzed using the “CIBERSORT” and “TIMER” algorithms through the R package IOBR. These tools deconvolute the RNA-seq data to estimate the proportions of various immune cell types within the samples. We then screened for genes significantly associated with the estimated “TIMER macrophages” (r > 0.5), “CIBERSORT M0 macrophages” (r > 0.5), “CIBERSORT M1 macrophages” (r < -0.3), and “CIBERSORT M2 macrophages” (r > 0.3). Finally, we identified 296 genes significantly associated with our candidate macrophage-associated genes.

4D-DIA quantitative proteomics

Samples were retrieved from the -80°C freezer, and an appropriate amount of lysis buffer (8M urea, 1mM PMSF, and 2mM EDTA) was added. Perform ultrasonic lysis for 5 minutes on ice, followed by centrifugation at 15,000 g at 4°C for 10 minutes to collect the supernatant. Subsequently, determine the protein concentration using a BCA assay kit. Liquid chromatography (LC) was conducted on a nanoElute UHPLC (Bruker Daltonics). MS raw data were analyzed using DIA-NN (v1.8.1) with a library-free approach. The uniprotkb_proteome_UP000005640_human_82493_20240528.fasta database (totaling 82,493 sequences) was utilized to generate a spectral library using deep learning algorithms based on neural networks. The Match Between Runs (MBR) option was employed to create a spectral library from the DIA data, which was then reanalyzed using this library. The false discovery rate (FDR) of the search results was adjusted to < 1% at both the protein and precursor ion levels. The remaining identifications were used for subsequent quantification analysis. Differentially expressed proteins were selected using the following criteria: p ≤ 0.05 and FC ≥ 1.5 or F ≤ 0.6667. The enrichment analysis was conducted using clusterProfiler (4.10.0).

Direct co-culture

After extracting exosomes from PDAC cells, 100µL of exosome suspension, diluted based on the NTA results, was added to each well of the macrophage culture medium (using 24-well plates, with 600µL of medium per well). The final exosome concentrations in the medium were as follows: 1×10¹¹ particles/mL for the addition of 100µL of the stock solution; 1×10¹⁰ particles/mL for the addition of 100µL of the diluted solution (the stock solution was diluted 10 times). Fresh exosomes from the same extraction batch were used in this assay to ensure consistency.

Interactome analysis

Co-IP experiments were performed in CCT6A overexpressing AsPC-1 cells, followed by SDS-PAGE, 4D-DIA quantification, and mass spectrometry analysis to identify proteins interacting with CCT6A.


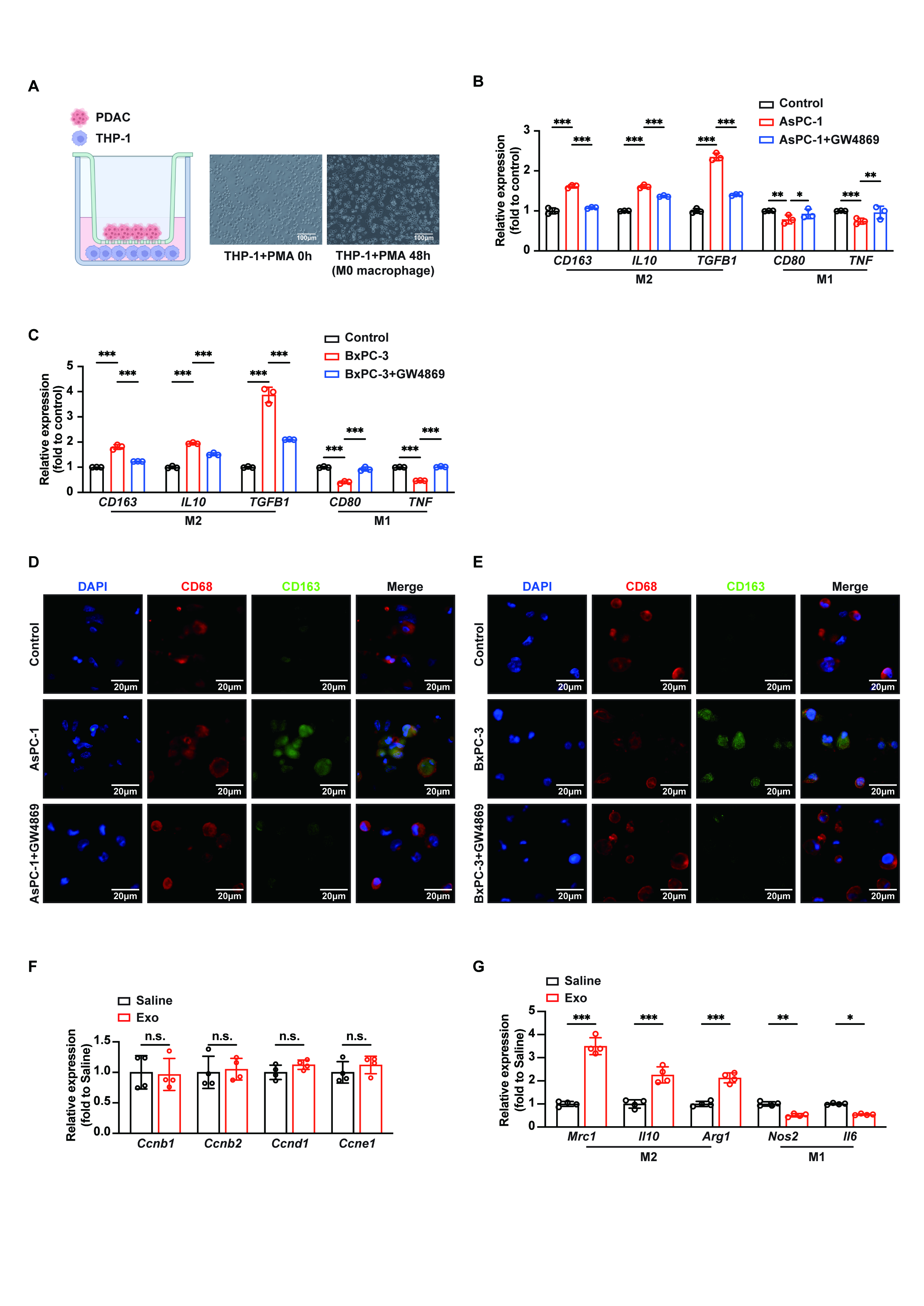


Fig. S1. Macrophages undergo M2 polarization in response to exosomes secreted by PDAC cell lines in vitro and in vivo

**(A**) Schematic diagram of the indirect co-culture (left), illustrating the 48h co-culture of THP-1 cell-derived M0 macrophages and PDAC cells in Transwell chambers (0.4μm). The PDAC cells are placed in the upper chamber, and the macrophages are in the lower chamber. Created with BioRender.com. Representative bright-field images (right) of THP-1 cells treated with PMA at 0h and 48h, observed under a light microscope. Scale bars, 100μm. **(B-C)** RT-qPCR of M2 and M1 markers in macrophages after co-culture with PDAC cell lines AsPC-1 (B) and BxPC-3 (C). Control group: The co-culture system contained macrophages only. AsPC-1/BxPC-3 + GW4869: Exosome inhibitor GW4869 was added to the co-culture system. (n = 3). Two-way ANOVA analysis. **(D-E)** Representative IF images of PMA-pretreated THP-1 cells co-cultured with AsPC-1 (D) and BxPC-3 (E) cells. Scale bars, 20μm. **(F-G)** RT-qPCR analysis of cell cycle (F), M2 and M1 (G) markers in tumors. (n = 4). Two-way ANOVA analysis. Data presented as mean ± SD. n.s., no significant, **p* ≤ 0.05, ***p* ≤ 0.01, ****p* ≤ 0.001.

**
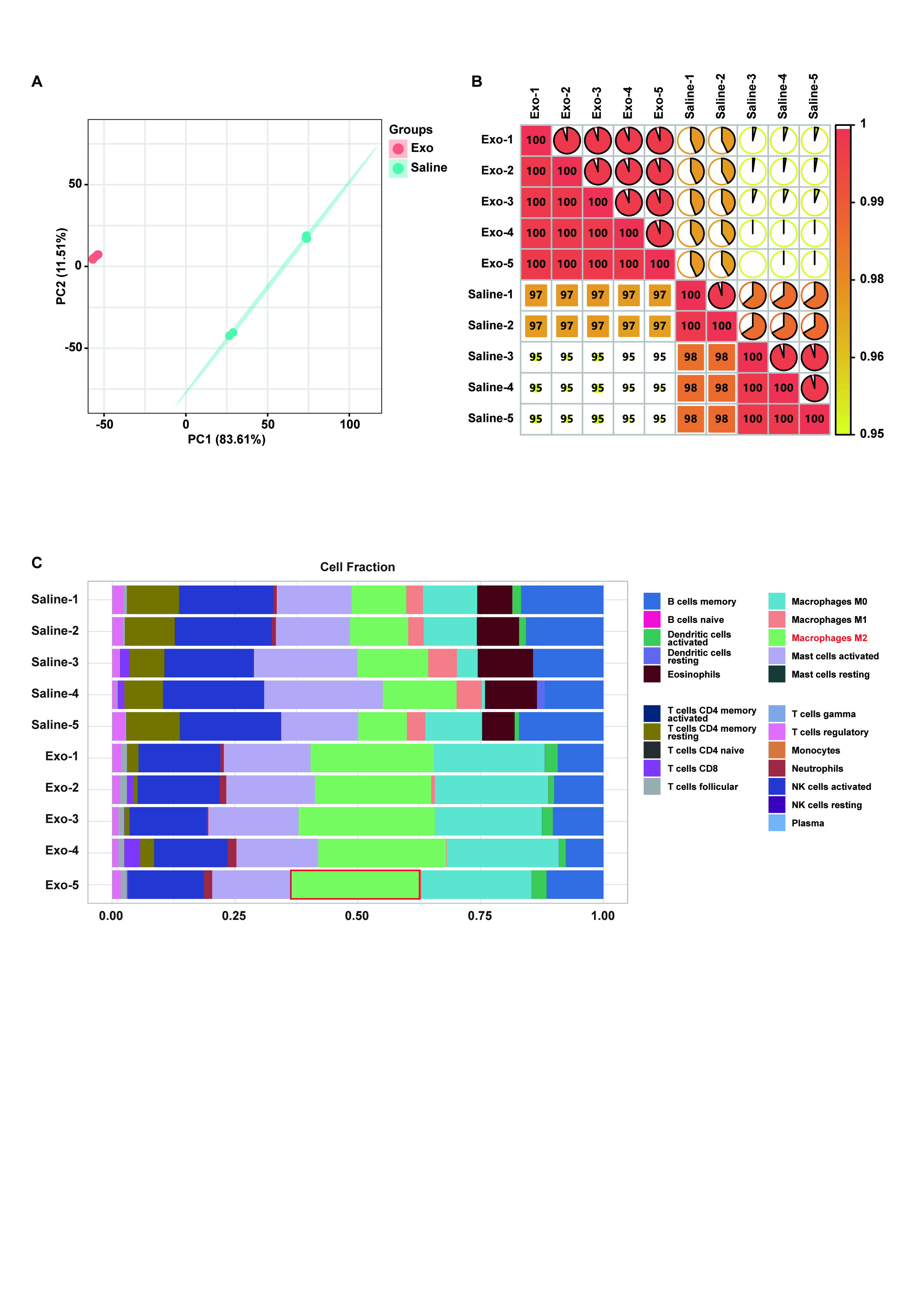
**

Fig. S2. Quality control of tumor tissue RNA-seq and analysis

**(A**) PCA plot showing group separation: Exo (red) and Saline (cyan). PC1 explained 83.61% and PC2 11.51% of variance. **(B)** Heatmap of sample correlations of transcriptome data in Exo and Saline groups. Colors indicated correlation strength, with pie charts showing similarity. **(C)** Estimated immune cell proportions in tumor samples calculated by CIBERSORT.


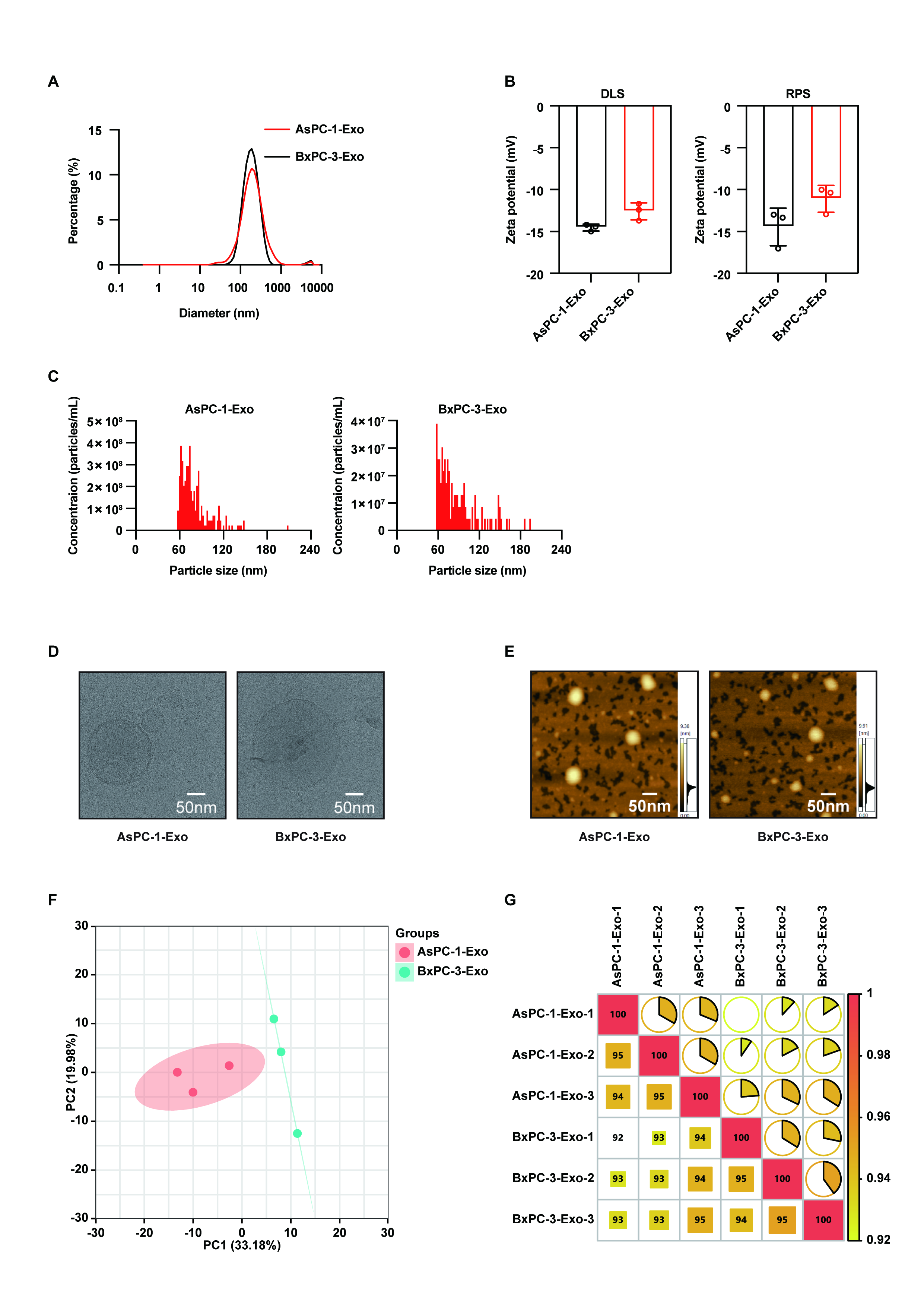


Fig. S3. Detailed characterization analyses of the purified exosomes and quality control of exosome proteomic

**(A)** Size distribution of AsPC-1 and BxPC-3 exosomal particles measured by DLS. Average particle sizes of 162.8nm for AsPC-1-Exo and 166.7nm for BxPC-3-Exo. **(B)** Average zeta potentials (ζ) of -14.53mV for AsPC-1-Exo and -12.6mV for BxPC-3-Exo via DLS (left). Average zeta potentials (ζ) of -14.46mV for AsPC-1-Exo and -11.11mV for BxPC-3-Exo via RPS (right). Data are presented as mean ± SD. n = 3. **(C)** Size distribution of AsPC-1 and BxPC-3 exosomal particles measured by RPS. Average particle sizes of 75nm for AsPC-1-Exo (left) and 79nm for BxPC-3-Exo (right) via RPS. **(D-E)** Representative Cryo-EM (D) and AFM (E) images of AsPC-1-Exo (left) and BxPC-3-Exo (right), showing membranous vesicles with predominantly spherical or elliptical shapes, ranging in size from 40 to 160nm. Scale bar, 50nm. **(F)** PCA plot showing group separation: AsPC-1-Exo (red) and BxPC-3-Exo (cyan). PC1 explains 19.98% and PC2 33.18% of variance. **(G)** Heatmap of sample correlations of transcriptome data in AsPC-1-Exo and BxPC-3-Exo groups. Colors indicated correlation strength, with pie charts showing similarity.


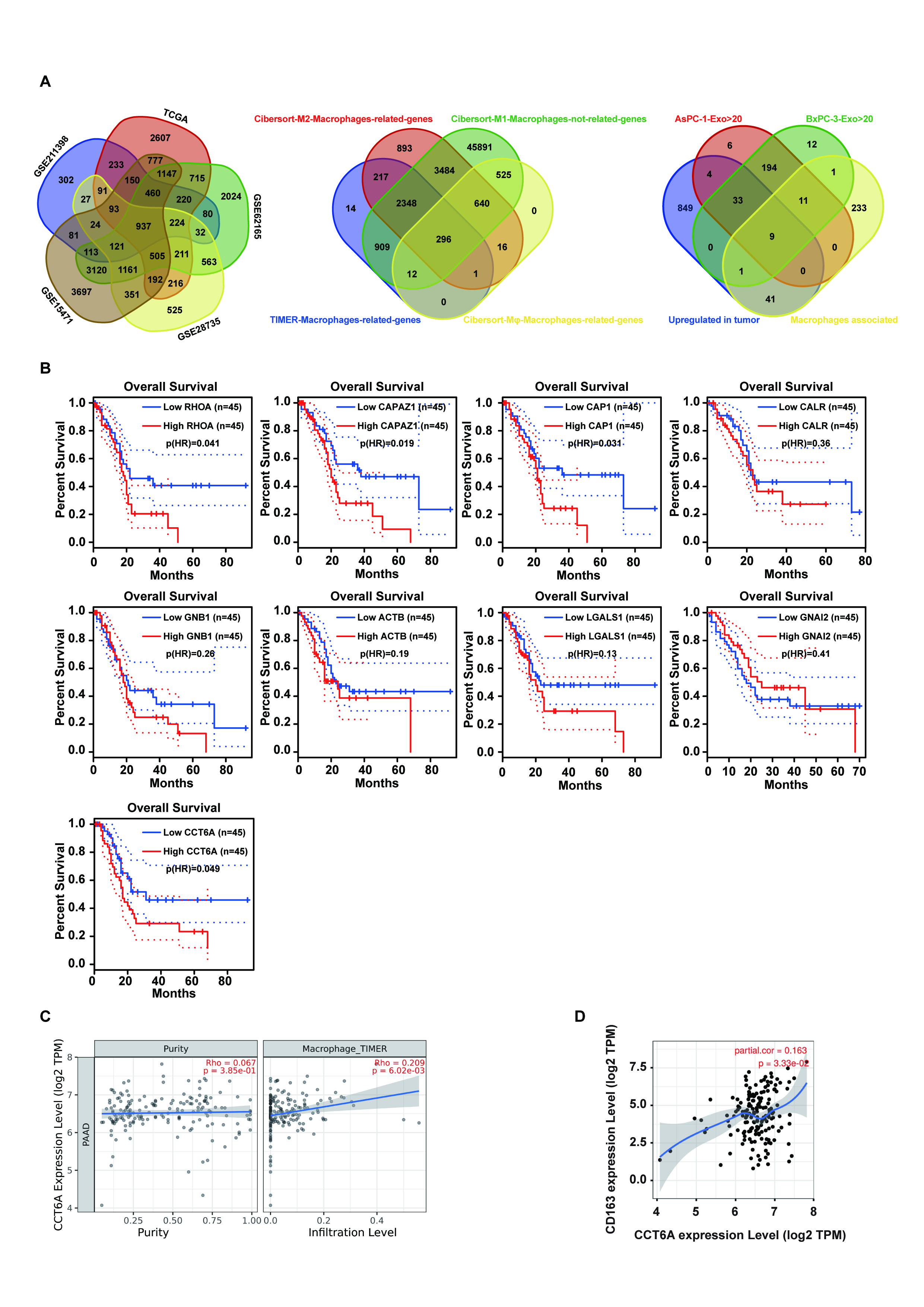


Fig. S4. Identification of CCT6A through bioinformatics analysis

**(A)** Venn diagrams illustrating the procedure for identifying molecules of interest. **(B)** Kaplan-Meier survival curves of the 9 selected genes in PDAC patients. log-rank test analysis. **(C)** The relationship between CCT6A and macrophages in TIMER database. **(D)** The relationship between CCT6A and M2 macrophage marker CD163 in TIMER database. Simple linear regression and the Pearson correlation coefficient analysis.


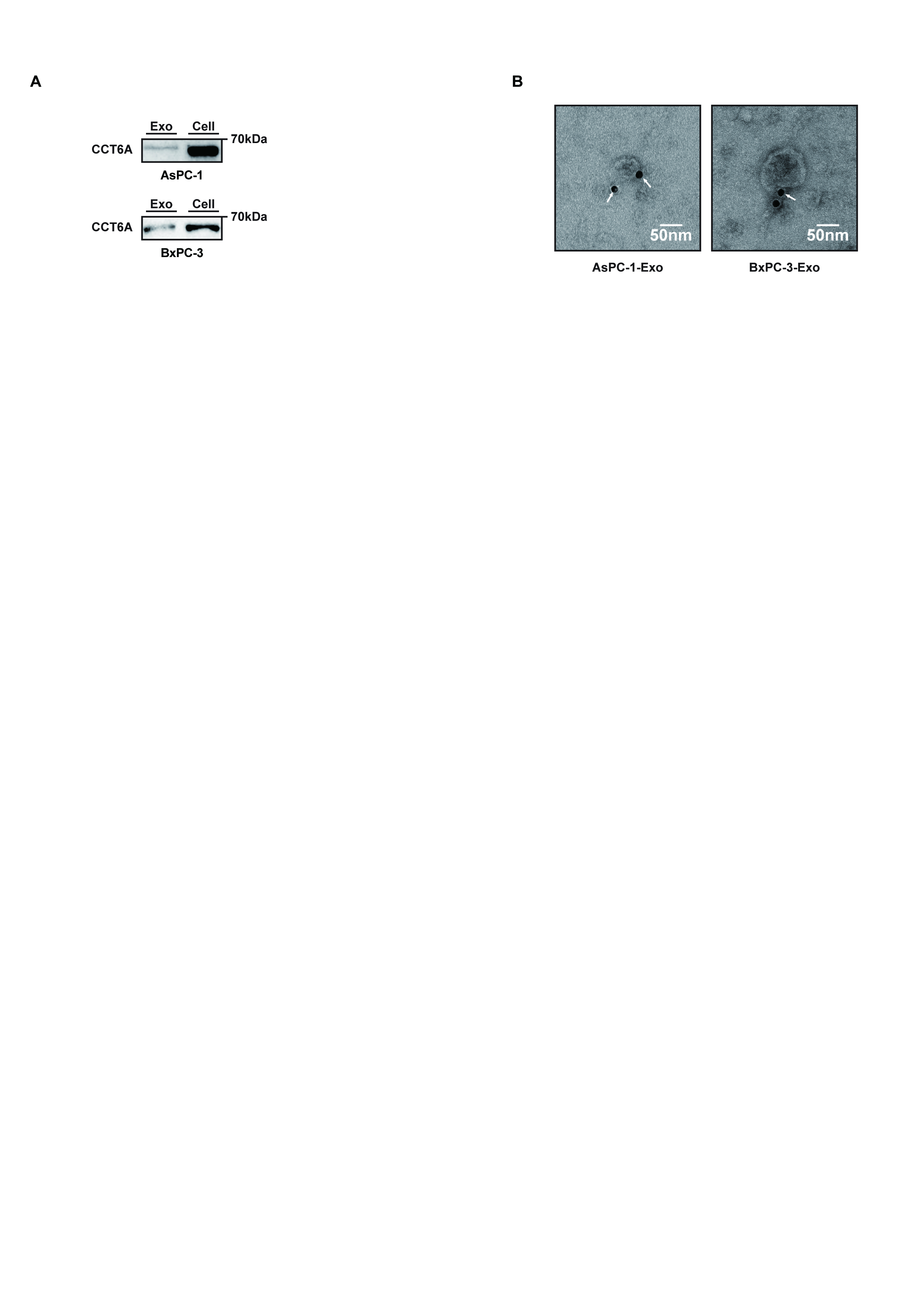


Fig. S5. PDAC cell-derived exosomes highly express CCT6A

**(A)** Western blotting analysis of CCT6A expression in exosomes isolated from AsPC-1 and BxPC-3 cells. **(B)** Detection of CCT6A expression in exosomes via immunoelectron microscopy. Scale bar, 50nm.


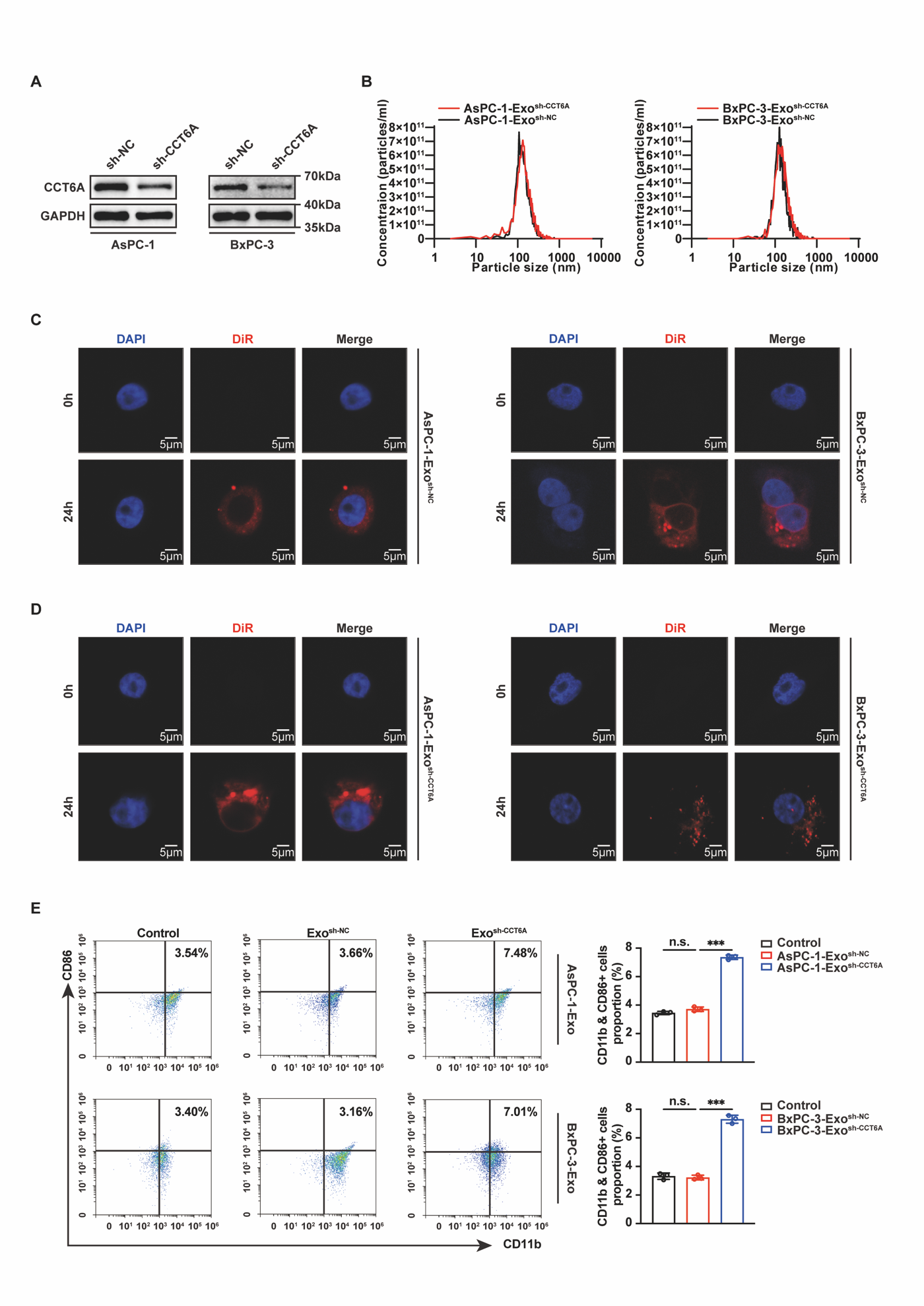


Fig. S6. PDAC-derived exosomal CCT6A influences macrophage M2 polarization without affecting exosome secretion or uptake

**(A)** Western blotting analysis for the knockdown efficacy of CCT6A in PDAC cell lines (AsPC-1 and BxPC-3). **(B)** Size distribution of exosomes derived from CCT6A-silenced and control PDAC cells measured by NTA. **(C-D)** Representative confocal micrographs of DiR-labeled Exo^sh-NC^ (C) and Exo^sh-CCT6A^ (D) co-incubated with macrophages for 0h and 24h. Scale bar, 5μm. **(E)** Flow cytometry analysis for distribution of M1-type (CD11b^+^CD86^+^) macrophages after exosomes treatments (left). Quantification (right) of M1-type macrophages. (n = 3). One-way ANOVA analysis. Data presented as mean ± SD. n.s., no significant, **p* ≤ 0.05, ***p* ≤ 0.01, ****p* ≤ 0.001.


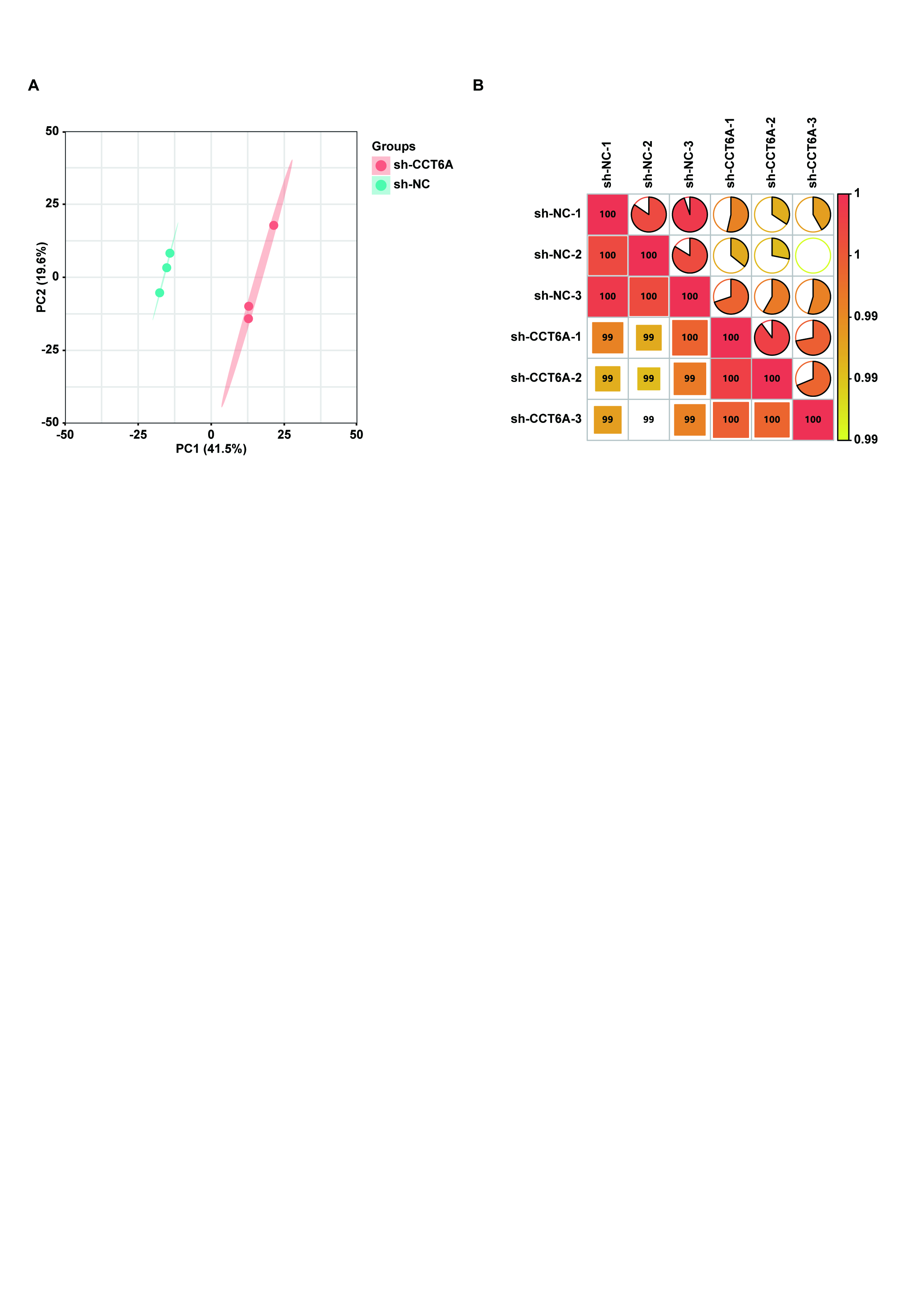


Fig. S7. Quality control of exosome-treated macrophages’ RNA-seq

**(A**) PCA plot showing group separation: sh-CCT6A (red) and sh-NC (cyan). PC1 explains 41.5% and PC2 explains 19.6% of variance. **(B)** Heatmap of sample correlations of transcriptome data in sh-CCT6A and sh-NC groups. Colors indicate correlation strength, with pie charts showing similarity.


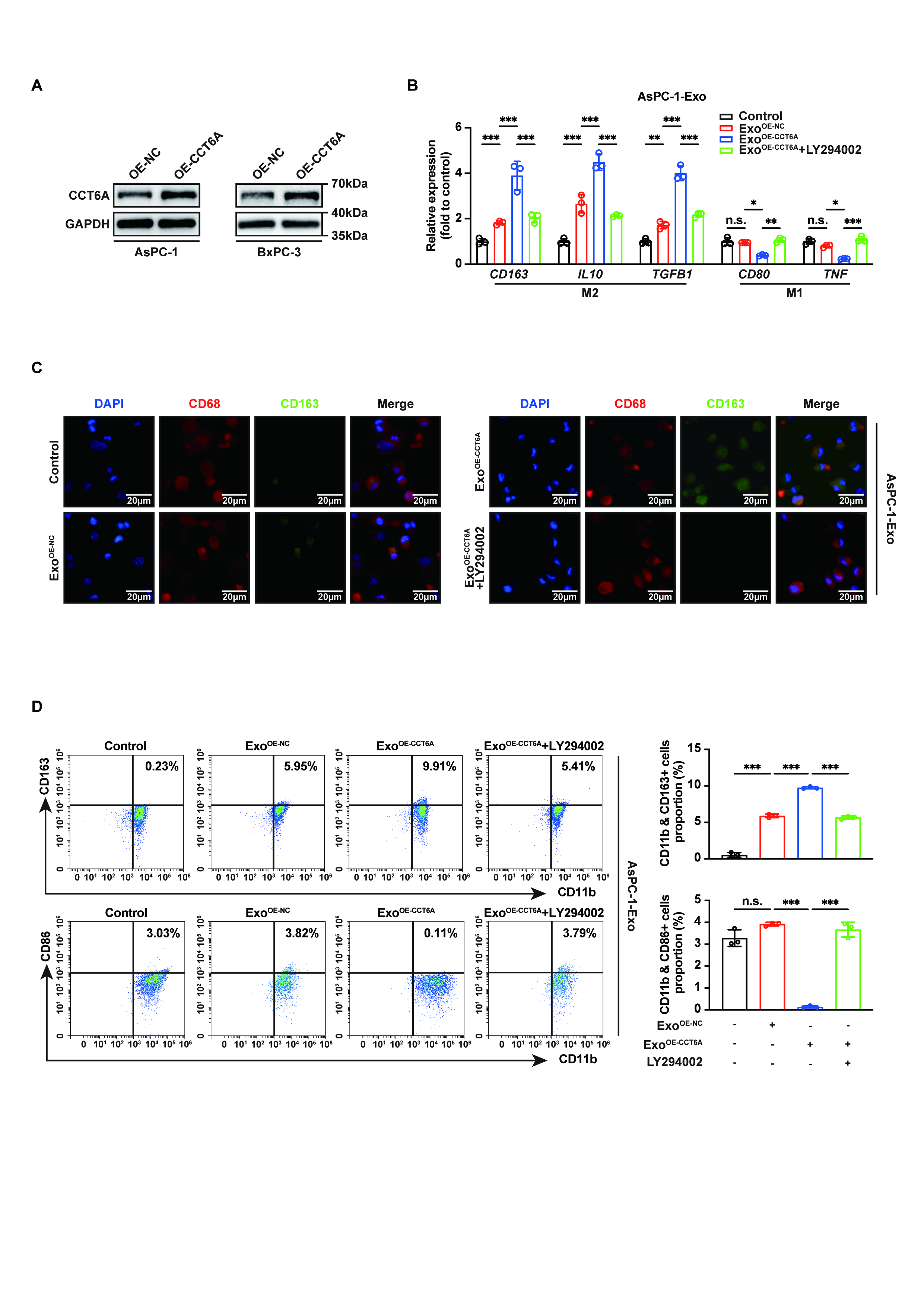


Fig. S8. Exosomal CCT6A induces M2 macrophage polarization via the PI3K-AKT pathway

**(A)** Western blotting analysis for the overexpression efficacy of CCT6A in PDAC cell lines (AsPC-1 and BxPC-3). **(B)** RT-qPCR of M2 and M1 markers in macrophages treated with different AsPC-1-Exo groups. (n = 3). Two-way ANOVA analysis. **(C)** Representative images of IF staining for CD68 (red) and CD163 (green) in macrophages treated with different AsPC-1-Exo groups. Scale bar, 20μm. **(D)** Flow cytometry analysis for distribution of M2-type (CD11b^+^CD163^+^) and M1-type (CD11b^+^CD86^+^) macrophages after different AsPC-1-Exo treatments (left). Quantification (right) of M2-type and M1-type macrophages. (n = 3). One-way ANOVA analysis. Data presented as mean ± SD. n.s., no significant, **p* ≤ 0.05, ***p* ≤ 0.01, ****p* ≤ 0.001.

**
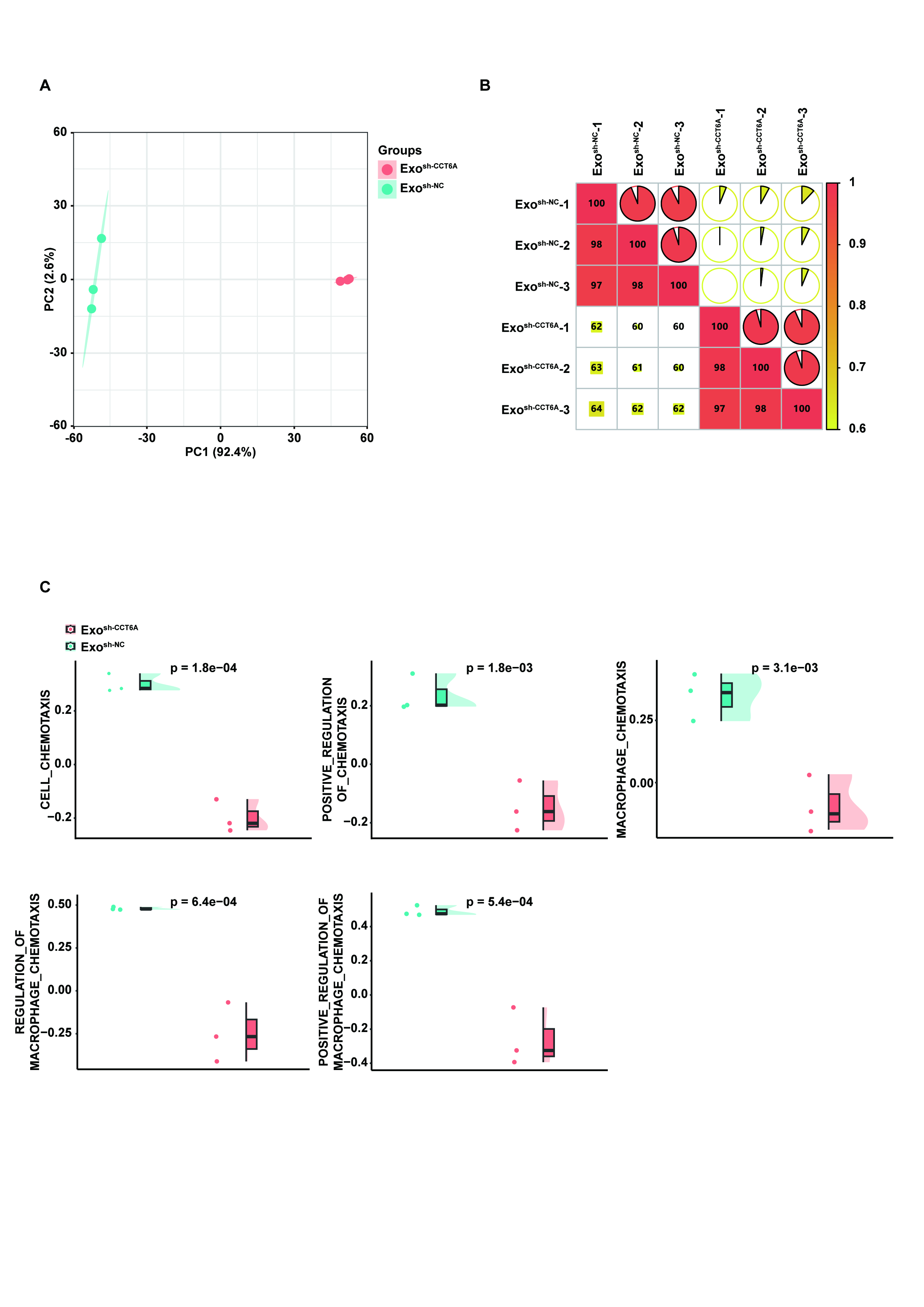
**

Fig. S9. Quality control and GSVA analysis of exosome proteomic

**(A)** PCA plot showing group separation: Exo^sh-CCT6A^ (red) and Exo^sh-NC^ (cyan). PC1 explains 92.4% and PC2 explains 2.6% of variance. **(B)** Heatmap of sample correlations of transcriptome data in Exo^sh-CCT6A^ and Exo^sh-NC^ groups. Colors indicate correlation strength, with pie charts showing similarity. **(C)** GSVA scores of Gene Ontology (GO) CHEMOTAXIS-related pathways in Exo^sh-NC^ and Exo^sh-CCT6A^ groups. Wilcoxon rank-sum test analysis. Data presented as mean ± SD.


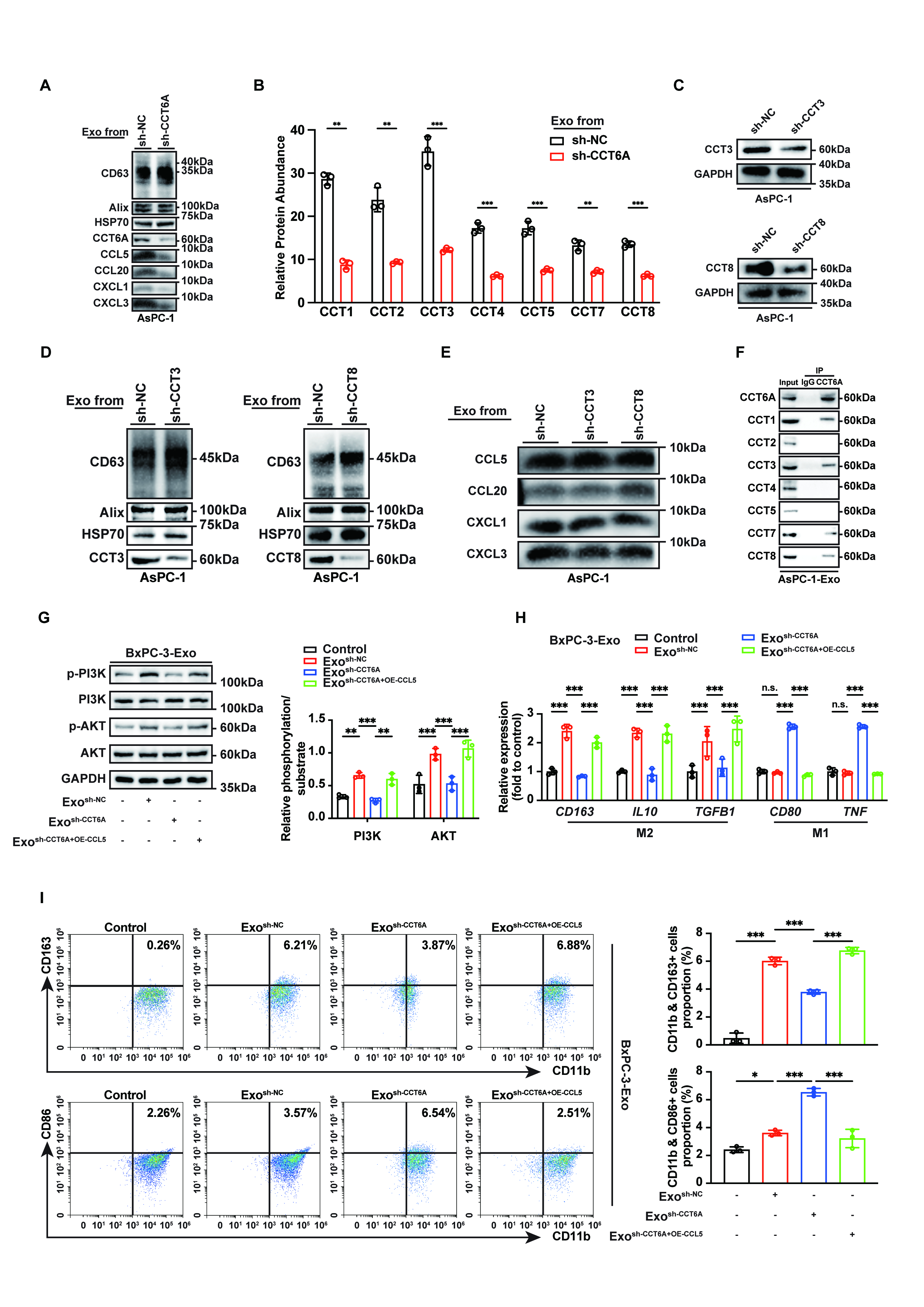


Fig. S10. CCL5 overexpression rescues the capacity of Exo^sh-CCT6A^ to promote PI3K/AKT signaling and M2 polarization in macrophages

**(A)** Analysis of the exosomal chemokines levels in Exo^sh-NC^ and Exo^sh-CCT6A^ groups via Western blotting. **(B)** Changes in other TRiC subunits assessed via proteomics. t-test analysis. **(C)** Western blotting analysis for the knockdown efficacy of CCT3 or CCT8 in AsPC-1 cells. **(D)** Analysis of the expression of specific exosomal biomarkers and CCT3 or CCT8 in exosomes isolated from AsPC-1 cells via Western blotting. **(E)** Western blotting analysis of CCL5, CCL20, CXCL1, and CXCL3 levels in exosomes with low expression of CCT3 or CCT8. **(F)** Detection of endogenous interaction between CCT6A and other TRiC subunits in AsPC-1-Exo via Co-IP and Western blotting. **(G)** Detection of PI3K-AKT signaling in Control, BxPC-3-Exo^sh-NC^, BxPC-3-Exo^sh-CCT6A^, or exosomes isolated from BxPC-3 cells with downregulated CCT6A and overexpressed CCL5 (BxPC-3-Exo^sh-CCT6A+OE-CCL5^) treated macrophages via Western blotting (left). Quantification (right) of p-PI3K/PI3K and p-AKT/AKT levels. (n = 3). Two-way ANOVA analysis. **(H)** RT-qPCR of M2 and M1 markers in macrophages treated with different BxPC-3-Exo groups. (n = 3). Two-way ANOVA analysis. **(I)** Flow cytometry analysis for distribution of M2-type (CD11b^+^CD163^+^) and M1-type (CD11b^+^CD86^+^) macrophages after different BxPC-3-Exo treatments (left). Quantification (right) of M2-type and M1-type macrophages. (n = 3). One-way ANOVA analysis. Data presented as mean ± SD. n.s., no significant, **p* ≤ 0.05, ***p* ≤ 0.01, ****p* ≤ 0.001.


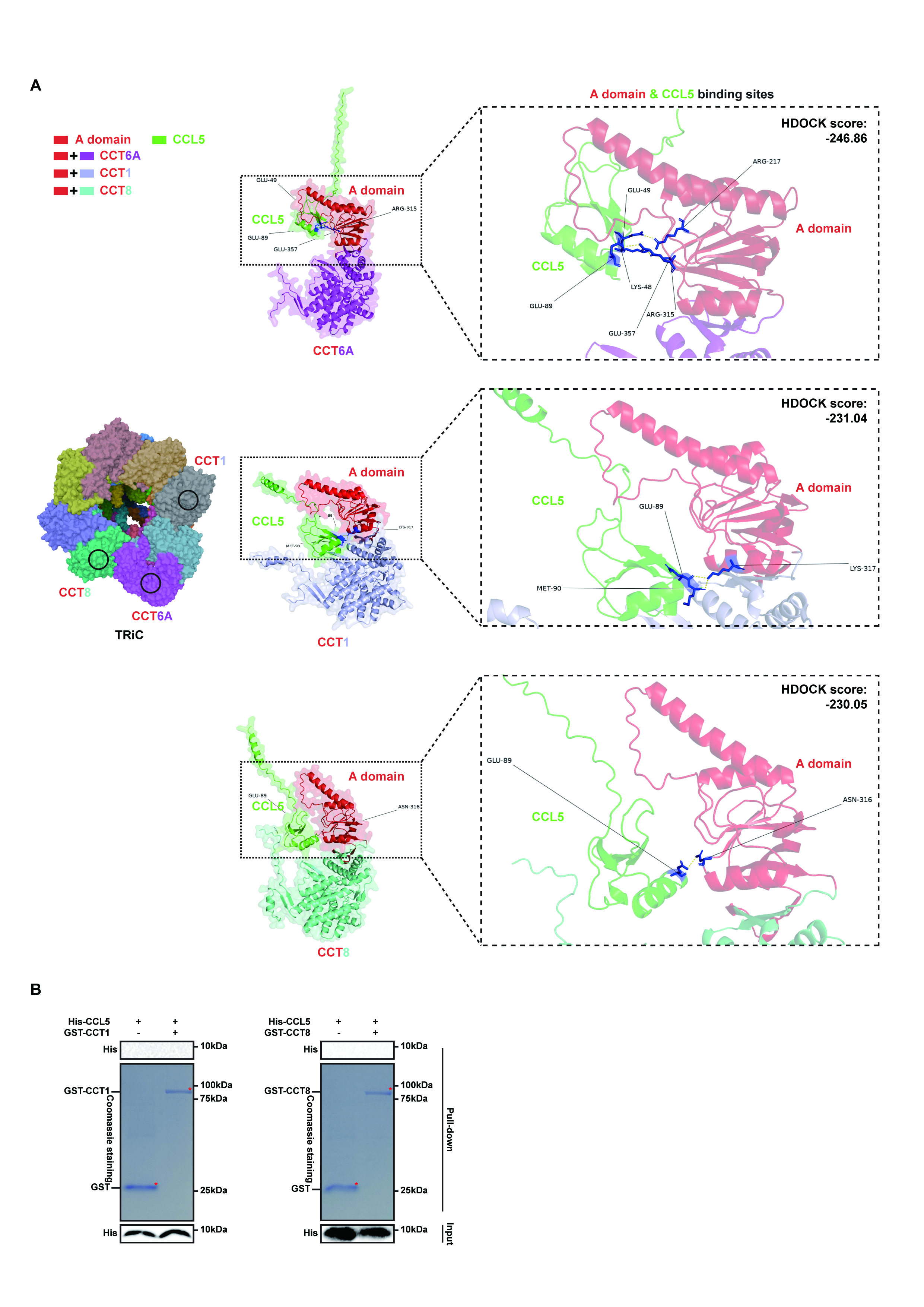


Fig. S11. CCL5 overexpression rescues the capacity of Exo^sh-CCT6A^ to promote PI3K/AKT signaling and M2 polarization in macrophages

**(A**) Molecular docking and HDOCK scores of CCL5 with CCT6A, CCT1, and CCT8. Created with BioRender.com. **(B)** GST pulldown assays performed by Western blotting or Coomassie Brilliant Blue (CBB) staining to detect direct binding of CCL5 with CCT1 or CCT8 in vitro.


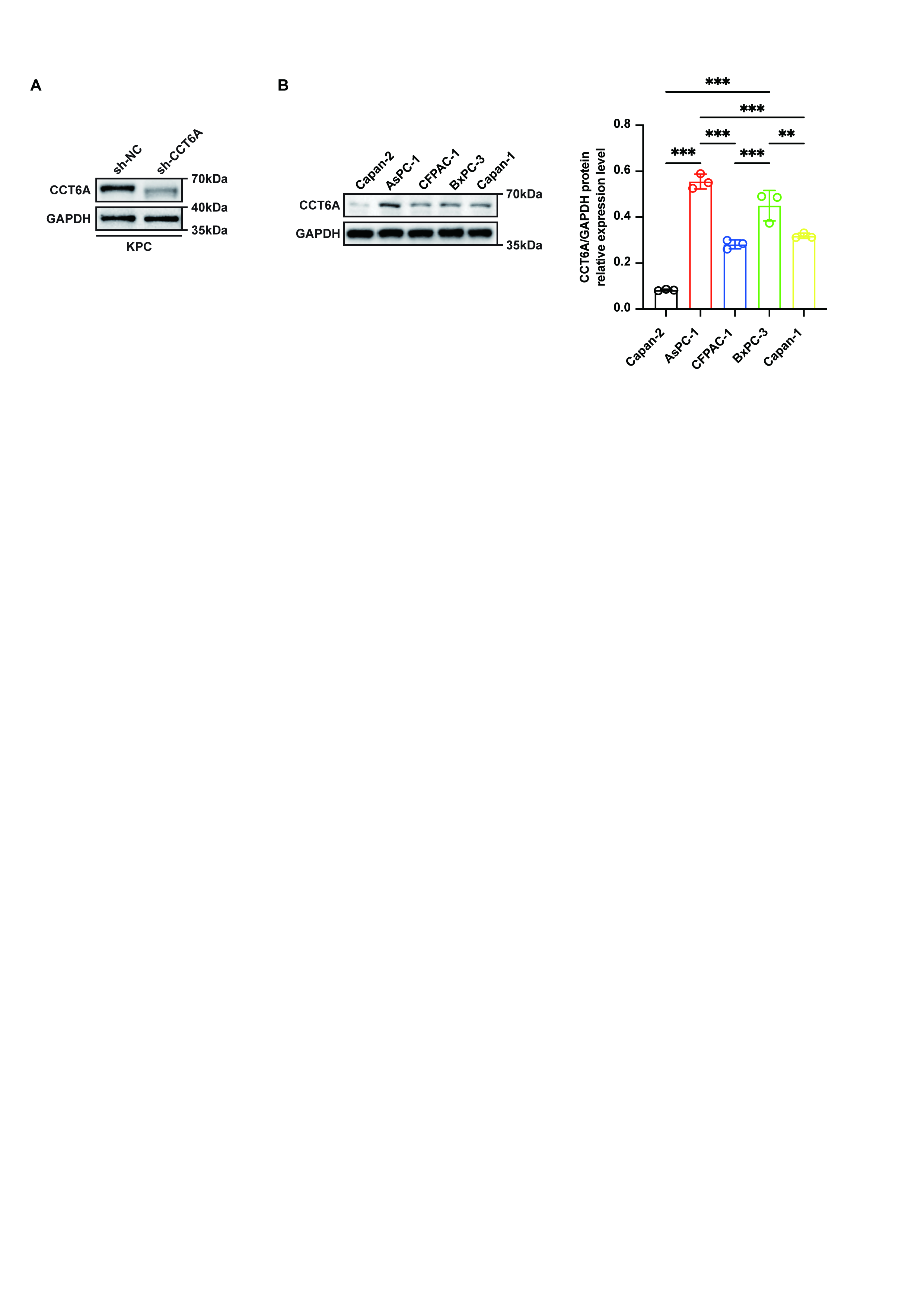


Fig. S12.

**(A)** Western blotting analysis for the knockdown efficacy of CCT6A in KPC cells. **(B)** Analysis of CCT6A levels in five PDAC cell lines through Western blotting (left). Quantification (right) of CCT6A/GAPDH level. (n = 3). One-way ANOVA analysis. Data presented as mean ± SD. n.s., no significant, **p* ≤ 0.05, ***p* ≤ 0.01, ****p* ≤ 0.001.

Table S1.

**Primer sequences used in this study.**

| Primer name | Sequence (5’-3’) |
| --- | --- |
| H-GAPDH-F | ACCACAGTCCATGCCATCAC |
| H-GAPDH-R | TCCACCACCCTGTTGCTGTA |
| H-CD163-F | TTTGTCAACTTGAGTCCCTTCAC |
| H-CD163-R | TCCCGCTACACTTGTTTTCAC |
| H-IL10-F | TCAAGGCGCATGTGAACTCC |
| H-IL10-R | GATGTCAAACTCACTCATGGCT |
| H-TGFB1-F | CAATTCCTGGCGATACCTCAG |
| H-TGFB1-R | GCACAACTCCGGTGACATCAA |
| H-CD80-F | GGCCCGAGTACAAGAACCG |
| H-CD80-R | TCGTATGTGCCCTCGTCAGAT |
| H-TNF-F | CTGCACTTTGGAGTGATCGG |
| H-TNF-R | TCAGCTTGAGGGTTTGCTAC |
| M-Actb-F | CGTTGACATCCGTAAAGACC |
| M-Actb-R | TAGGAGCCAGAGCAGTAATC |
| M-Mrc1-F | CTCTGTTCAGCTATTGGACGC |
| M-Mrc1-R | CGGAATTTCTGGGATTCAGCTTC |
| M-Il10-F | CAGGGATCTTAGCTAACGGAAA |
| M-Il10-R | GCTCAGTGAATAAATAGAATGGGAAC |
| M-Arg-1-F | CTCCAAGCCAAAGTCCTTAGAG |
| M-Arg-1-R | AGGAGCTGTCATTAGGGACATC |
| M-Nos2-F | GTTCTCAGCCCAACAATACAAGA |
| M-Nos2-R | GTGGACGGGTCGATGTCAC |
| M-Il6-F | CCAAGAGGTGAGTGCTTCCC |
| M-Il6-R | CTGTTGTTCAGACTCTCTCCCT |
| M-Ccnb1-F | GAAGAGCAGTCAGTTAGACC |
| M-Ccnb1-R | GTGTCCATTCACCGTTGTCA |
| M-Ccnb2-F | CCTCAGAACACCAAAGTACCAG |
| M-Ccnb2-R | CCTTCATGGAGACATCCTCAG |
| M-Ccnd1-F | TGCCGAGAAGTTGTGCATCT |
| M-Ccnd1-R | GCTTGTTCTCATCCGCCTCT |
| M-Ccne1-F | ACAGCAGGTCTTCGTGCAGATCG |
| M-Ccne1-R | CAGCGAGGACACCATAAGGAAATTC |
| shRNA for KD | Sequence (5’-3’) |
| H-shCCT6A-F | CCGGCGTGTCATTAGAGTATGAGAACTCGAGTTCTCATACTCTAATGACACGTTTTTG |
| H-shCCT6A-R | AATTCAAAAACGTGTCATTAGAGTATGAGAACTCGAGTTCTCATACTCTAATGACACG |
| H-shCCT3-F | CCGGGCGTGGAGTCATGATTAACAACTCGAGTTGTTAATCATGACTCCACGCTTTTTG |
| H-shCCT3-R | AATTCAAAAAGCGTGGAGTCATGATTAACAACTCGAGTTGTTAATCATGACTCCACGC |
| H-shCCT8-F | CCGGACATGCTGGAAGCTGGTATTCCTCGAGGAATACCAGCTTCCAGCATGTTTTTTG |
| H-shCCT8-R | AATTCAAAAAACATGCTGGAAGCTGGTATTCCTCGAGGAATACCAGCTTCCAGCATGT |
| H-shNC-F | CCGGTTCTCCGAACGTGTCACGTCTCGAGACGTGACACGTTCGGAGAATTTTTG |
| H-shNC-R | AATTCAAAAATTCTCCGAACGTGTCACGTCTCGAGACGTGACACGTTCGGAGAA |
| M-shCct6a-F | CCGGGCGGCGGTAAAGACCCTAAATCTCGAGATTTAGGGTCTTTACCGCCGCTTTTTG |
| M-shCct6a-R | AATTCAAAAAGCGGCGGTAAAGACCCTAAATCTCGAGATTTAGGGTCTTTACCGCCGC |
| M-shNC-F | CCGGTTCTCCGAACGTGTCACGTTTCGAGACGTGACACGTTCGGAGAATTTTTG |
| M-shNC-R | AATTCAAAAATTCTCCGAACGTGTCACGTCTCGAAACGTGACACGTTCGGAGAA |
