# Supplementary material for: Tumor-derived exosomal CCT6A serves as a matchmaker introducing chemokines to tumor-associated macrophages in pancreatic ductal adenocarcinoma
Source: Cell Death Dis. 2025 May 15;16(1):382. doi: 10.1038/s41419-025-07720-y (PMC12081750; doi:10.1038/s41419-025-07720-y)
Supplement: Supplementary file 2 — Original western blots [file 41419_2025_7720_MOESM2_ESM.pdf]

**Fig. 1C** Repeat#1

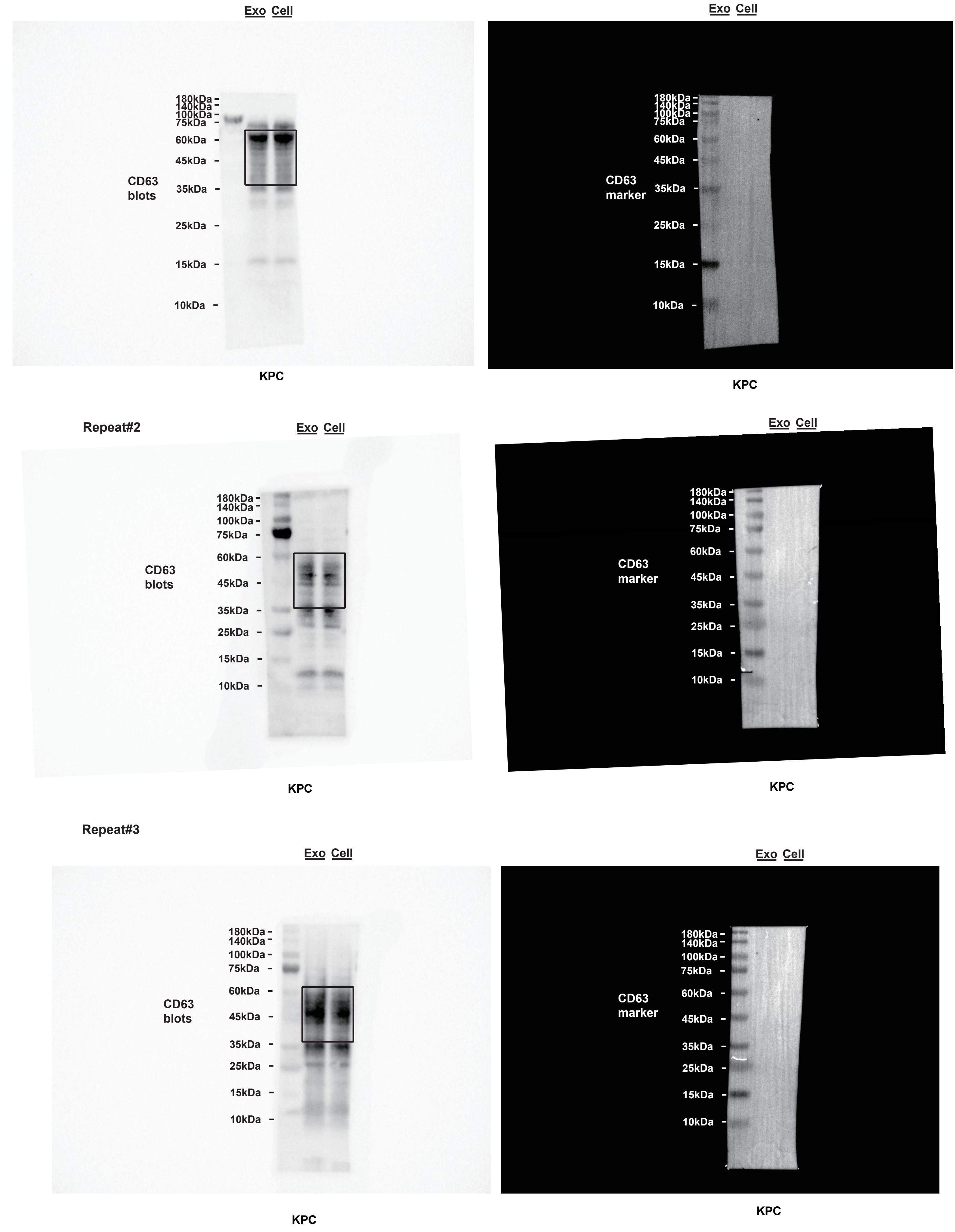


The exosomes were randomly selected from different extraction batches with varying storage times at -80°C. CD63 band shifts may result from its high glycosylation and cell-type-specific modification patterns.

Repeat#1

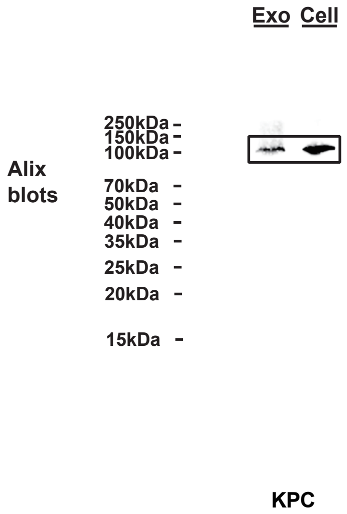


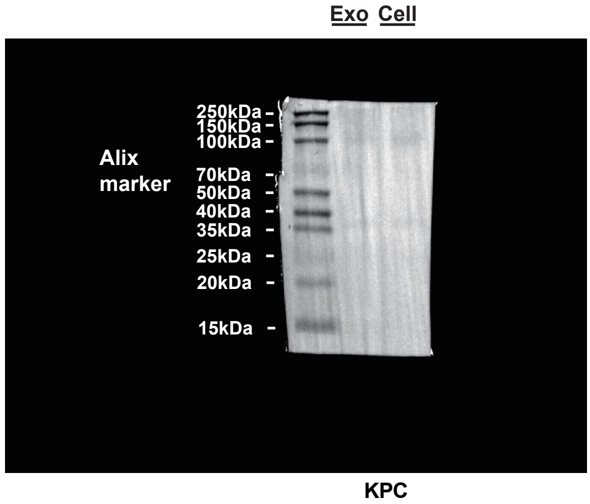


Repeat#2

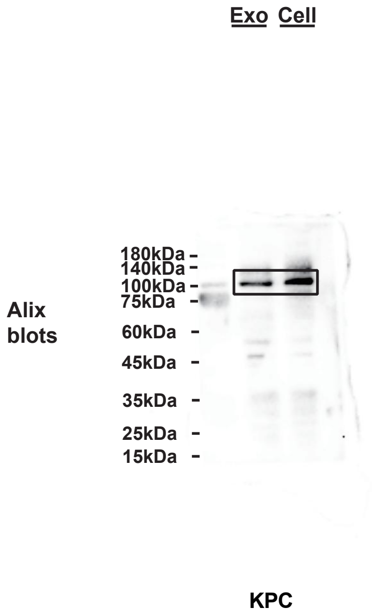


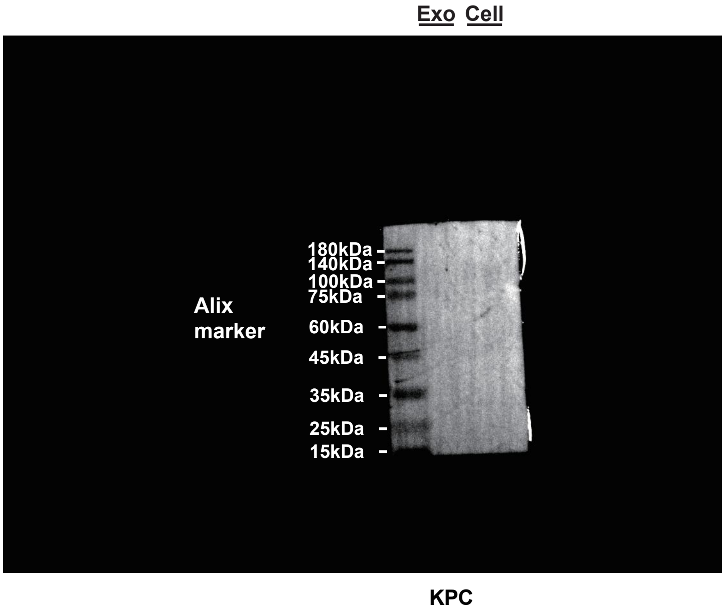


Repeat#3

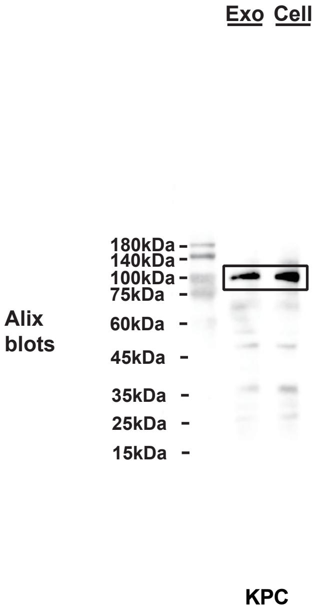


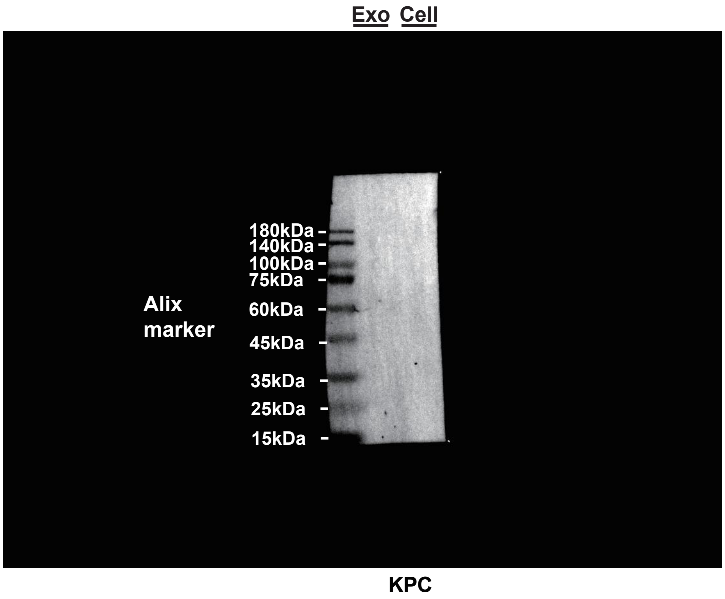

Repeat#1

Exo Cell

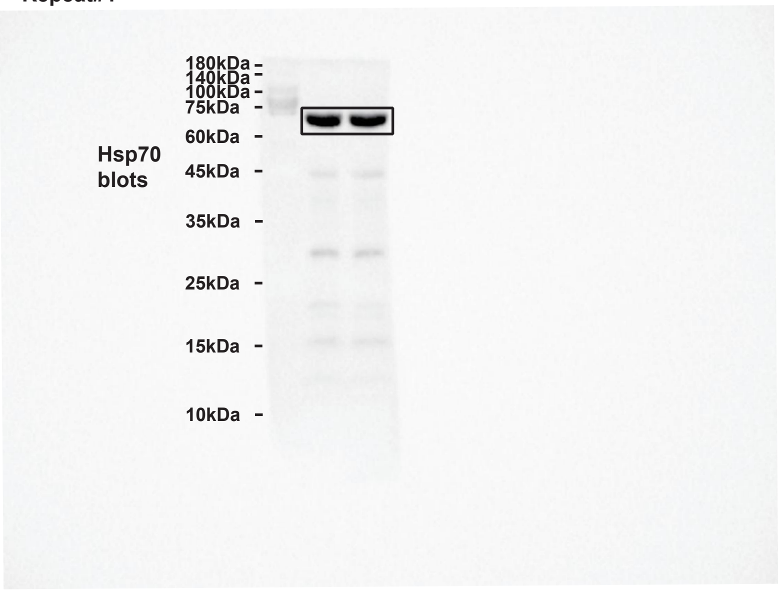


KPC

Exo Cell

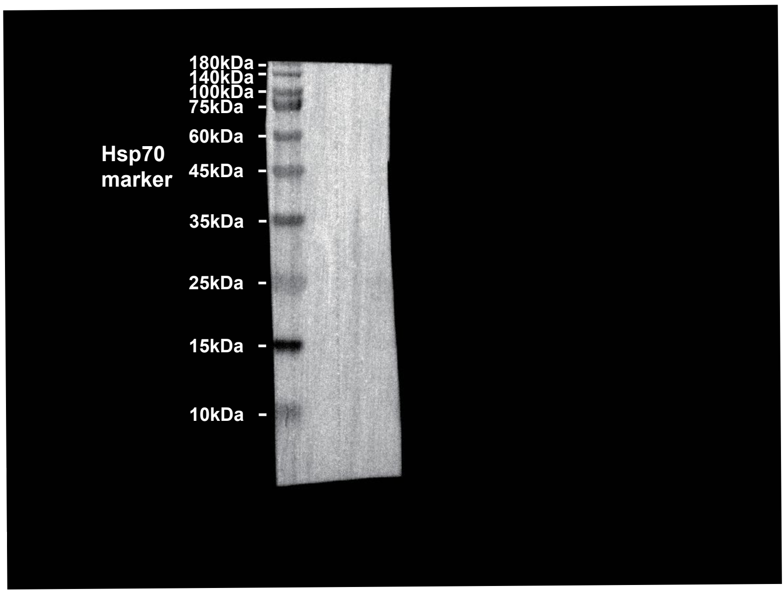


KPC

Repeat#2

Exo Cell

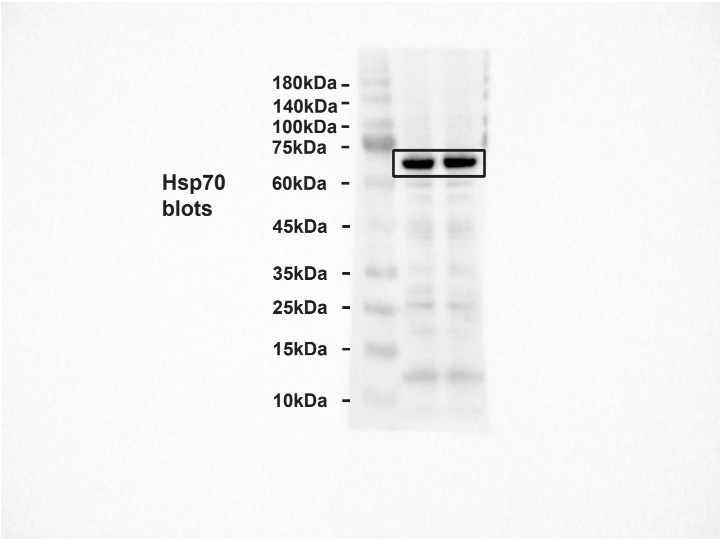


KPC

Exo Cell

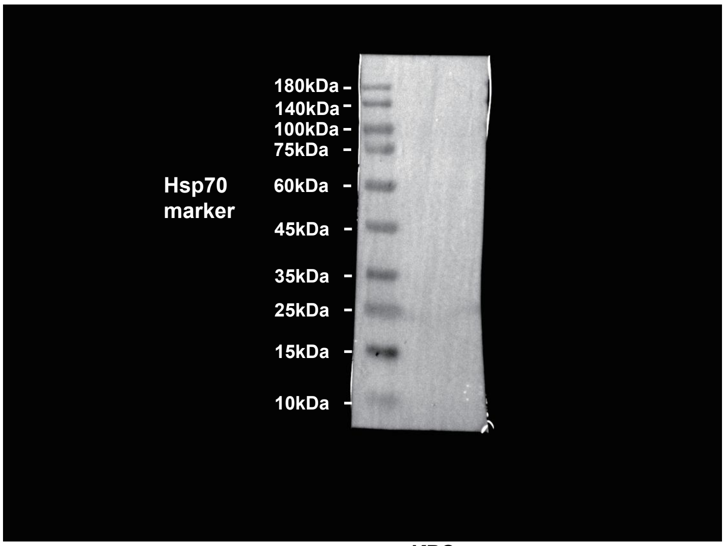


KPC

Repeat#3

Exo Cell

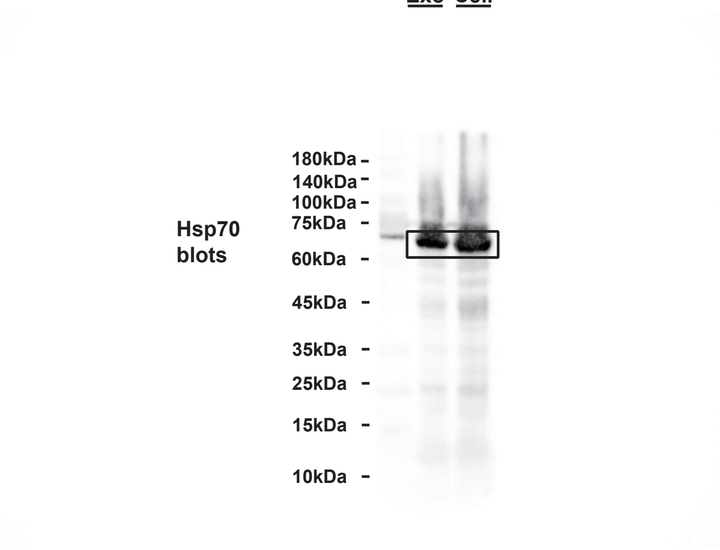


KPC

Exo Cell

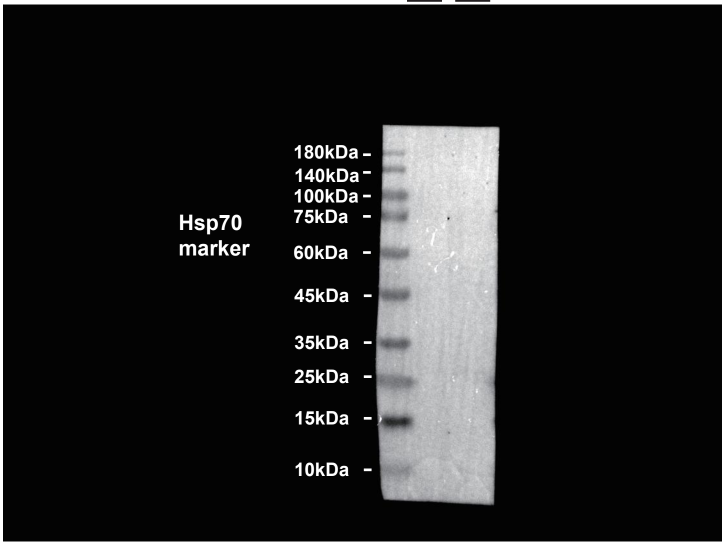


KPC

Repeat#1

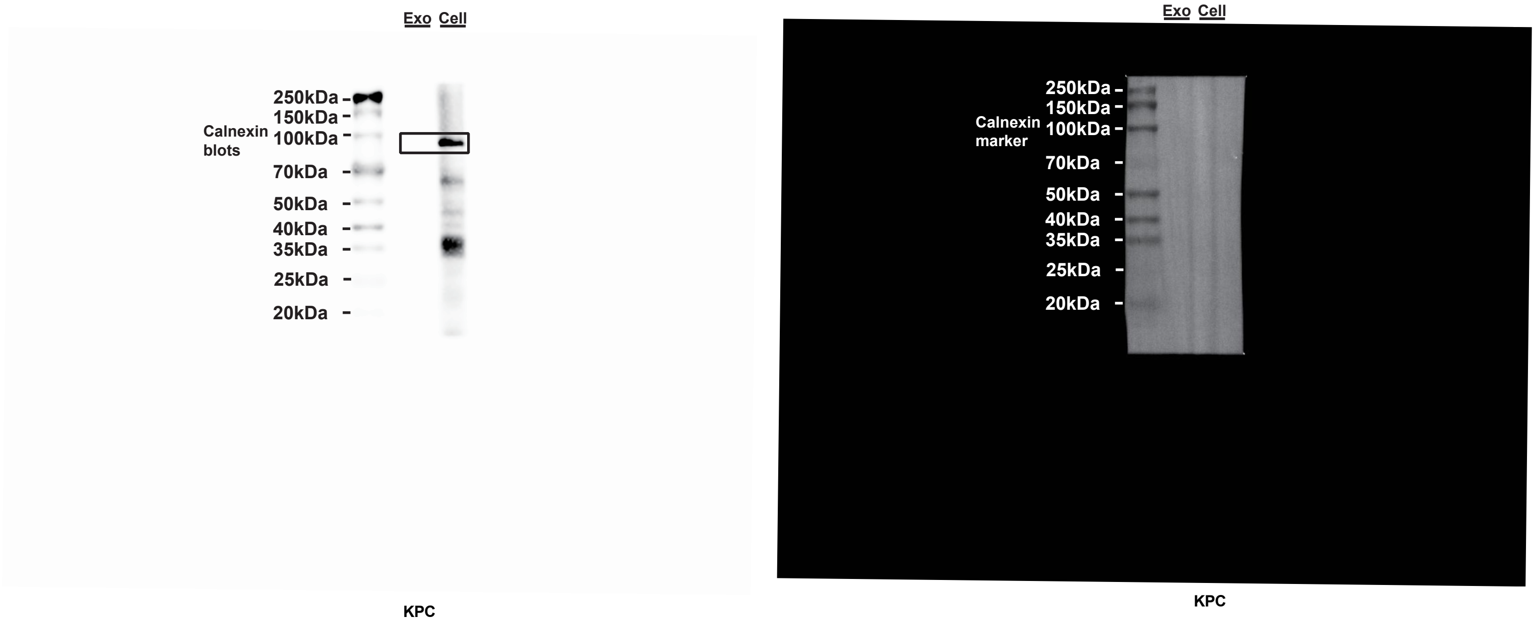


Repeat#2

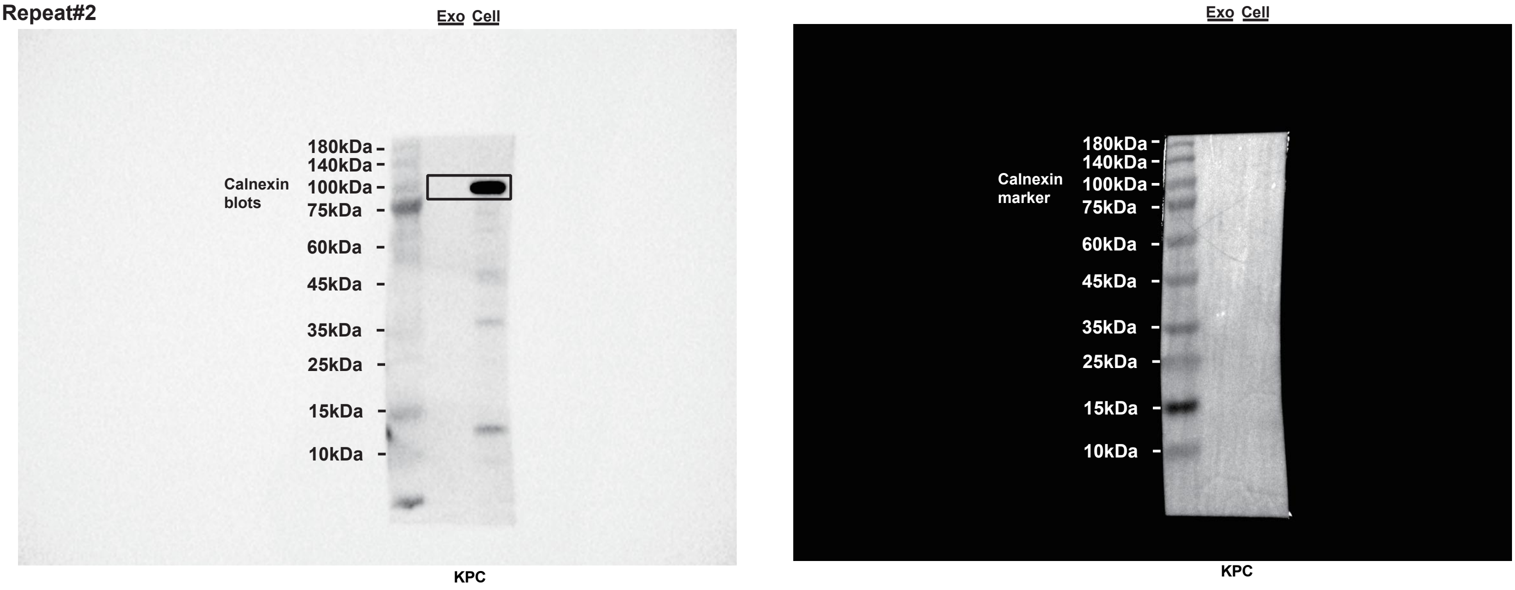


Repeat#3

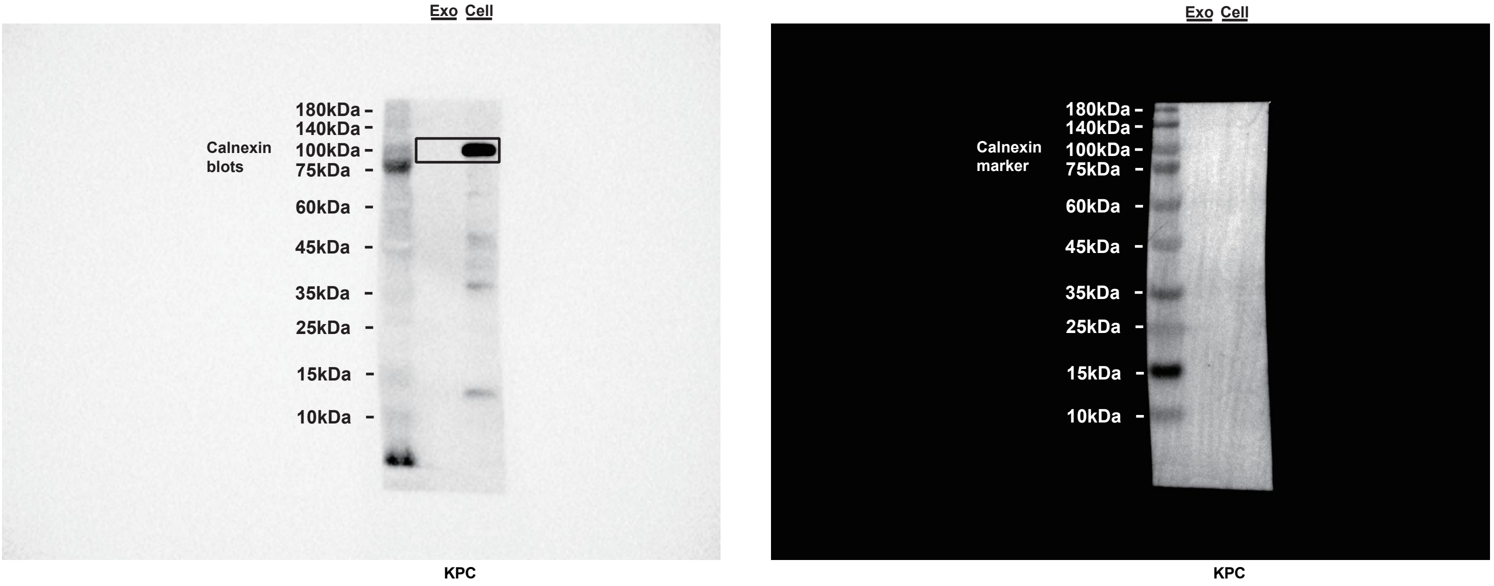

**Repeat#1**

Exo Cell

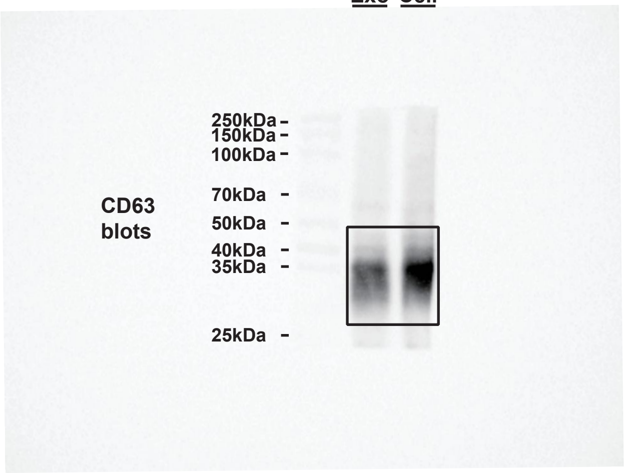


AsPC-1

Exo Cell

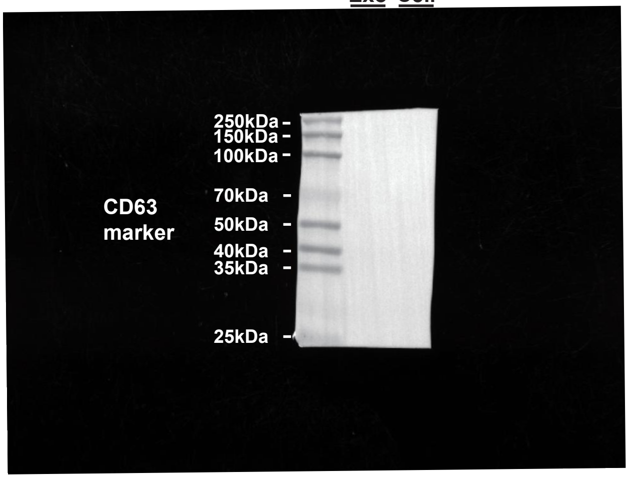


AsPC-1

**Repeat#2**

Exo Cell

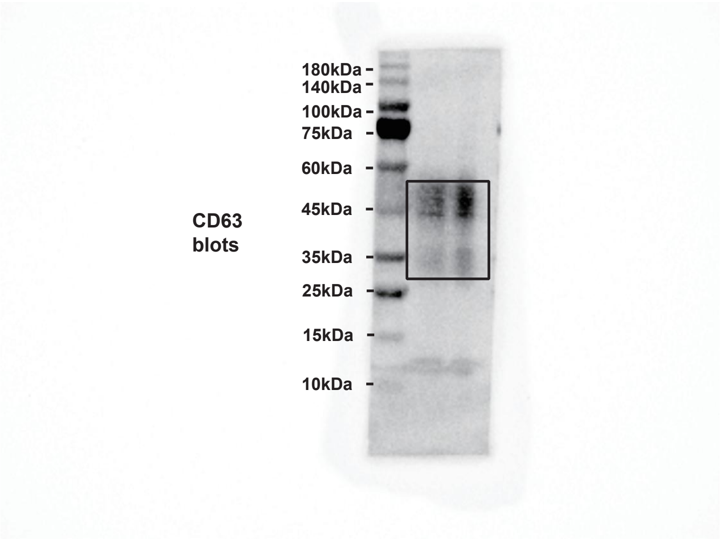


AsPC-1

Exo Cell

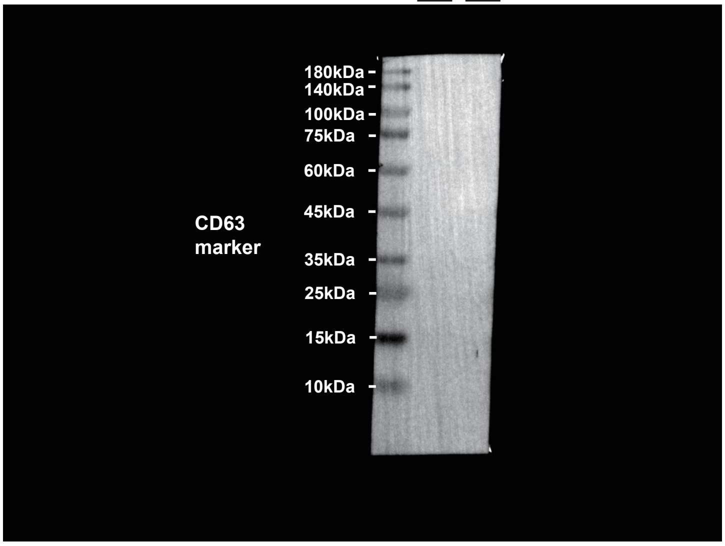


AsPC-1

**Repeat#3**

Exo Cell

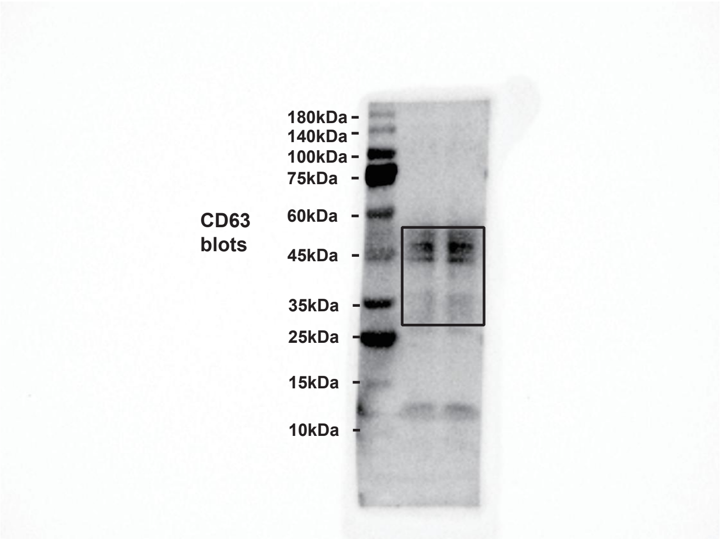


AsPC-1

Exo Cell

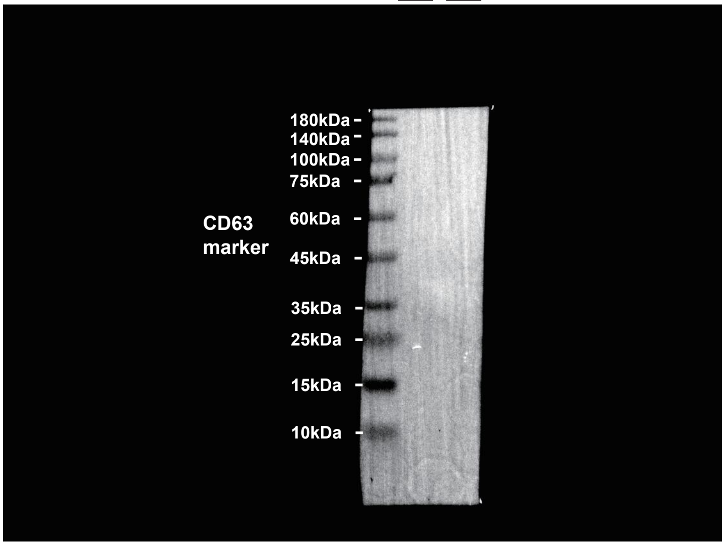


AsPC-1

**Repeat#1**

Exo Cell

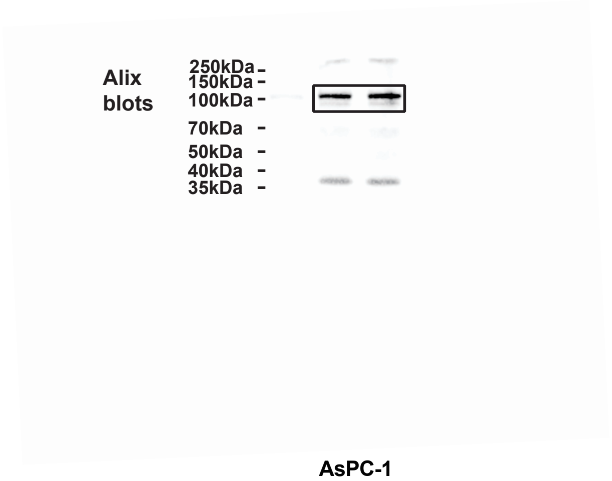


AsPC-1

Exo Cell

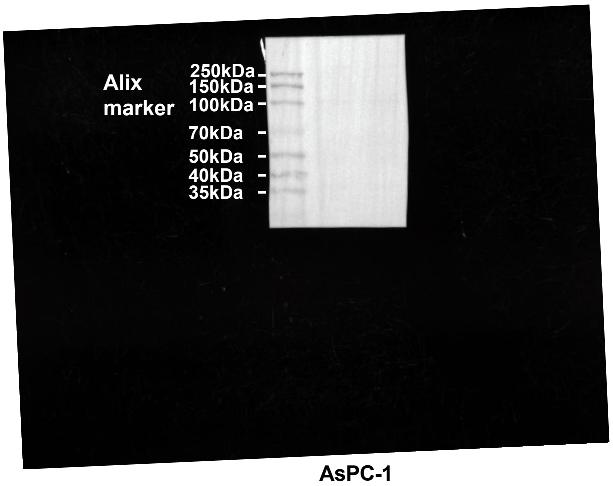


AsPC-1

**Repeat#2**

Exo Cell

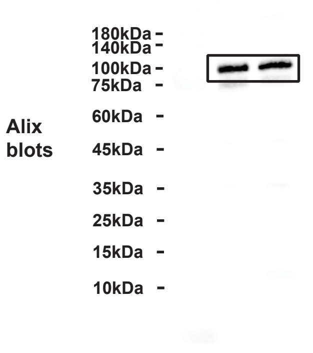


AsPC-1

Exo Cell

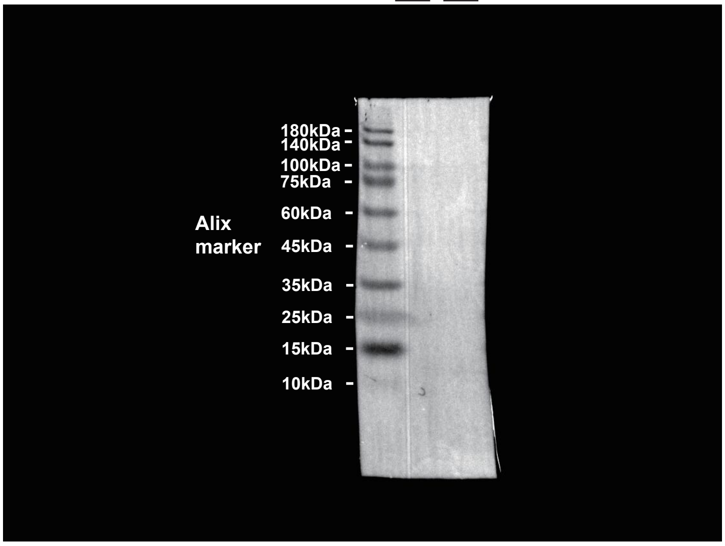


AsPC-1

**Repeat#2**

Exo Cell

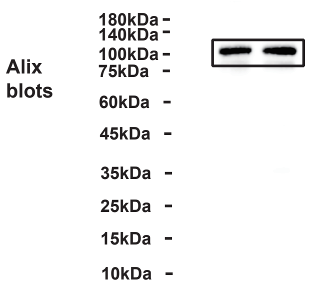


AsPC-1

Exo Cell

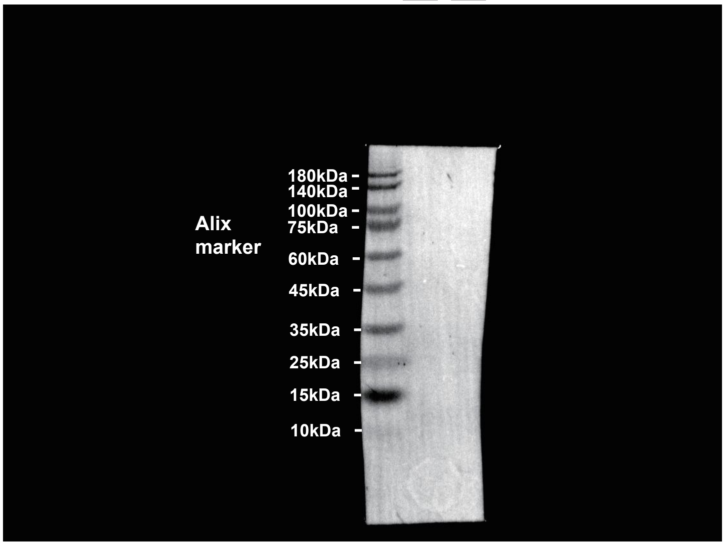


AsPC-1

Repeat#1

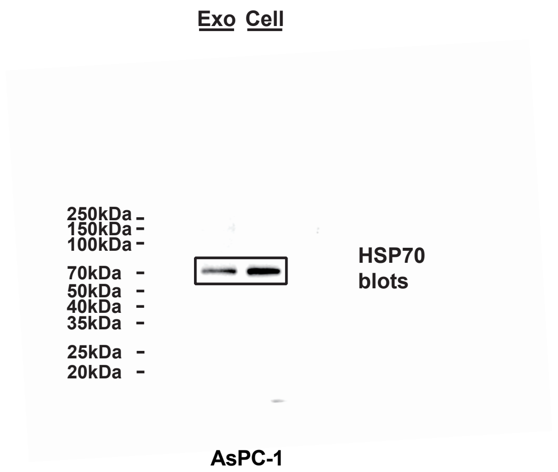


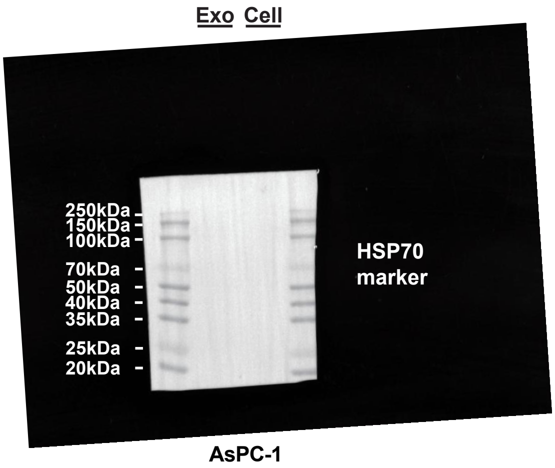


Repeat#2

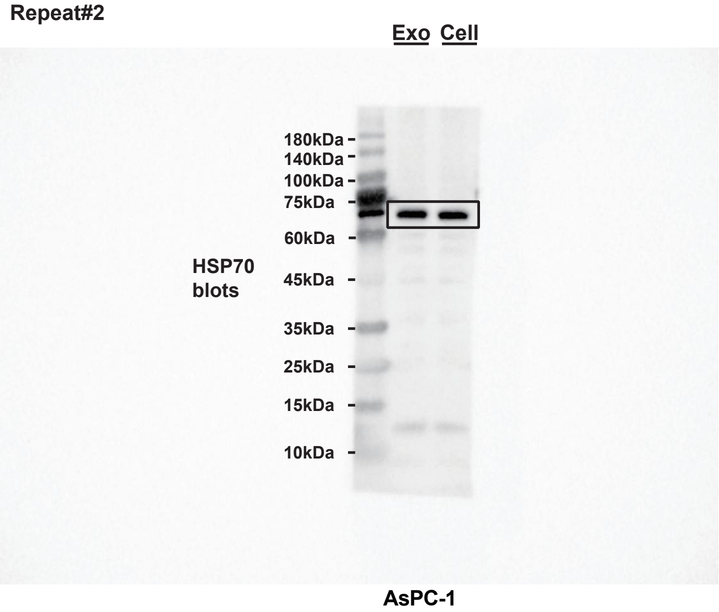


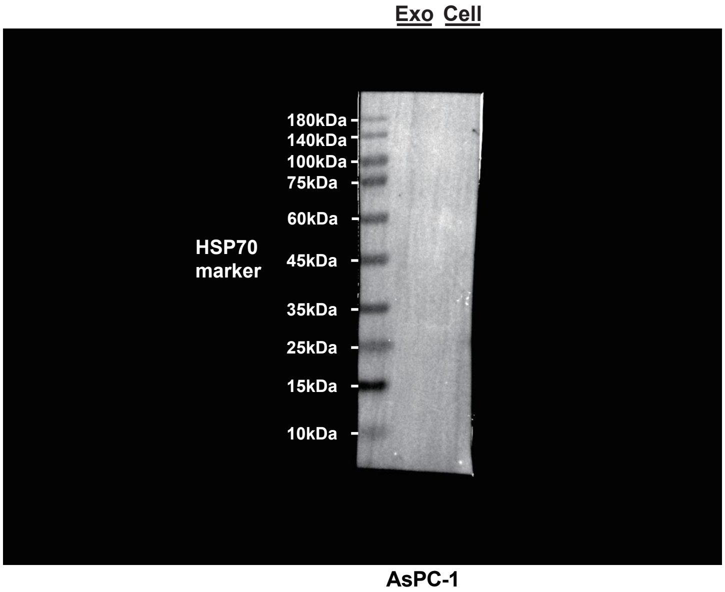


Repeat#3

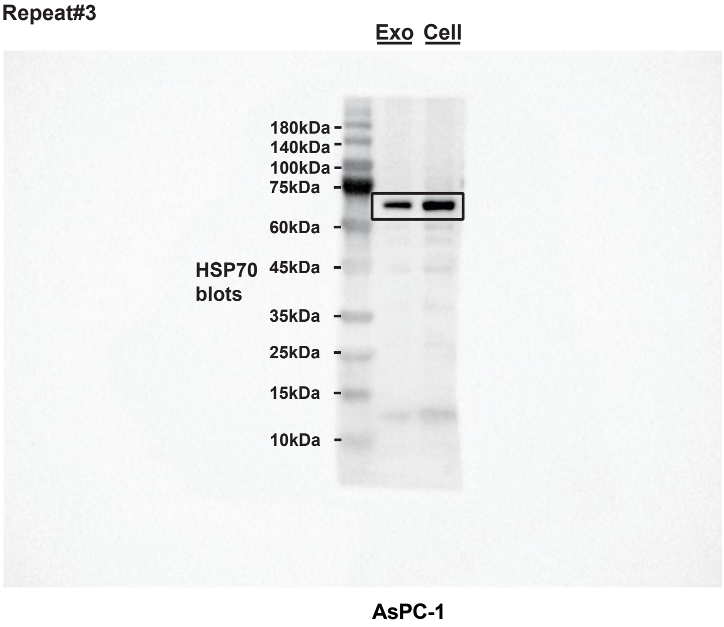


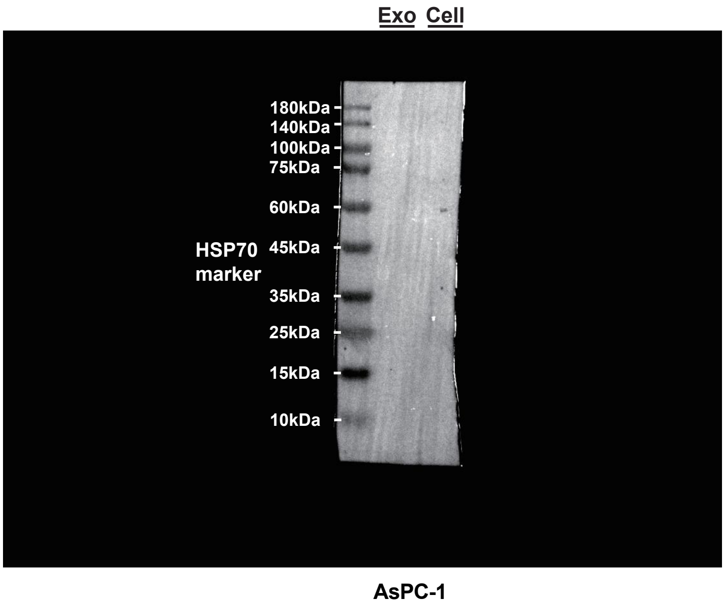

Repeat#1

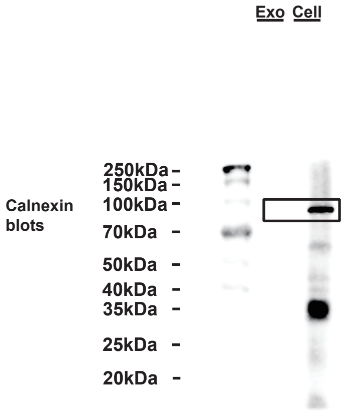


AsPC-1

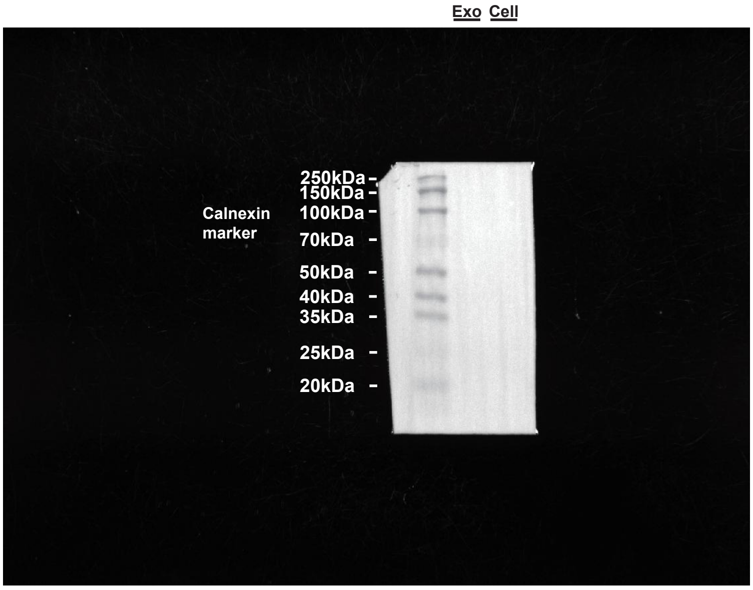


AsPC-1

Repeat#2

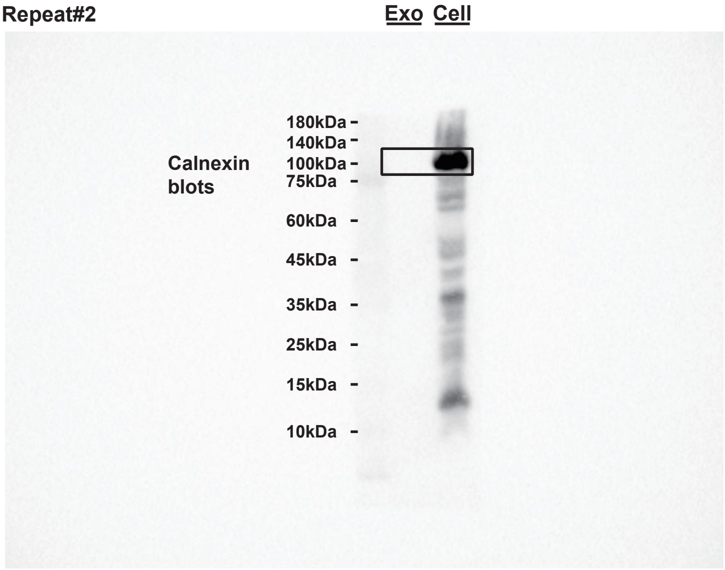


AsPC-1

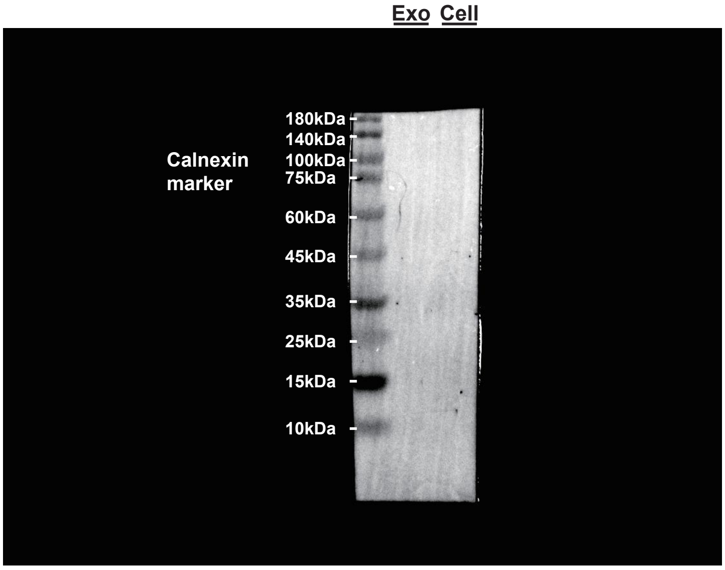


AsPC-1

Repeat#3

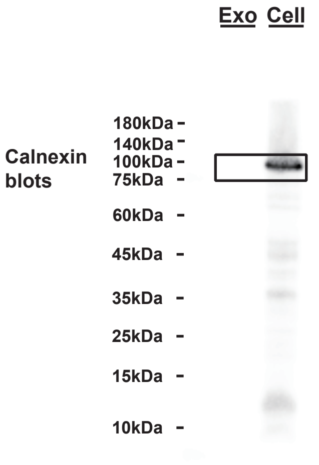


AsPC-1

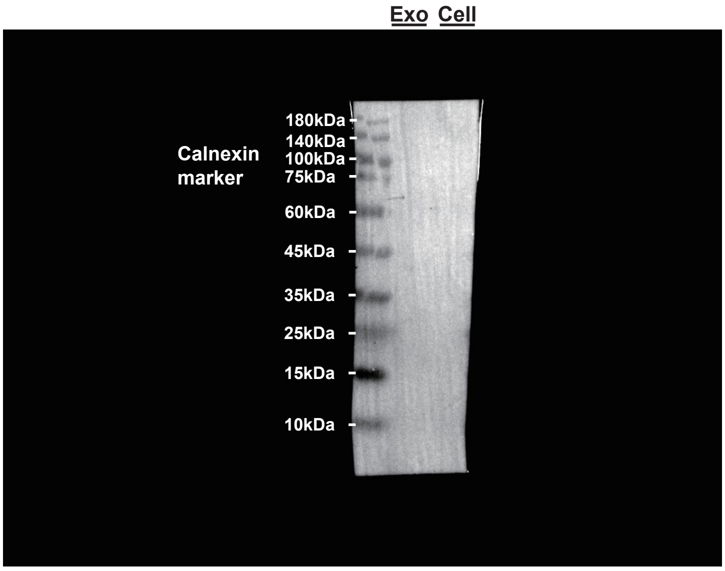


AsPC-1

Repeat#1

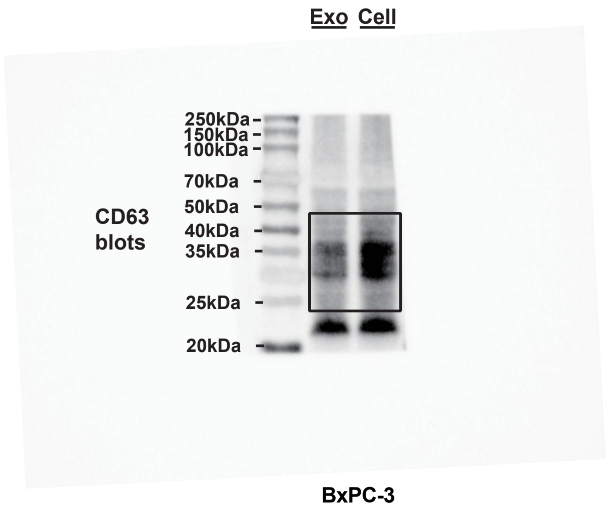


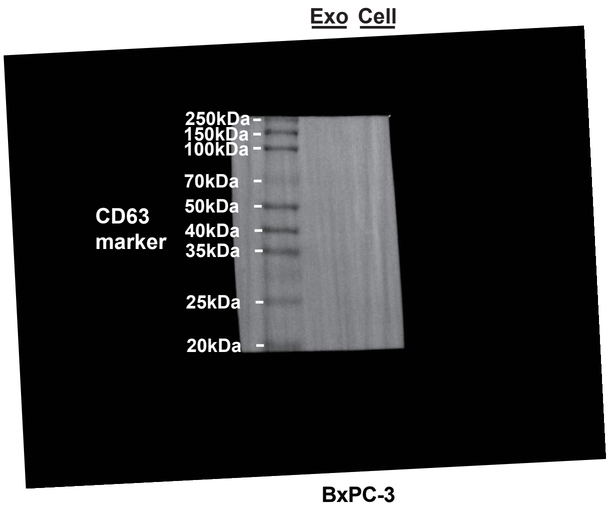


Repeat#2

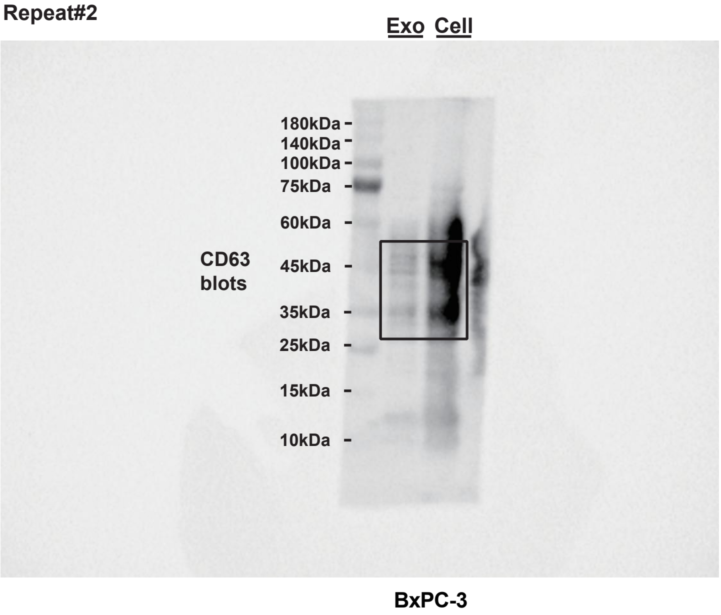


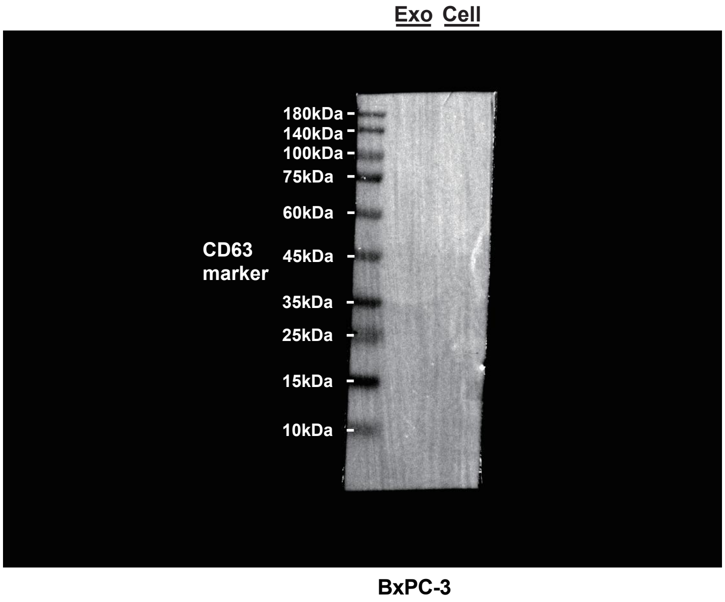


Repeat#3

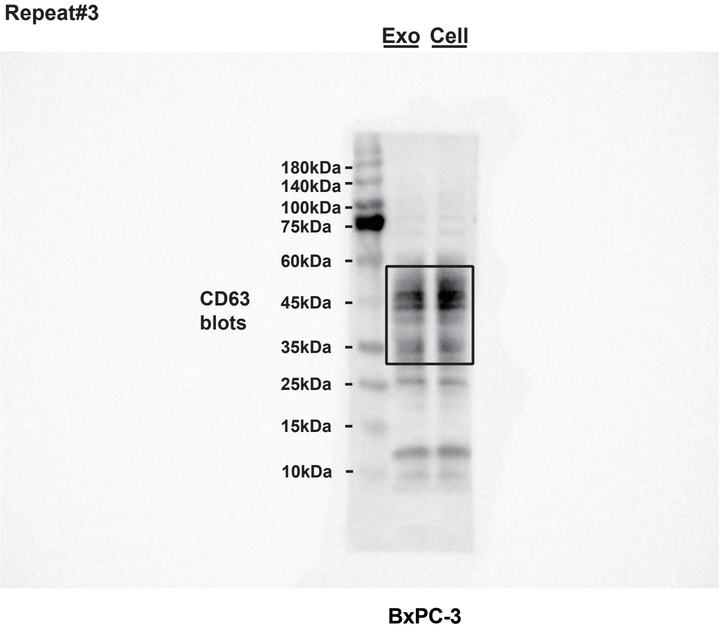


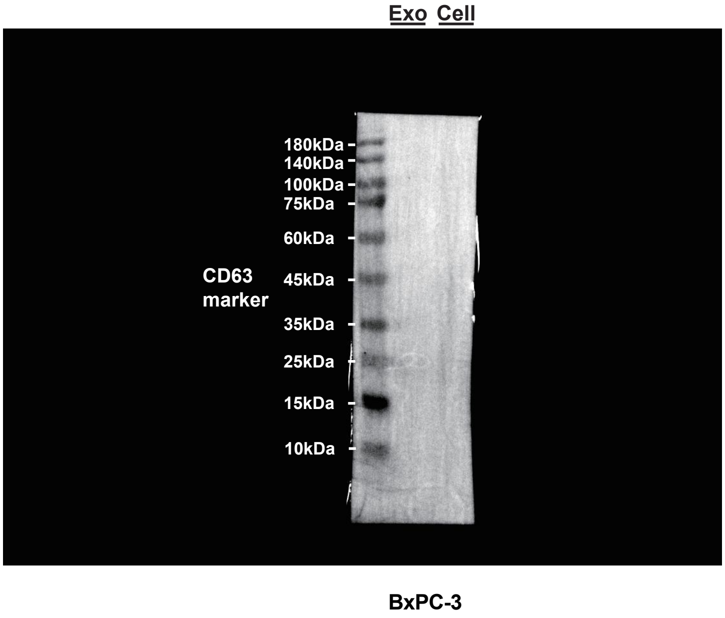

Repeat#1

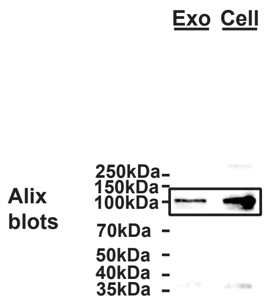


BxPC-3

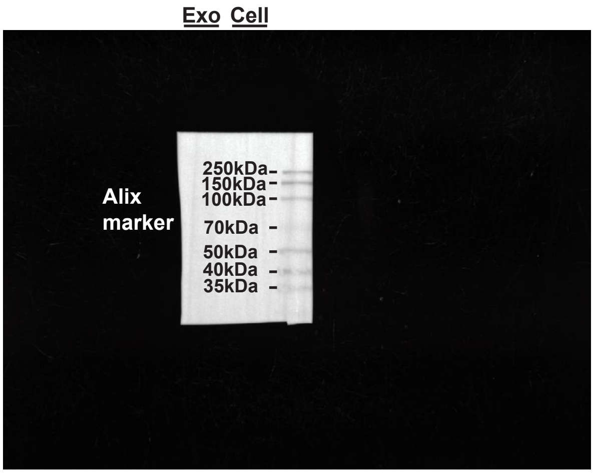


BxPC-3

Repeat#2

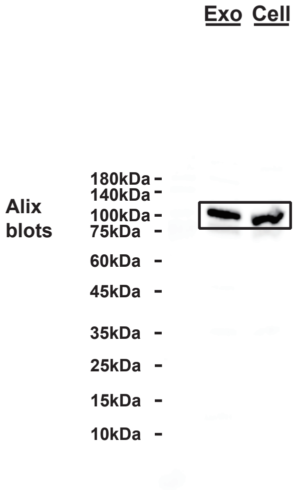


BxPC-3

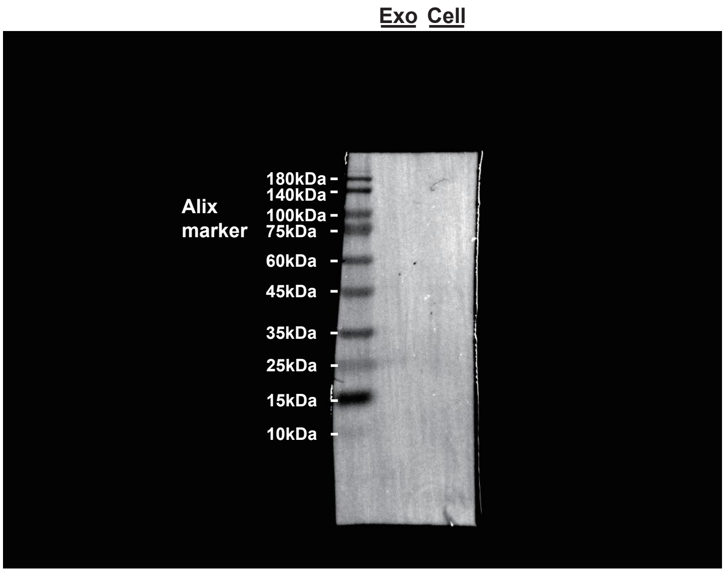


BxPC-3

Repeat#3

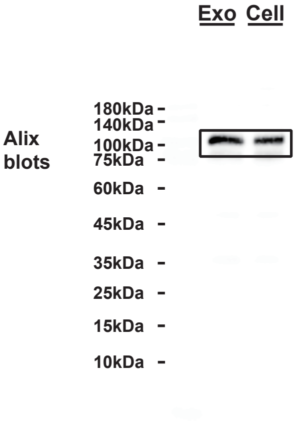


BxPC-3

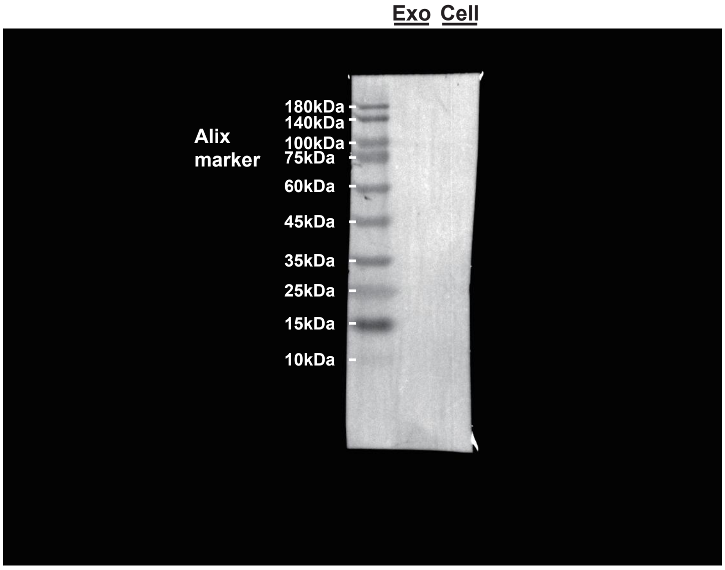


BxPC-3

Repeat#1

Exo Cell

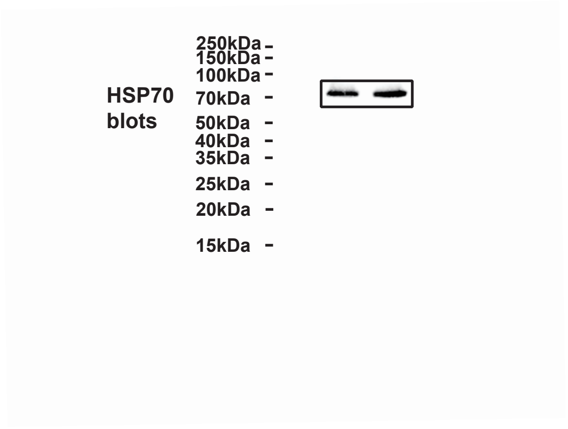


BxPC-3

Exo Cell

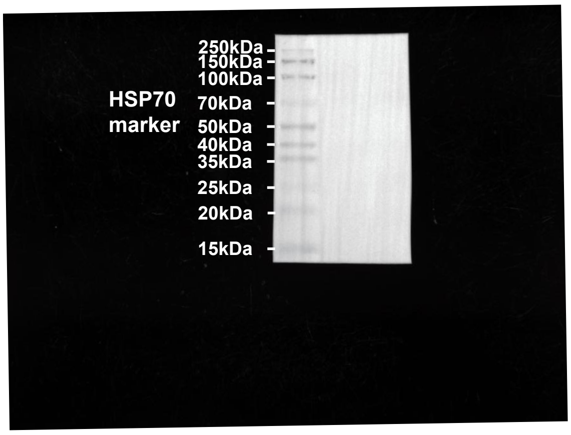


BxPC-3

Repeat#2

Exo Cell

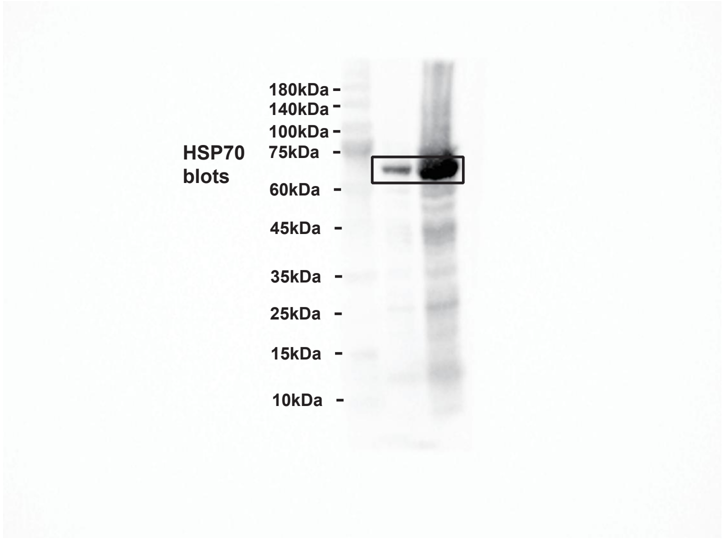


BxPC-3

Exo Cell

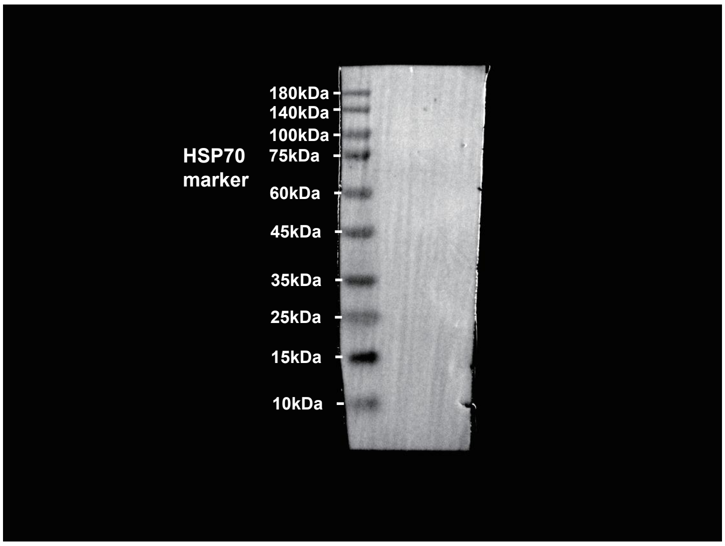


BxPC-3

Repeat#3

Exo Cell

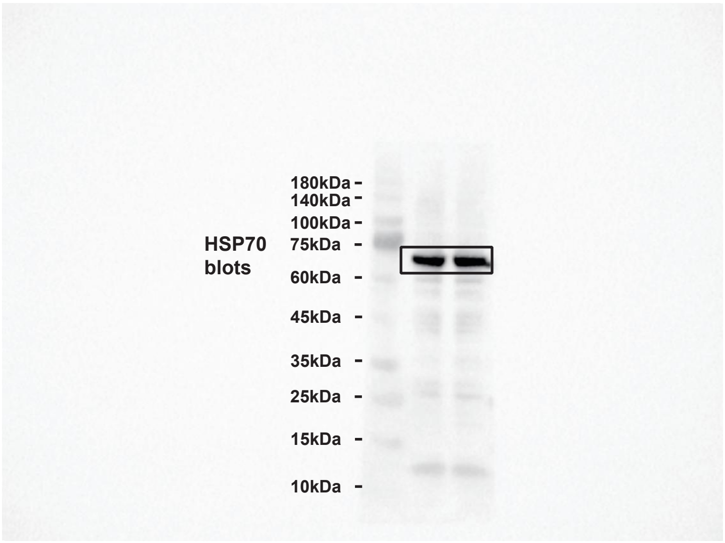


BxPC-3

Exo Cell

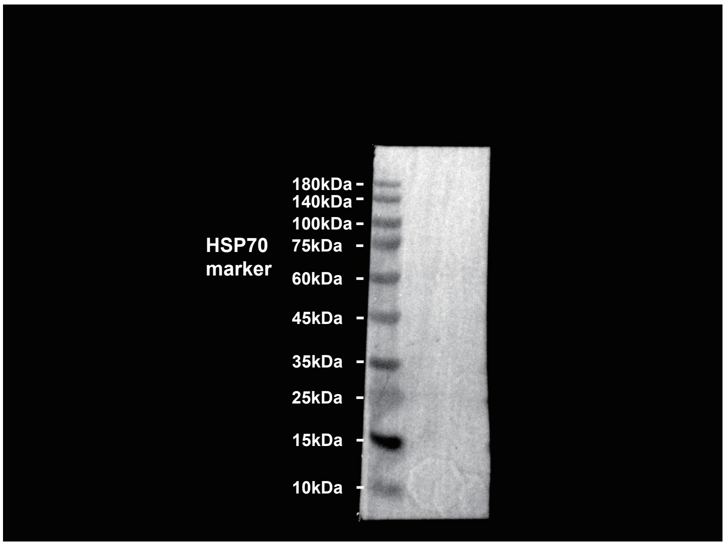


BxPC-3

## Repeat#1

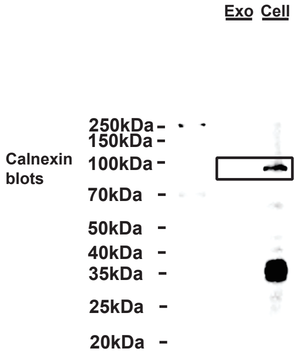


BxPC-3

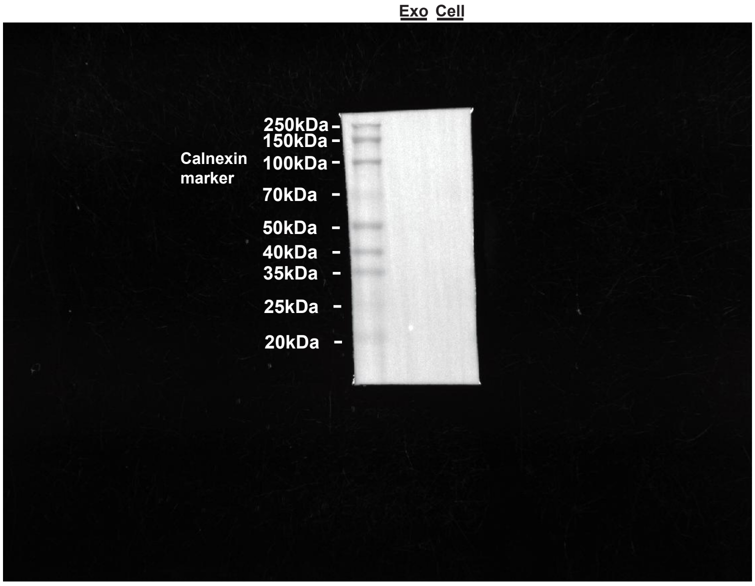


BxPC-3

## Repeat#2

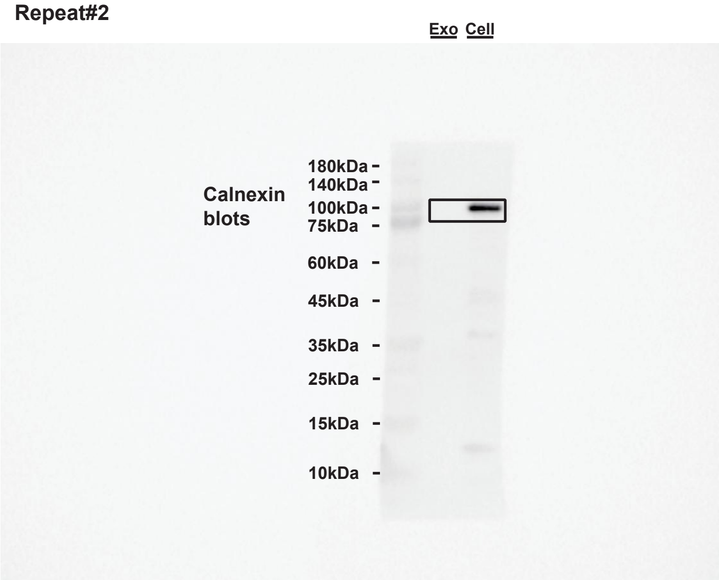


BxPC-3

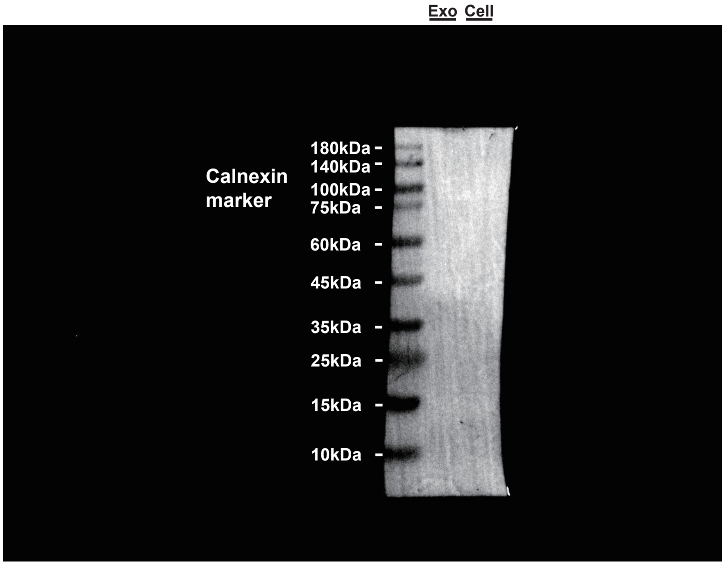


BxPC-3

## Repeat#3

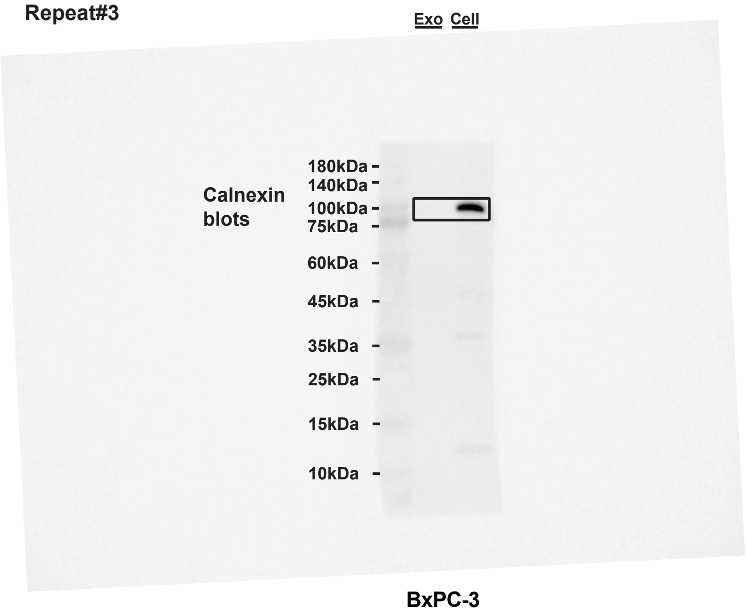


BxPC-3

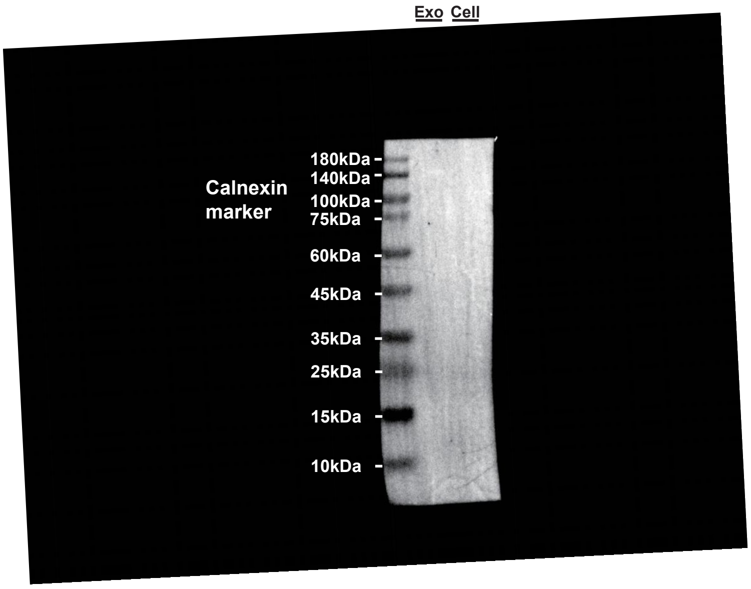


BxPC-3

**Fig. 2E**

Repeat#1

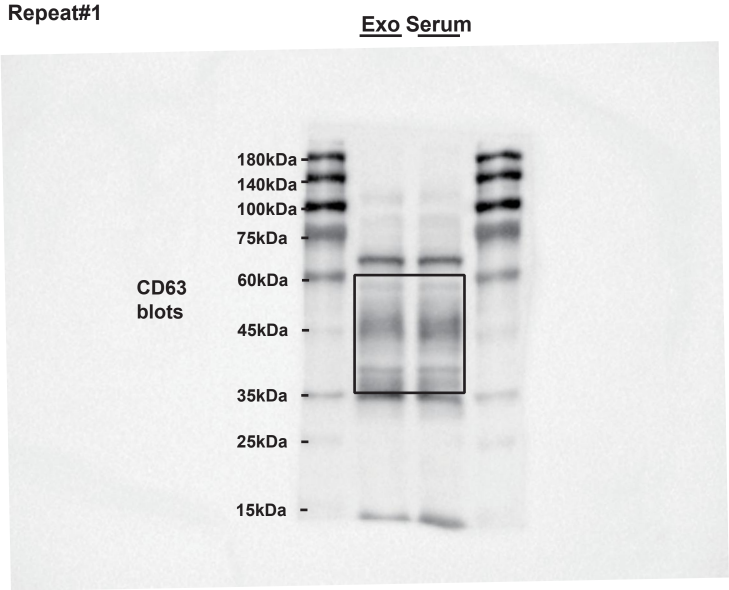


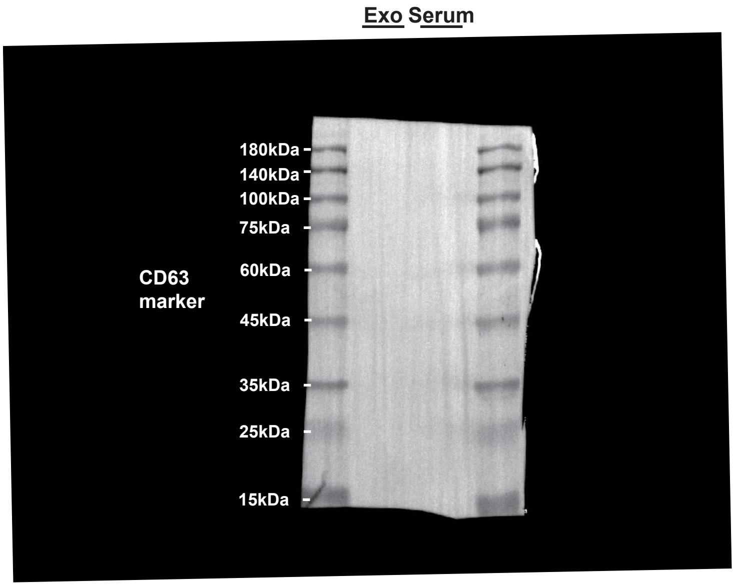


Repeat#2

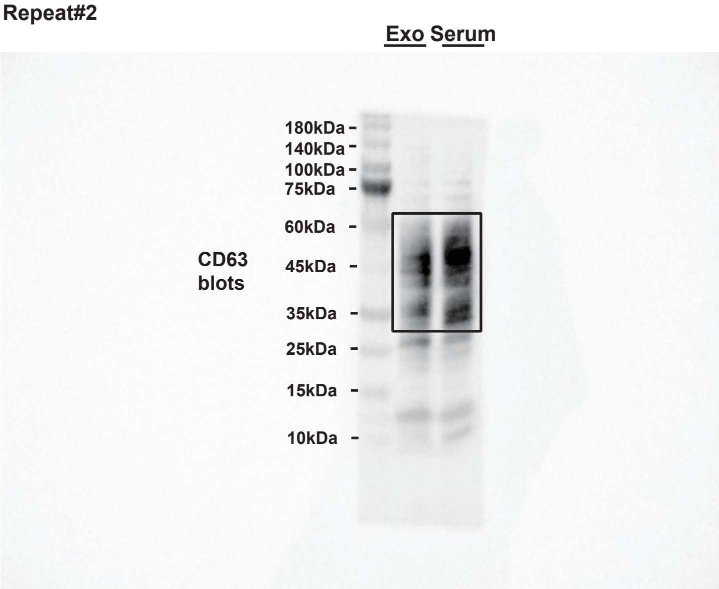


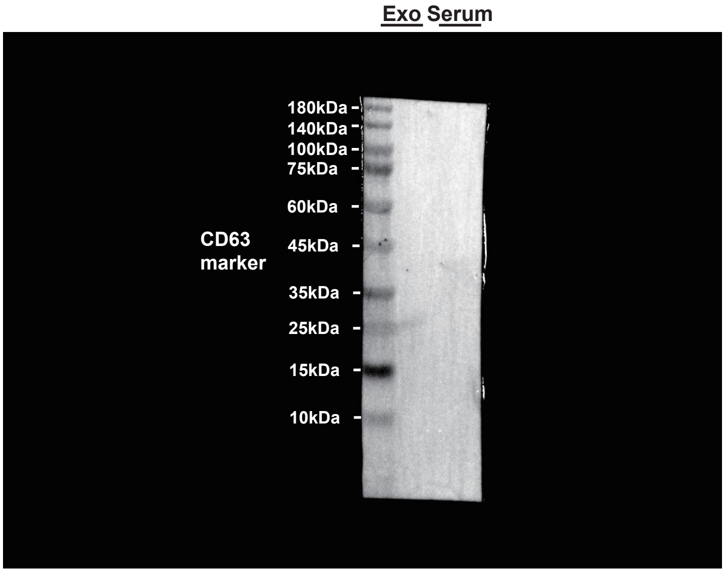


Repeat#3

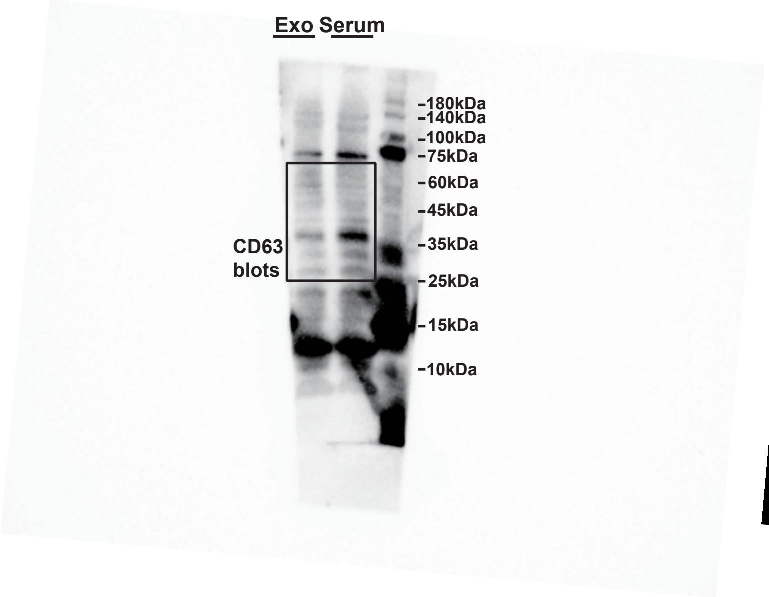


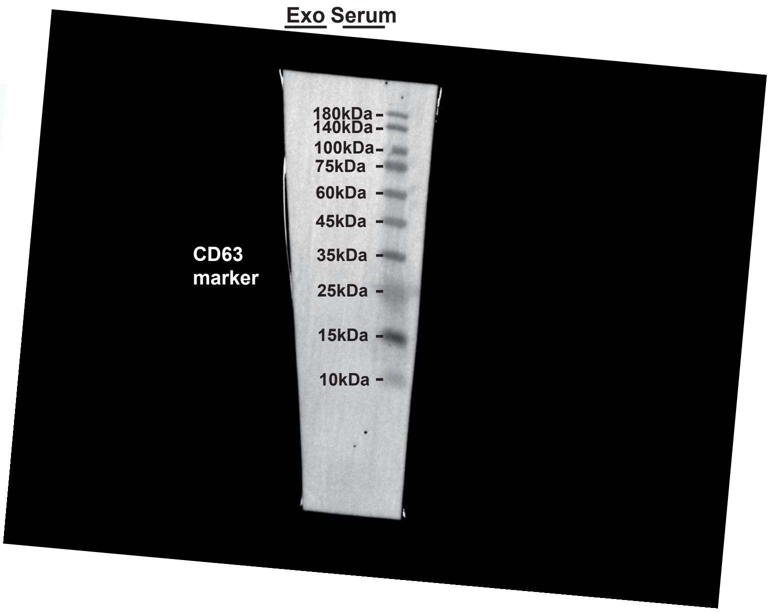

**Repeat#1**

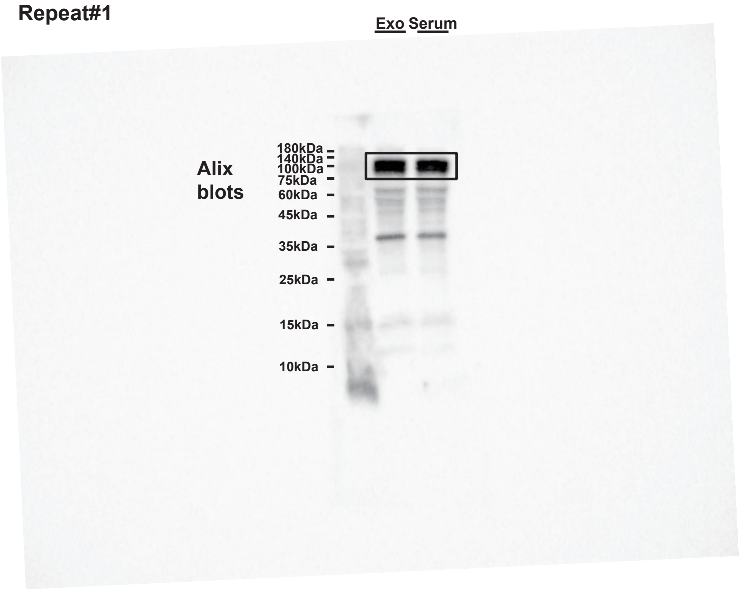


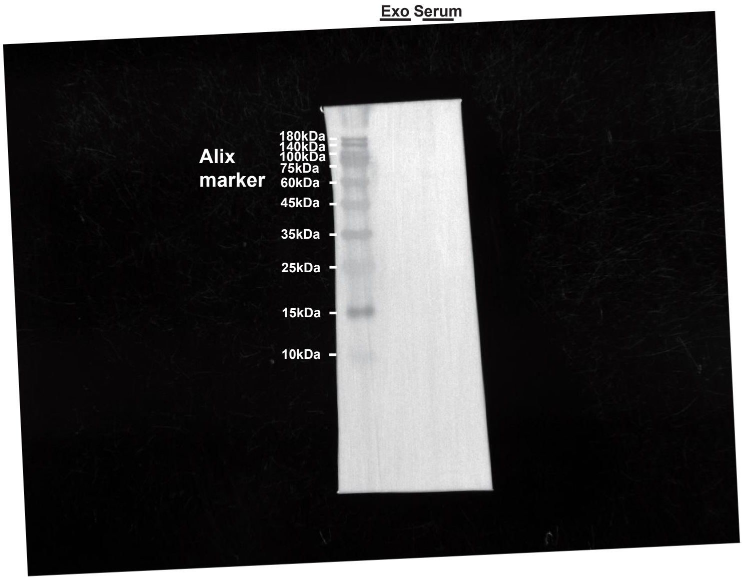


**Repeat#2**

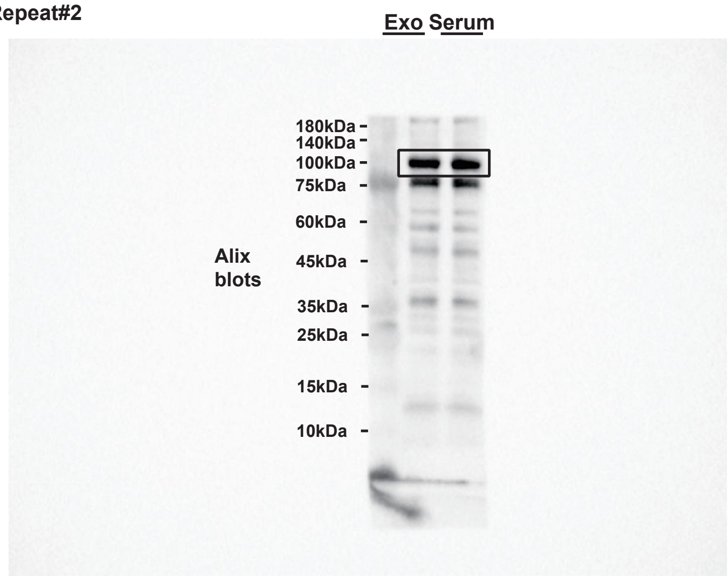


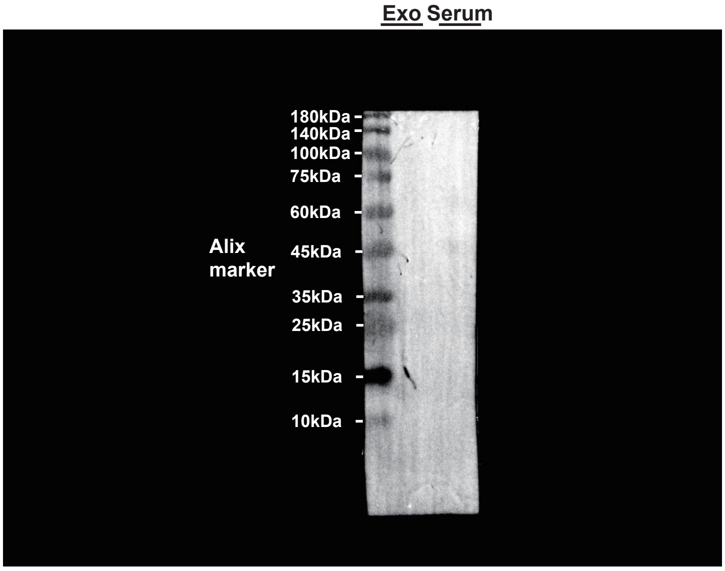


**Repeat#3**

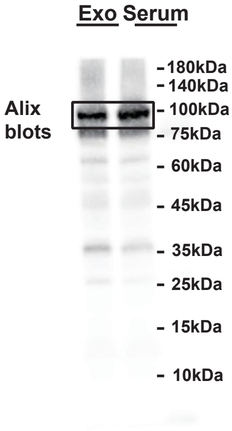


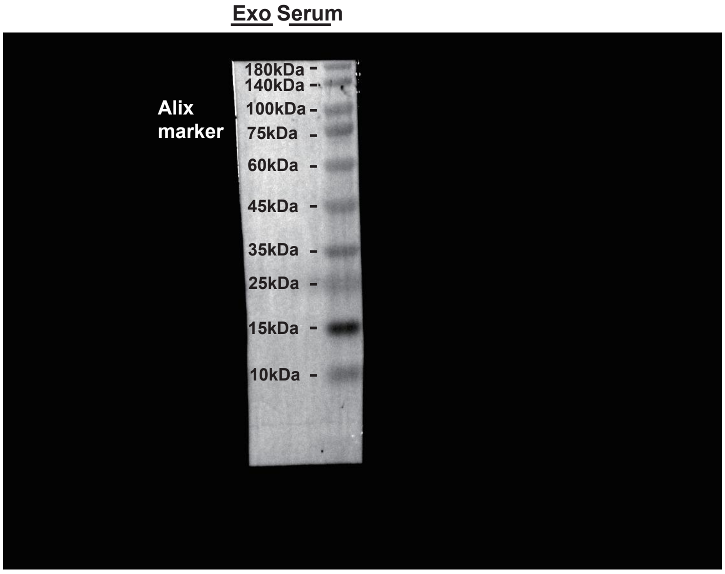

Repeat#1

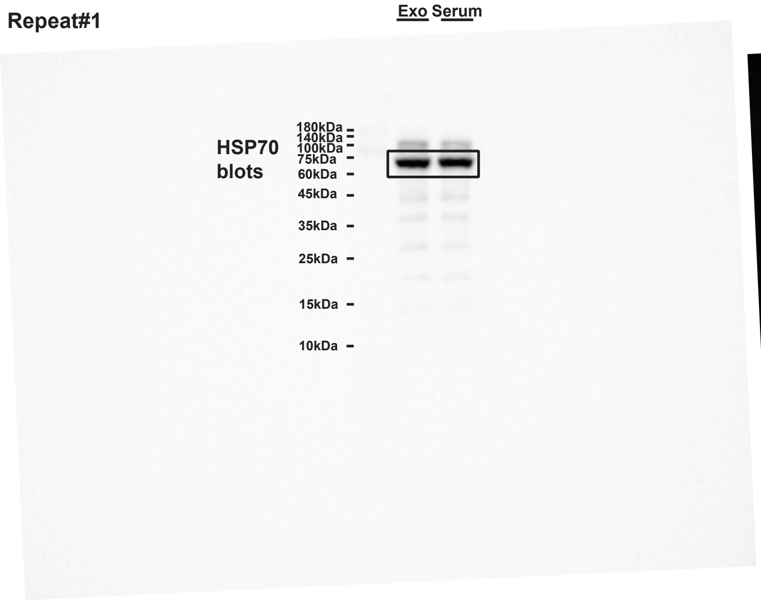


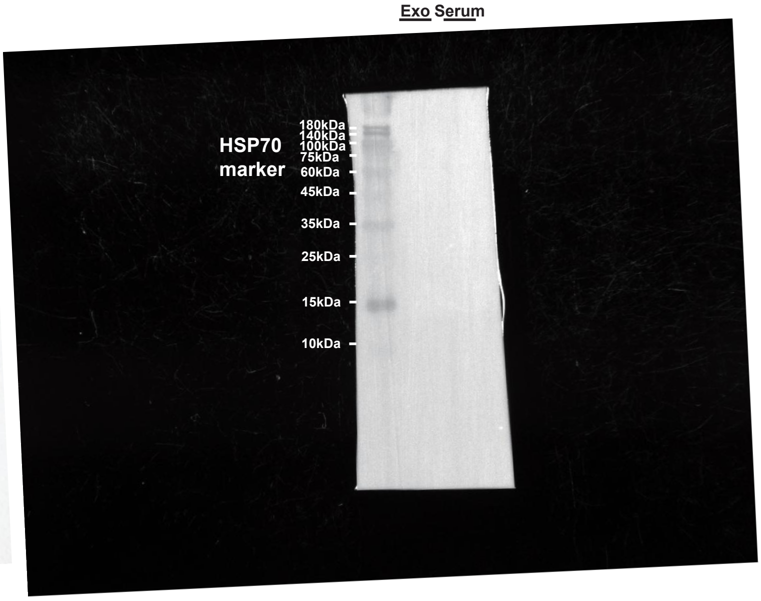


Repeat#2

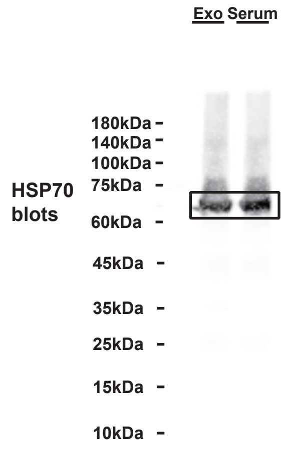


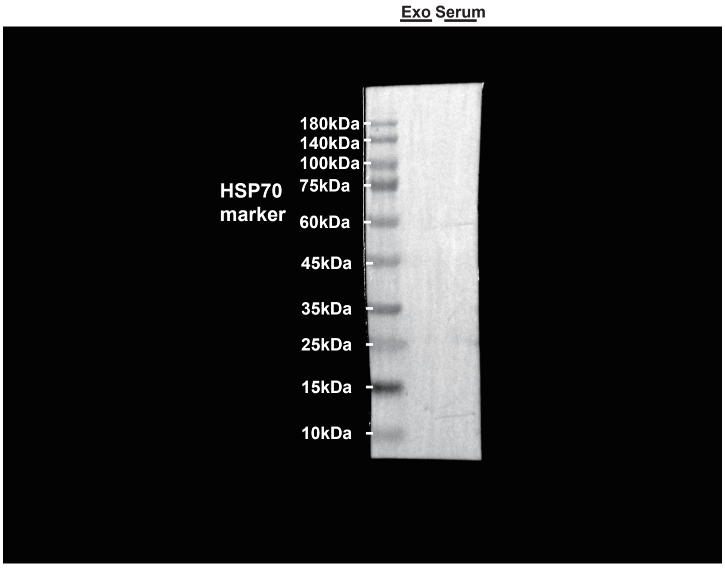


Repeat#3

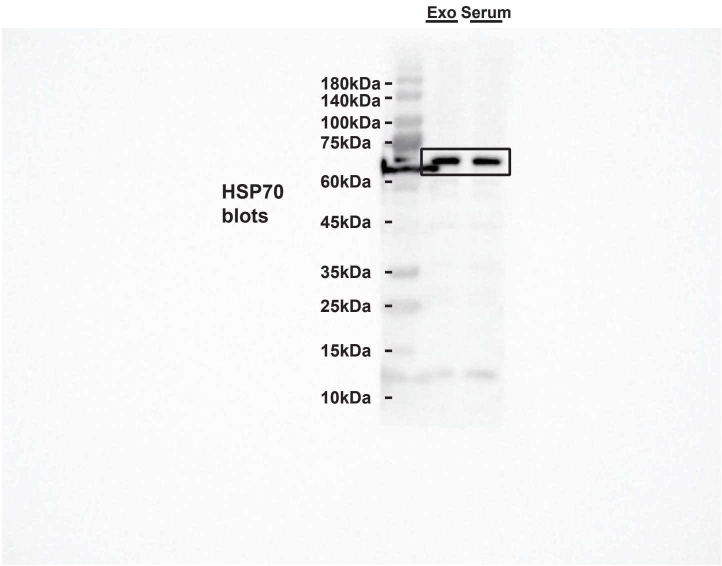


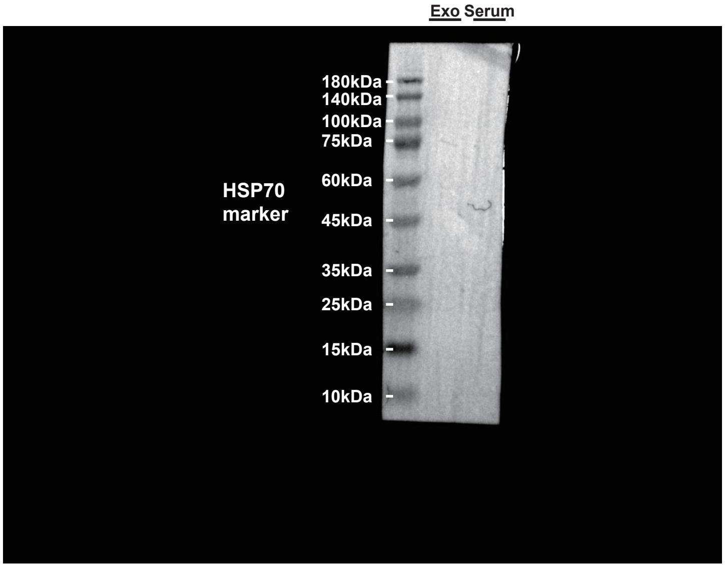

Repeat#1

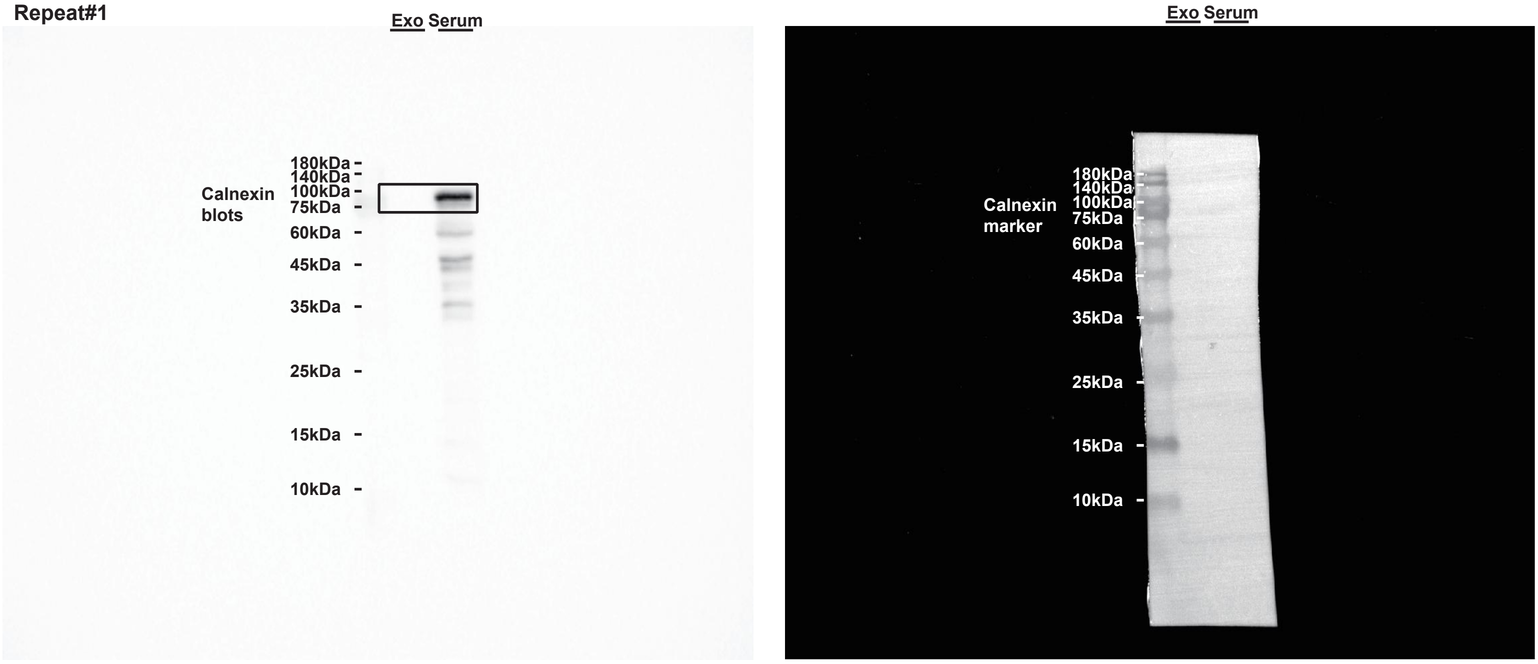


Repeat#2

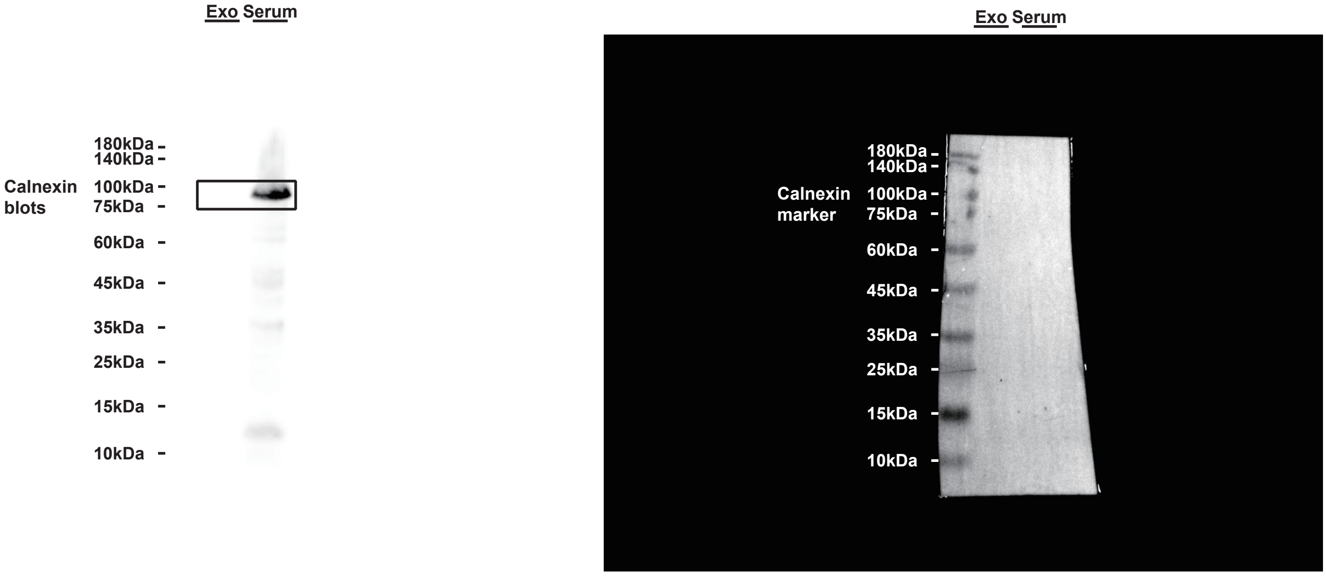


Repeat#3

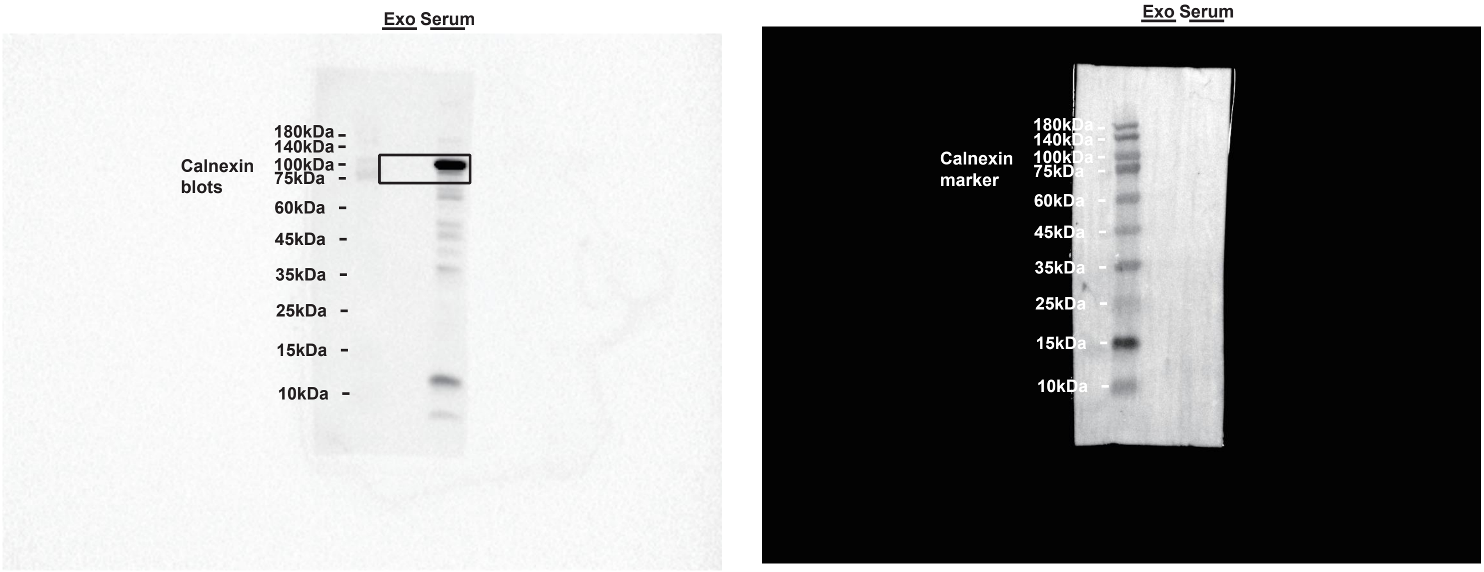

## Repeat#1

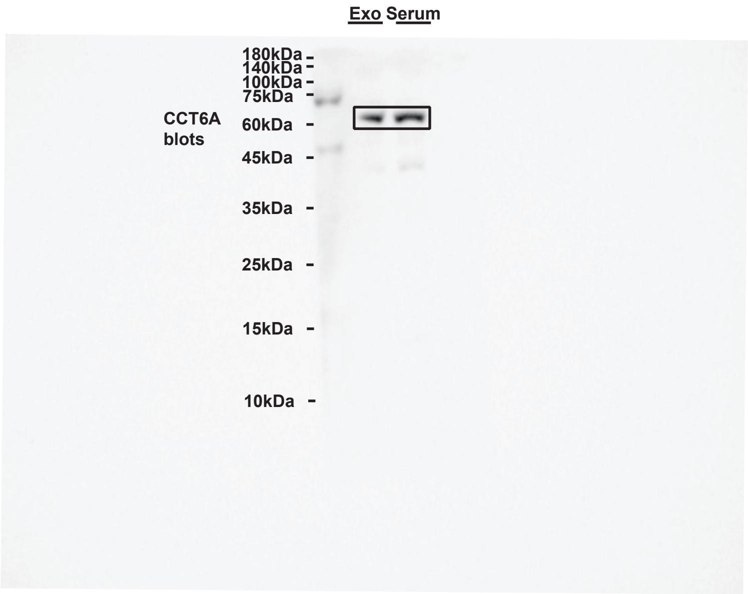


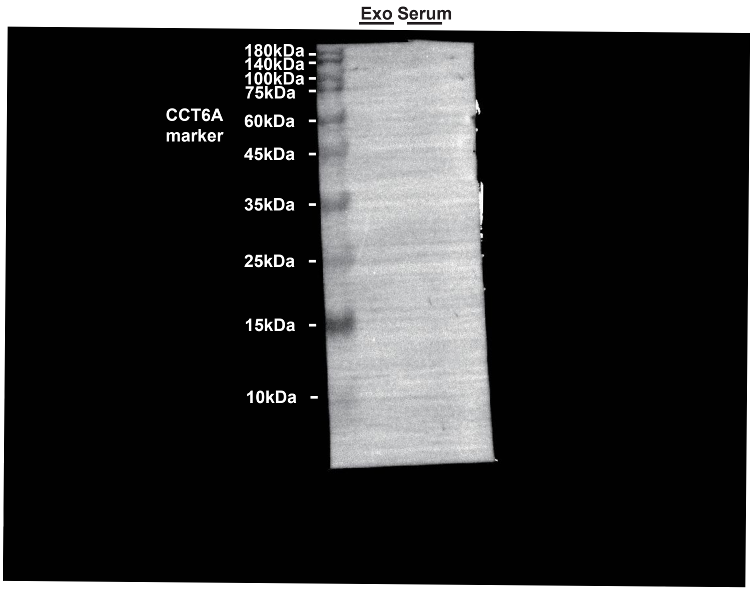


## Repeat#2

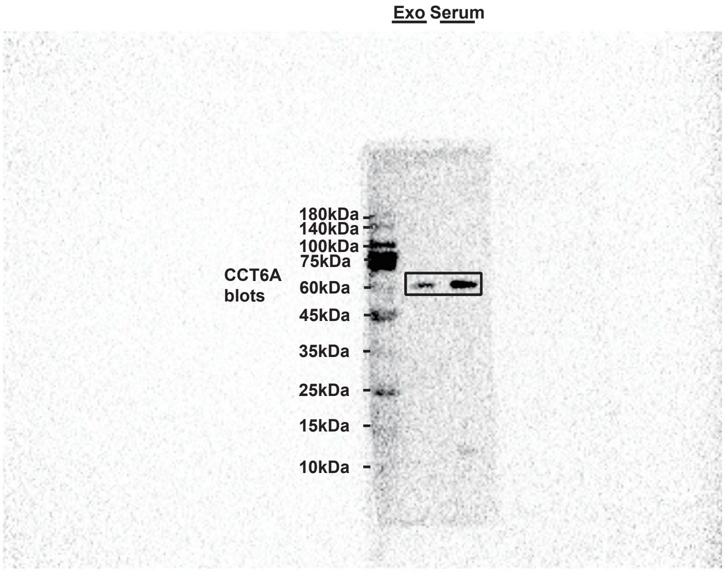


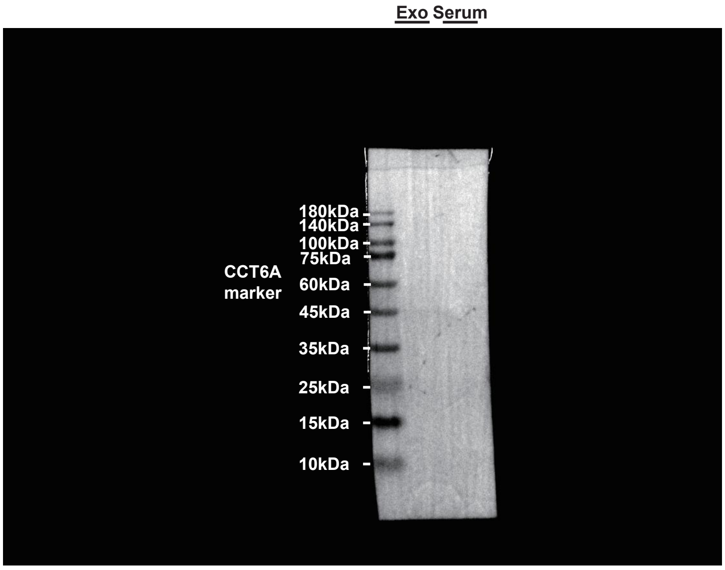


## Repeat#3

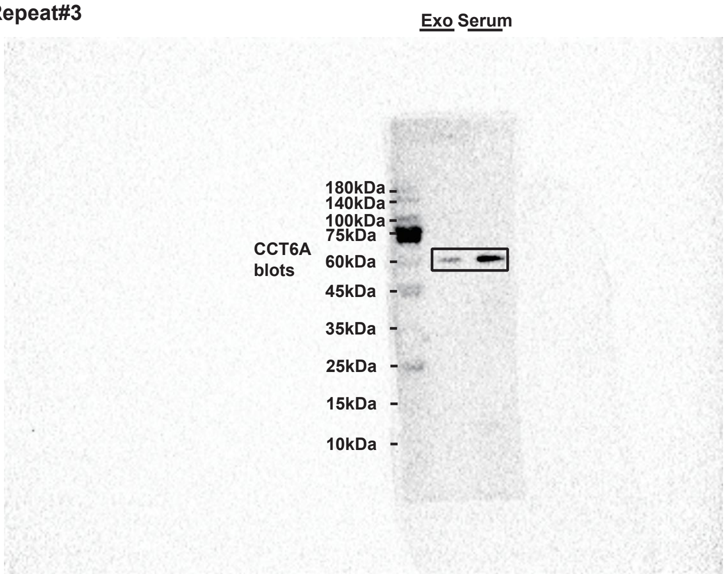


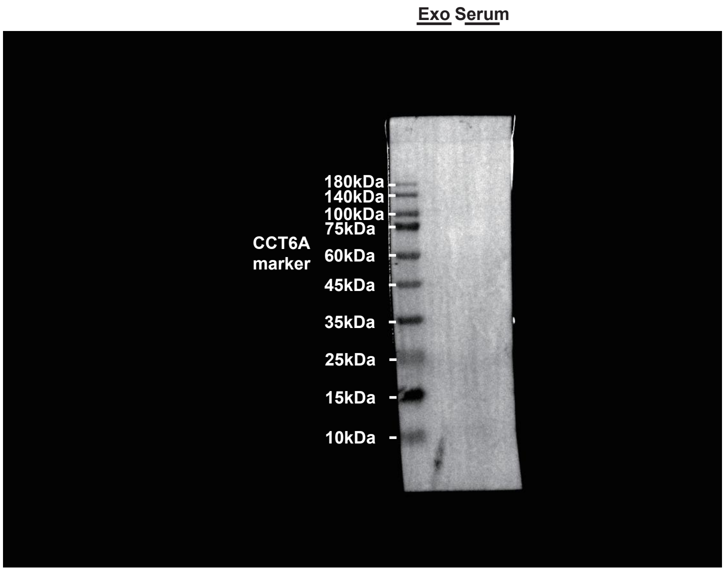

Repeat#1

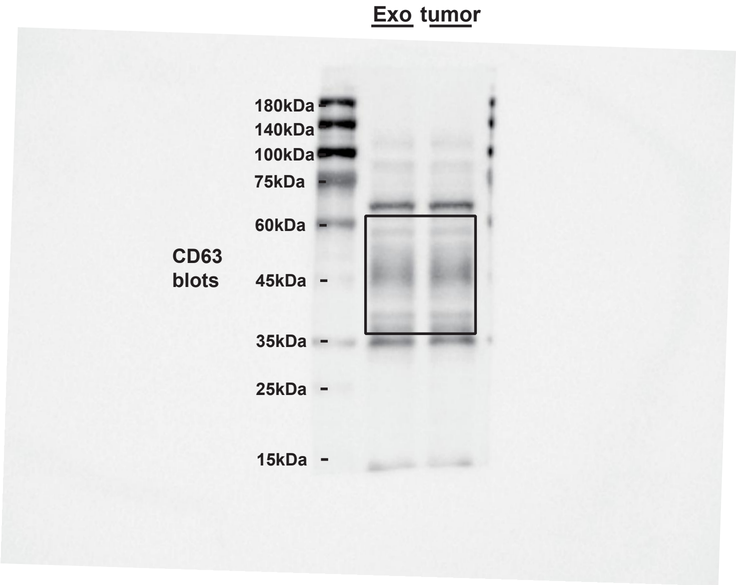


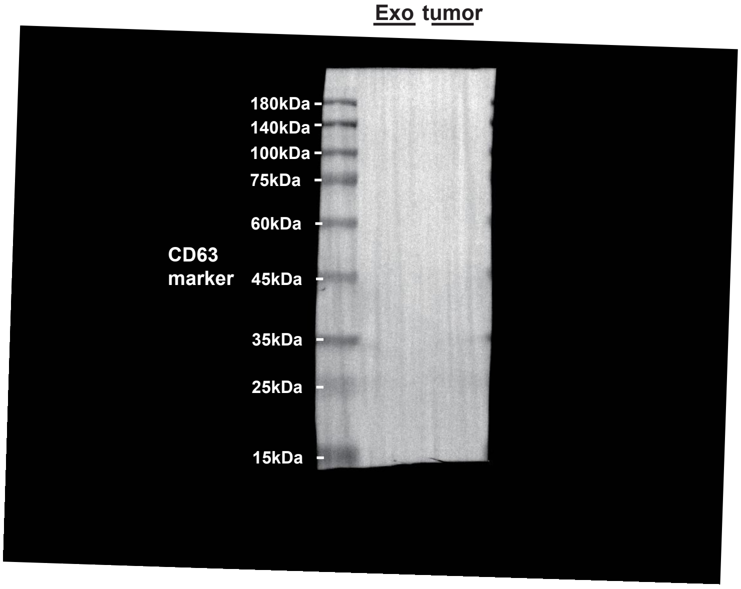


Repeat#2

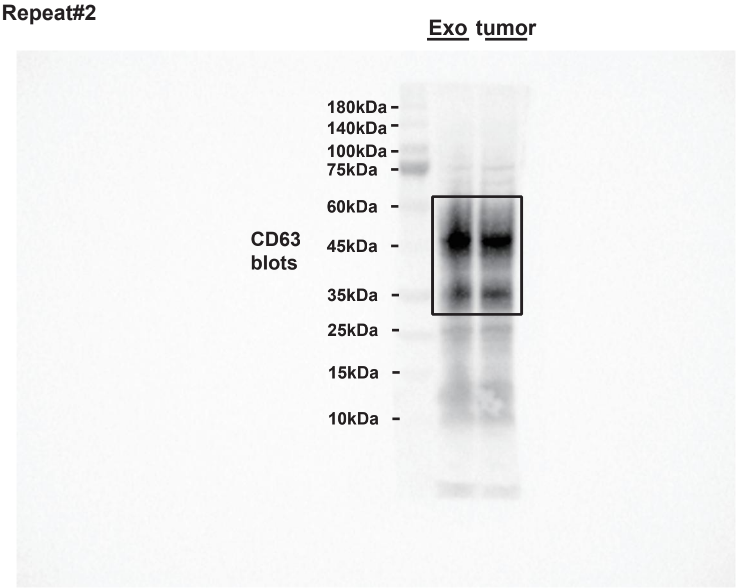


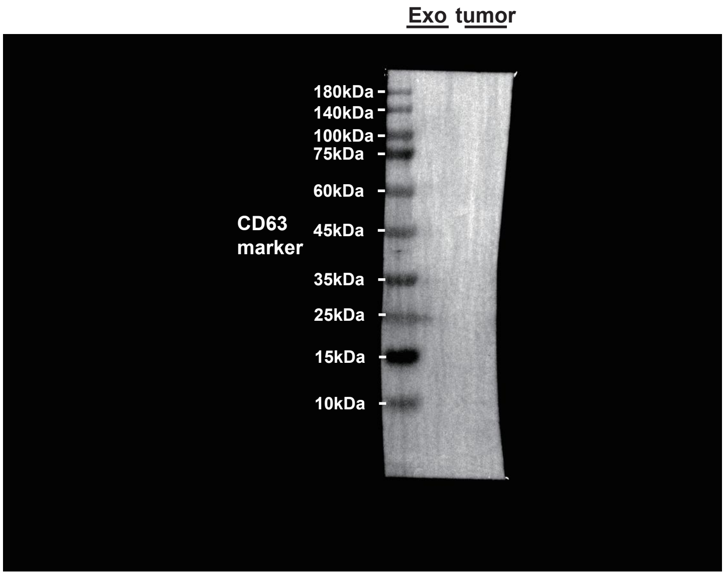


Repeat#3

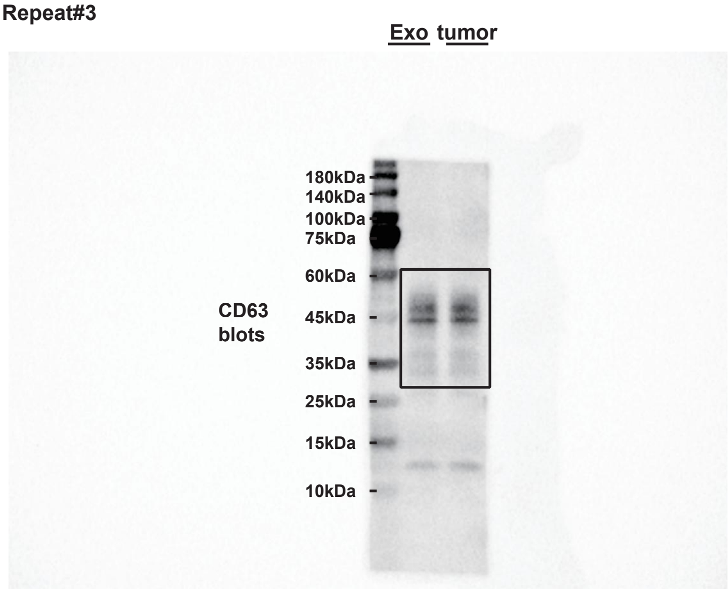


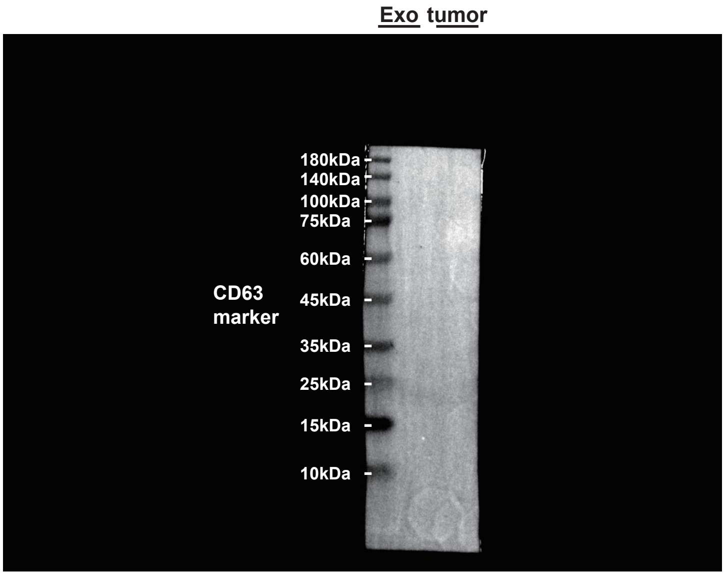

Repeat#1

Exo tumor

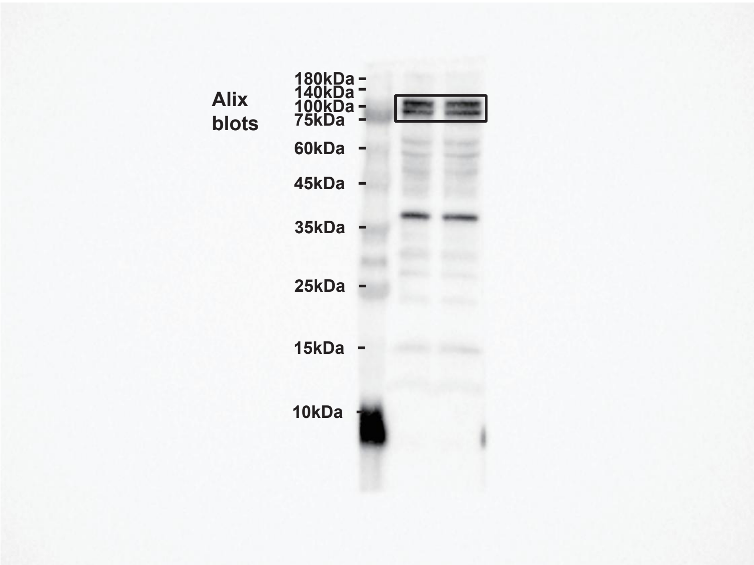


Exo tumor

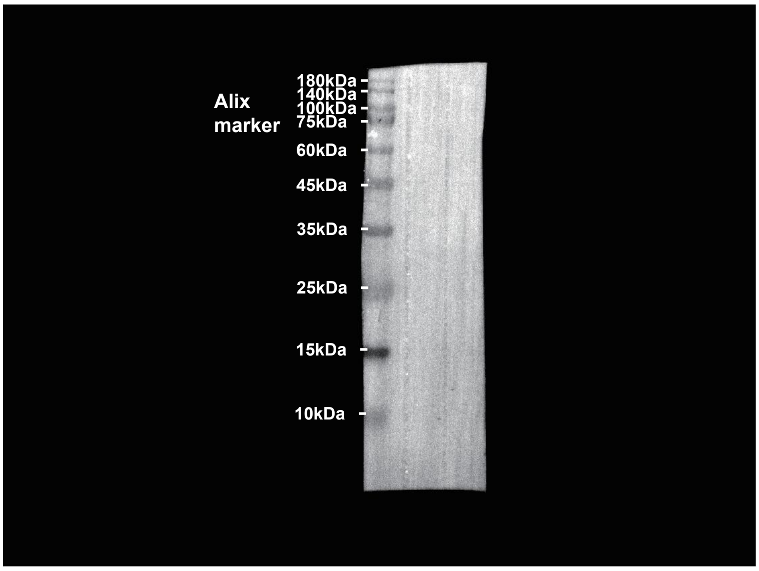


Repeat#2

Exo tumor

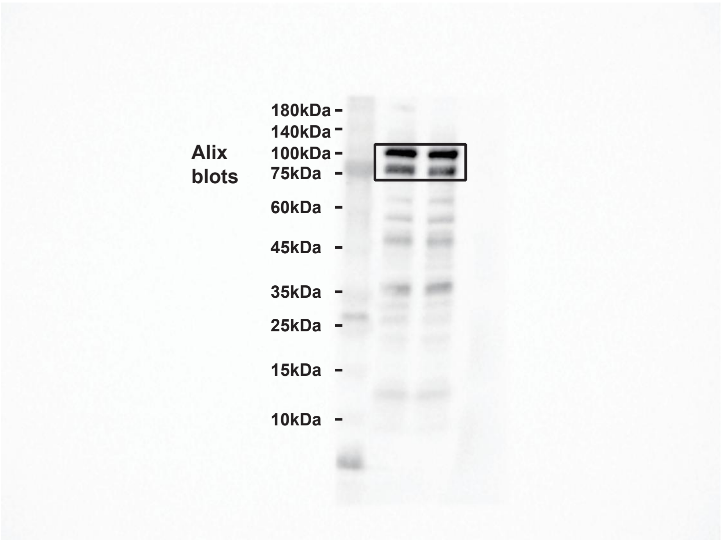


Exo tumor

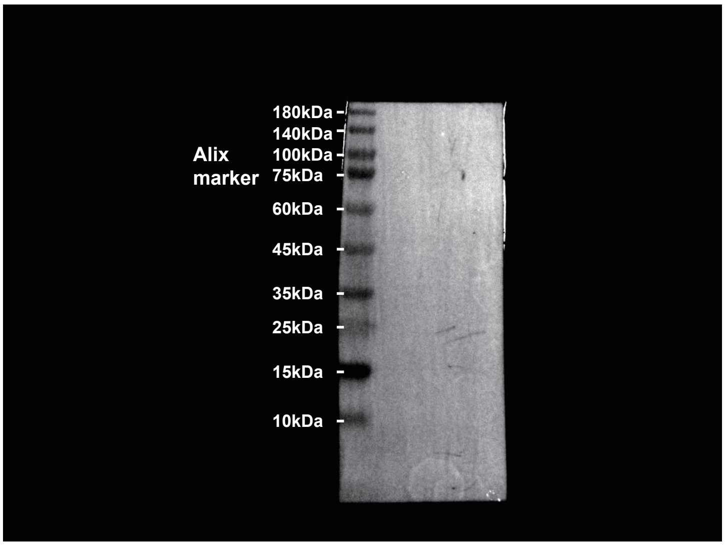


Repeat#3

Exo tumor

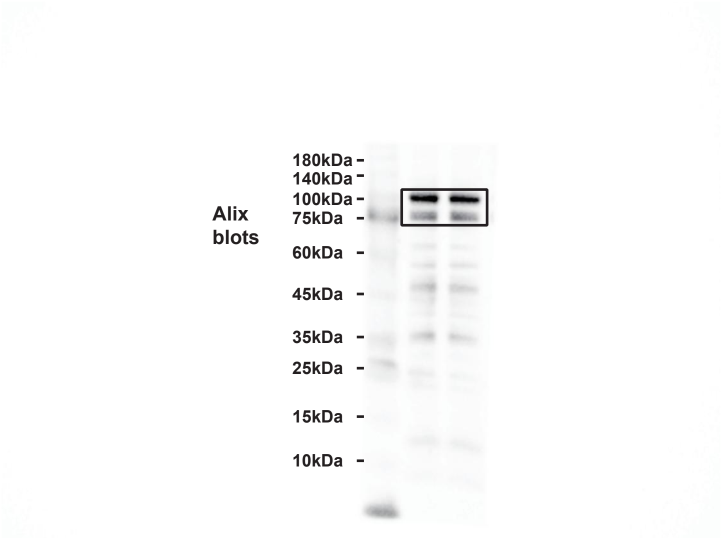


Exo tumor

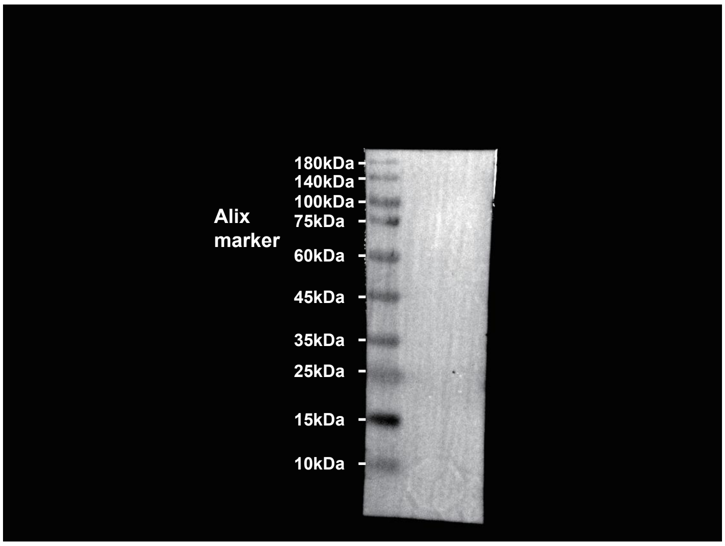

**Repeat#1**

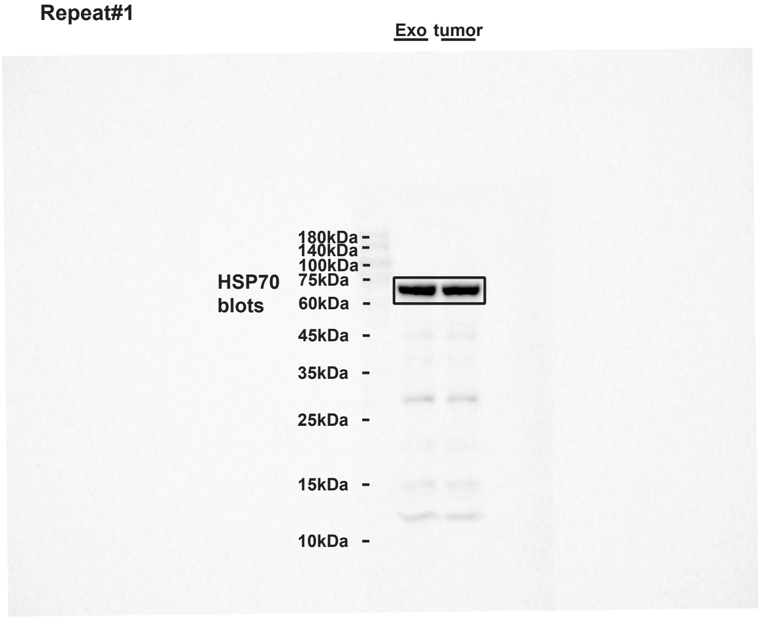


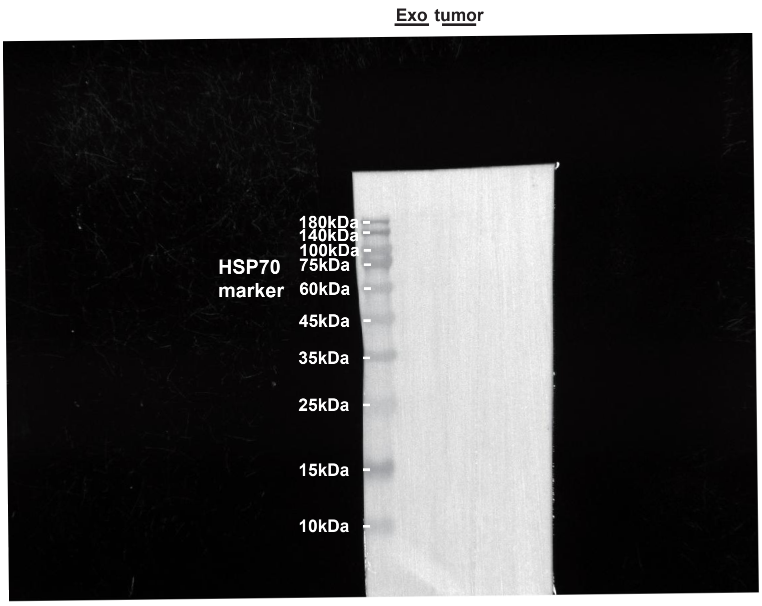


**Repeat#2**

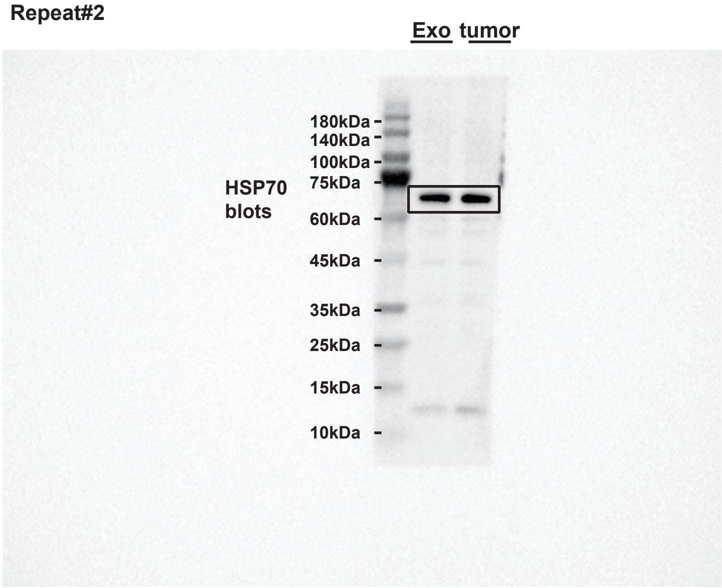


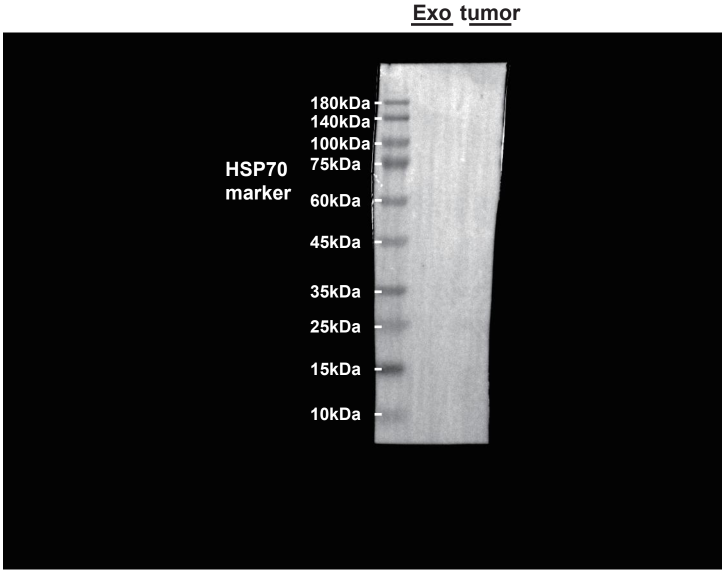


**Repeat#3**

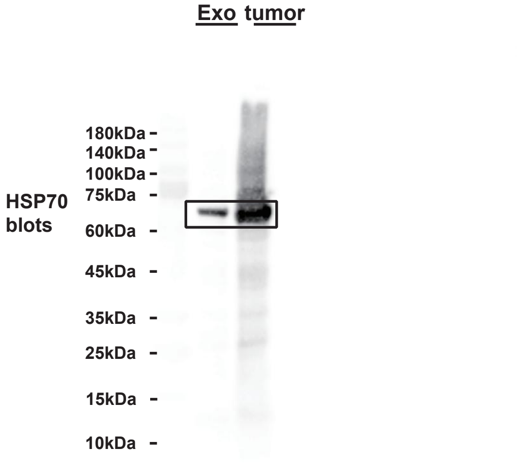


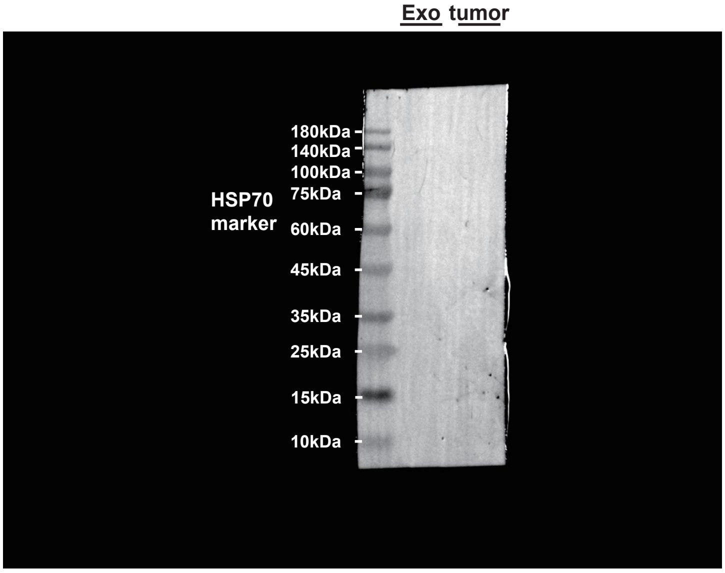

**Repeat#1**

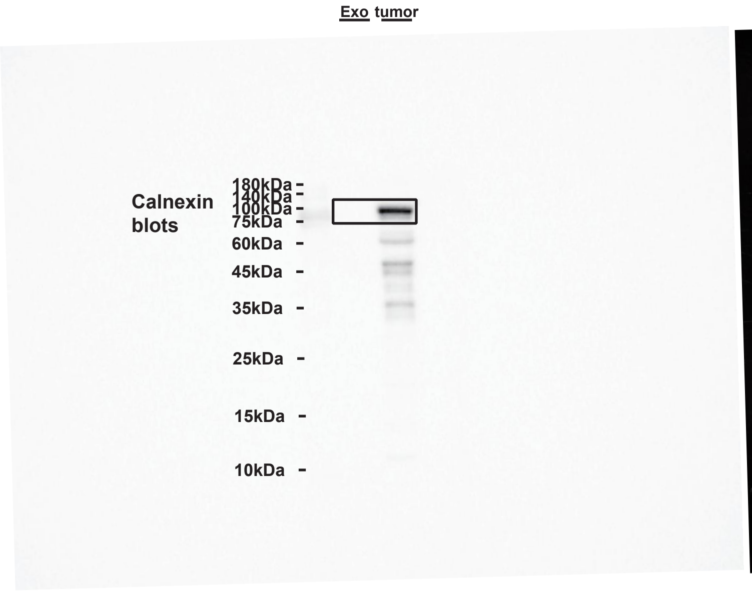


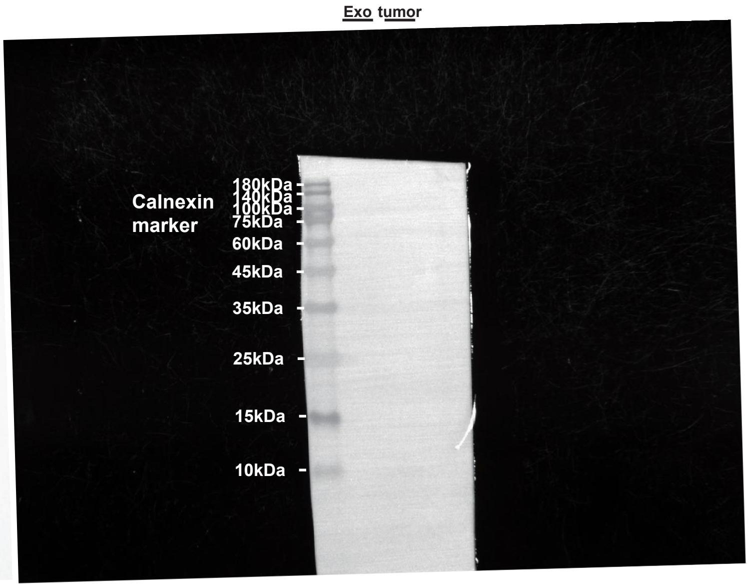


**Repeat#2**

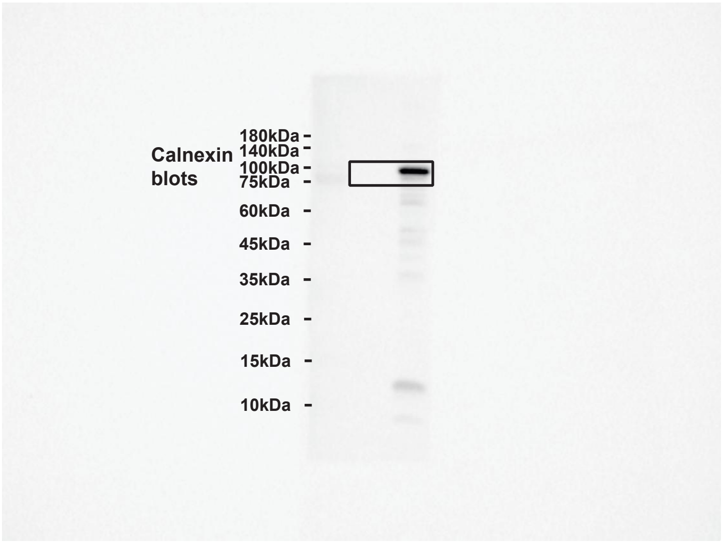


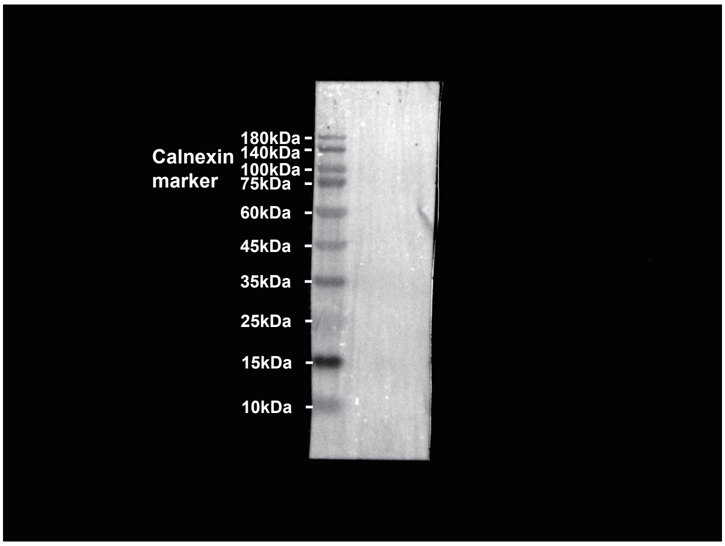


**Repeat#3**

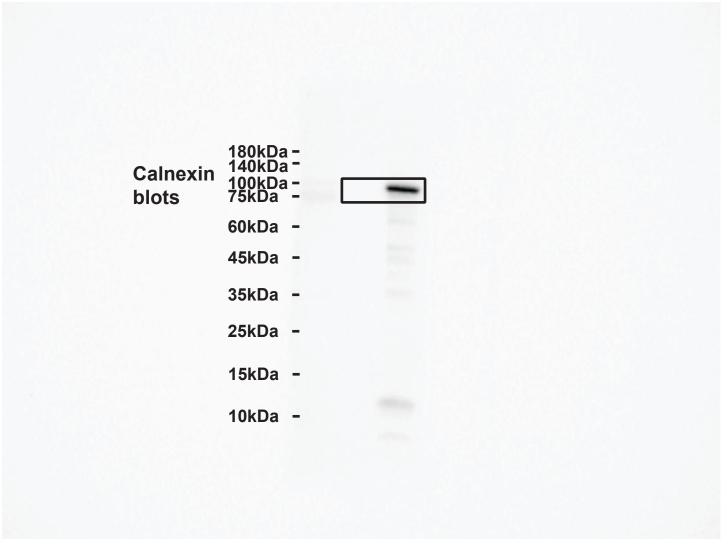


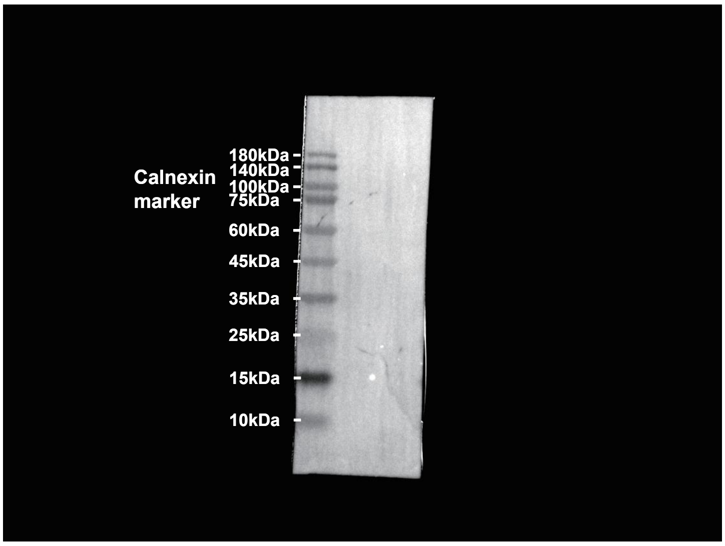

Repeat#1

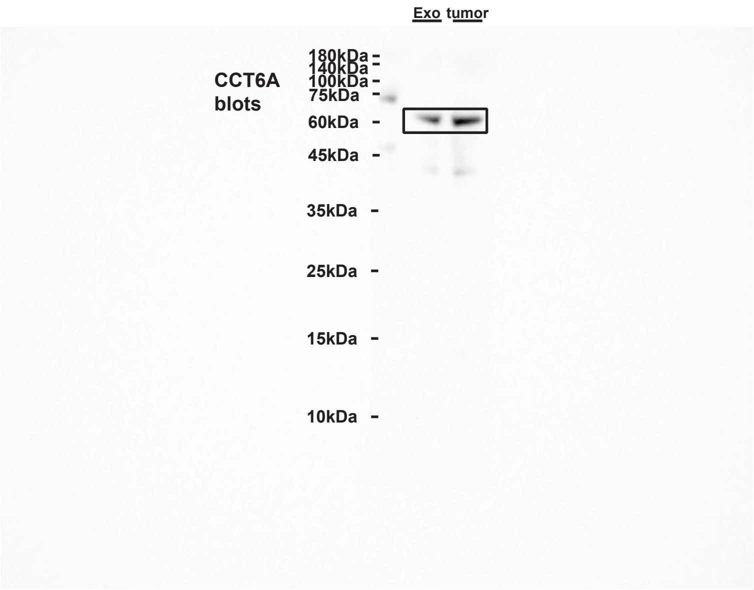


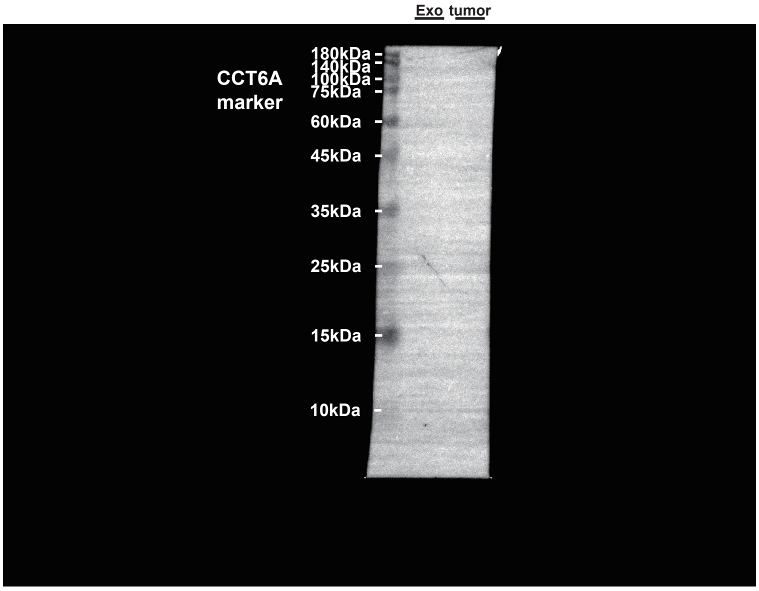


Repeat#2

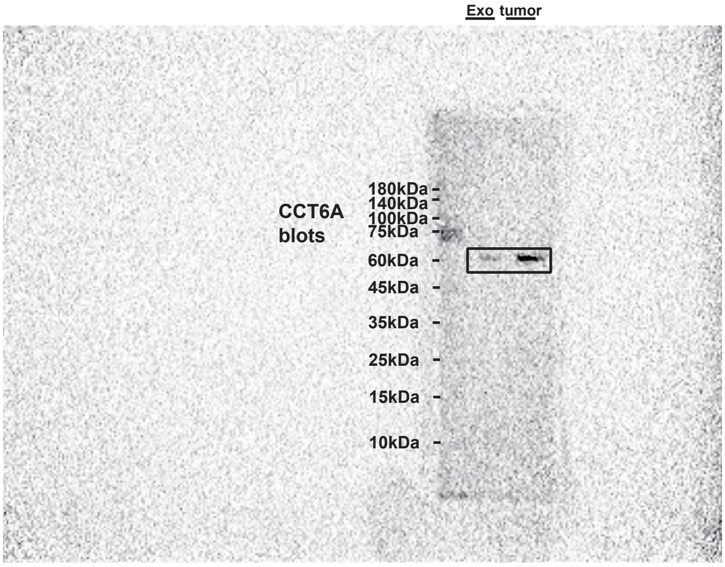


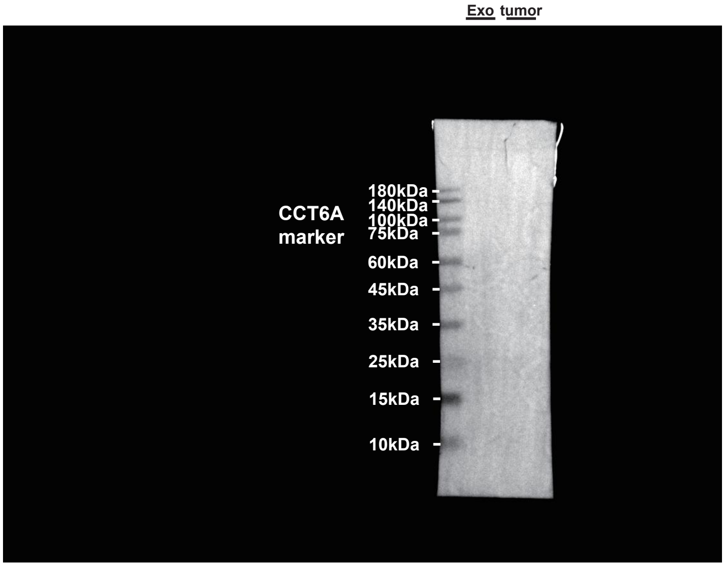


Repeat#3

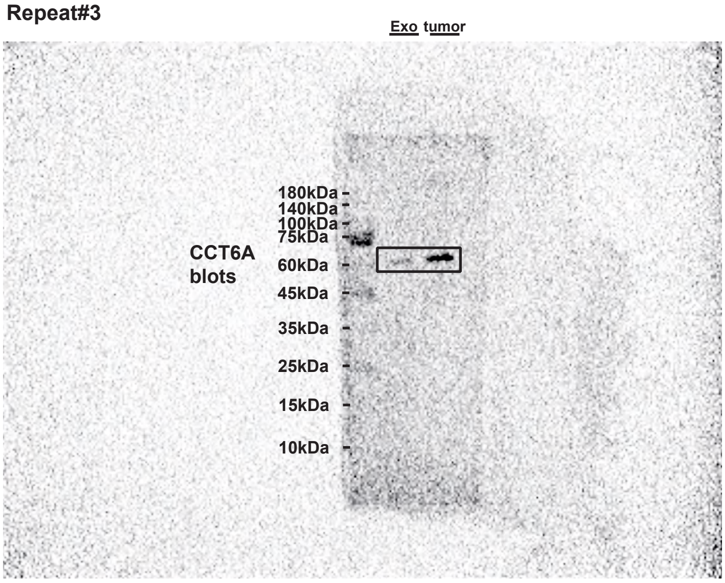


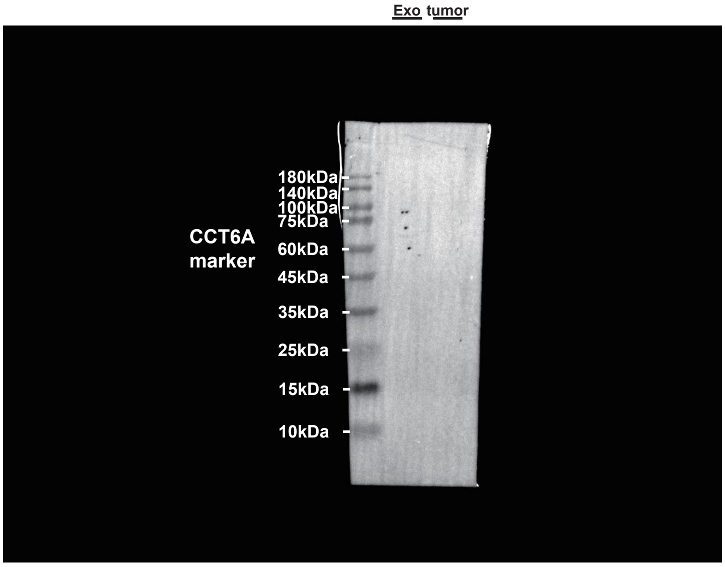

**Fig. 2G**

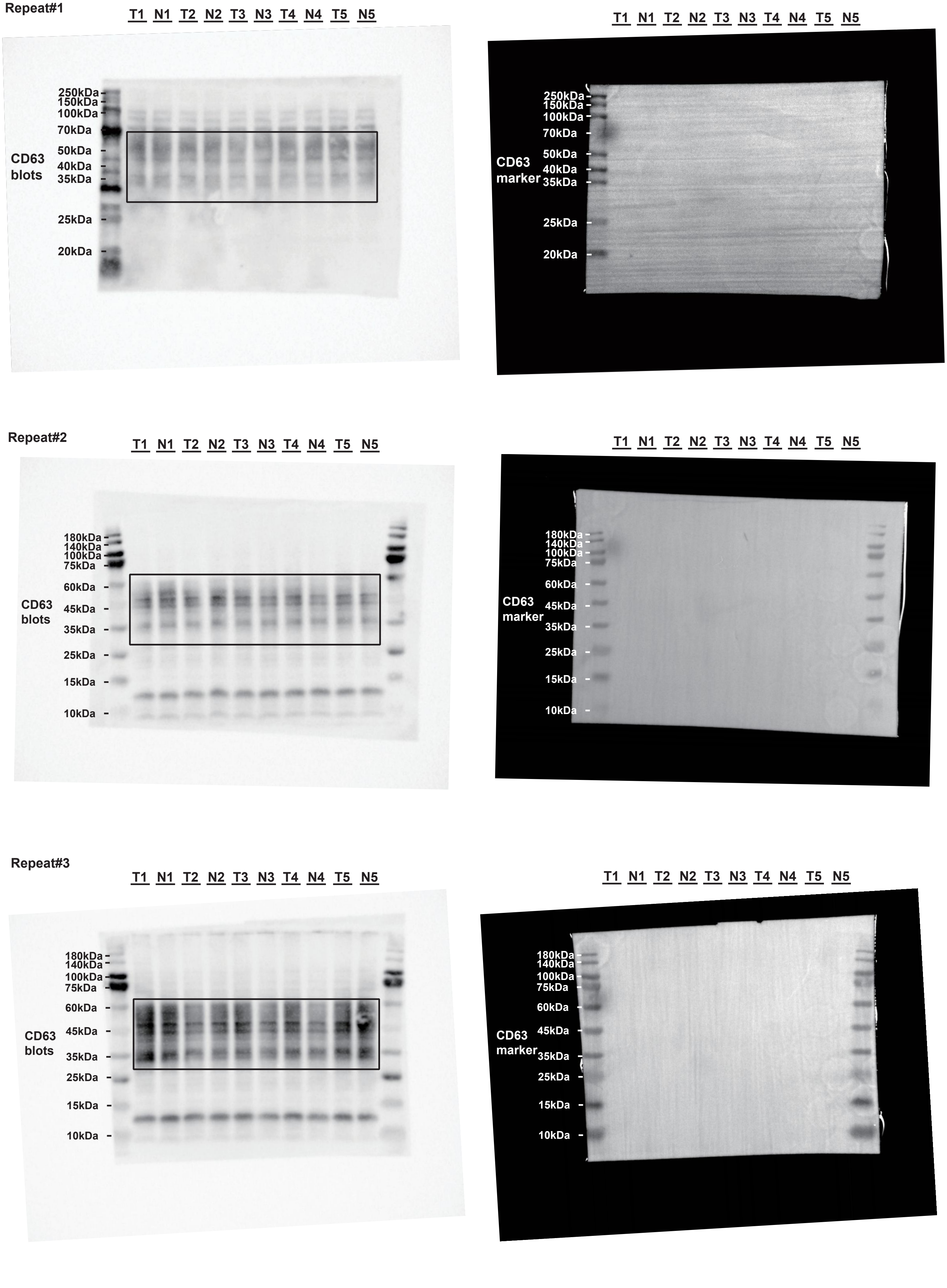


Note: Some of the Western blot membranes were re-probed after stripping with a stripping buffer; hence, the membranes in the bright-field images containing markers are identical.

Repeat#1

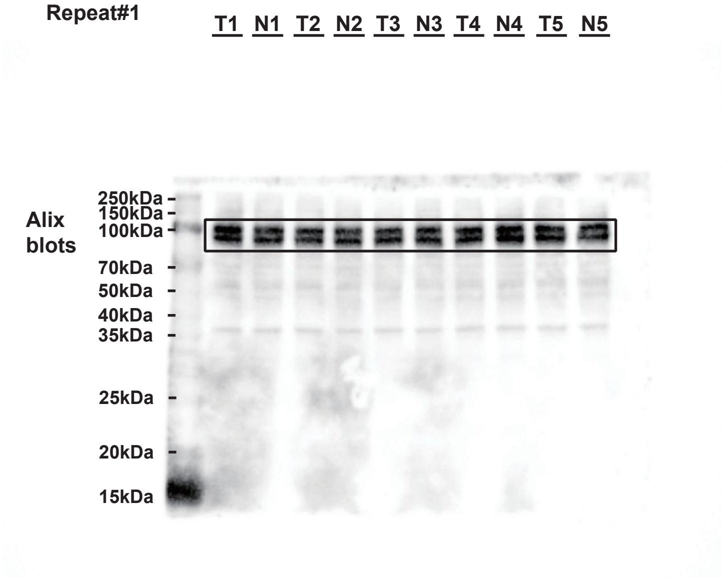


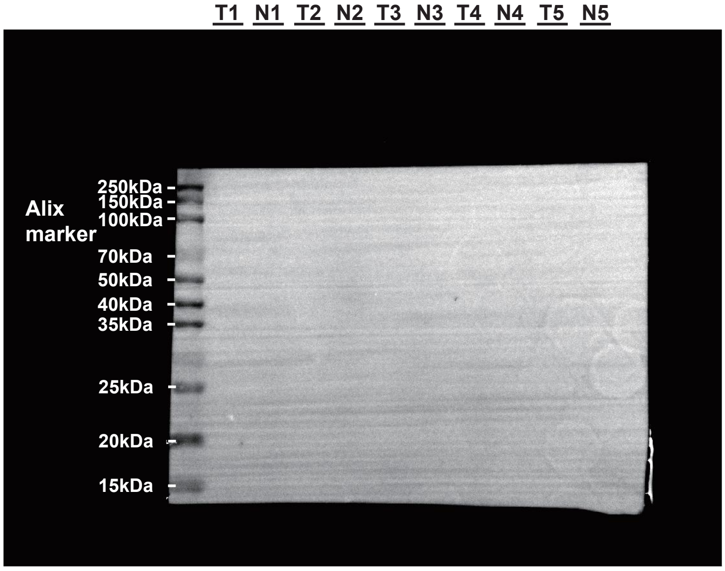


Repeat#2

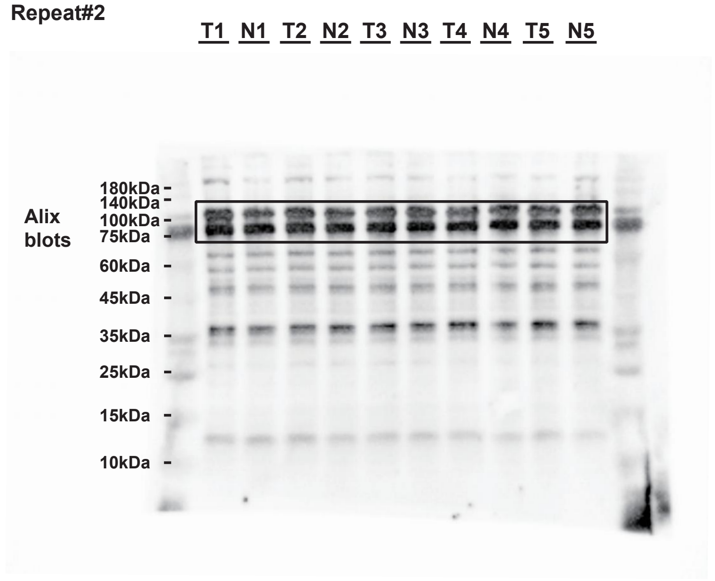


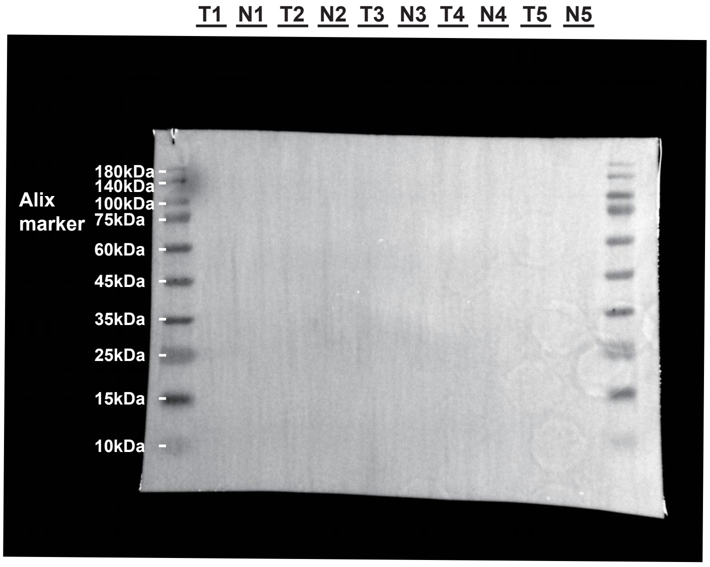


Repeat#3

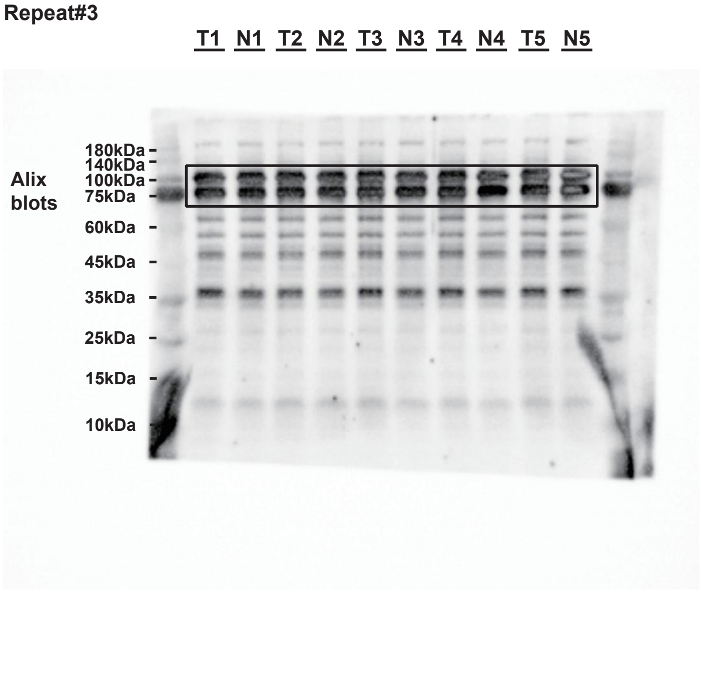


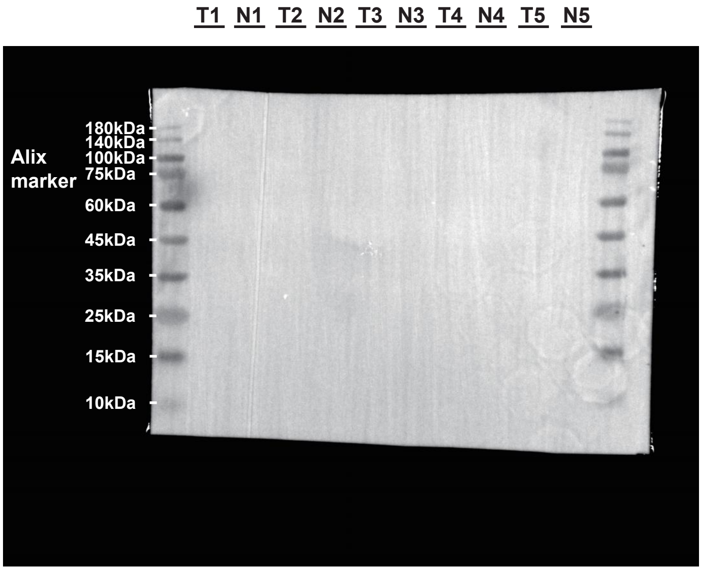

Repeat#1

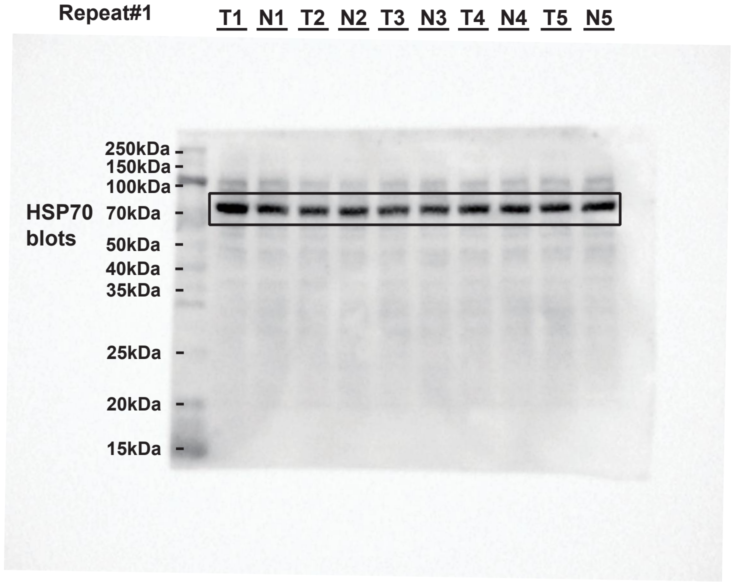


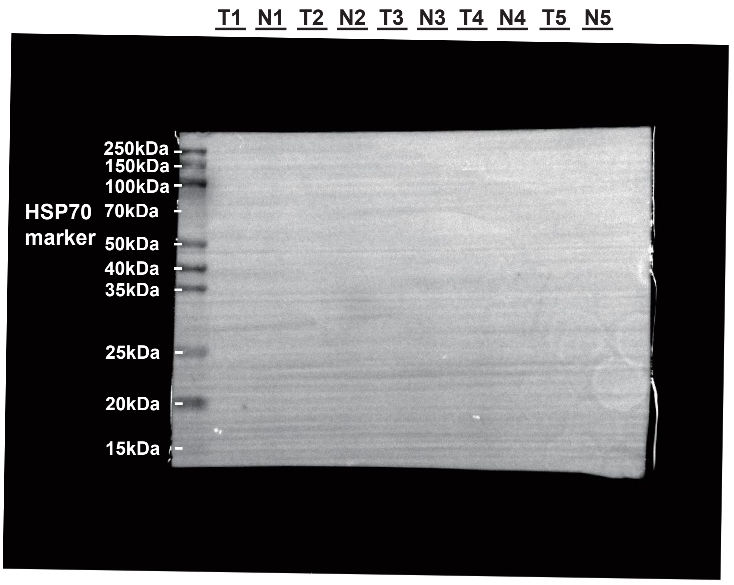


Repeat#2

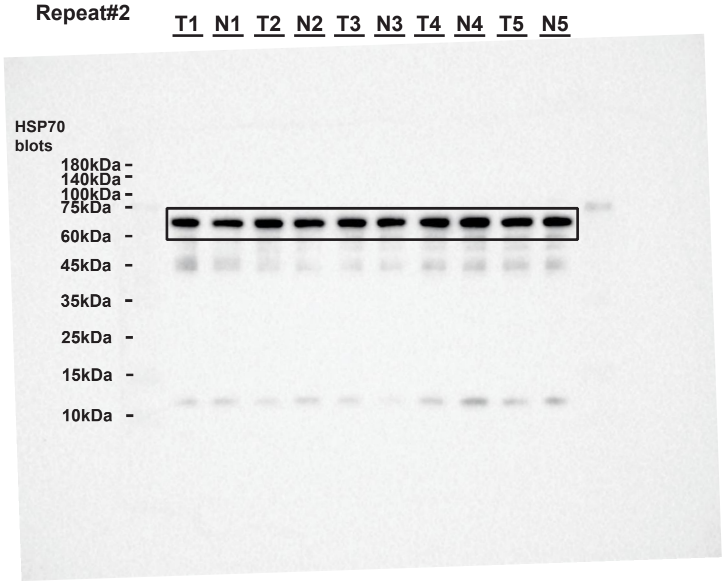


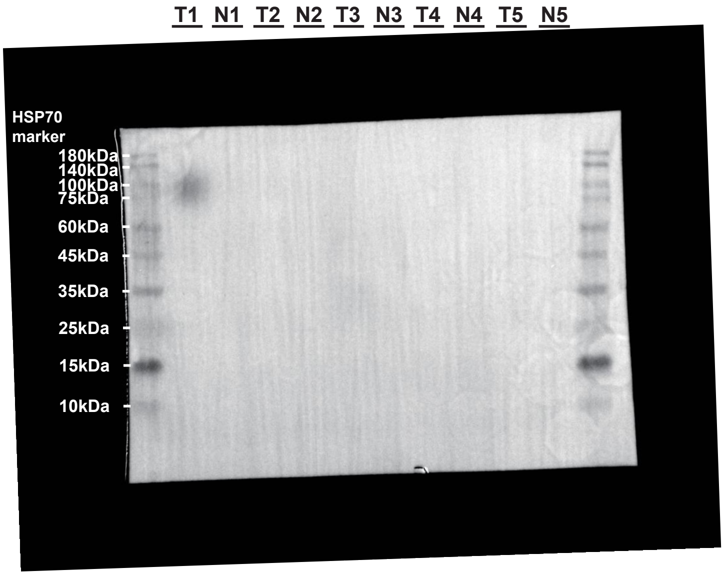


Repeat#3

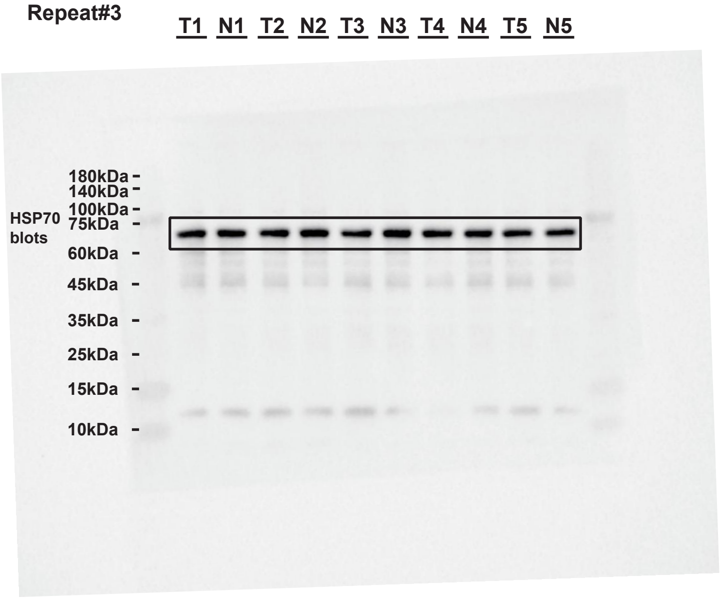


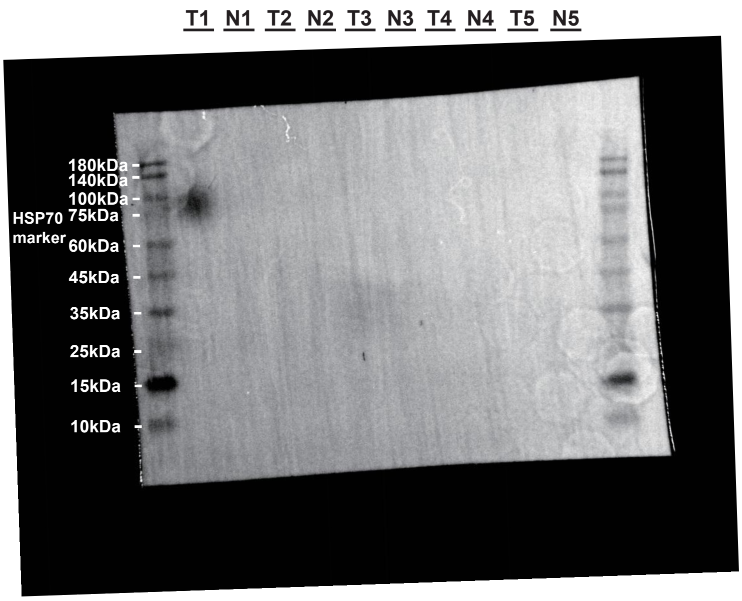

Repeat#1

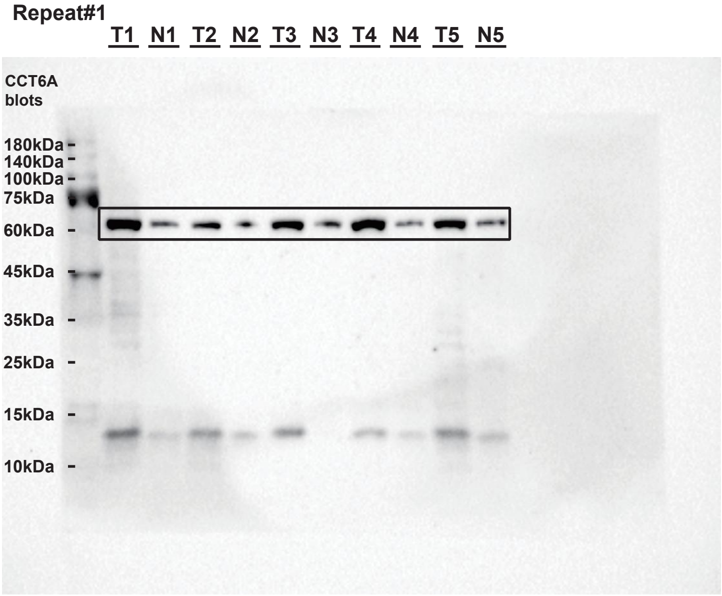


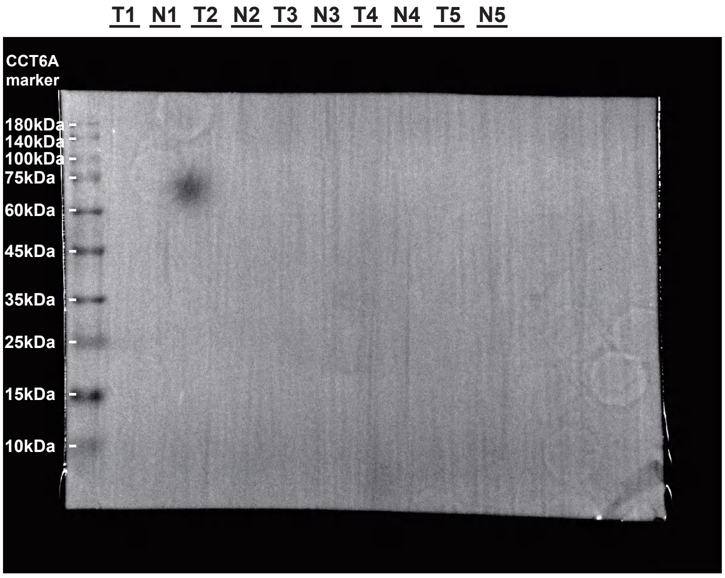


Repeat#2

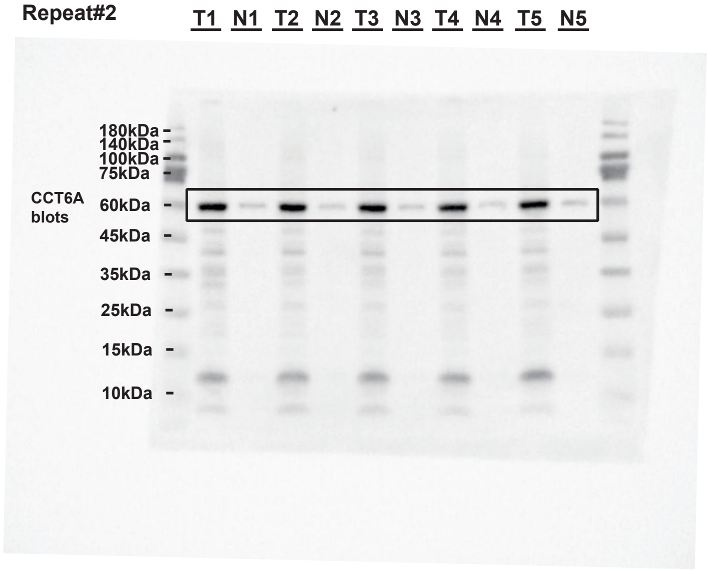


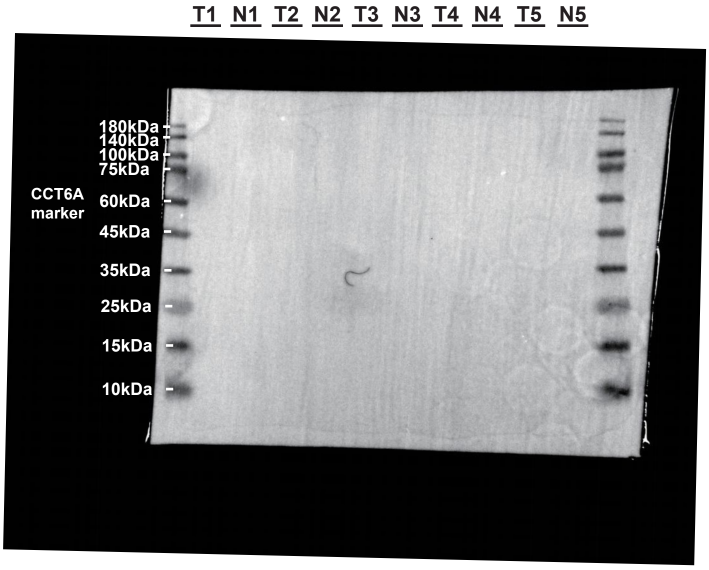


Repeat#3

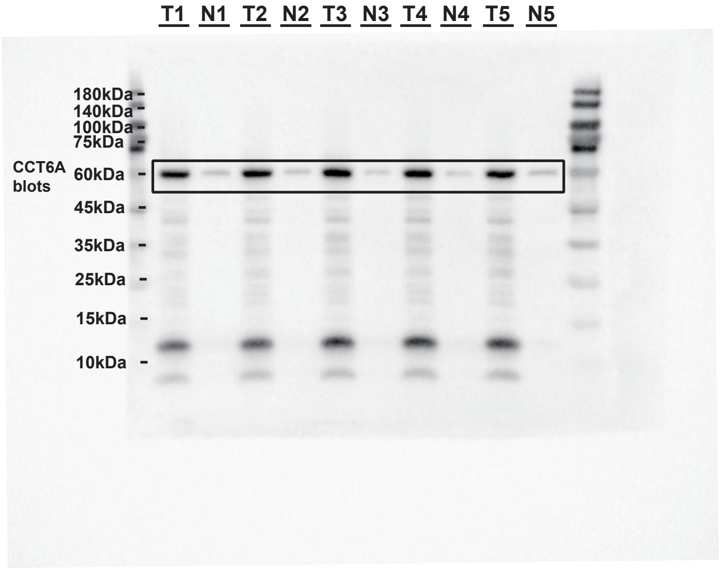


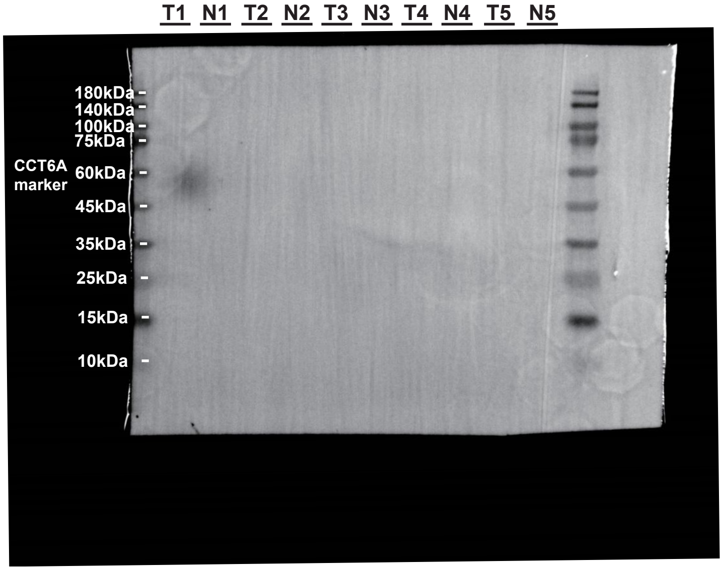

**Fig. 3A**

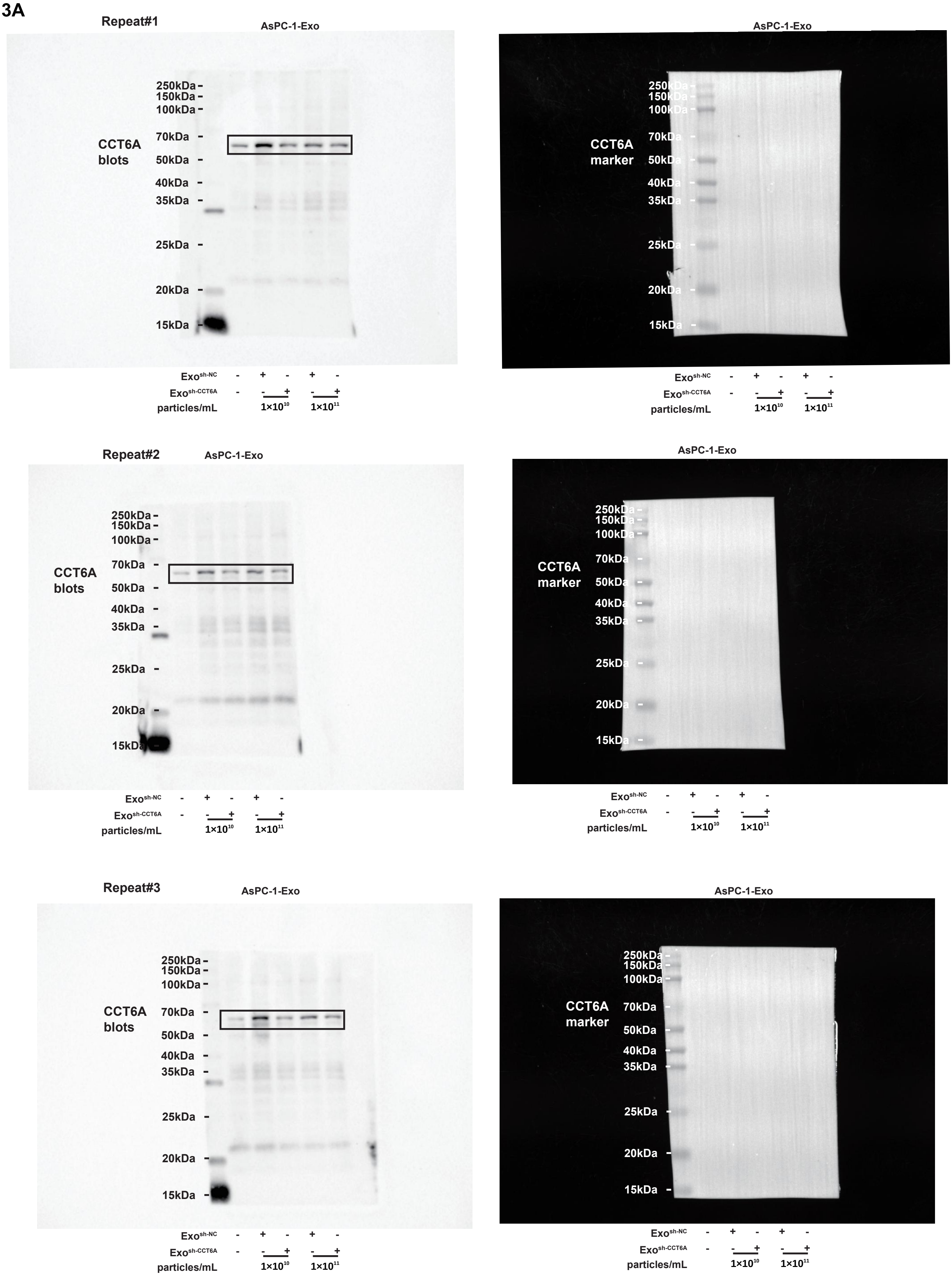


| AsPC-1 | CCT6A protein expression | | |
|---|---|---|---|
| Saline | 166148.6667 | 155616.33 | 163362.6667 |
| 1.00E+10 | 996943.6667 | 954022.67 | 979621 |
| 100000000( | 242341.3333 | 328361 | 340669.6667 |
| 1.0E+11 | 571651.6667 | 567157 | 665714 |
| 1.0E+11 | 261106 | 408853.33 | 352774.3333 |

Repeat#1

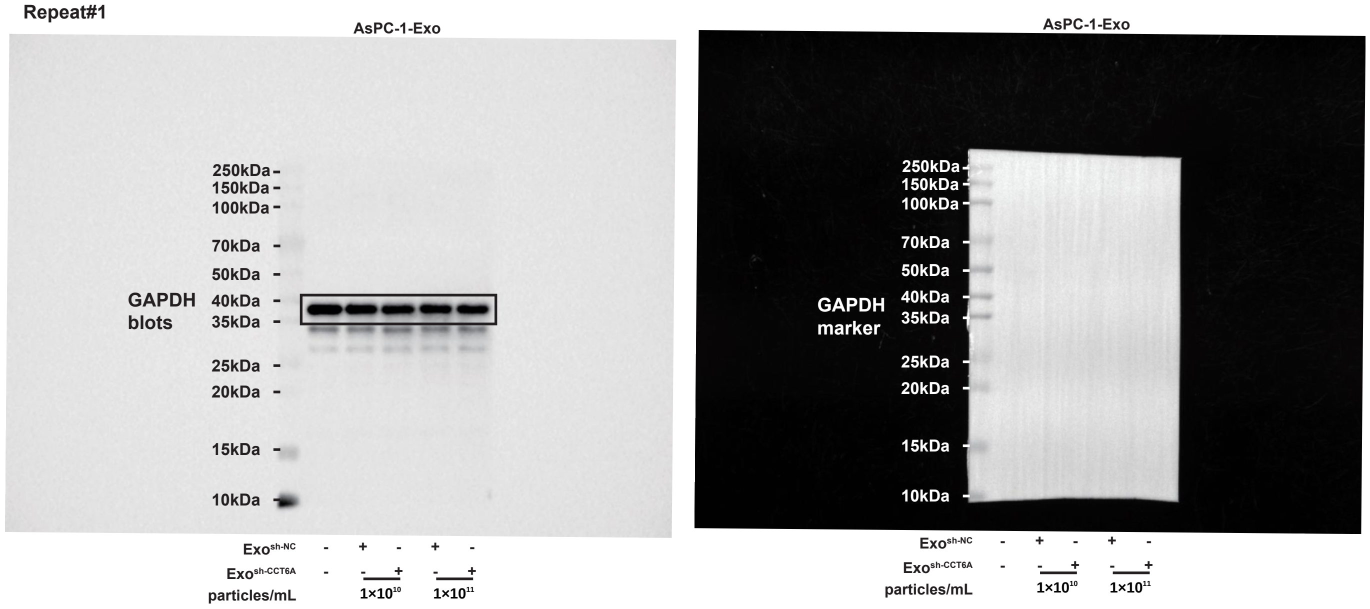


Repeat#2

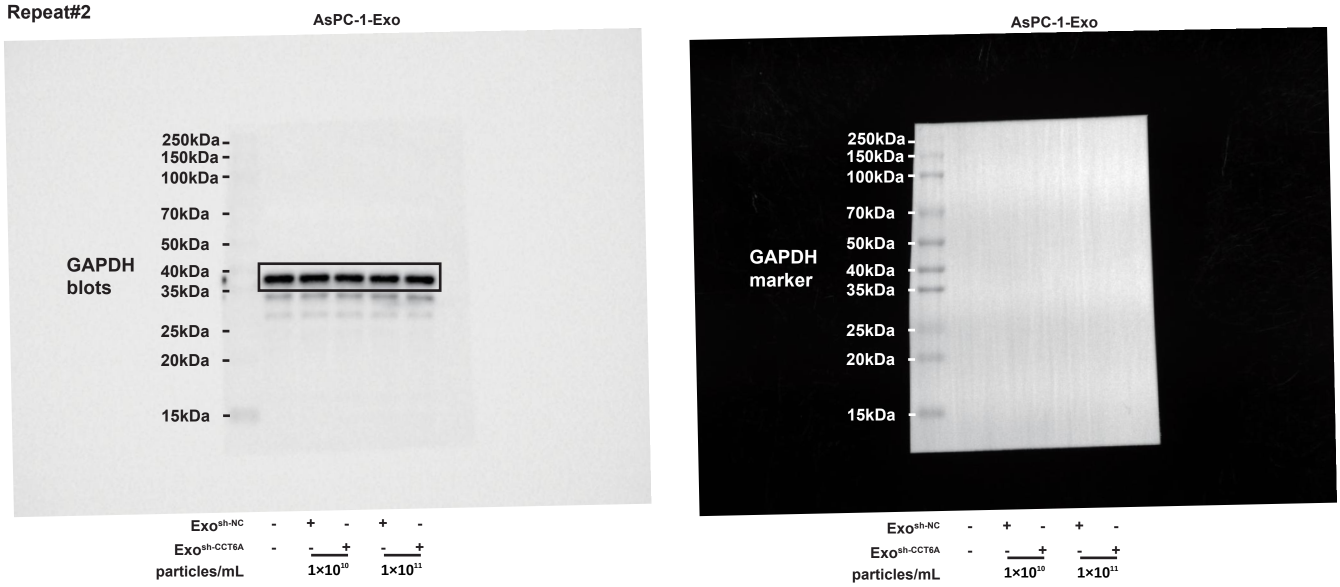


Repeat#3

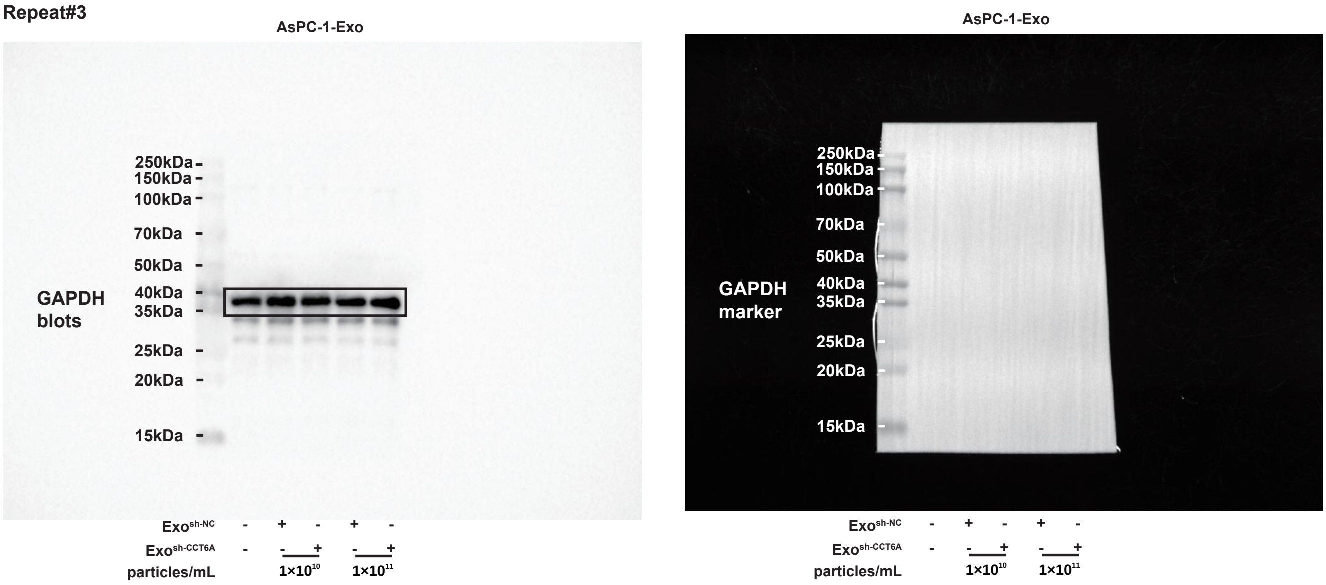


| | GAPDH protein expression | | |
|---|---|---|---|
| Saline | 3,107,912.86 | 2,416,774.71 | 2,792,372.57 |
| 1.00E+10 | 2,839,619.86 | 2,387,856.29 | 2,863,465.86 |
| 100000000( | 3,018,750.86 | 3,102,664.43 | 3,216,272.14 |
| 1.0E+11 | 3,231,420.43 | 2,524,171.71 | 3,385,637.43 |
| 1.0E+11 | 3,342,520.14 | 3,222,506.00 | 2,822,179.29 |

Repeat#1

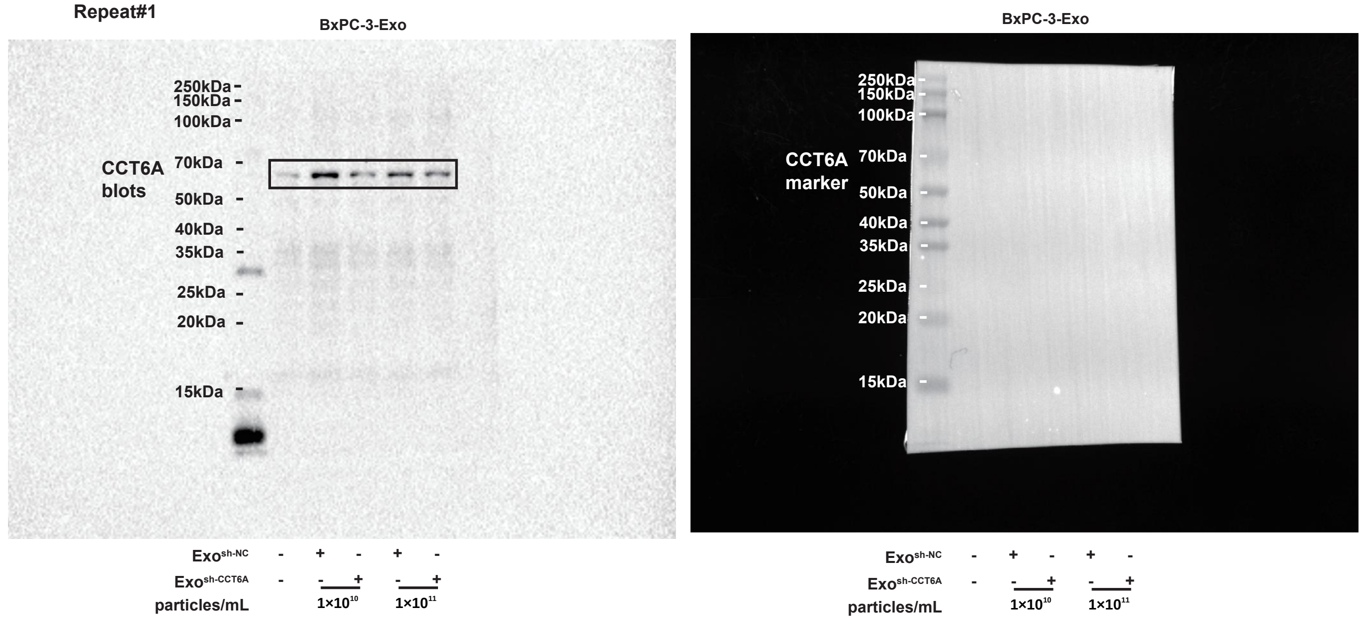


Repeat#2

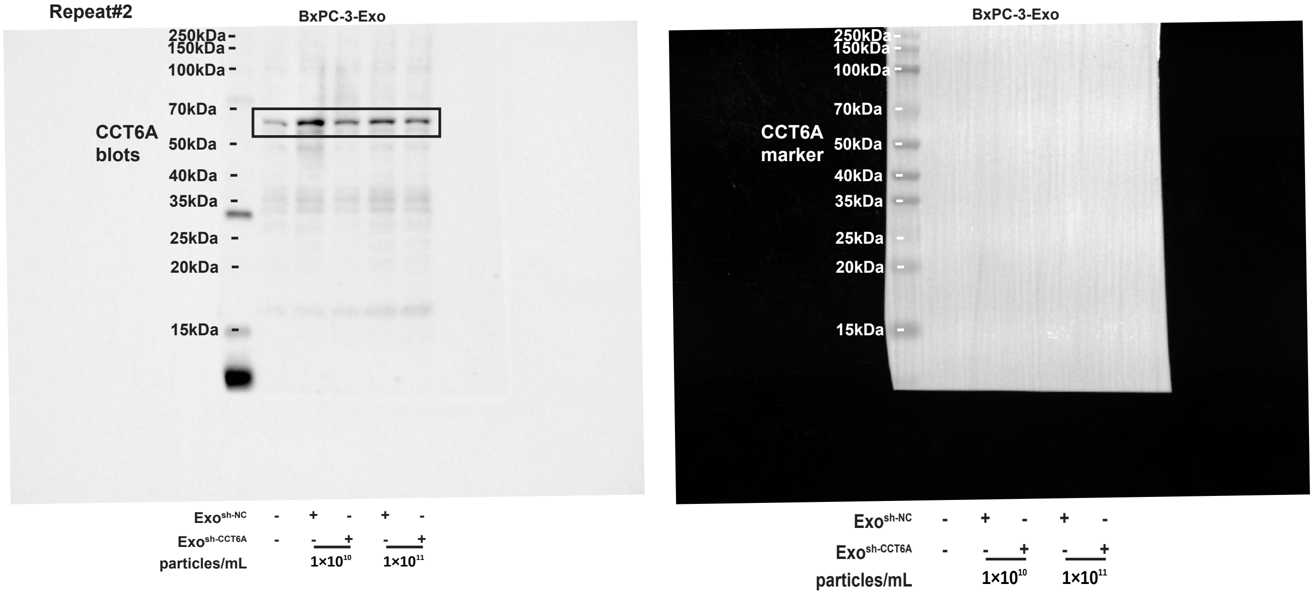


Repeat#3

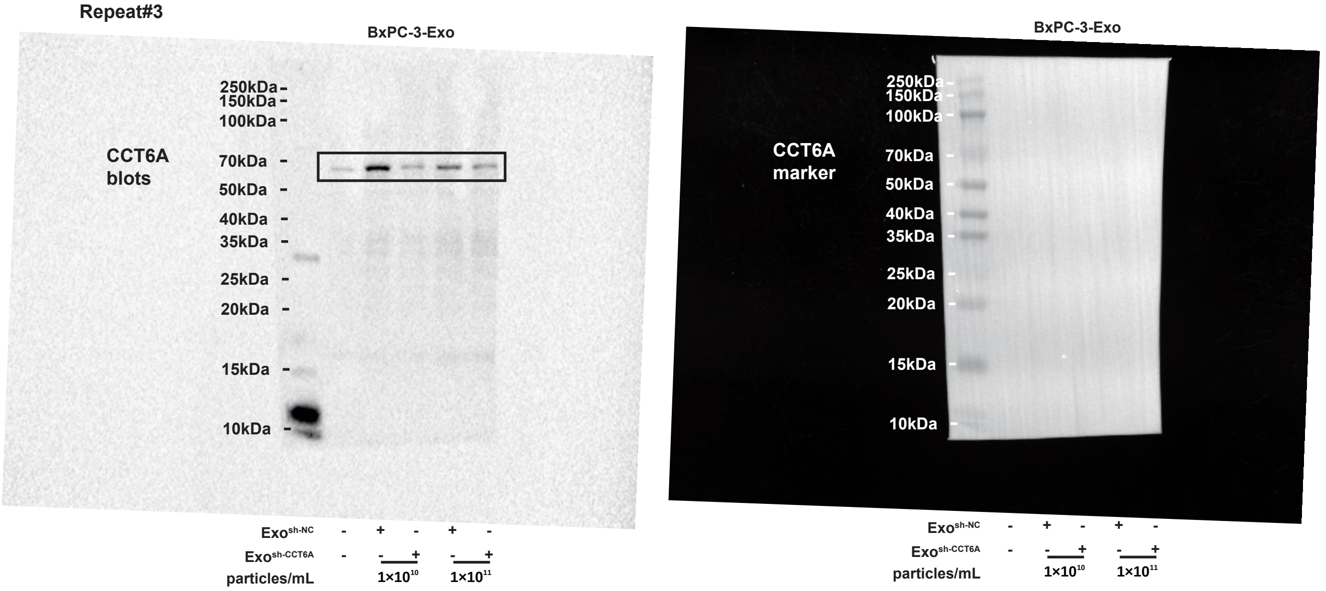


| BxPC-3 | CCT6A protein expression | | |
|---|---|---|---|
| Saline | 165488.3097 | 174126.83 | 175605.4924 |
| 1.00E+10 | 981110.5937 | 957450.51 | 950152.1413 |
| 1000000000 | 255344.9483 | 267254.23 | 227589.6313 |
| 1.0E+11 | 530772.0287 | 565090.66 | 614015.9532 |
| 1.0E+11 | 277272.3292 | 265808.99 | 263282.6269 |

Repeat#1

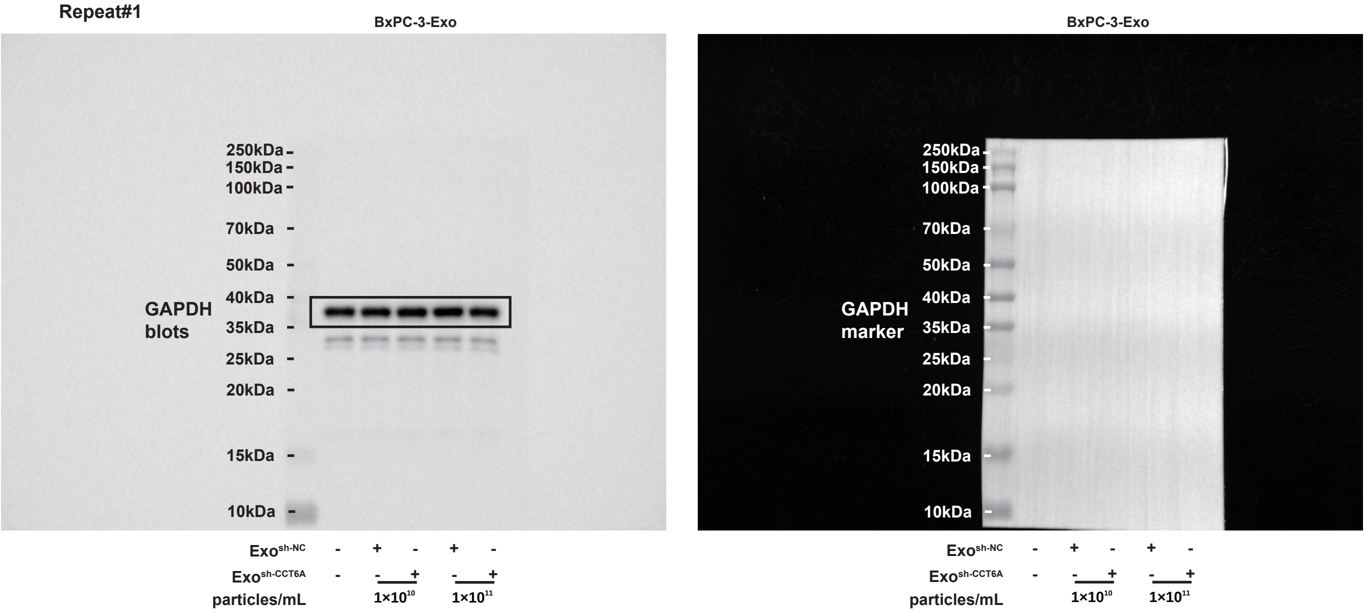


Repeat#2

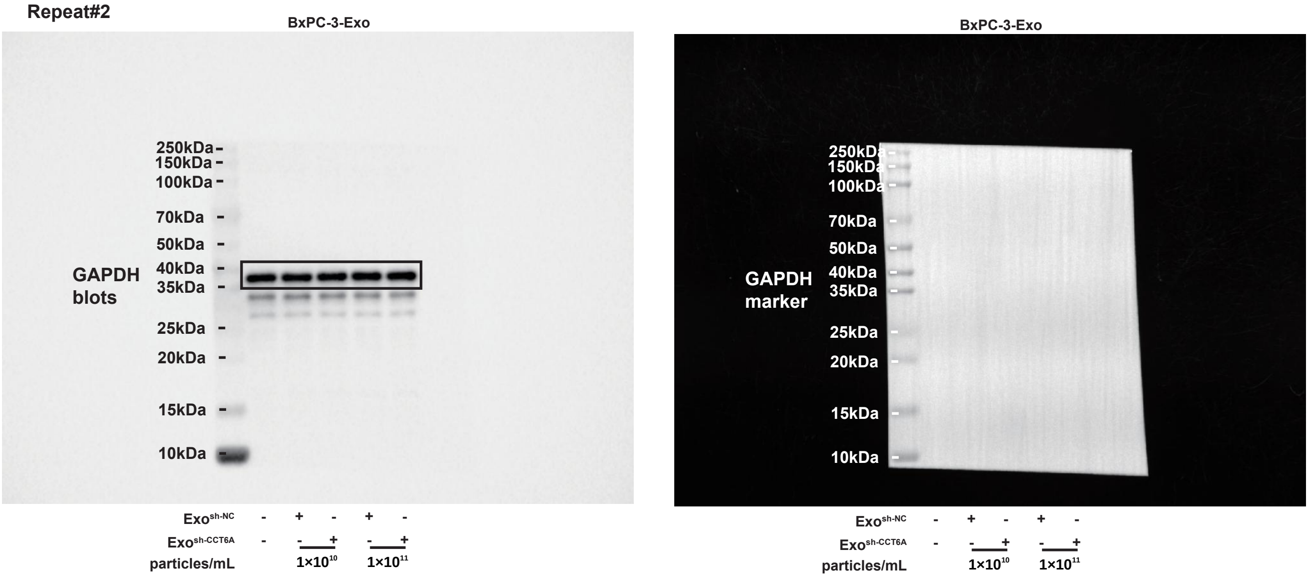


Repeat#3

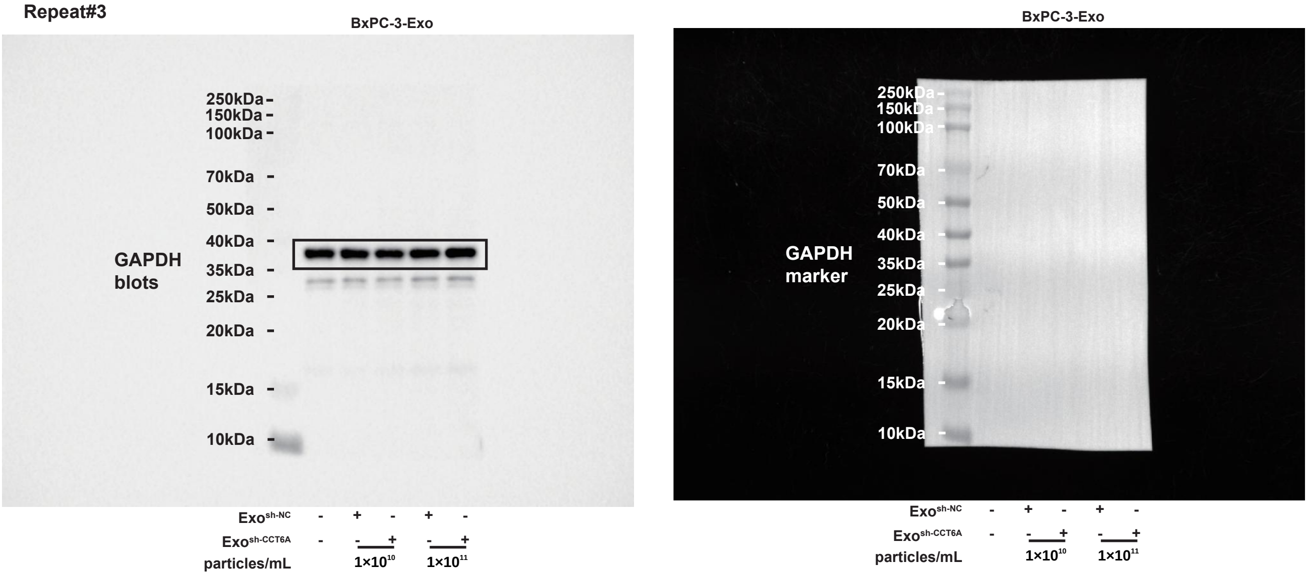


| | GAPDH protein expression | | |
|---|---|---|---|
| Saline | 2,936,677.57 | 2,928,024.32 | 2,853,032.91 |
| 1.00E+10 | 3,028,045.16 | 3,129,755.20 | 3,020,119.80 |
| 1000000000( | 3,126,335.23 | 3,102,664.43 | 3,116,550.42 |
| 1.0E+11 | 3,014,672.20 | 2,962,224.10 | 3,328,199.43 |
| 1.0E+11 | 2,927,111.73 | 3,059,871.06 | 2,917,707.61 |

Fig. 4B

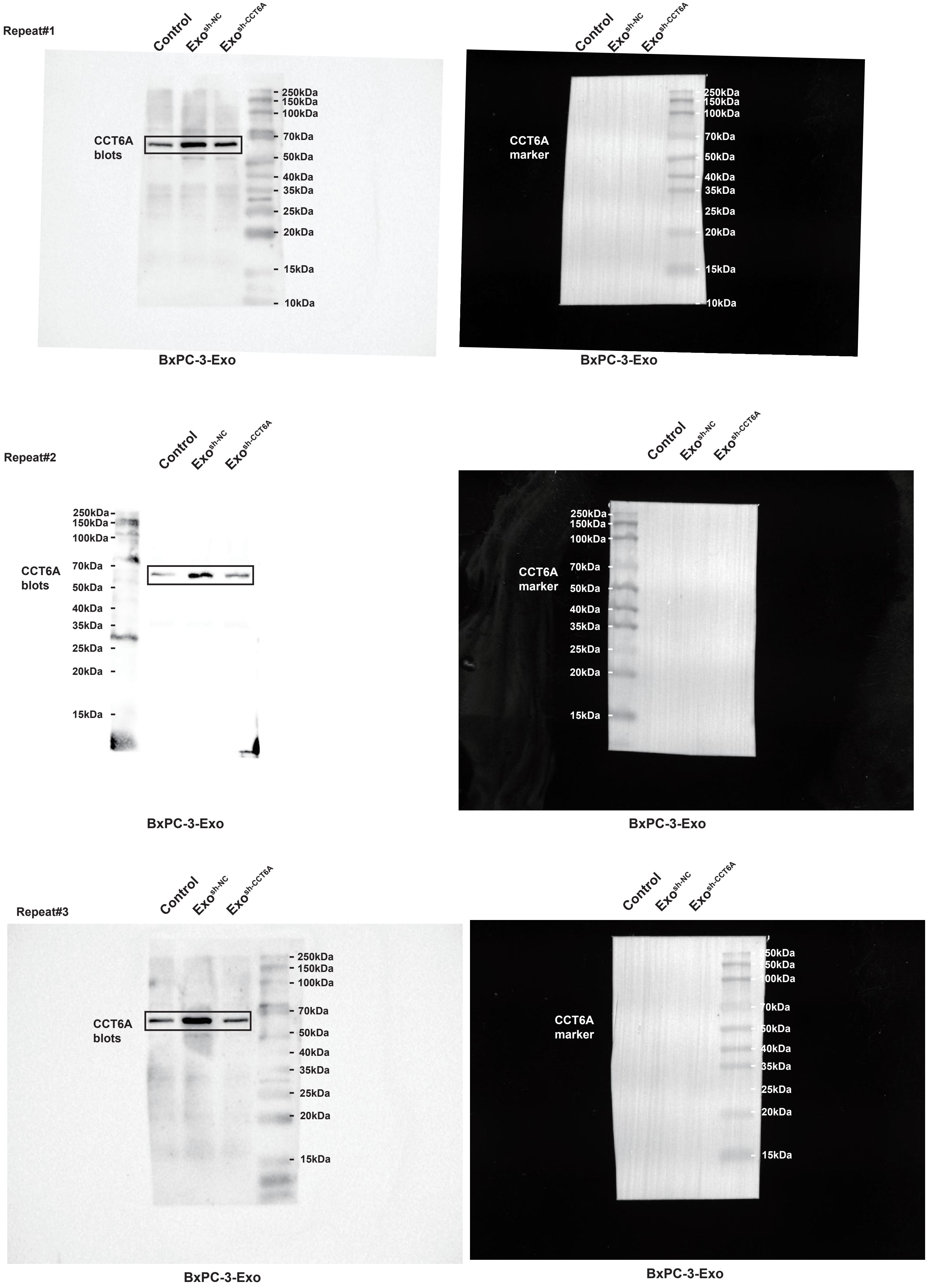
Repeat#1
Control
Exo sh-NC
Exo sh-CCT6A
CCT6A blots
250kDa
150kDa
100kDa
70kDa
50kDa
40kDa
35kDa
25kDa
20kDa
15kDa
10kDa
BxPC-3-Exo
CCT6A marker
BxPC-3-Exo
Repeat#2
CCT6A blots
BxPC-3-Exo
CCT6A marker
BxPC-3-Exo
Repeat#3
CCT6A blots
BxPC-3-Exo
CCT6A marker
BxPC-3-Exo

Repeat#1

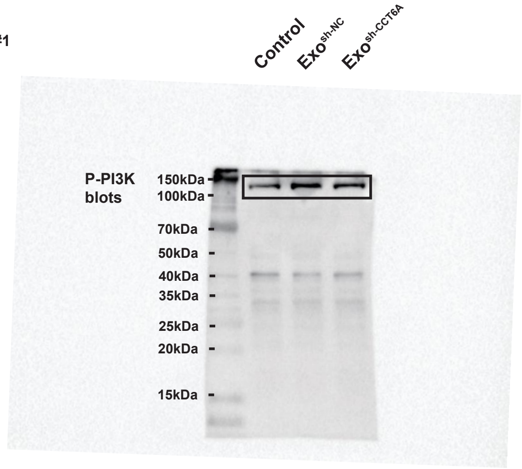


BxPC-3-Exo

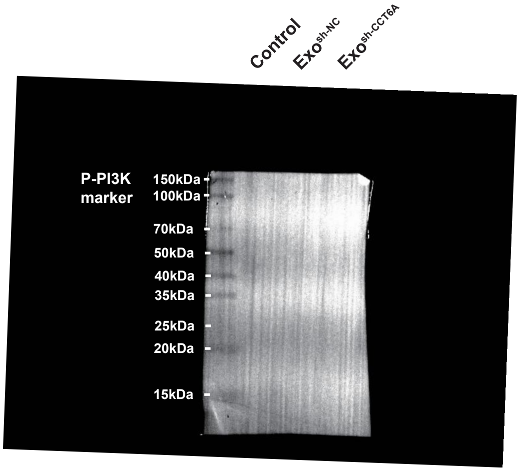


BxPC-3-Exo

Repeat#2

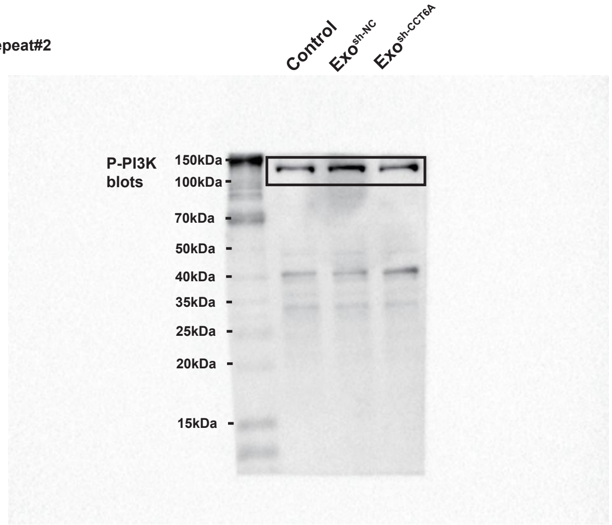


BxPC-3-Exo

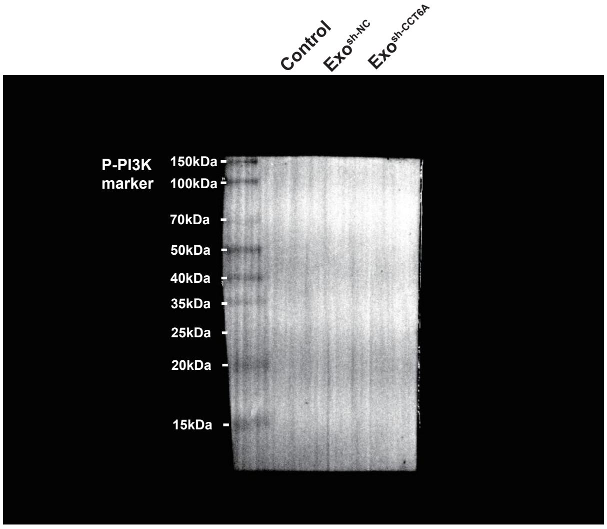


BxPC-3-Exo

Repeat#3

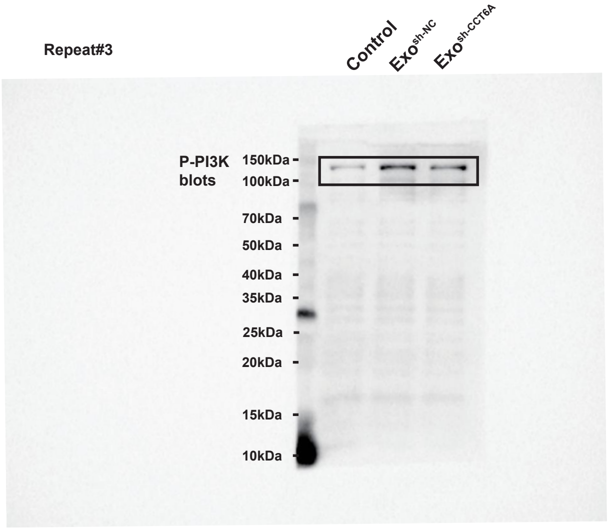


BxPC-3-Exo

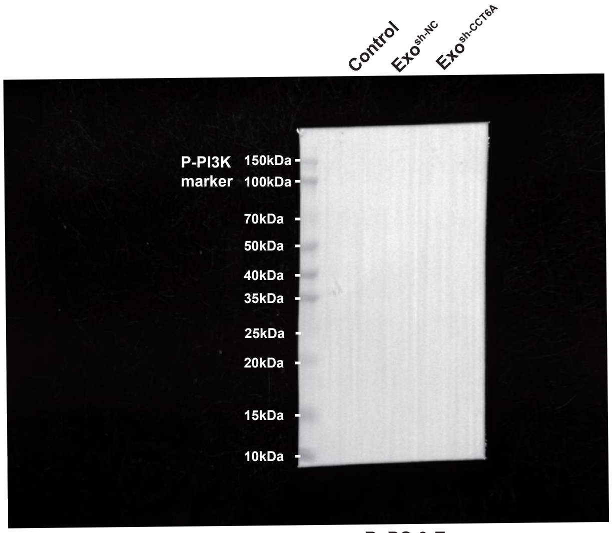


BxPC-3-Exo

| | p-PI3K | PI3K |
|---|---|---|
| Saline | 1992047 | 5780428 |
| sh-nc | 3400000.5 | 5431959 |
| sh-cct6a | 1749874 | 5518310 |
| | | |
| Saline | 1367382.5 | 3942274.667 |
| sh-nc | 2346750.5 | 3325636 |
| sh-cct6a | 1469920.5 | 3994698 |
| | | |
| Saline | 740160.7353 | 2063631.412 |
| sh-nc | 1239776.456 | 1952648.353 |
| sh-cct6a | 866898.9706 | 2388018.412 |

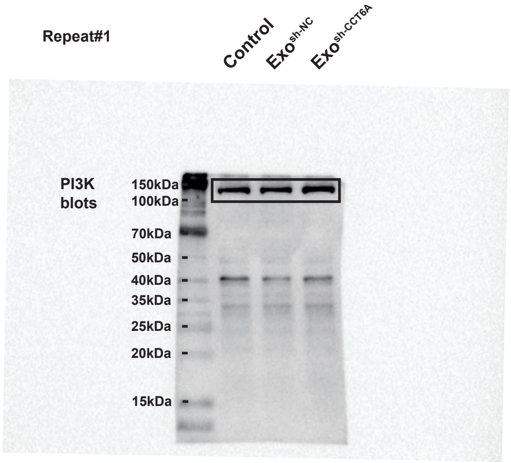

BxPC-3-Exo

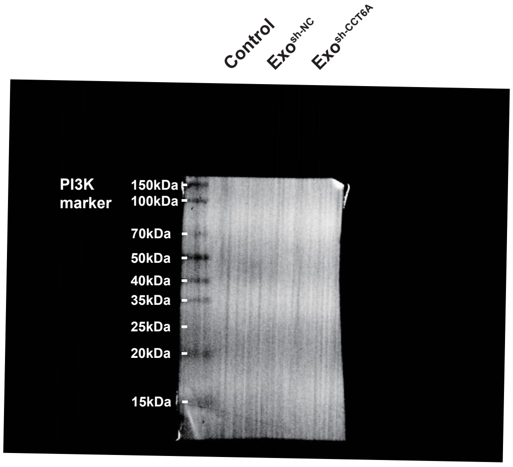

BxPC-3-Exo

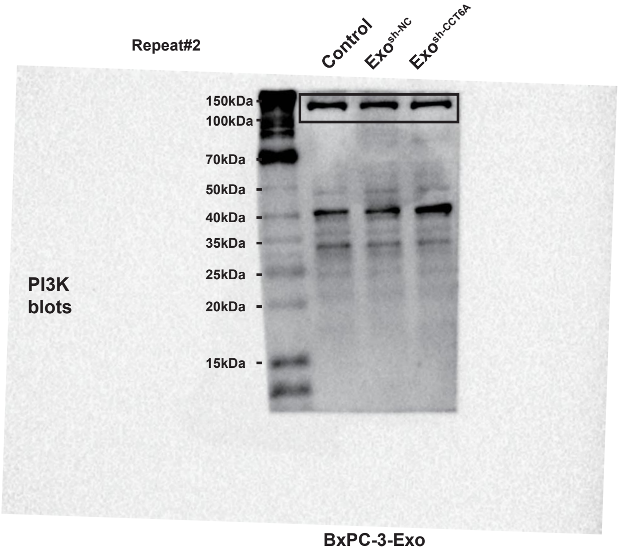

BxPC-3-Exo

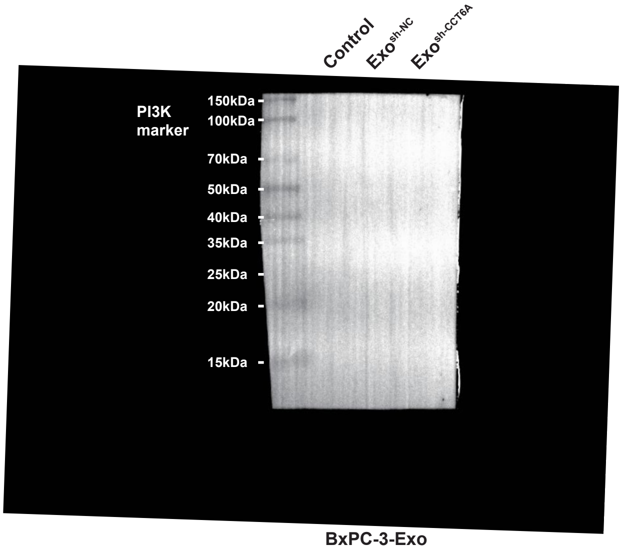

BxPC-3-Exo

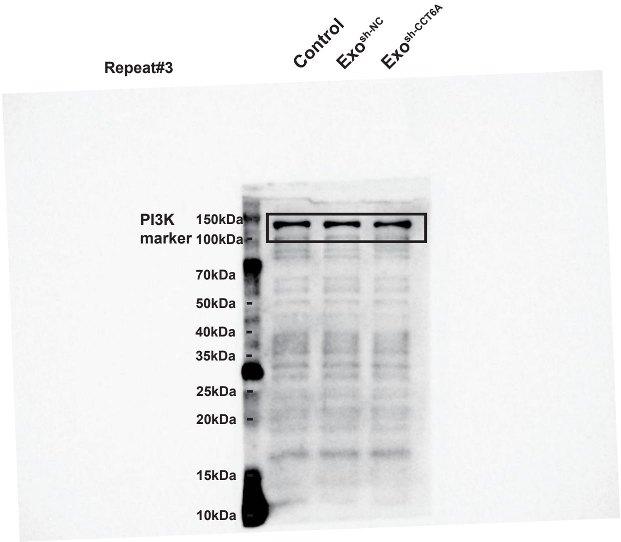

BxPC-3-Exo

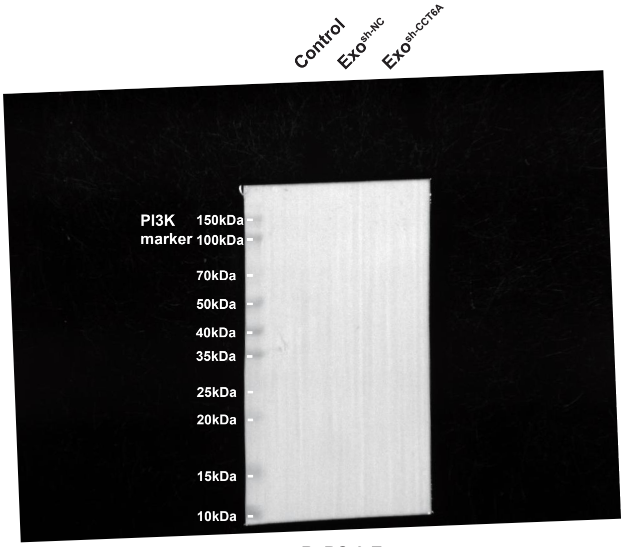

BxPC-3-Exo

| | p-PI3K | PI3K |
|---|---|---|
| Saline | 1992047 | 5780428 |
| sh-nc | 3400000.5 | 5431959 |
| sh-cct6a | 1749874 | 5518310 |
| | | |
| Saline | 1367382.5 | 3942274.667 |
| sh-nc | 2346750.5 | 3325636 |
| sh-cct6a | 1469920.5 | 3994698 |
| | | |
| Saline | 740160.7353 | 2063631.412 |
| sh-nc | 1239776.456 | 1952648.353 |
| sh-cct6a | 866898.9706 | 2388018.412 |

Repeat#1

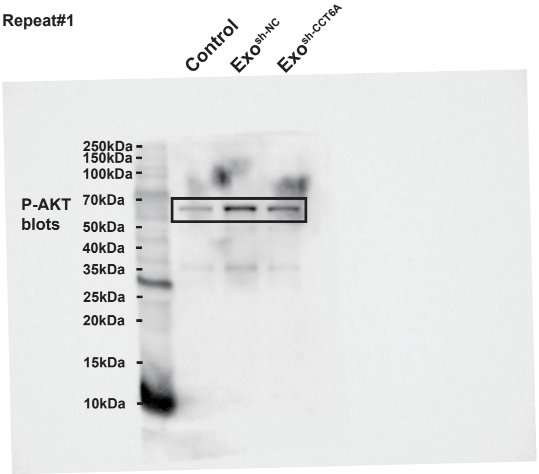


BxPC-3-Exo

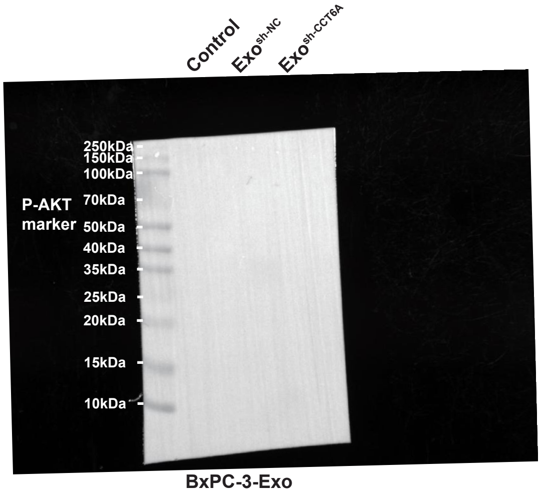


BxPC-3-Exo

Repeat#2

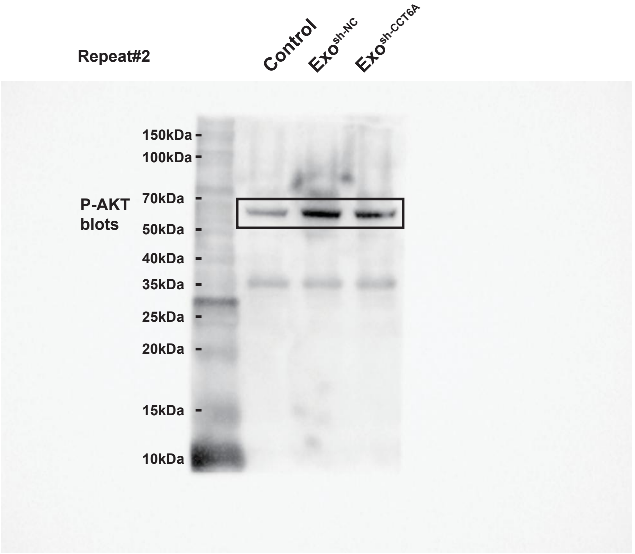


BxPC-3-Exo

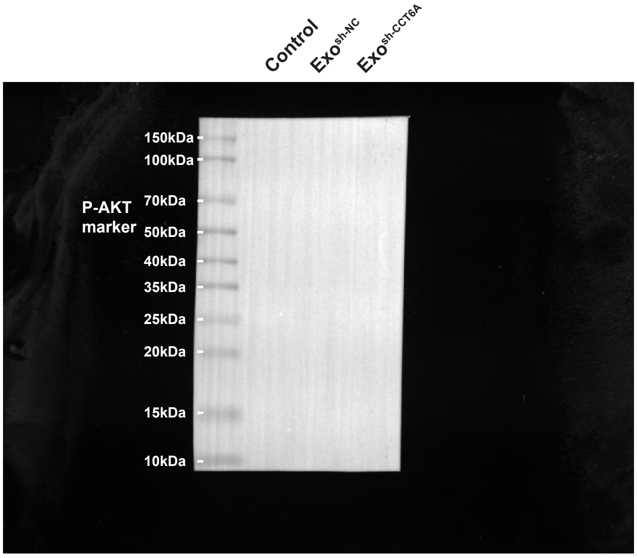


BxPC-3-Exo

Repeat#3

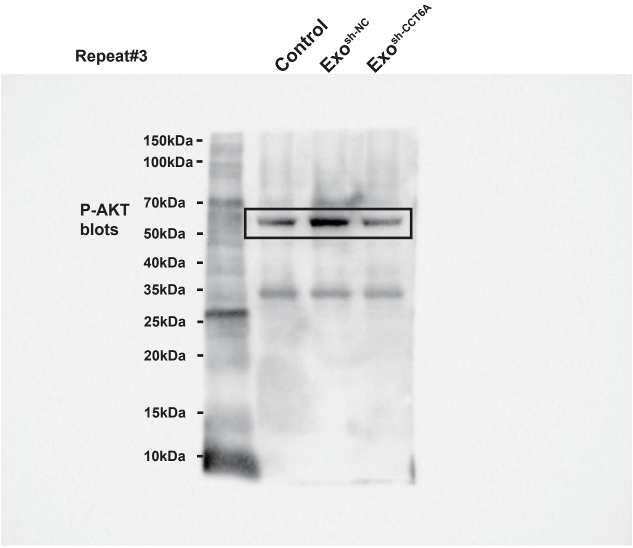


BxPC-3-Exo

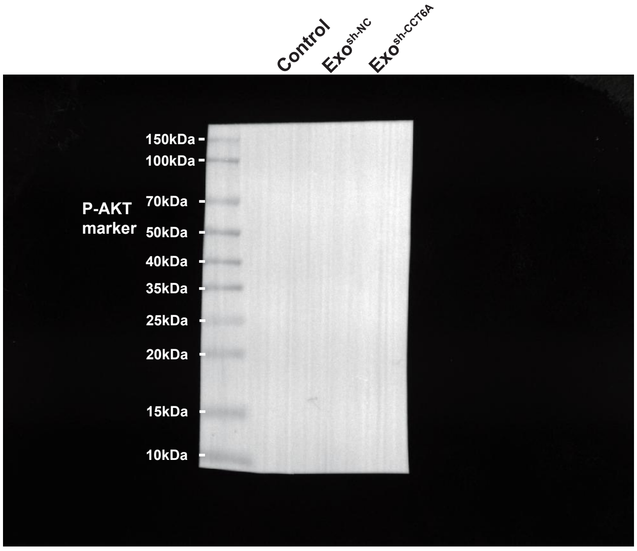


BxPC-3-Exo

| | p-AKT | AKT |
|---|---|---|
| Saline | 2114064 | 7306553.5 |
| sh-nc | 4098877.375 | 6236672.5 |
| sh-cct6a | 1738977.625 | 6724506 |
| | | |
| Saline | 1517513.769 | 6239059.846 |
| sh-nc | 4292149.231 | 5889551.077 |
| sh-cct6a | 3104070.769 | 8691948.231 |
| | | |
| Saline | 896142.7692 | 4698257.077 |
| sh-nc | 2808265.923 | 3596025.231 |
| sh-cct6a | 1739455.692 | 4070796.923 |

Repeat#1

Control $Exo^{sh-NC}$ $Exo^{sh-CCT6A}$

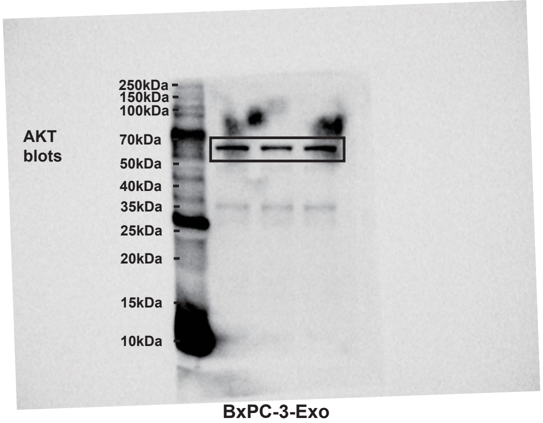


BxPC-3-Exo

Control $Exo^{sh-NC}$ $Exo^{sh-CCT6A}$

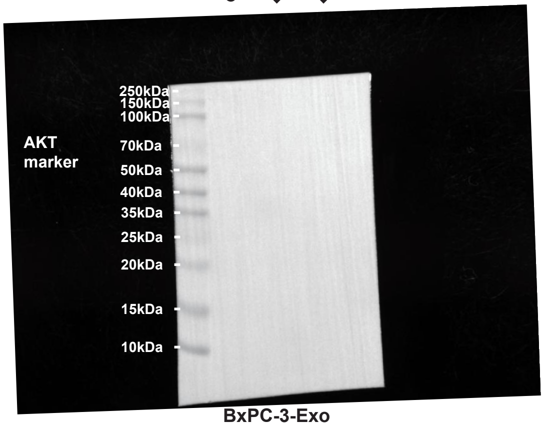


BxPC-3-Exo

Repeat#2

Control $Exo^{sh-NC}$ $Exo^{sh-CCT6A}$

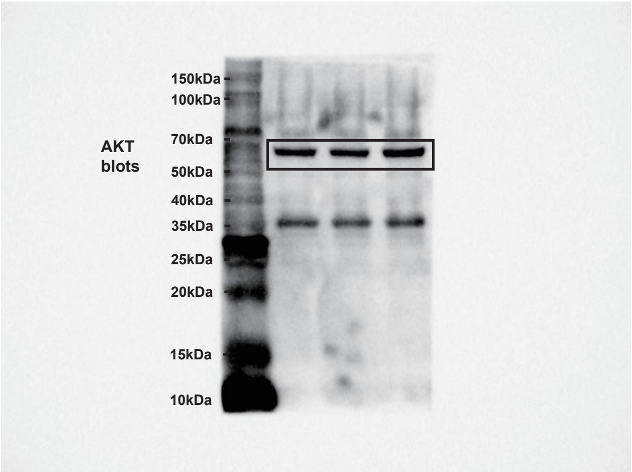


BxPC-3-Exo

Control $Exo^{sh-NC}$ $Exo^{sh-CCT6A}$

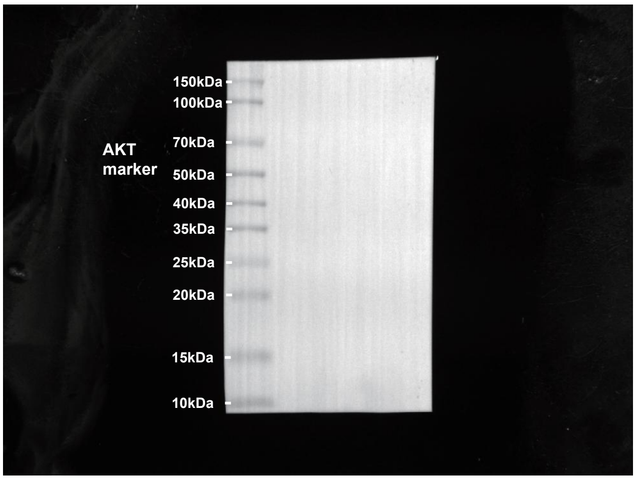


BxPC-3-Exo

Repeat#3

Control $Exo^{sh-NC}$ $Exo^{sh-CCT6A}$

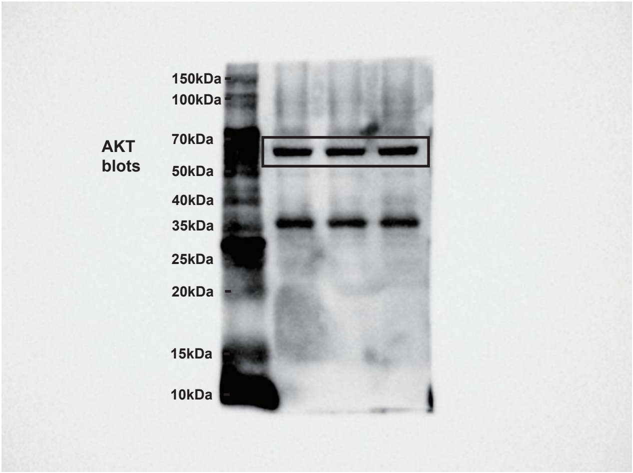


BxPC-3-Exo

Control $Exo^{sh-NC}$ $Exo^{sh-CCT6A}$

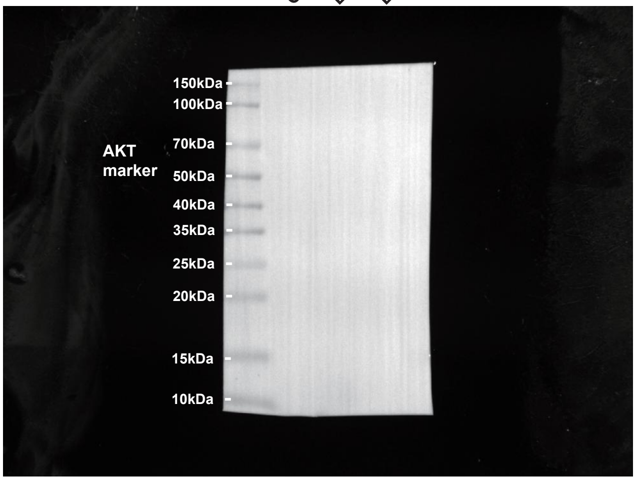


BxPC-3-Exo

| | p-AKT | AKT |
|---|---|---|
| Saline | 2114064 | 7306553.5 |
| sh-nc | 4098877.375 | 6236672.5 |
| sh-cct6a | 1738977.625 | 6724506 |
| | | |
| Saline | 1517513.769 | 6239059.846 |
| sh-nc | 4292149.231 | 5889551.077 |
| sh-cct6a | 3104070.769 | 8691948.231 |
| | | |
| Saline | 896142.7692 | 4698257.077 |
| sh-nc | 2808265.923 | 3596025.231 |
| sh-cct6a | 1739455.692 | 4070796.923 |

Repeat#1

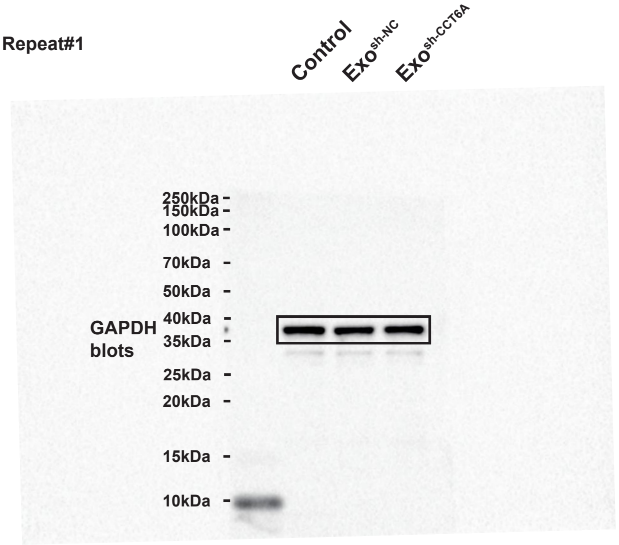


BxPC-3-Exo

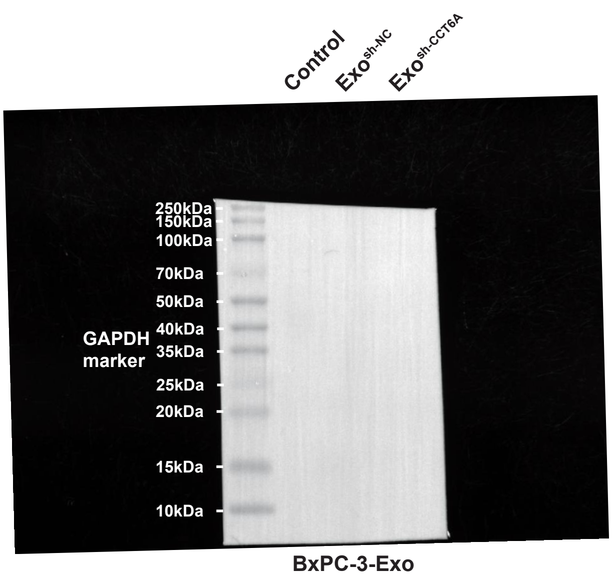


BxPC-3-Exo

Repeat#2

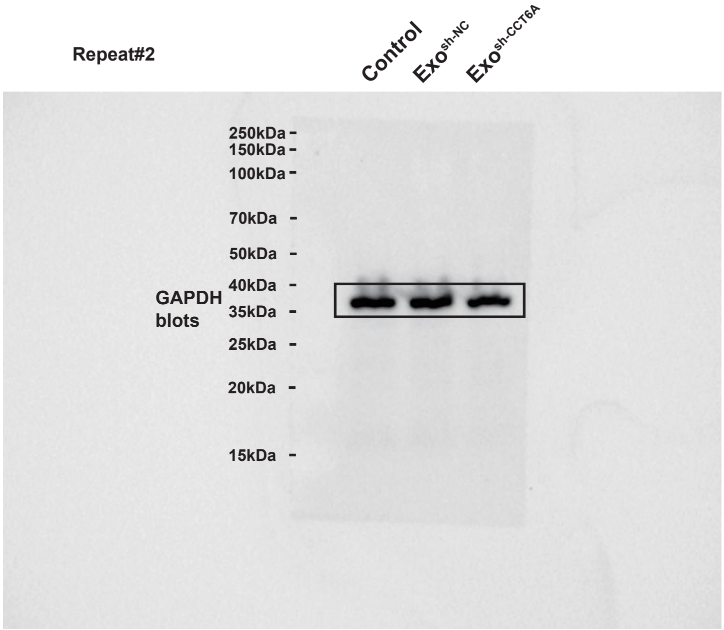


BxPC-3-Exo

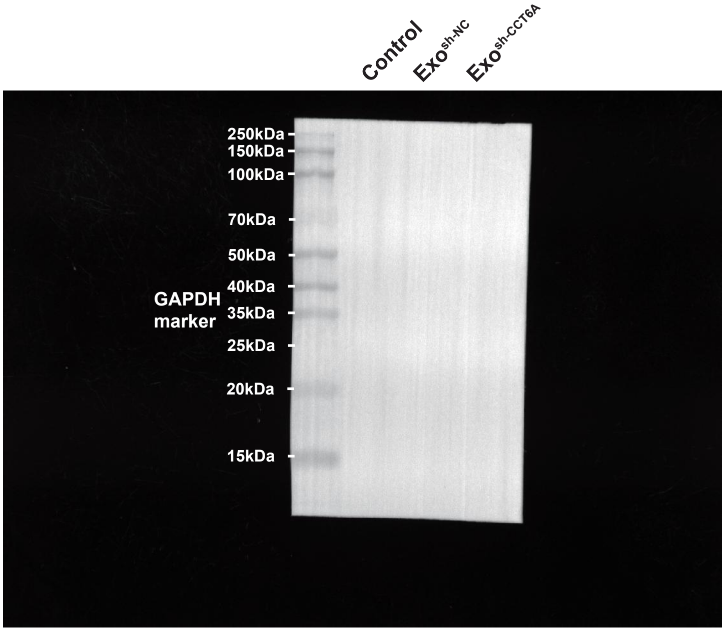


BxPC-3-Exo

Repeat#3

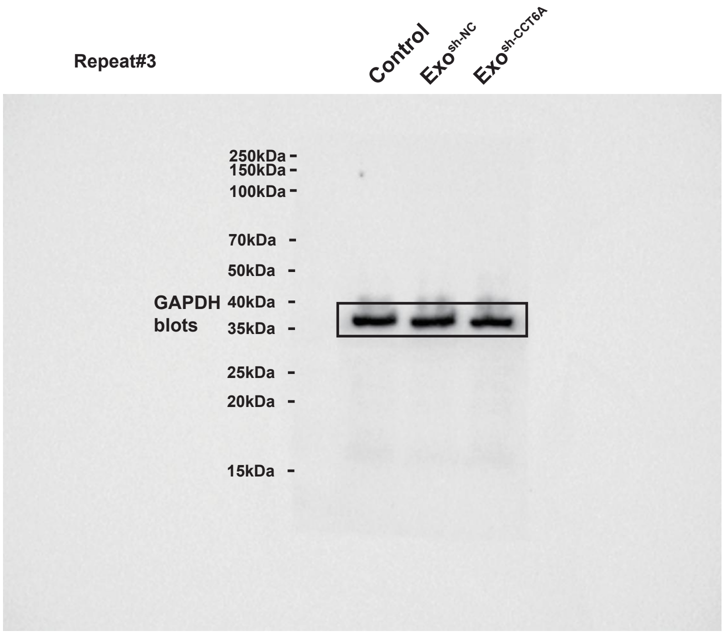


BxPC-3-Exo

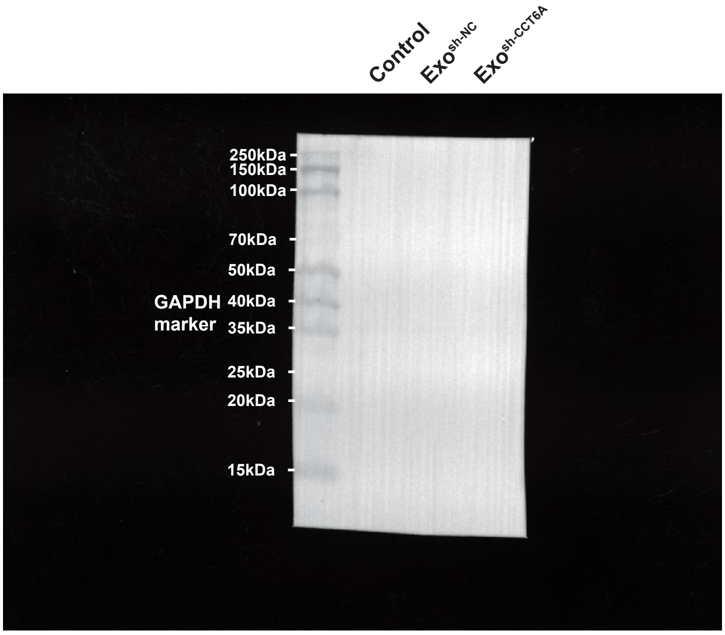


BxPC-3-Exo

**Fig. 4C**

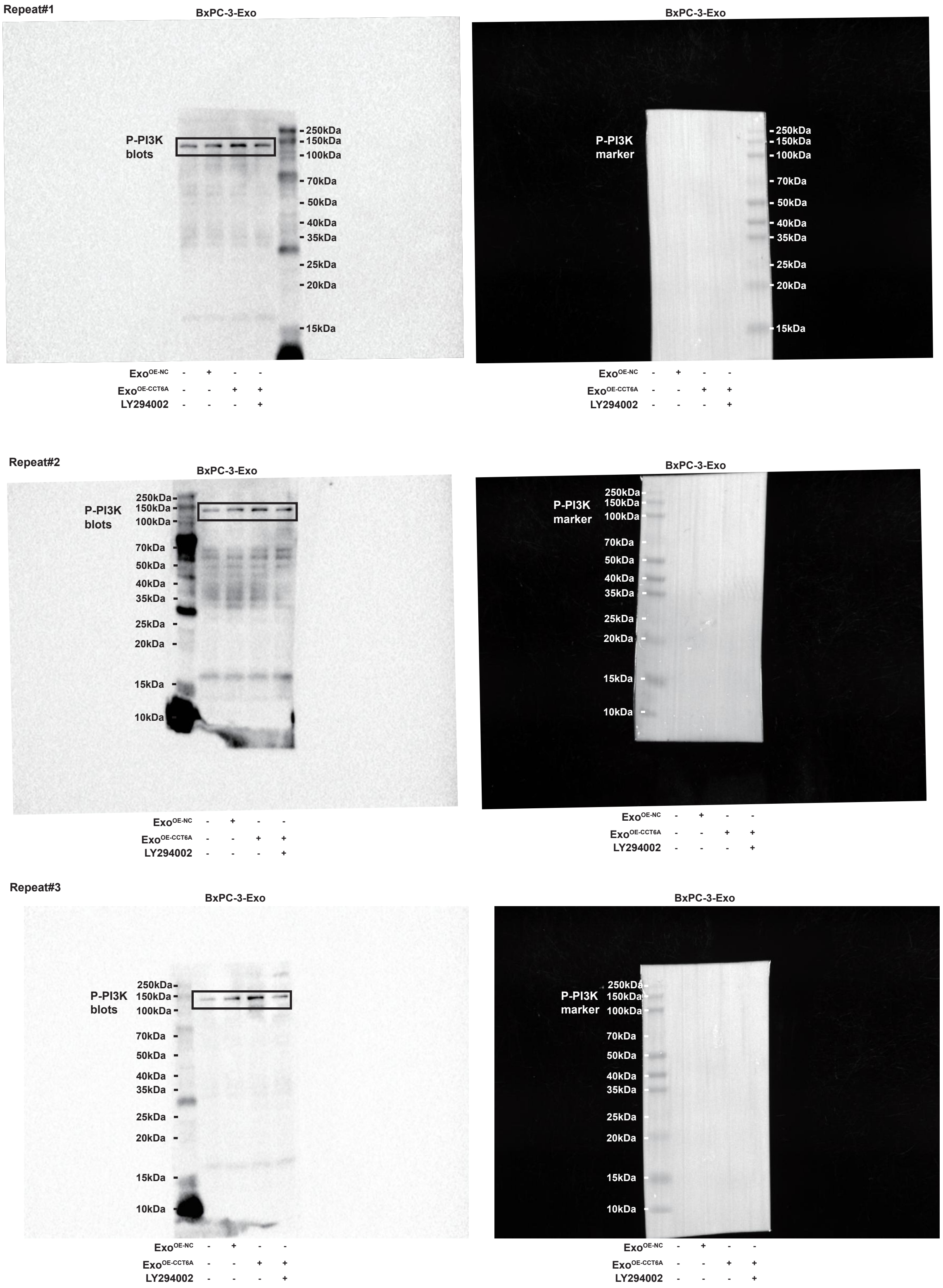


| | p-PI3K | PI3K |
|---|---|---|
| Control | 223437.91 | 693844.17 |
| Oe-nc | 328504 | 671801.04 |
| Oe-cct6a | 427045.39 | 542631.83 |
| LY294002 | 363701.74 | 679555.48 |
| | | |
| Control | 226828 | 701664 |
| Oe-nc | 330268 | 701636 |
| Oe-cct6a | 499836 | 772140 |
| LY294002 | 325980 | 656340 |
| | | |
| Control | 221593.33 | 684424 |
| Oe-nc | 348706.67 | 728152 |
| Oe-cct6a | 537661.33 | 812777.33 |
| LY294002 | 251925.33 | 505981.33 |

Repeat#1

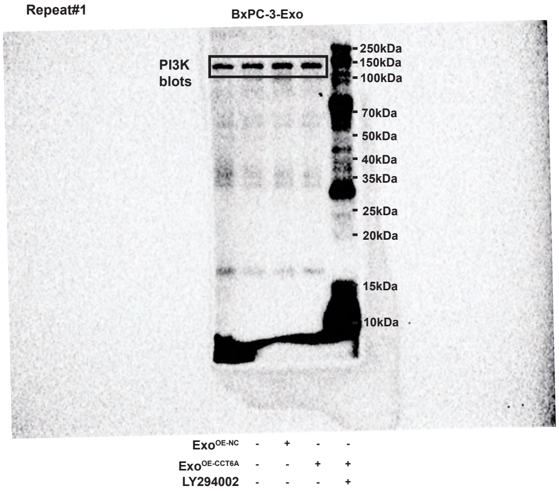


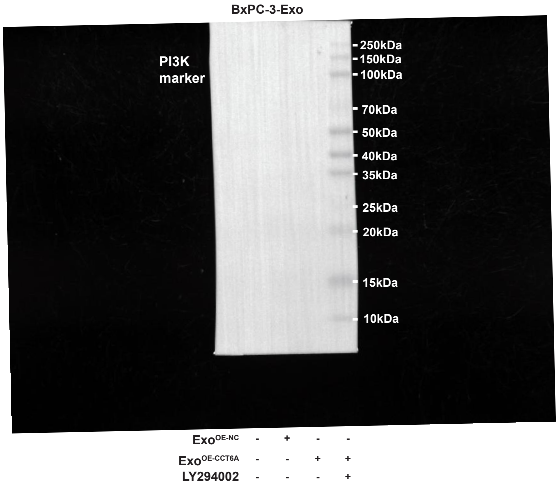


Repeat#2

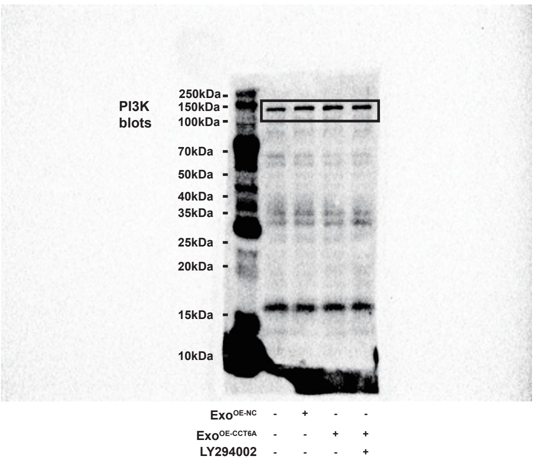


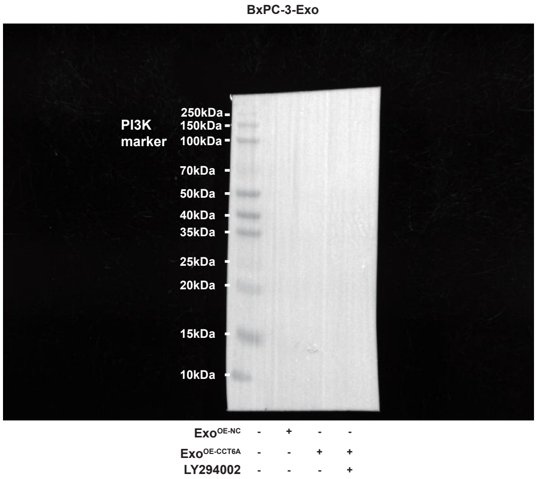


Repeat#3

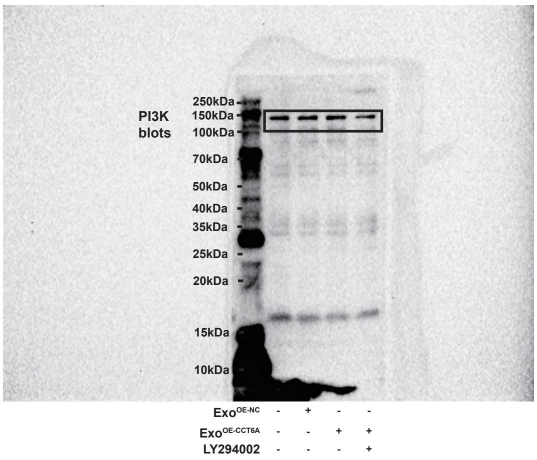


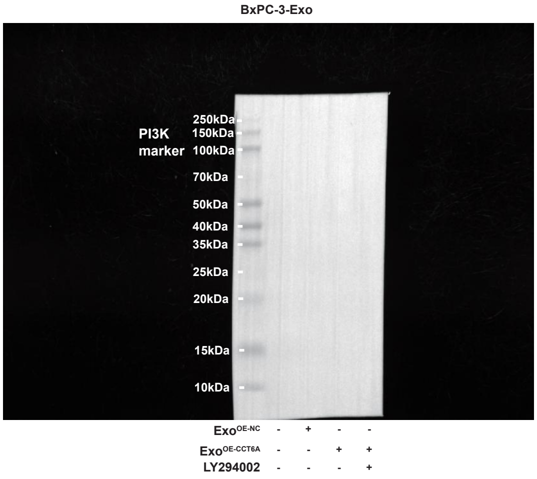


| | p-PI3K | PI3K |
|---|---|---|
| Control | 223437.91 | 693844.17 |
| Oe-nc | 328504 | 671801.04 |
| Oe-cct6a | 427045.39 | 542631.83 |
| LY294002 | 363701.74 | 679555.48 |
| | | |
| Control | 226828 | 701664 |
| Oe-nc | 330268 | 701636 |
| Oe-cct6a | 499836 | 772140 |
| LY294002 | 325980 | 656340 |
| | | |
| Control | 221593.33 | 684424 |
| Oe-nc | 348706.67 | 728152 |
| Oe-cct6a | 537661.33 | 812777.33 |
| LY294002 | 251925.33 | 505981.33 |

Repeat#1

BxPC-3-Exo

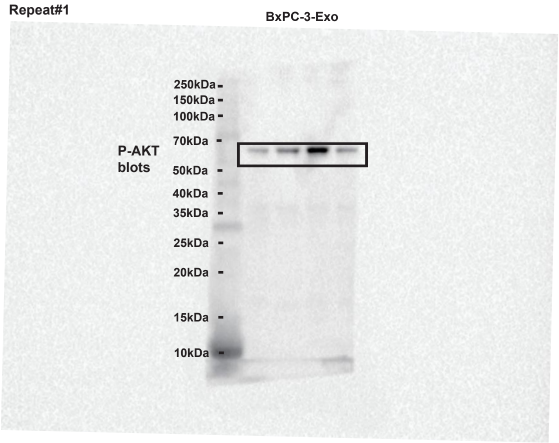


Exo^OE-NC - + - -
Exo^OE-CCT6A - - + +
LY294002 - - - +

BxPC-3-Exo

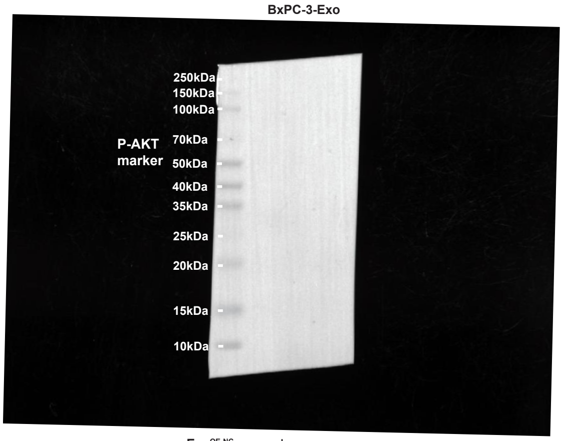


Exo^OE-NC - + - -
Exo^OE-CCT6A - - + +
LY294002 - - - +

Repeat#2

BxPC-3-Exo

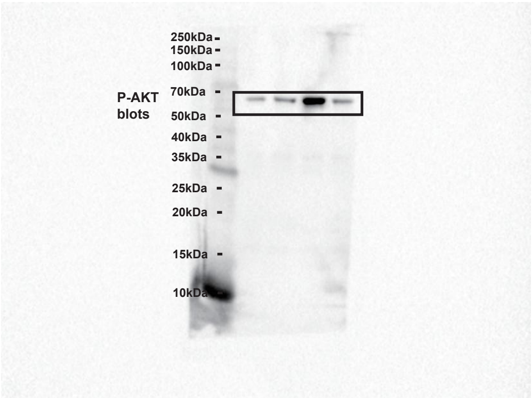


Exo^OE-NC - + - -
Exo^OE-CCT6A - - + +
LY294002 - - - +

BxPC-3-Exo

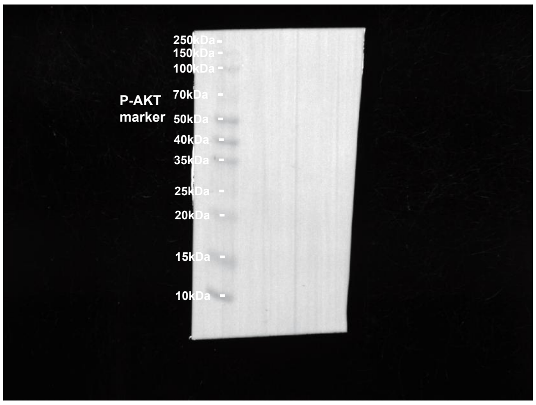


Exo^OE-NC - + - -
Exo^OE-CCT6A - - + +
LY294002 - - - +

Repeat#3

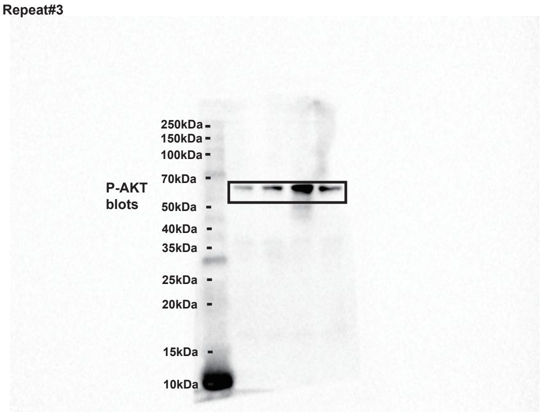


Exo^OE-NC - + - -
Exo^OE-CCT6A - - + +
LY294002 - - - +

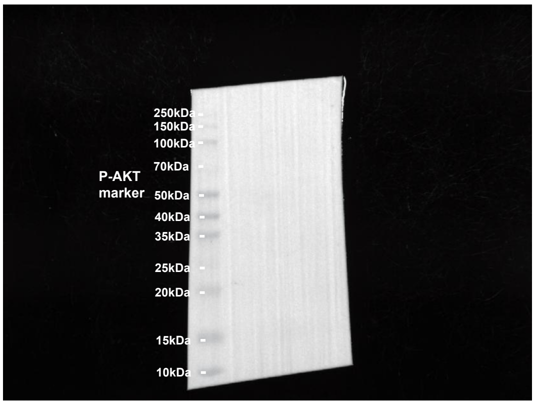


Exo^OE-NC - + - -
Exo^OE-CCT6A - - + +
LY294002 - - - +

| | p-Akt | Akt |
|---|---|---|
| Control | 300681.82 | 2321446.4 |
| Oe-nc | 820899.91 | 3271143.3 |
| Oe-cct6a | 2844738 | 3396569.2 |
| LY294002 | 459320 | 3871703.7 |
| | | |
| Control | 219860.7 | 2303900.9 |
| Oe-nc | 627368.17 | 2257721.2 |
| Oe-cct6a | 2020997 | 2166482.6 |
| LY294002 | 375329.91 | 2618177.2 |
| | | |
| Control | 347859.2 | 3324520.8 |
| Oe-nc | 793895.2 | 2906349.6 |
| Oe-cct6a | 2765901 | 2997988.8 |
| LY294002 | 919060.8 | 3120422.4 |

Repeat#1

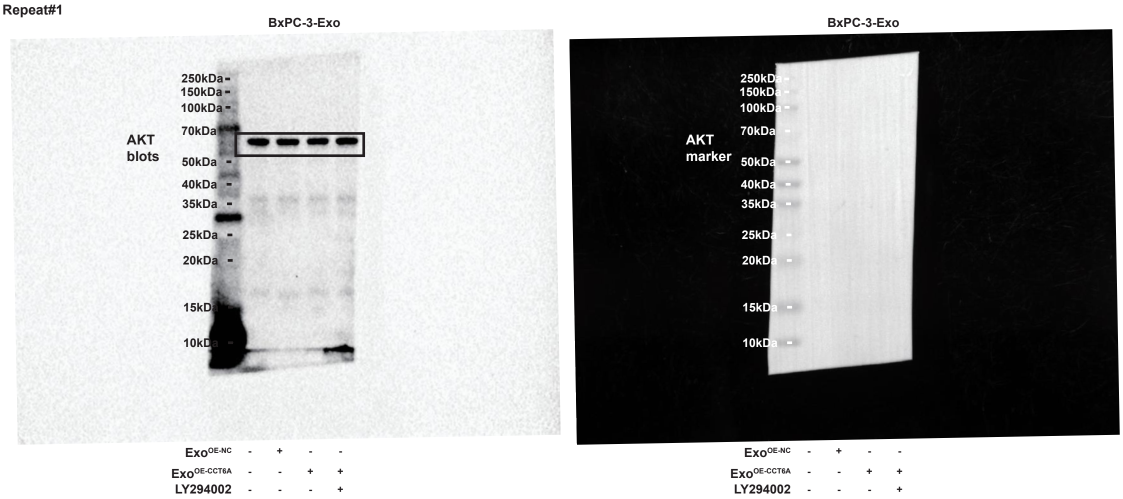


Repeat#2

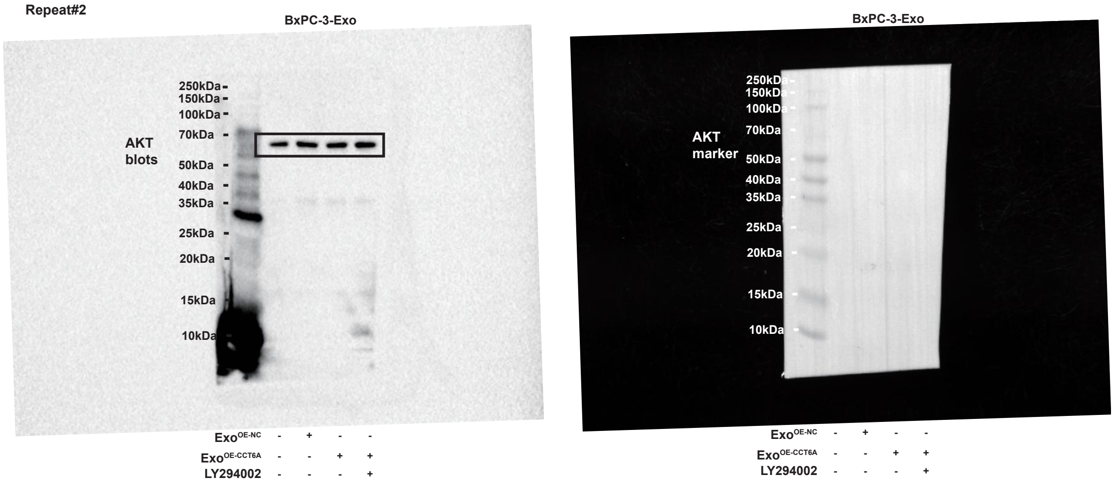


Repeat#3

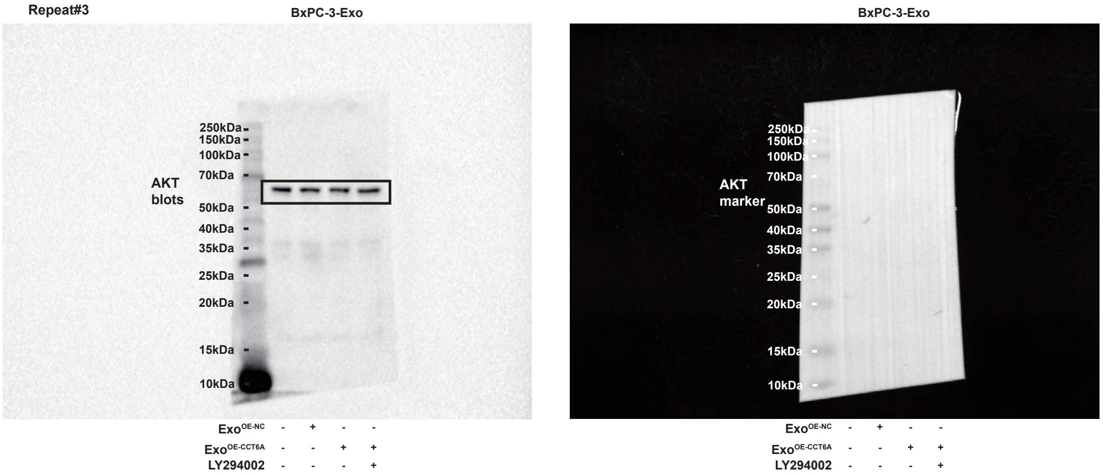


| | p-Akt | Akt |
|---|---|---|
| Control | 300681.82 | 2321446.4 |
| Oe-nc | 820899.91 | 3271143.3 |
| Oe-cct6a | 2844738 | 3396569.2 |
| LY294002 | 459320 | 3871703.7 |
| | | |
| Control | 219860.7 | 2303900.9 |
| Oe-nc | 627368.17 | 2257721.2 |
| Oe-cct6a | 2020997 | 2166482.6 |
| LY294002 | 375329.91 | 2618177.2 |
| | | |
| Control | 347859.2 | 3324520.8 |
| Oe-nc | 793895.2 | 2906349.6 |
| Oe-cct6a | 2765901 | 2997988.8 |
| LY294002 | 919060.8 | 3120422.4 |

**Repeat#1**

BxPC-3-Exo

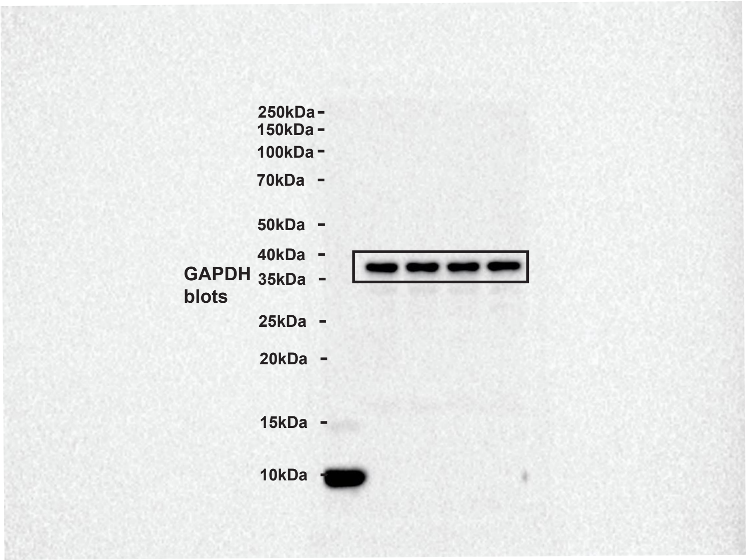


| | | | | |
|---|---|---|---|---|
| Exo$^{OE-NC}$ | - | + | - | - |
| Exo$^{OE-CCT6A}$ | - | - | + | + |
| LY294002 | - | - | - | + |

BxPC-3-Exo

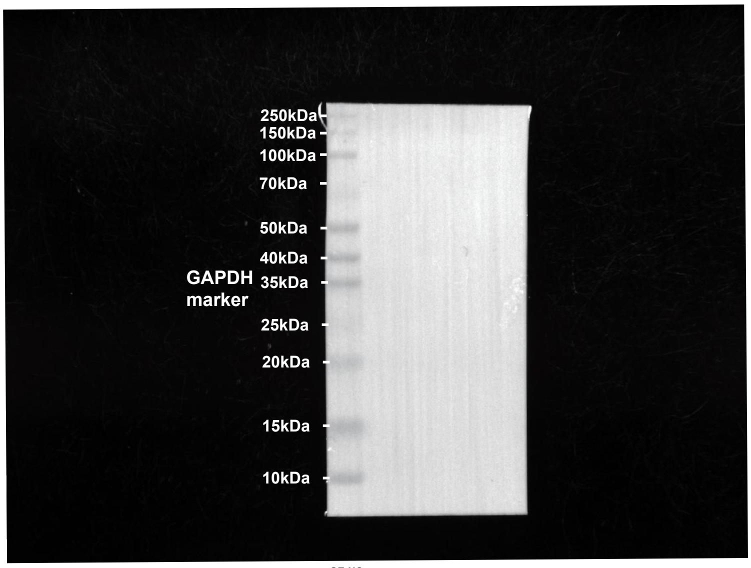


| | | | | |
|---|---|---|---|---|
| Exo$^{OE-NC}$ | - | + | - | - |
| Exo$^{OE-CCT6A}$ | - | - | + | + |
| LY294002 | - | - | - | + |

**Repeat#2**

BxPC-3-Exo

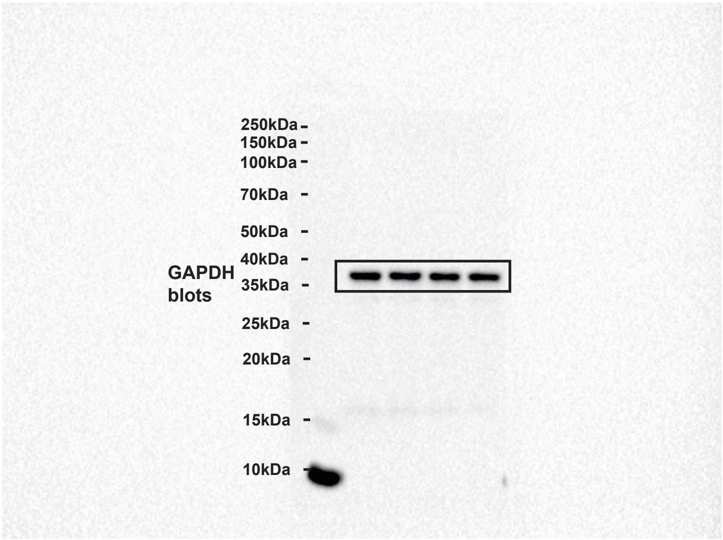


| | | | | |
|---|---|---|---|---|
| Exo$^{OE-NC}$ | - | + | - | - |
| Exo$^{OE-CCT6A}$ | - | - | + | + |
| LY294002 | - | - | - | + |

BxPC-3-Exo

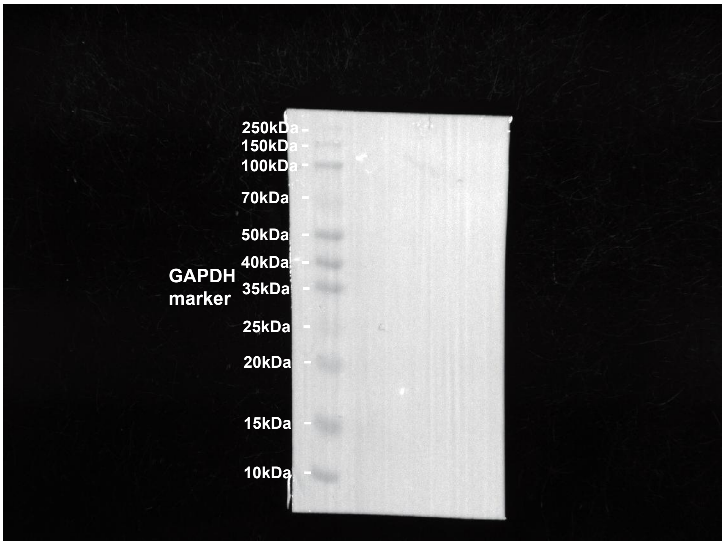


| | | | | |
|---|---|---|---|---|
| Exo$^{OE-NC}$ | - | + | - | - |
| Exo$^{OE-CCT6A}$ | - | - | + | + |
| LY294002 | - | - | - | + |

**Repeat#3**

BxPC-3-Exo

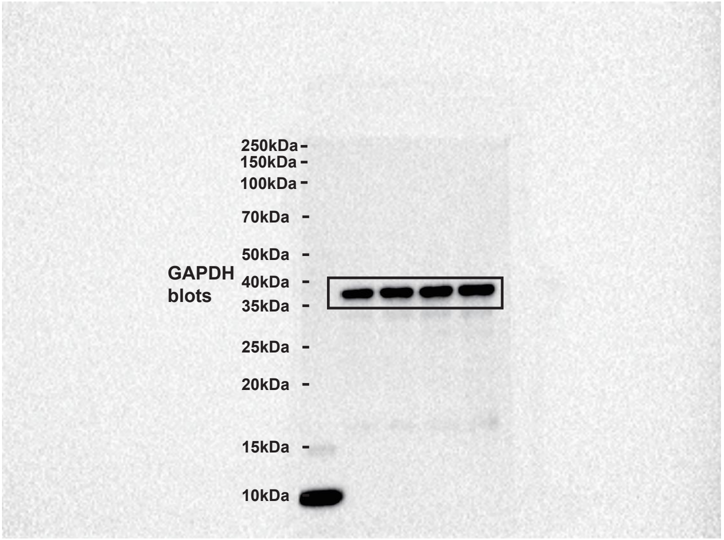


| | | | | |
|---|---|---|---|---|
| Exo$^{OE-NC}$ | - | + | - | - |
| Exo$^{OE-CCT6A}$ | - | - | + | + |
| LY294002 | - | - | - | + |

BxPC-3-Exo

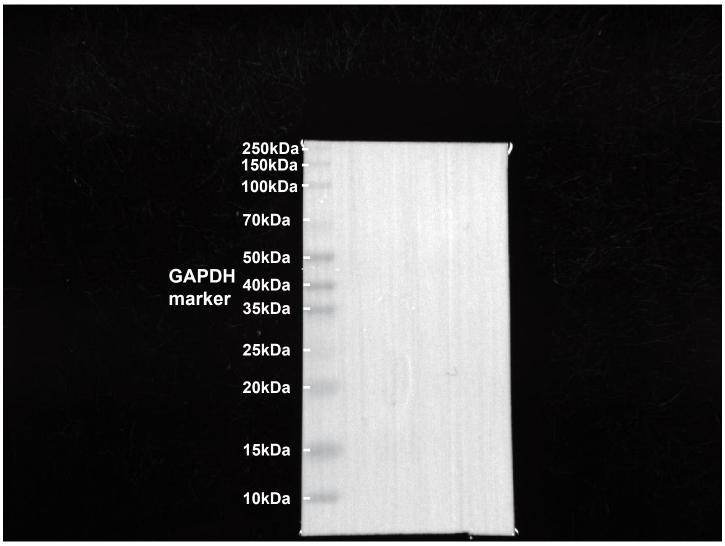


| | | | | |
|---|---|---|---|---|
| Exo$^{OE-NC}$ | - | + | - | - |
| Exo$^{OE-CCT6A}$ | - | - | + | + |
| LY294002 | - | - | - | + |

**Fig. 5C**

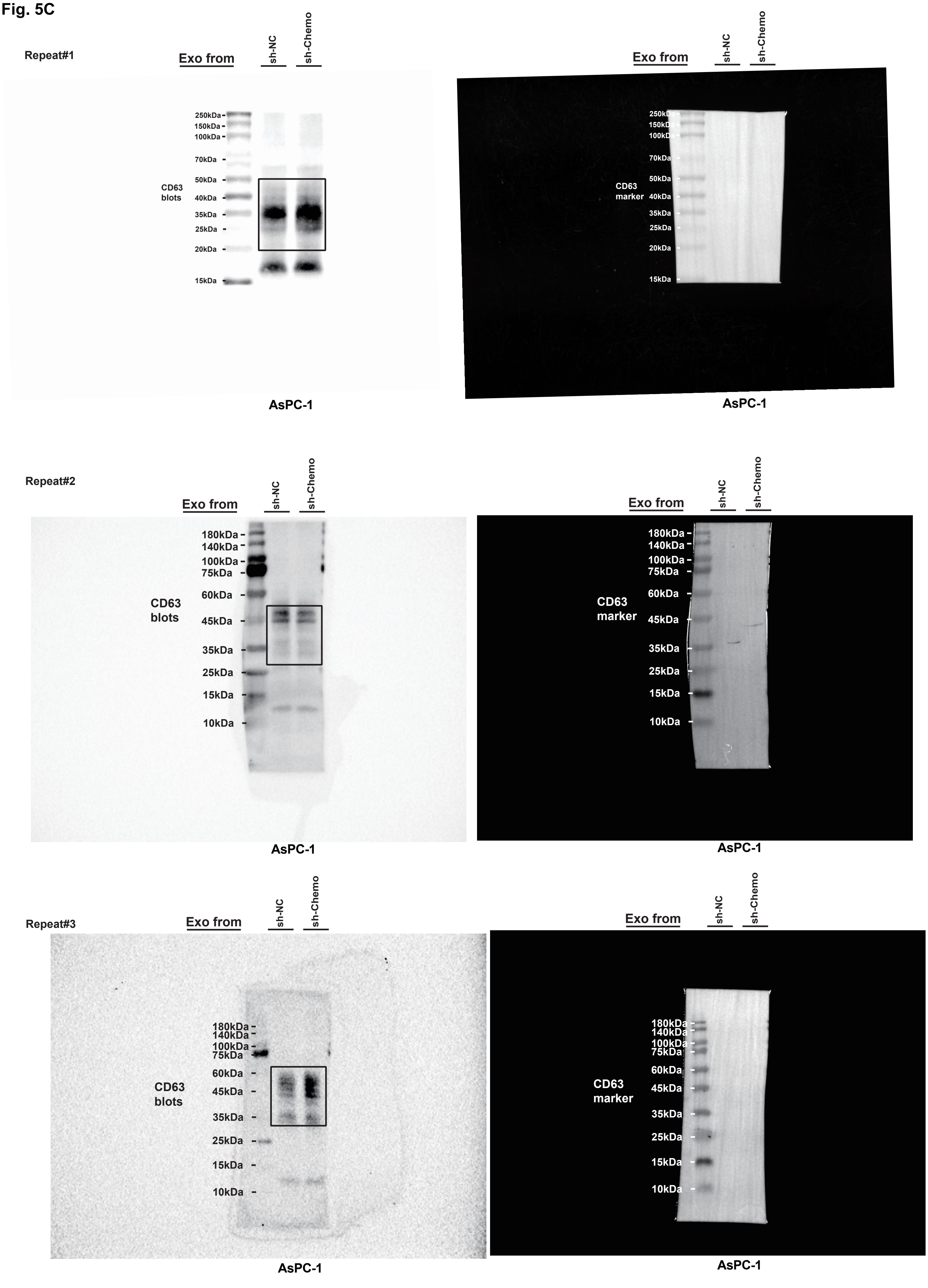
Repeat#1
Exo from
sh-NC
sh-Chemo
250kDa
150kDa
100kDa
70kDa
50kDa
40kDa
35kDa
25kDa
20kDa
15kDa
CD63
blots
AsPC-1
Exo from
sh-NC
sh-Chemo
CD63
marker
AsPC-1
Repeat#2
Exo from
sh-NC
sh-Chemo
180kDa
140kDa
100kDa
75kDa
60kDa
45kDa
35kDa
25kDa
15kDa
10kDa
CD63
blots
AsPC-1
Exo from
sh-NC
sh-Chemo
CD63
marker
AsPC-1
Repeat#3
Exo from
sh-NC
sh-Chemo
180kDa
140kDa
100kDa
75kDa
60kDa
45kDa
35kDa
25kDa
15kDa
10kDa
CD63
blots
AsPC-1
Exo from
sh-NC
sh-Chemo
CD63
marker
AsPC-1

Repeat#1

Exo from | sh-NC | sh-Chemo

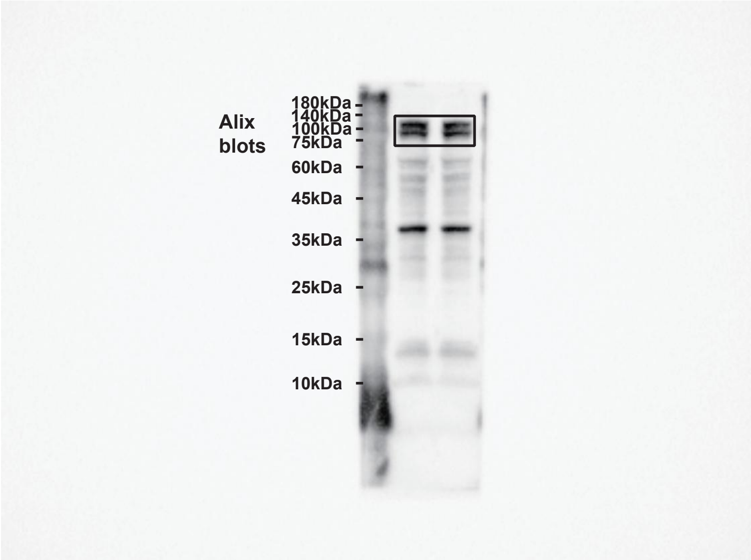


AsPC-1

Exo from | sh-NC | sh-Chemo

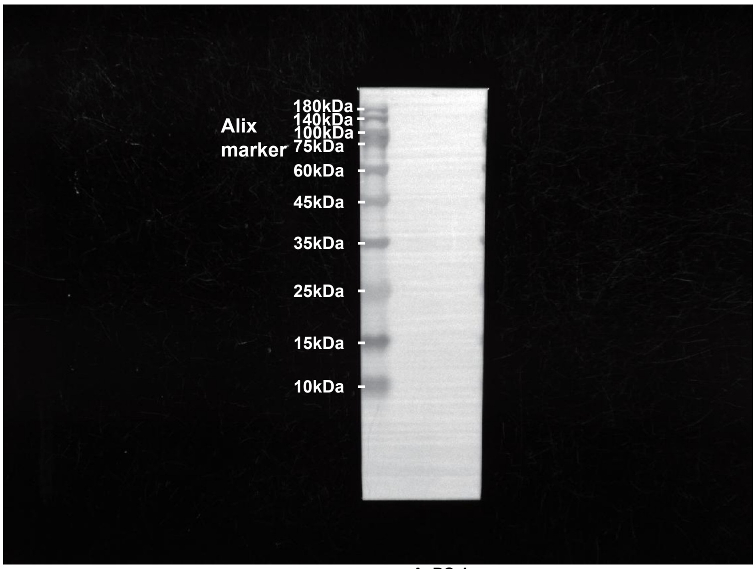


AsPC-1

Repeat#2

Exo from | sh-NC | sh-Chemo

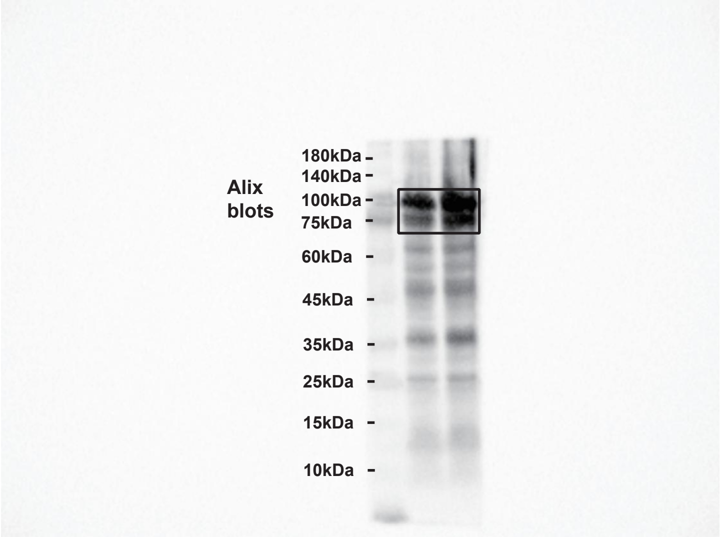


AsPC-1

Exo from | sh-NC | sh-Chemo

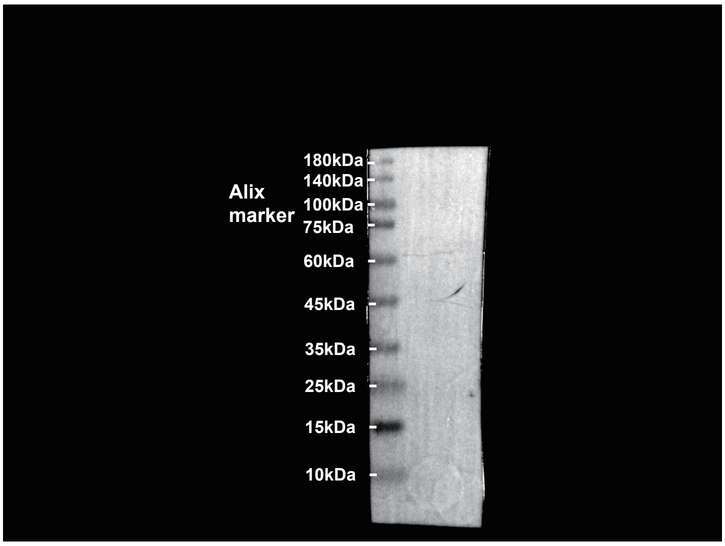


AsPC-1

Repeat#3

Exo from | sh-NC | sh-Chemo

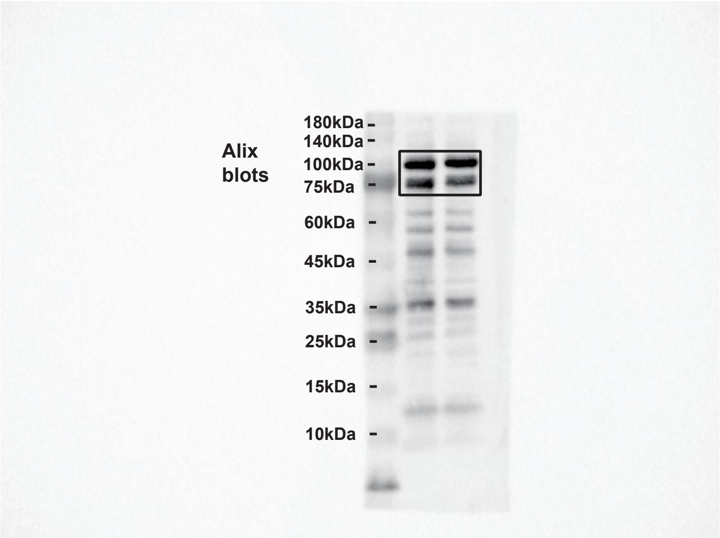


AsPC-1

Exo from | sh-NC | sh-Chemo

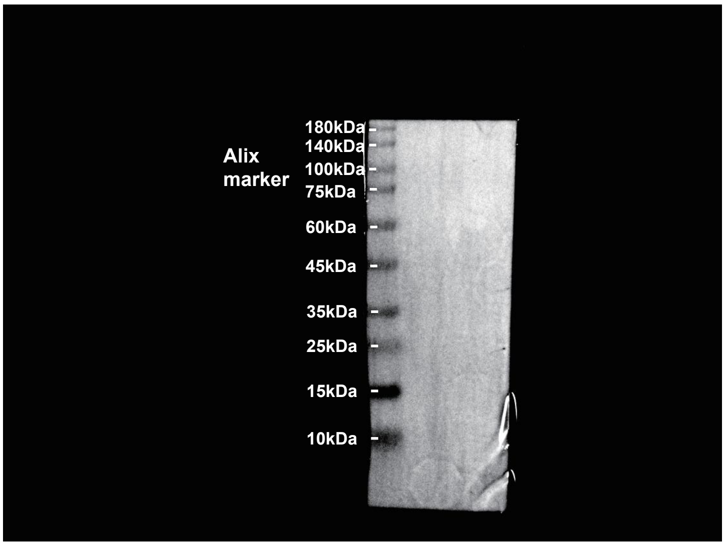


AsPC-1

Repeat#1

Exo from sh-NC sh-Chemo

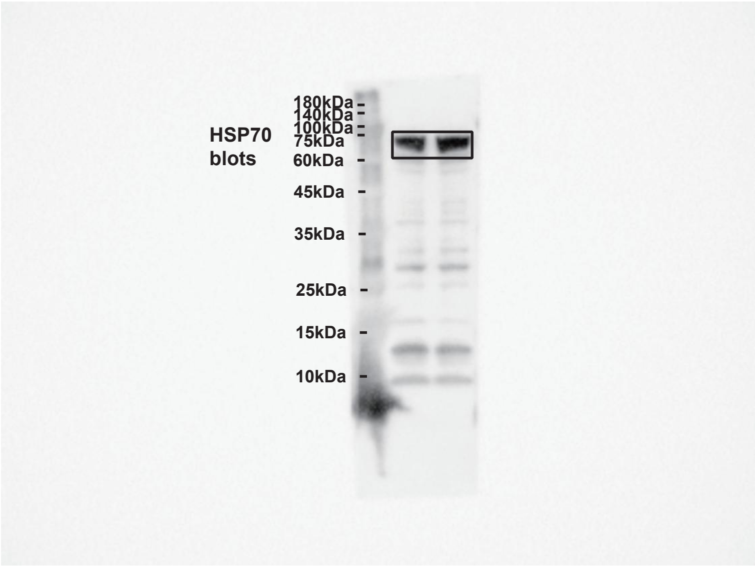


AsPC-1

Exo from sh-NC sh-Chemo

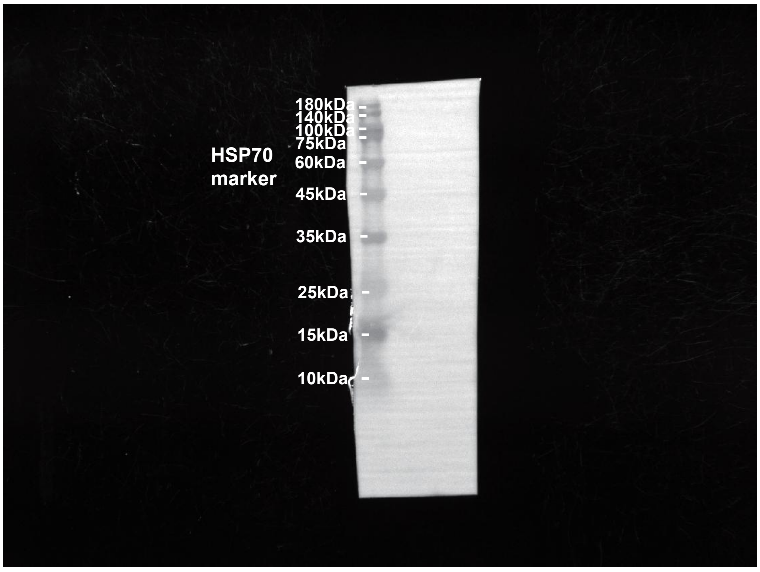


AsPC-1

Repeat#2

Exo from sh-NC sh-Chemo

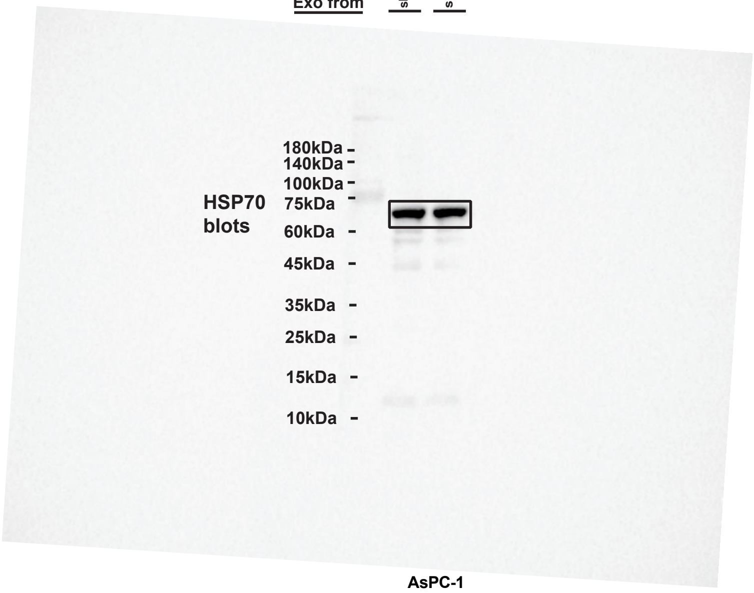


AsPC-1

Exo from sh-NC sh-Chemo

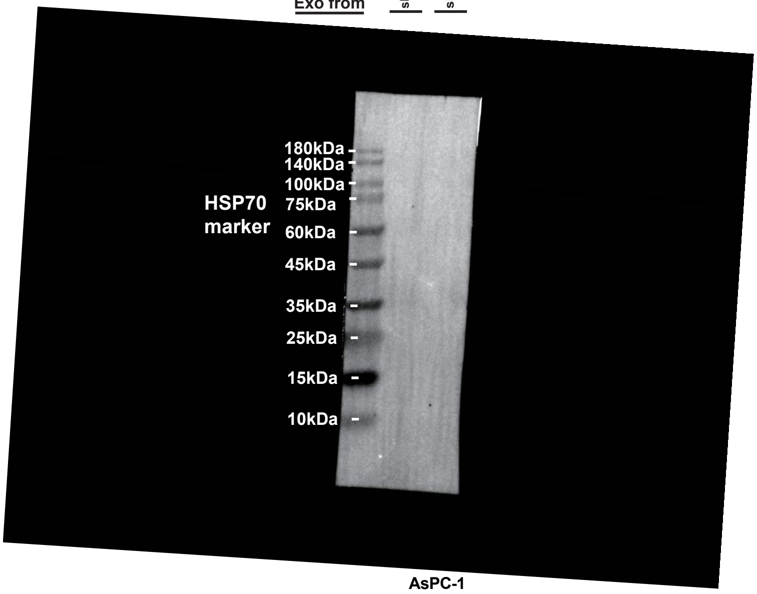


AsPC-1

Repeat#3

Exo from sh-NC sh-Chemo

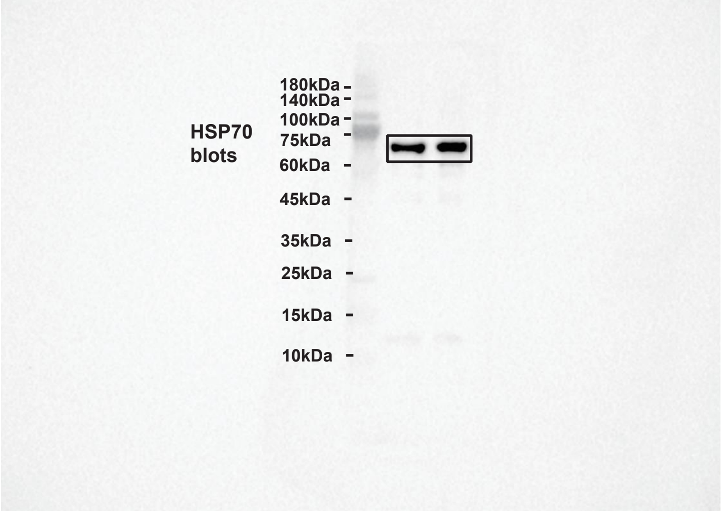


AsPC-1

Exo from sh-NC sh-Chemo

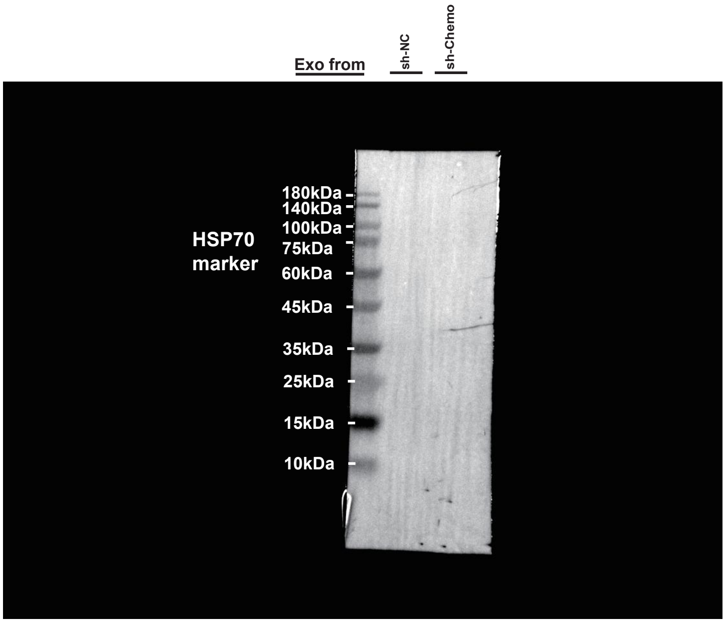


AsPC-1

Repeat#1

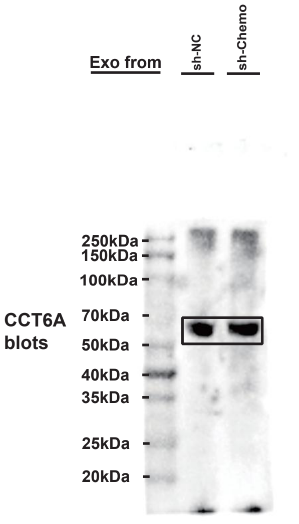


AsPC-1

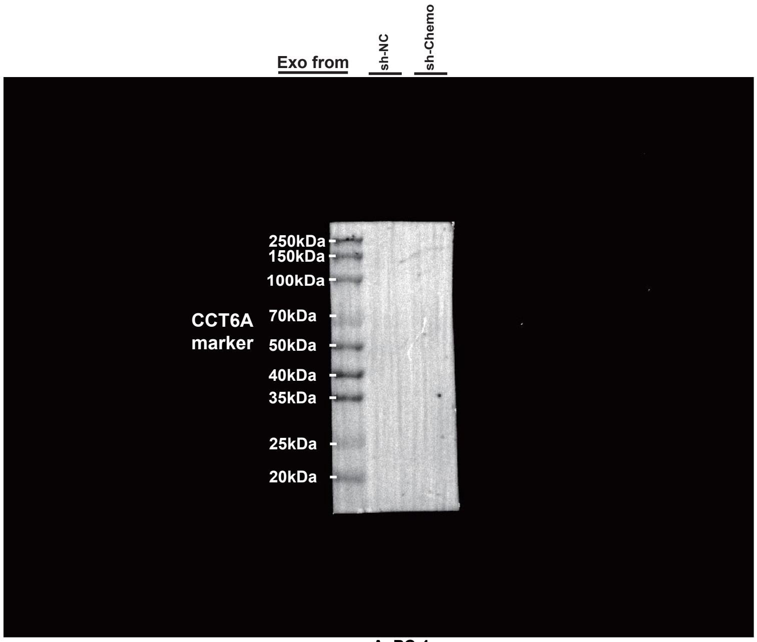


AsPC-1

Repeat#2

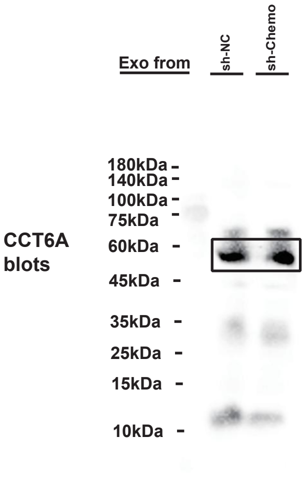


AsPC-1

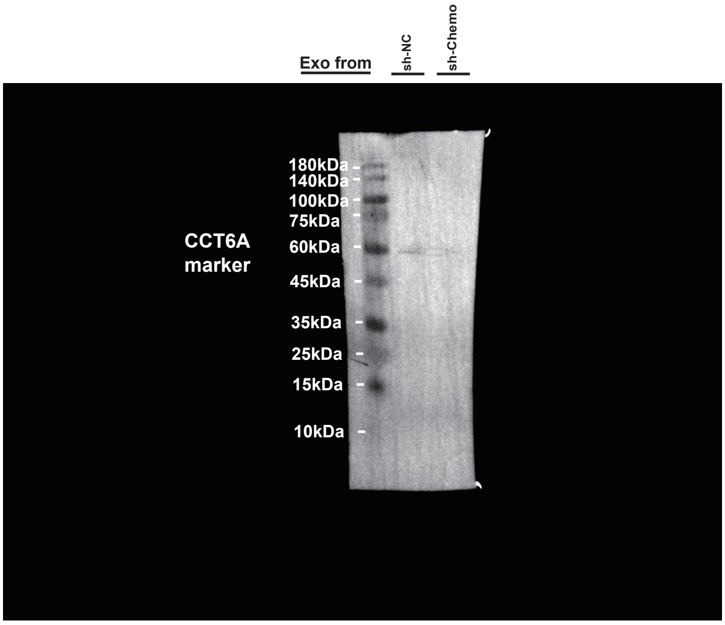


AsPC-1

Repeat#3

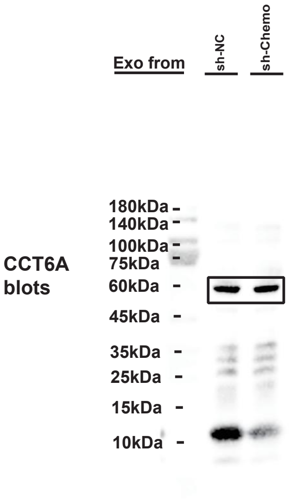


AsPC-1

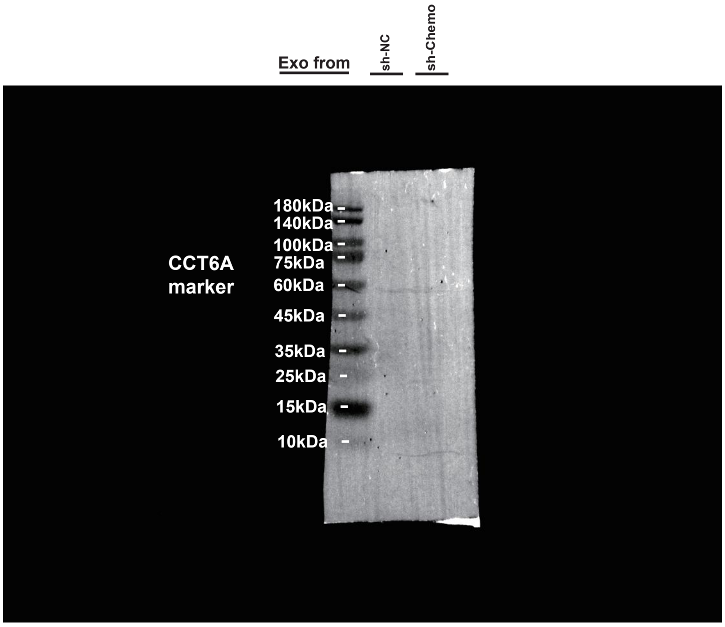


AsPC-1

Repeat#1

Exo from sh-NC sh-Chemo

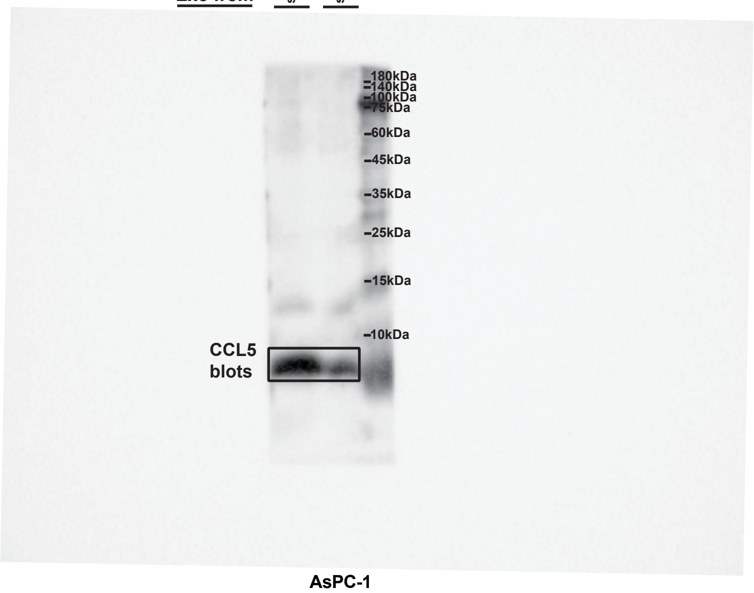


AsPC-1

Exo from sh-NC sh-Chemo

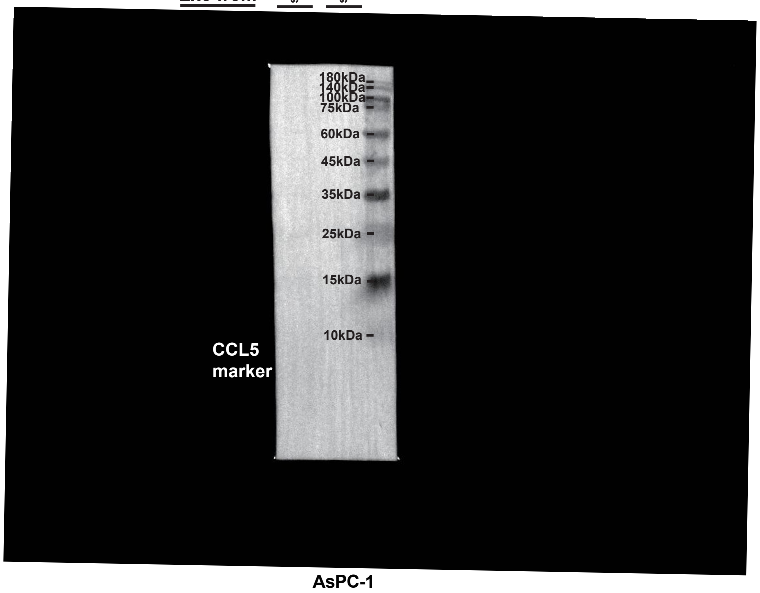


AsPC-1

Repeat#2

Exo from sh-NC sh-Chemo

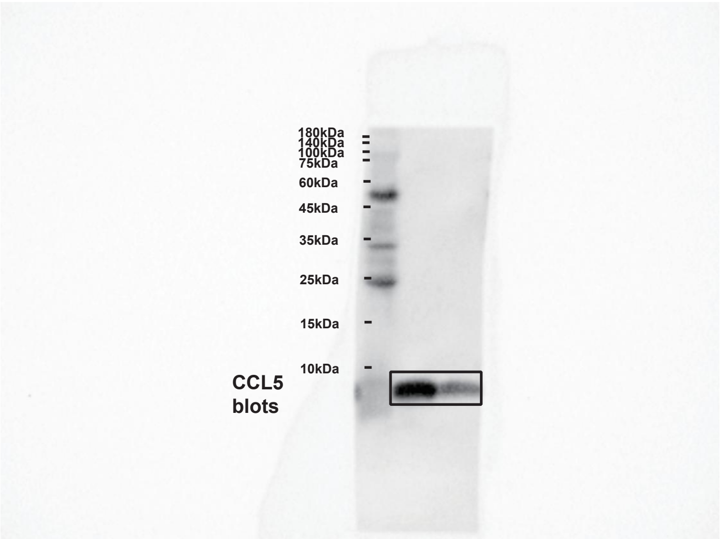


AsPC-1

Exo from sh-NC sh-Chemo

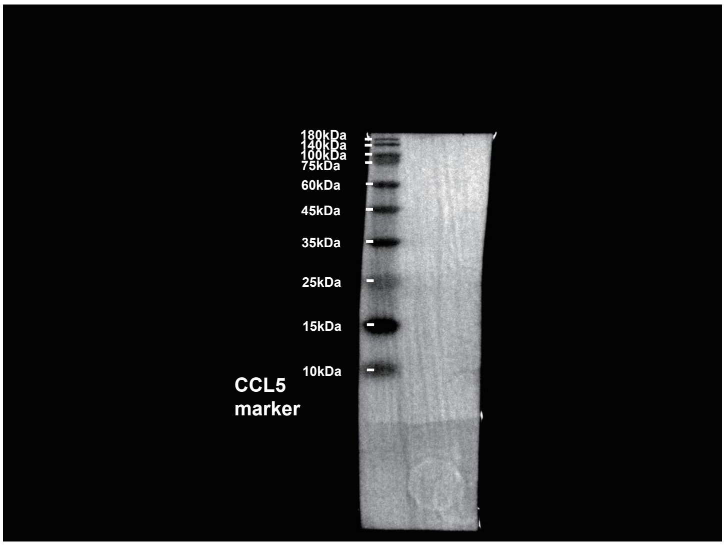


AsPC-1

Repeat#3

Exo from sh-NC sh-Chemo

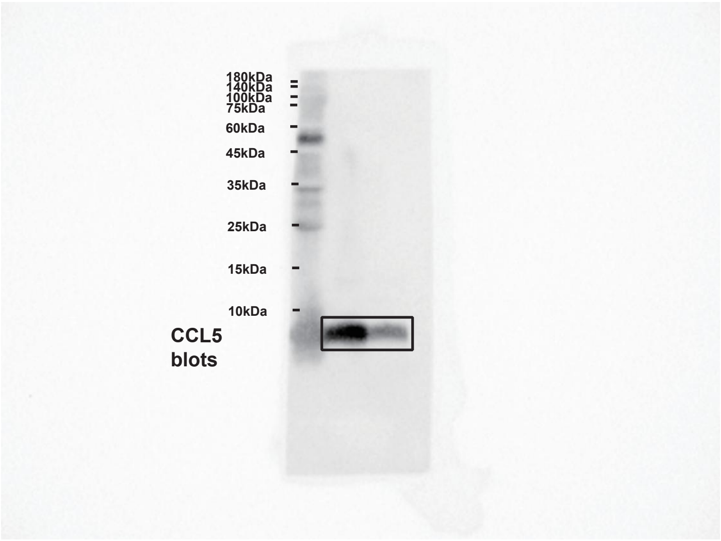


AsPC-1

Exo from sh-NC sh-Chemo

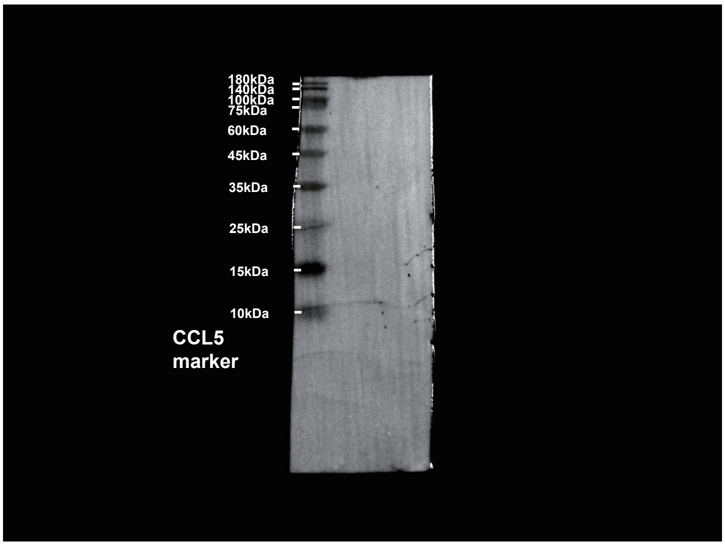


AsPC-1

Repeat#1

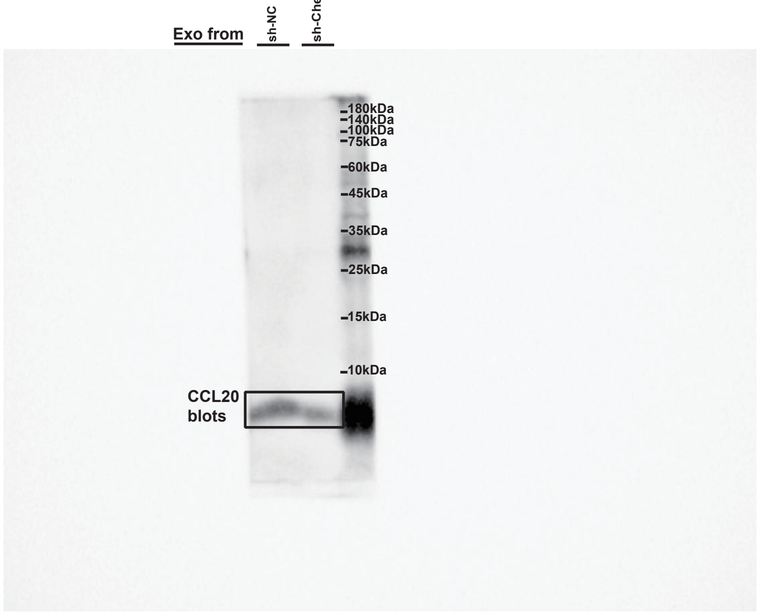


AsPC-1

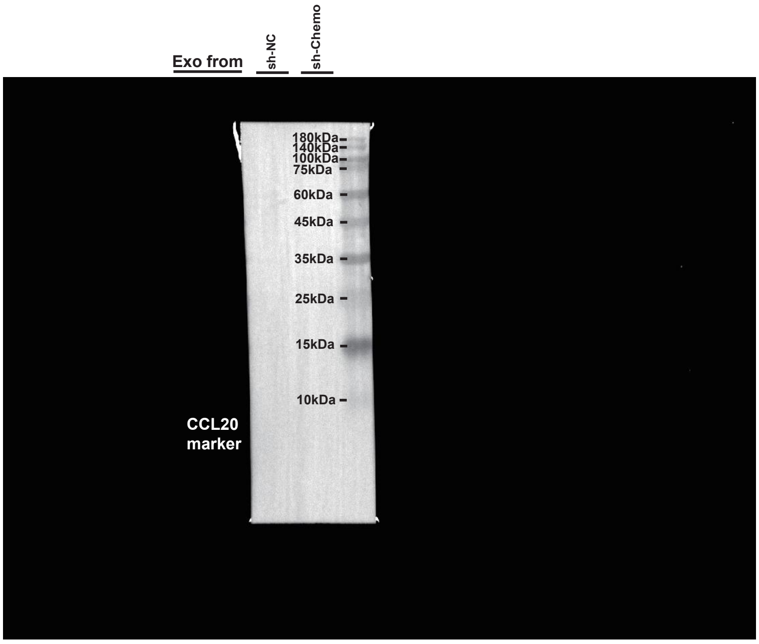


AsPC-1

Repeat#2

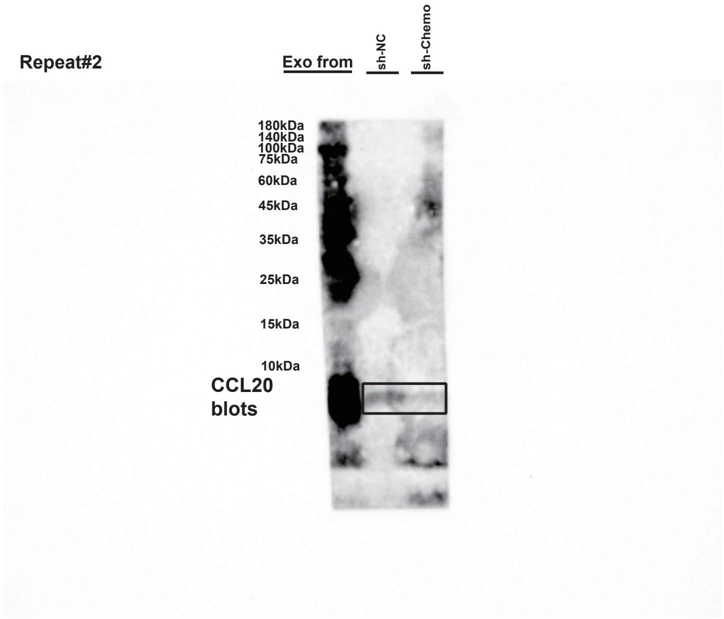


AsPC-1

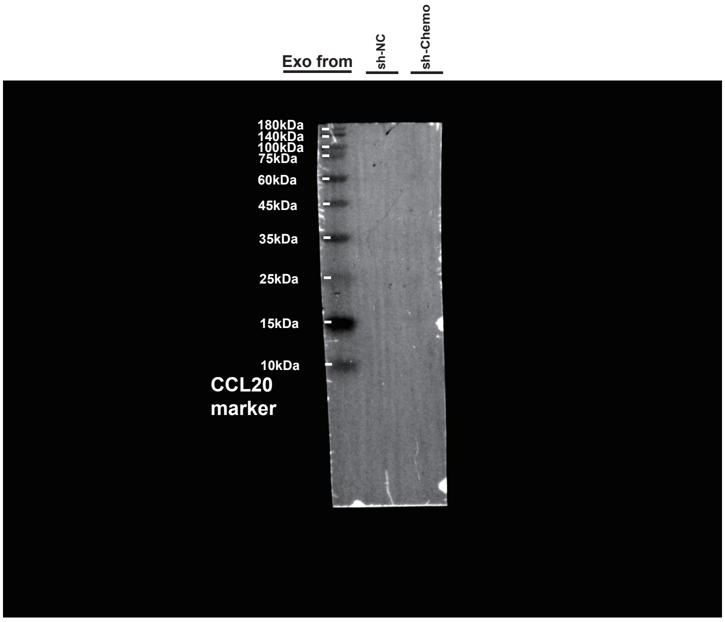


AsPC-1

Repeat#3

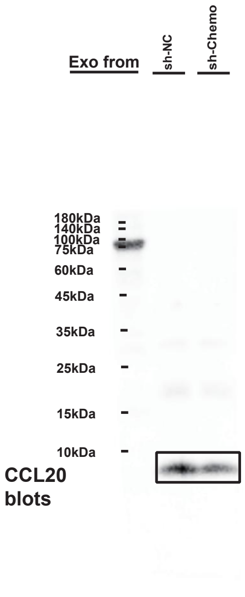


AsPC-1

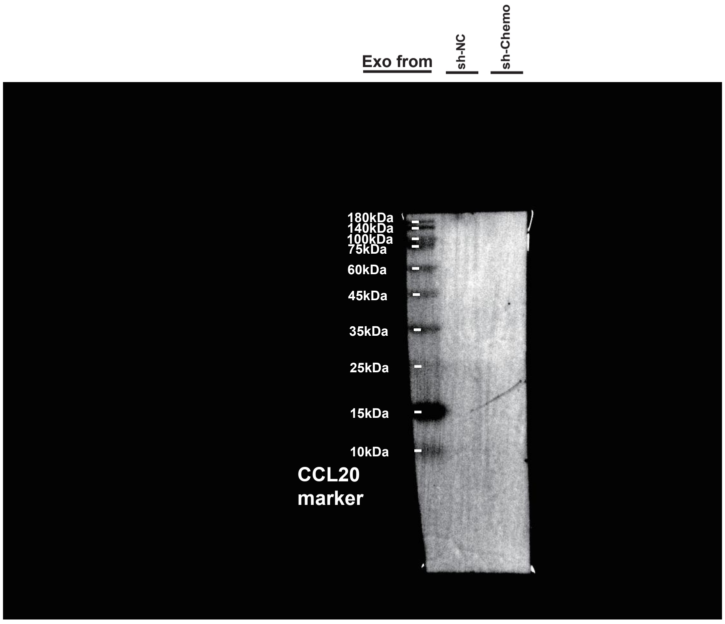


AsPC-1

**Repeat#1**

Exo from sh-NC sh-Chemo

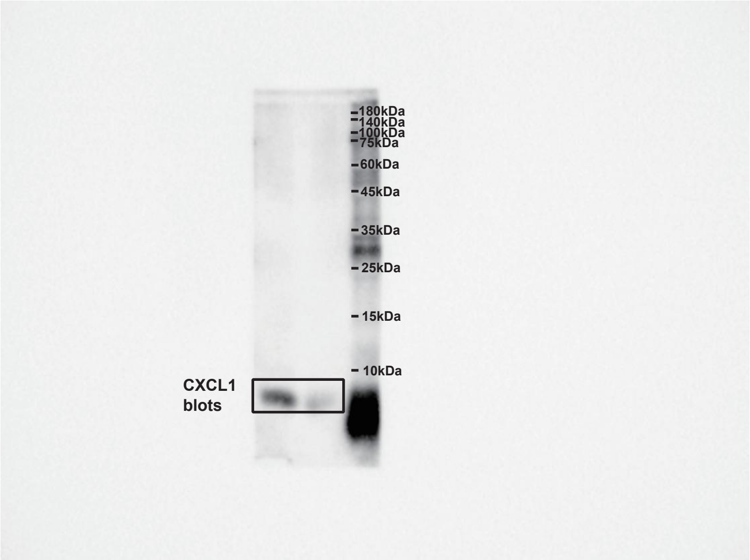


AsPC-1

Exo from sh-NC sh-Chemo

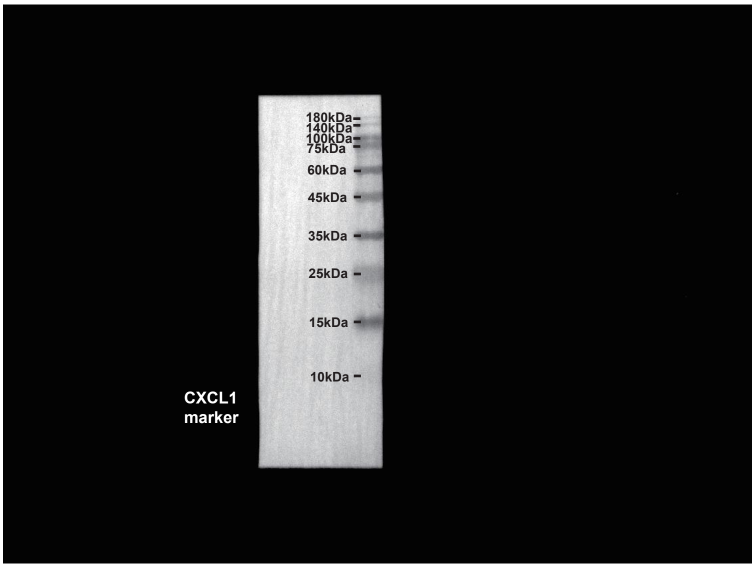


AsPC-1

**Repeat#2**

Exo from sh-NC sh-Chemo

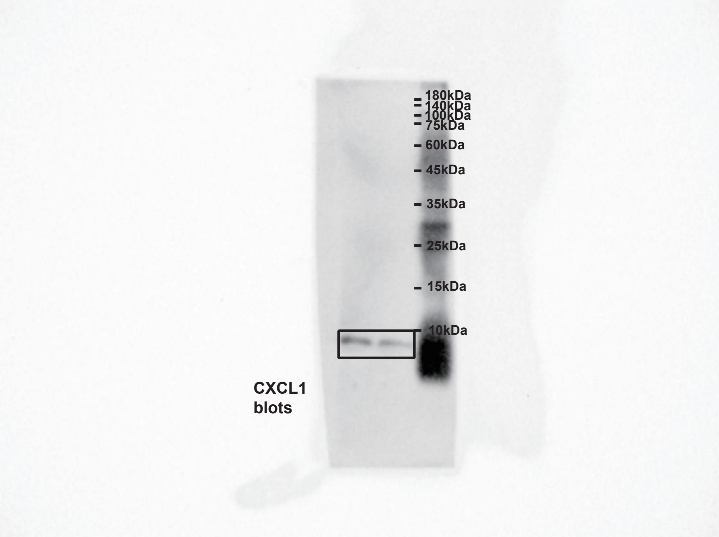


AsPC-1

Exo from sh-NC sh-Chemo

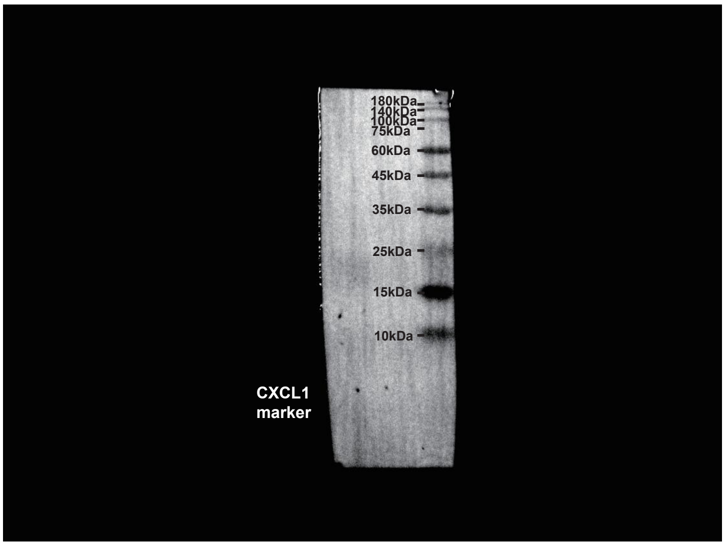


AsPC-1

**Repeat#3**

Exo from sh-NC sh-Chemo

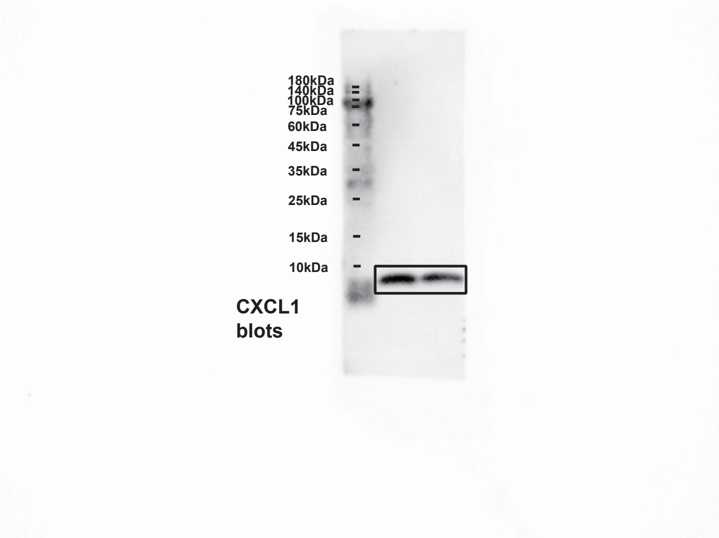


AsPC-1

Exo from sh-NC sh-Chemo

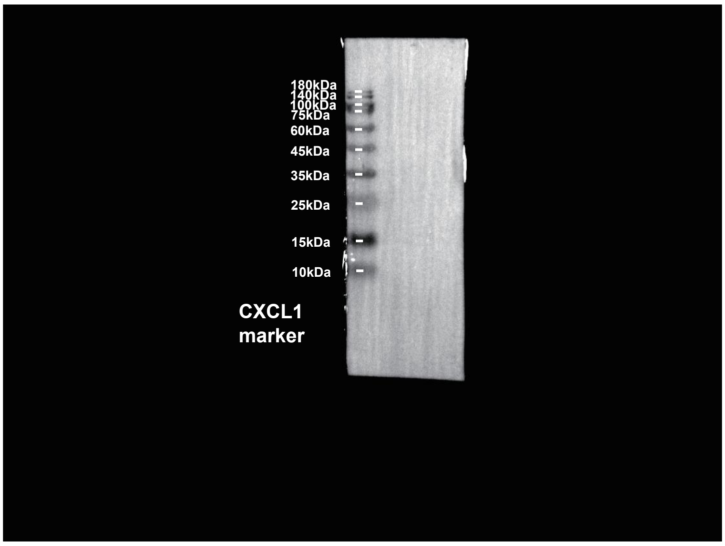


AsPC-1

Repeat#1

Exo from sh-NC sh-Chemo

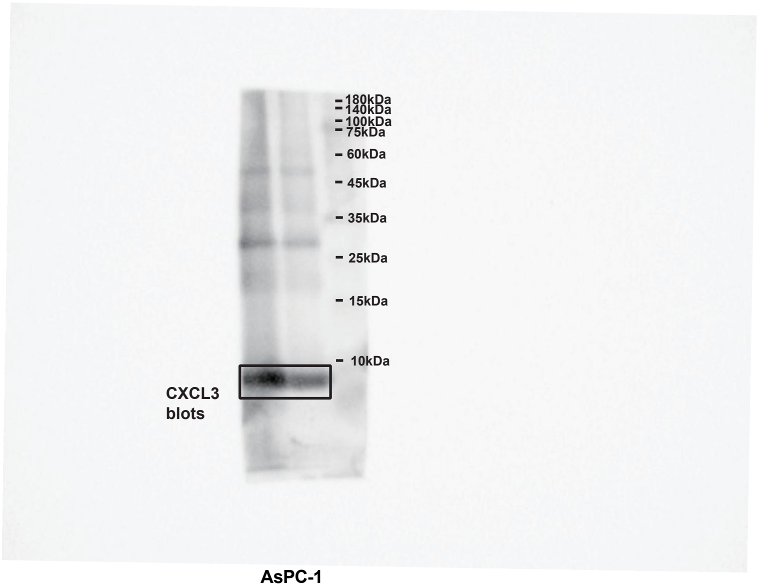


AsPC-1

Exo from sh-NC sh-Chemo

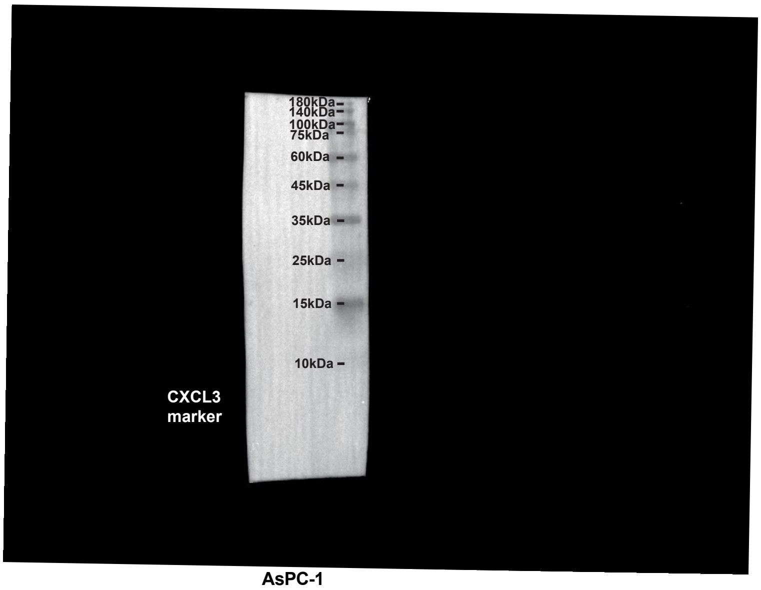


AsPC-1

Repeat#2

Exo from sh-NC sh-Chemo

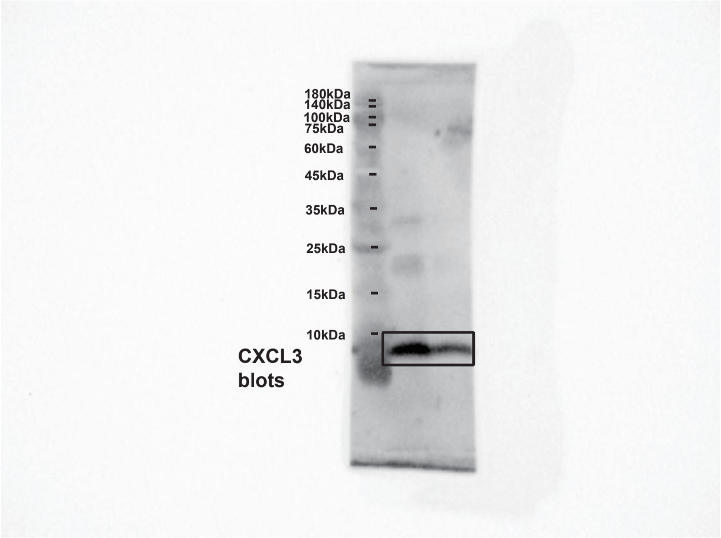


AsPC-1

Exo from sh-NC sh-Chemo

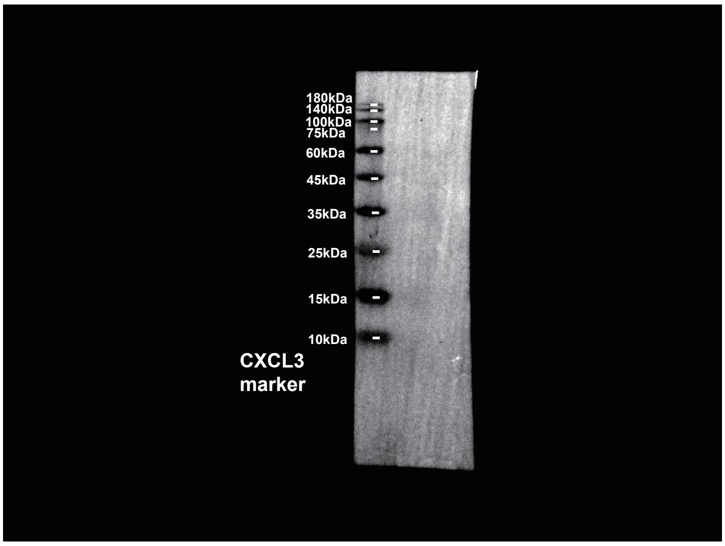


AsPC-1

Repeat#3

Exo from sh-NC sh-Chemo

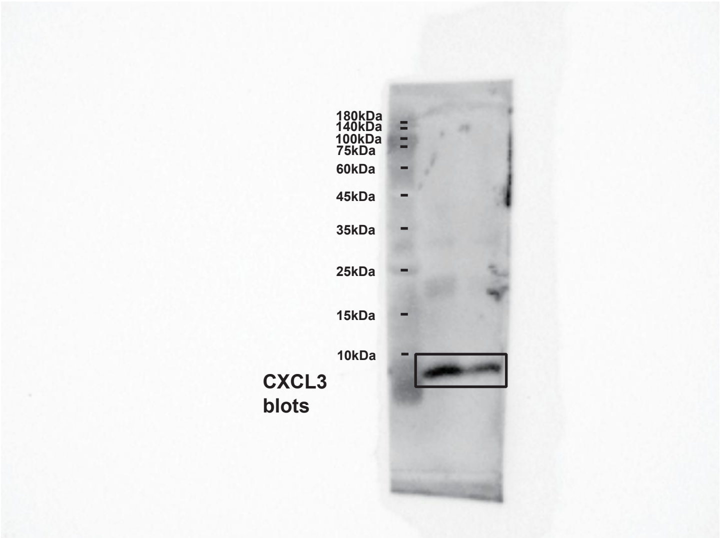


AsPC-1

Exo from sh-NC sh-Chemo

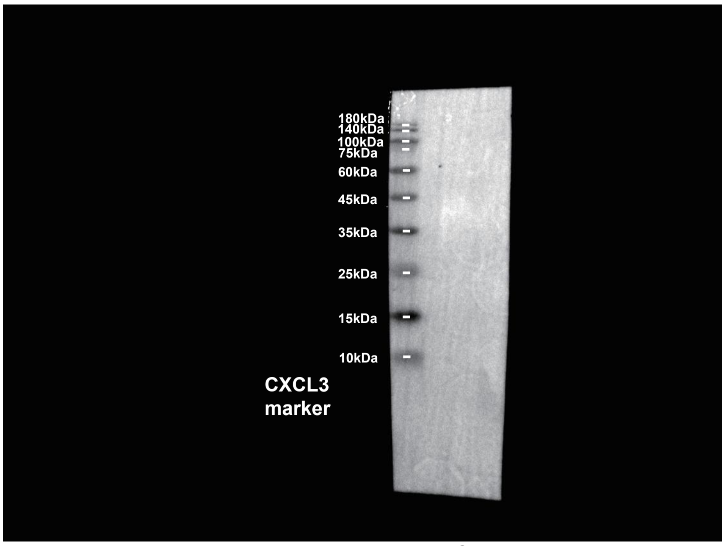


AsPC-1

**Fig. 5D**

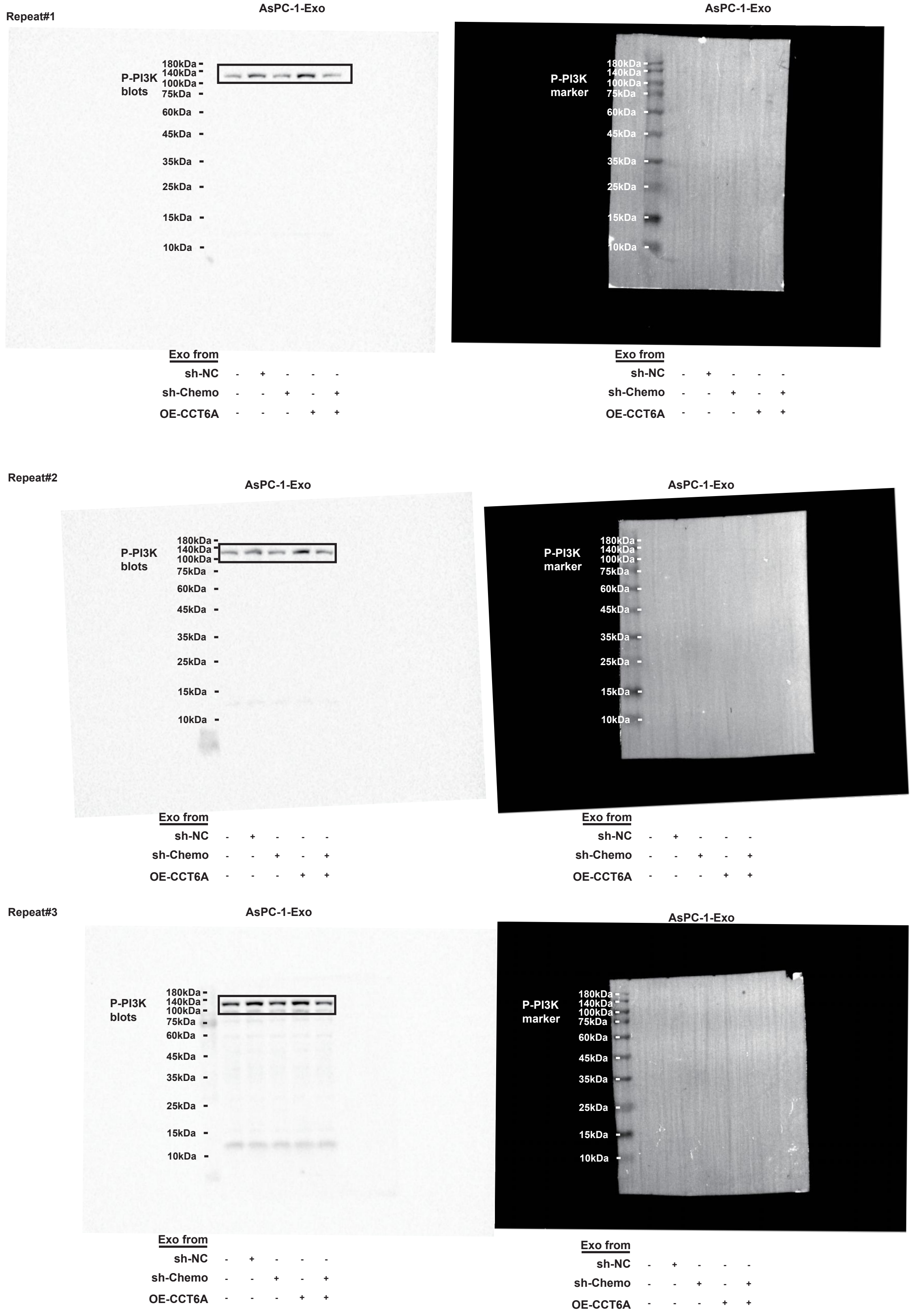


| | p-PI3K | PI3K |
|---|---|---|
| Saline | 6419.25 | 18111.295 |
| OE-NC | 11556.75 | 15369.366 |
| sh-chemokines | 7448.875 | 18649.795 |
| OE-CCT6A | 15268.5 | 16772.545 |
| sh-chemokines+OE-CCT6A | 6458.125 | 17192.518 |
| | | |
| Saline | 7266.5765 | 13803.85 |
| OE-NC | 10979.129 | 17359.693 |
| sh-chemokines | 7270.7765 | 13918.895 |
| OE-CCT6A | 14578.035 | 15229.752 |
| sh-chemokines+OE-CCT6A | 8006.2706 | 14261.654 |
| | | |
| Saline | 7039.3775 | 15035.875 |
| OE-NC | 9591.2617 | 11530.803 |
| sh-chemokines | 6875.7475 | 12676 |
| OE-CCT6A | 9376.7893 | 10124.454 |
| sh-chemokines+OE-CCT6A | 5773.55 | 10493.98 |

Repeat#1

AsPC-1-Exo

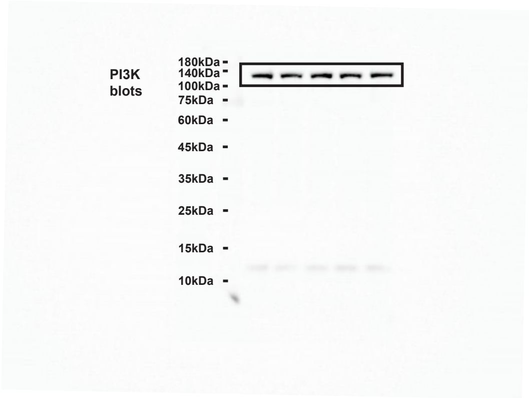


Exo from

| | | | | | |
|---|---|---|---|---|---|
| sh-NC | - | + | - | - | - |
| sh-Chemo | - | - | + | - | + |
| OE-CCT6A | - | - | - | + | + |

AsPC-1-Exo

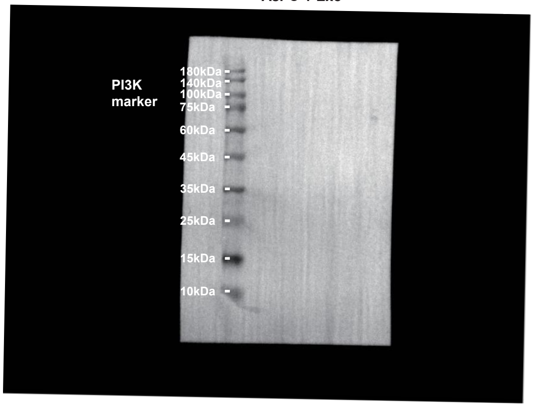


Exo from

| | | | | | |
|---|---|---|---|---|---|
| sh-NC | - | + | - | - | - |
| sh-Chemo | - | - | + | - | + |
| OE-CCT6A | - | - | - | + | + |

Repeat#2

AsPC-1-Exo

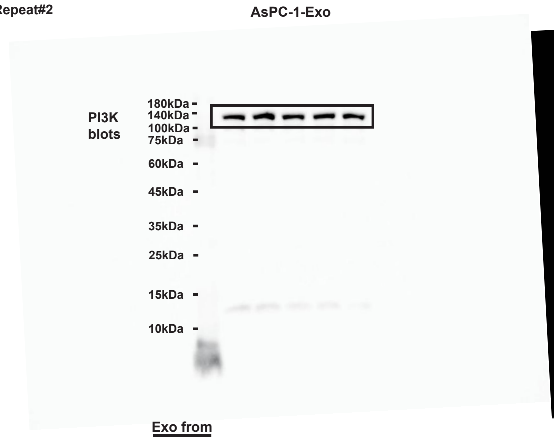


Exo from

| | | | | | |
|---|---|---|---|---|---|
| sh-NC | - | + | - | - | - |
| sh-Chemo | - | - | + | - | + |
| OE-CCT6A | - | - | - | + | + |

AsPC-1-Exo

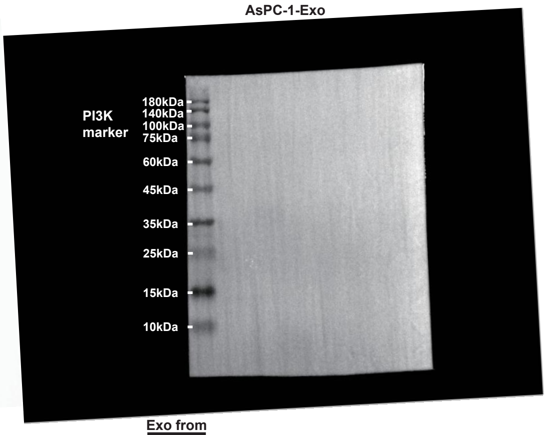


Exo from

| | | | | | |
|---|---|---|---|---|---|
| sh-NC | - | + | - | - | - |
| sh-Chemo | - | - | + | - | + |
| OE-CCT6A | - | - | - | + | + |

Repeat#3

AsPC-1-Exo

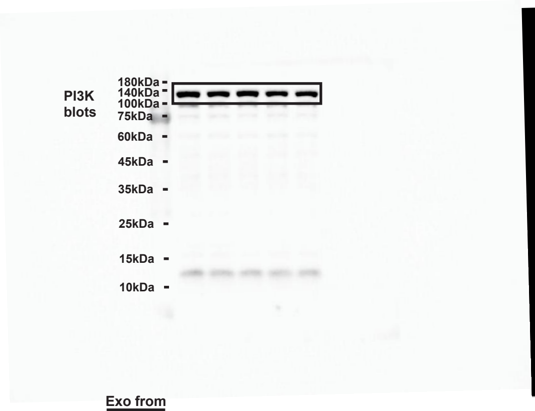


Exo from

| | | | | | |
|---|---|---|---|---|---|
| sh-NC | - | + | - | - | - |
| sh-Chemo | - | - | + | - | + |
| OE-CCT6A | - | - | - | + | + |

AsPC-1-Exo

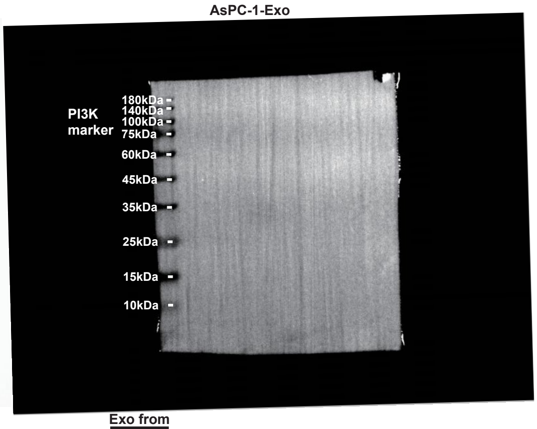


Exo from

| | | | | | |
|---|---|---|---|---|---|
| sh-NC | - | + | - | - | - |
| sh-Chemo | - | - | + | - | + |
| OE-CCT6A | - | - | - | + | + |

| | p-PI3K | PI3K |
|---|---|---|
| Saline | 6419.25 | 18111.295 |
| OE-NC | 11556.75 | 15369.366 |
| sh-chemokines | 7448.875 | 18649.795 |
| OE-CCT6A | 15268.5 | 16772.545 |
| sh-chemokines+OE-CCT6A | 6458.125 | 17192.518 |
| | | |
| Saline | 7266.5765 | 13803.85 |
| OE-NC | 10979.129 | 17359.693 |
| sh-chemokines | 7270.7765 | 13918.895 |
| OE-CCT6A | 14578.035 | 15229.752 |
| sh-chemokines+OE-CCT6A | 8006.2706 | 14261.654 |
| | | |
| Saline | 7039.3775 | 15035.875 |
| OE-NC | 9591.2617 | 11530.803 |
| sh-chemokines | 6875.7475 | 12676 |
| OE-CCT6A | 9376.7893 | 10124.454 |
| sh-chemokines+OE-CCT6A | 5773.55 | 10493.98 |

Repeat#1

AsPC-1-Exo

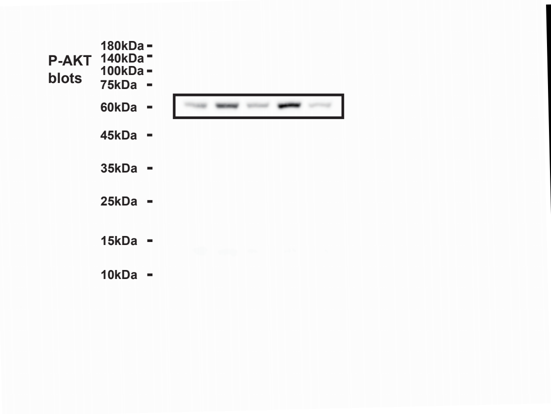


| Exo from | | | | | |
|---|---|---|---|---|---|
| sh-NC | - | + | - | - | - |
| sh-Chemo | - | - | + | - | + |
| OE-CCT6A | - | - | - | + | + |

AsPC-1-Exo

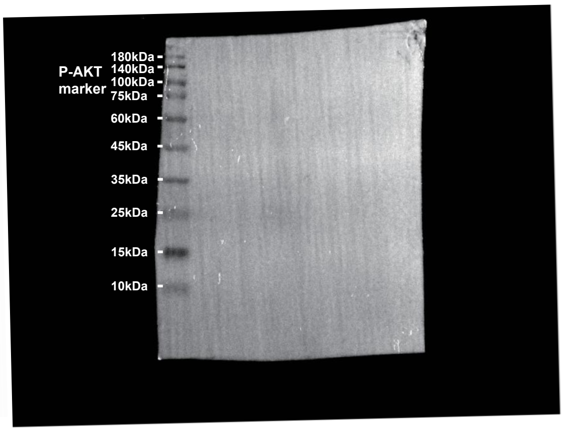


| Exo from | | | | | |
|---|---|---|---|---|---|
| sh-NC | - | + | - | - | - |
| sh-Chemo | - | - | + | - | + |
| OE-CCT6A | - | - | - | + | + |

Repeat#2

AsPC-1-Exo

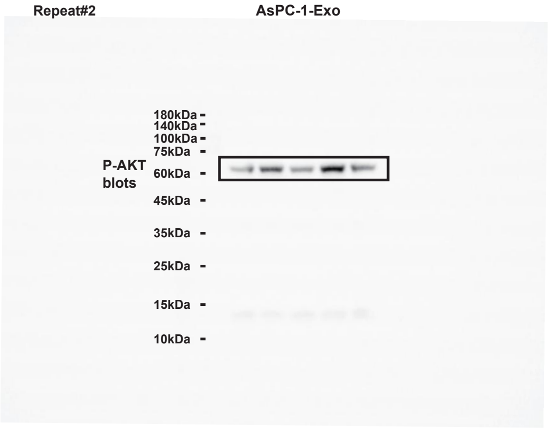


| Exo from | | | | | |
|---|---|---|---|---|---|
| sh-NC | - | + | - | - | - |
| sh-Chemo | - | - | + | - | + |
| OE-CCT6A | - | - | - | + | + |

AsPC-1-Exo

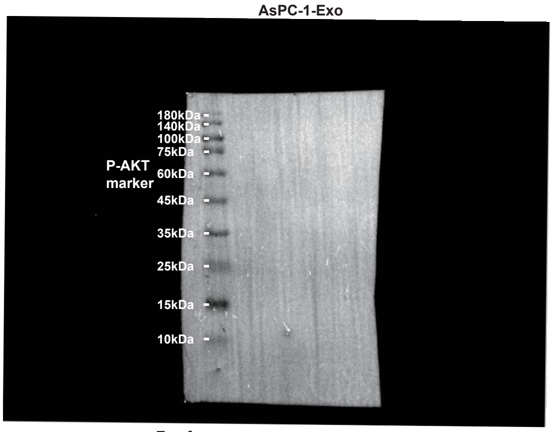


| Exo from | | | | | |
|---|---|---|---|---|---|
| sh-NC | - | + | - | - | - |
| sh-Chemo | - | - | + | - | + |
| OE-CCT6A | - | - | - | + | + |

Repeat#3

AsPC-1-Exo

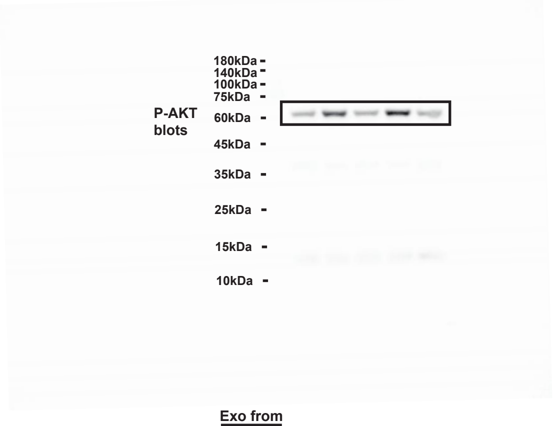


| Exo from | | | | | |
|---|---|---|---|---|---|
| sh-NC | - | + | - | - | - |
| sh-Chemo | - | - | + | - | + |
| OE-CCT6A | - | - | - | + | + |

AsPC-1-Exo

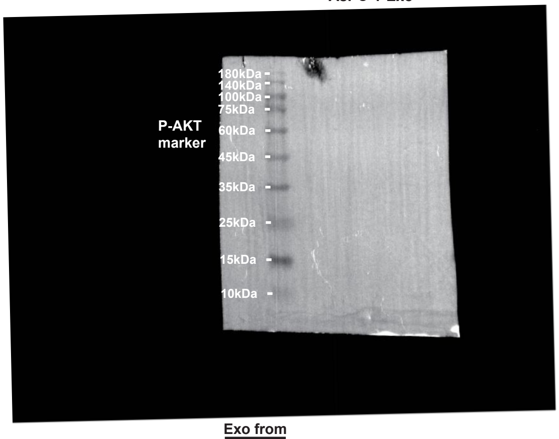


| Exo from | | | | | |
|---|---|---|---|---|---|
| sh-NC | - | + | - | - | - |
| sh-Chemo | - | - | + | - | + |
| OE-CCT6A | - | - | - | + | + |

| | p-AKT | AKT |
|---|---|---|
| Saline | 6327.775 | 15701.556 |
| OE-NC | 10117.865 | 13841.025 |
| sh-chemokines | 7153.6 | 16996.741 |
| OE-CCT6A | 17632.775 | 18707.784 |
| sh-chemokines+OE-CCT6A | 9366.145 | 20307.099 |
| | | |
| Saline | 7930.125 | 19503.34 |
| OE-NC | 14148.39 | 19459.142 |
| sh-chemokines | 7525.2162 | 19044.574 |
| OE-CCT6A | 16508.893 | 17533.525 |
| sh-chemokines+OE-CCT6A | 5535.8934 | 15818.537 |
| | | |
| Saline | 9522.8 | 24763.594 |
| OE-NC | 18295.435 | 24228.85 |
| sh-chemokines | 10244.546 | 25786.023 |
| OE-CCT6A | 20803.435 | 23619.083 |
| sh-chemokines+OE-CCT6A | 10961.67 | 27441.105 |

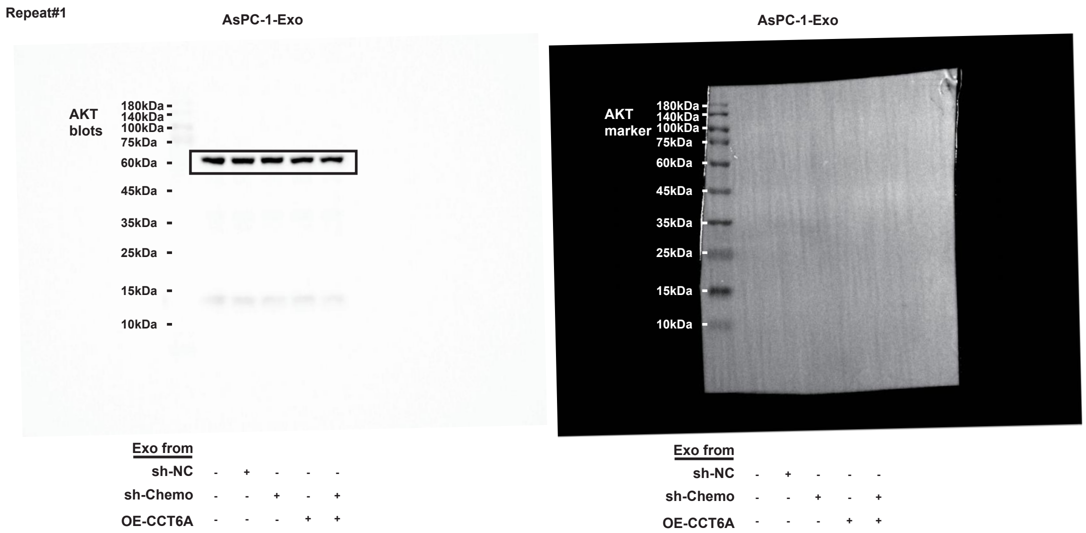


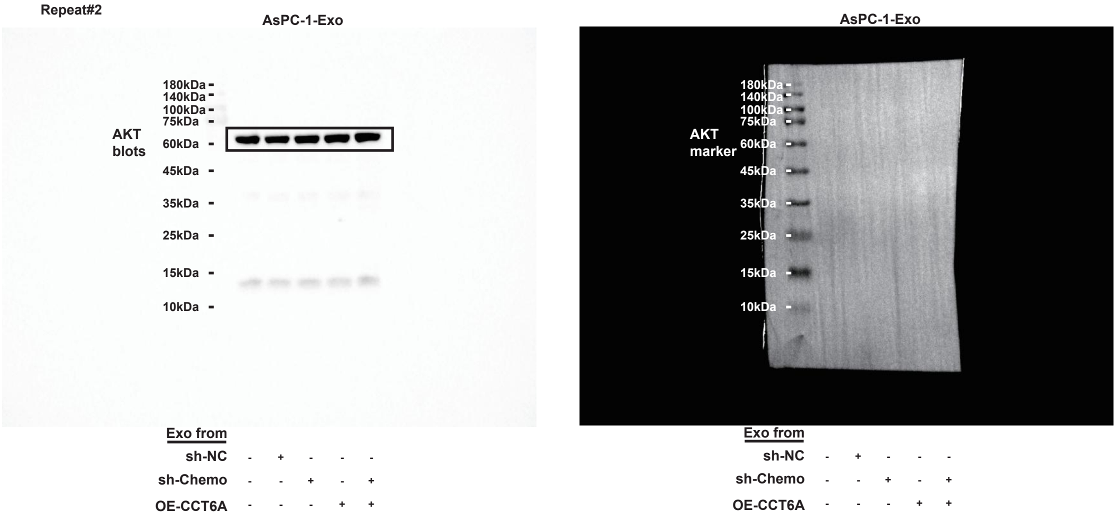


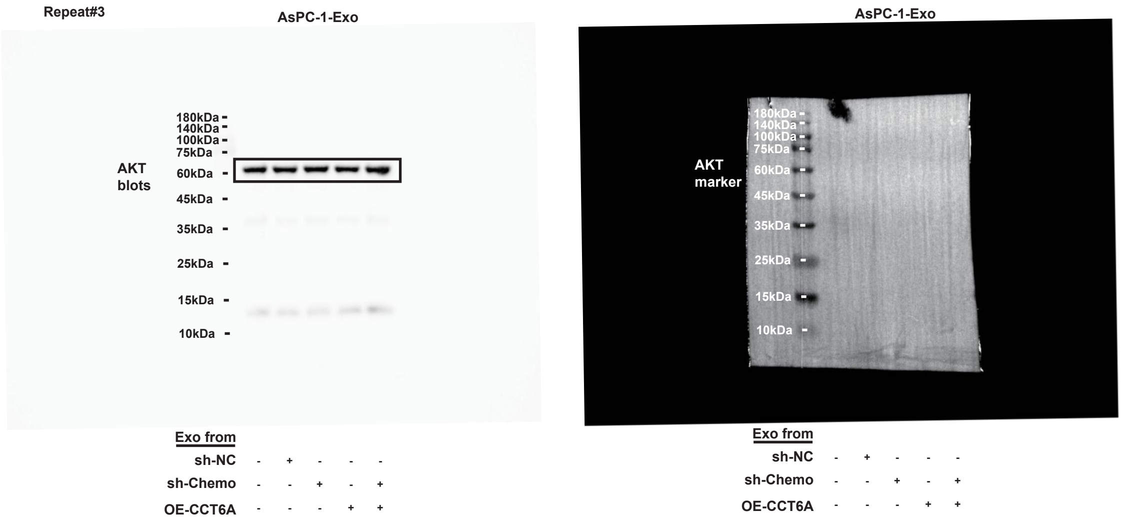


| | p-AKT | AKT |
|---|---|---|
| Saline | 6327.775 | 15701.556 |
| OE-NC | 10117.865 | 13841.025 |
| sh-chemokines | 7153.6 | 16996.741 |
| OE-CCT6A | 17632.775 | 18707.784 |
| sh-chemokines+OE-CCT6A | 9366.145 | 20307.099 |
| | | |
| Saline | 7930.125 | 19503.34 |
| OE-NC | 14148.39 | 19459.142 |
| sh-chemokines | 7525.2162 | 19044.574 |
| OE-CCT6A | 16508.893 | 17533.525 |
| sh-chemokines+OE-CCT6A | 5535.8934 | 15818.537 |
| | | |
| Saline | 9522.8 | 24763.594 |
| OE-NC | 18295.435 | 24228.85 |
| sh-chemokines | 10244.546 | 25786.023 |
| OE-CCT6A | 20803.435 | 23619.083 |
| sh-chemokines+OE-CCT6A | 10961.67 | 27441.105 |

Repeat#1

AsPC-1-Exo

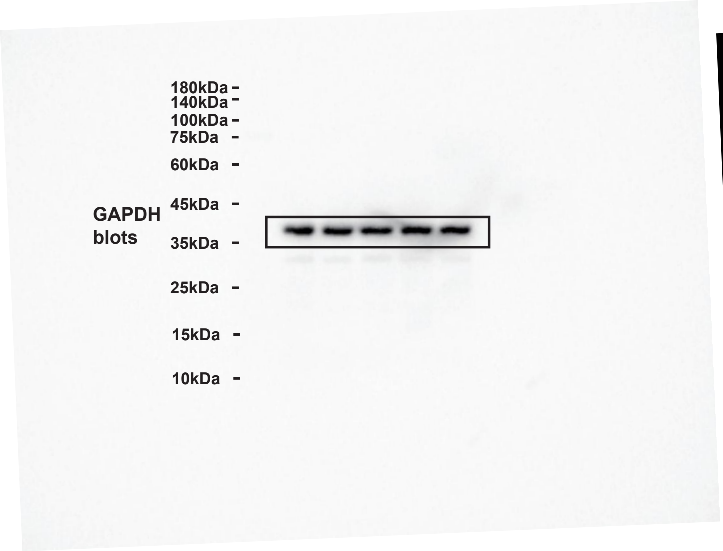


| Exo from | | | | | |
|---|---|---|---|---|---|
| sh-NC | - | + | - | - | - |
| sh-Chemo | - | - | + | - | + |
| OE-CCT6A | - | - | - | + | + |

AsPC-1-Exo

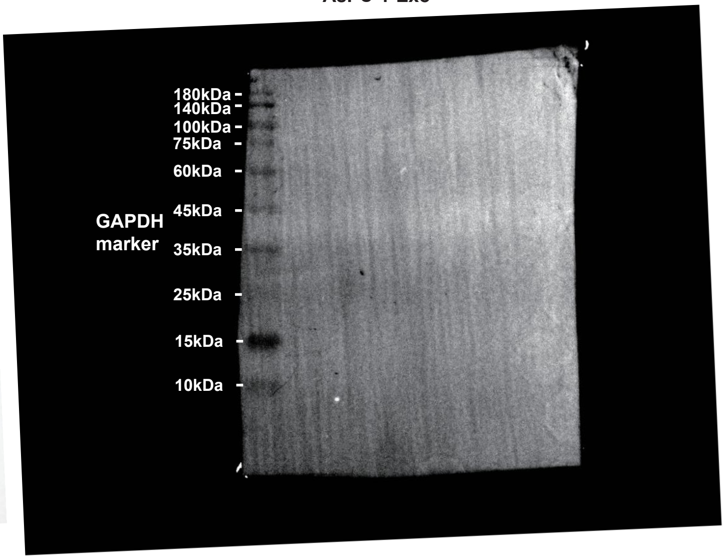


| Exo from | | | | | |
|---|---|---|---|---|---|
| sh-NC | - | + | - | - | - |
| sh-Chemo | - | - | + | - | + |
| OE-CCT6A | - | - | - | + | + |

Repeat#2

AsPC-1-Exo

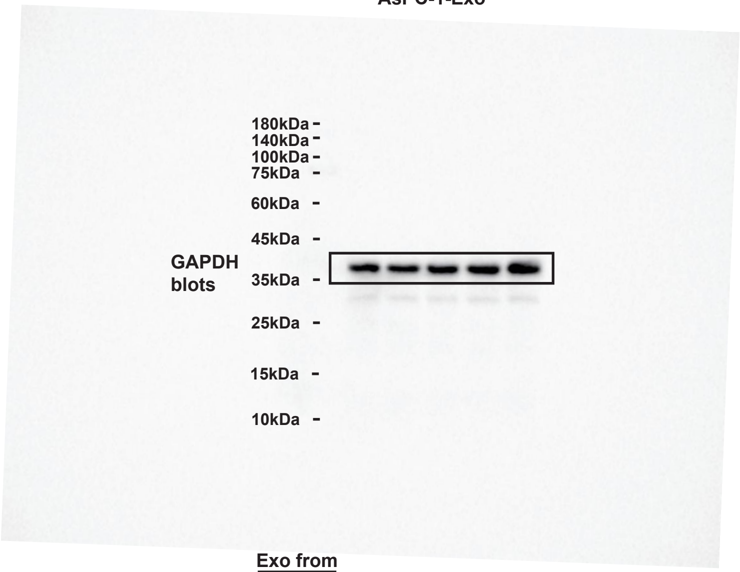


| Exo from | | | | | |
|---|---|---|---|---|---|
| sh-NC | - | + | - | - | - |
| sh-Chemo | - | - | + | - | + |
| OE-CCT6A | - | - | - | + | + |

AsPC-1-Exo

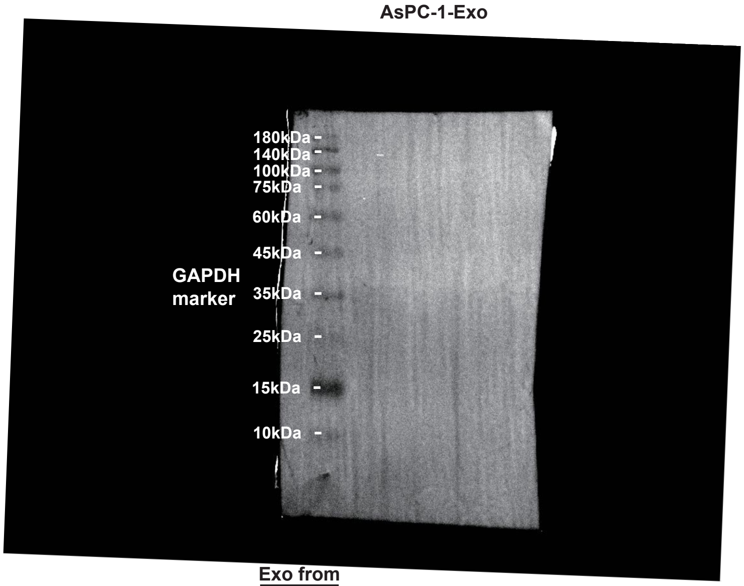


| Exo from | | | | | |
|---|---|---|---|---|---|
| sh-NC | - | + | - | - | - |
| sh-Chemo | - | - | + | - | + |
| OE-CCT6A | - | - | - | + | + |

Repeat#3

AsPC-1-Exo

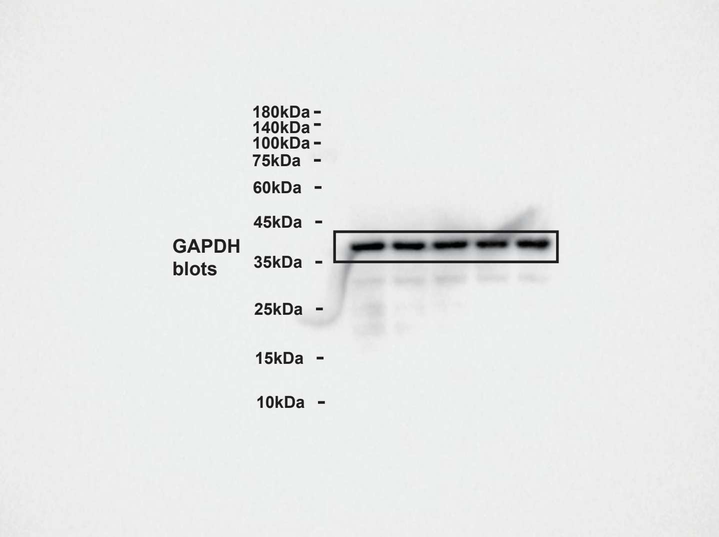


| Exo from | | | | | |
|---|---|---|---|---|---|
| sh-NC | - | + | - | - | - |
| sh-Chemo | - | - | + | - | + |
| OE-CCT6A | - | - | - | + | + |

AsPC-1-Exo

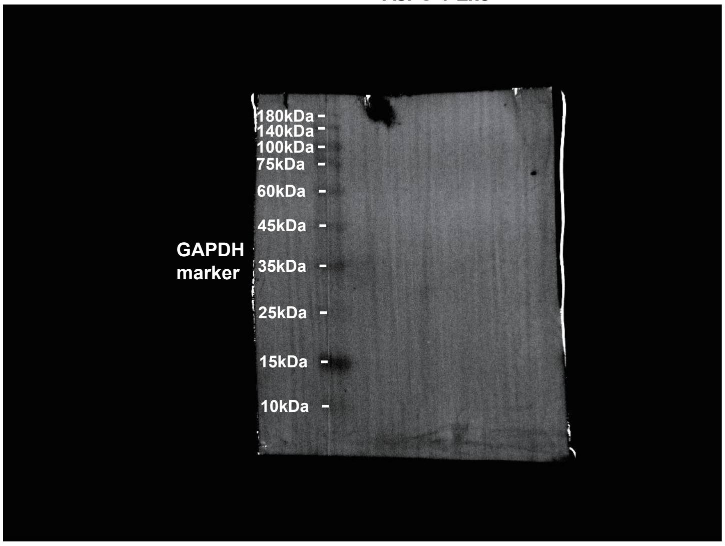


| Exo from | | | | | |
|---|---|---|---|---|---|
| sh-NC | - | + | - | - | - |
| sh-Chemo | - | - | + | - | + |
| OE-CCT6A | - | - | - | + | + |

**Fig. 6B**

**Repeat#1**

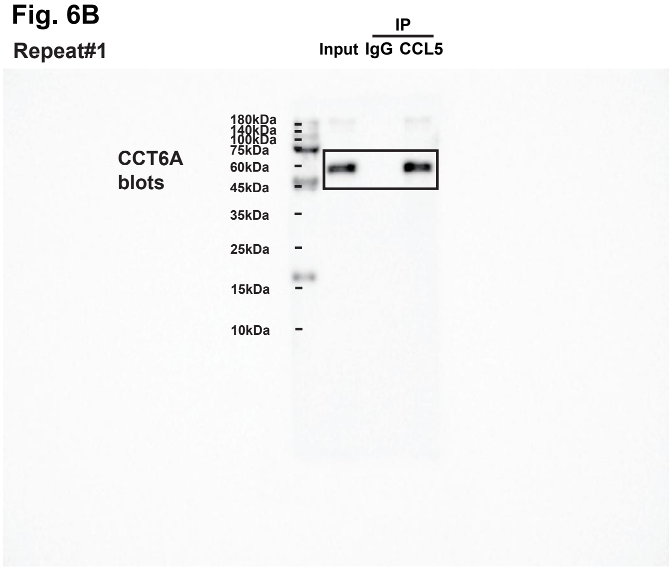


**AsPC-1-Exo**

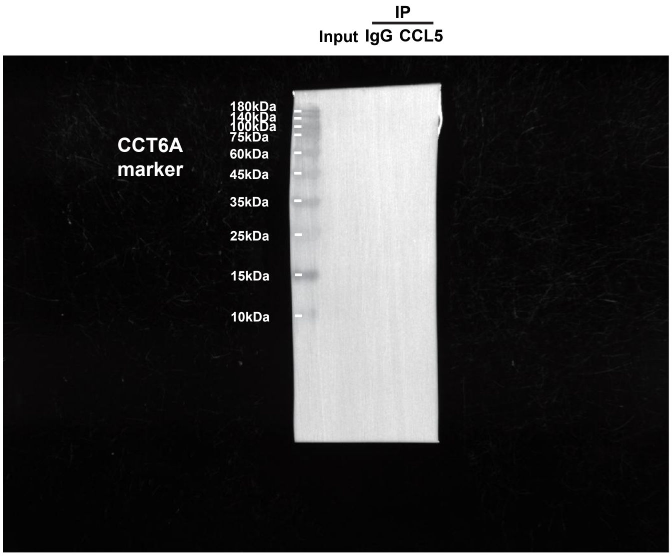


**AsPC-1-Exo**

**Repeat#2**

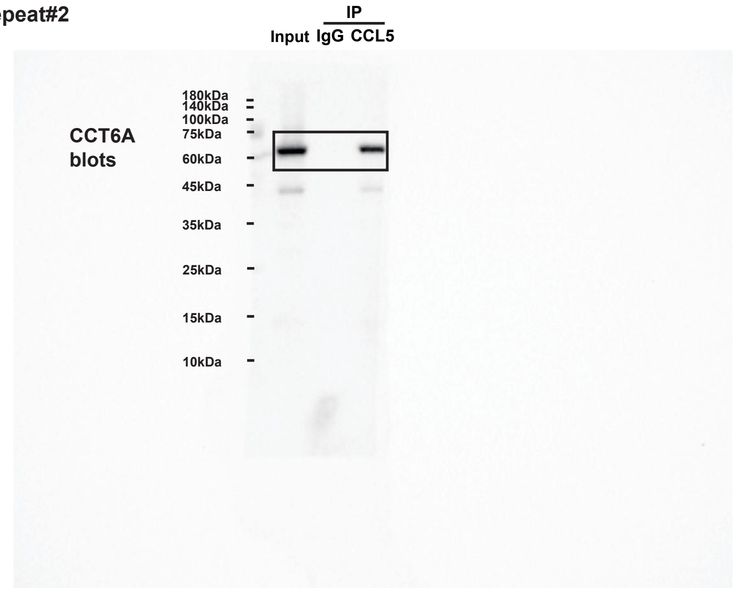


**AsPC-1-Exo**

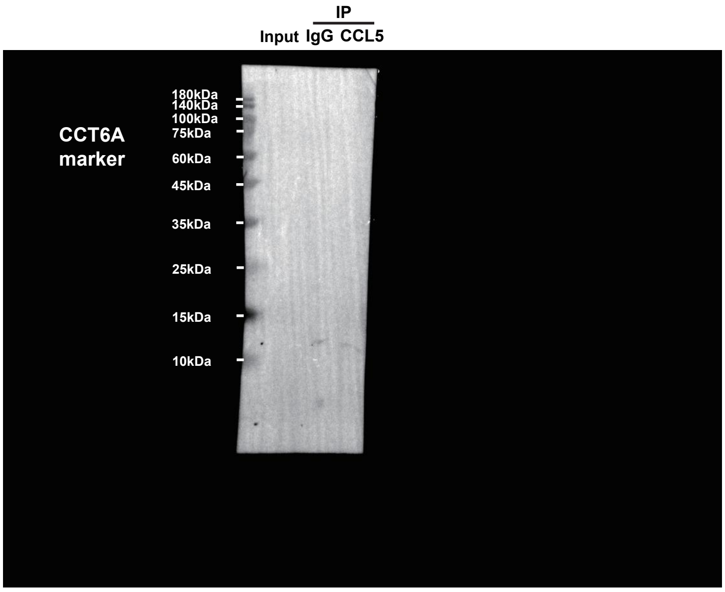


**AsPC-1-Exo**

**Repeat#3**

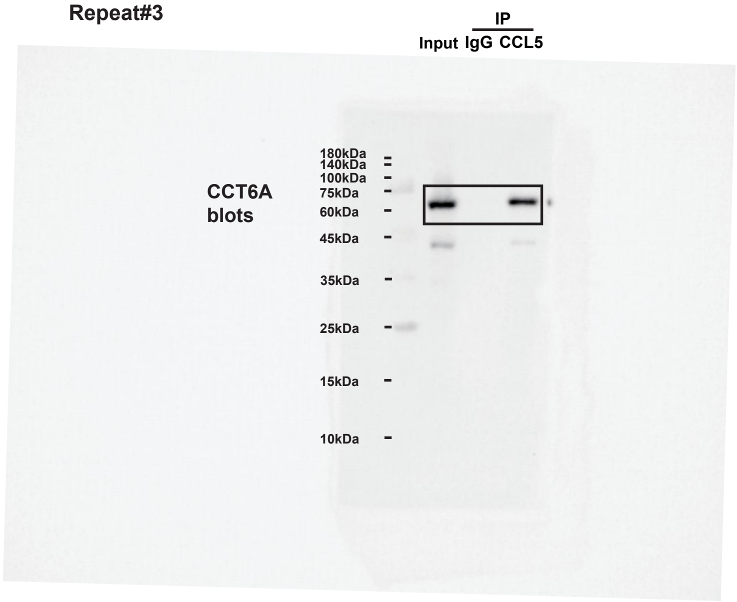


**AsPC-1-Exo**

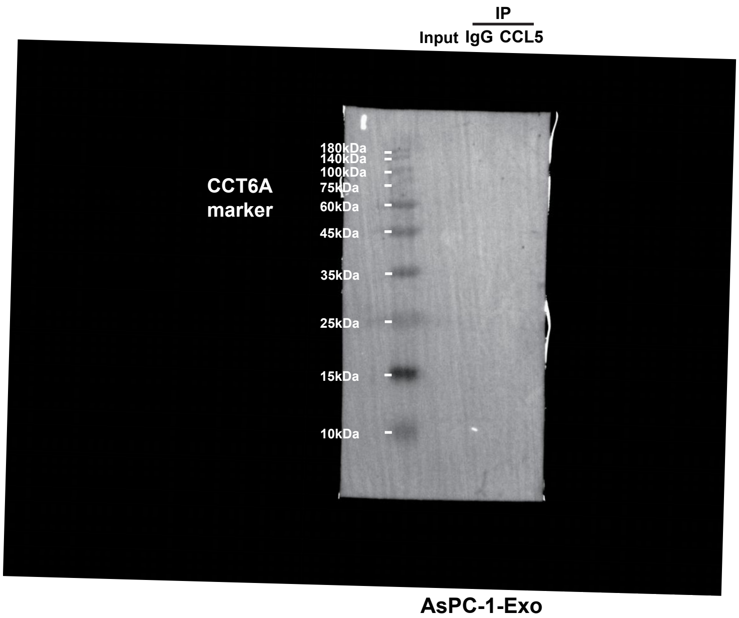


**AsPC-1-Exo**

Repeat#1

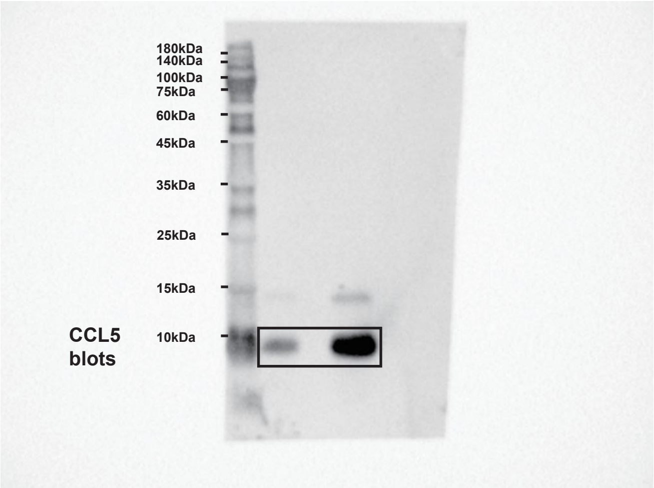


AsPC-1-Exo

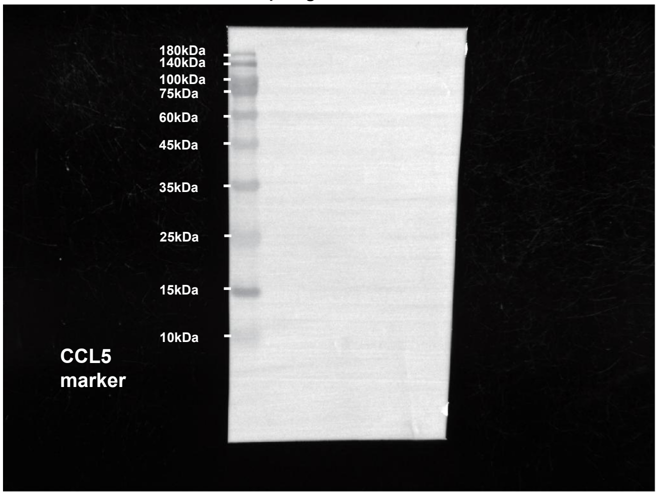


AsPC-1-Exo

Repeat#2

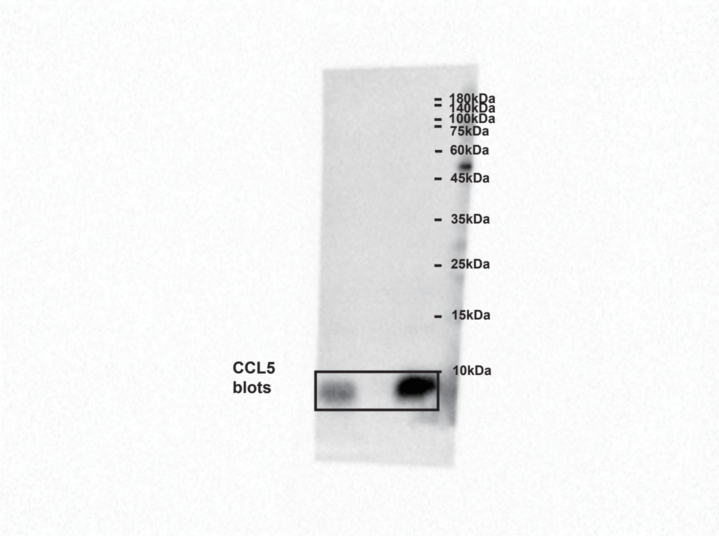


AsPC-1-Exo

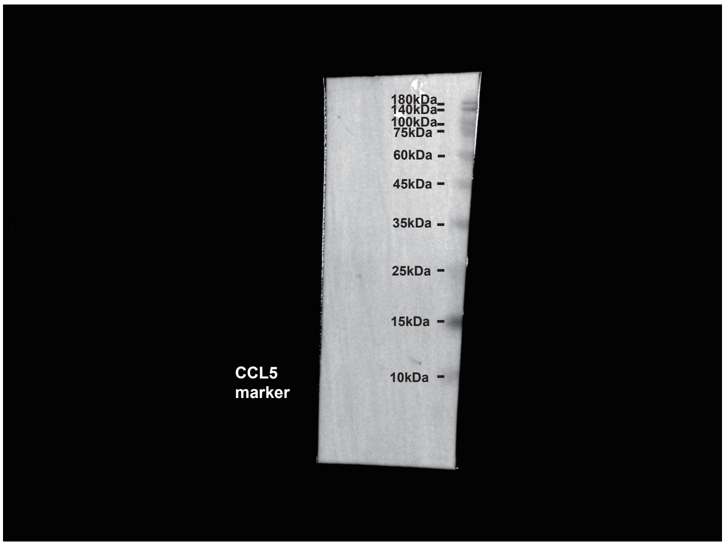


AsPC-1-Exo

Repeat#2

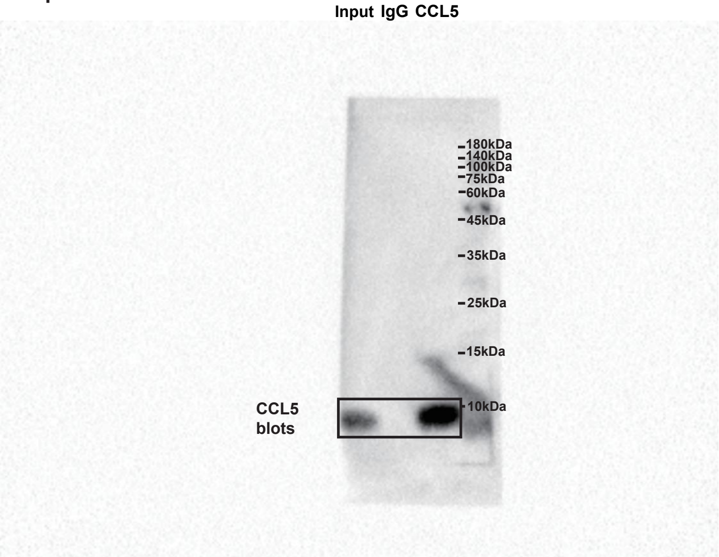


AsPC-1-Exo

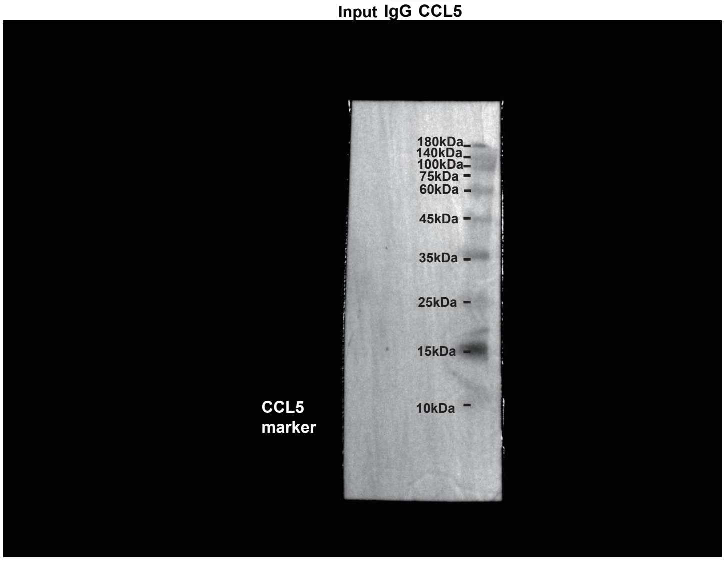


AsPC-1-Exo

**Repeat#1**

IP
Input IgG CCT6A

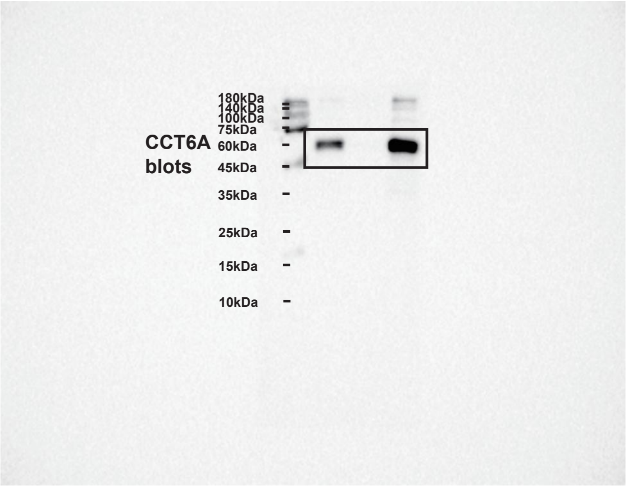


AsPC-1-Exo

IP
Input IgG CCT6A

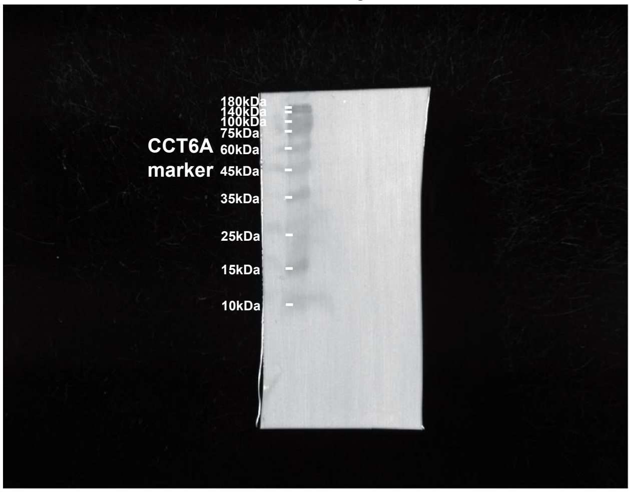


AsPC-1-Exo

**Repeat#2**

IP
Input IgG CCT6A

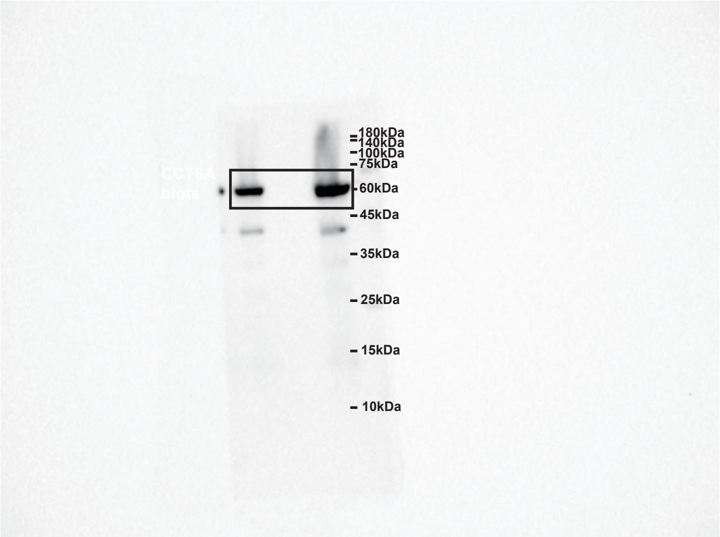


AsPC-1-Exo

IP
Input IgG CCT6A

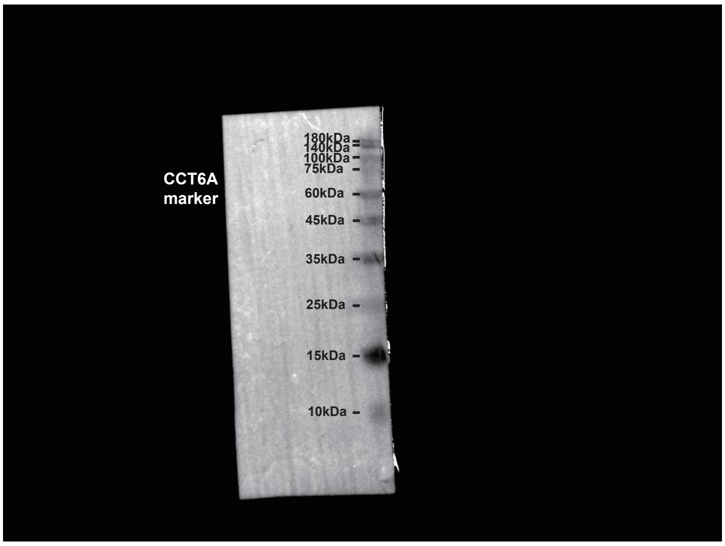


AsPC-1-Exo

**Repeat#3**

IP
Input IgG CCT6A

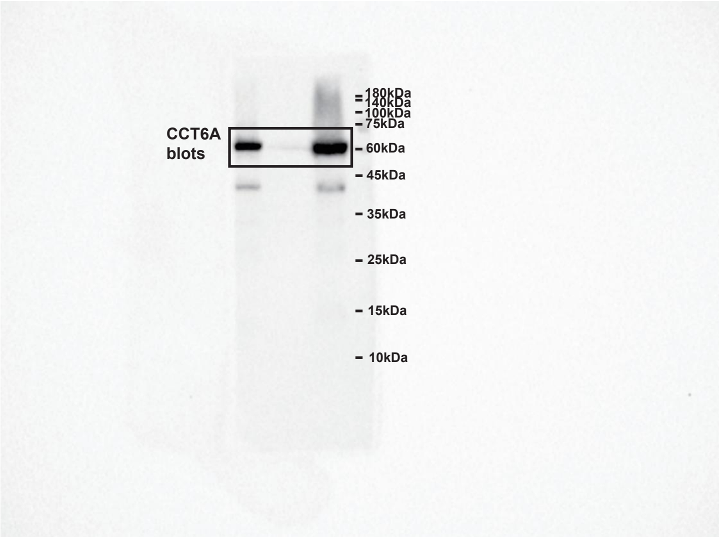


AsPC-1-Exo

IP
Input IgG CCT6A

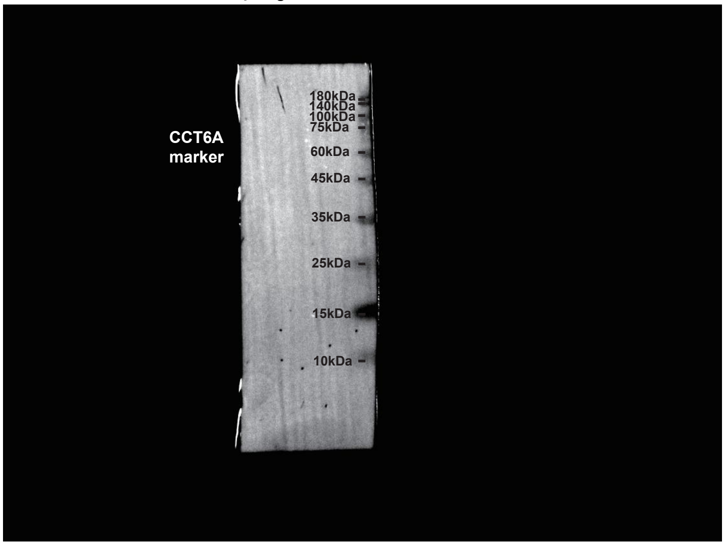


AsPC-1-Exo

Repeat#1



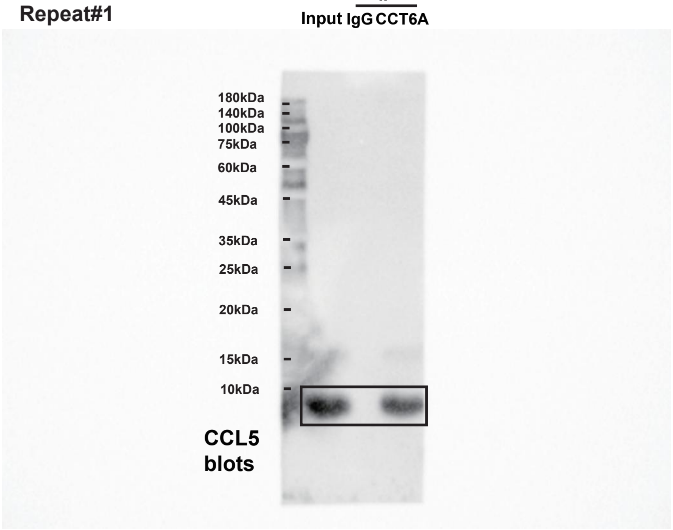


AsPC-1-Exo



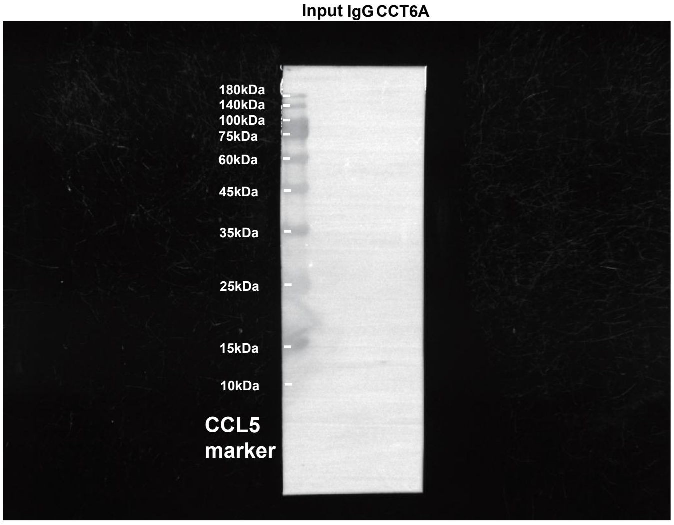


AsPC-1-Exo

Repeat#2



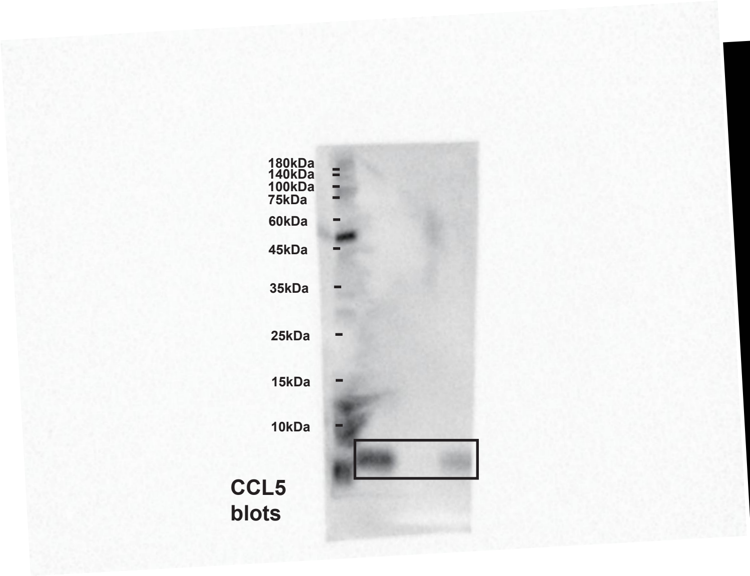


AsPC-1-Exo



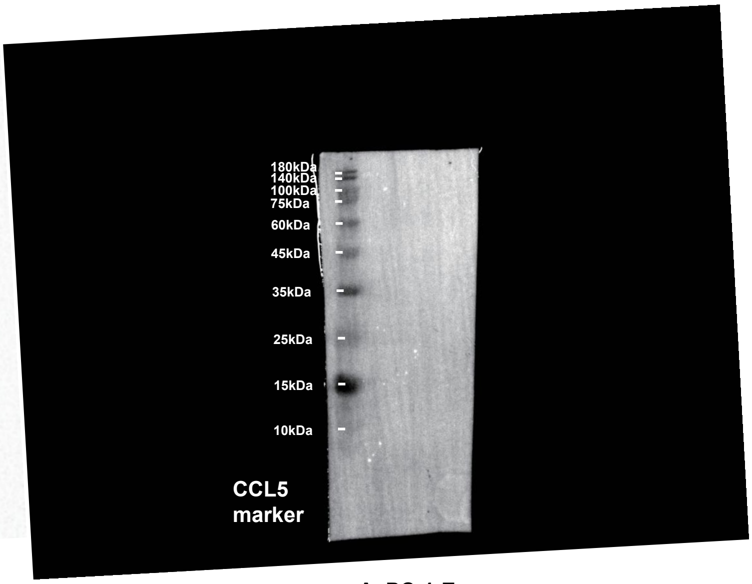


AsPC-1-Exo

Repeat#3



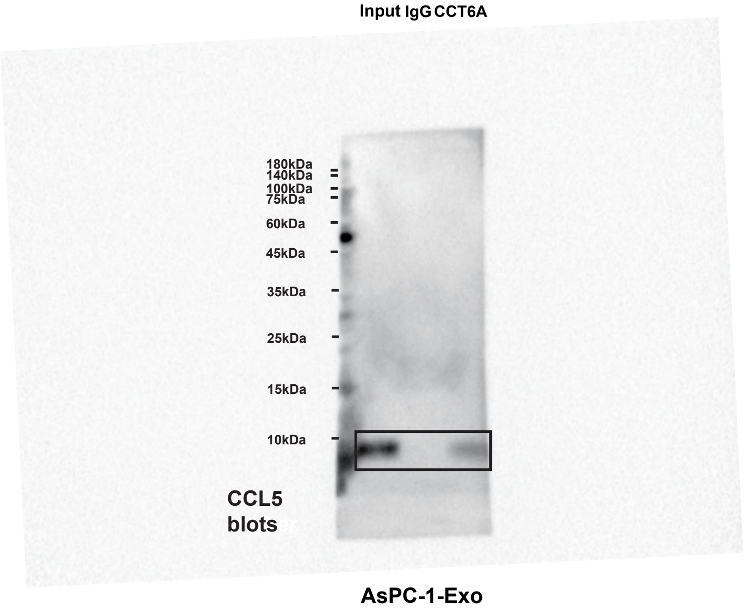


AsPC-1-Exo



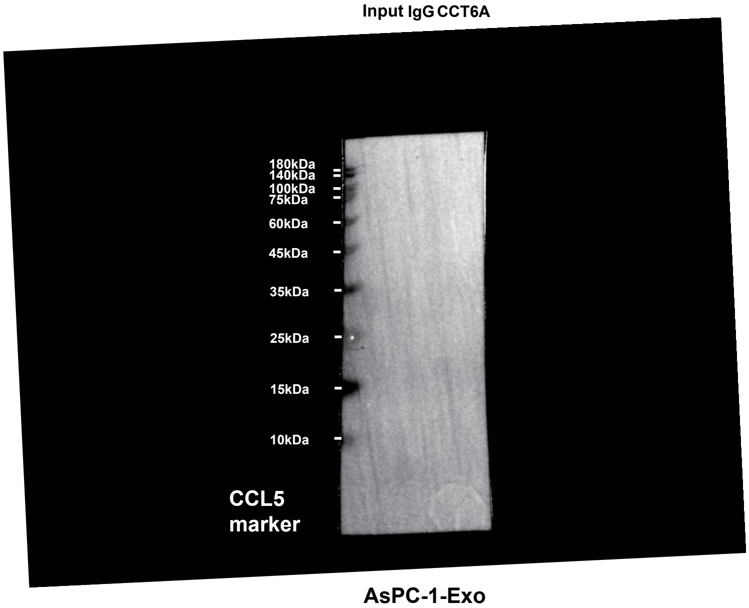


AsPC-1-Exo

Repeat#1

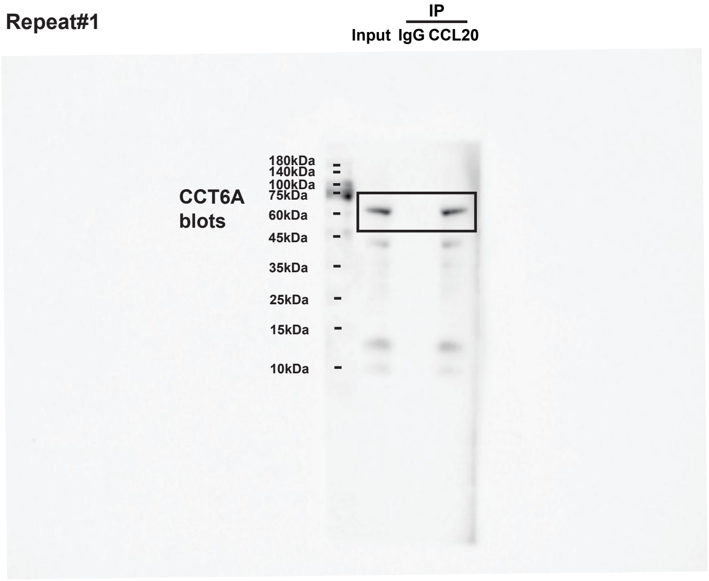


AsPC-1-Exo

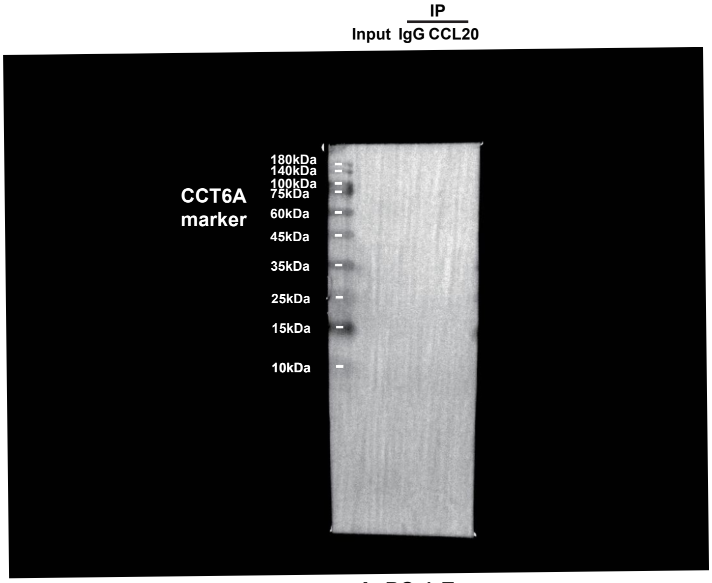


AsPC-1-Exo

Repeat#2

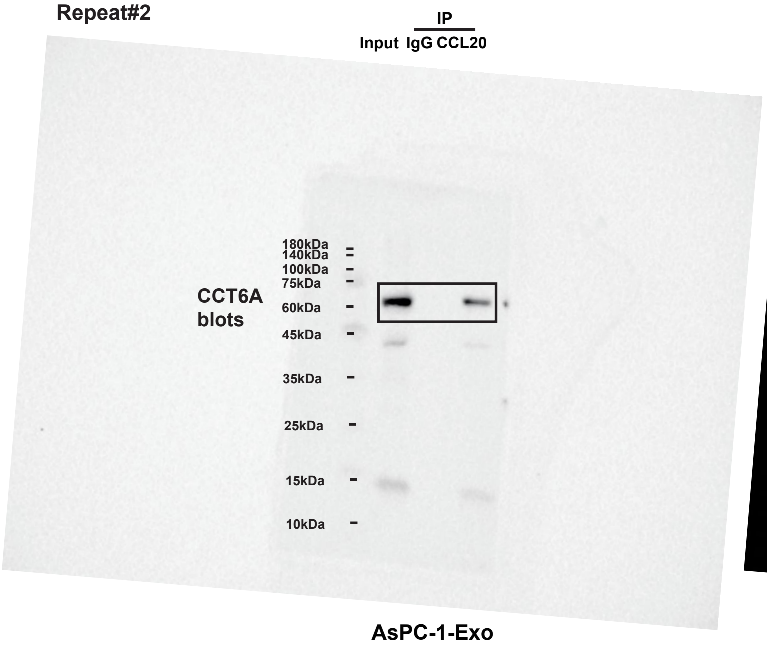


AsPC-1-Exo

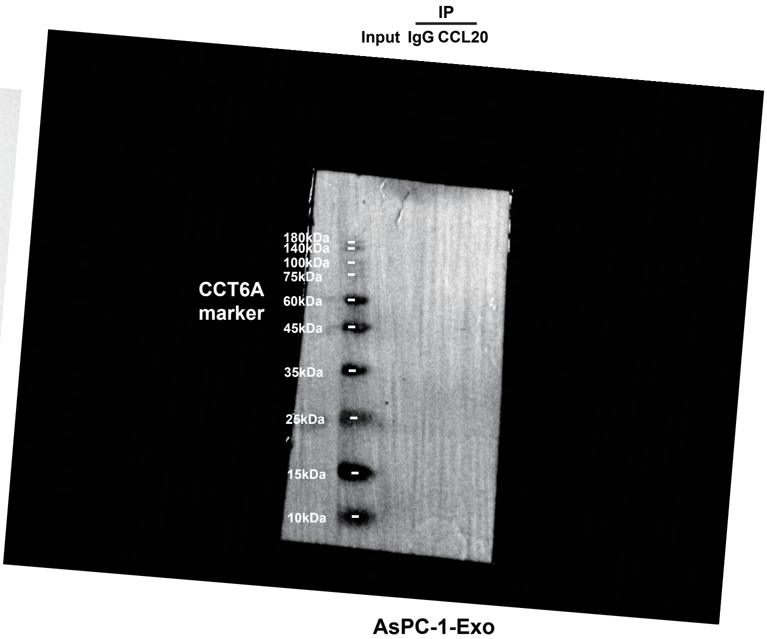


AsPC-1-Exo

Repeat#3

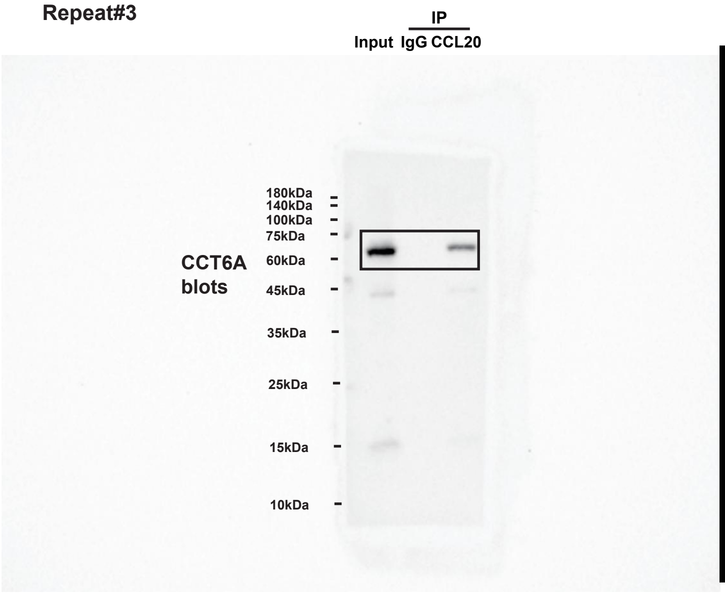


AsPC-1-Exo

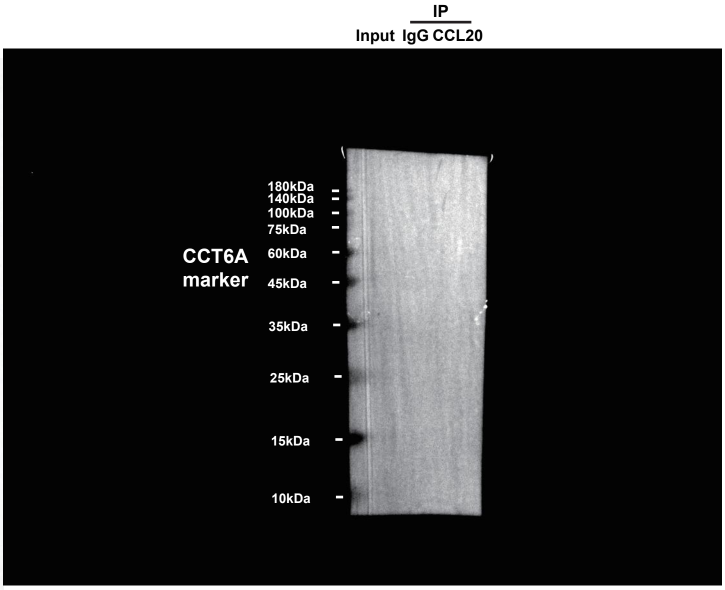


AsPC-1-Exo

Repeat#1

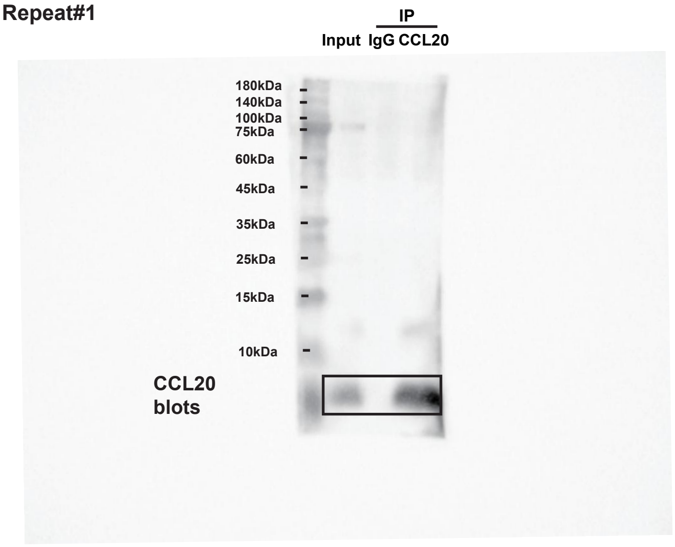


AsPC-1-Exo

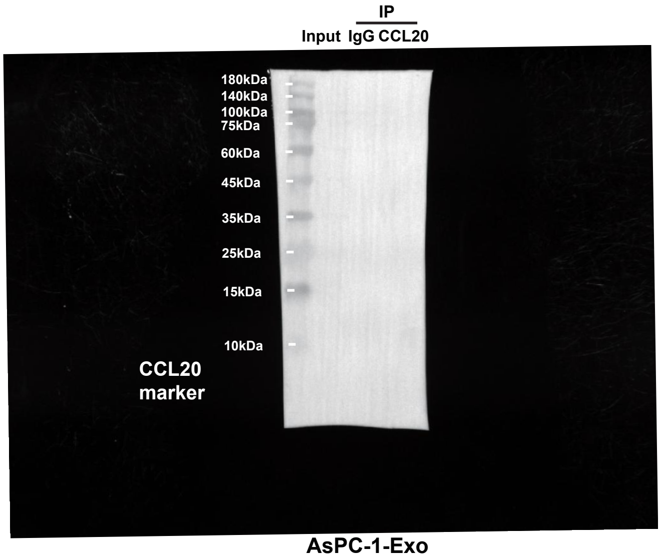


AsPC-1-Exo

Repeat#2

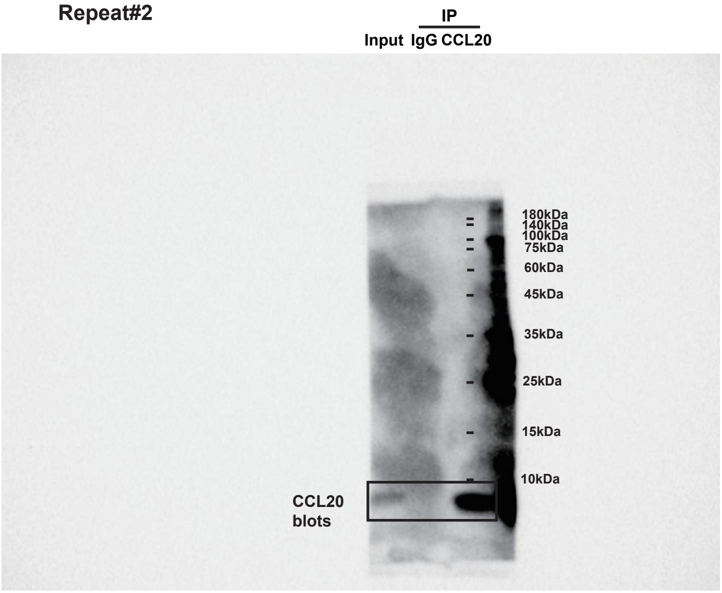


AsPC-1-Exo

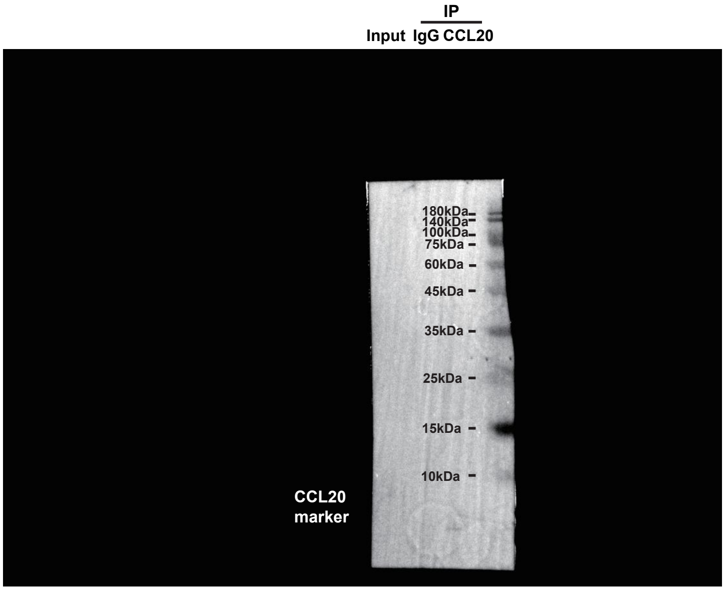


AsPC-1-Exo

Repeat#3

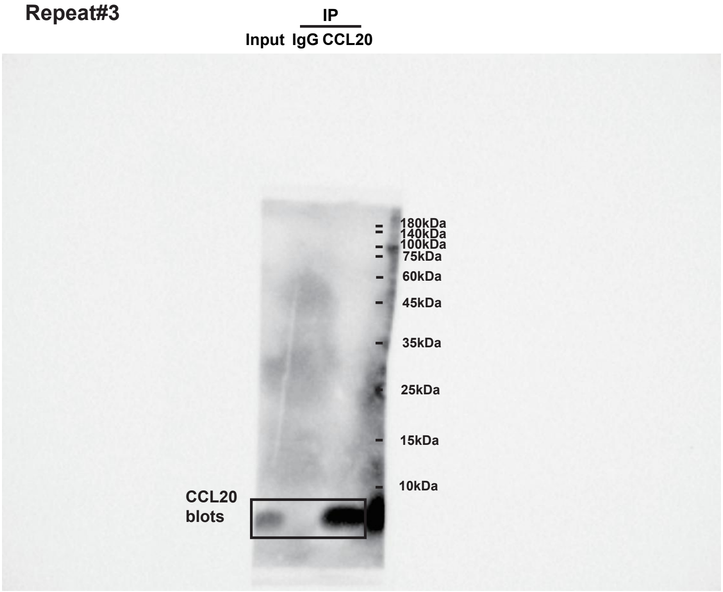


AsPC-1-Exo

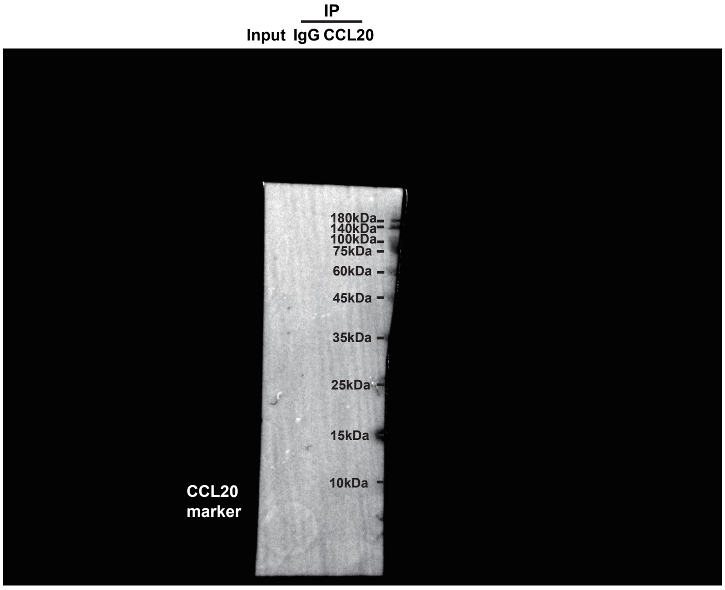


AsPC-1-Exo

Repeat#1

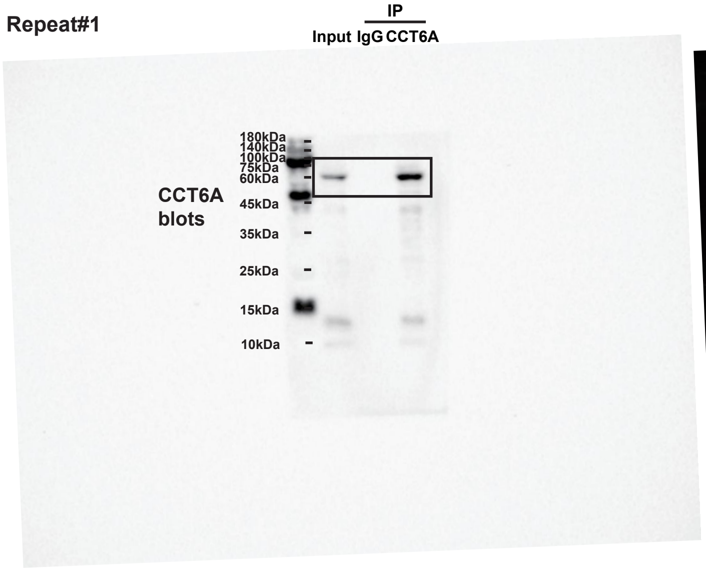


AsPC-1-Exo

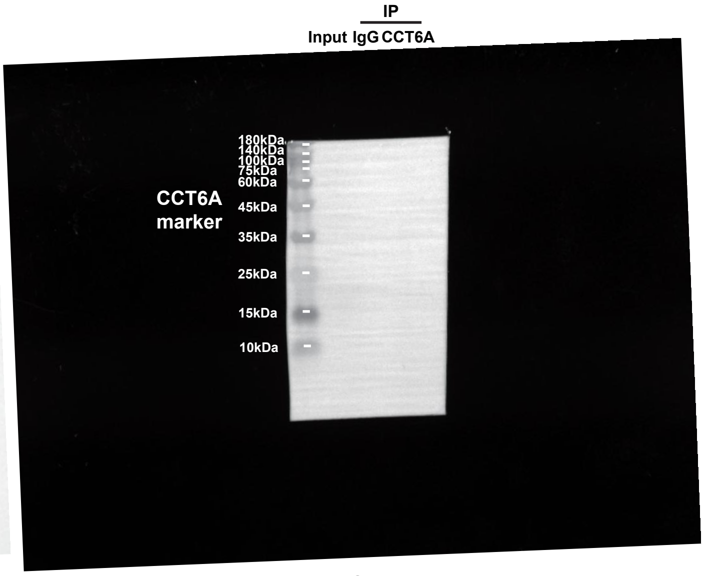


AsPC-1-Exo

Repeat#2

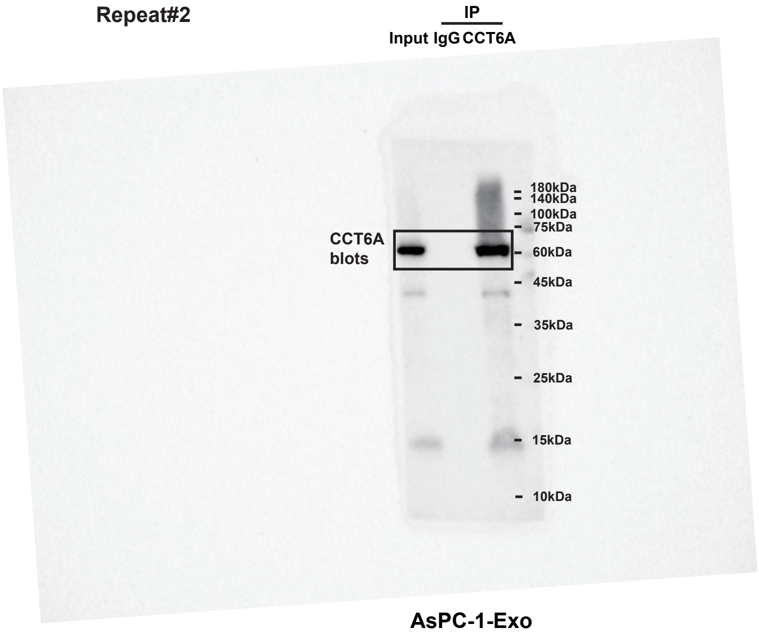


AsPC-1-Exo

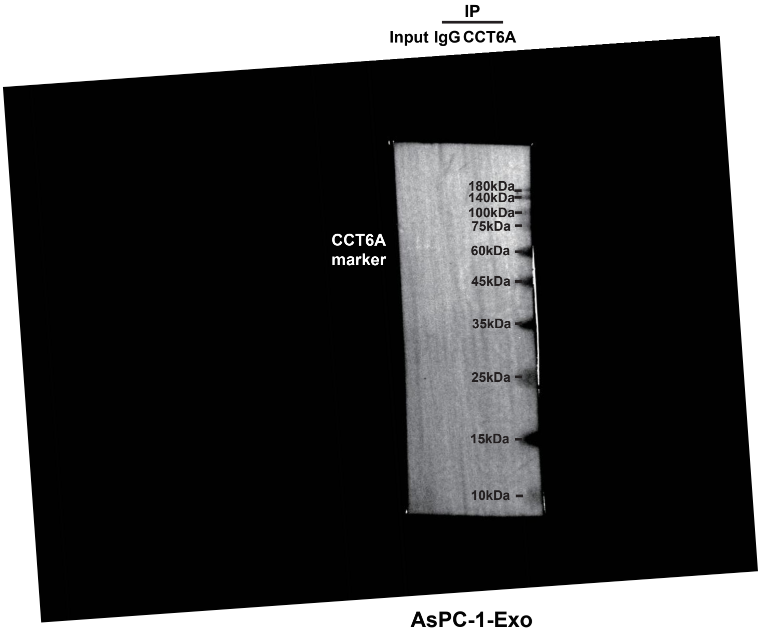


AsPC-1-Exo

Repeat#3

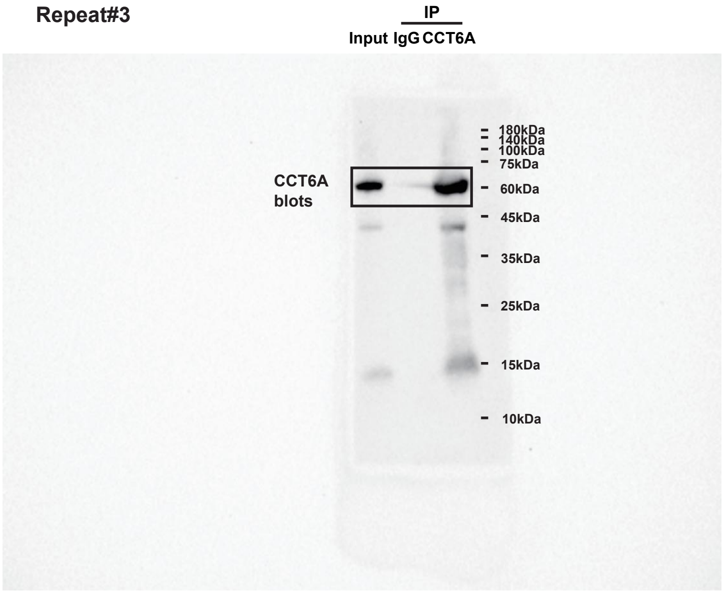


AsPC-1-Exo

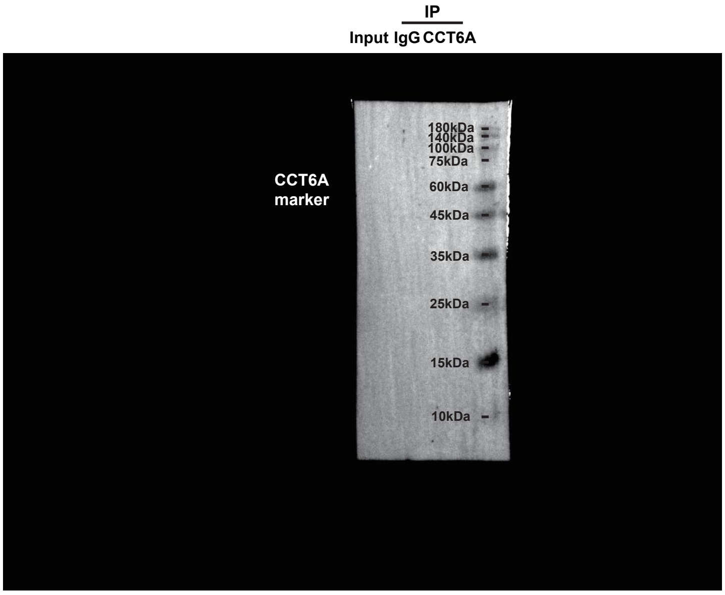


AsPC-1-Exo

Repeat#1

IP
Input IgG CCT6A

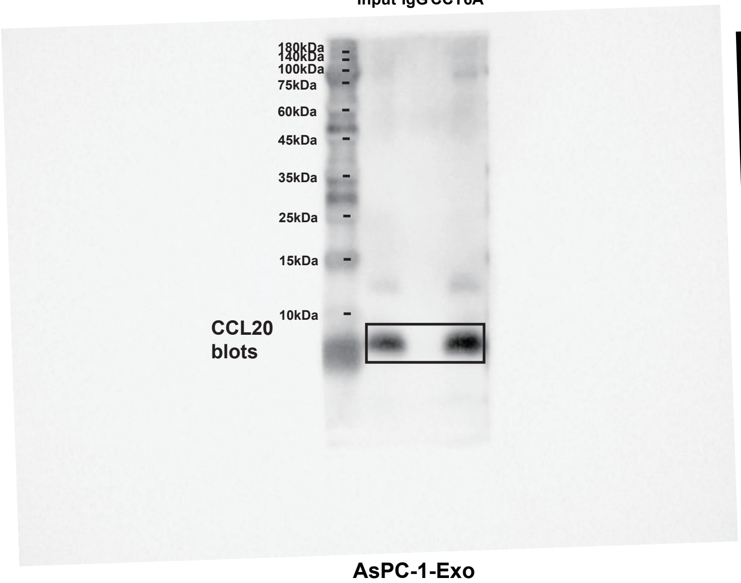


AsPC-1-Exo

IP
Input IgG CCT6A

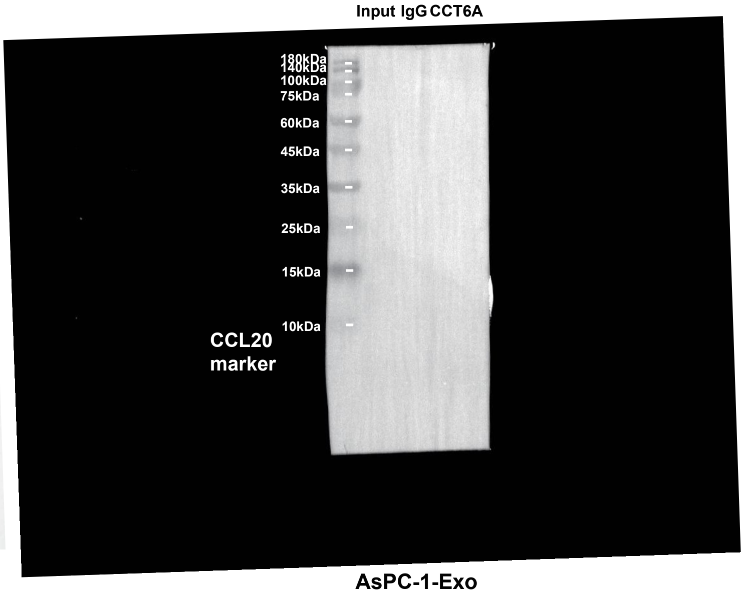


AsPC-1-Exo

Repeat#2

IP
Input IgG CCT6A

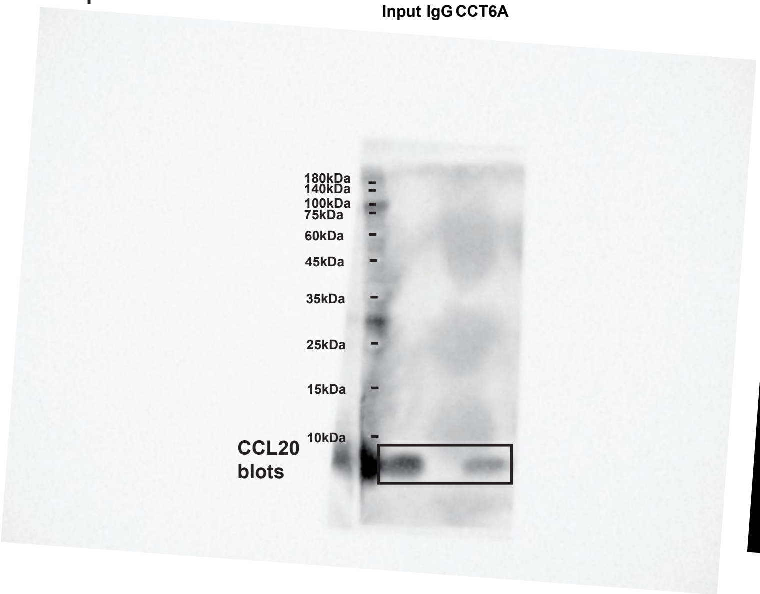


AsPC-1-Exo

IP
Input IgG CCT6A

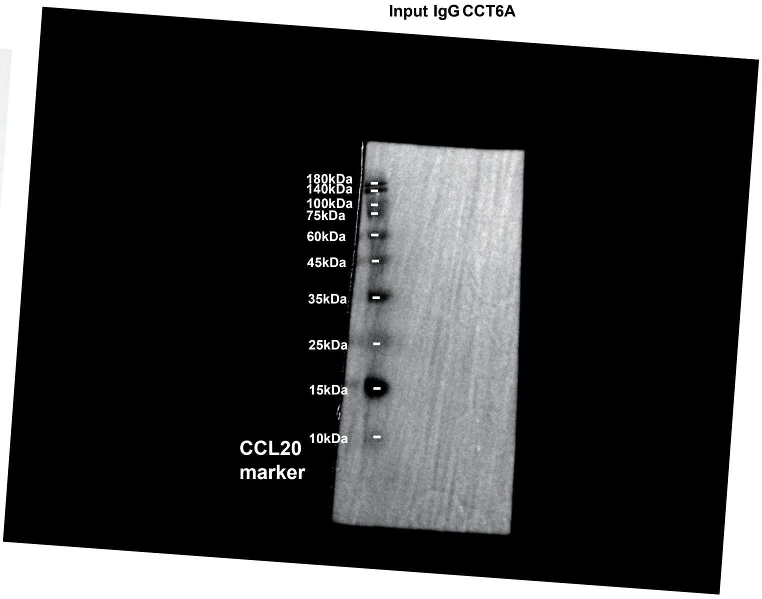


AsPC-1-Exo

Repeat#3

IP
Input IgG CCT6A

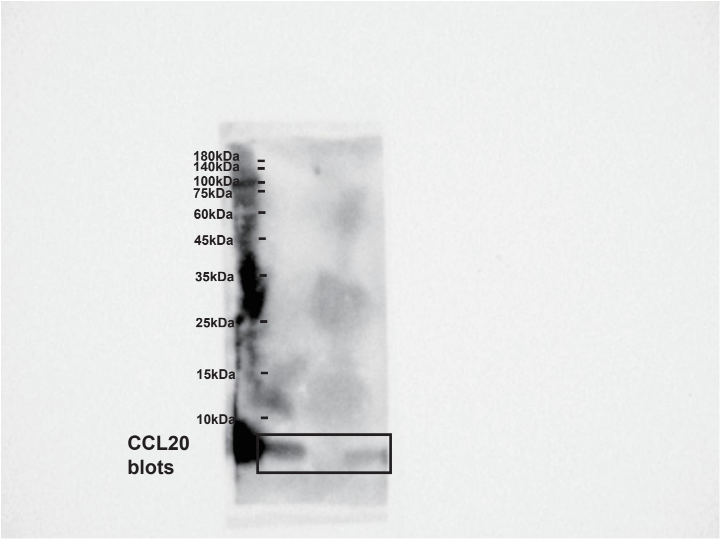


AsPC-1-Exo

IP
Input IgG CCT6A

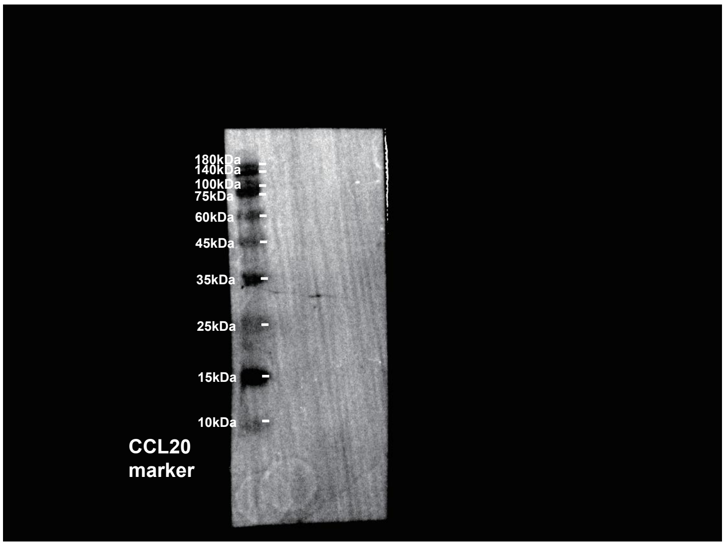


AsPC-1-Exo

Repeat#1

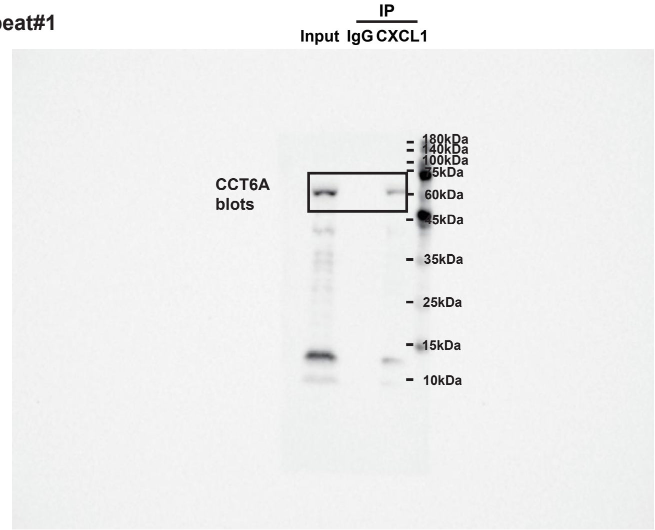


AsPC-1-Exo

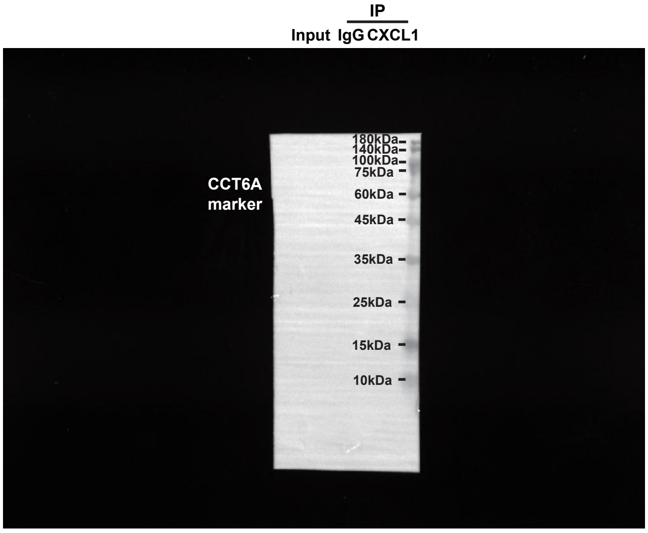


AsPC-1-Exo

Repeat#2

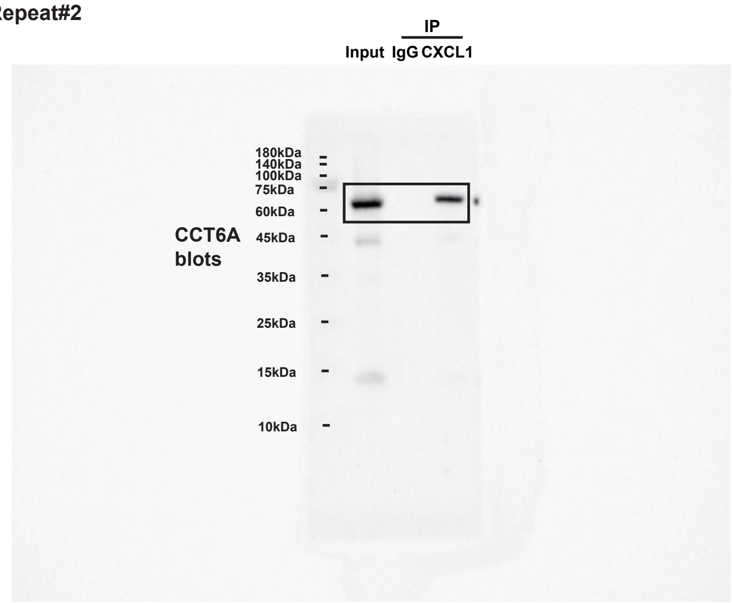


AsPC-1-Exo

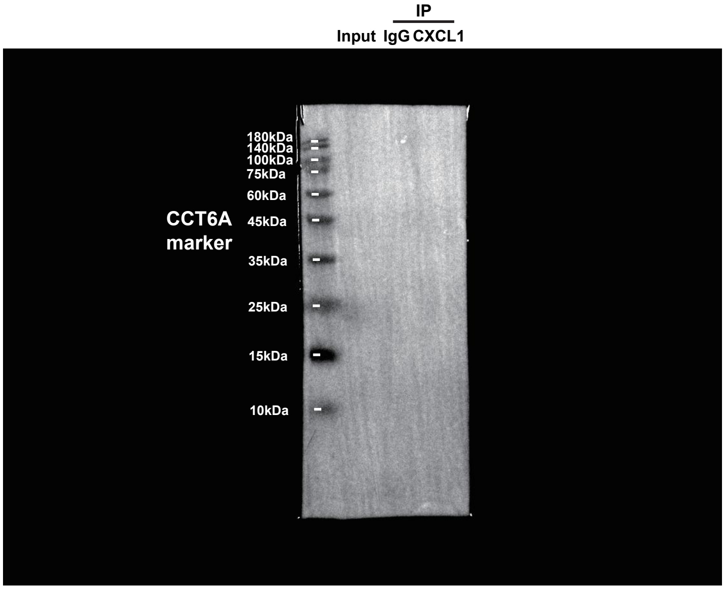


AsPC-1-Exo

Repeat#3

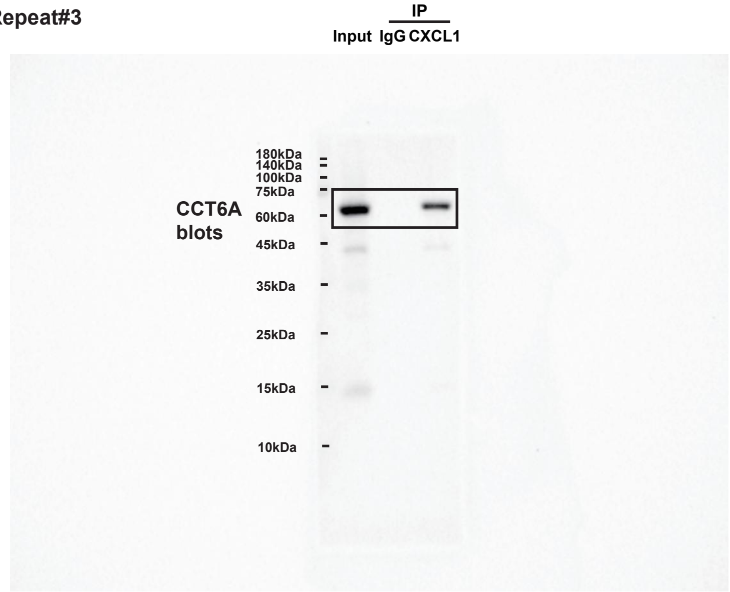


AsPC-1-Exo

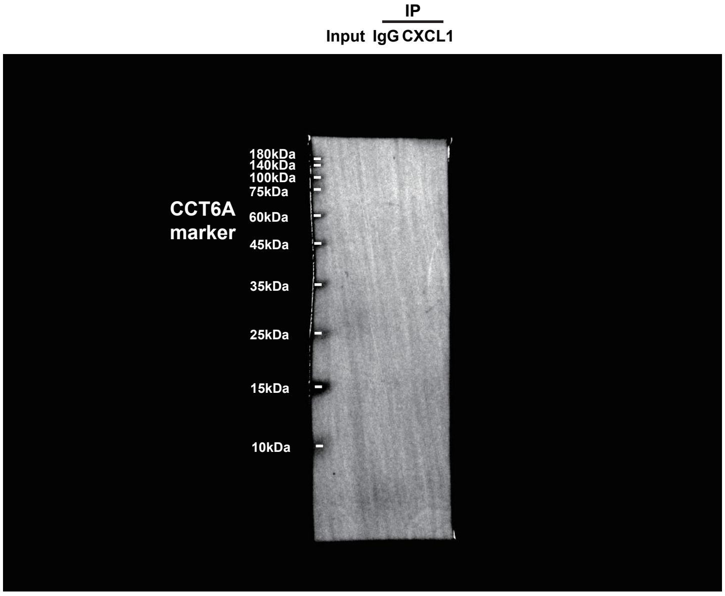


AsPC-1-Exo

Repeat#1

IP
Input IgG CXCL1

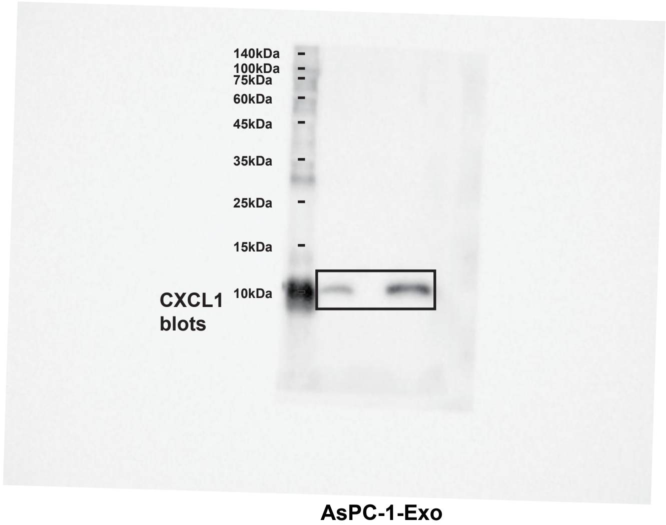


AsPC-1-Exo

IP
Input IgG CXCL1

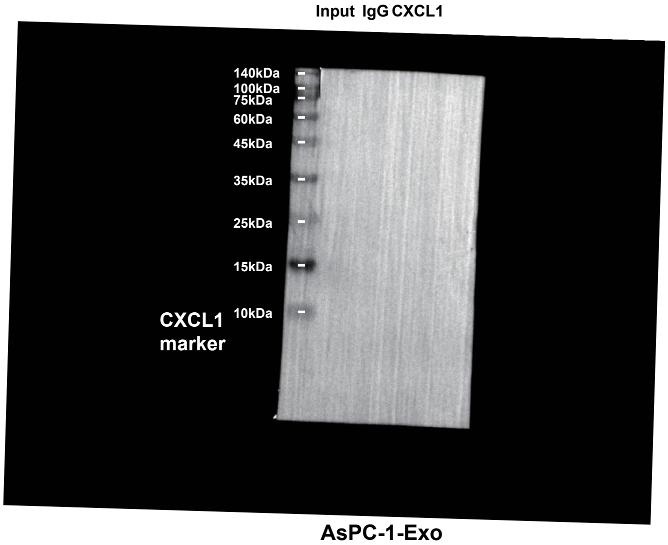


AsPC-1-Exo

Repeat#2

IP
Input IgG CXCL1

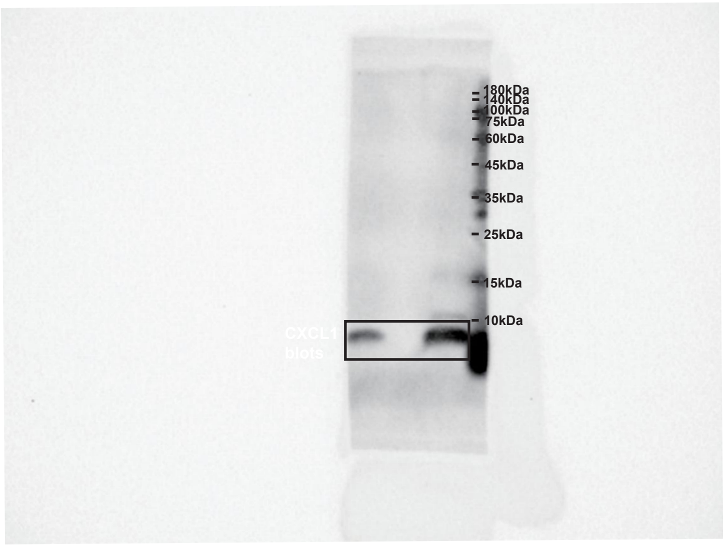


AsPC-1-Exo

IP
Input IgG CXCL1

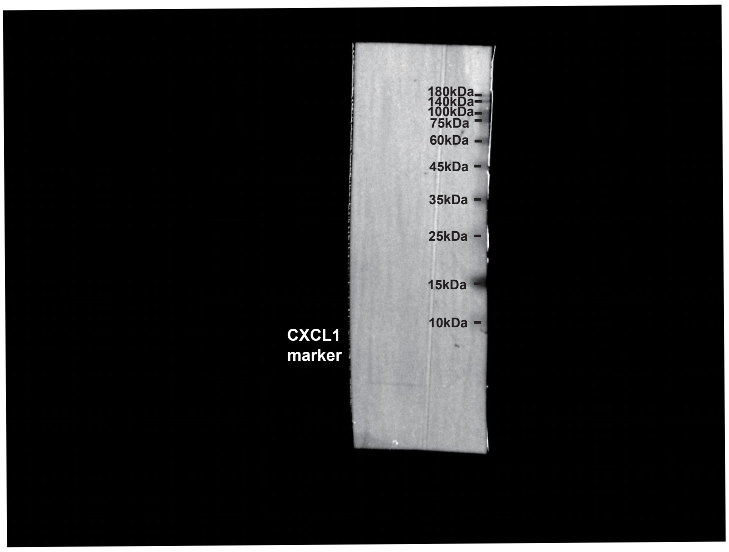


AsPC-1-Exo

Repeat#3

IP
Input IgG CXCL1

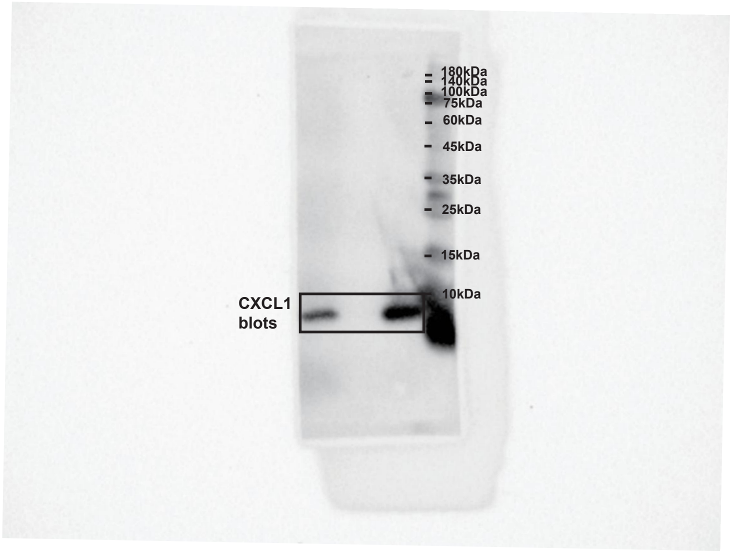


AsPC-1-Exo

IP
Input IgG CXCL1

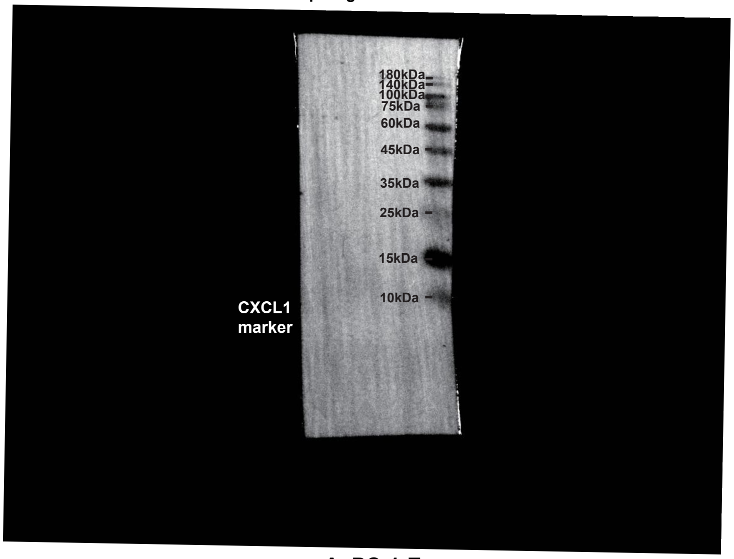


AsPC-1-Exo

Repeat#1

IP
Input IgG CCT6A

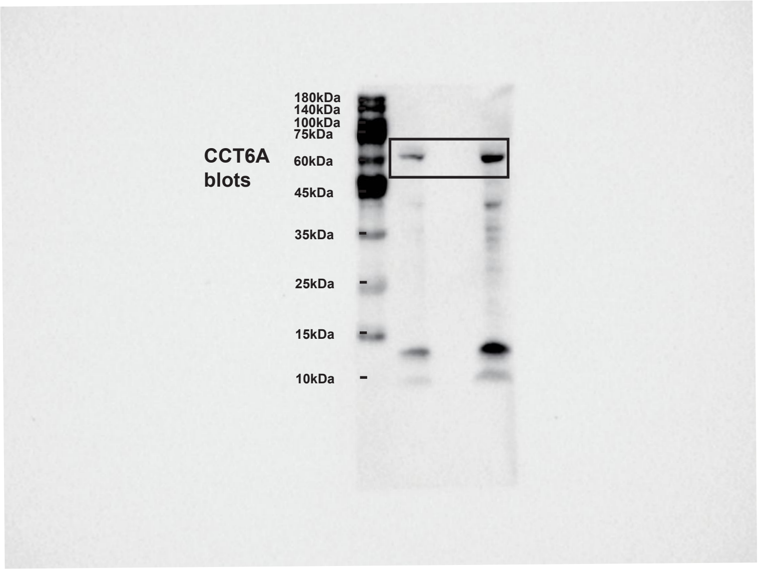


AsPC-1-Exo

IP
Input IgG CCT6A

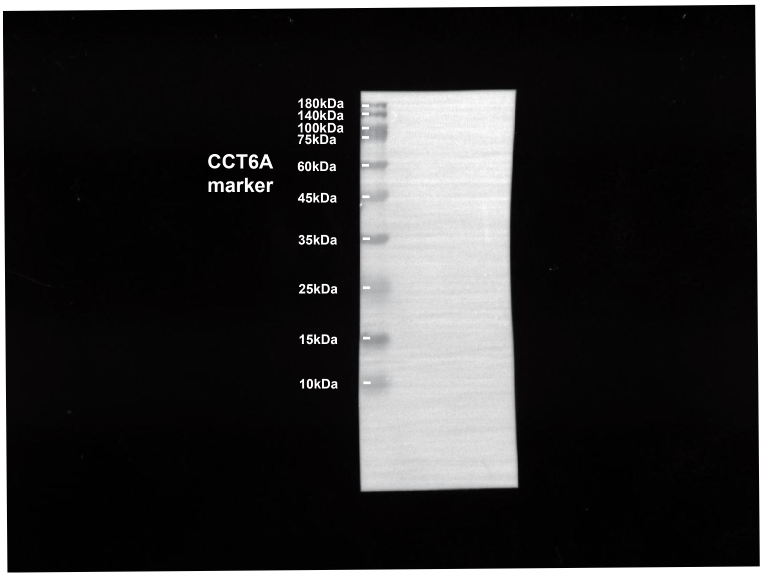


AsPC-1-Exo

Repeat#2

IP
Input IgG CCT6A

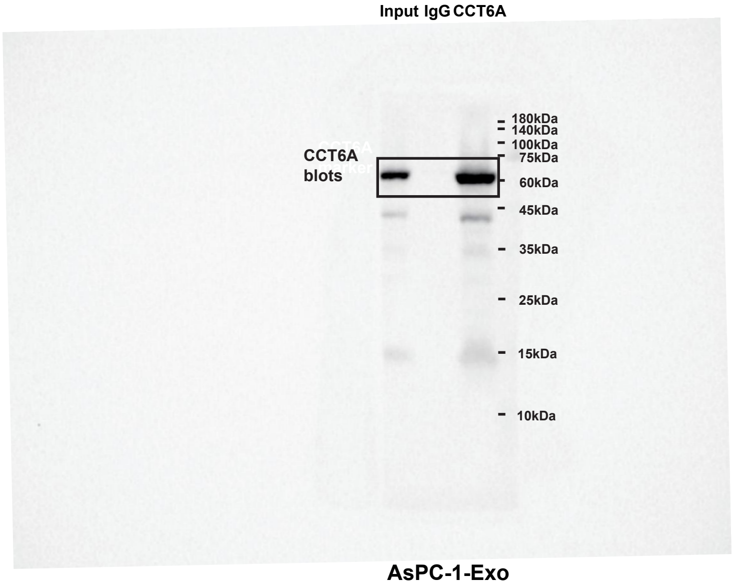


AsPC-1-Exo

IP
Input IgG CCT6A

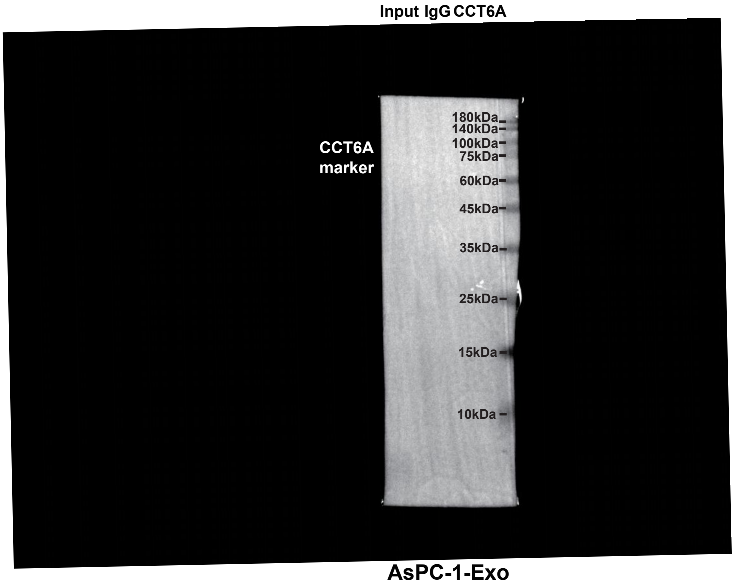


AsPC-1-Exo

Repeat#3

IP
Input IgG CCT6A

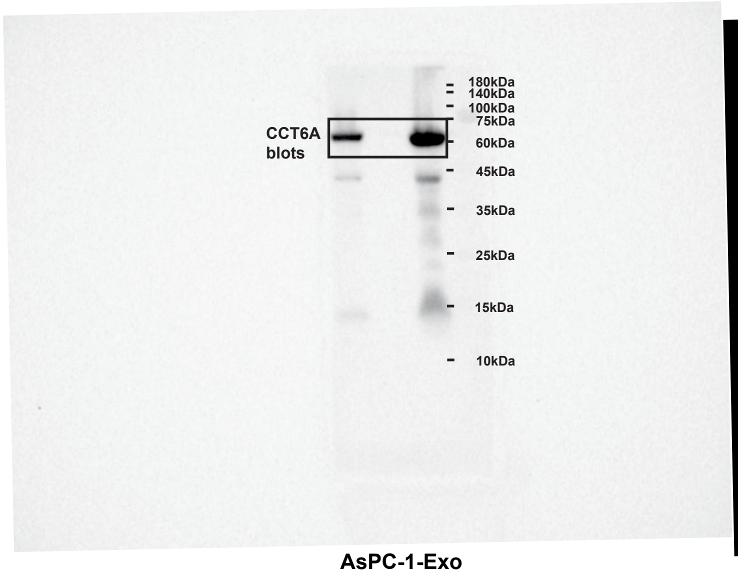


AsPC-1-Exo

IP
Input IgG CCT6A

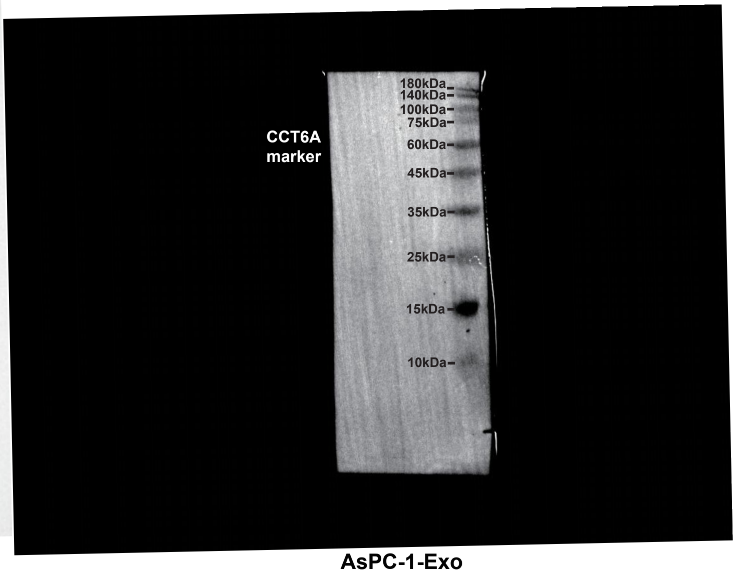


AsPC-1-Exo

**Repeat#1**

IP

Input IgG CCT6A

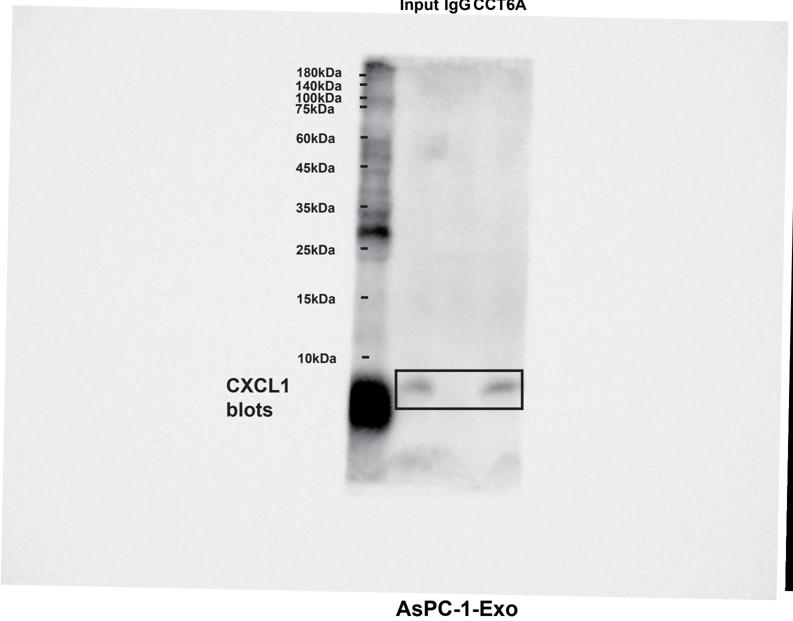


AsPC-1-Exo

IP

Input IgG CCT6A

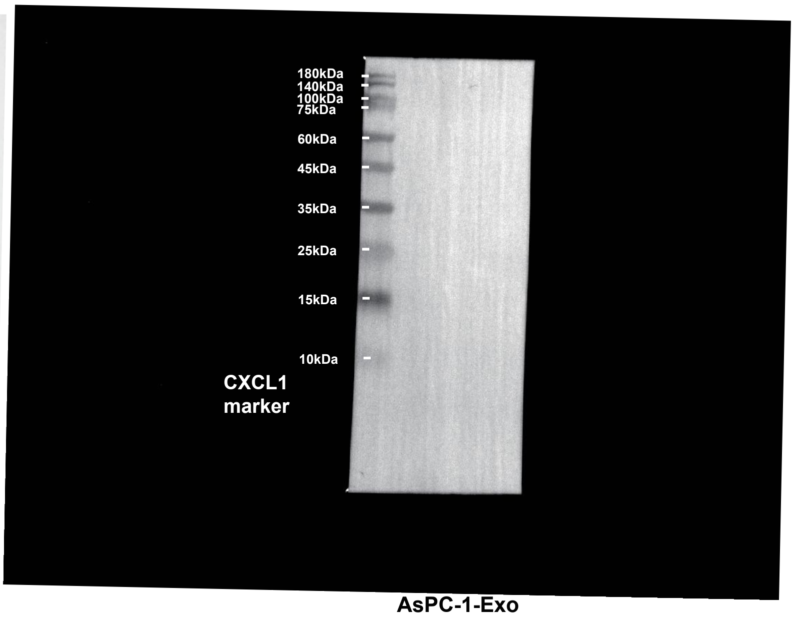


AsPC-1-Exo

**Repeat#2**

IP

Input IgG CCT6A

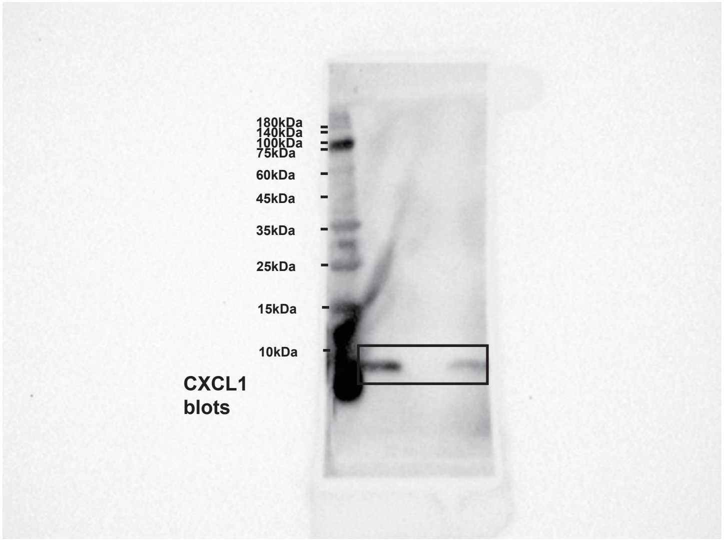


AsPC-1-Exo

IP

Input IgG CCT6A

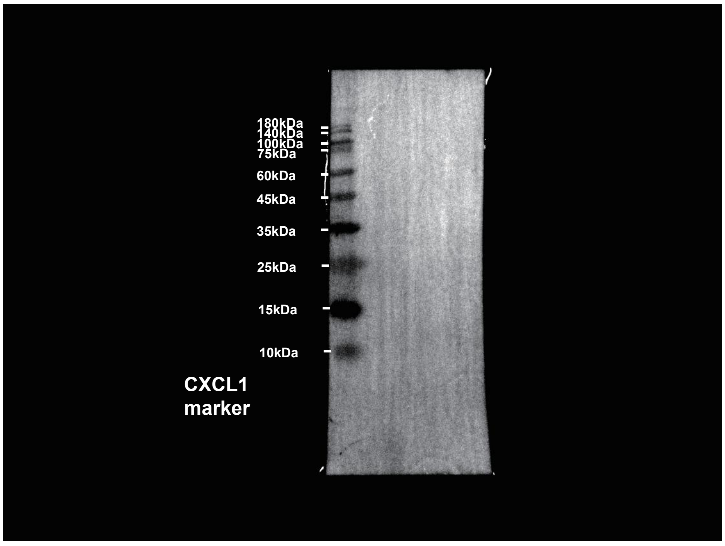


AsPC-1-Exo

**Repeat#3**

IP

Input IgG CCT6A

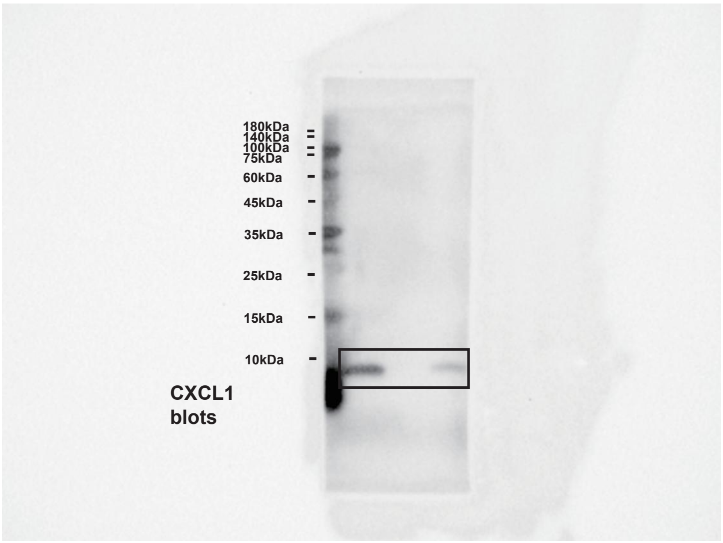


AsPC-1-Exo

IP

Input IgG CCT6A

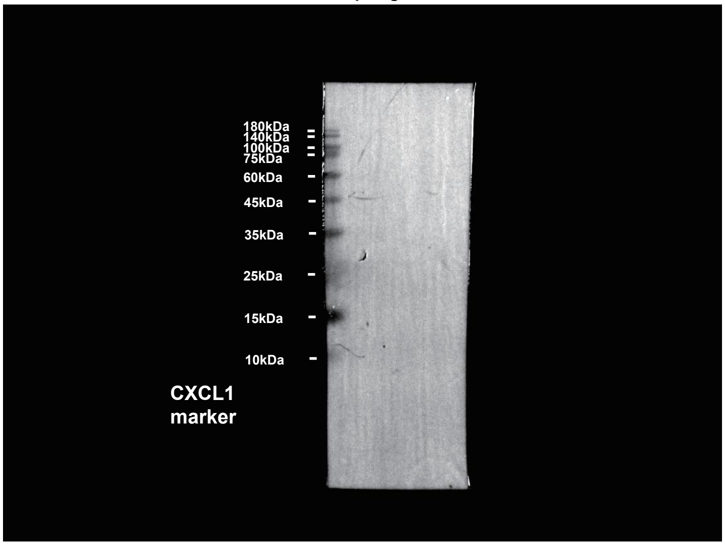


AsPC-1-Exo

Repeat#1

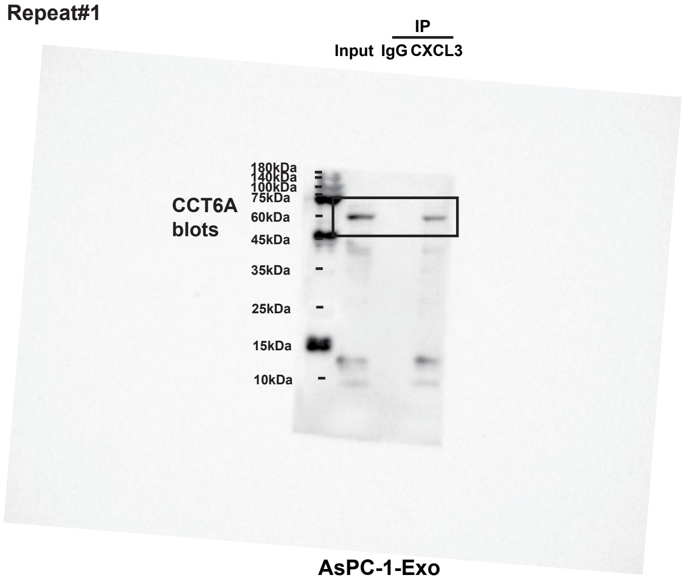


AsPC-1-Exo

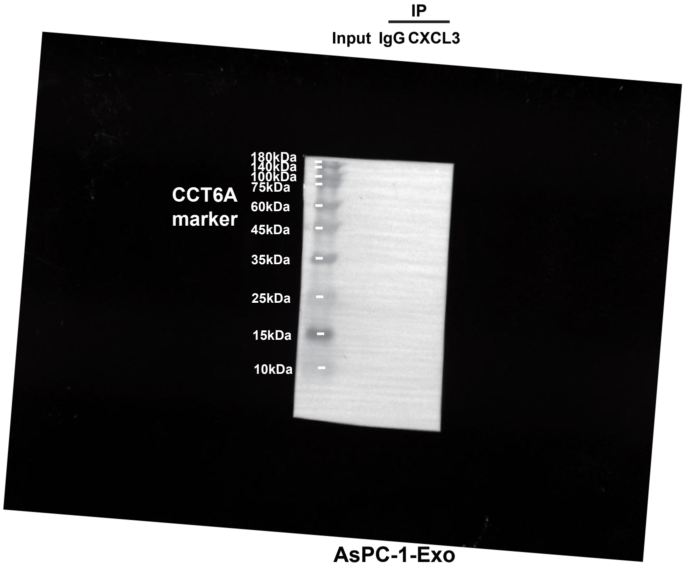


AsPC-1-Exo

Repeat#2

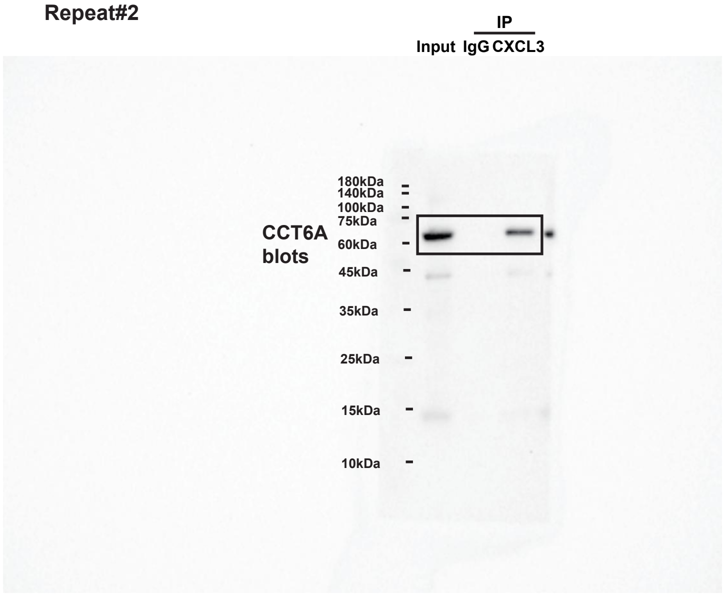


AsPC-1-Exo

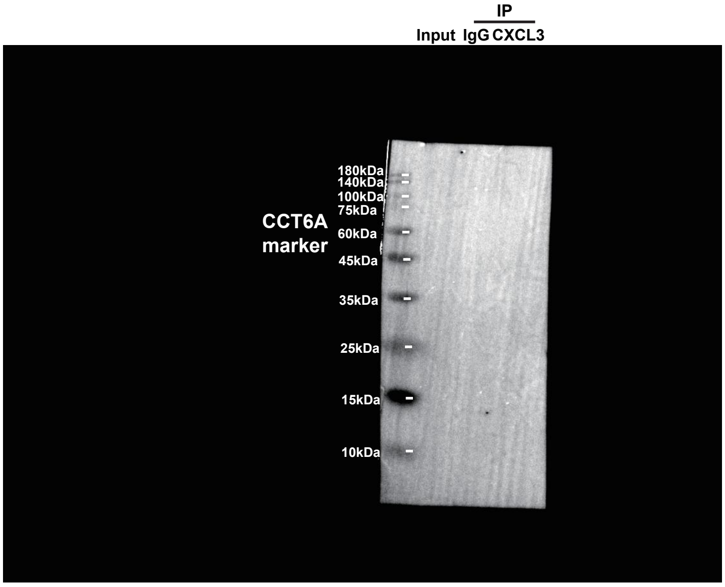


AsPC-1-Exo

Repeat#3

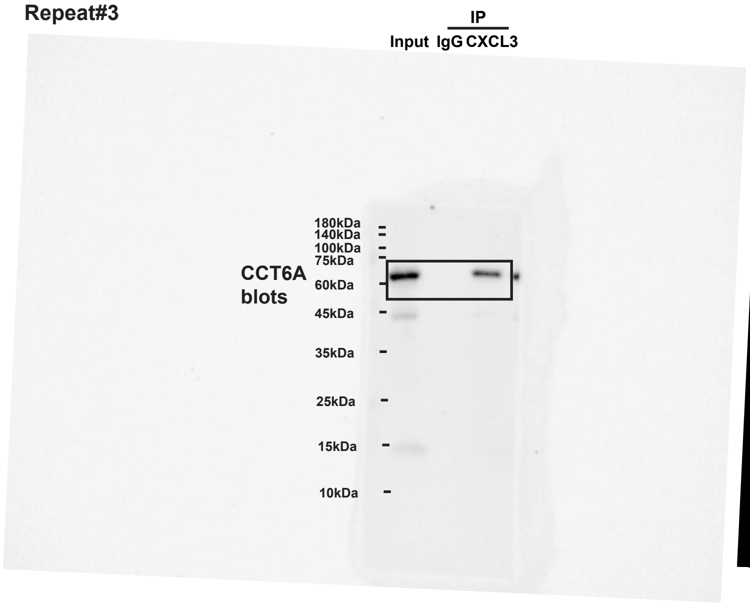


AsPC-1-Exo

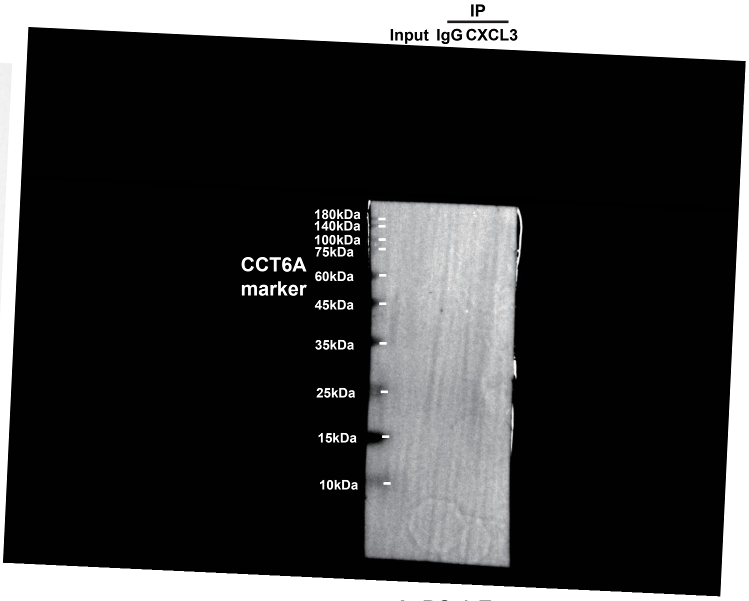


AsPC-1-Exo

Repeat#1

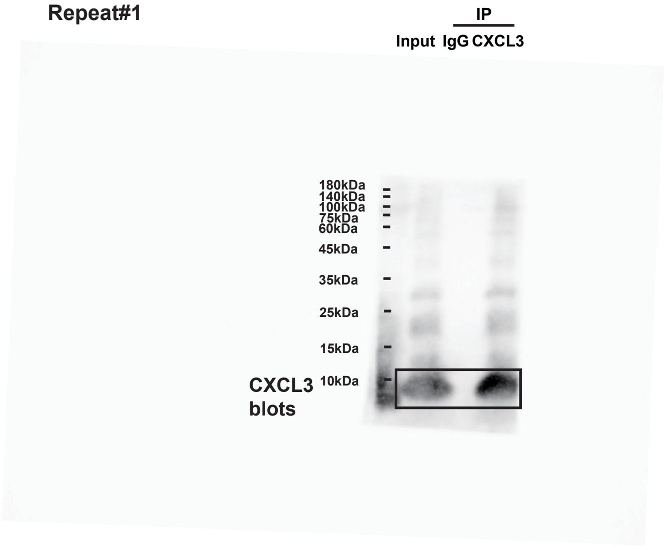


AsPC-1-Exo

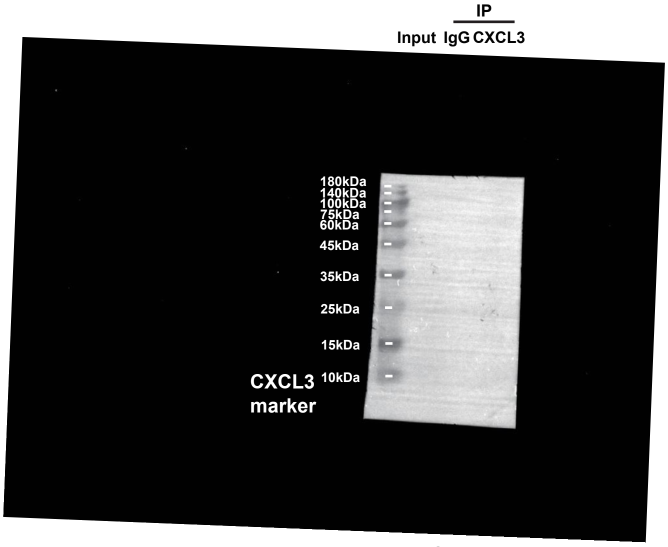


AsPC-1-Exo

Repeat#2

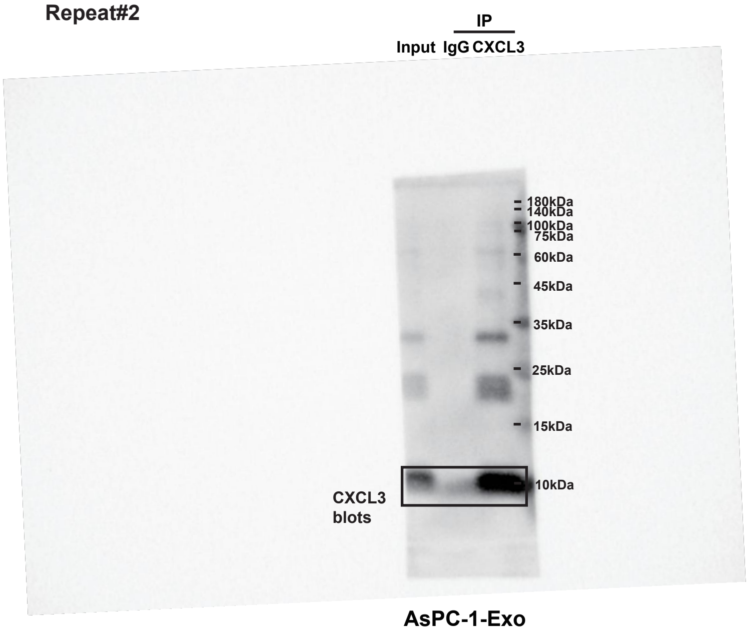


AsPC-1-Exo

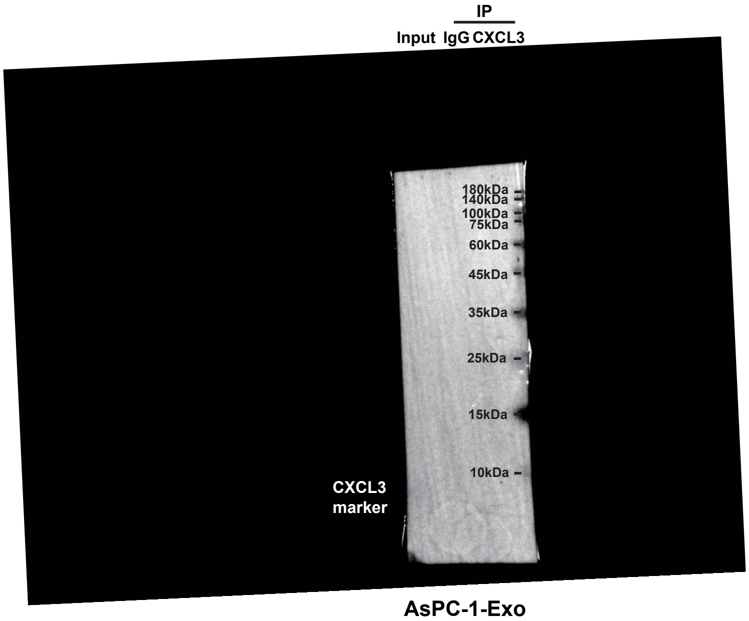


AsPC-1-Exo

Repeat#3

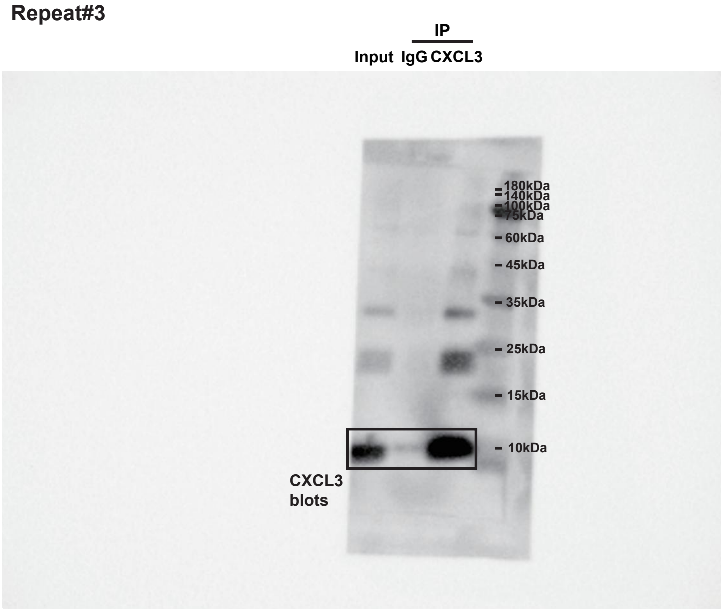


AsPC-1-Exo

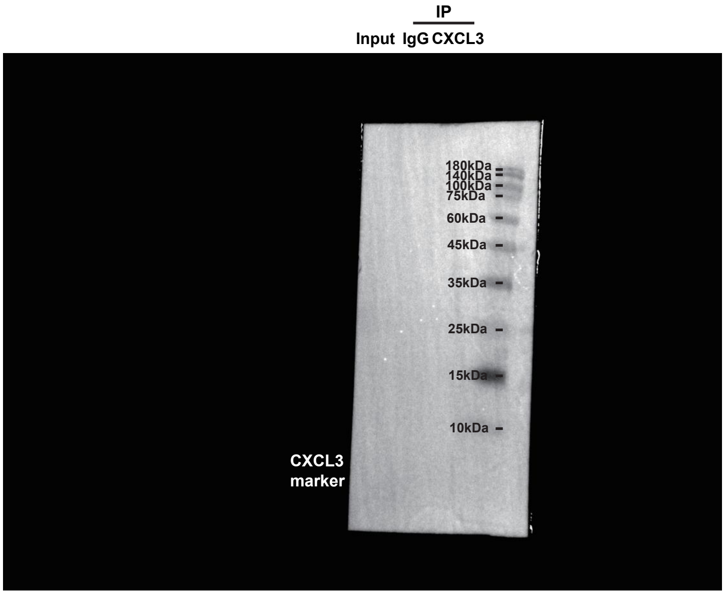


AsPC-1-Exo

Repeat#1

IP

Input IgG CCT6A

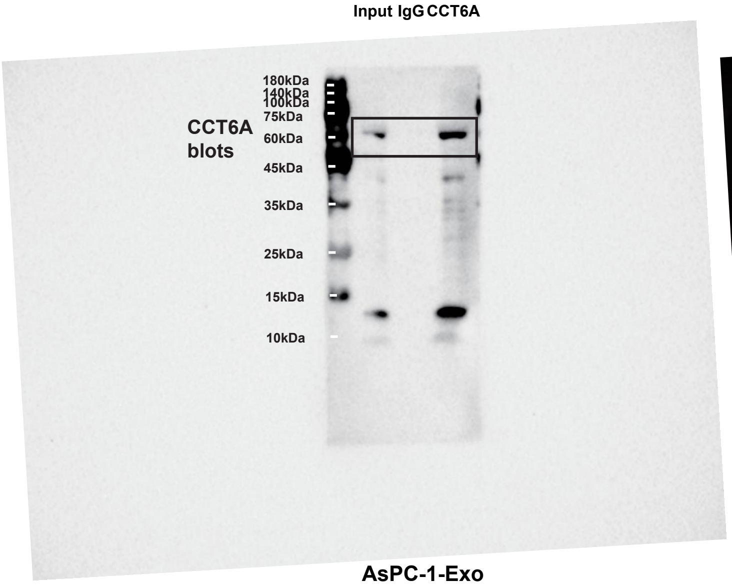


AsPC-1-Exo

IP

Input IgG CCT6A

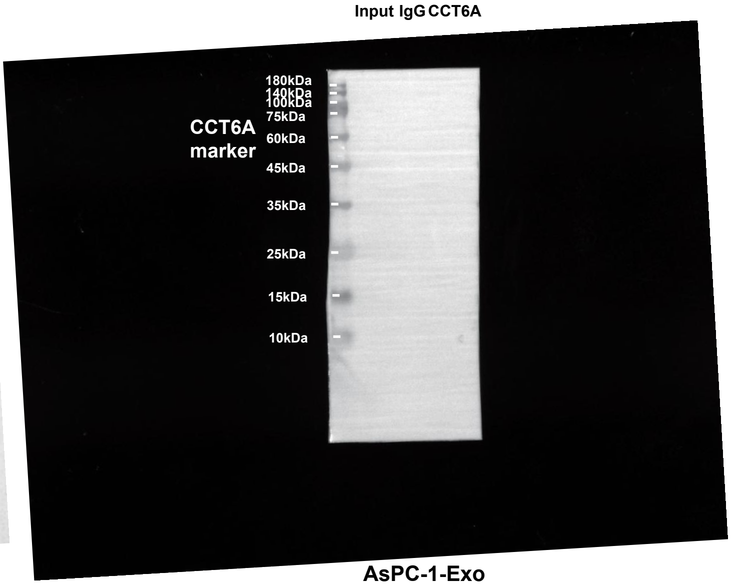


AsPC-1-Exo

Repeat#2

IP

Input IgG CCT6A

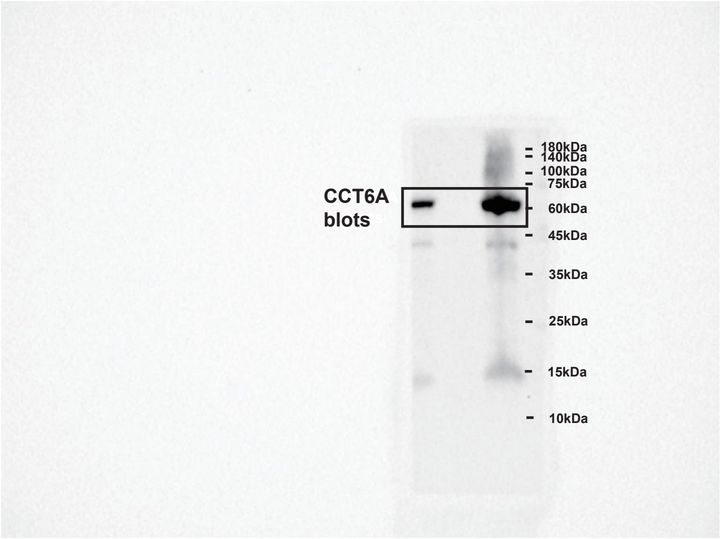


AsPC-1-Exo

IP

Input IgG CCT6A

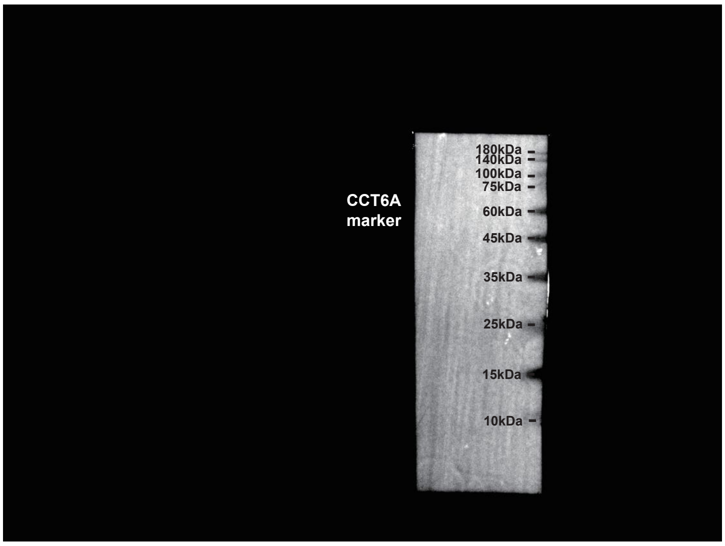


AsPC-1-Exo

Repeat#3

IP

Input IgG CCT6A

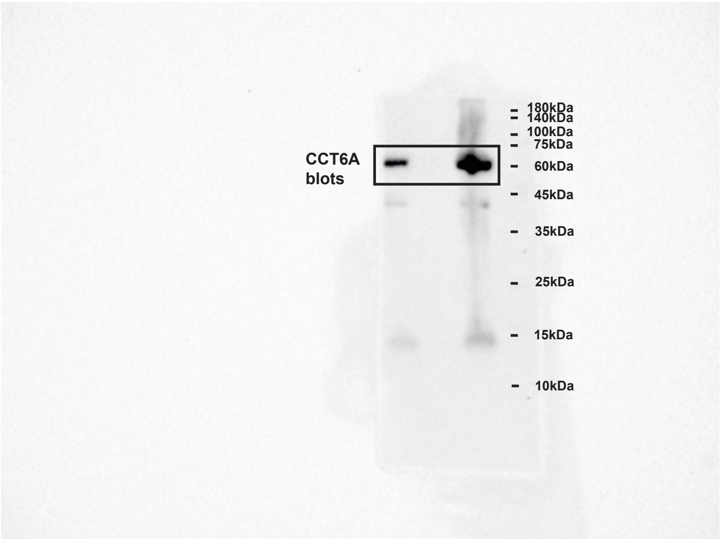


AsPC-1-Exo

IP

Input IgG CCT6A

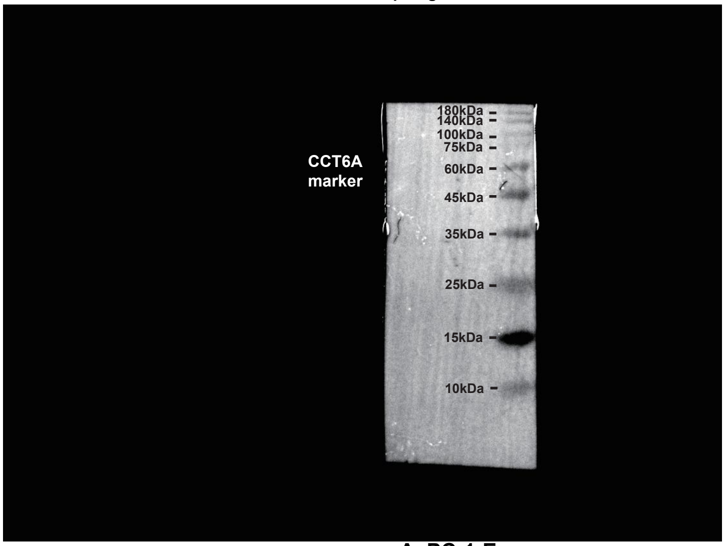


AsPC-1-Exo

**Repeat#1**

IP

Input IgG CCT6A

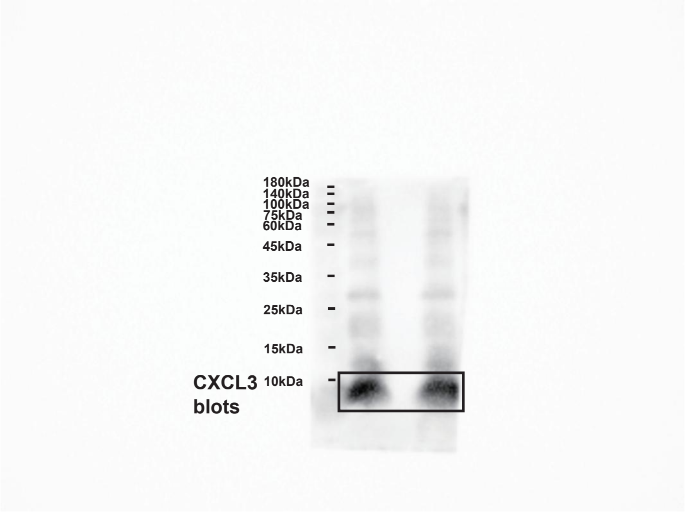


AsPC-1-Exo

IP

Input IgG CCT6A

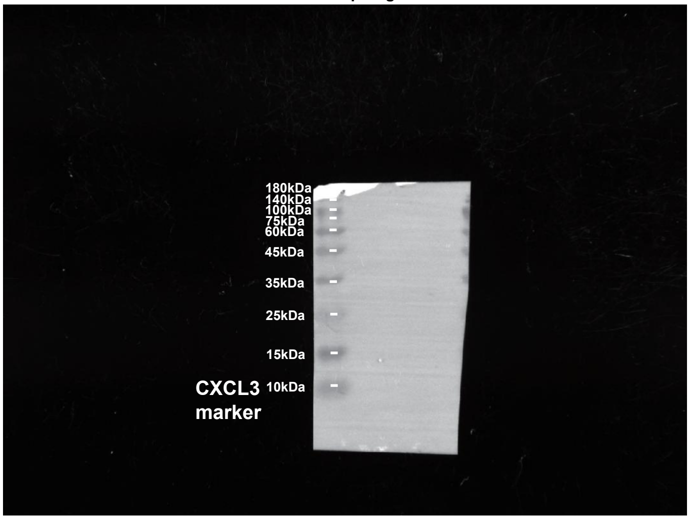


AsPC-1-Exo

**Repeat#2**

IP

Input IgG CCT6A

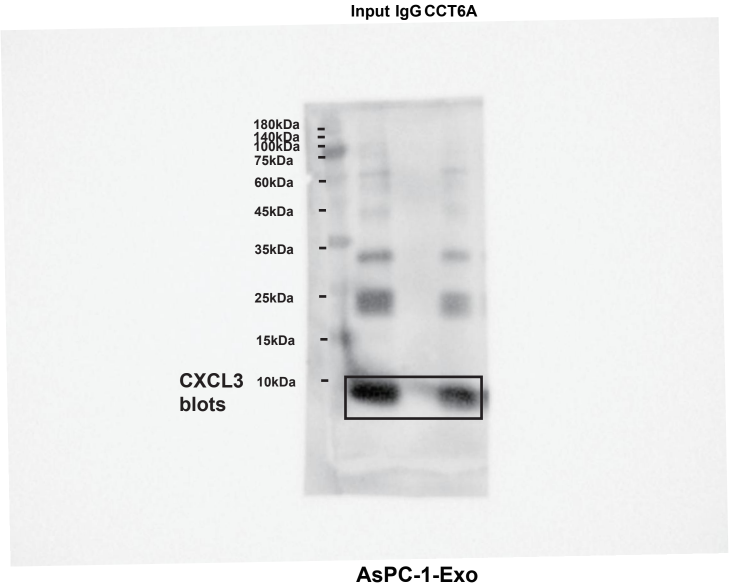


AsPC-1-Exo

IP

Input IgG CCT6A

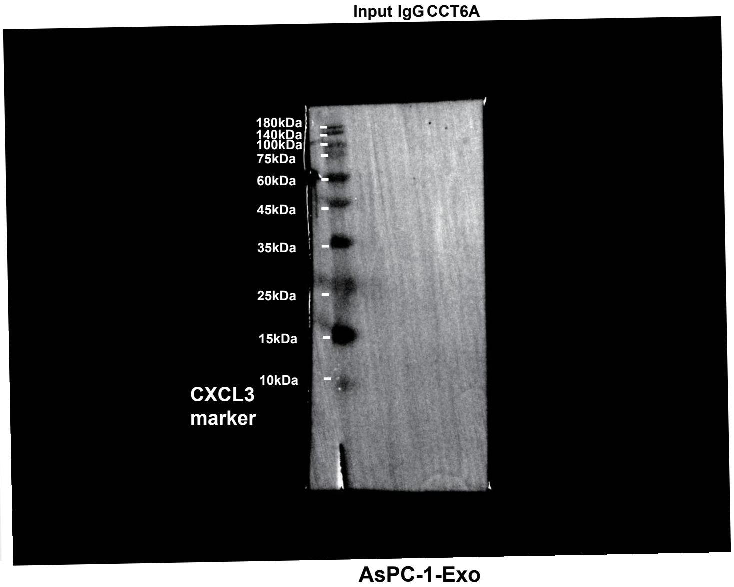


AsPC-1-Exo

**Repeat#3**

IP

Input IgG CCT6A

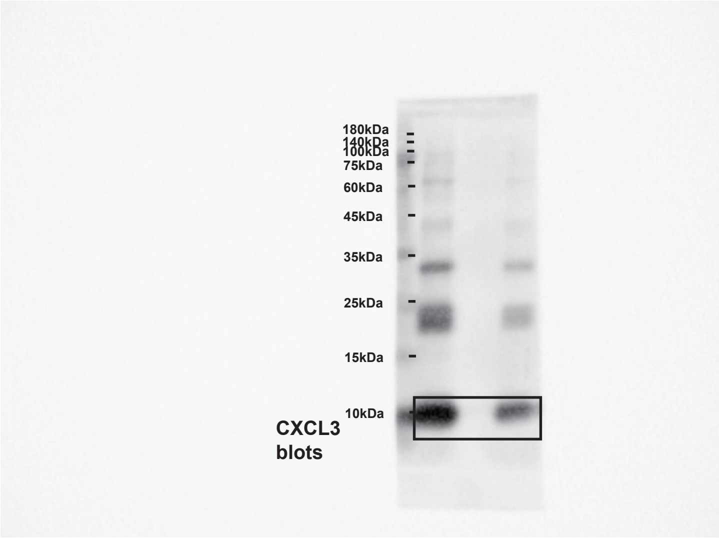


AsPC-1-Exo

IP

Input IgG CCT6A

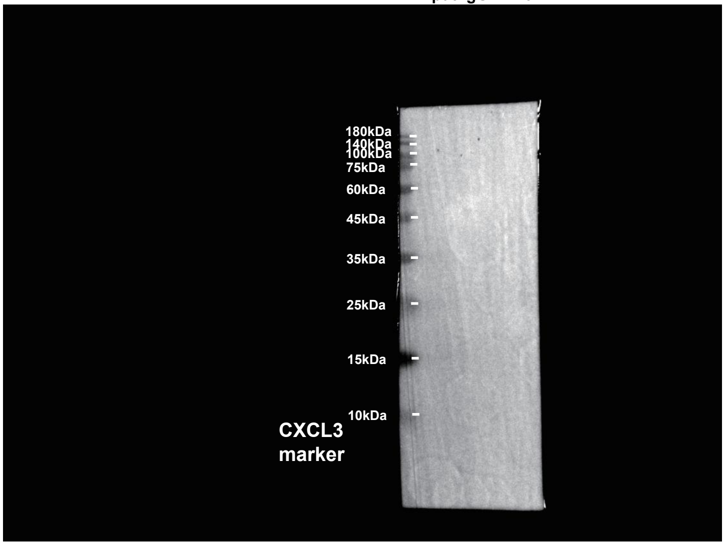


AsPC-1-Exo

Repeat#1

IP

Input IgG CCL5

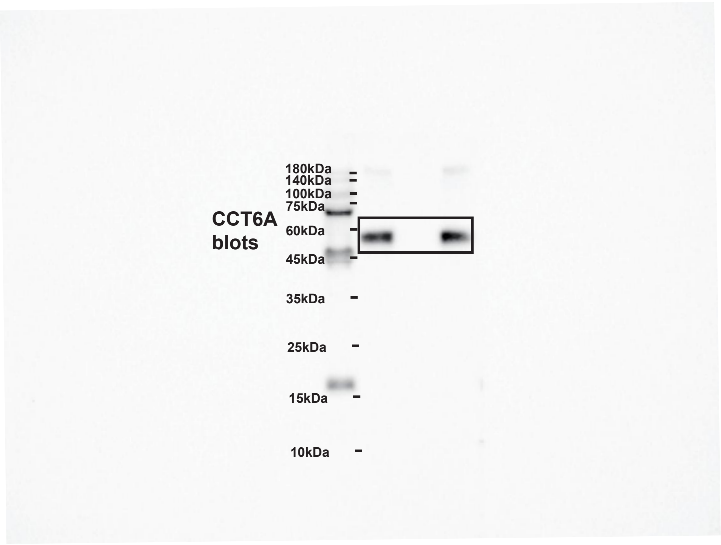


AsPC-1

IP

Input IgG CCL5

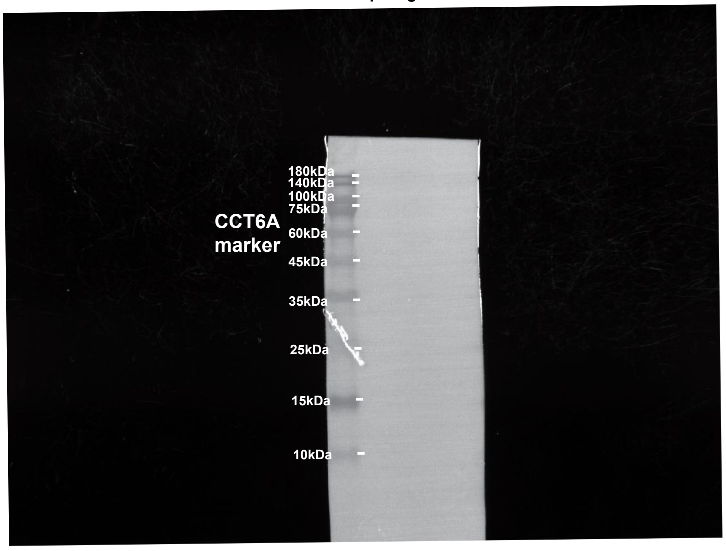


AsPC-1

Repeat#2 Repeat#3

IP IP

Input IgG CCL5 Input IgG CCL5

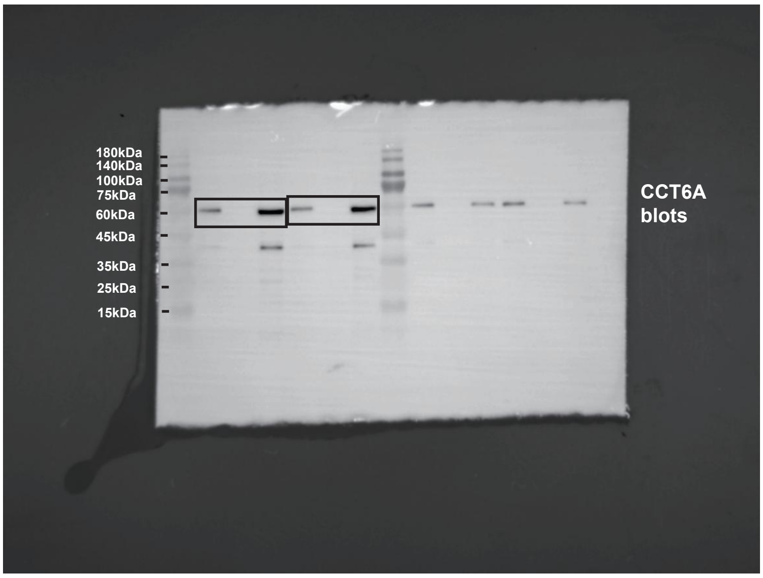


AsPC-1

Repeat#2 Repeat#3

IP IP

Input IgG CCL5 Input IgG CCL5

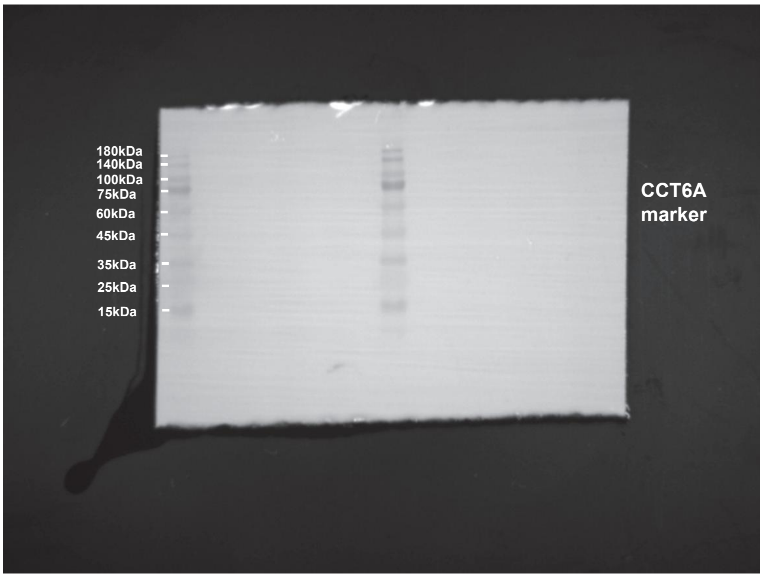


AsPC-1

Repeat#1

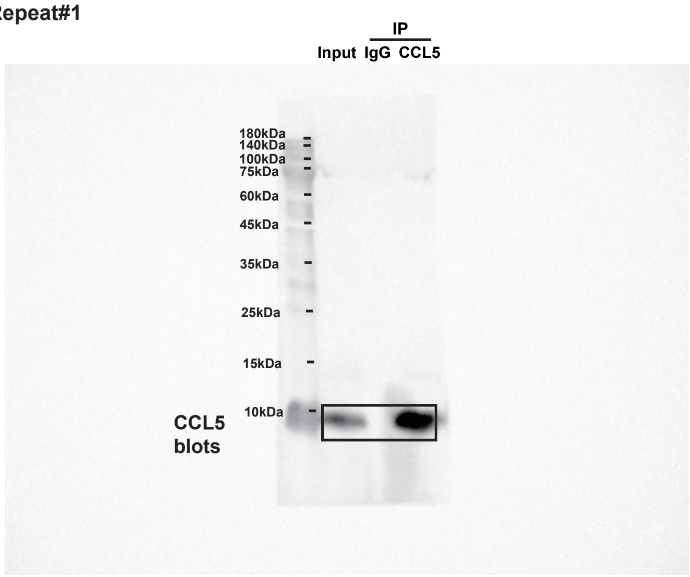


AsPC-1

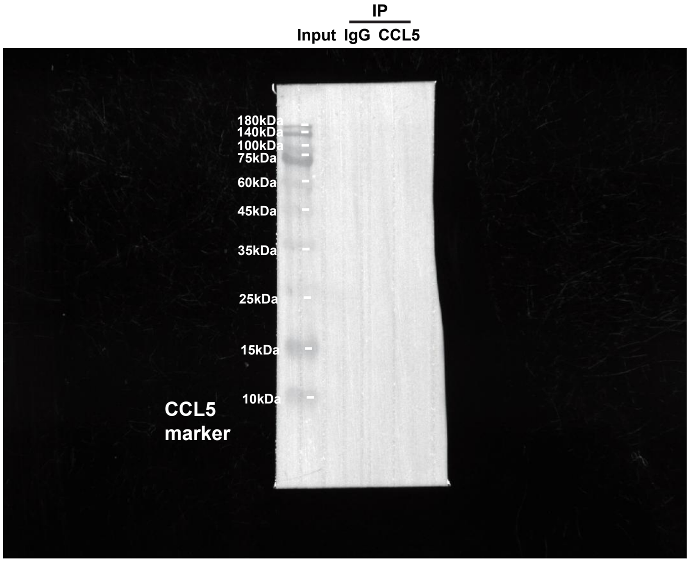


AsPC-1

Repeat#2 Repeat#3

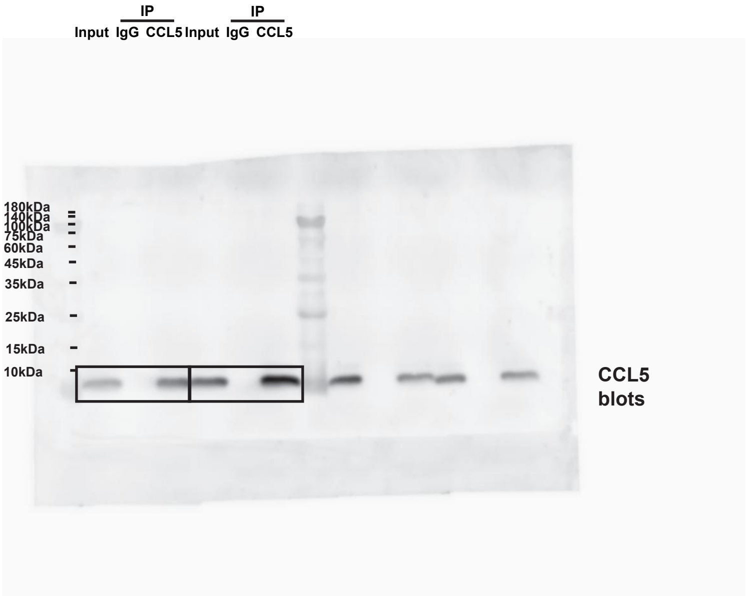


AsPC-1

Repeat#2 Repeat#3

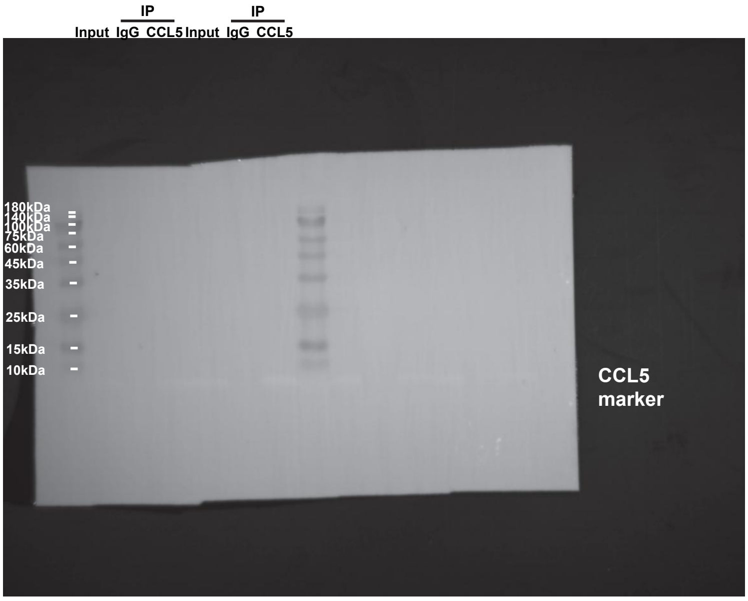


AsPC-1

Repeat#1

IP

Input IgG CCT6A

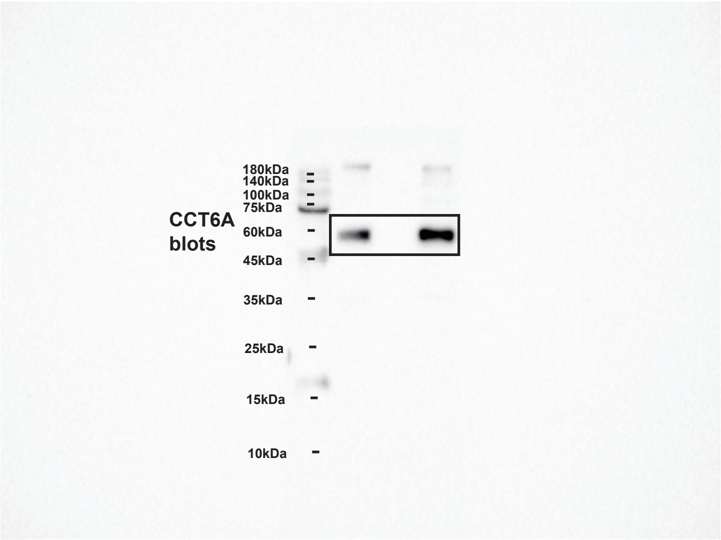


AsPC-1

IP

Input IgG CCT6A

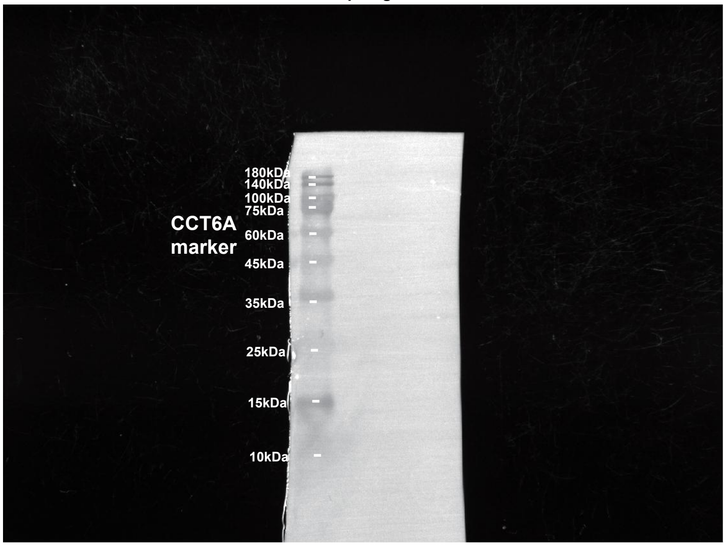


AsPC-1

Repeat#2 Repeat#3

IP IP

Input IgG CCT6A Input IgG CCT6A

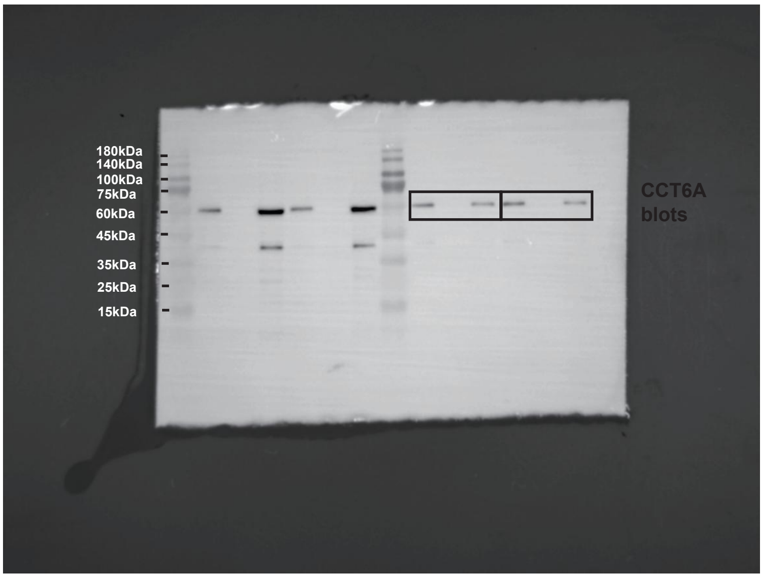


AsPC-1

Repeat#2 Repeat#3

IP IP

Input IgG CCT6A Input IgG CCT6A

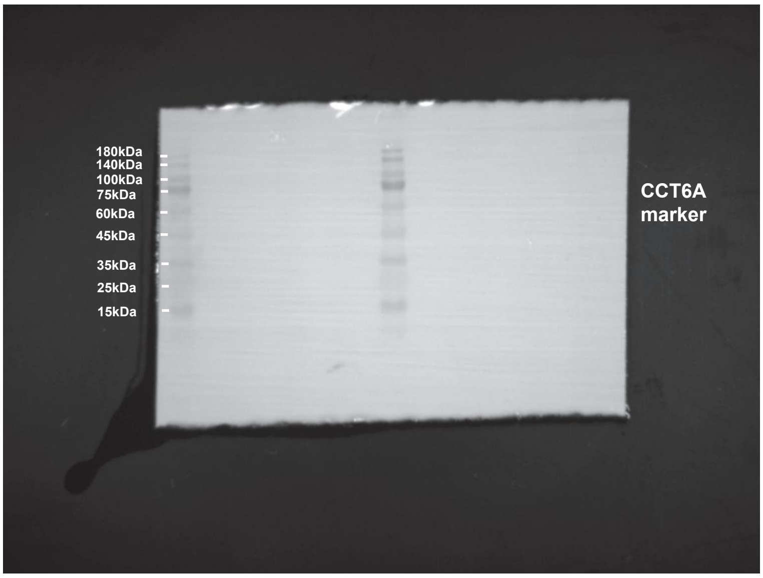


AsPC-1

Repeat#1

IP
Input IgG CCT6A

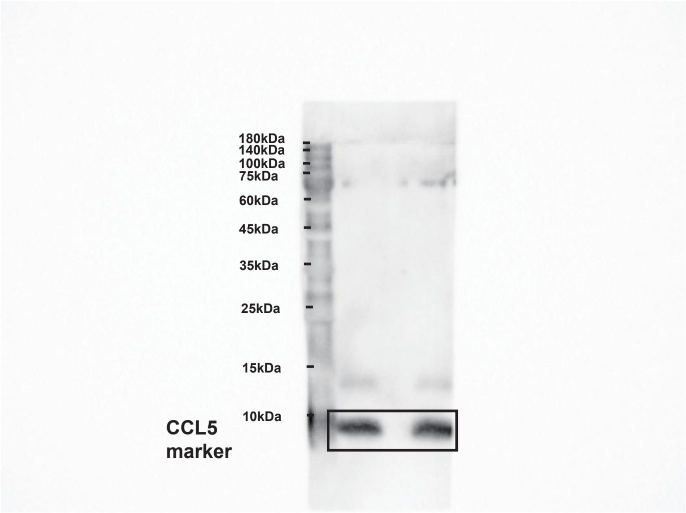


AsPC-1

IP
Input IgG CCT6A

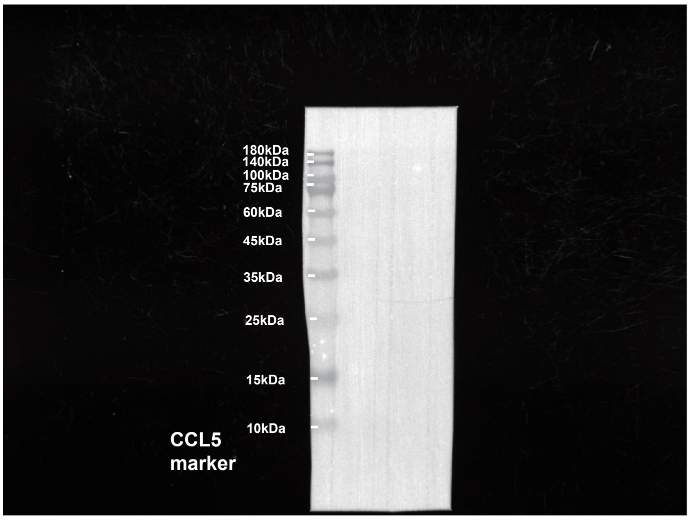


AsPC-1

Repeat#2 Repeat#3

IP IP
Input IgG CCT6A Input IgG CCT6A

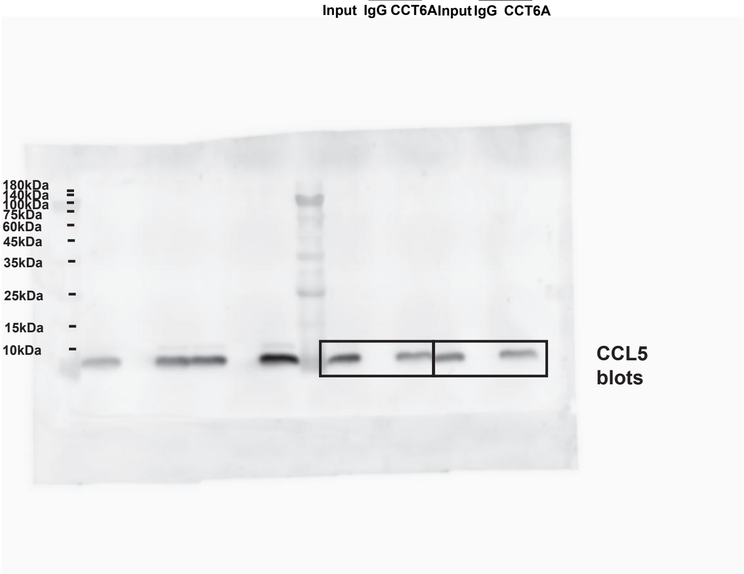


AsPC-1

Repeat#2 Repeat#3

IP IP
Input IgG CCT6A Input IgG CCT6A

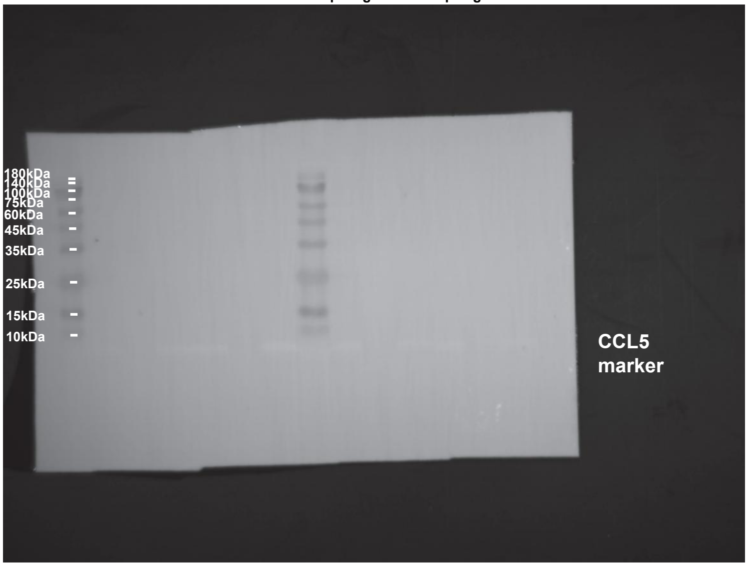


AsPC-1

Repeat#1

IP

Input IgG CCL20

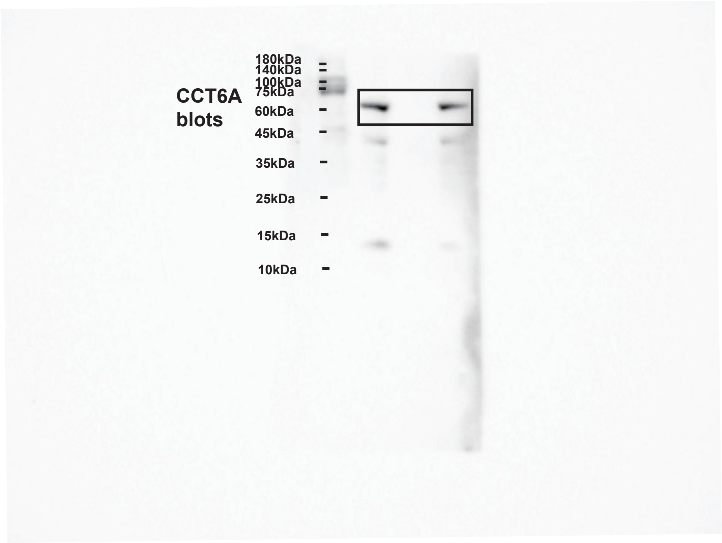


AsPC-1

IP

Input IgG CCL20

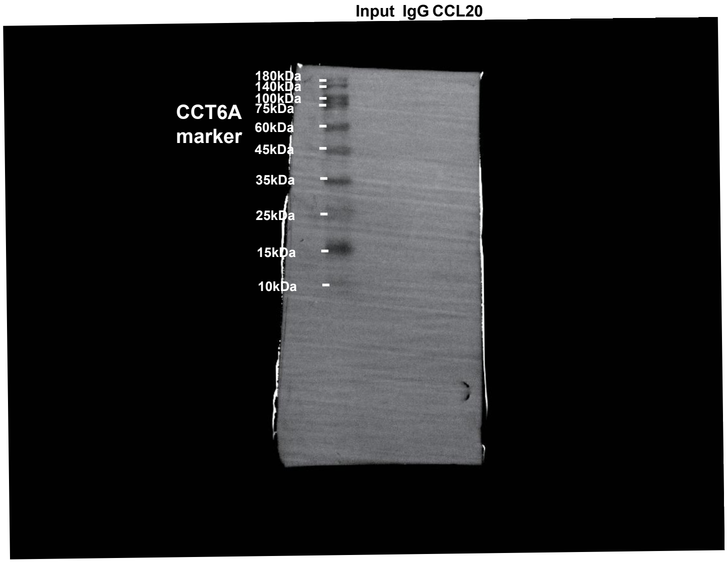


AsPC-1

Repeat#2 Repeat#3

IP IP

Input IgG CCL20 Input IgG CCL20

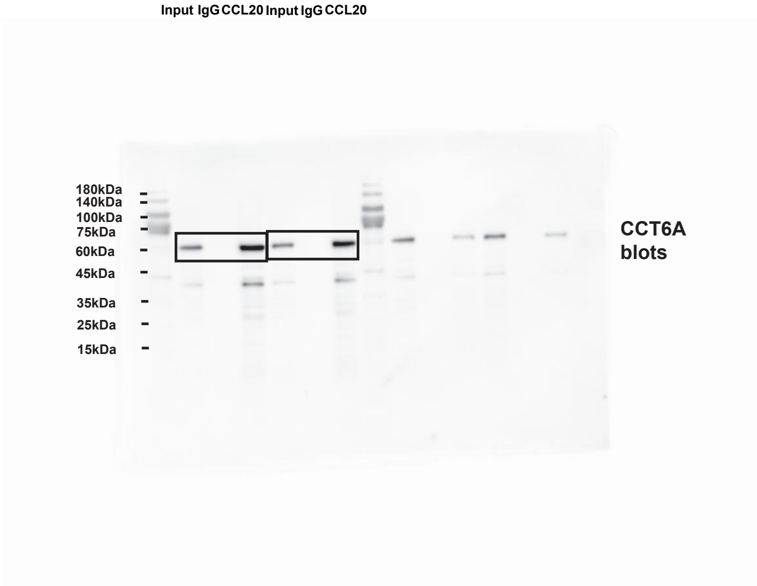


AsPC-1

Repeat#2 Repeat#3

IP IP

Input IgG CCL20 Input IgG CCL20

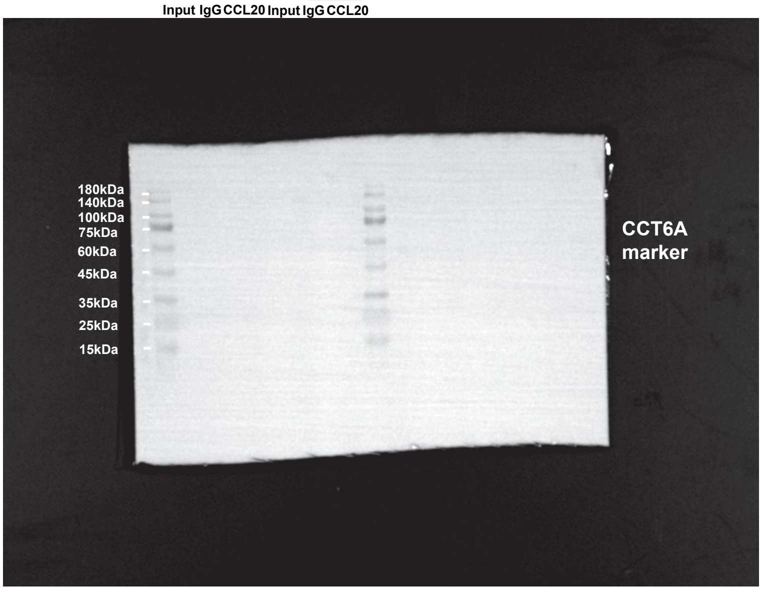


AsPC-1

Repeat#1

IP
Input IgG CCL20

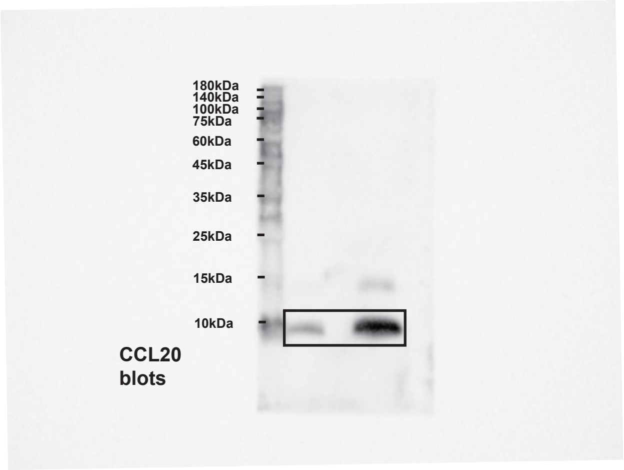


AsPC-1

IP
Input IgG CCL20

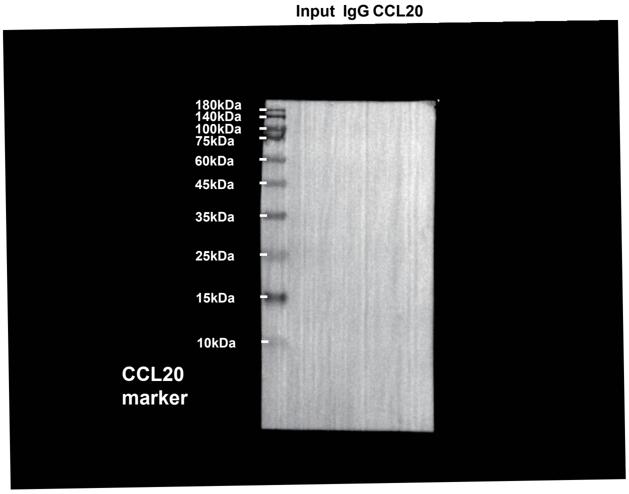


AsPC-1

Repeat#2 Repeat#3

IP IP
Input IgG CCL20 Input IgG CCL20

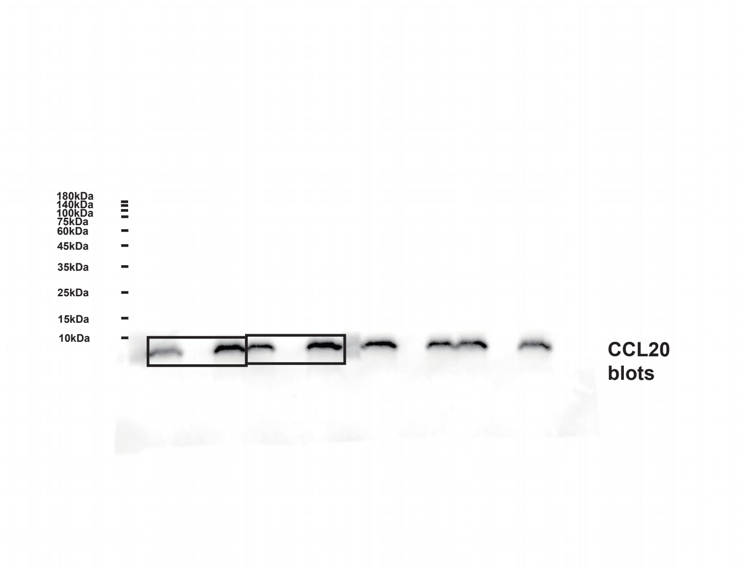


AsPC-1

Repeat#2 Repeat#3

IP IP
Input IgG CCL20 Input IgG CCL20

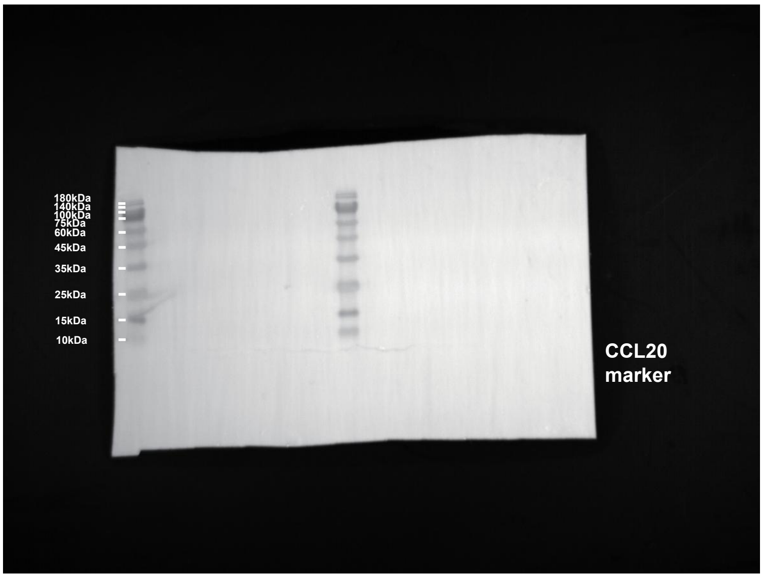


AsPC-1

Repeat#1

IP
Input IgG CCT6A

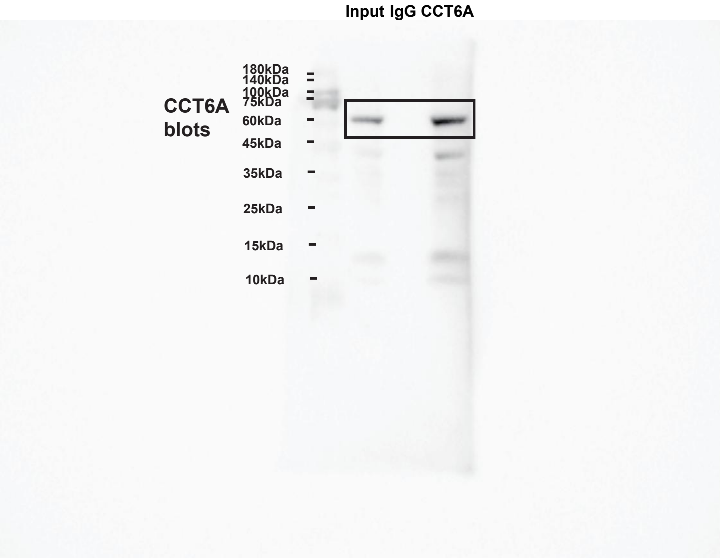


AsPC-1

IP
Input IgG CCT6A

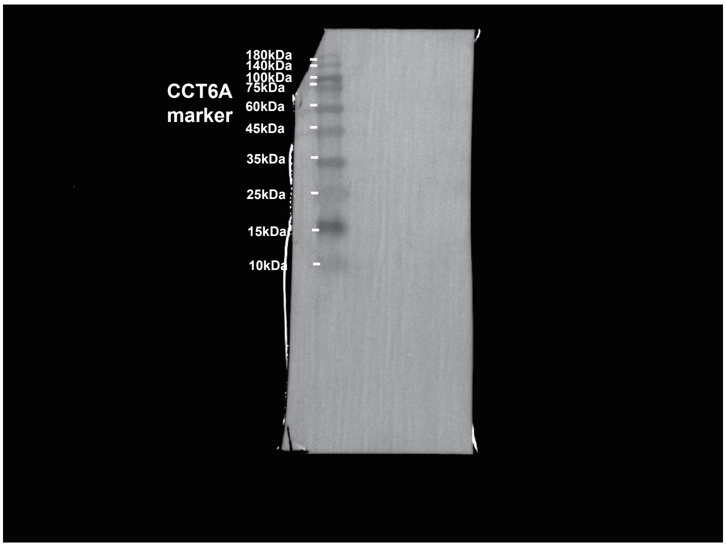


AsPC-1

Repeat#2 Repeat#3

IP IP
Input IgG CCT6A Input IgG CCT6A

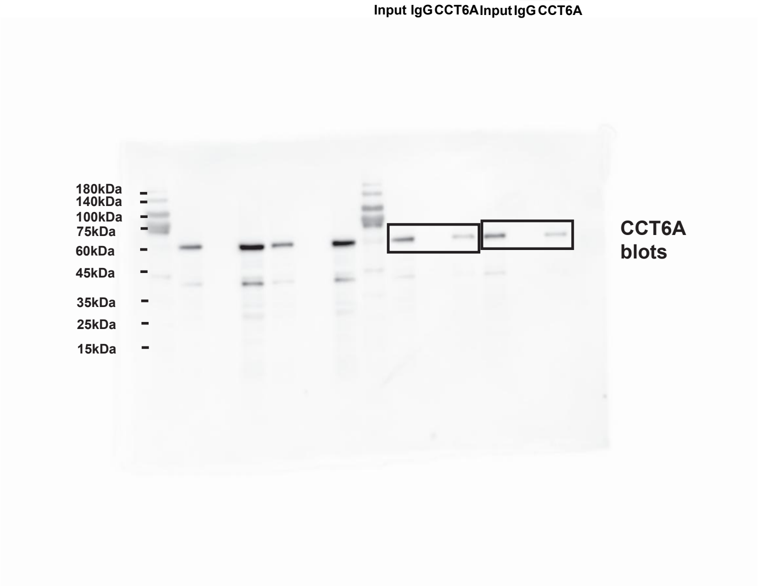


AsPC-1

Repeat#2 Repeat#3

IP IP
Input IgG CCT6A Input IgG CCT6A

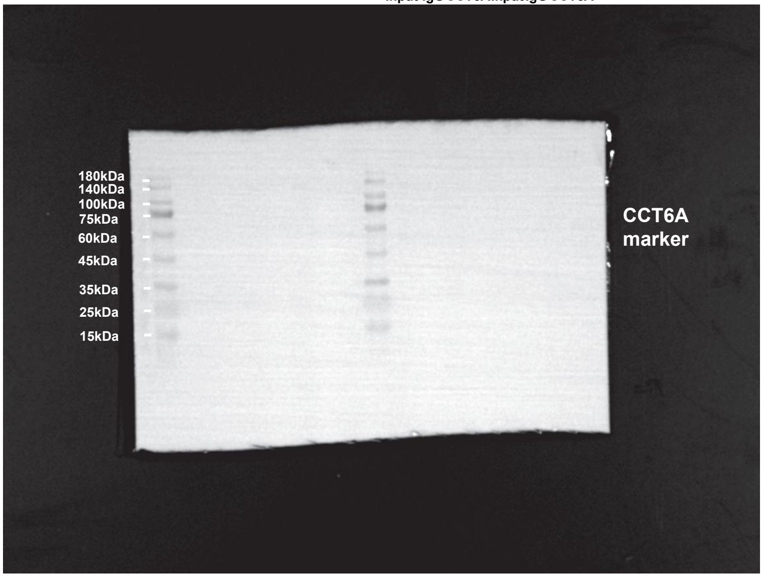


AsPC-1

Repeat#1

IP
Input IgG CCT6A

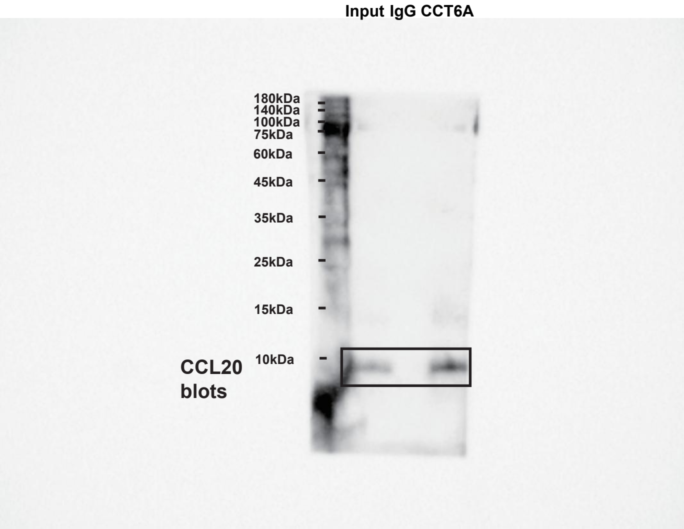


AsPC-1

IP
Input IgG CCT6A

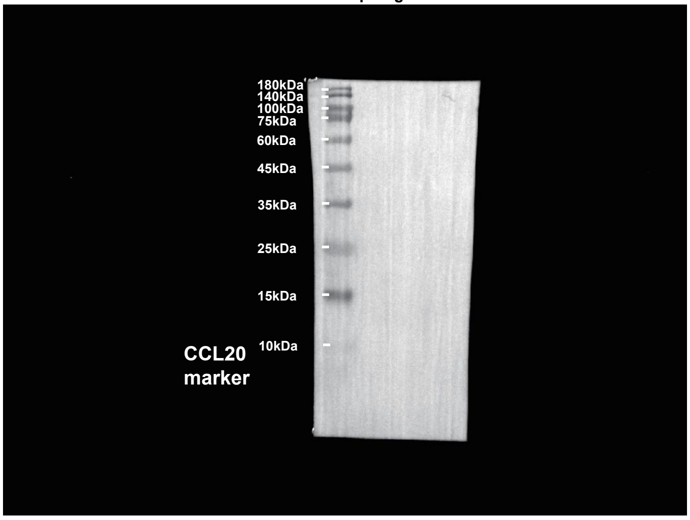


AsPC-1

Repeat#2 Repeat#3

IP IP
Input IgG CCT6A Input IgG CCT6A

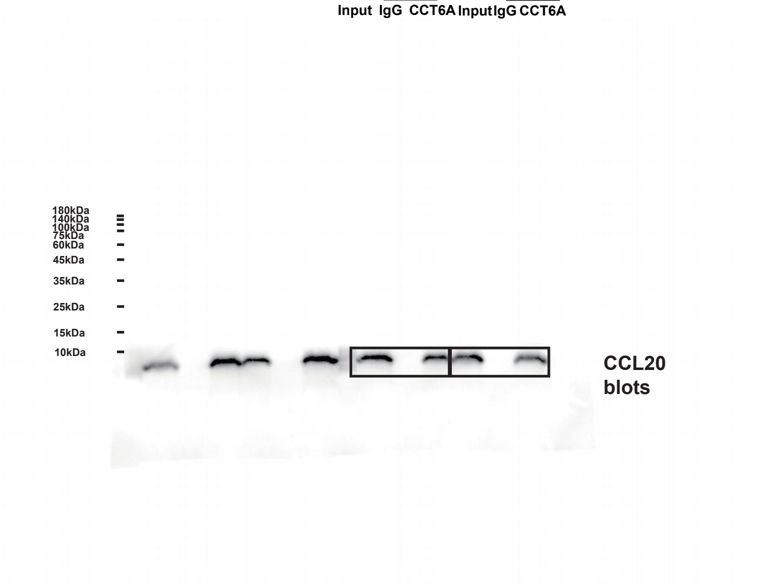


CCL20
blots

AsPC-1

Repeat#2 Repeat#3

IP IP
Input IgG CCT6A Input IgG CCT6A

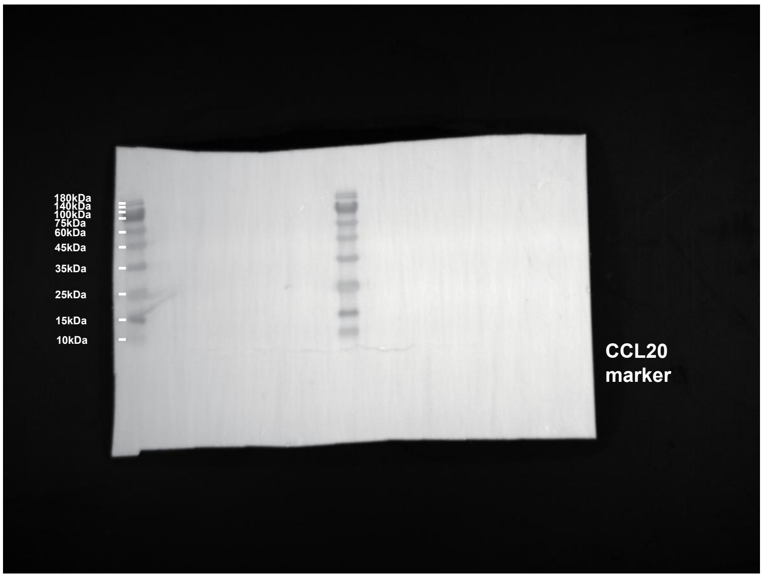


AsPC-1

Repeat#1

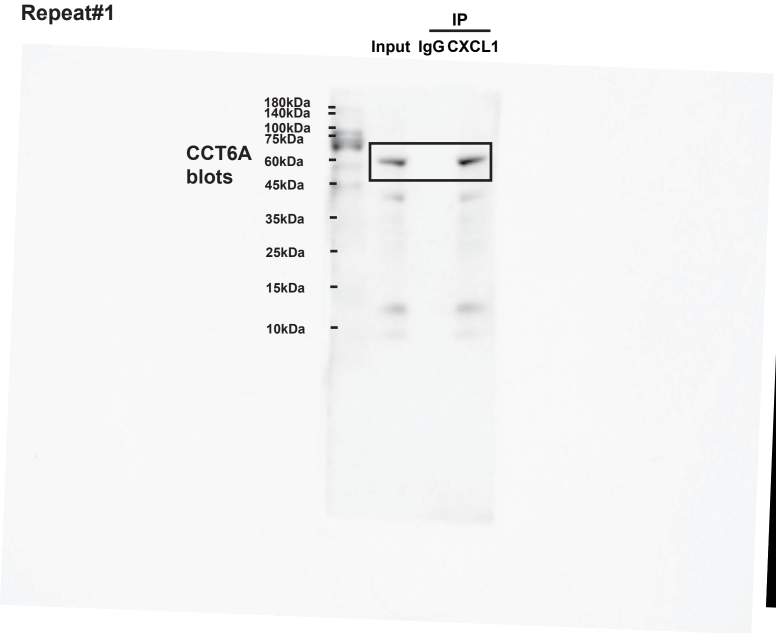


AsPC-1

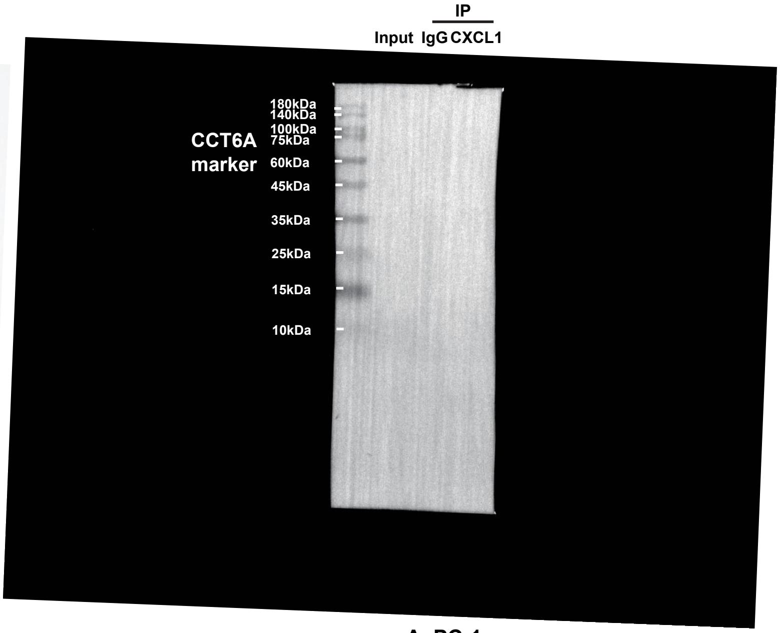


AsPC-1

Repeat#2 Repeat#3

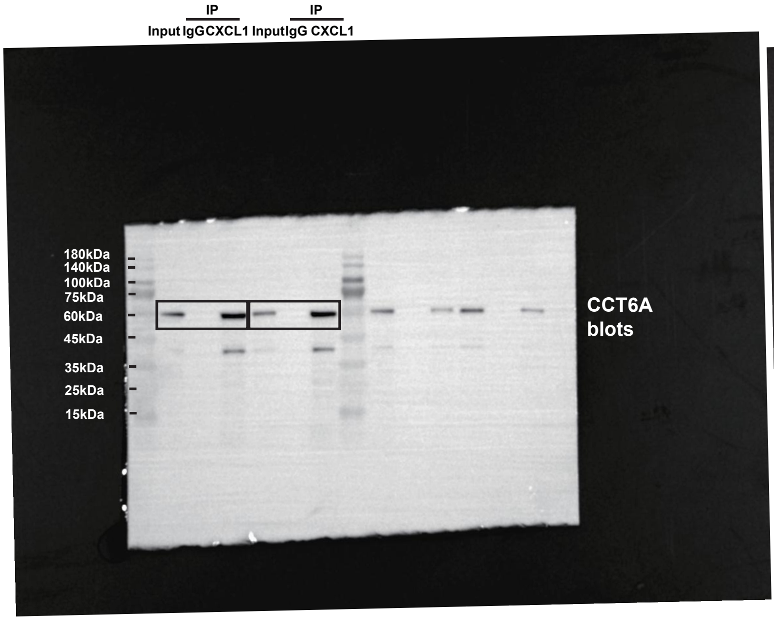


AsPC-1

Repeat#2 Repeat#3

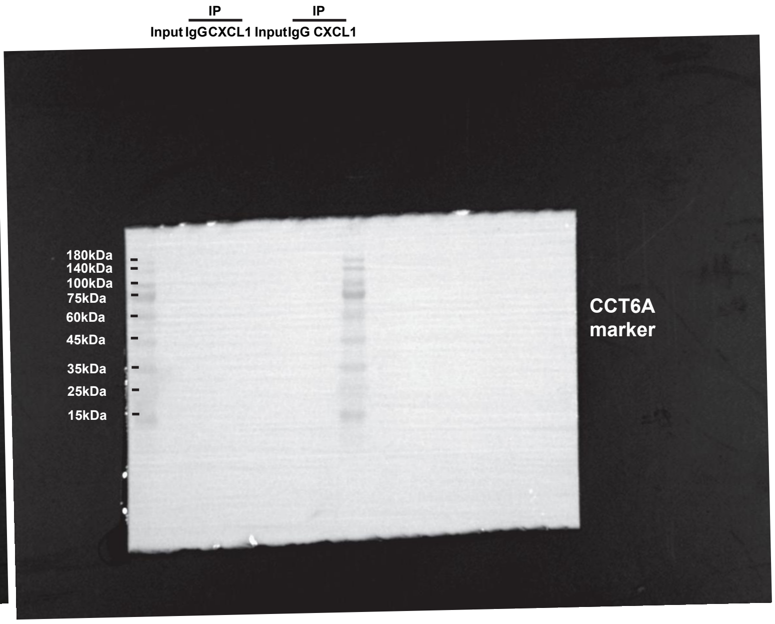


AsPC-1

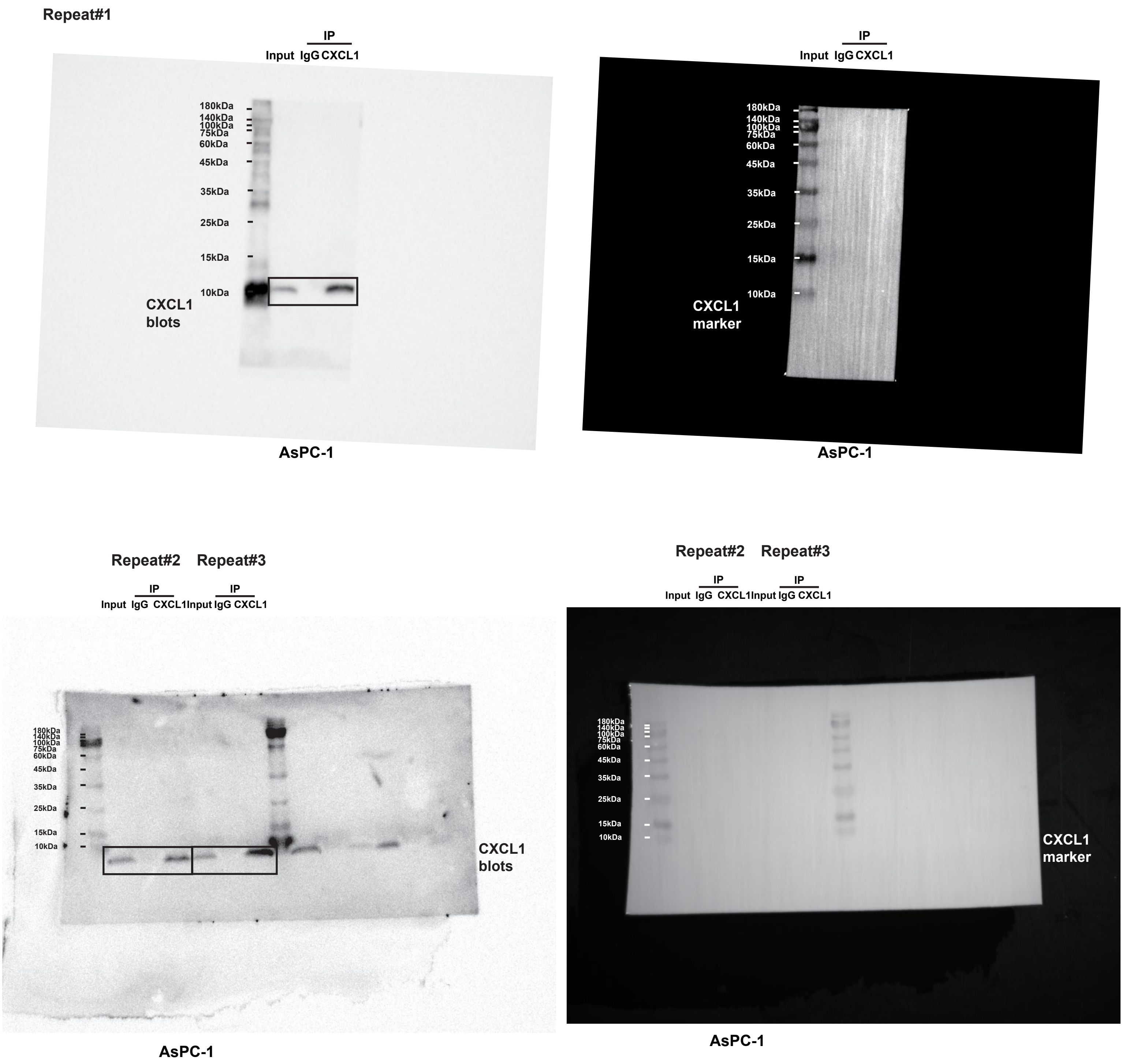

Repeat#1
IP
Input IgG CXCL1
180kDa
140kDa
100kDa
75kDa
60kDa
45kDa
35kDa
25kDa
15kDa
10kDa
CXCL1 blots
AsPC-1
IP
Input IgG CXCL1
180kDa
140kDa
100kDa
75kDa
60kDa
45kDa
35kDa
25kDa
15kDa
10kDa
CXCL1 marker
AsPC-1
Repeat#2 Repeat#3
IP IP
Input IgG CXCL1 Input IgG CXCL1
180kDa
140kDa
100kDa
75kDa
60kDa
45kDa
35kDa
25kDa
15kDa
10kDa
CXCL1 blots
AsPC-1
Repeat#2 Repeat#3
IP IP
Input IgG CXCL1 Input IgG CXCL1
180kDa
140kDa
100kDa
75kDa
60kDa
45kDa
35kDa
25kDa
15kDa
10kDa
CXCL1 marker
AsPC-1

Repeat#1

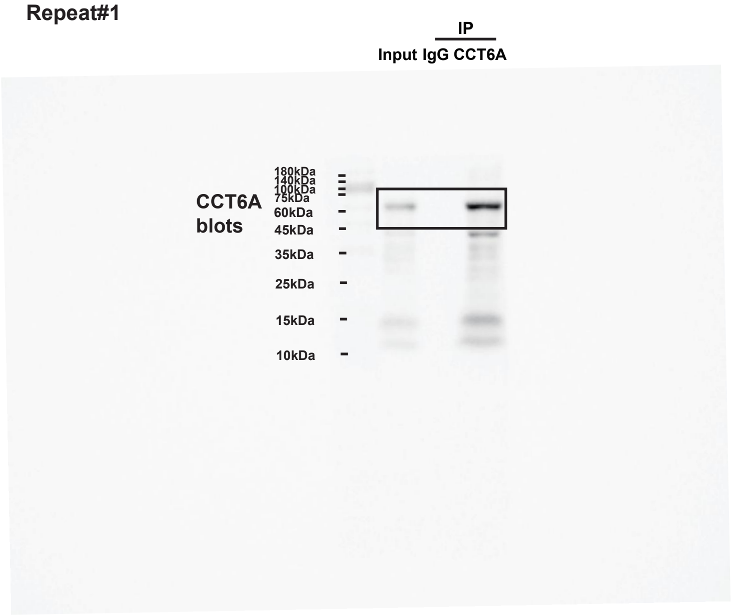


AsPC-1

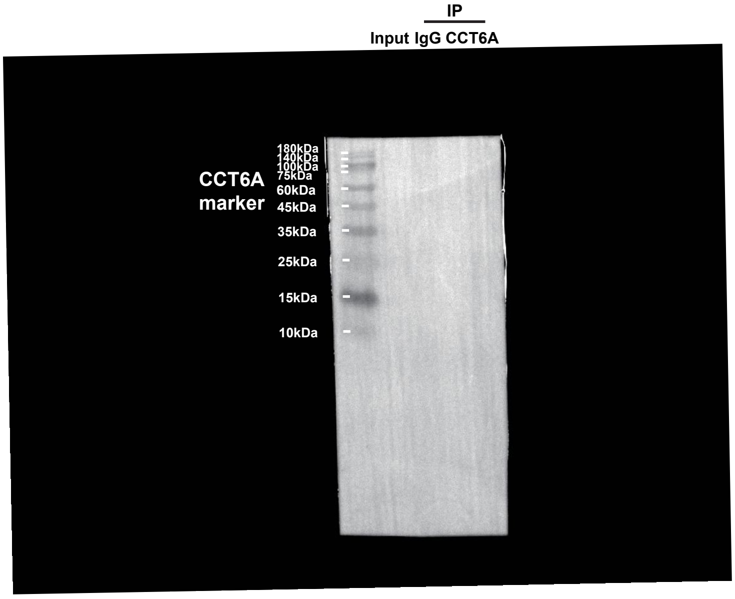


AsPC-1

Repeat#2 Repeat#3

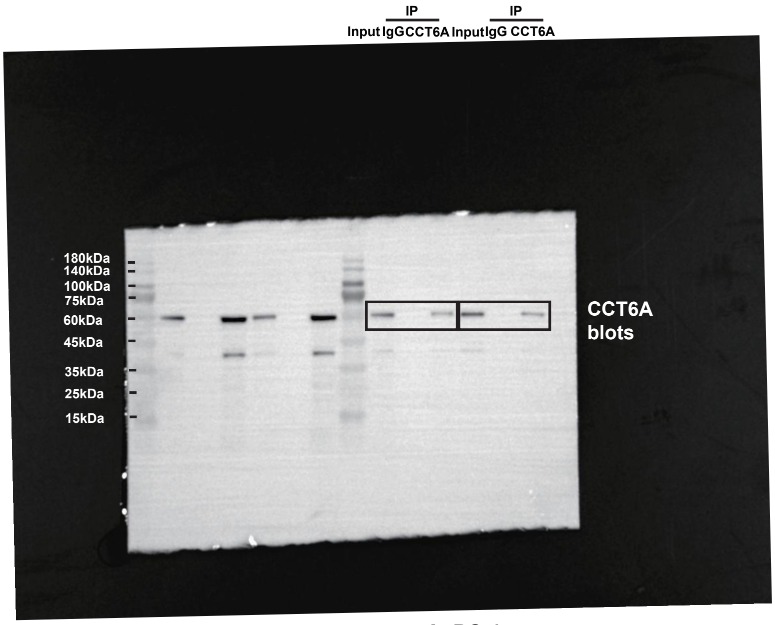


AsPC-1

Repeat#2 Repeat#3

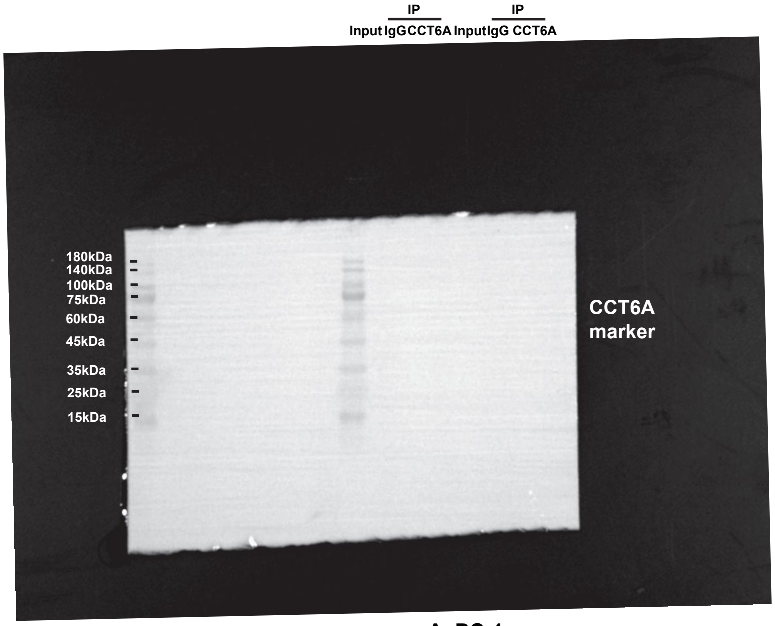


AsPC-1

**Repeat#1**

IP

Input IgG CCT6A

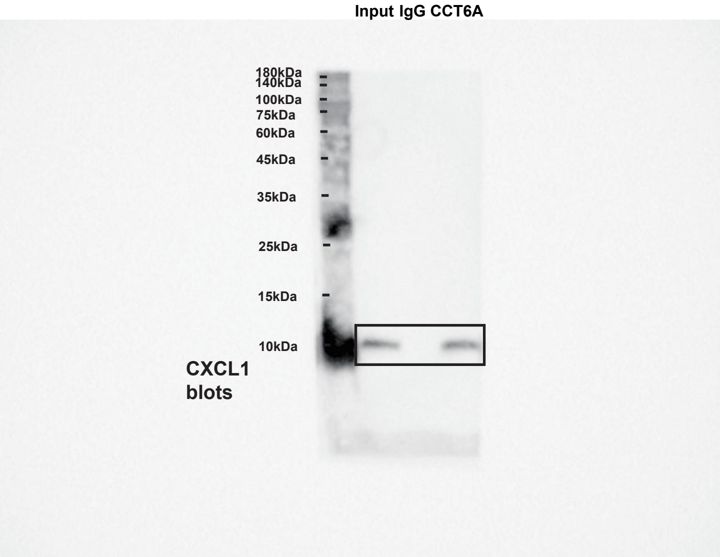


**AsPC-1**

IP

Input IgG CCT6A

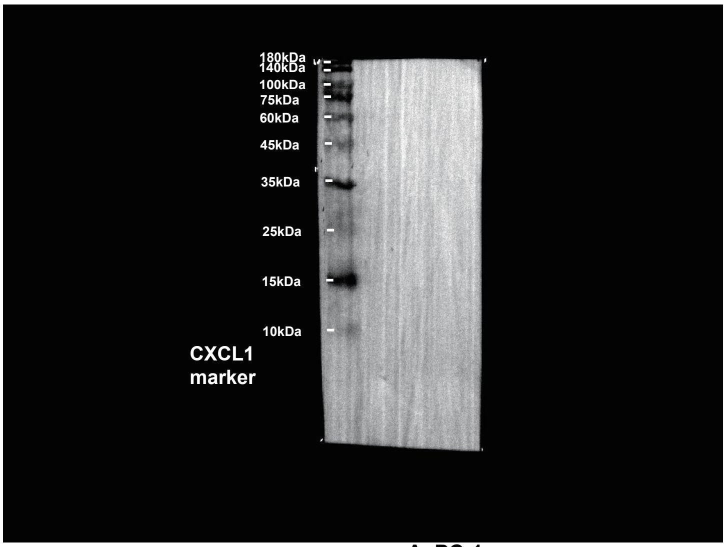


**AsPC-1**

**Repeat#2 Repeat#3**

IP IP

Input IgG CCT6A Input IgG CCT6A

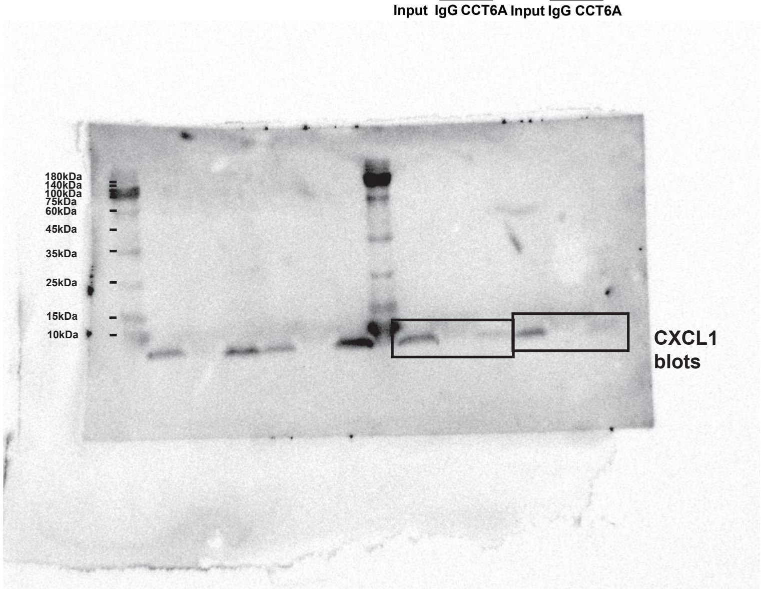


**AsPC-1**

**Repeat#2 Repeat#3**

IP IP

Input IgG CCT6A Input IgG CCT6A

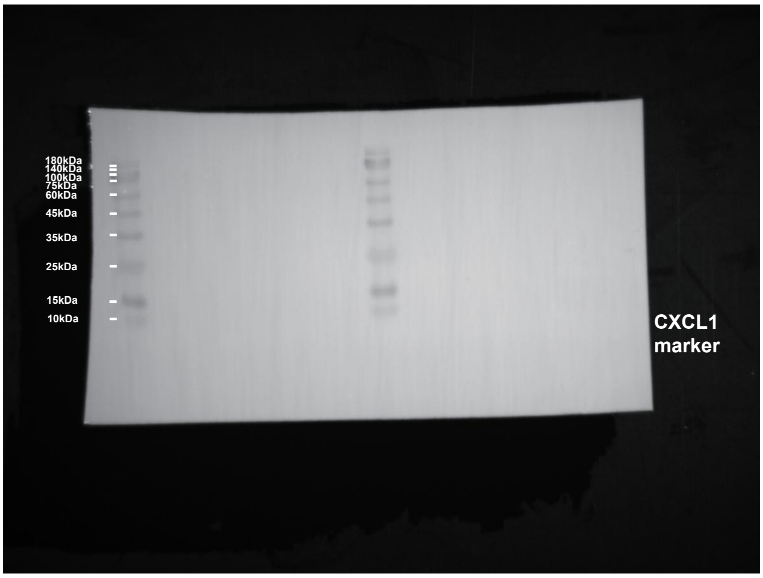


**AsPC-1**

Repeat#1

IP

Input IgG CXCL3

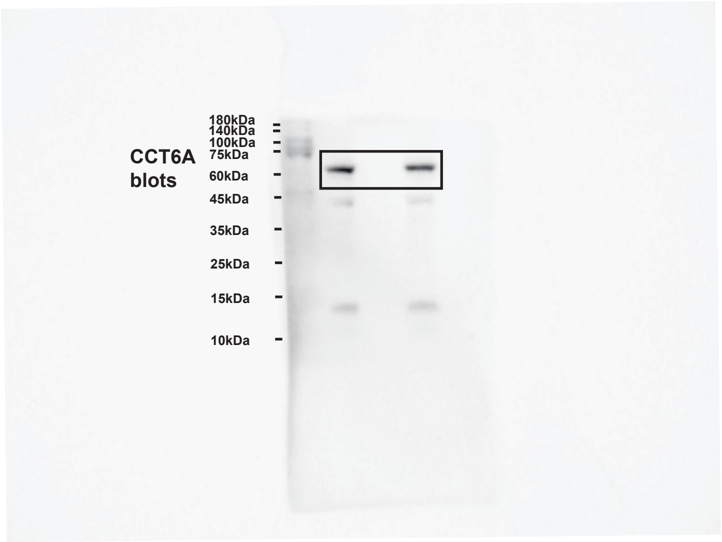


AsPC-1

IP

Input IgG CXCL3

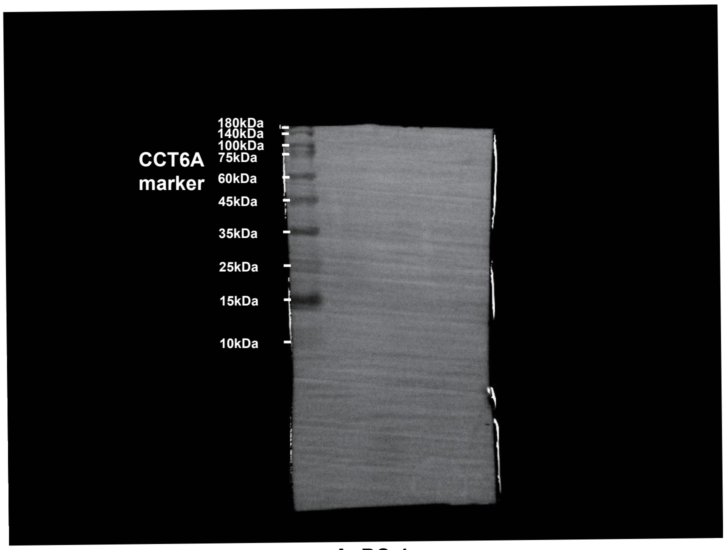


AsPC-1

Repeat#2 Repeat#3

IP IP

Input IgG CXCL3 Input IgG CXCL3

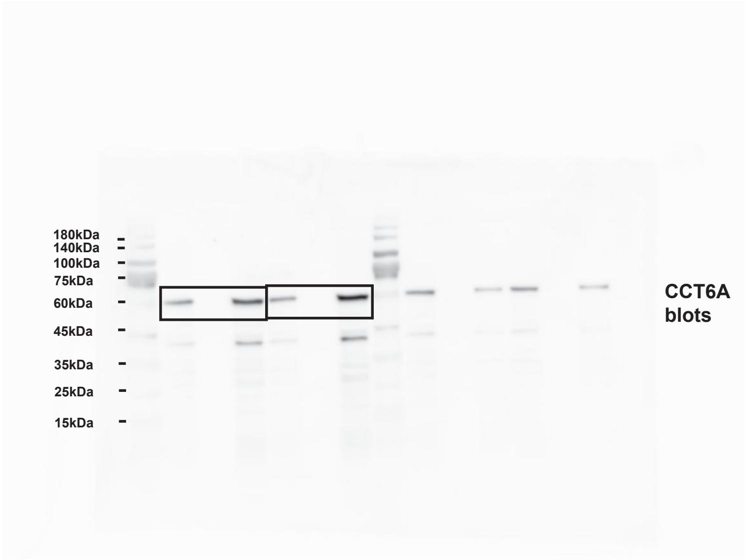


AsPC-1

Repeat#2 Repeat#3

IP IP

Input IgG CXCL3 Input IgG CXCL3

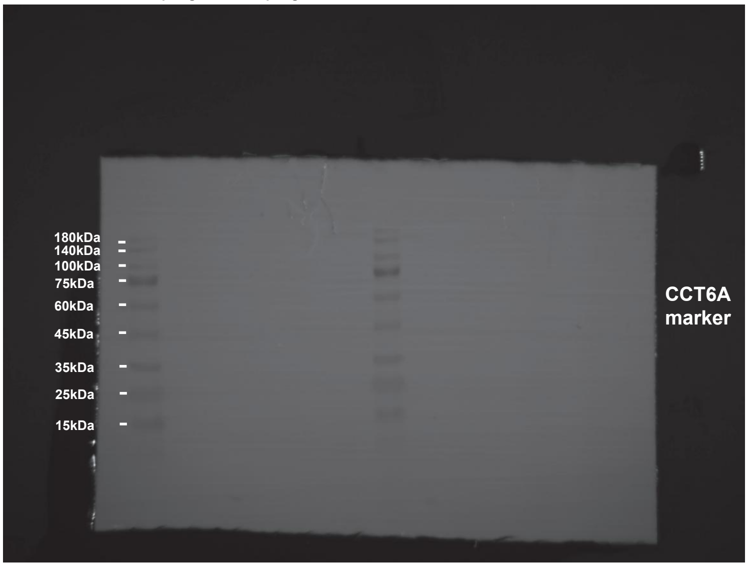


AsPC-1

**Repeat#1**

IP

Input IgG CXCL3

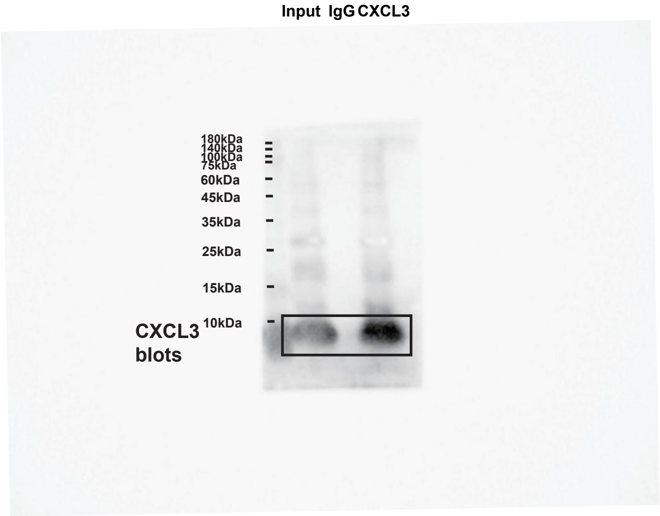


**AsPC-1**

IP

Input IgG CXCL3

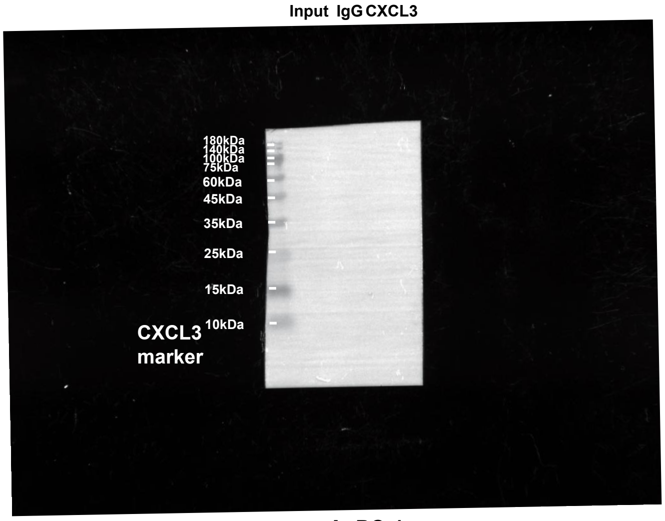


**AsPC-1**

**Repeat#2 Repeat#3**

IP IP

Input IgG CXCL3 Input IgG CXCL3

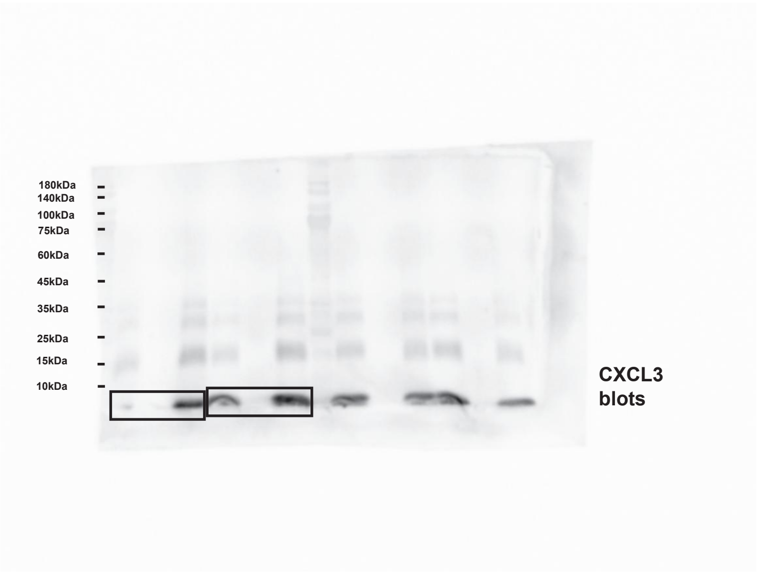


CXCL3
blots

**AsPC-1**

**Repeat#2 Repeat#3**

IP IP

Input IgG CXCL3 Input IgG CXCL3

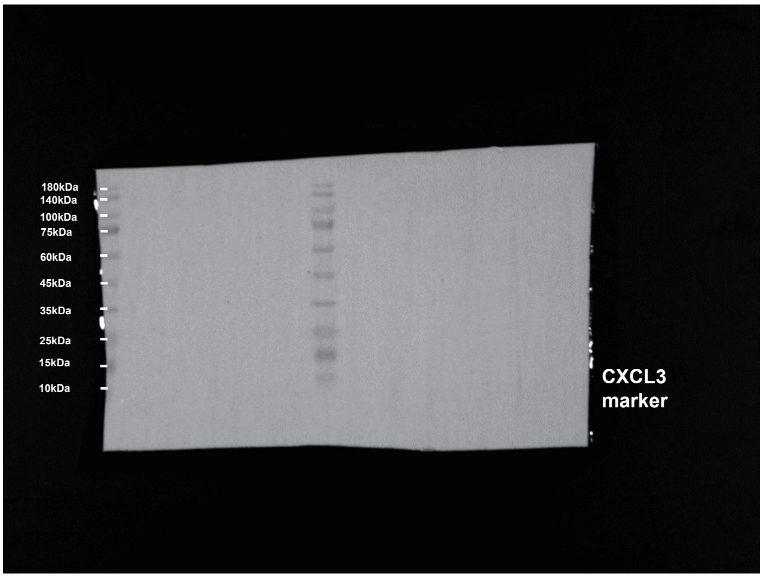


**AsPC-1**

Repeat#1

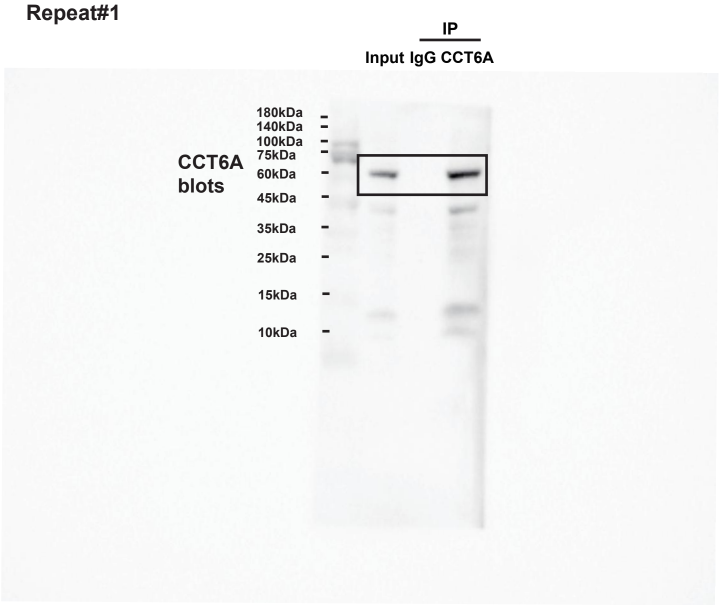


AsPC-1

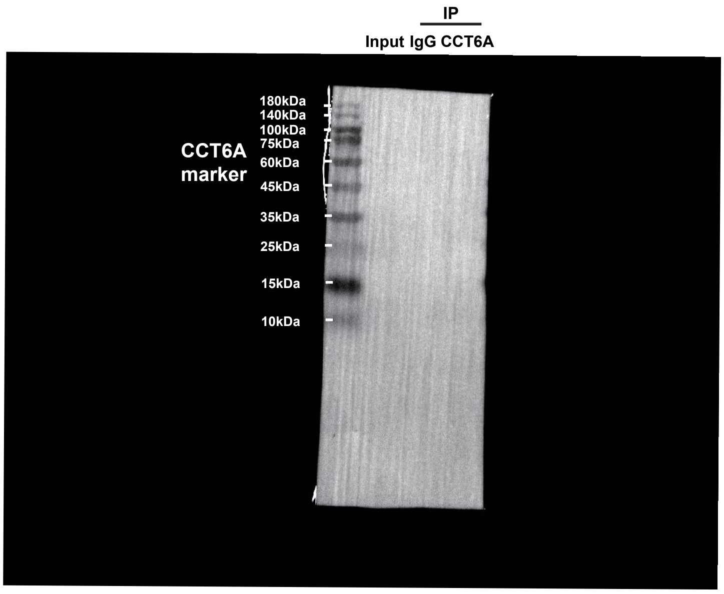


AsPC-1

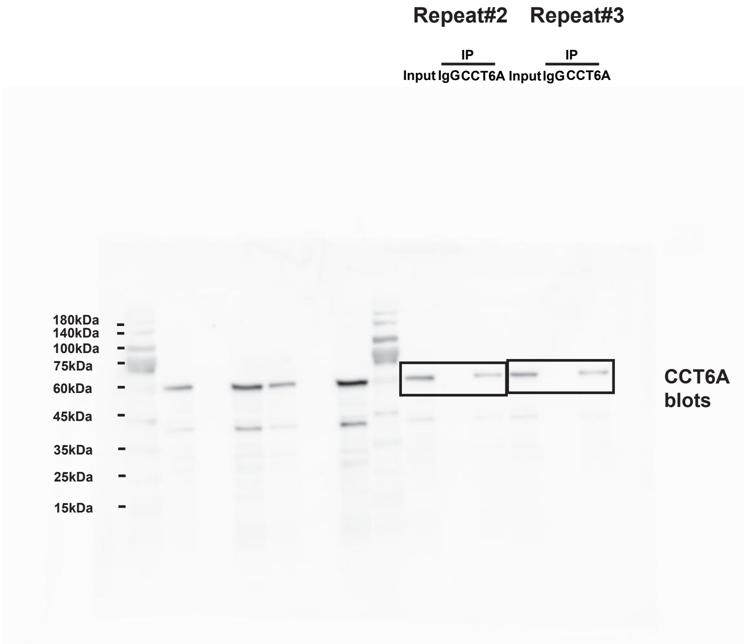


AsPC-1

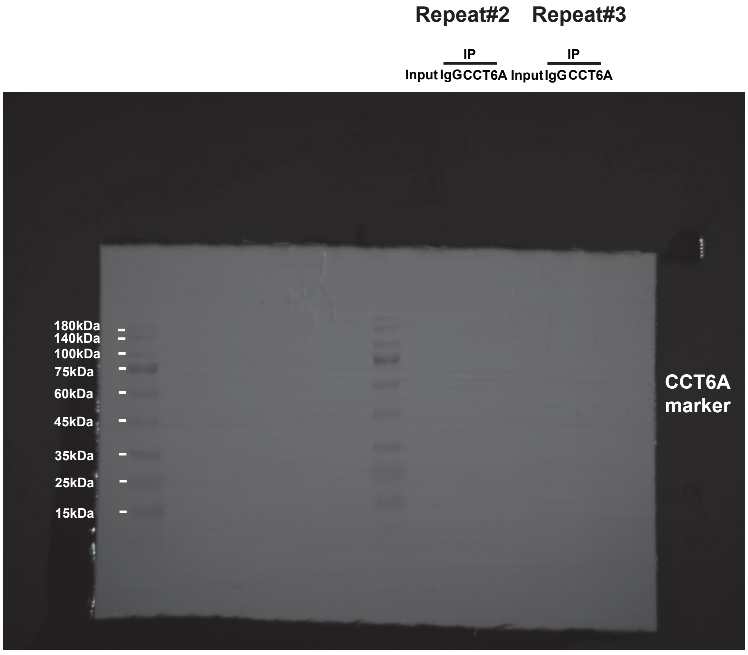


AsPC-1

**Repeat#1**

IP

Input IgG CCT6A

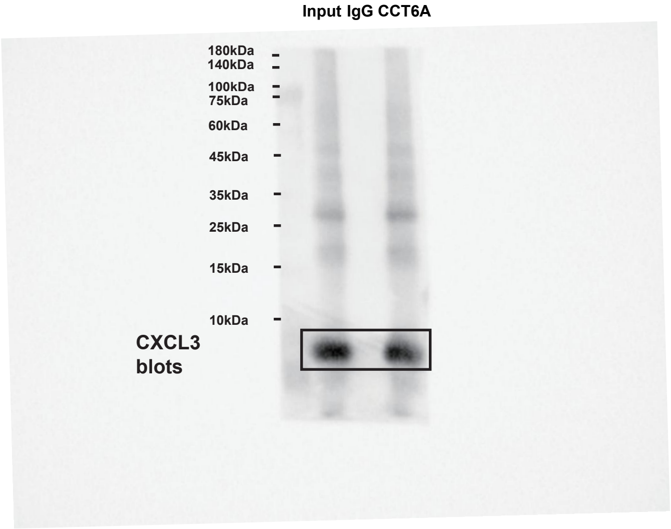


AsPC-1

IP

Input IgG CCT6A

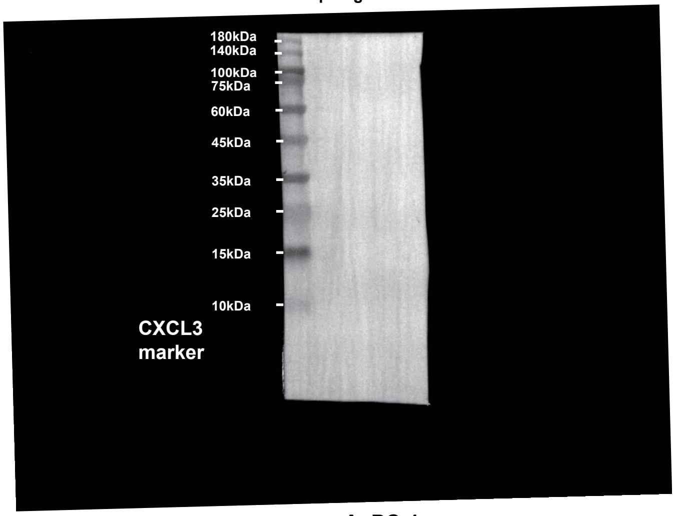


AsPC-1

Repeat#2 Repeat#3

IP IP

Input IgG CCT6A Input IgG CCT6A

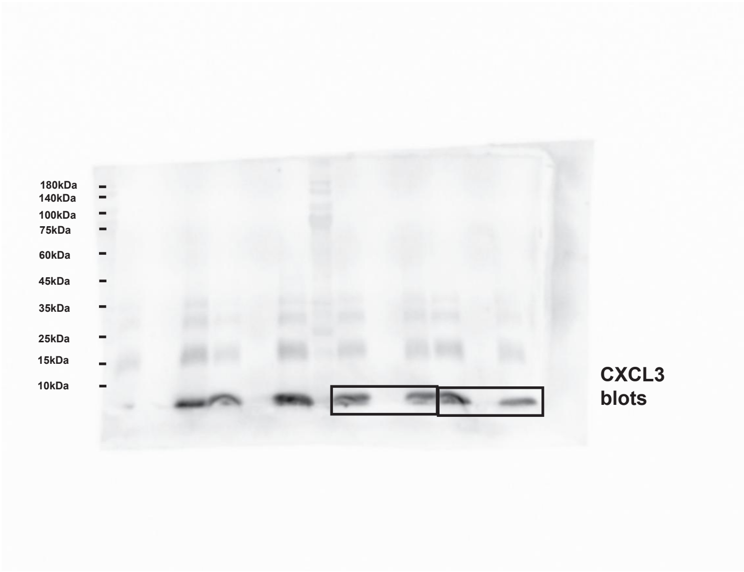


CXCL3
blots

AsPC-1

Repeat#2 Repeat#3

IP IP

Input IgG CCT6A Input IgG CCT6A

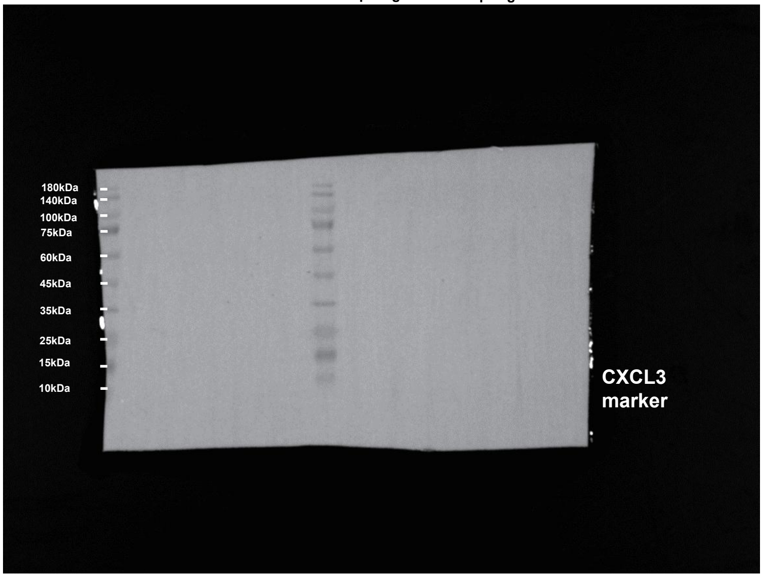


AsPC-1

**Fig. 6D**

Repeat#1

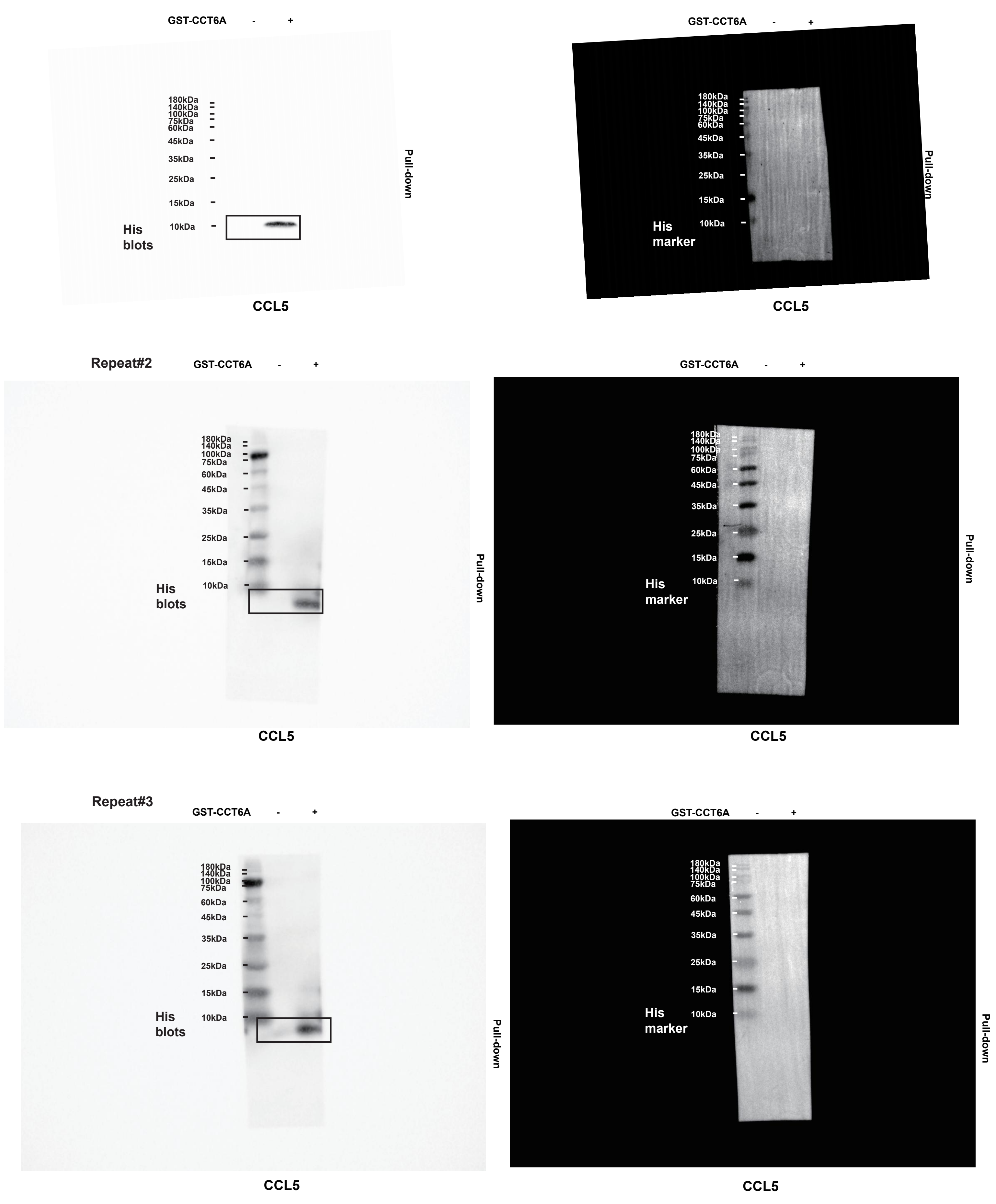

Repeat#1

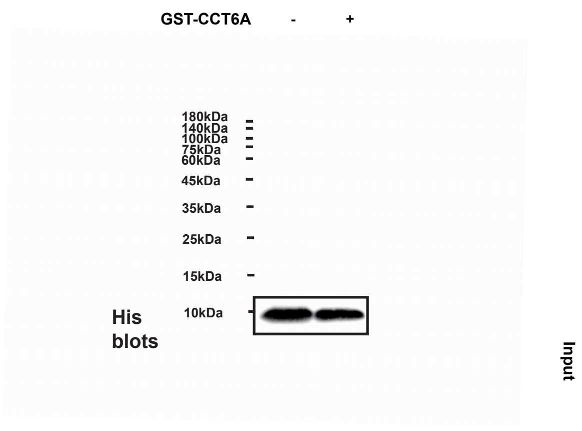


CCL5

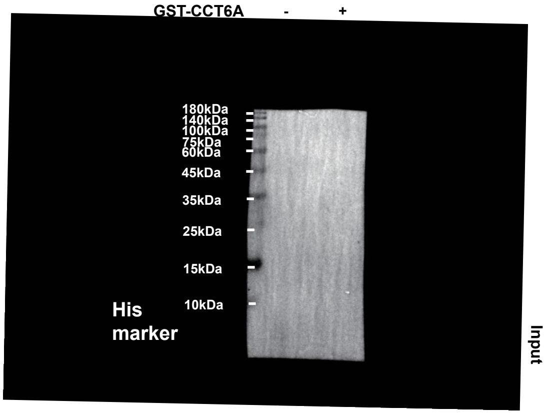


CCL5

Repeat#2

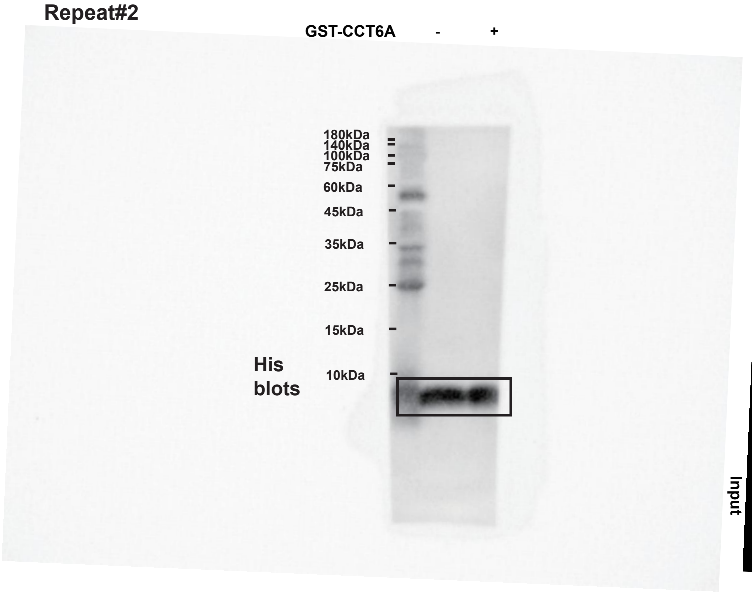


CCL5

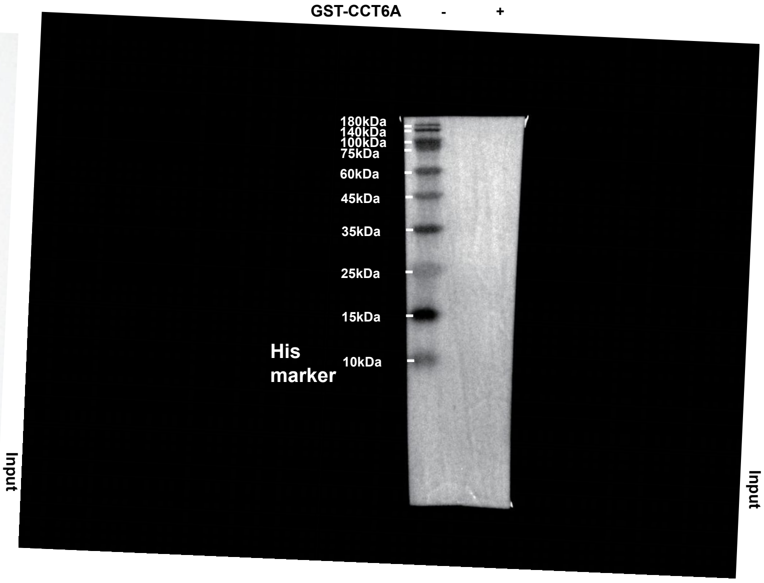


CCL5

Repeat#3

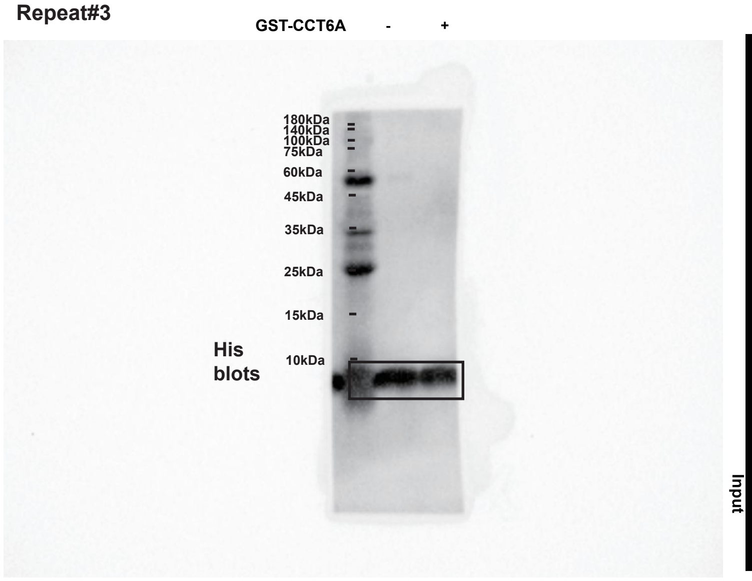


CCL5

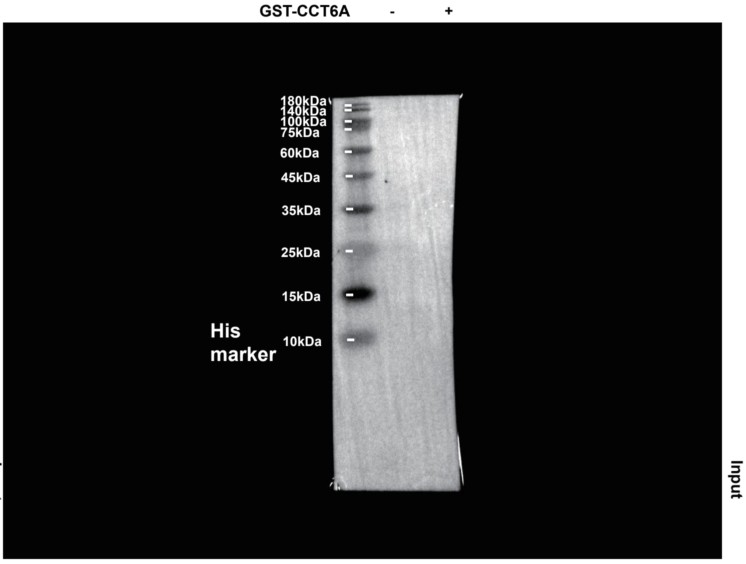


CCL5

Repeat#1

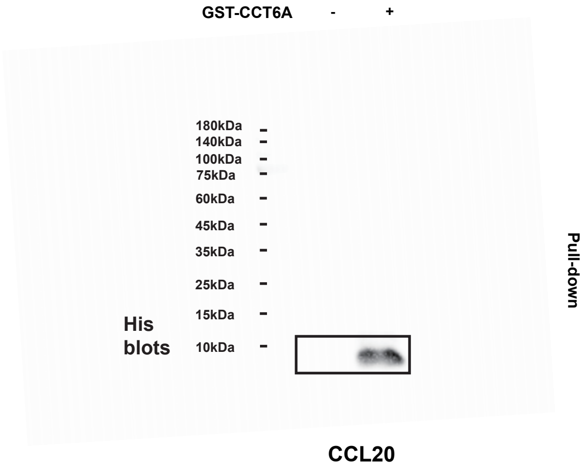


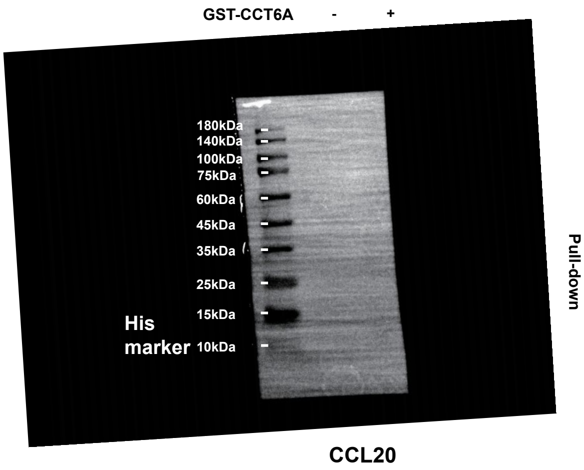


Repeat#2

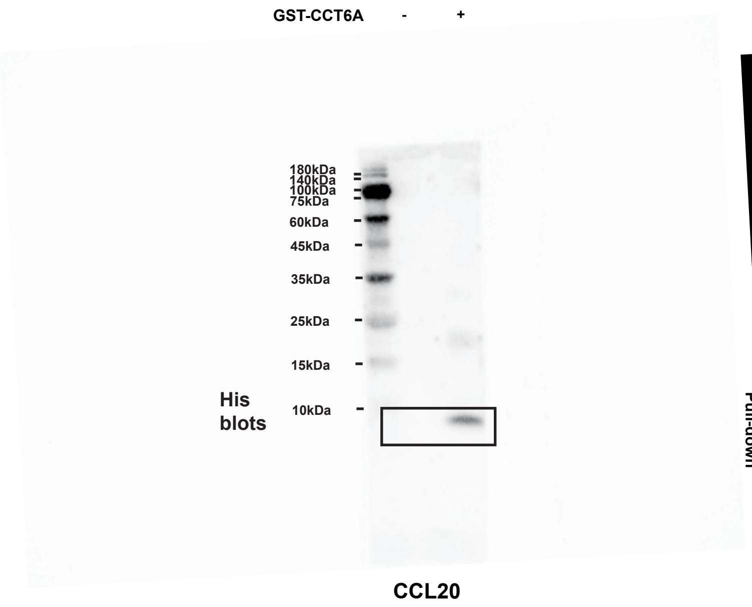


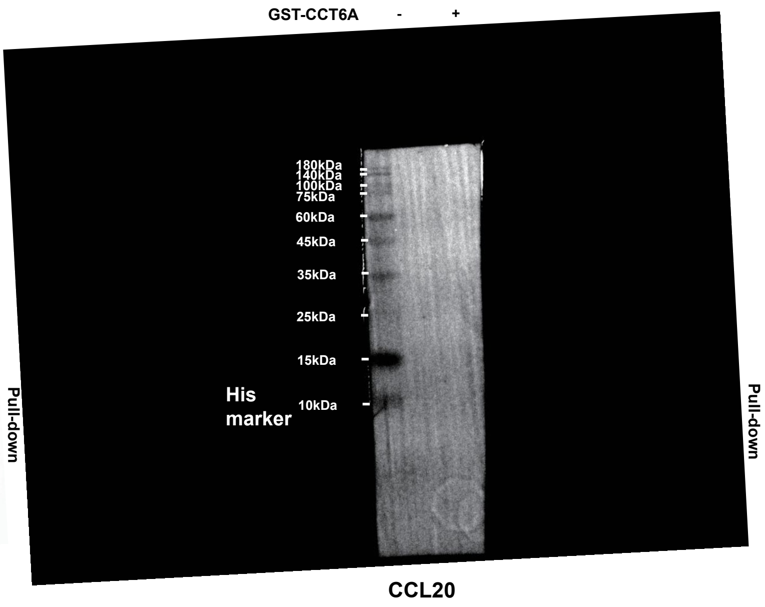


Repeat#3

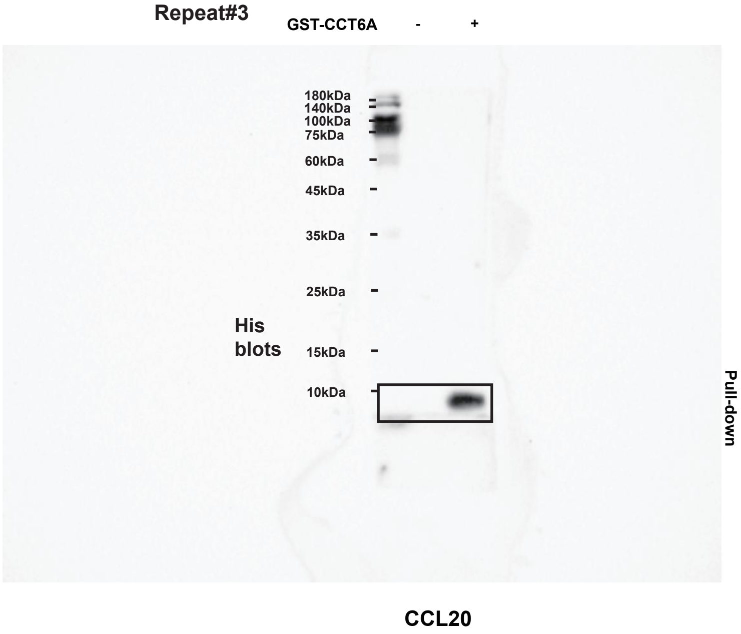


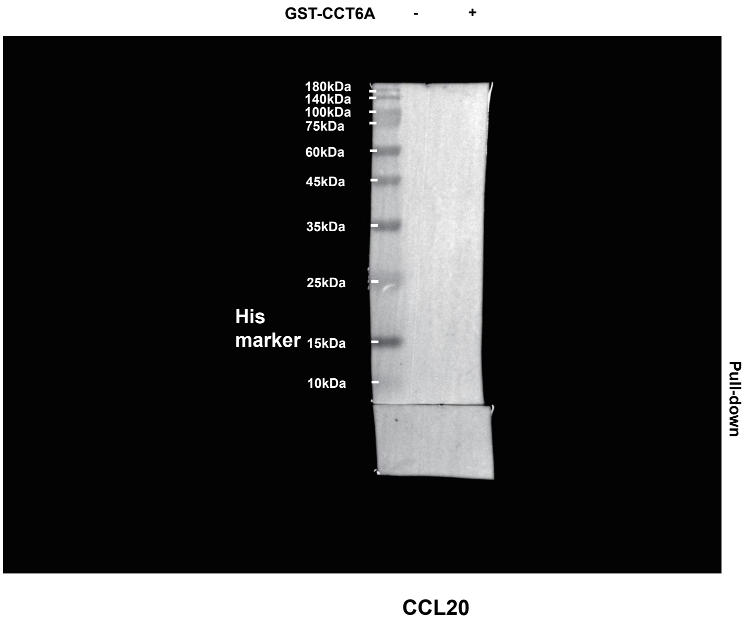

Repeat#1

GST-CCT6A - +

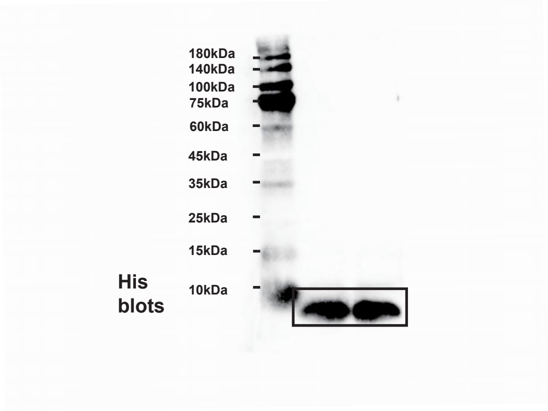


CCL20

Input

GST-CCT6A - +

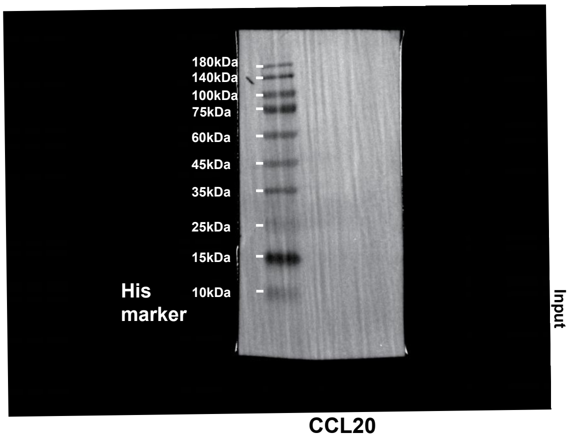


CCL20

Input

Repeat#2

GST-CCT6A - +

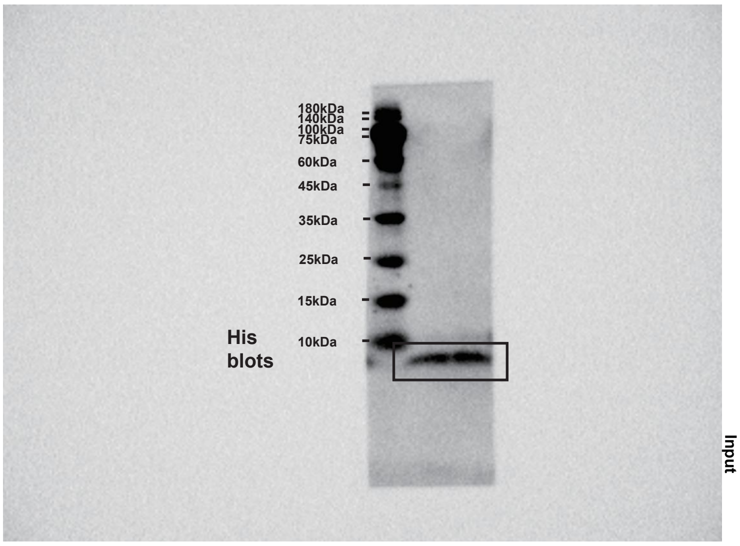


CCL20

Input

GST-CCT6A - +

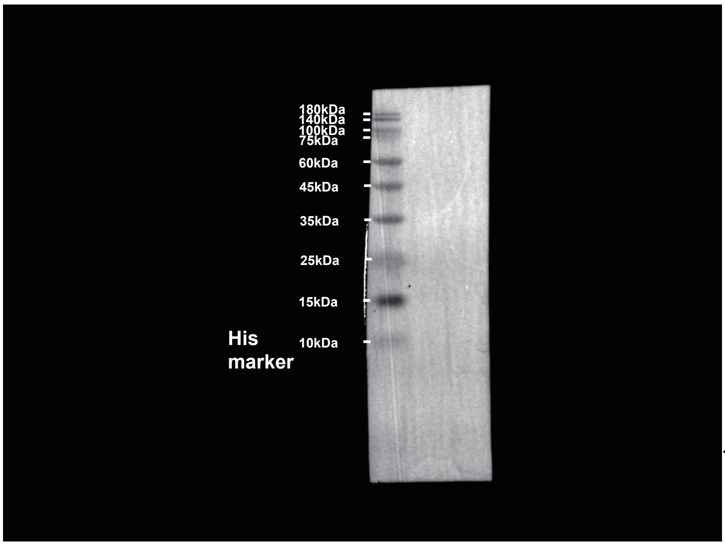


CCL20

Input

Repeat#3

GST-CCT6A - +

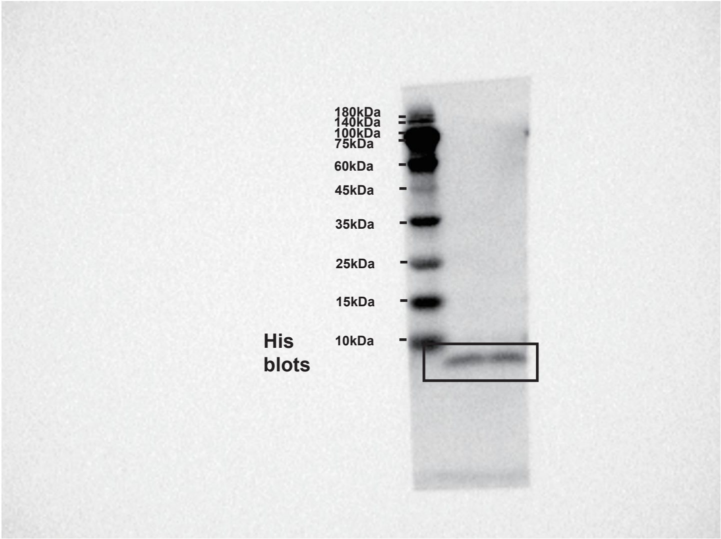


CCL20

Input

GST-CCT6A - +

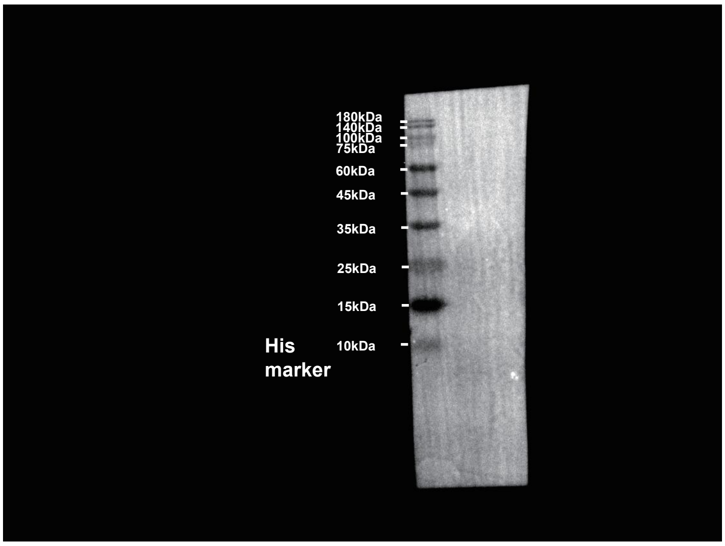


CCL20

Input

Repeat#1

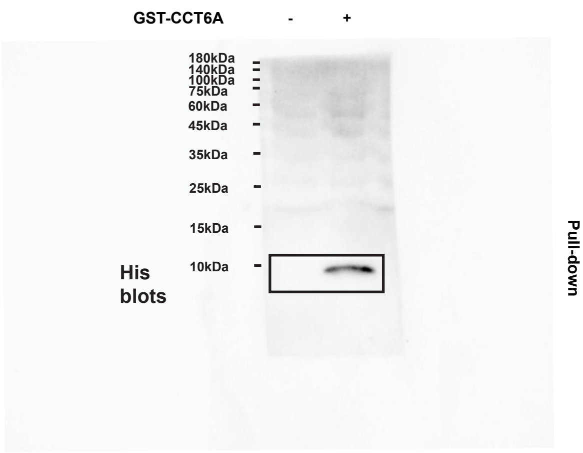


CXCL1

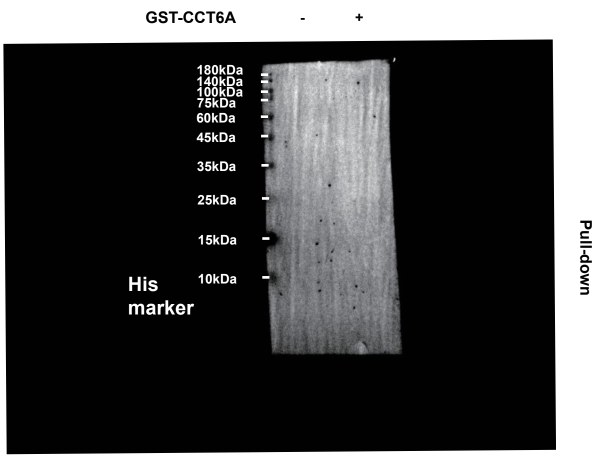


CXCL1

Repeat#2

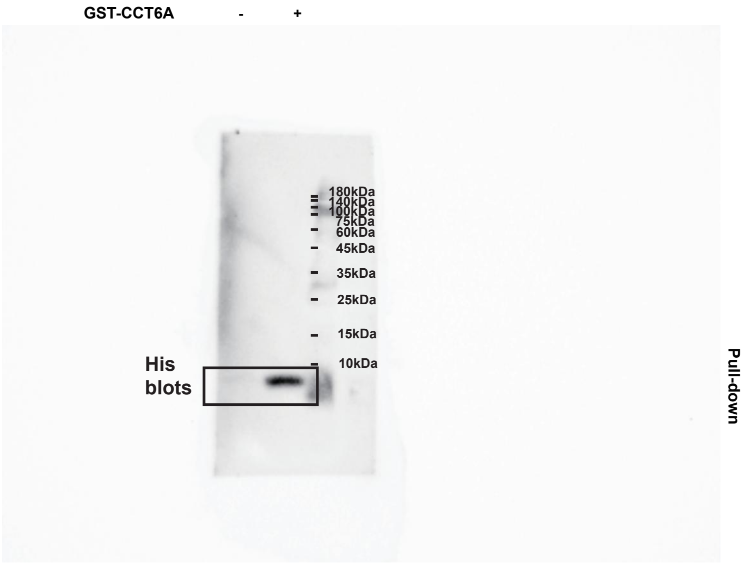


CXCL1

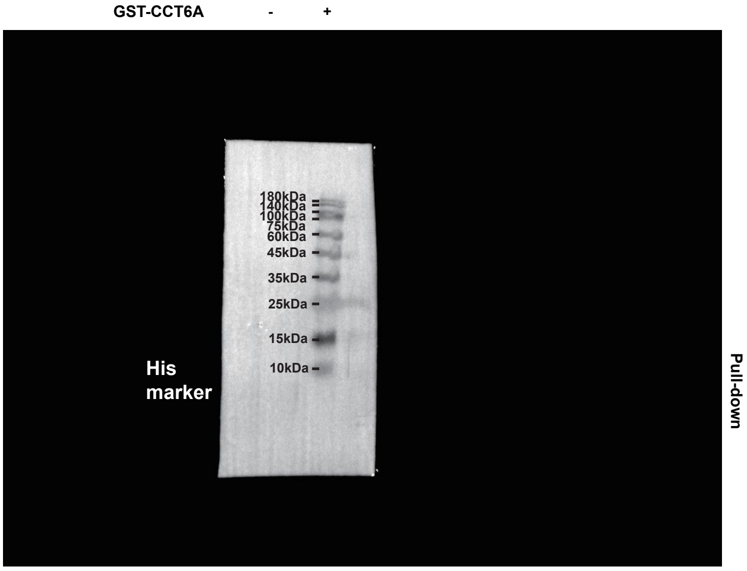


CXCL1

Repeat#3

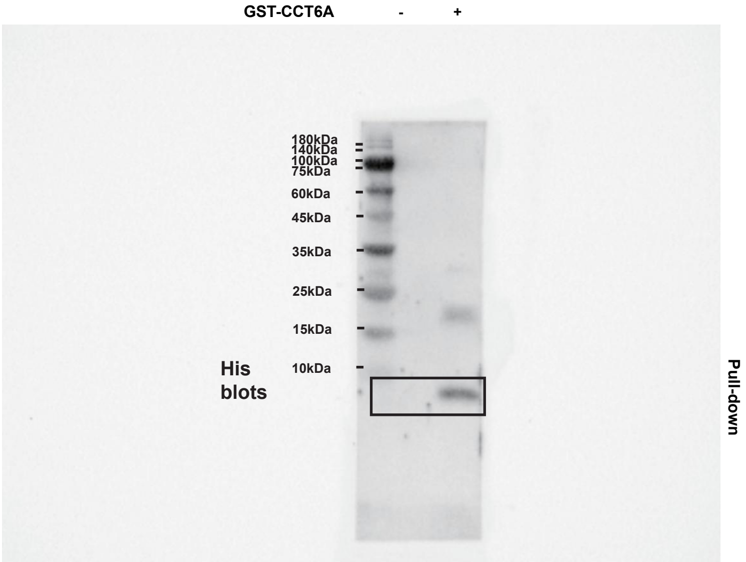


CXCL1

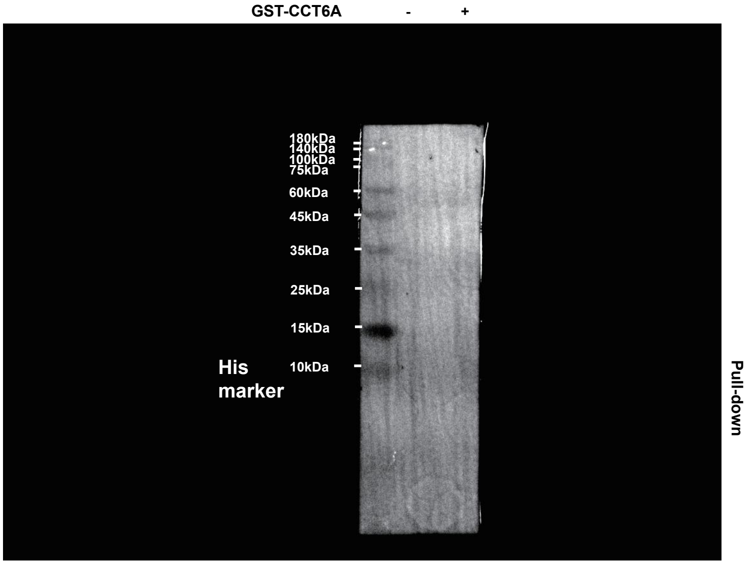


CXCL1

Repeat#1

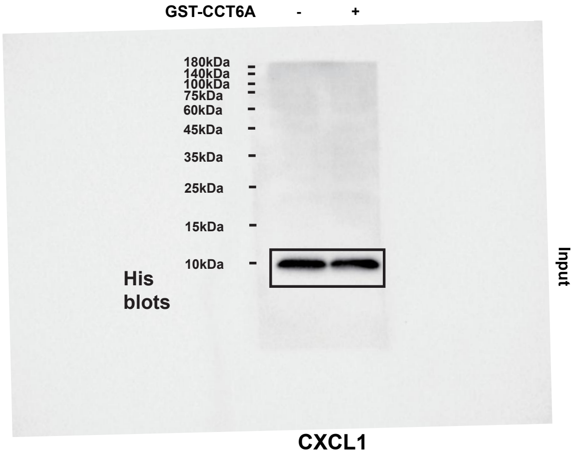


CXCL1

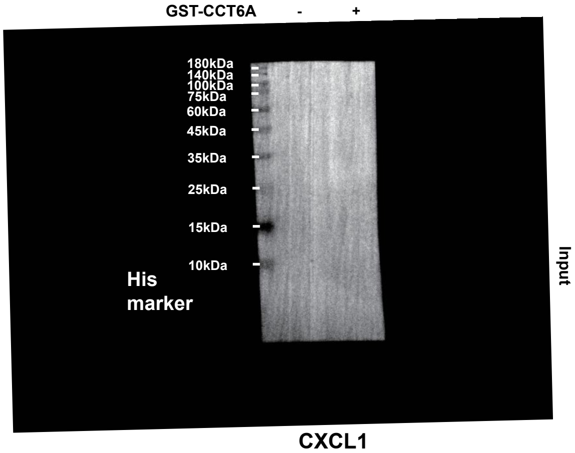


CXCL1

Repeat#2

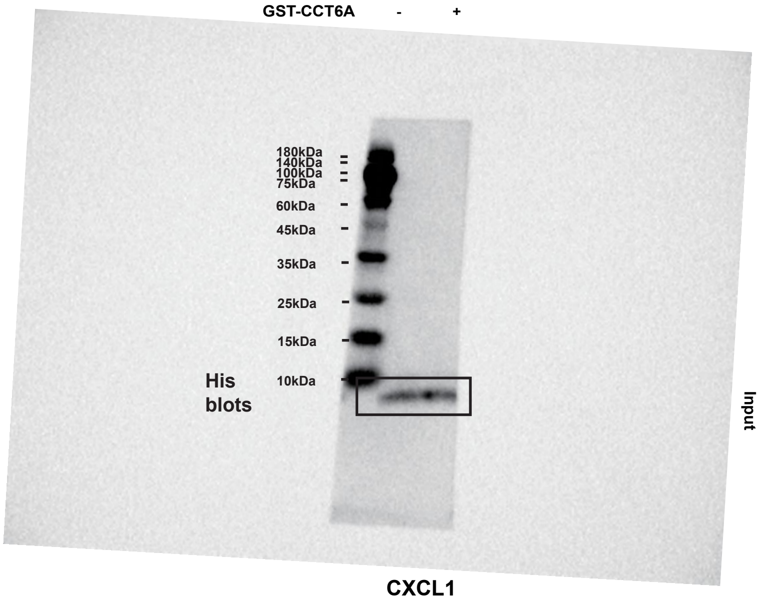


CXCL1

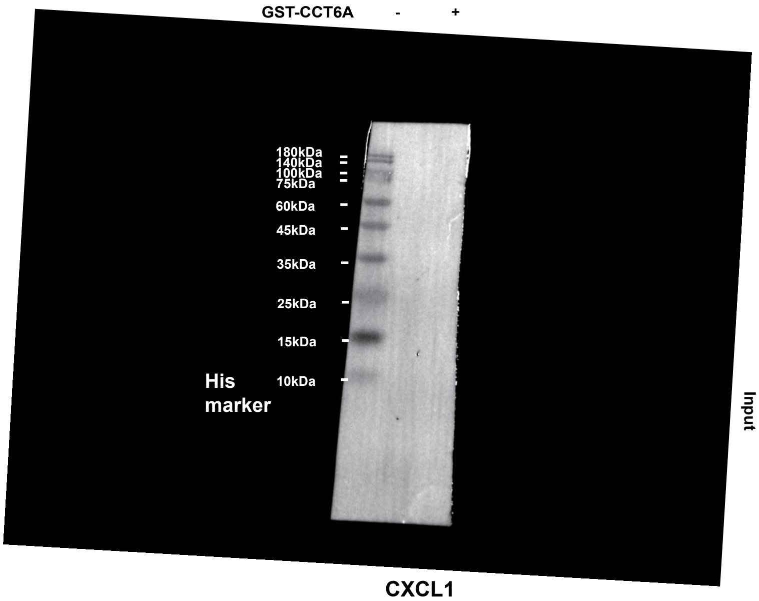


CXCL1

Repeat#3

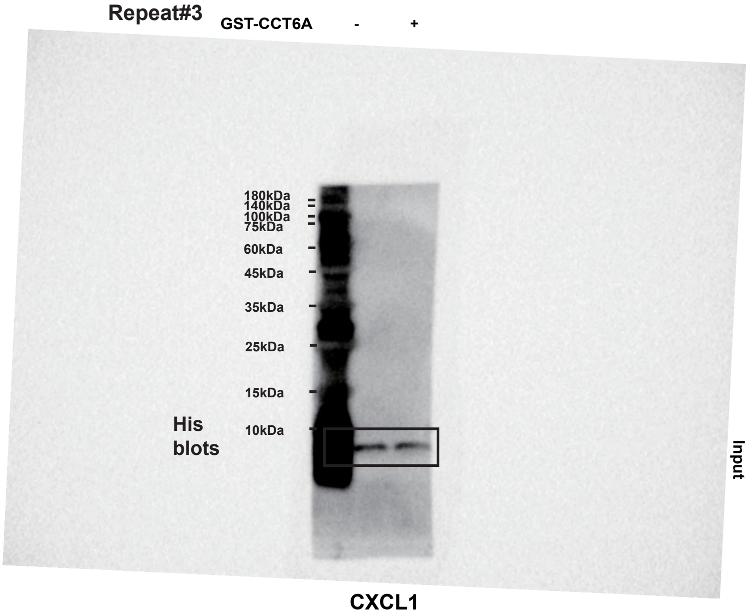


CXCL1

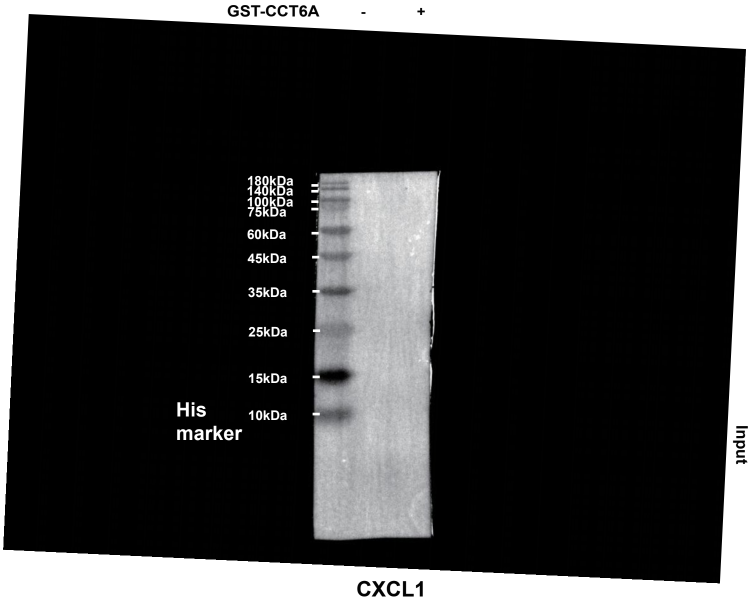


CXCL1

**Repeat#1**

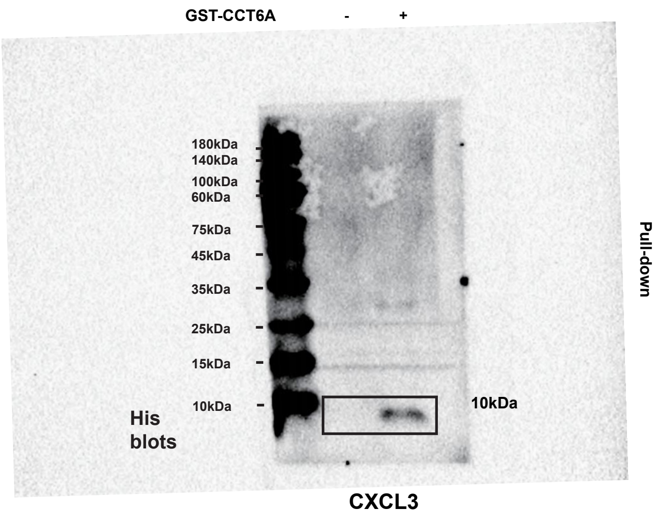


CXCL3

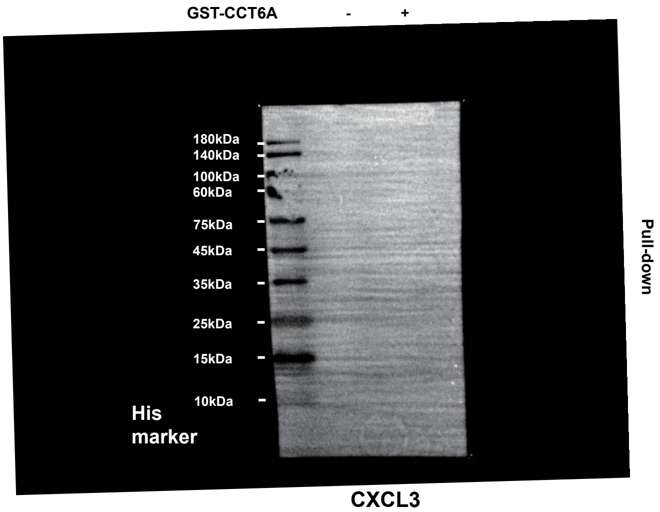


CXCL3

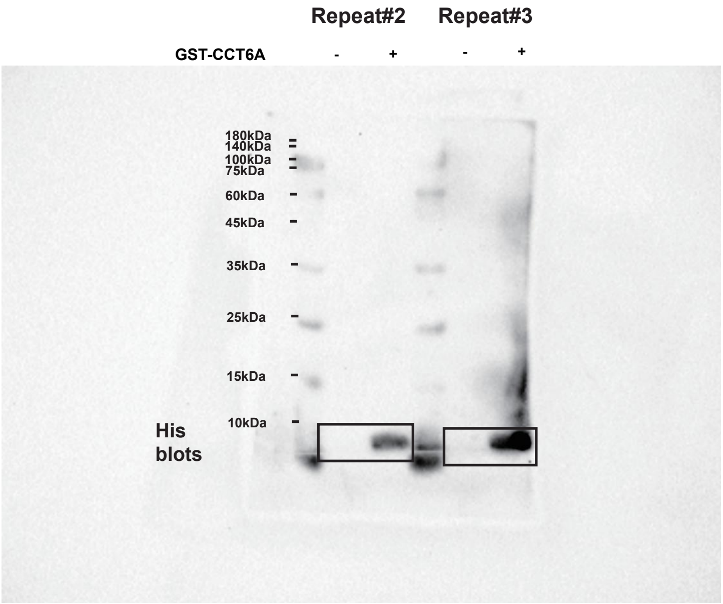


CXCL3

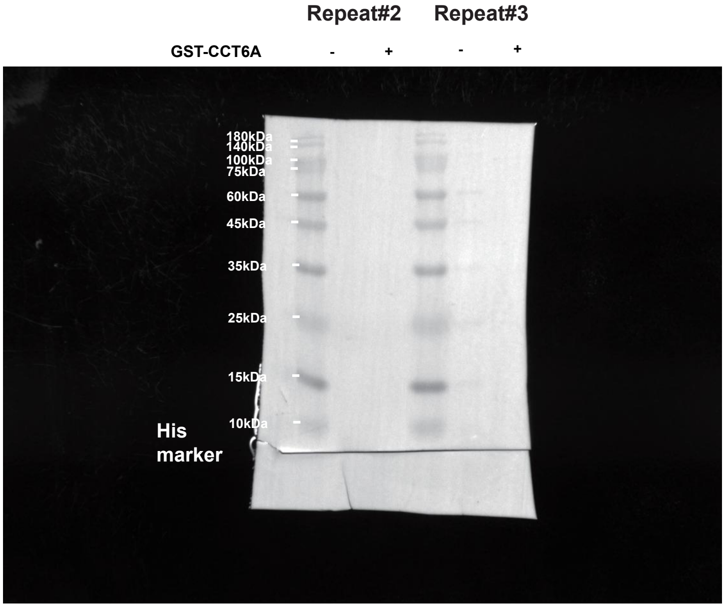


CXCL3

## Repeat#1

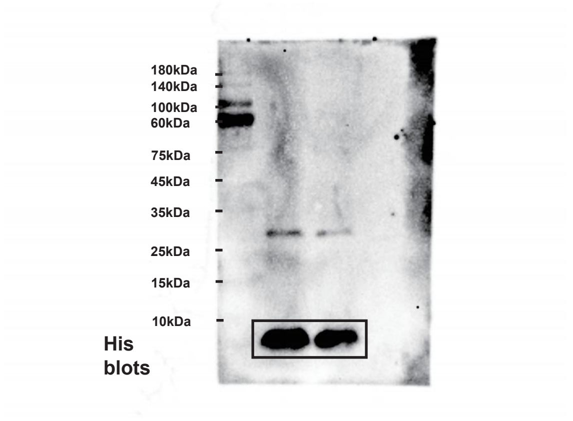


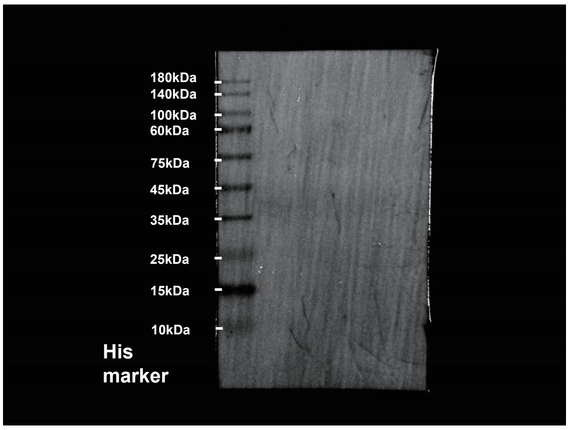


## Repeat#2

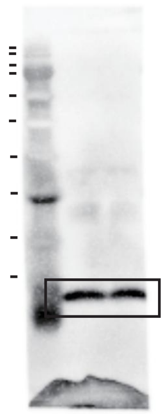


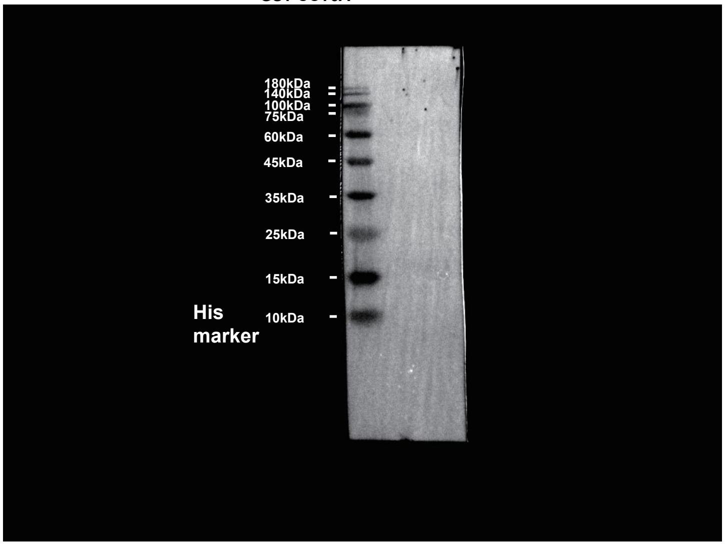


## Repeat#3

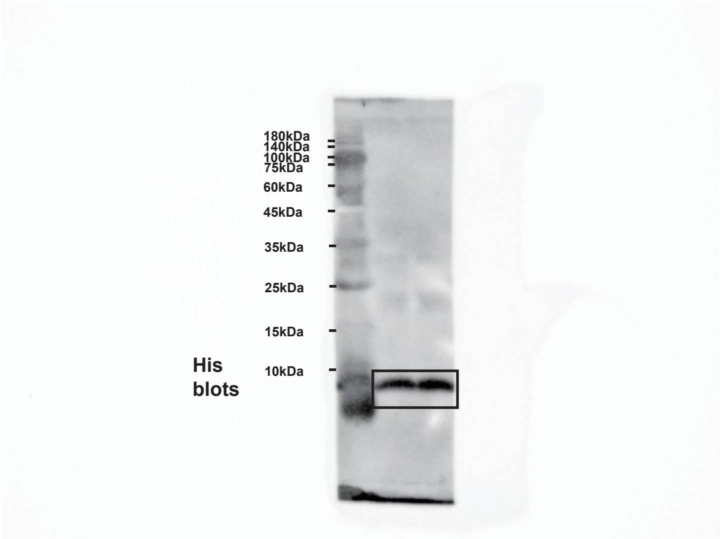


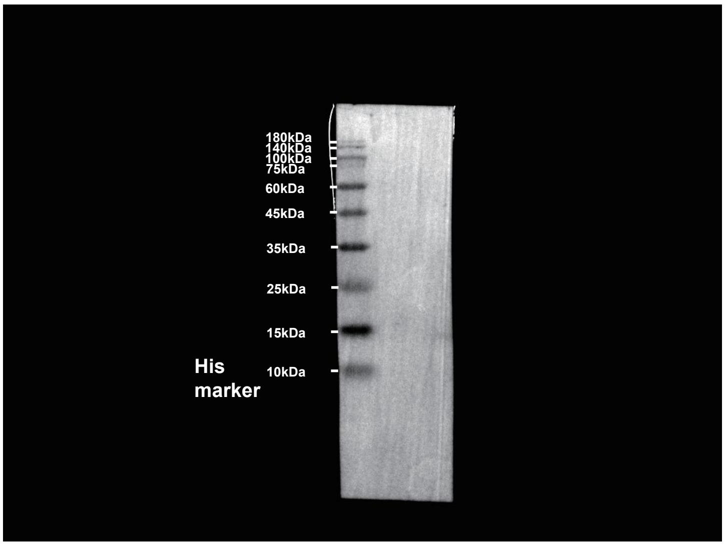

Fig. S5A

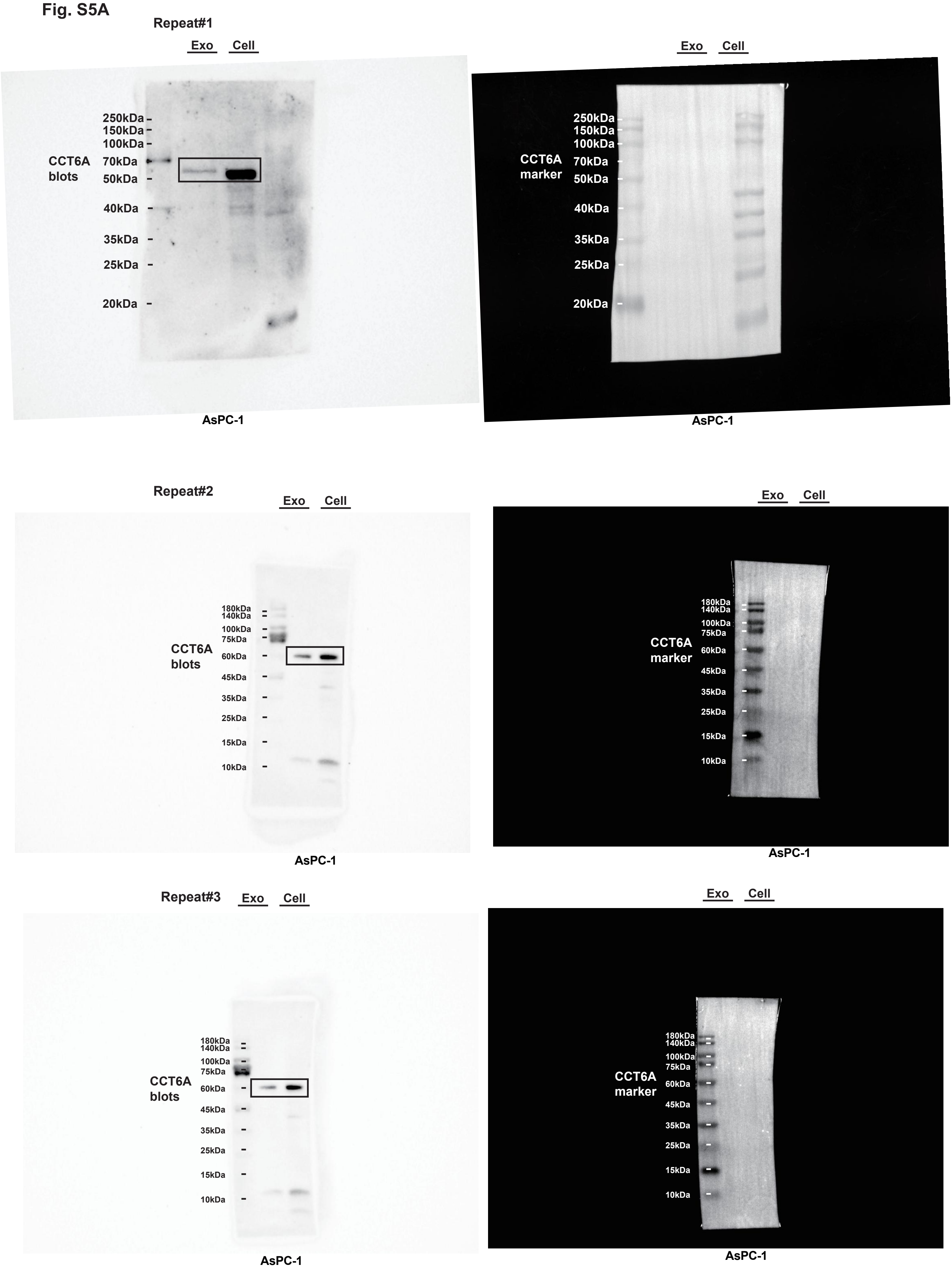
Repeat#1
Exo Cell
250kDa
150kDa
100kDa
CCT6A blots
70kDa
50kDa
40kDa
35kDa
25kDa
20kDa
AsPC-1
Exo Cell
CCT6A marker
250kDa
150kDa
100kDa
70kDa
50kDa
40kDa
35kDa
25kDa
20kDa
AsPC-1
Repeat#2
Exo Cell
CCT6A blots
180kDa
140kDa
100kDa
75kDa
60kDa
45kDa
35kDa
25kDa
15kDa
10kDa
AsPC-1
Exo Cell
CCT6A marker
180kDa
140kDa
100kDa
75kDa
60kDa
45kDa
35kDa
25kDa
15kDa
10kDa
AsPC-1
Repeat#3
Exo Cell
CCT6A blots
180kDa
140kDa
100kDa
75kDa
60kDa
45kDa
35kDa
25kDa
15kDa
10kDa
AsPC-1
Exo Cell
CCT6A marker
180kDa
140kDa
100kDa
75kDa
60kDa
45kDa
35kDa
25kDa
15kDa
10kDa
AsPC-1

**Repeat#1**

Exo Cell

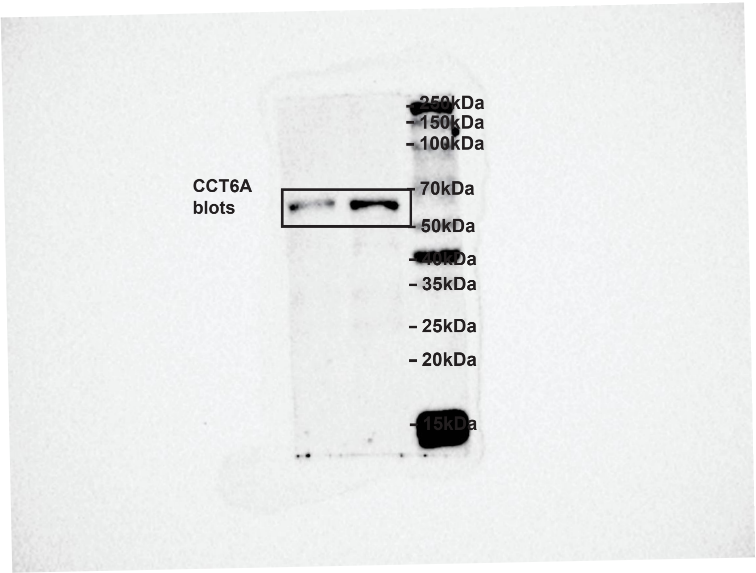


BxPC-3

Exo Cell

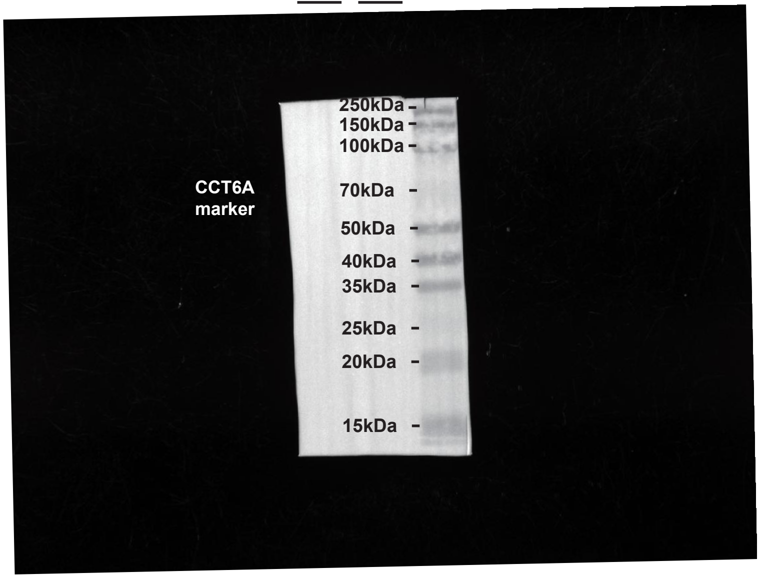


BxPC-3

**Repeat#2**

Exo Cell

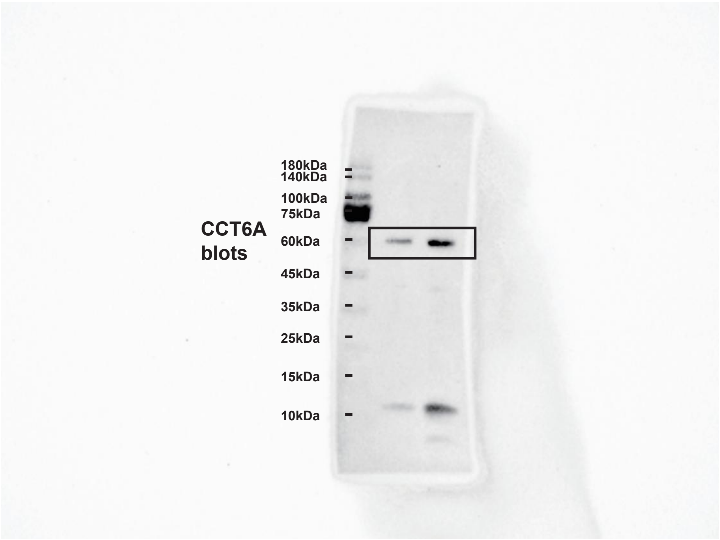


BxPC-3

Exo Cell

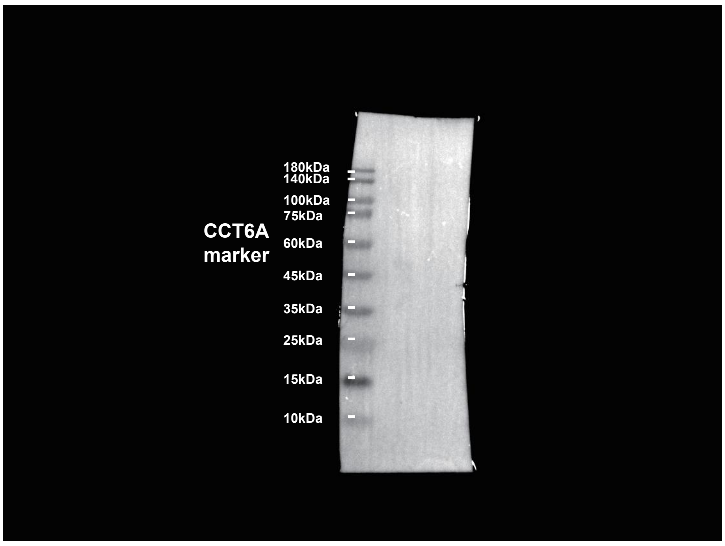


BxPC-3

**Repeat#3**

Exo Cell

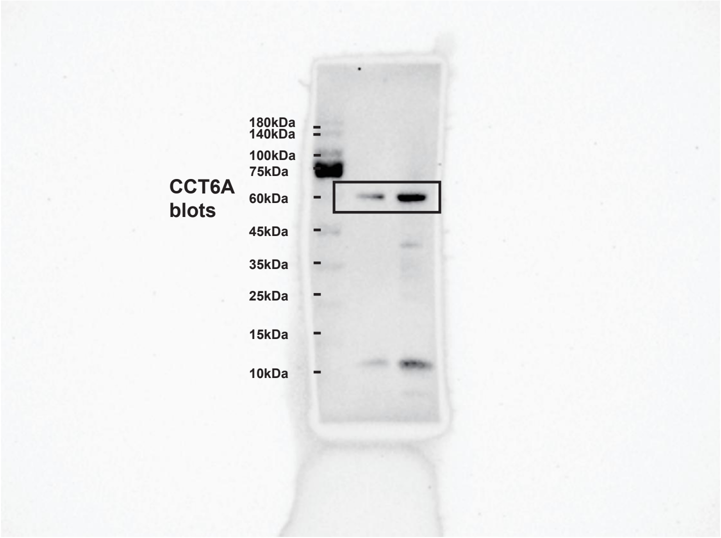


BxPC-3

Exo Cell

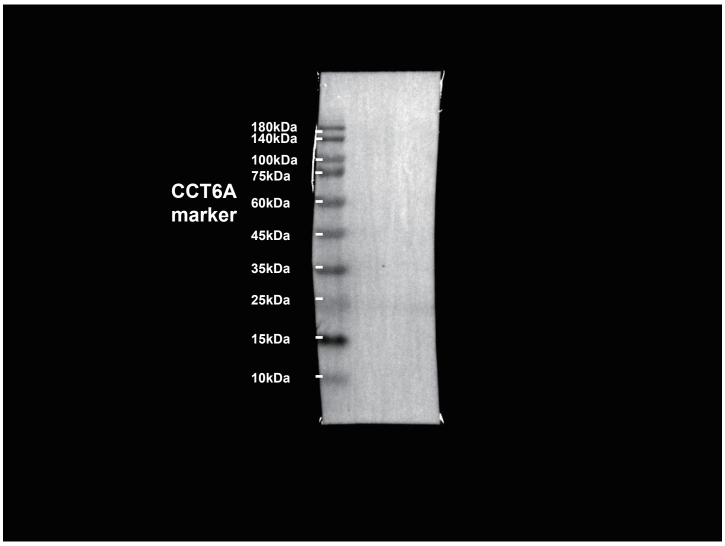


BxPC-3

## Fig. S6A

Repeat#1

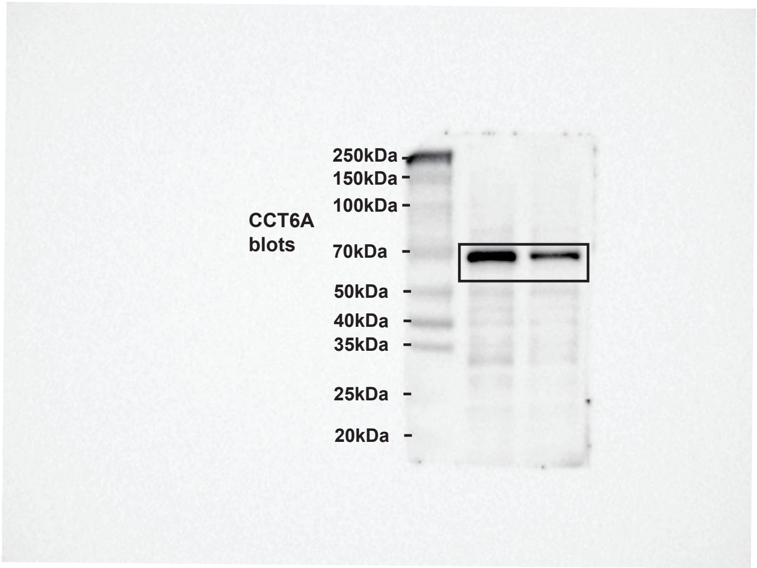


AsPC-1

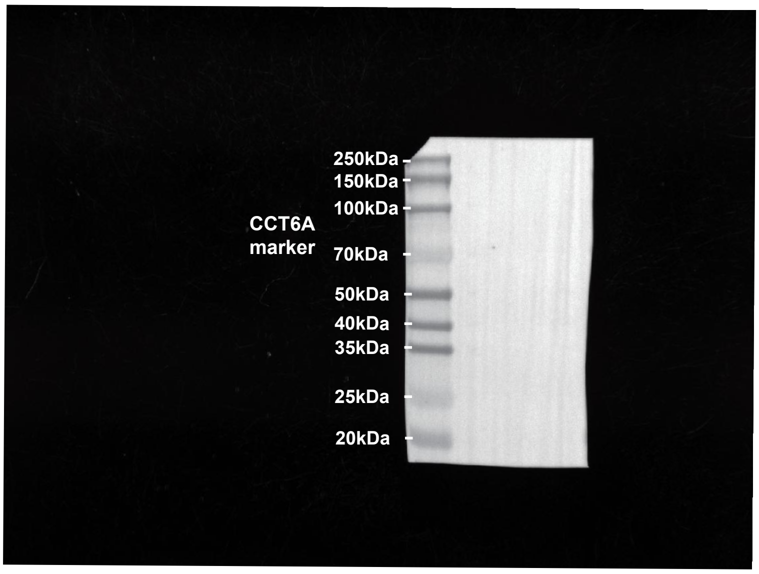


AsPC-1

Repeat#2

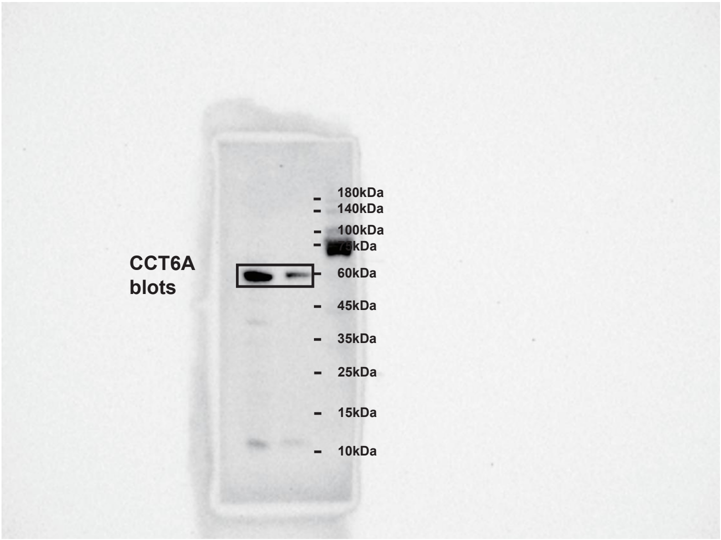


AsPC-1

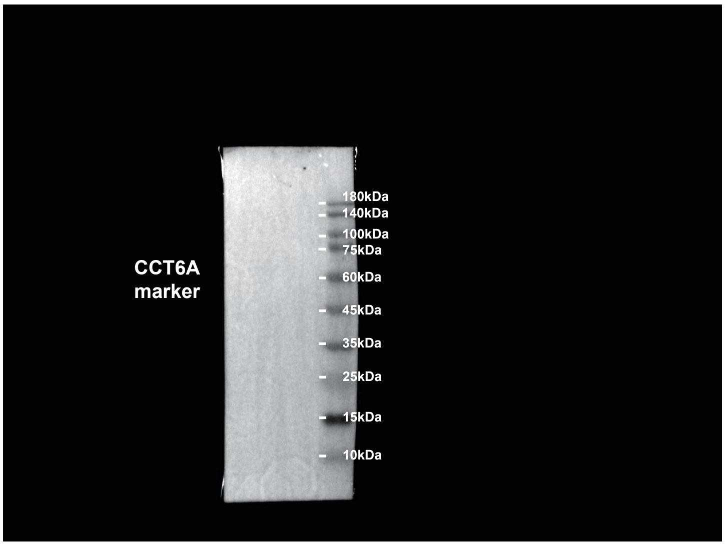


AsPC-1

Repeat#3

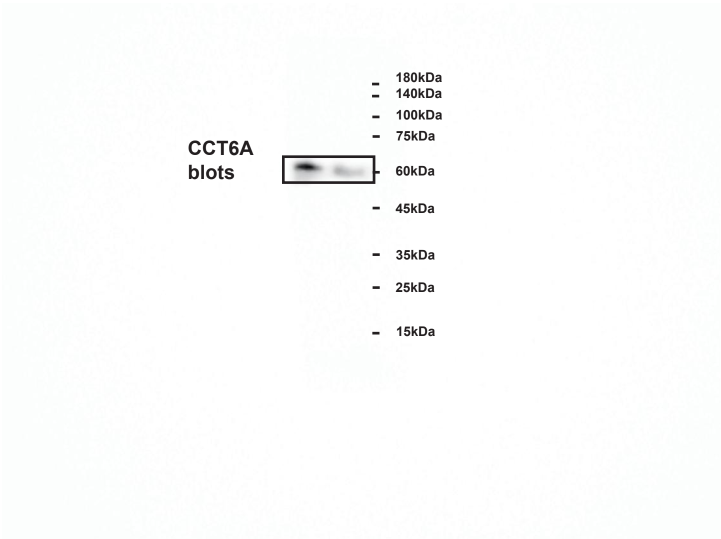


AsPC-1

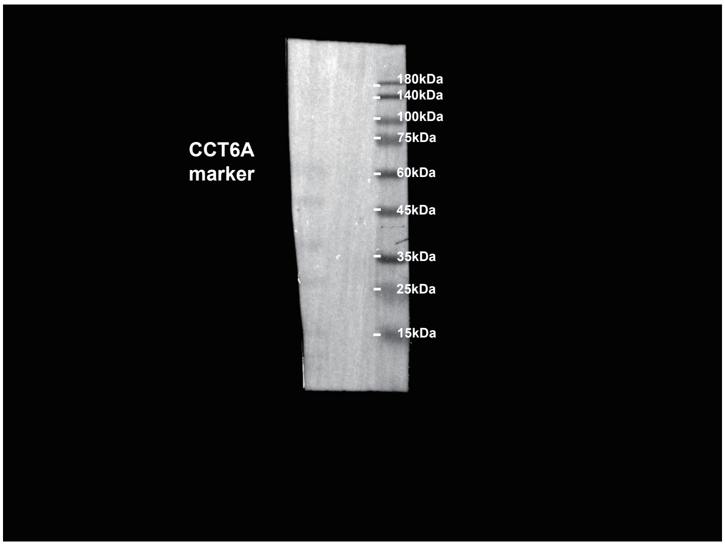


AsPC-1

**Repeat#1**

sh-NC sh-CCT6A

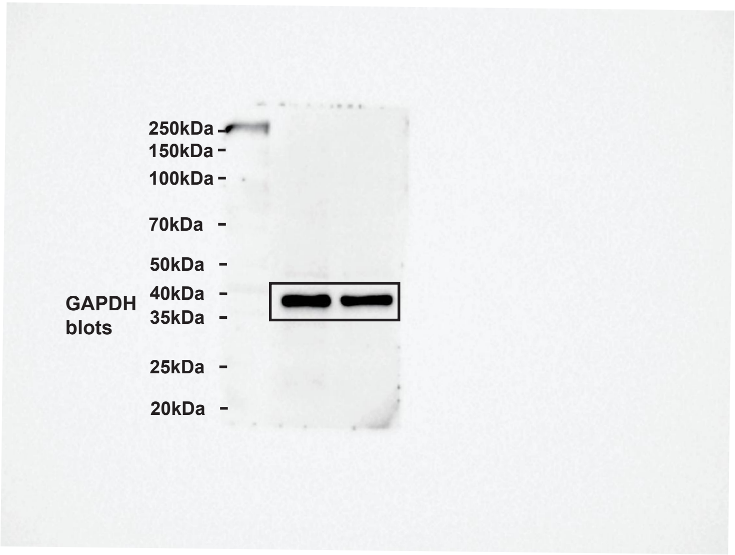


AsPC-1

sh-NC sh-CCT6A

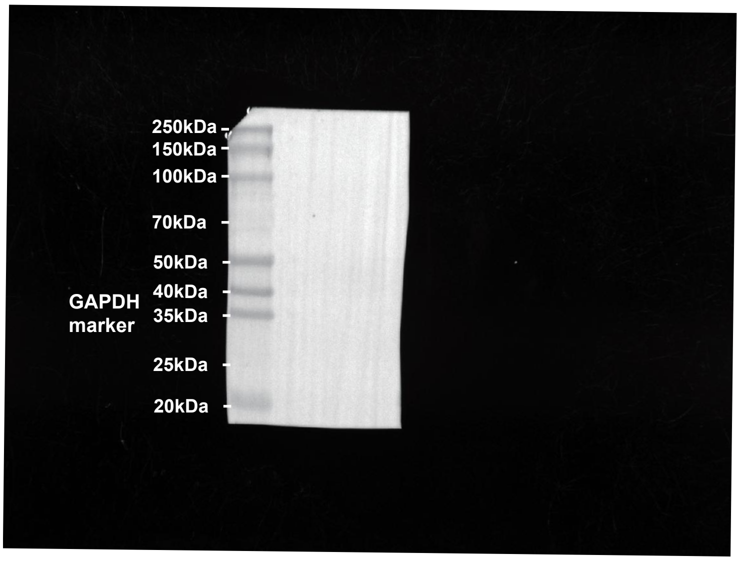


AsPC-1

**Repeat#2**

sh-NC sh-CCT6A

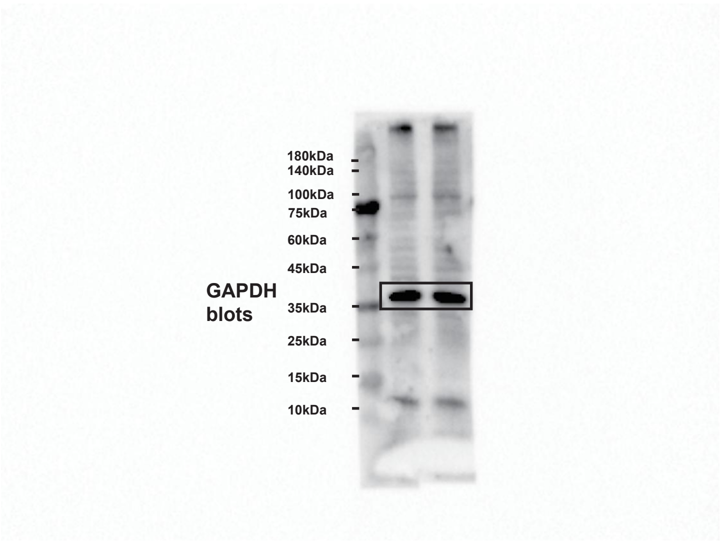


AsPC-1

sh-NC sh-CCT6A

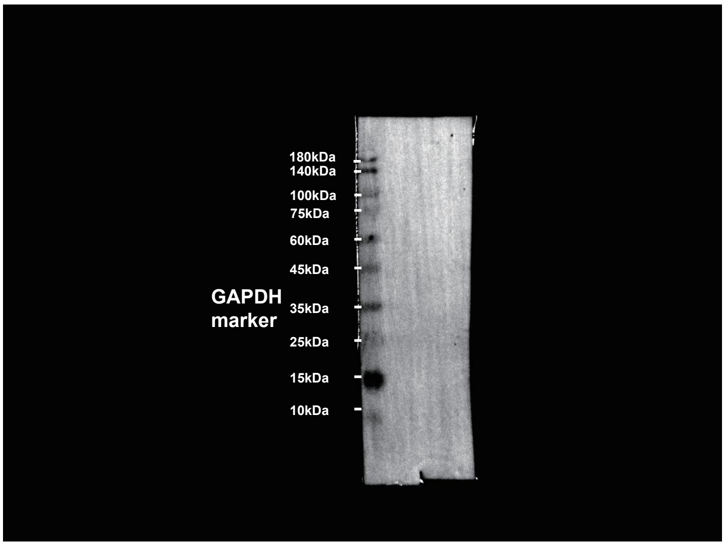


AsPC-1

**Repeat#3**

sh-NC sh-CCT6A

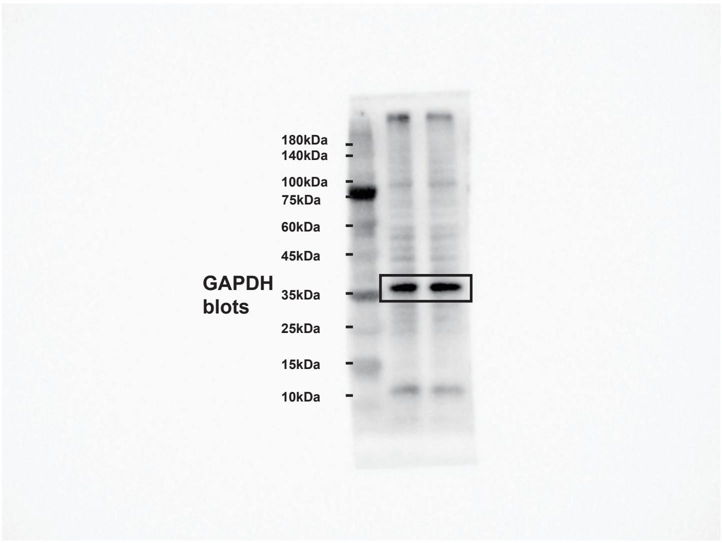


AsPC-1

sh-NC sh-CCT6A

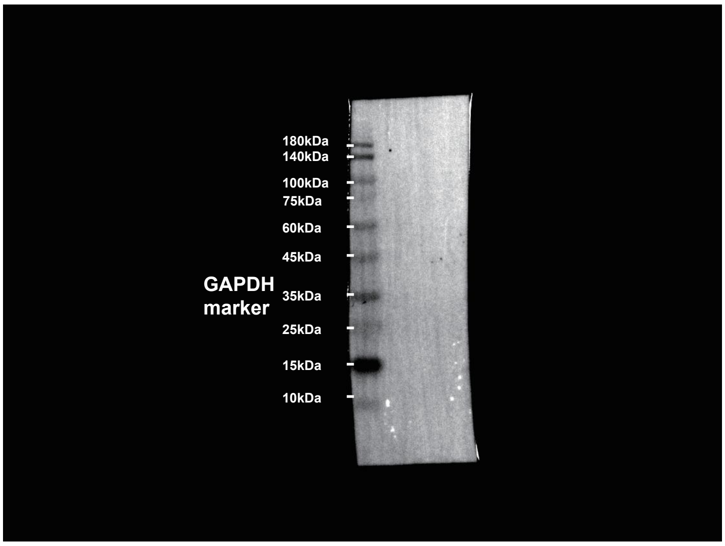


AsPC-1

Repeat#1

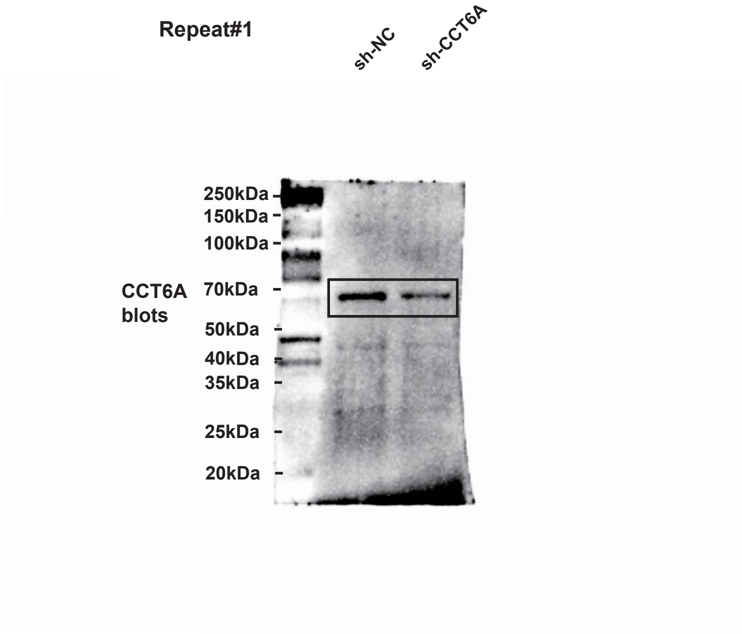


BxPC-3

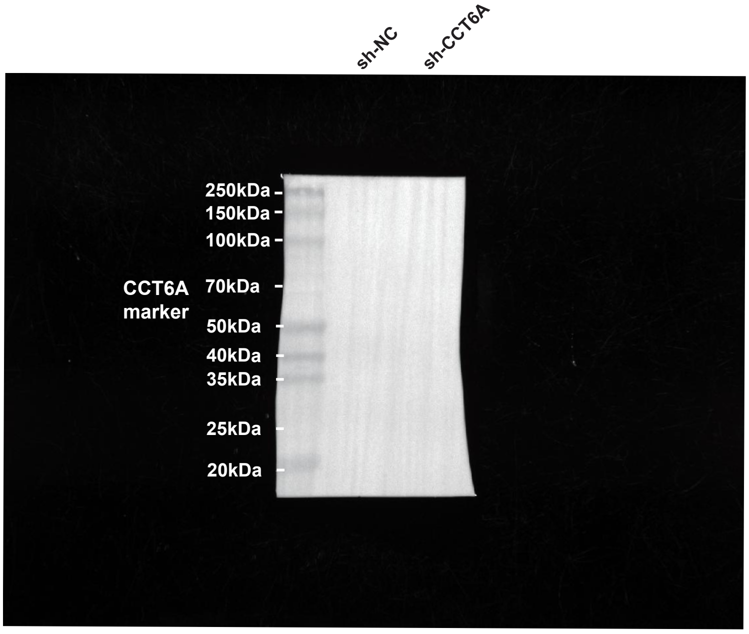


BxPC-3

Repeat#2

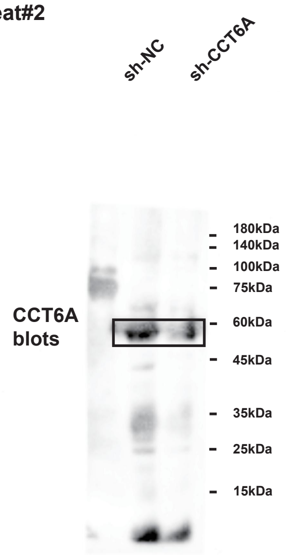


BxPC-3

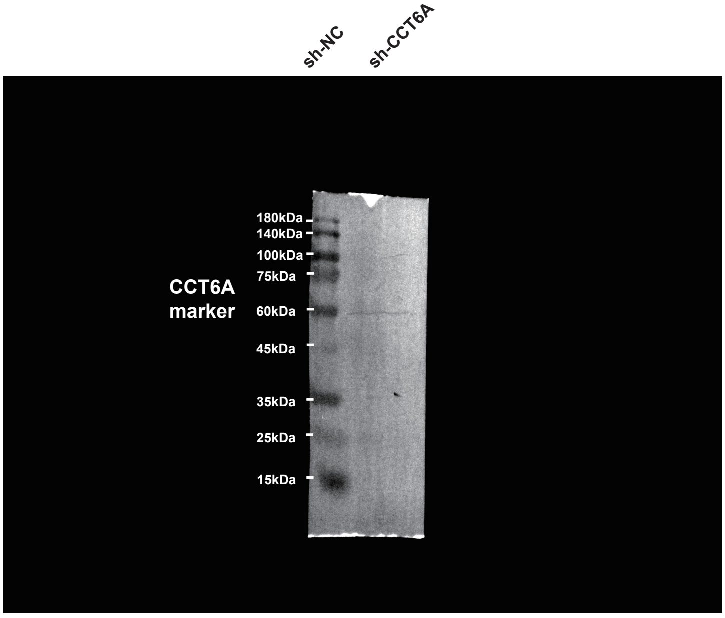


BxPC-3

Repeat#3

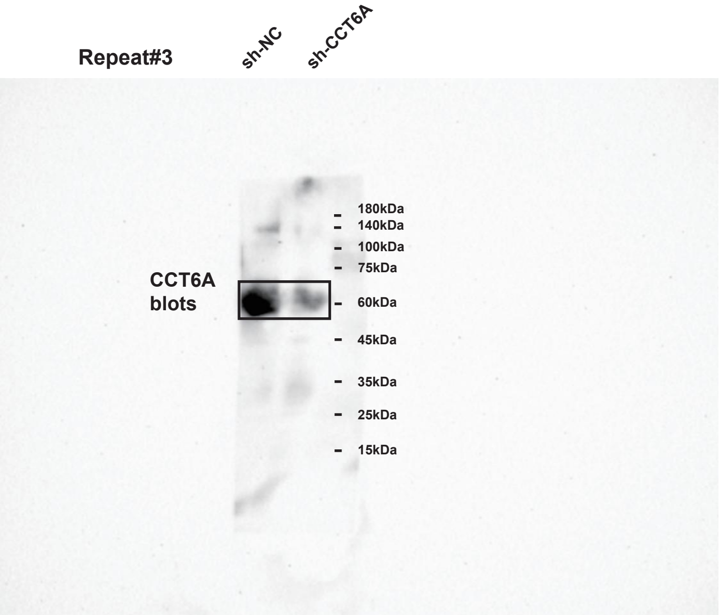


BxPC-3

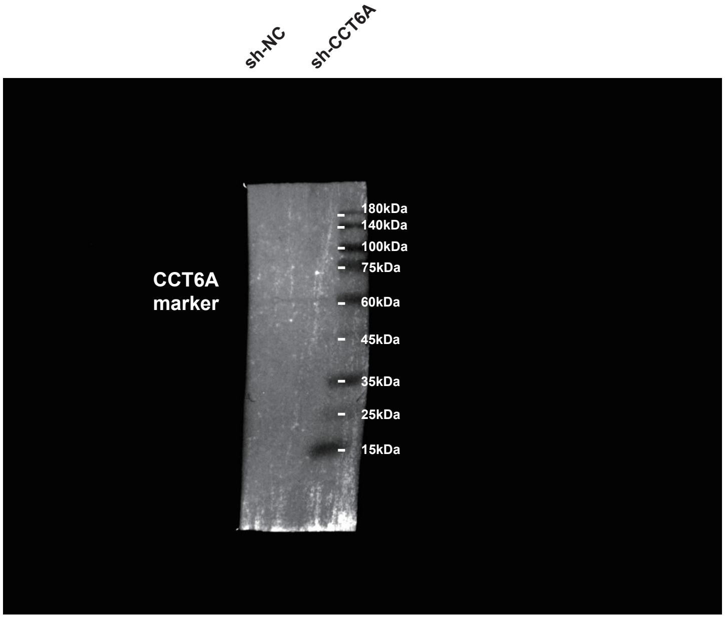


BxPC-3

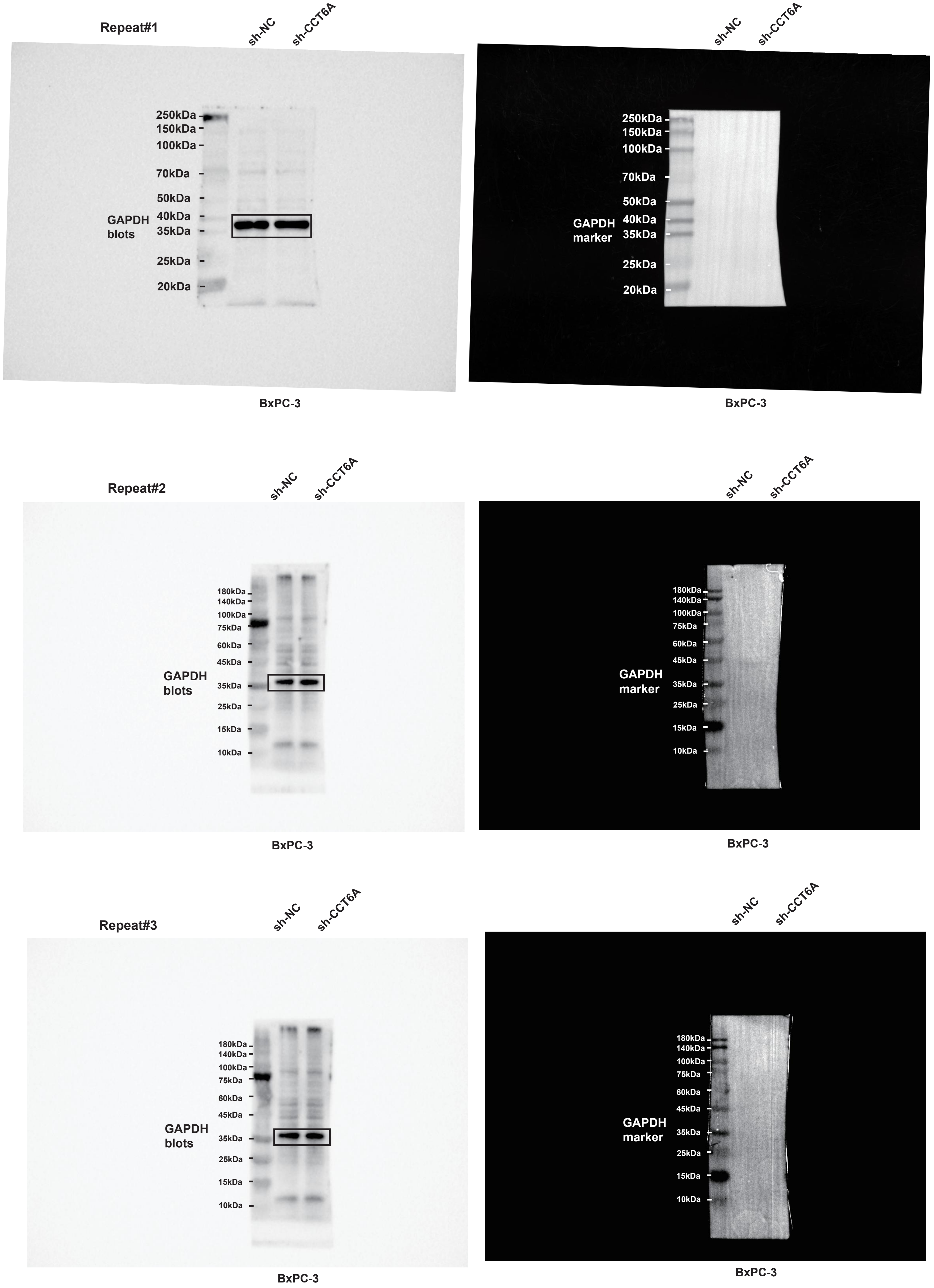
Repeat#1
sh-NC
sh-CCT6A
250kDa
150kDa
100kDa
70kDa
50kDa
40kDa
35kDa
25kDa
20kDa
GAPDH blots
GAPDH marker
BxPC-3
BxPC-3
Repeat#2
sh-NC
sh-CCT6A
180kDa
140kDa
100kDa
75kDa
60kDa
45kDa
35kDa
25kDa
15kDa
10kDa
GAPDH blots
GAPDH marker
BxPC-3
BxPC-3
Repeat#3
sh-NC
sh-CCT6A
180kDa
140kDa
100kDa
75kDa
60kDa
45kDa
35kDa
25kDa
15kDa
10kDa
GAPDH blots
GAPDH marker
BxPC-3
BxPC-3

**Fig. S8A**

**Repeat#1**

OE-NC OE-CCT6A

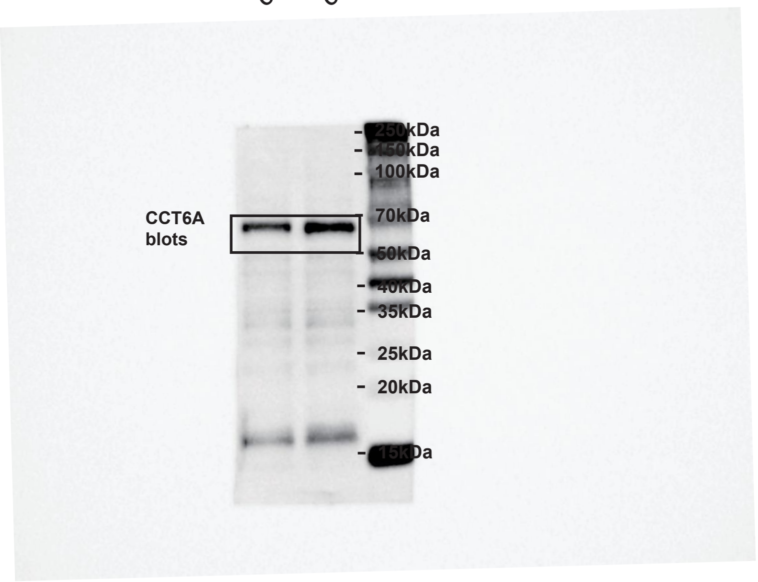


AsPC-1

OE-NC OE-CCT6A

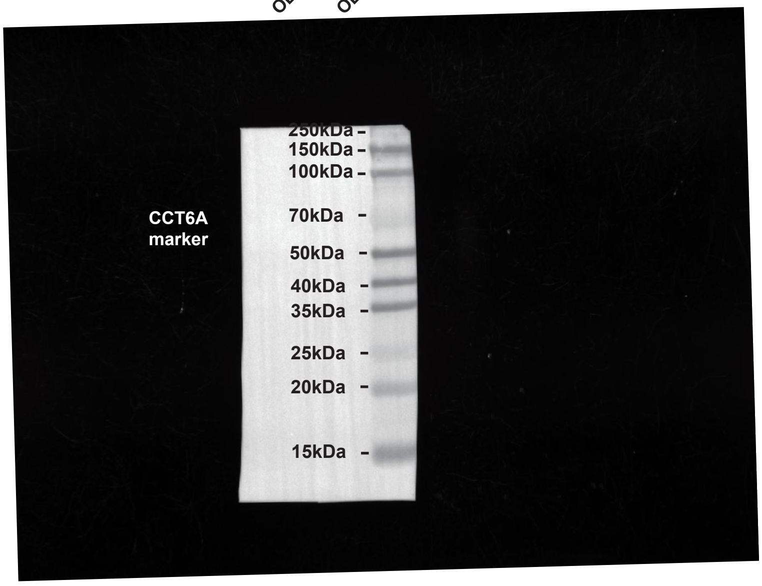


AsPC-1

**Repeat#2**

OE-NC OE-CCT6A

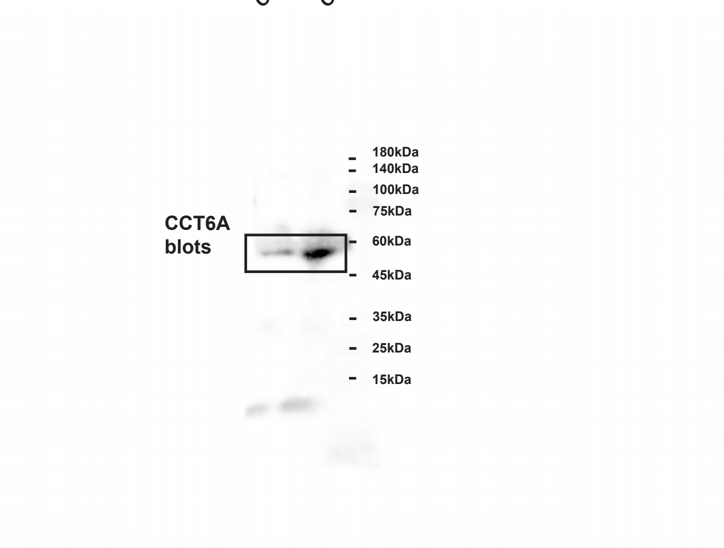


AsPC-1

OE-NC OE-CCT6A

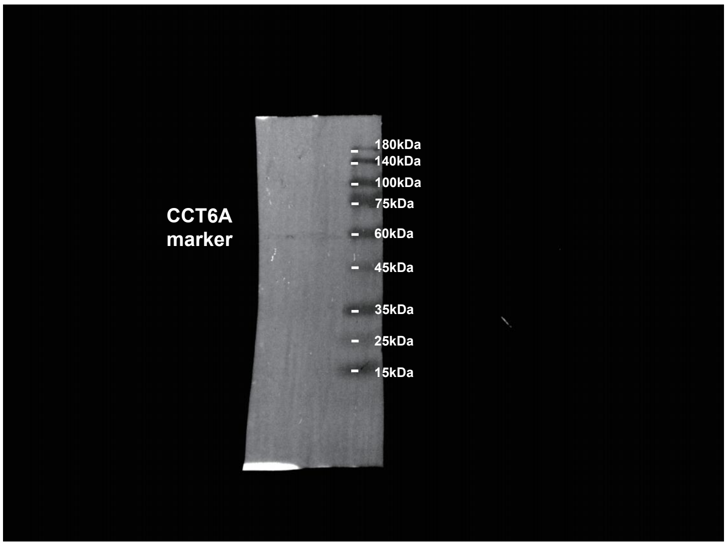


AsPC-1

**Repeat#3**

OE-NC OE-CCT6A

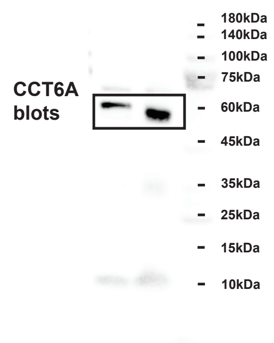


AsPC-1

OE-NC OE-CCT6A

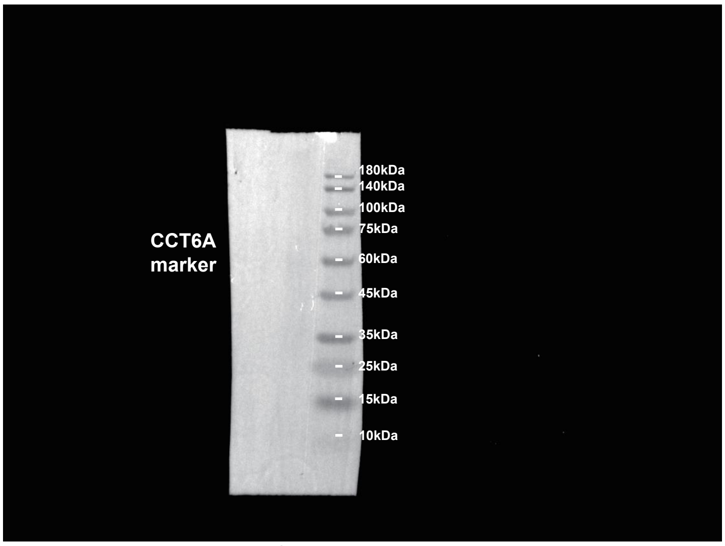


AsPC-1

Repeat#1

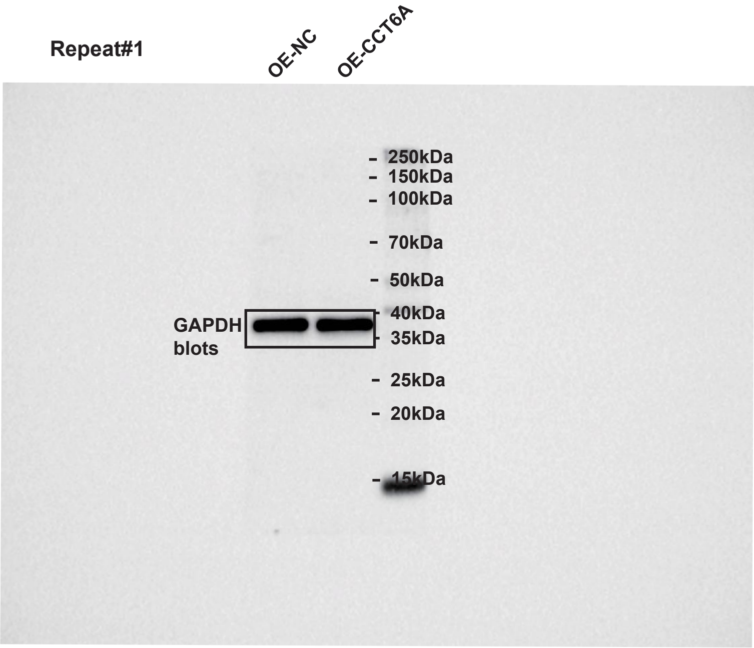


AsPC-1

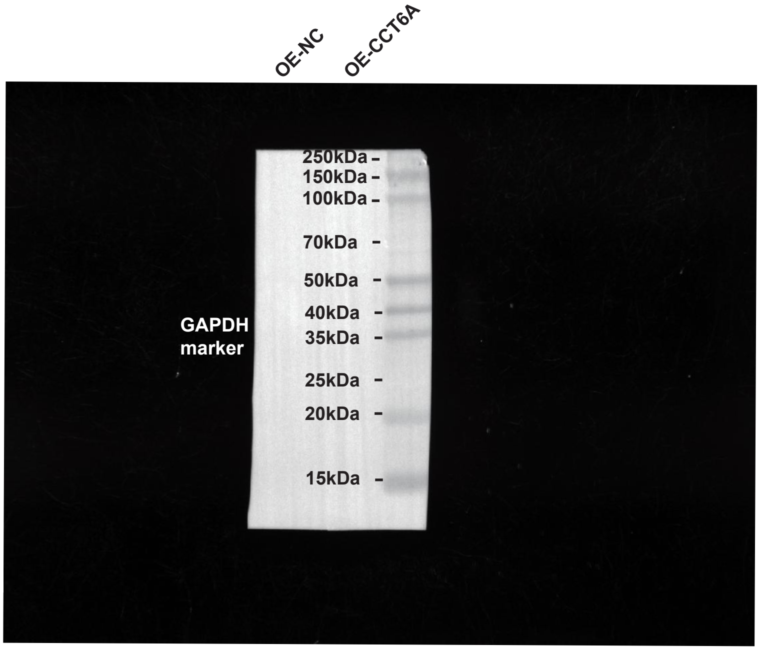


AsPC-1

Repeat#2

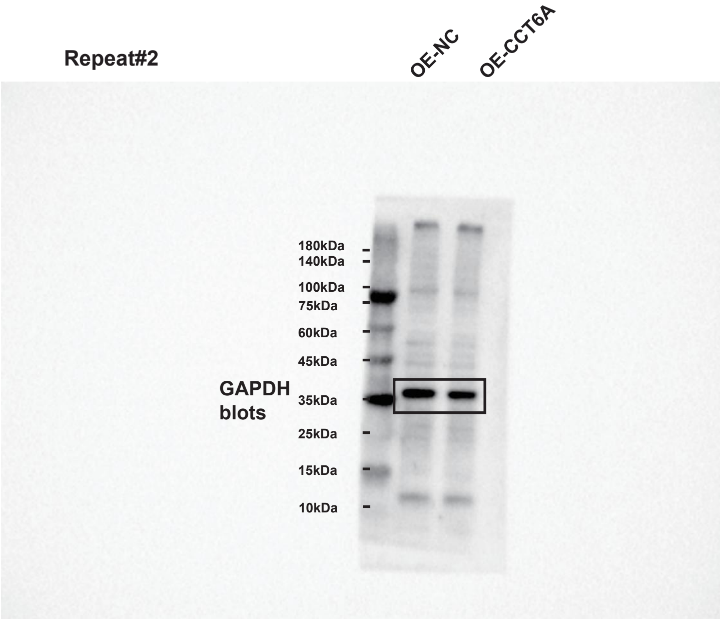


AsPC-1

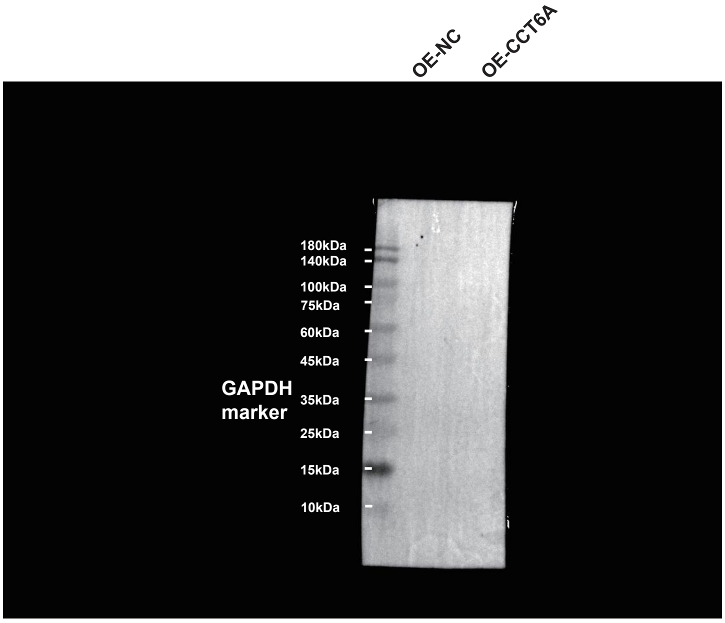


AsPC-1

Repeat#3

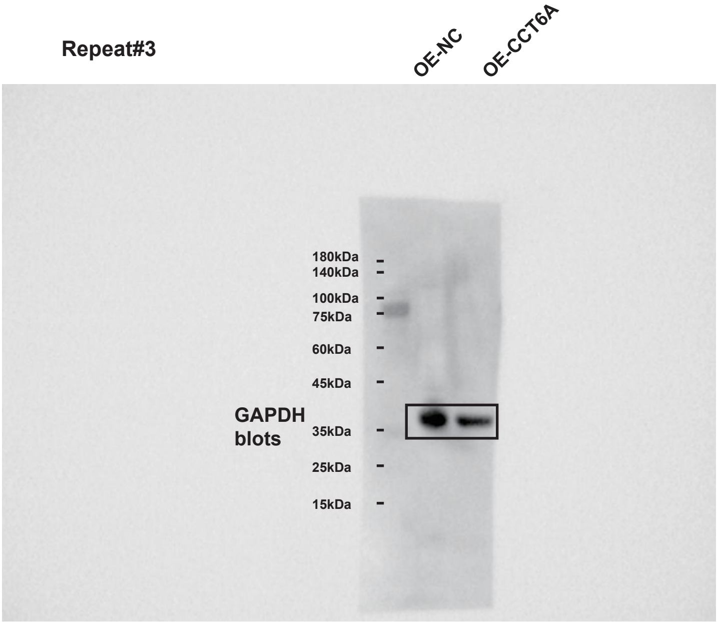


AsPC-1

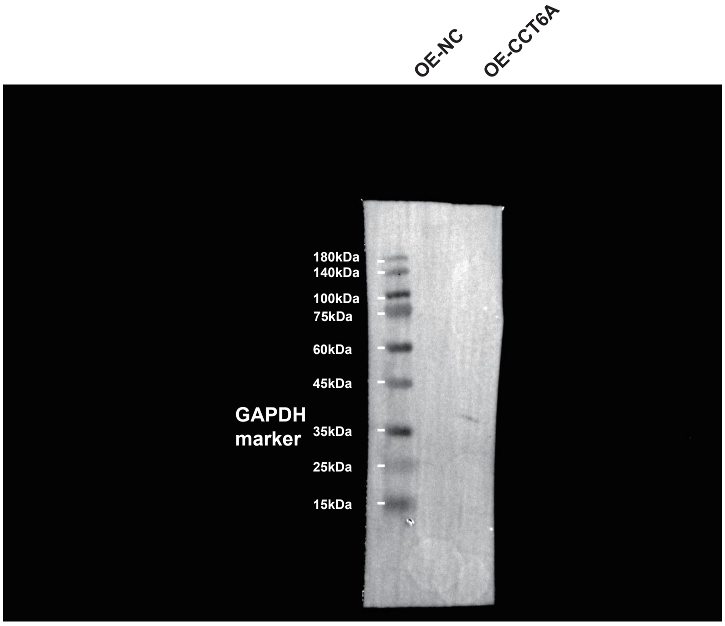


AsPC-1

**Repeat#1**

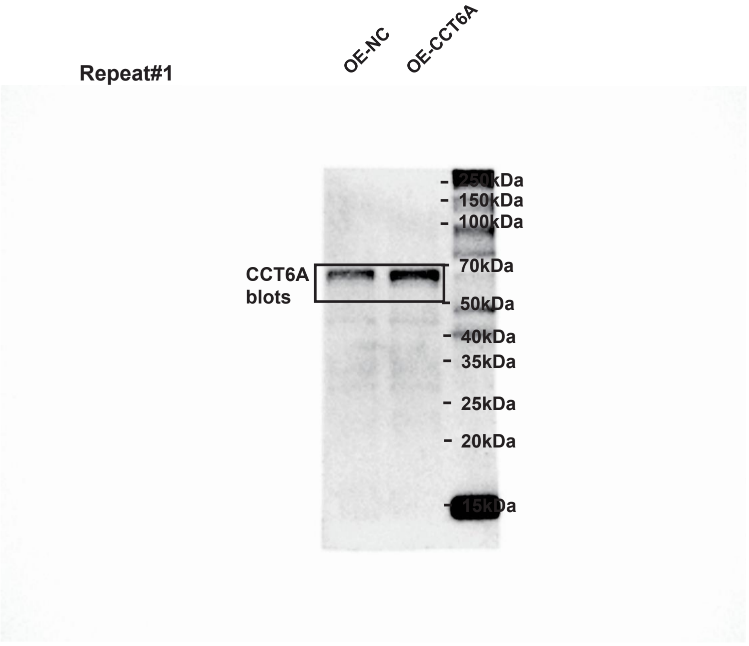


BxPC-3

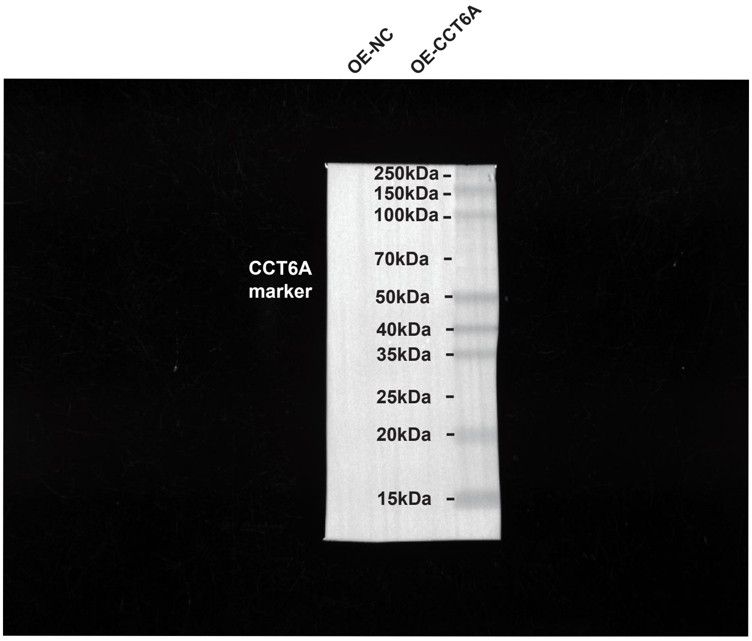


BxPC-3

**Repeat#2**

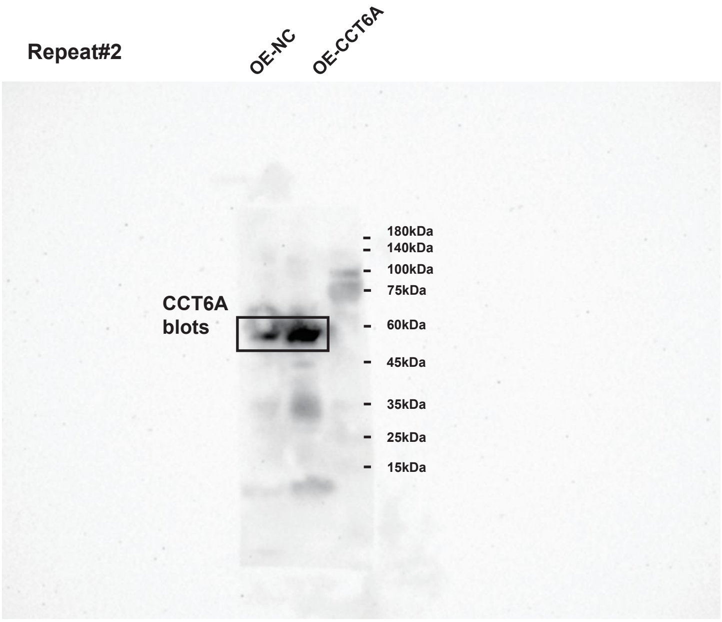


BxPC-3

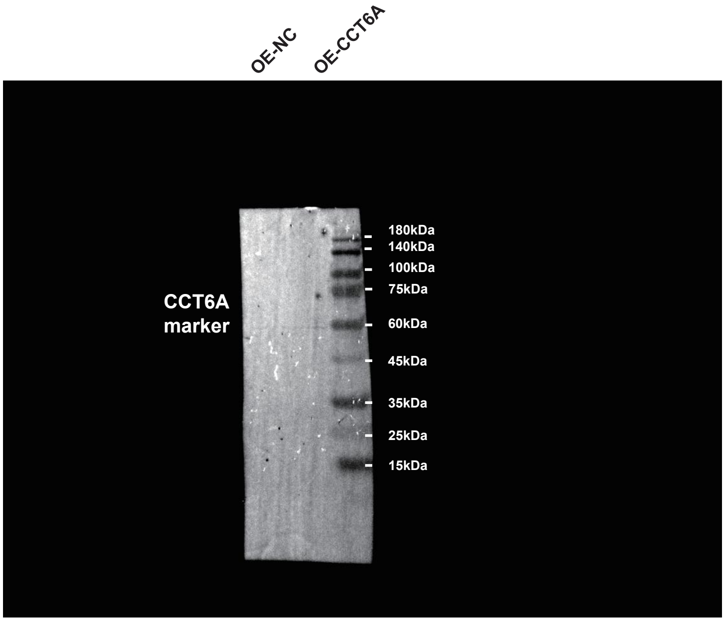


BxPC-3

**Repeat#3**

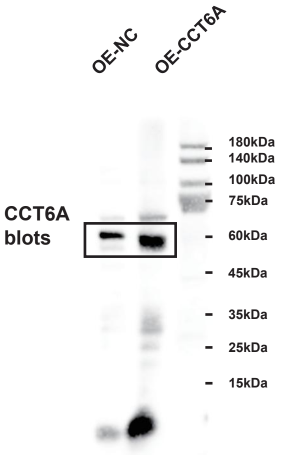


BxPC-3

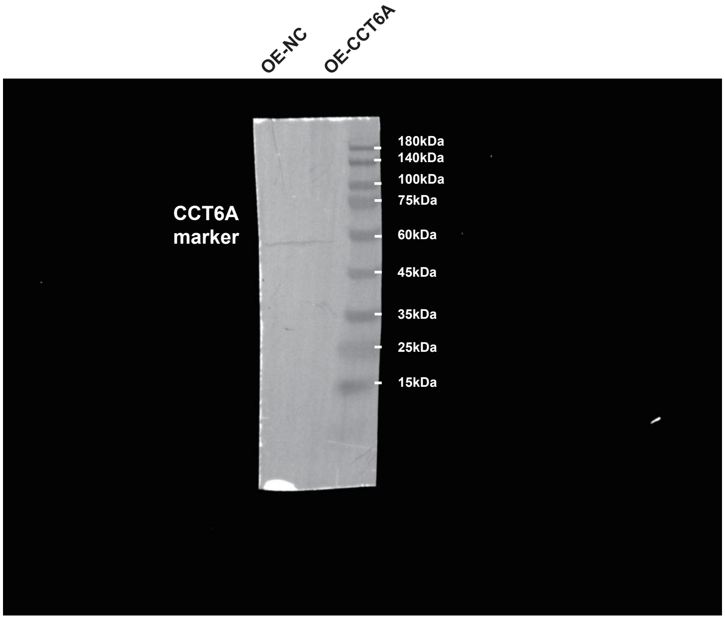


BxPC-3

Repeat#1

OE-NC OE-CCT6A

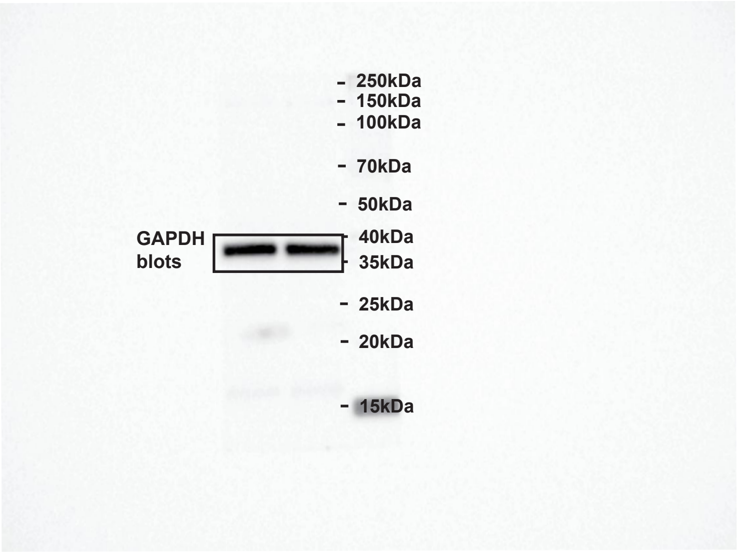


BxPC-3

OE-NC OE-CCT6A

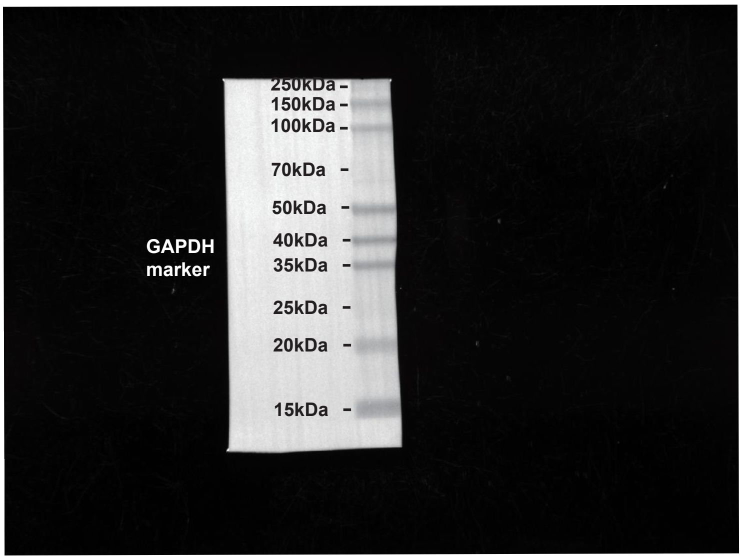


BxPC-3

Repeat#2

OE-NC OE-CCT6A

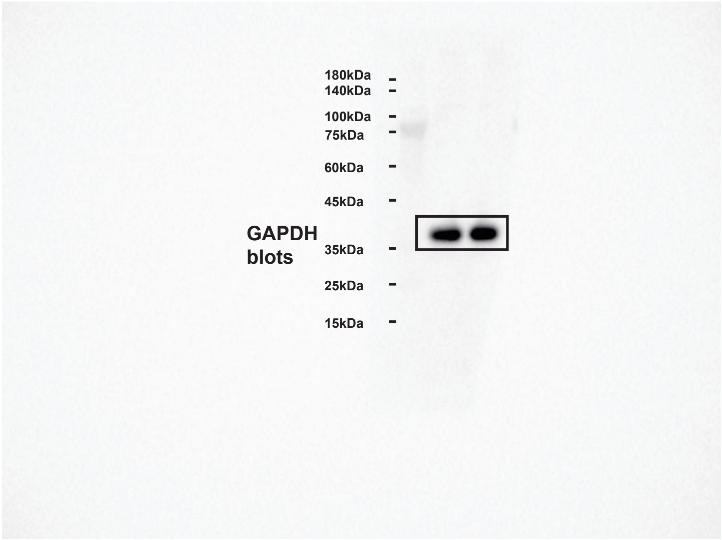


BxPC-3

OE-NC OE-CCT6A

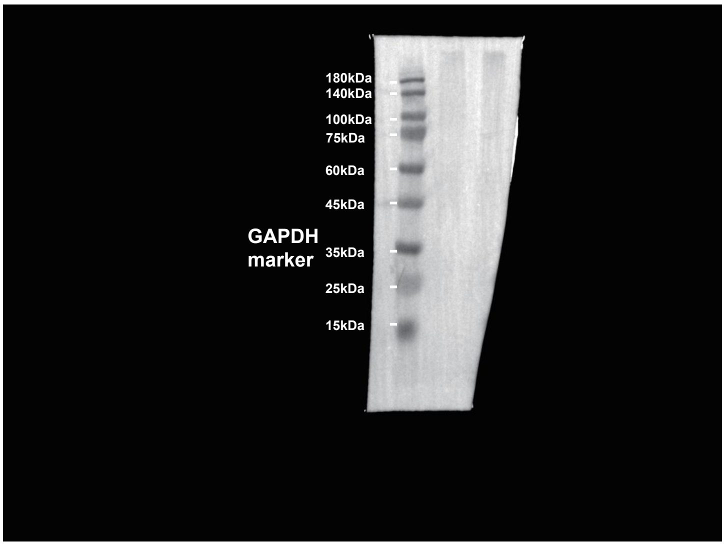


BxPC-3

Repeat#3

OE-NC OE-CCT6A

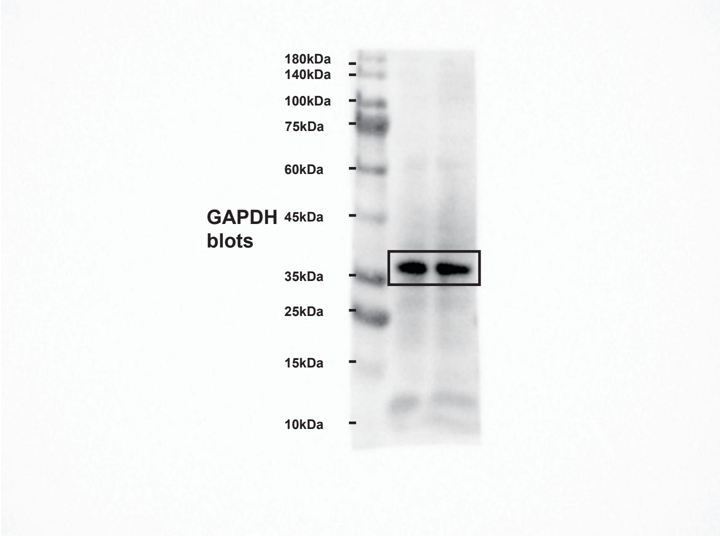


BxPC-3

OE-NC OE-CCT6A

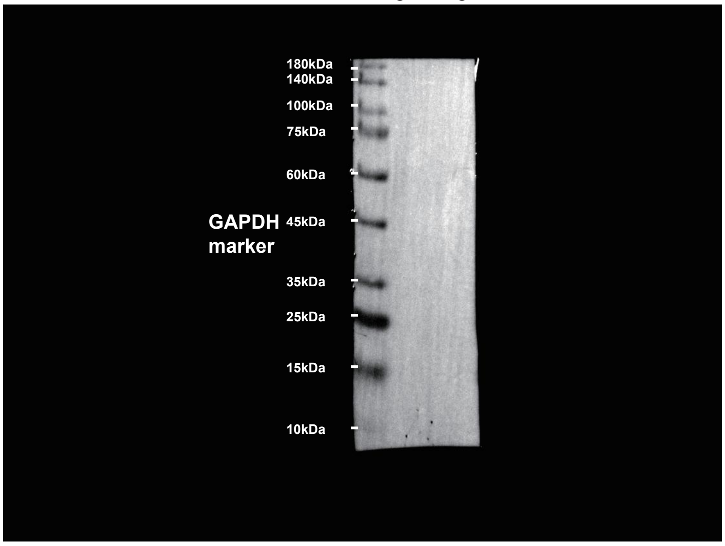


BxPC-3

Fig. S10A

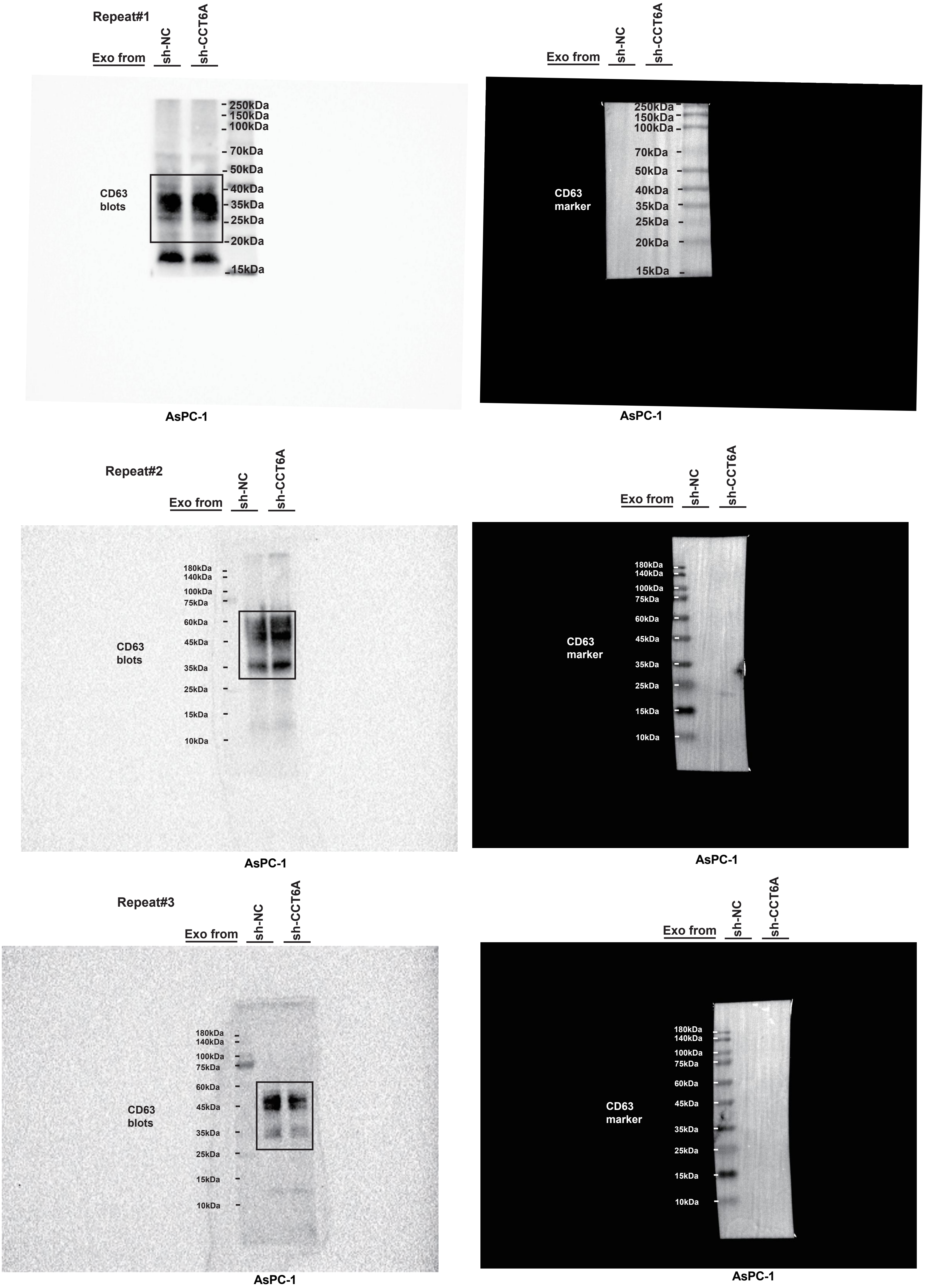

Repeat#1
Exo from
sh-NC
sh-CCT6A
CD63 blots
250kDa
150kDa
100kDa
70kDa
50kDa
40kDa
35kDa
25kDa
20kDa
15kDa
AsPC-1
CD63 marker
AsPC-1
Repeat#2
CD63 blots
180kDa
140kDa
100kDa
75kDa
60kDa
45kDa
35kDa
25kDa
15kDa
10kDa
AsPC-1
CD63 marker
AsPC-1
Repeat#3
CD63 blots
AsPC-1
CD63 marker
AsPC-1

Repeat#1

Exo from sh-NC sh-CCT6A

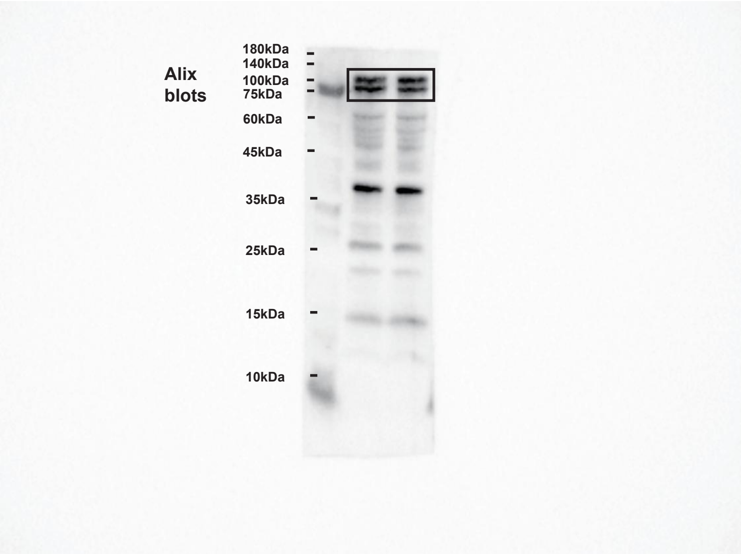


AsPC-1

Exo from sh-NC sh-CCT6A

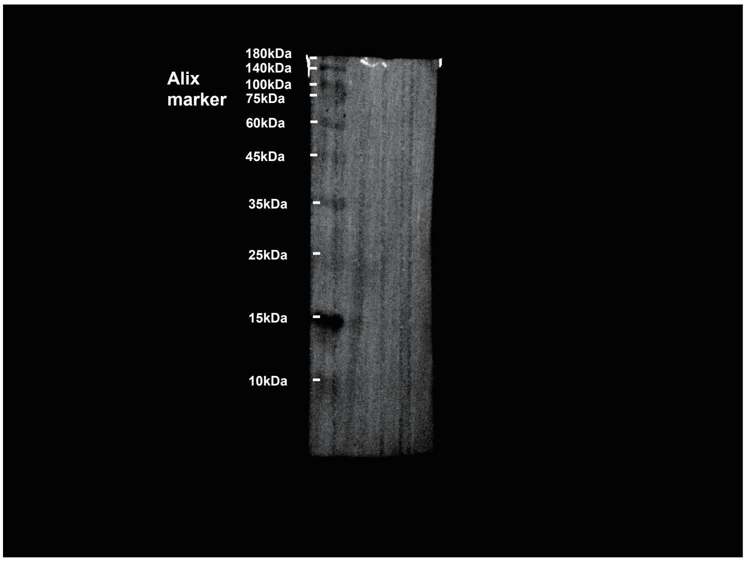


AsPC-1

Repeat#2

Exo from sh-NC sh-CCT6A

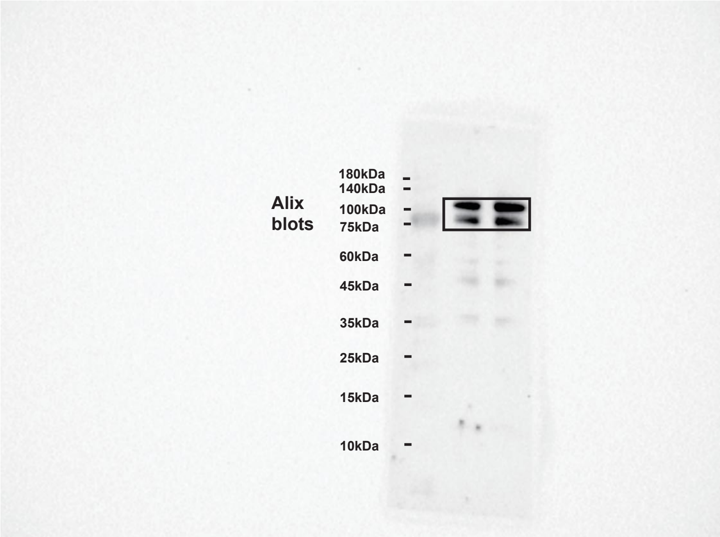


AsPC-1

Exo from sh-NC sh-CCT6A

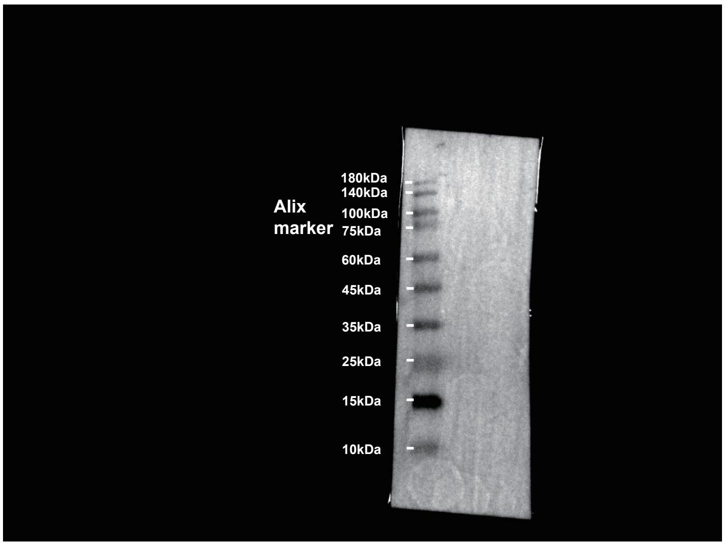


AsPC-1

Repeat#3

Exo from sh-NC sh-CCT6A

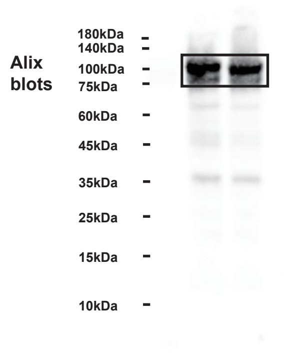


AsPC-1

Exo from sh-NC sh-CCT6A

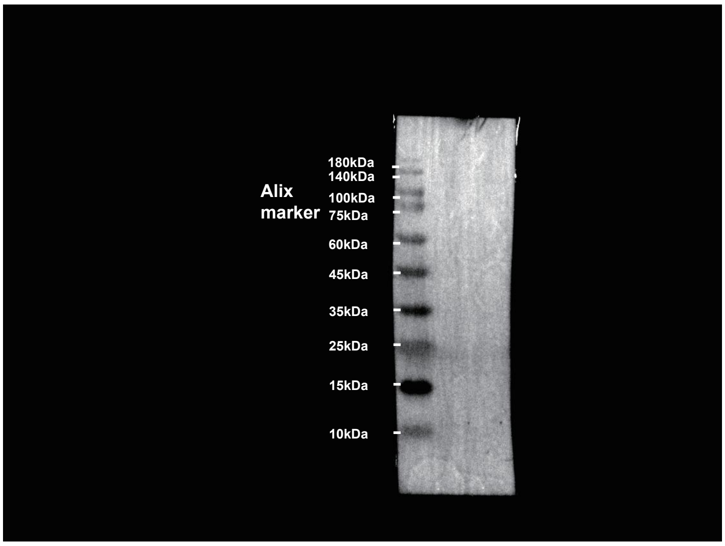


AsPC-1

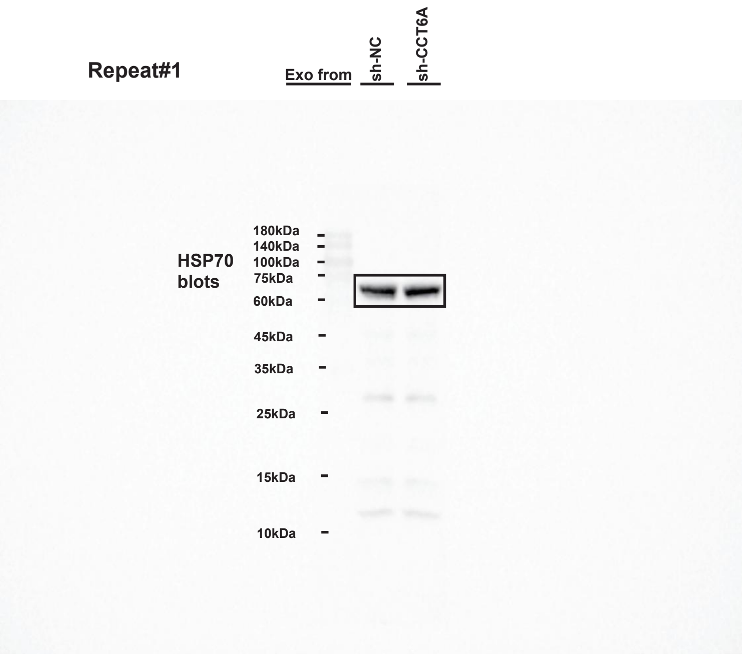


AsPC-1

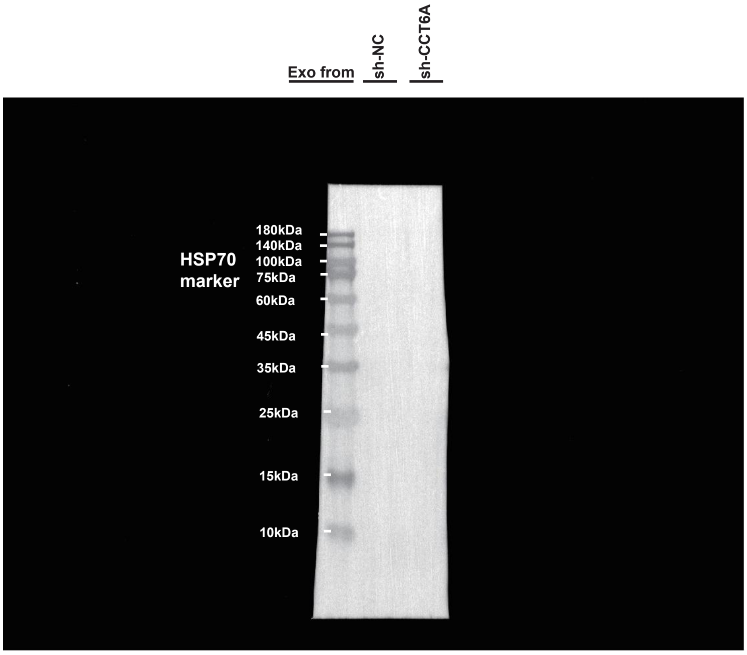


AsPC-1

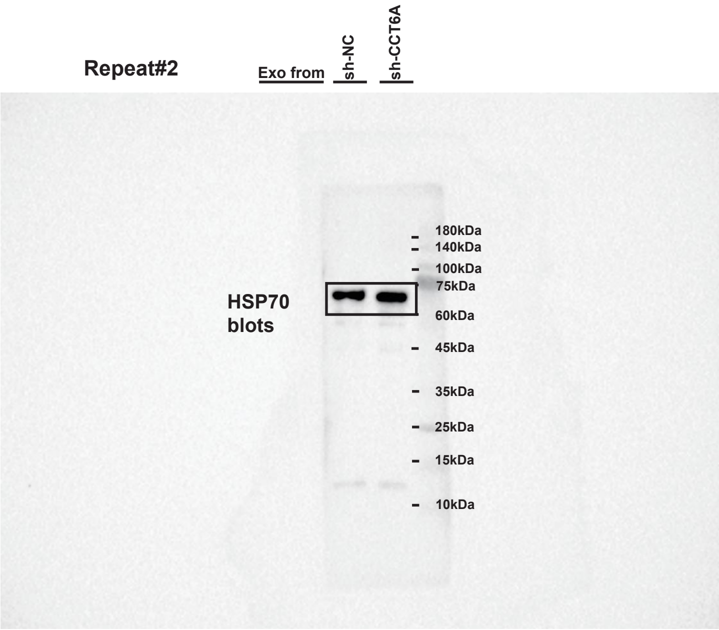


AsPC-1

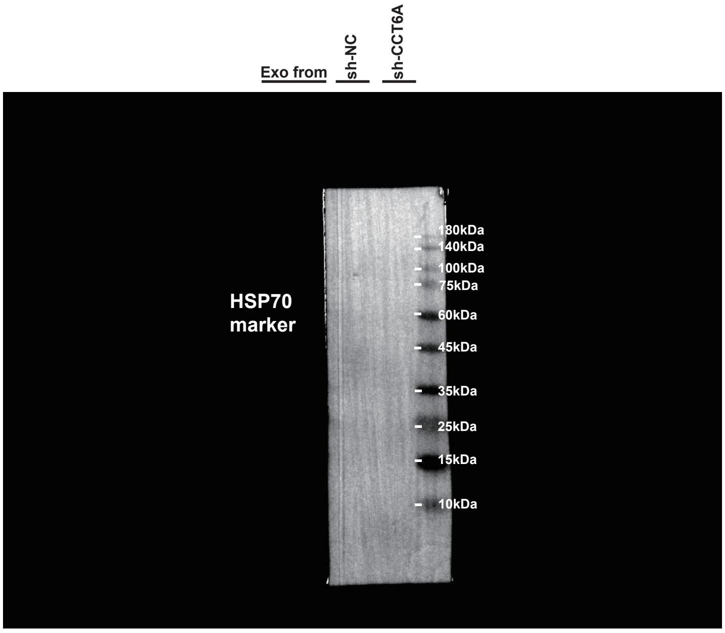


AsPC-1

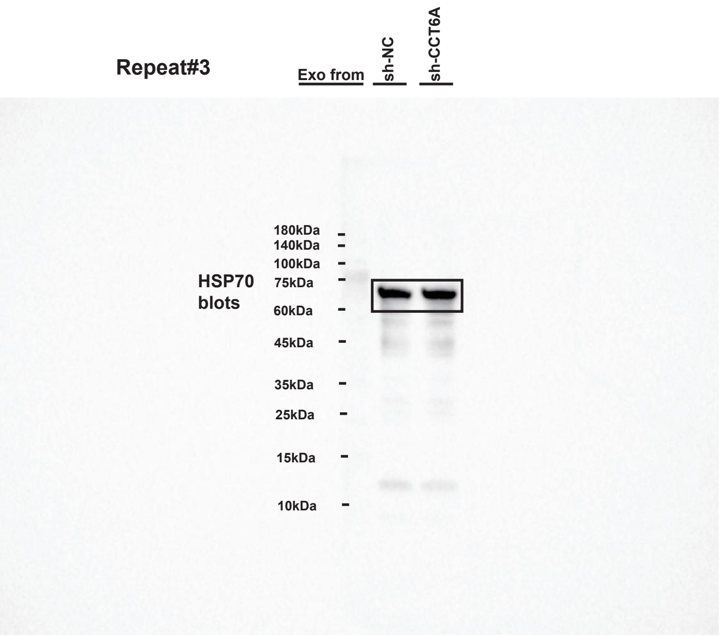


AsPC-1

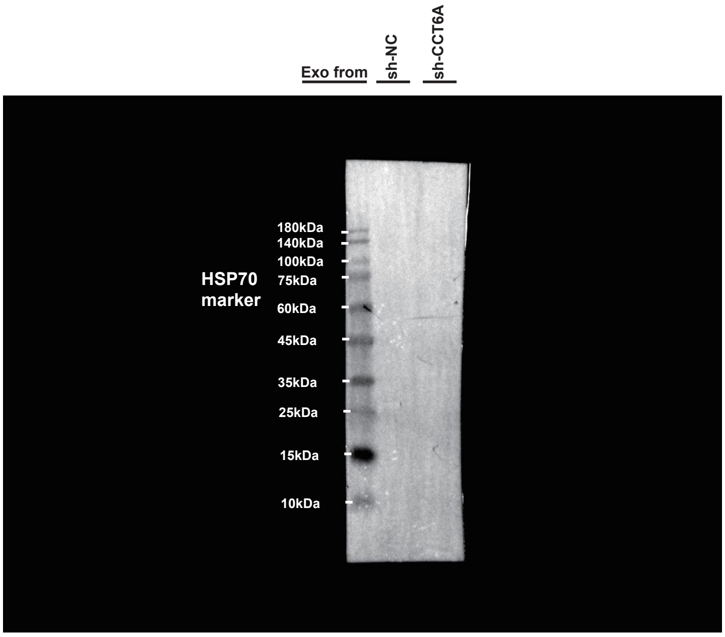


AsPC-1

Repeat#1

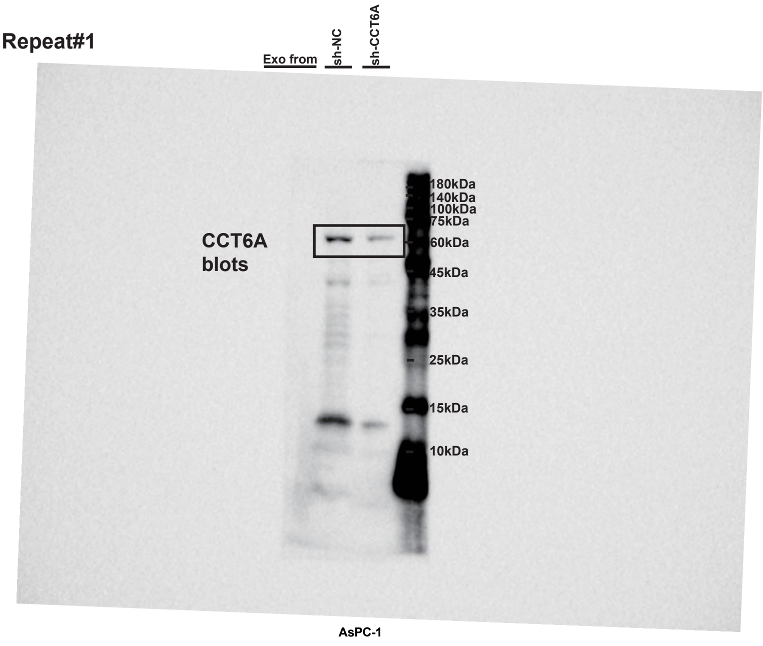


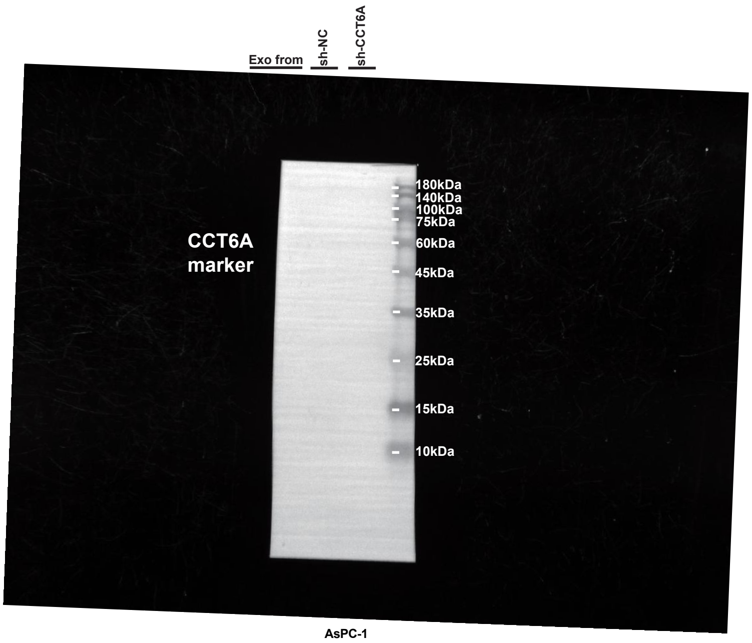


Repeat#2

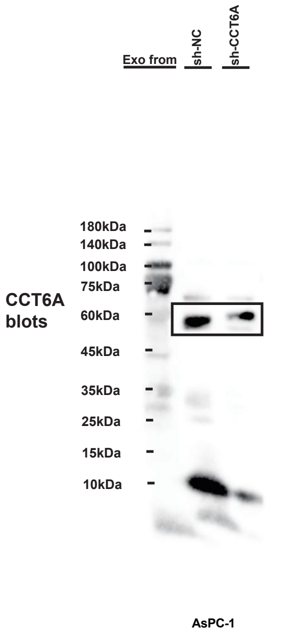


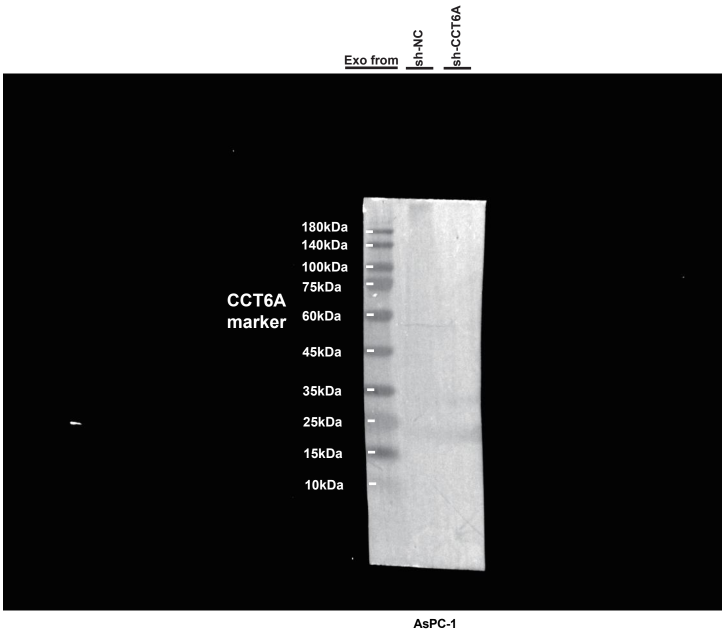


Repeat#3

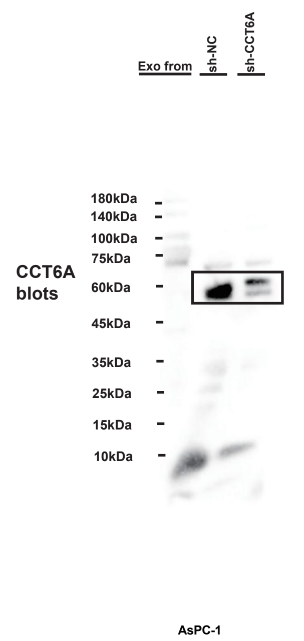


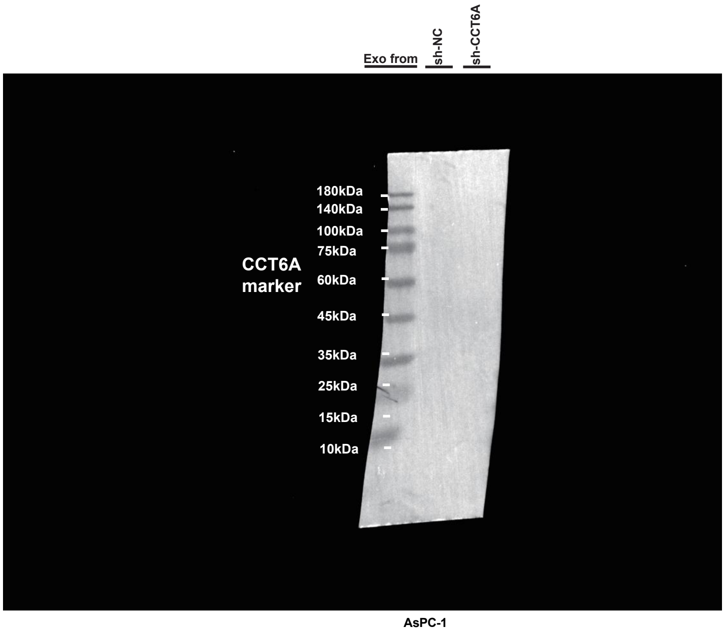

**Repeat#1**

Exo from sh-NC sh-CCT6A

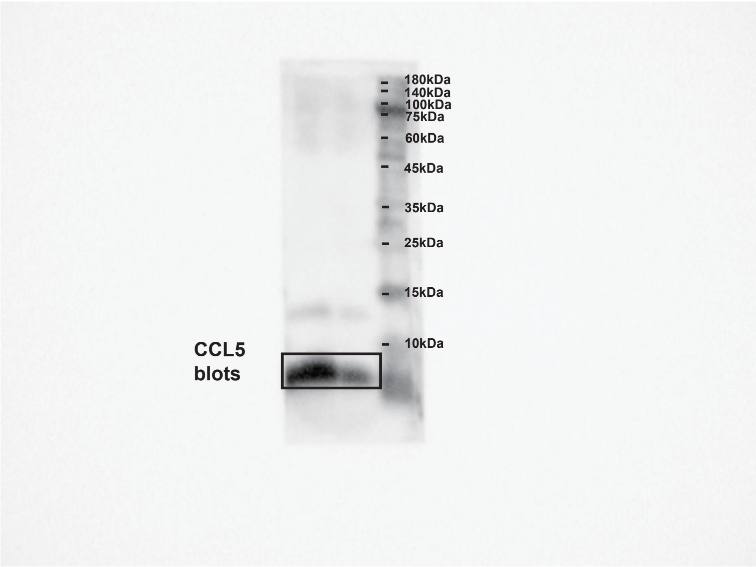


AsPC-1

Exo from sh-NC sh-CCT6A

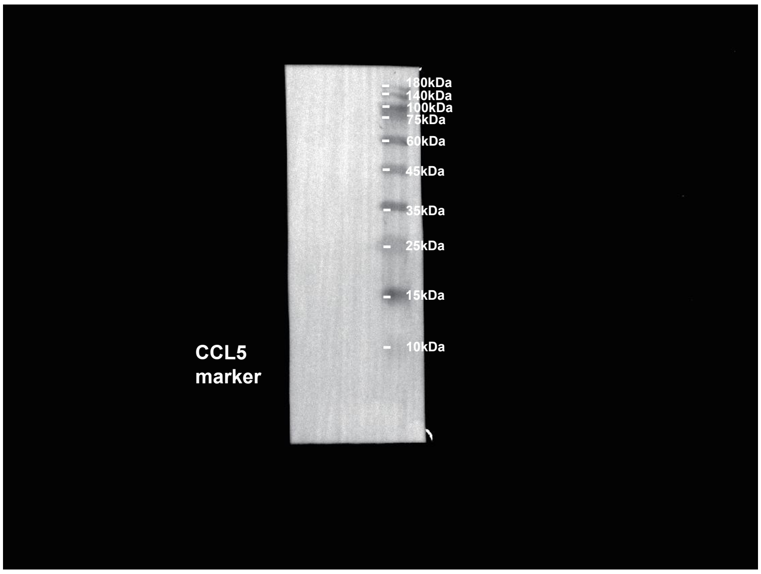


AsPC-1

**Repeat#2**

Exo from sh-NC sh-CCT6A

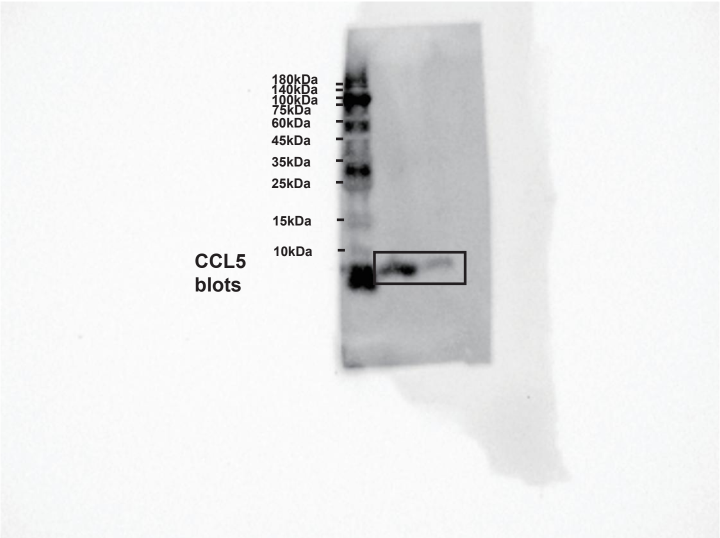


AsPC-1

Exo from sh-NC sh-CCT6A

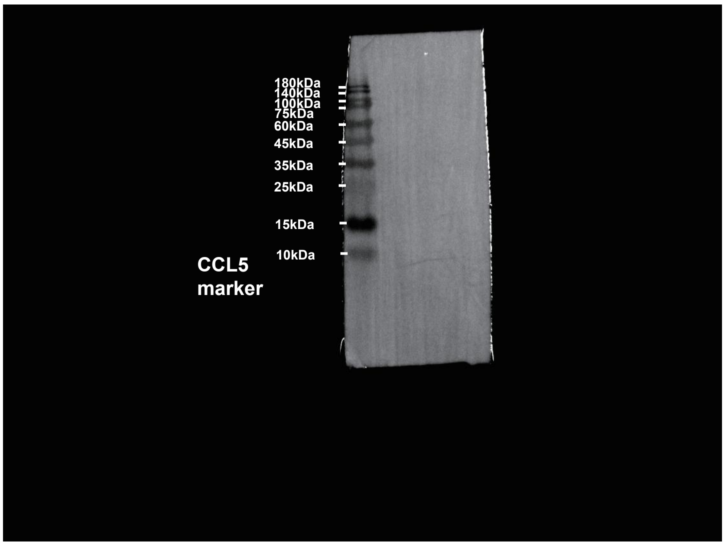


AsPC-1

**Repeat#3**

Exo from sh-NC sh-CCT6A

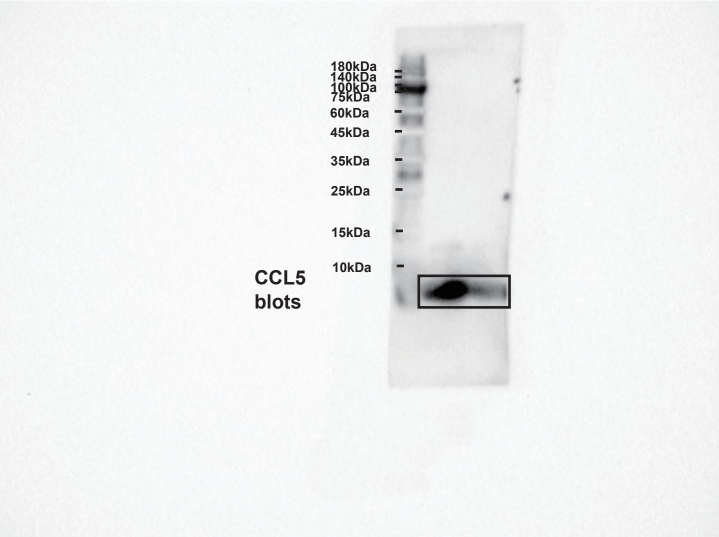


AsPC-1

Exo from sh-NC sh-CCT6A

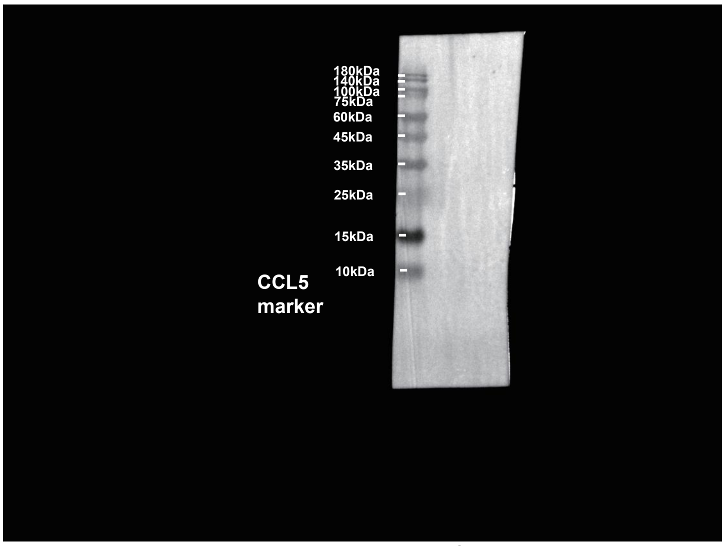


AsPC-1

Repeat#1

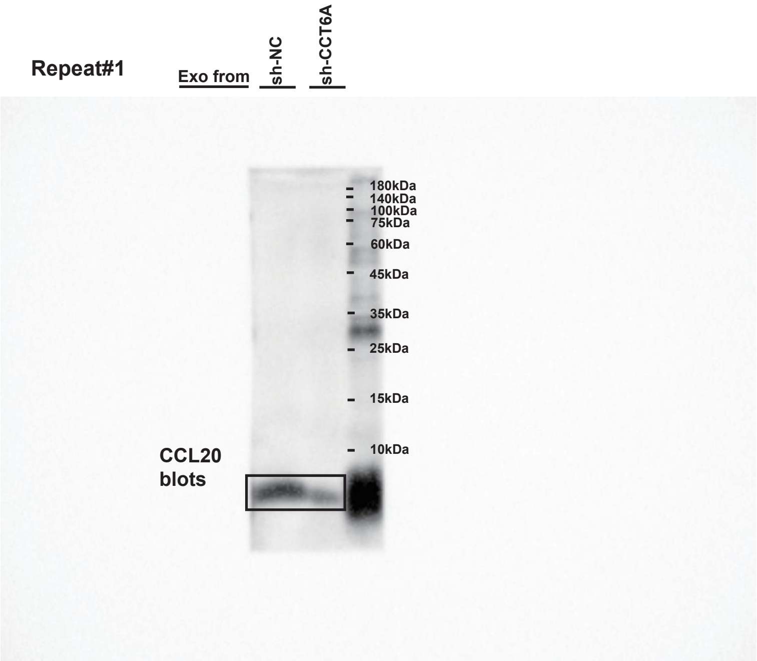


AsPC-1

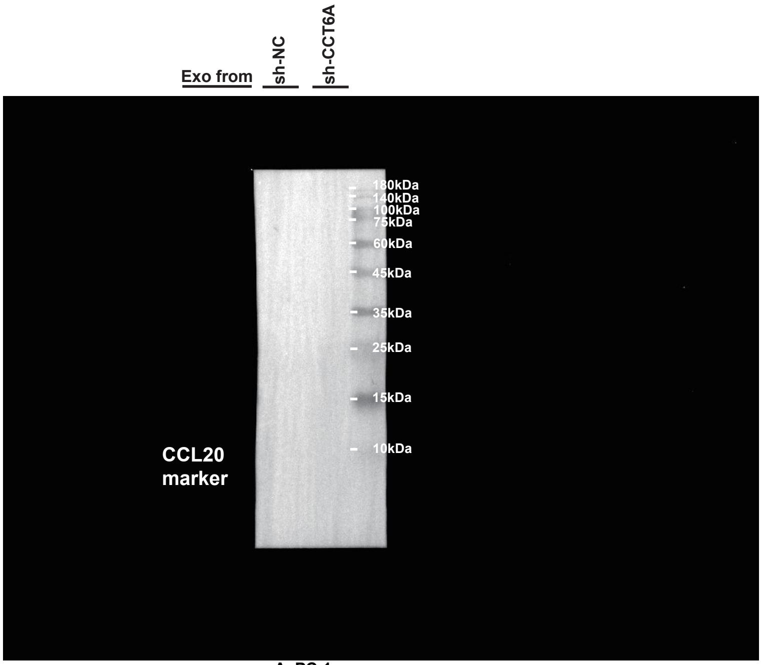


AsPC-1

Repeat#2

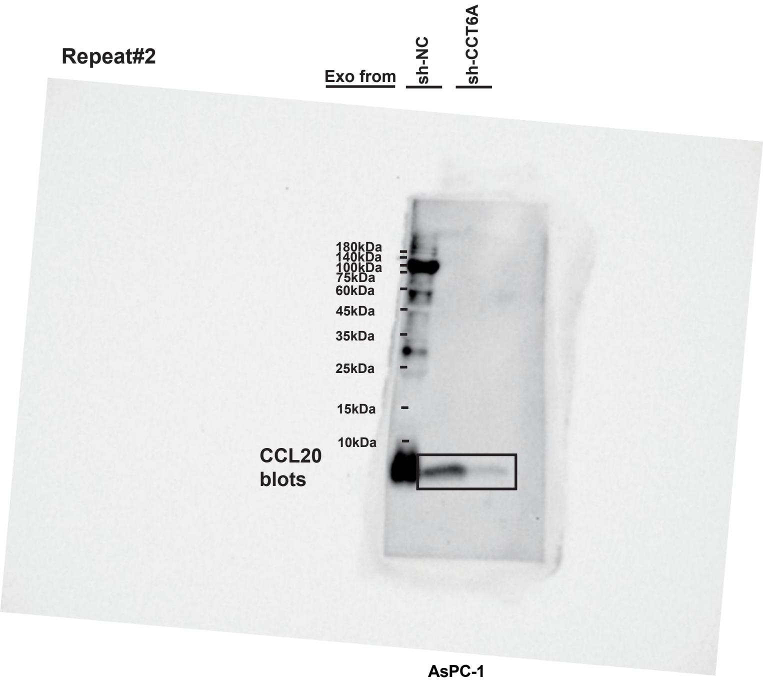


AsPC-1

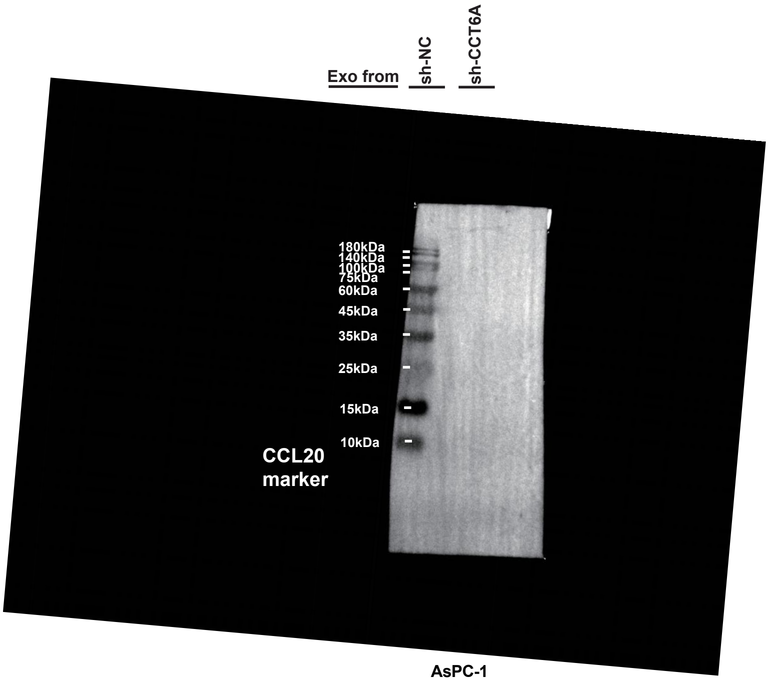


AsPC-1

Repeat#3

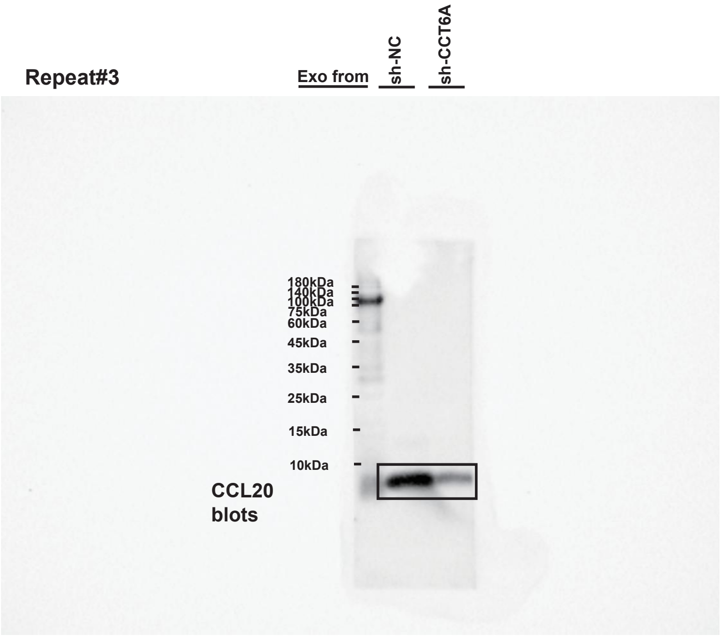


AsPC-1

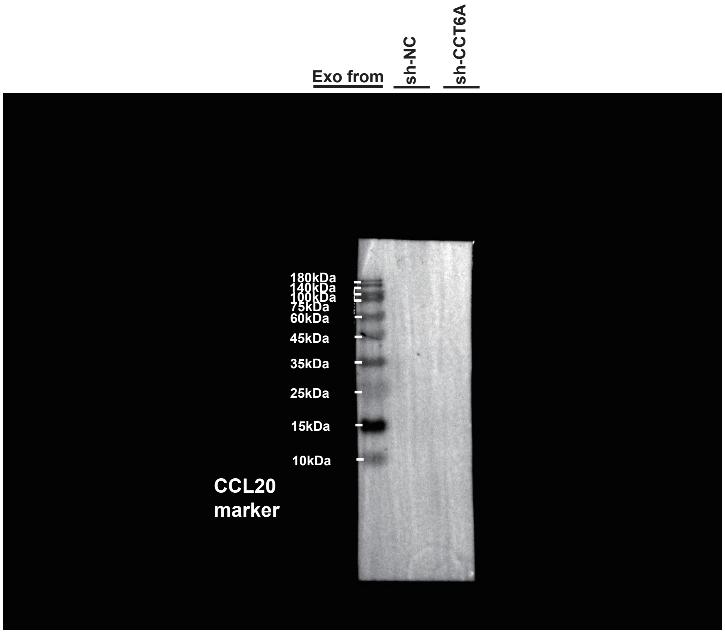


AsPC-1

Repeat#1

Exo from sh-NC sh-CCT6A

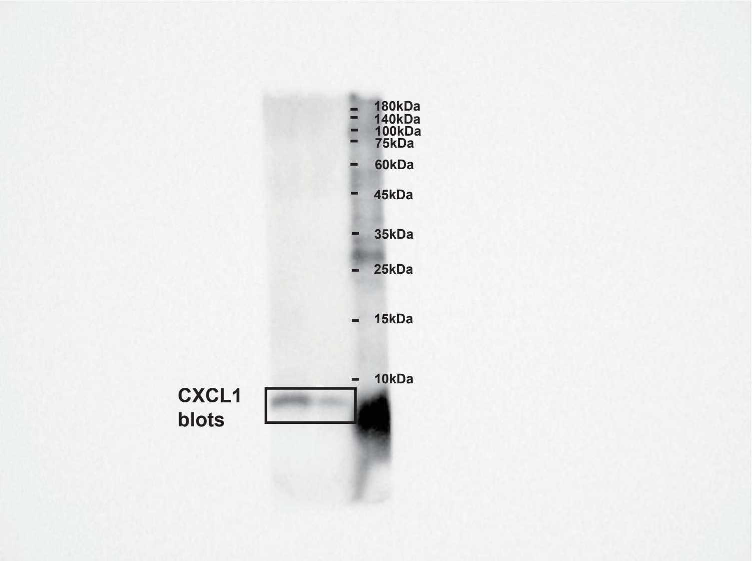


AsPC-1

Exo from sh-NC sh-CCT6A

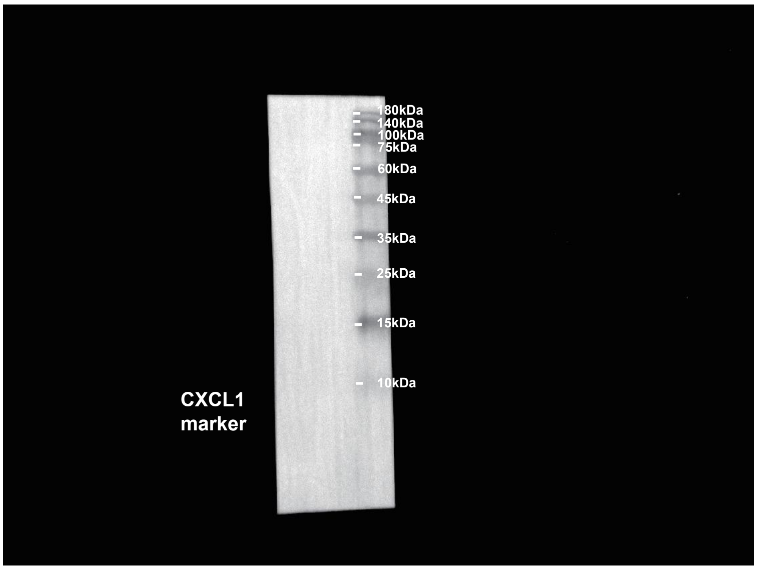


AsPC-1

Repeat#2

Exo from sh-NC sh-CCT6A

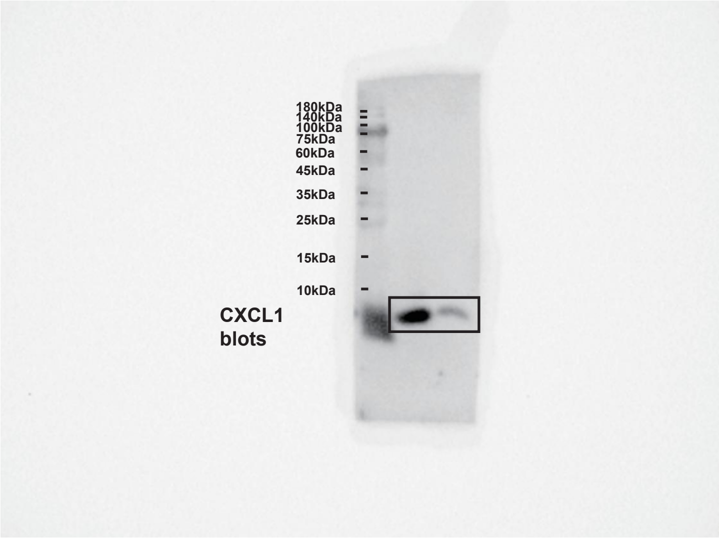


AsPC-1

Exo from sh-NC sh-CCT6A

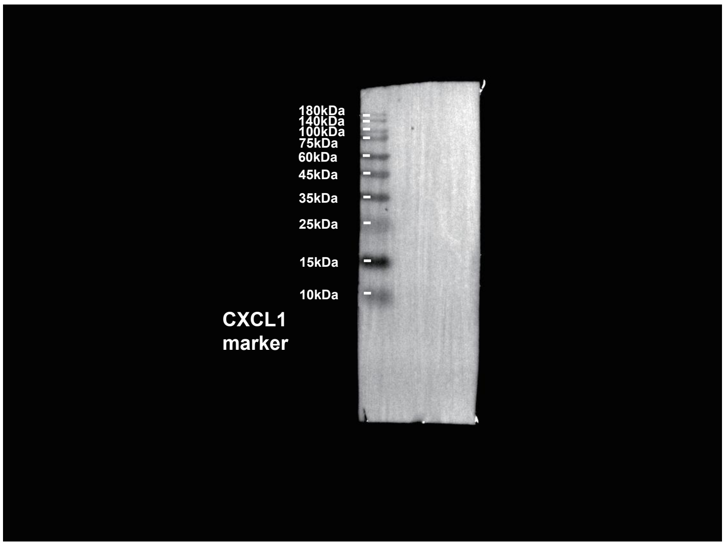


AsPC-1

Repeat#3

Exo from sh-NC sh-CCT6A

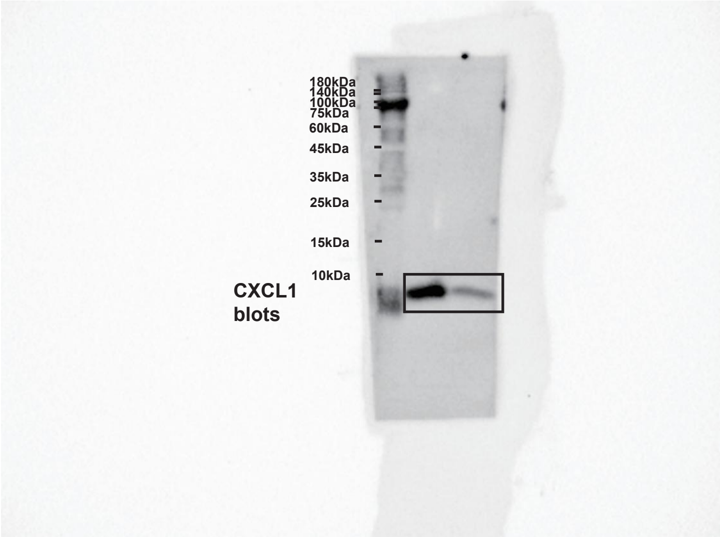


AsPC-1

Exo from sh-NC sh-CCT6A

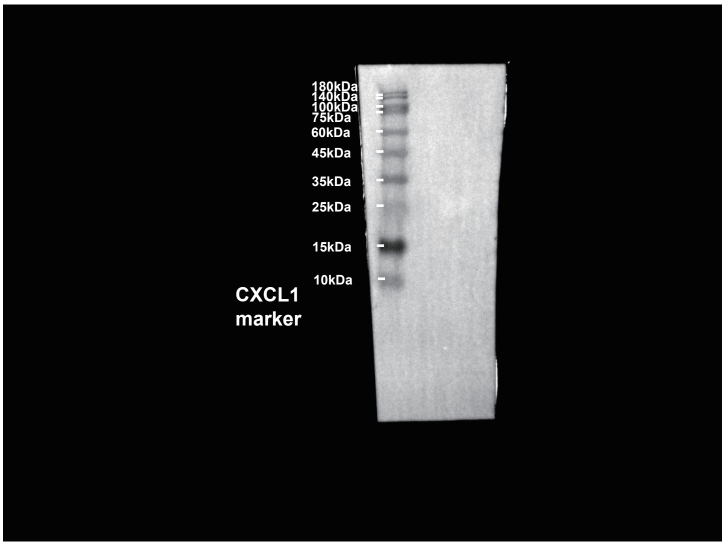


AsPC-1

Repeat#1

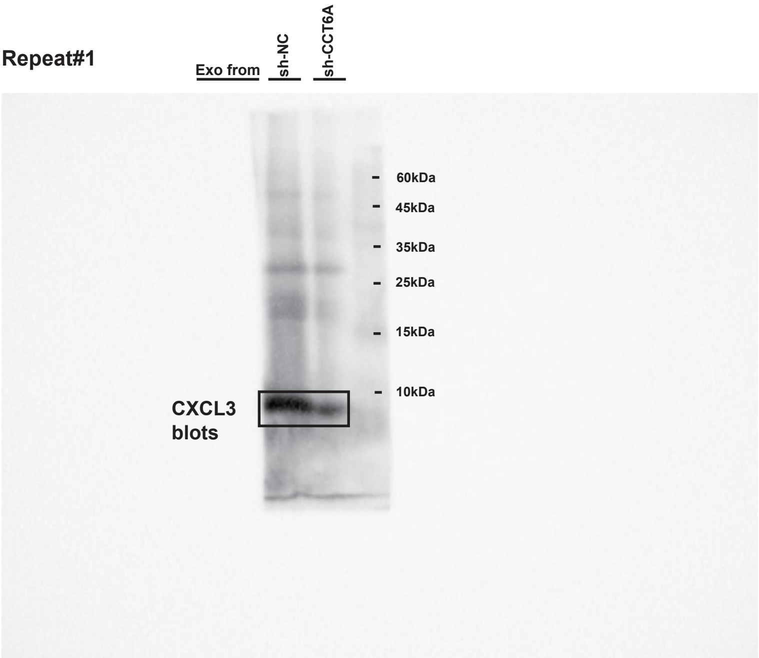


AsPC-1

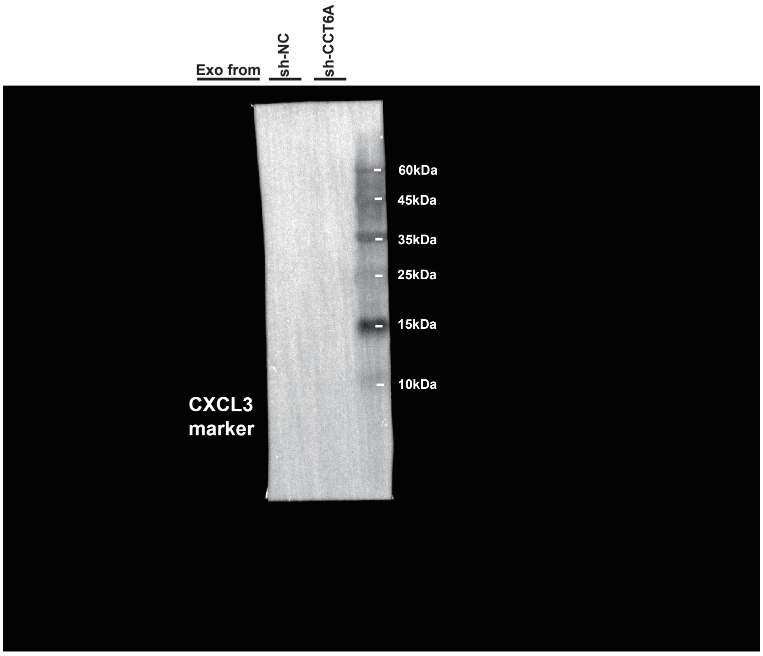


AsPC-1

Repeat#2

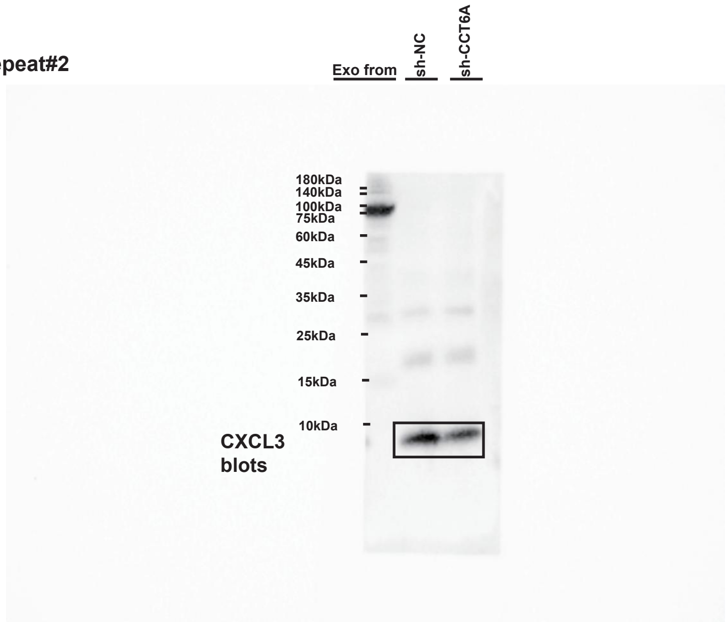


AsPC-1

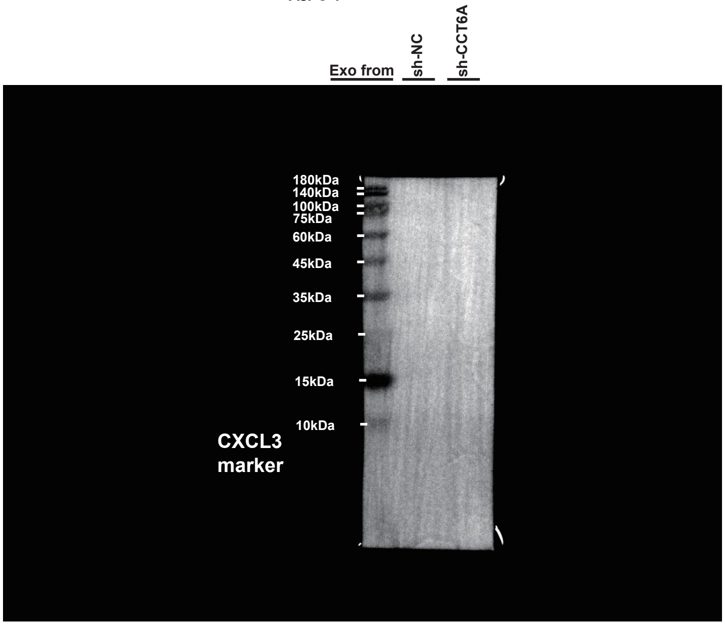


AsPC-1

Repeat#3

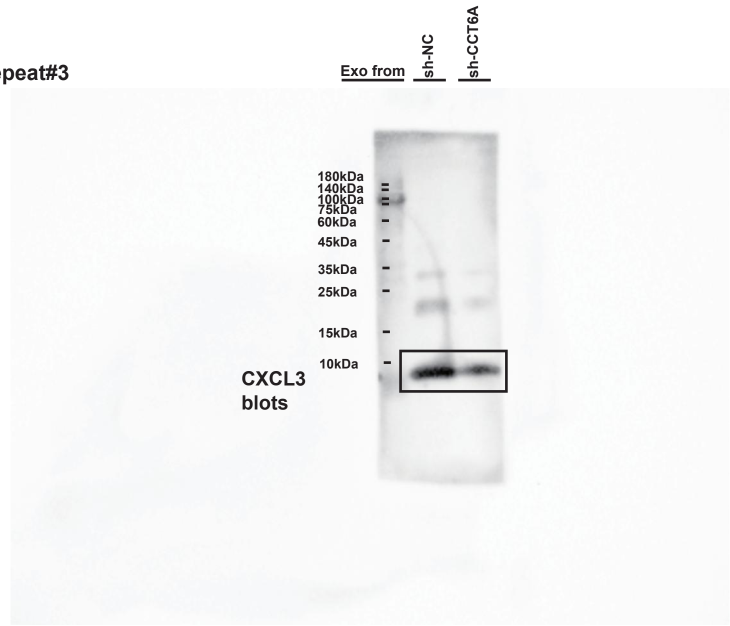


AsPC-1

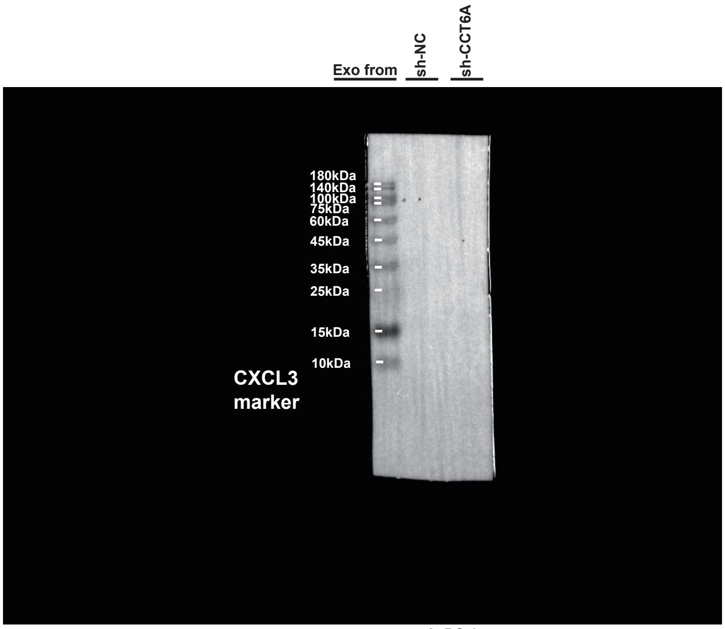


AsPC-1

**Fig. S10C**

**Repeat#1**

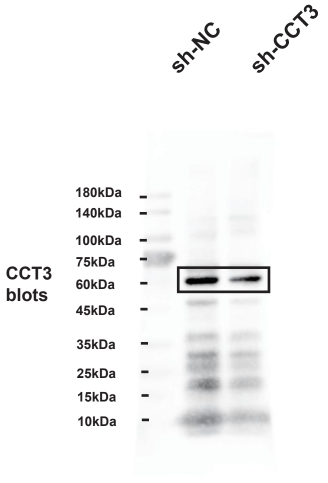


**AsPC-1**

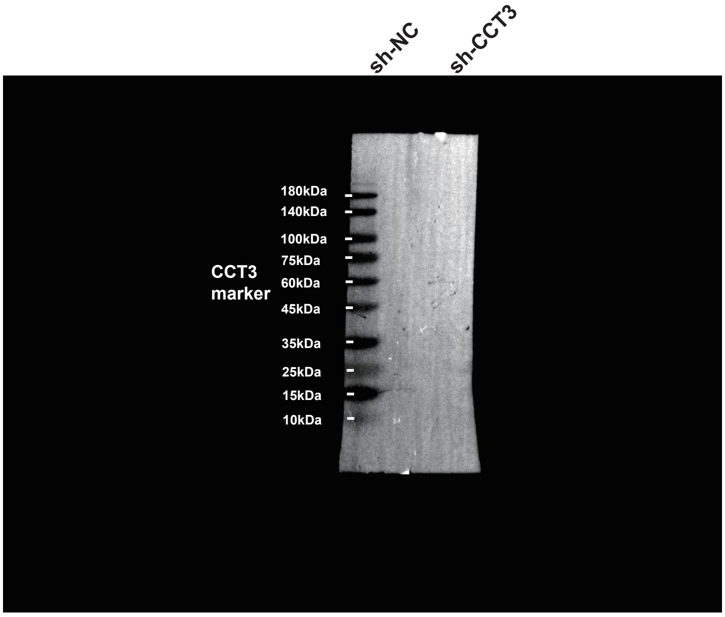


**AsPC-1**

**Repeat#2**

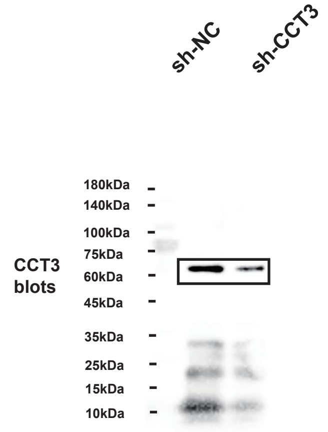


**AsPC-1**

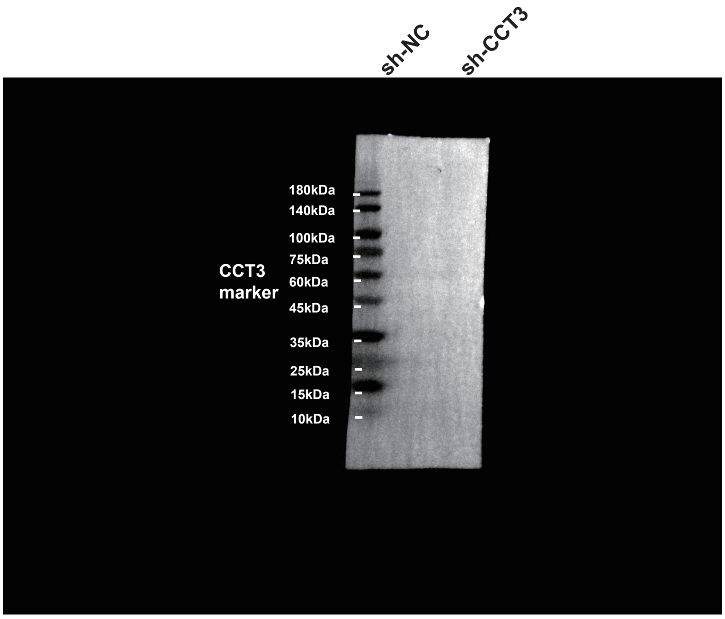


**AsPC-1**

**Repeat#3**

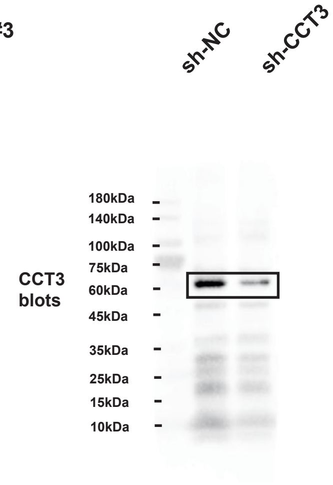


**AsPC-1**

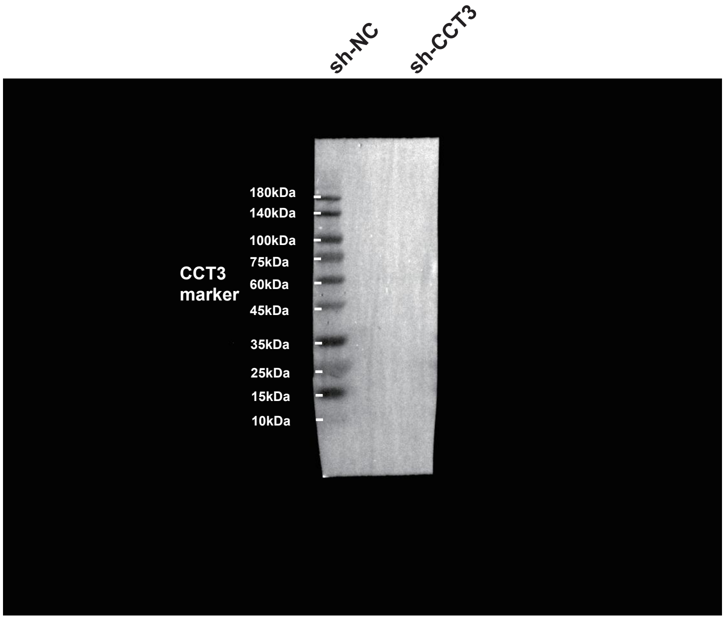


**AsPC-1**

Repeat#1

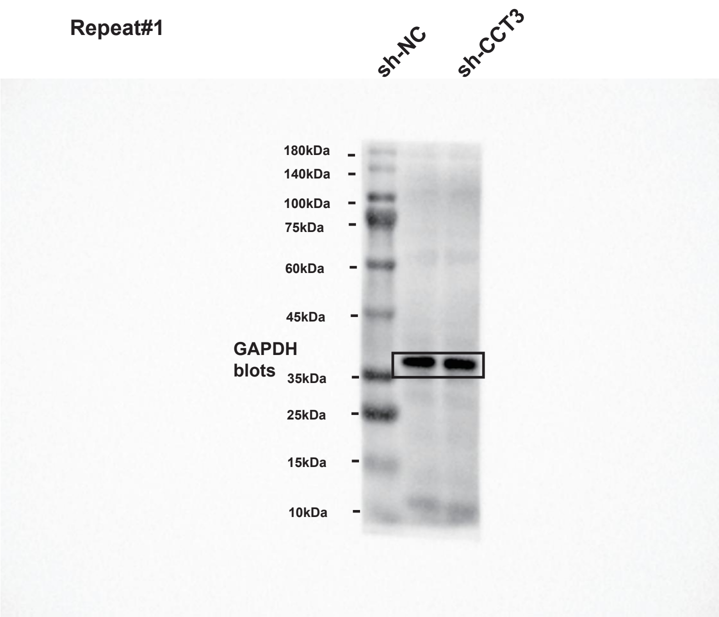


AsPC-1

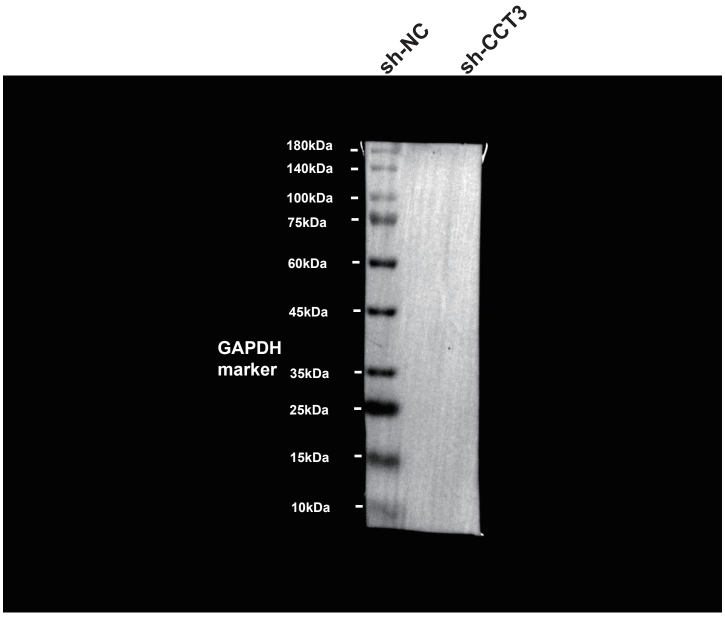


AsPC-1

Repeat#2

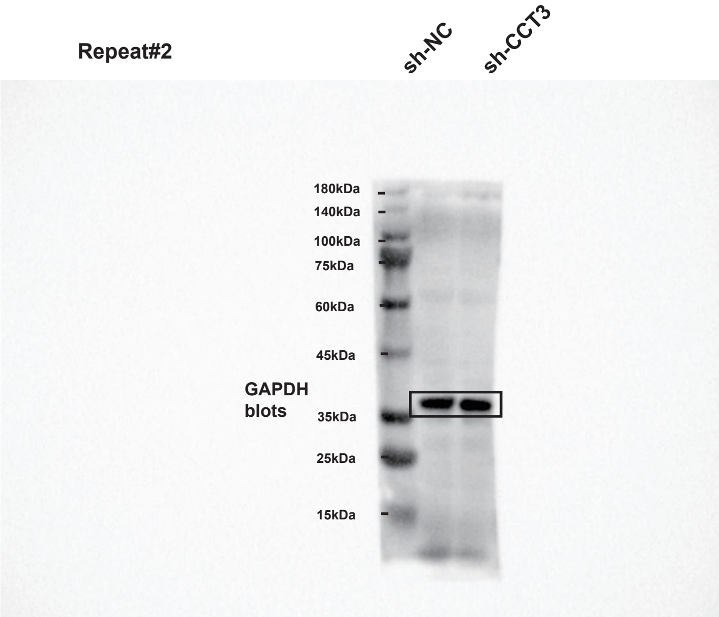


AsPC-1

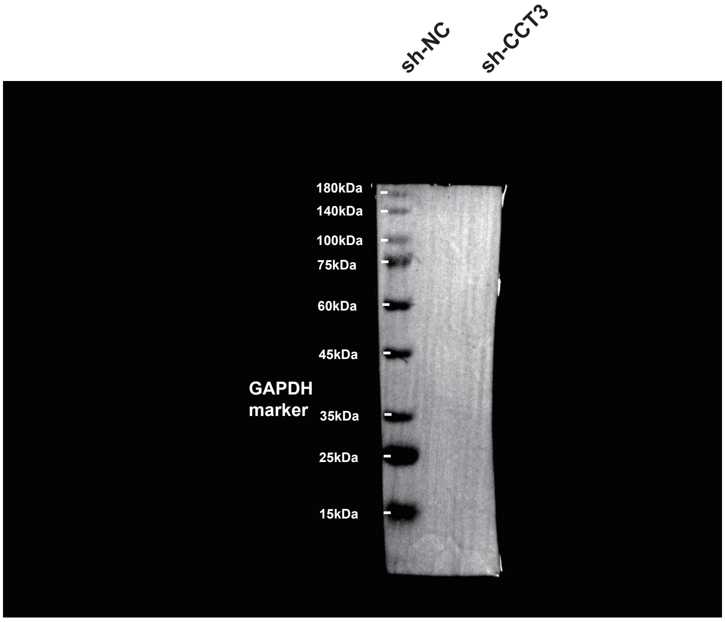


AsPC-1

Repeat#3

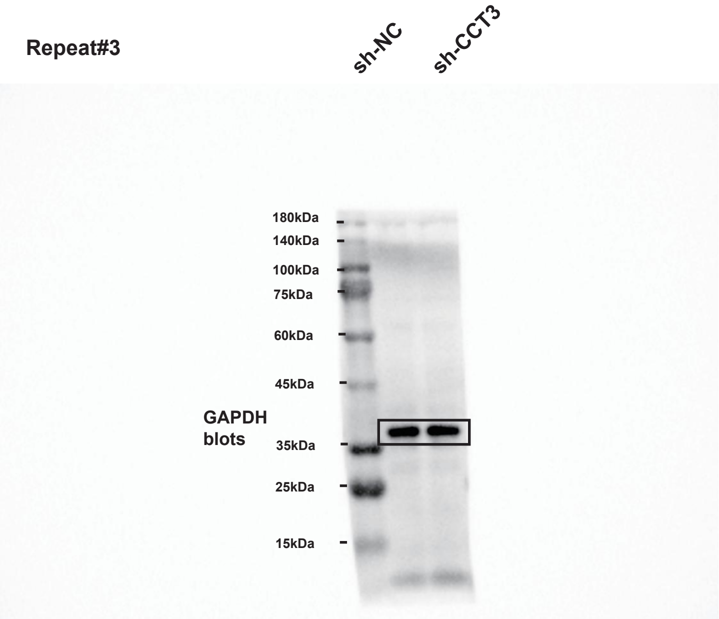


AsPC-1

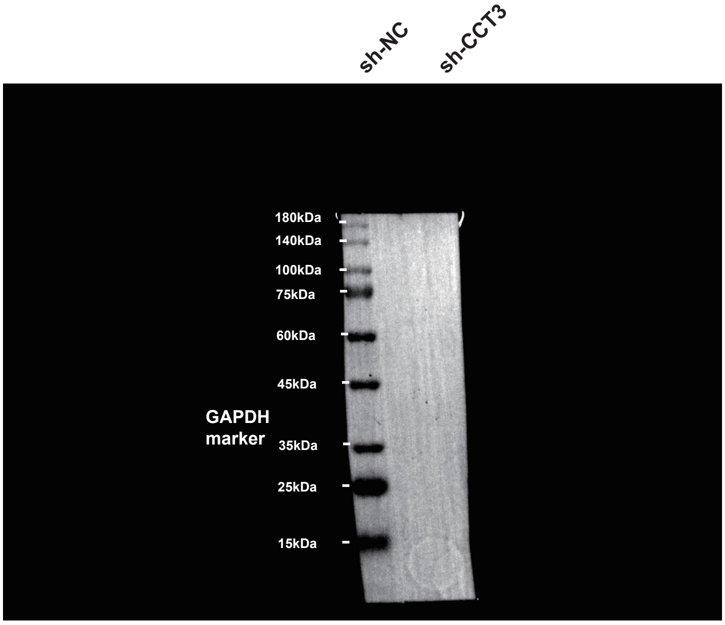


AsPC-1

Repeat#1

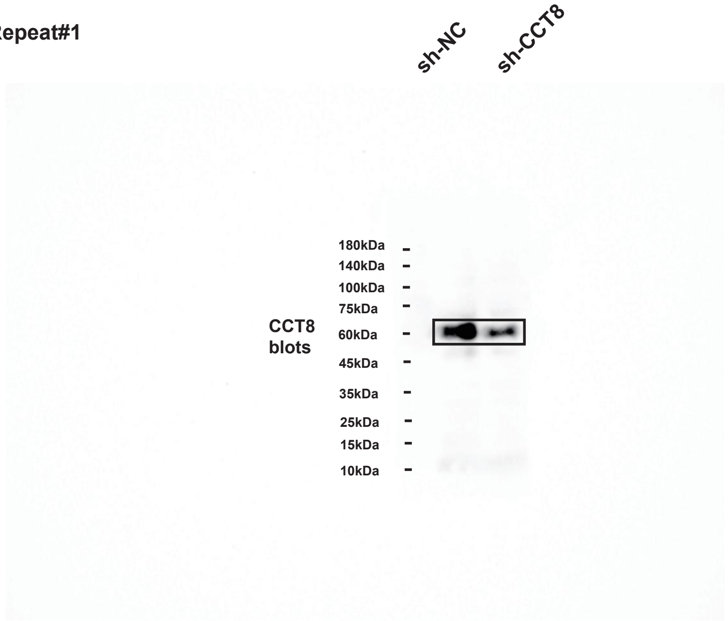


AsPC-1

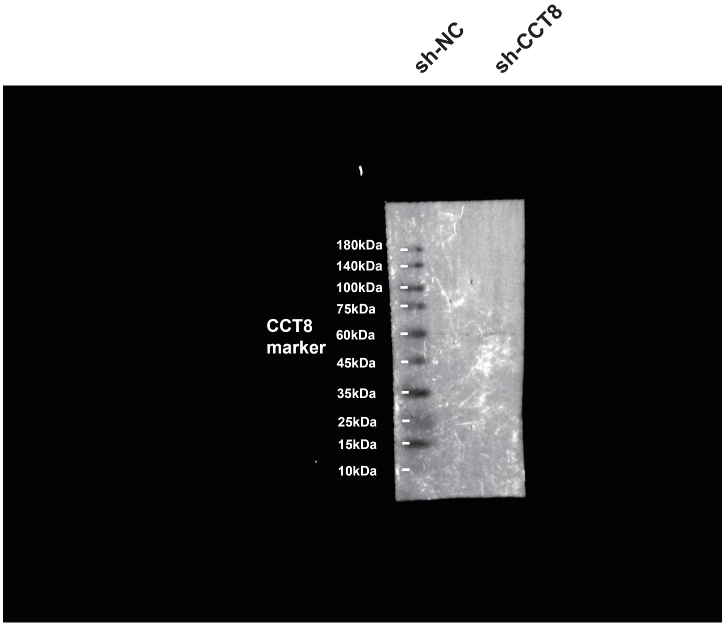


AsPC-1

Repeat#2

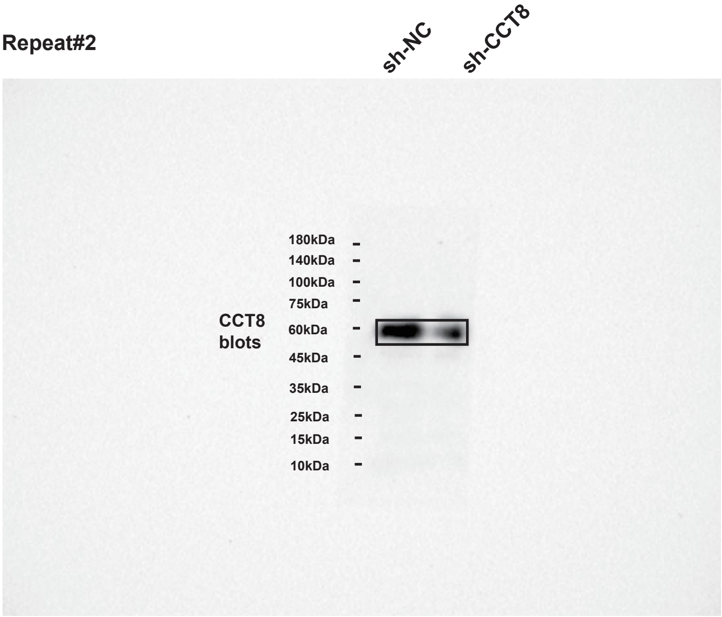


AsPC-1

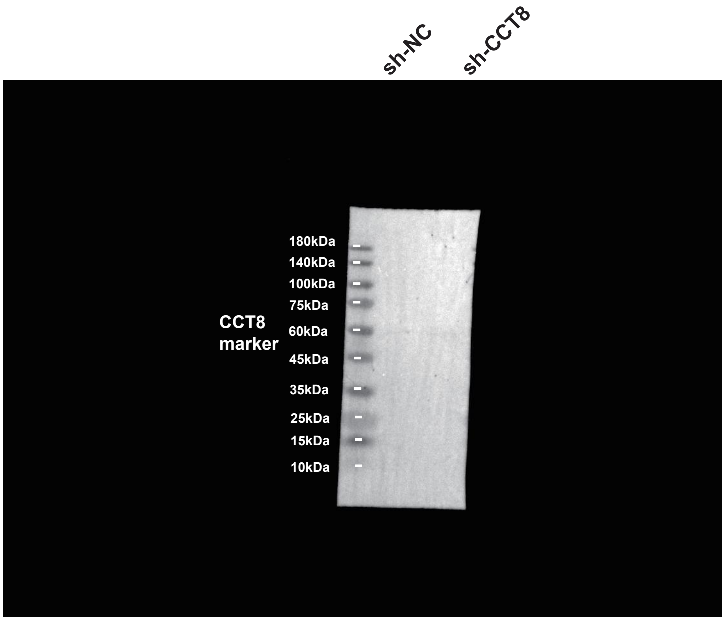


AsPC-1

Repeat#3

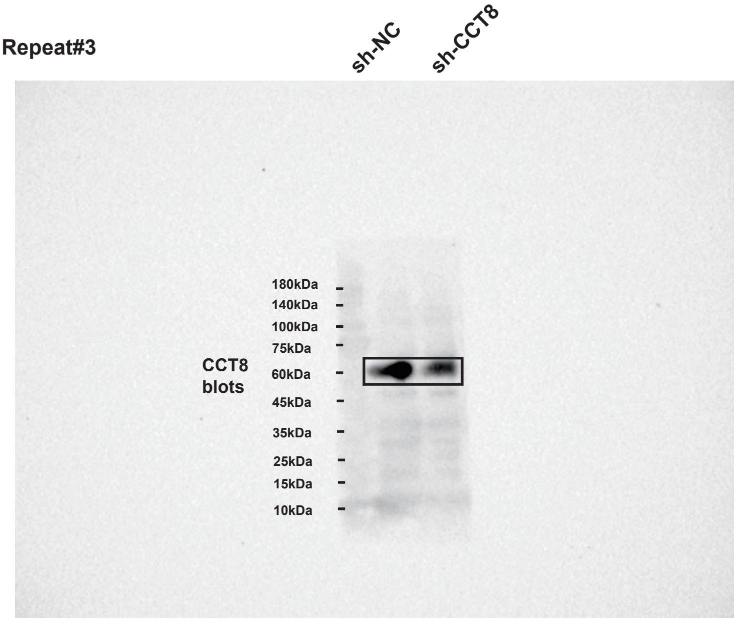


AsPC-1

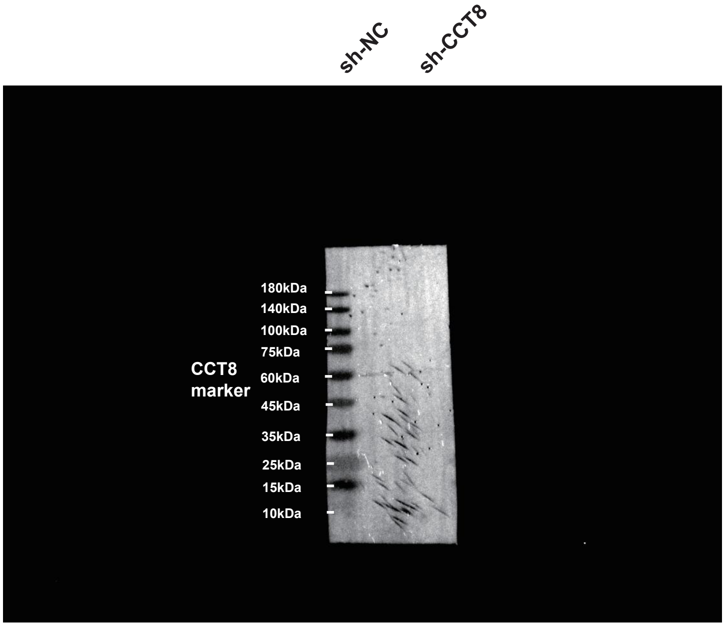


AsPC-1

Repeat#1

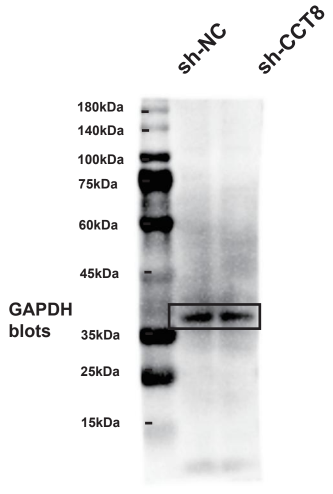


AsPC-1

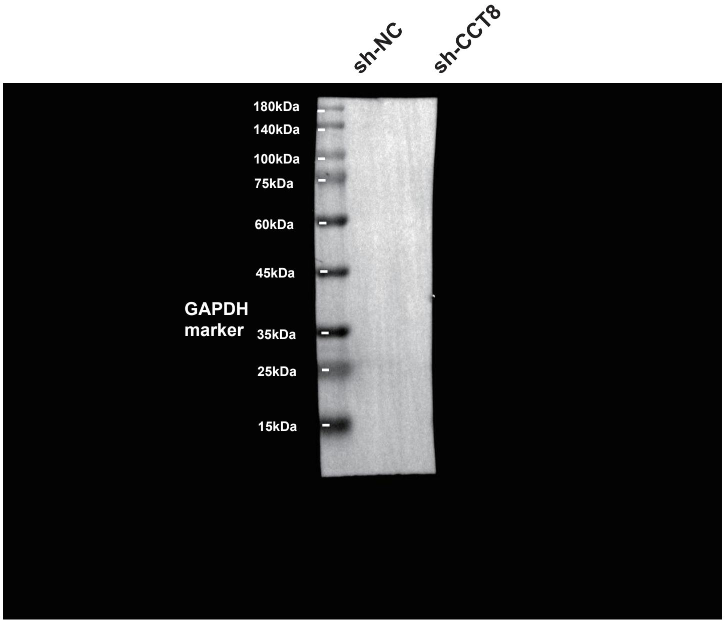


AsPC-1

Repeat#2

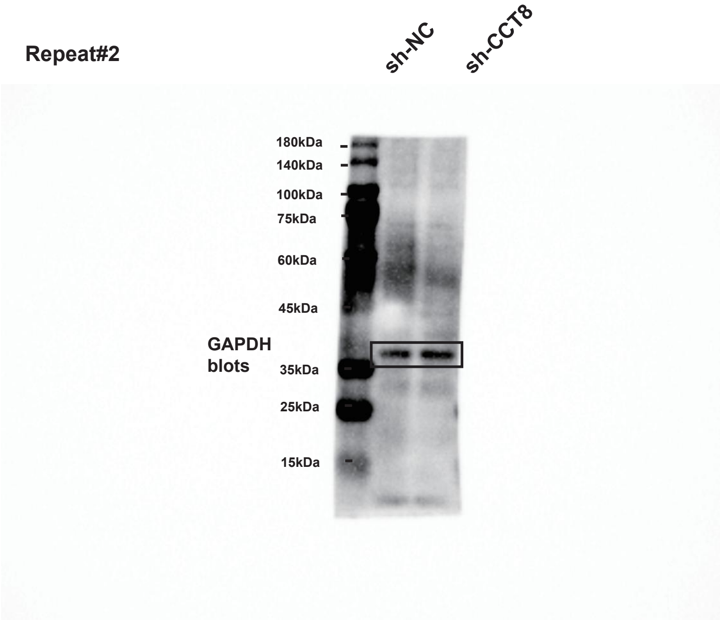


AsPC-1

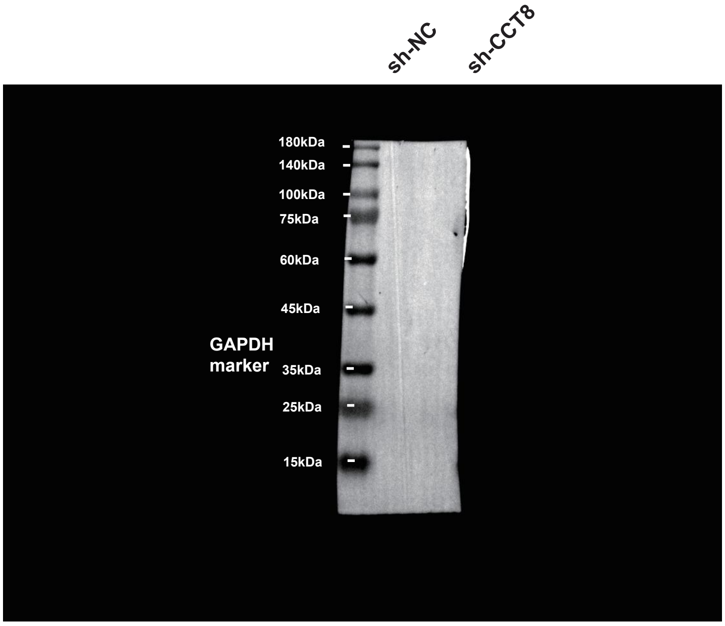


AsPC-1

Repeat#3

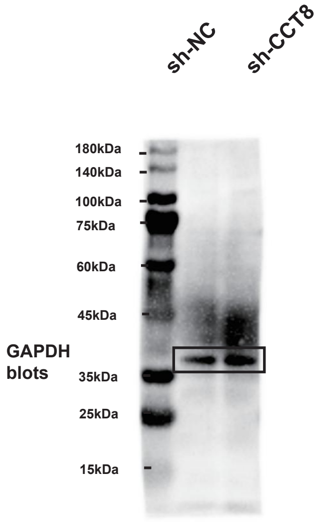


AsPC-1

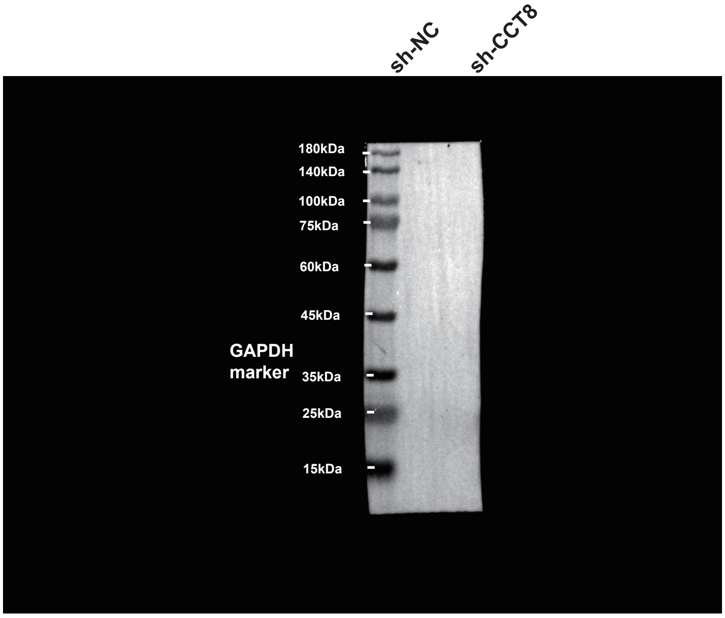


AsPC-1

**Fig. S10D**

**Repeat#1**

Exo from sh-NC sh-CCT3

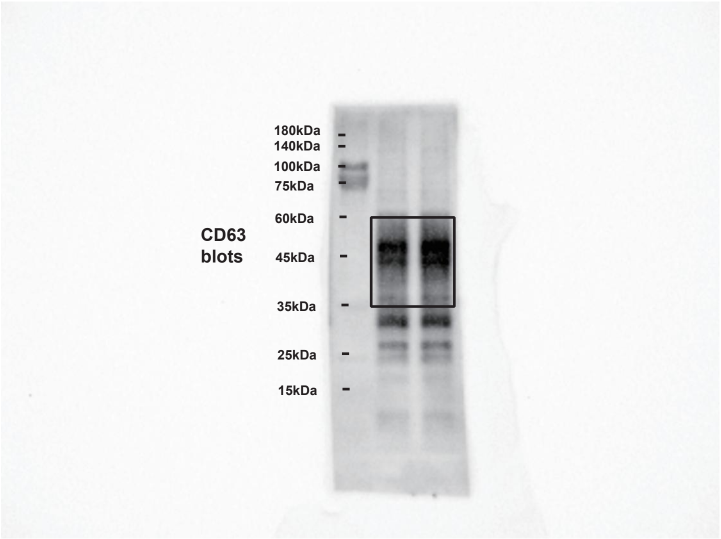


AsPC-1

Exo from sh-NC sh-CCT3

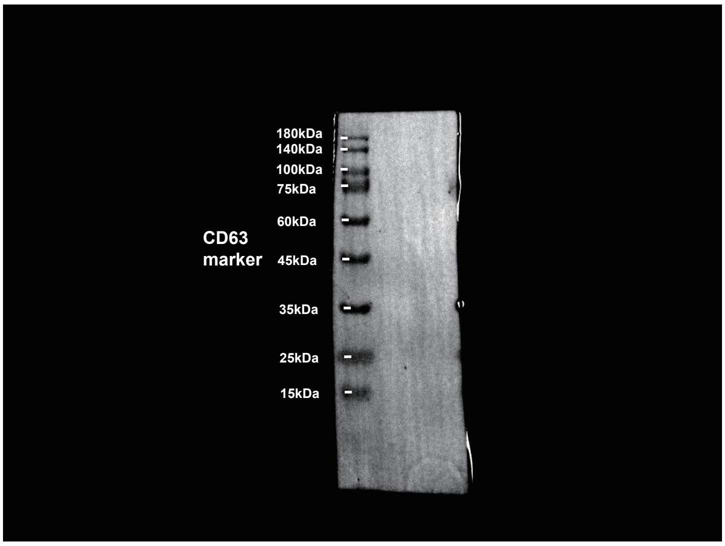


AsPC-1

**Repeat#2**

Exo from sh-NC sh-CCT3

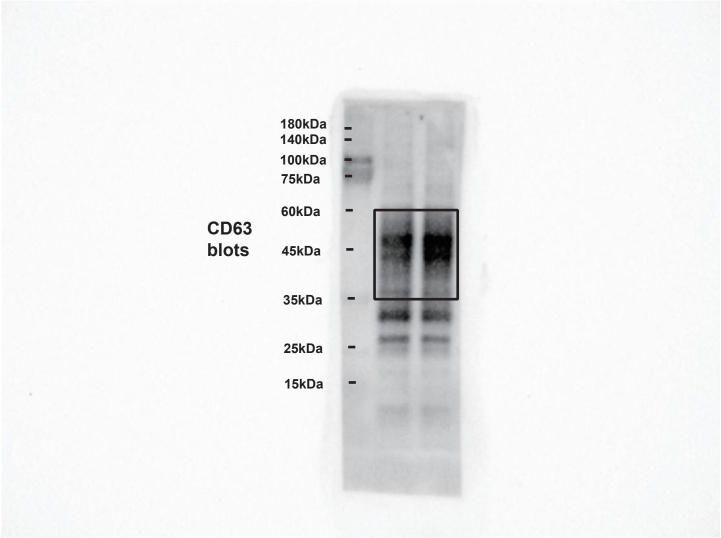


AsPC-1

Exo from sh-NC sh-CCT3

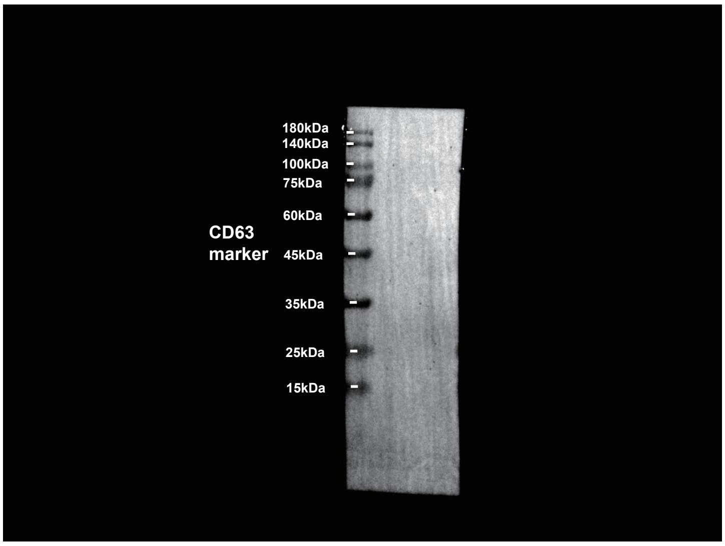


AsPC-1

**Repeat#3**

Exo from sh-NC sh-CCT3

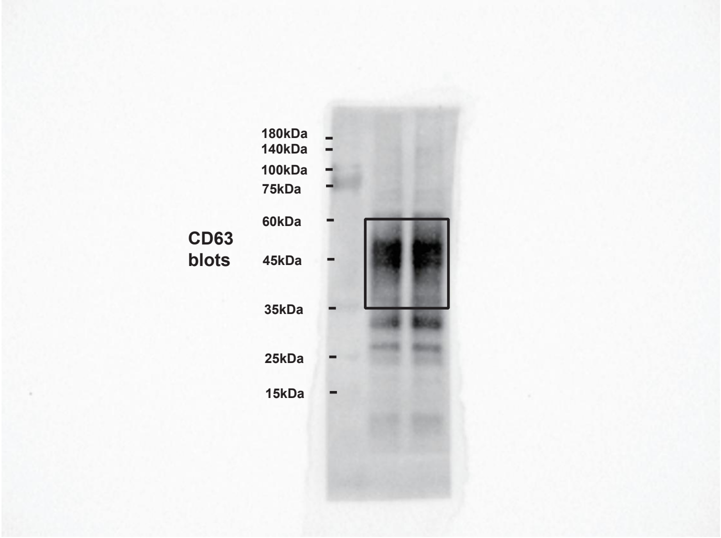


AsPC-1

Exo from sh-NC sh-CCT3

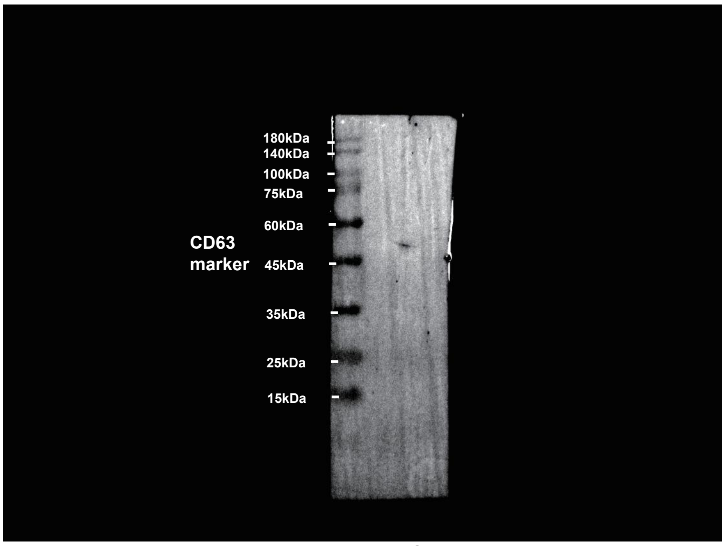


AsPC-1

Repeat#1

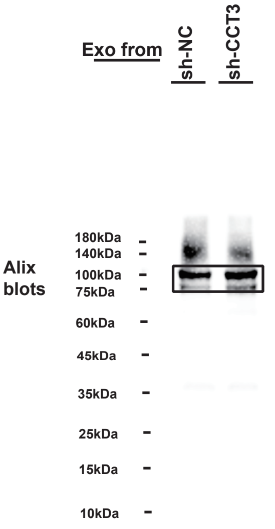


AsPC-1

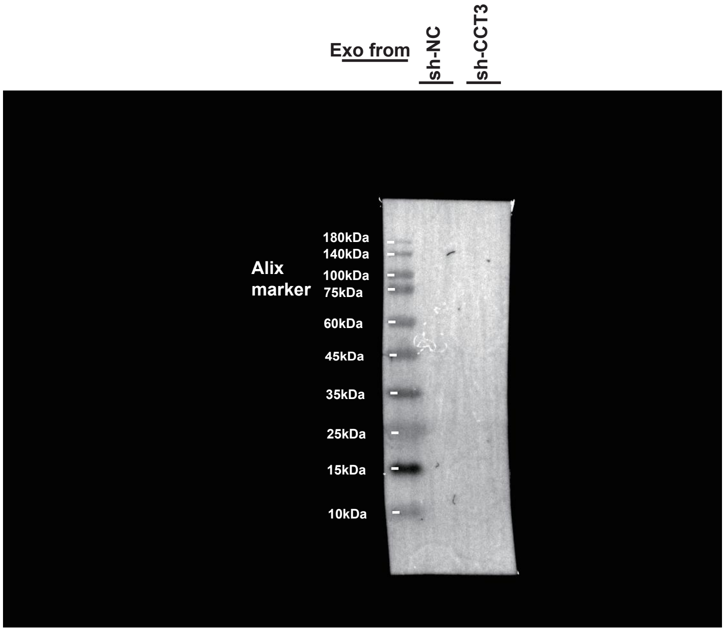


AsPC-1

Repeat#2

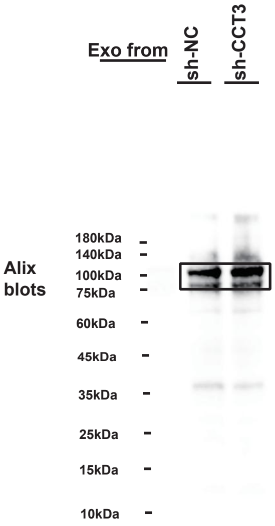


AsPC-1

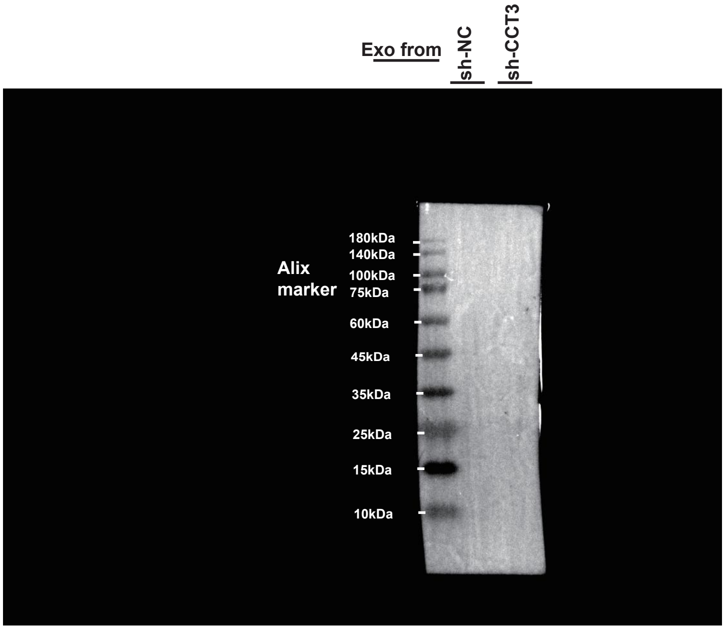


AsPC-1

Repeat#3

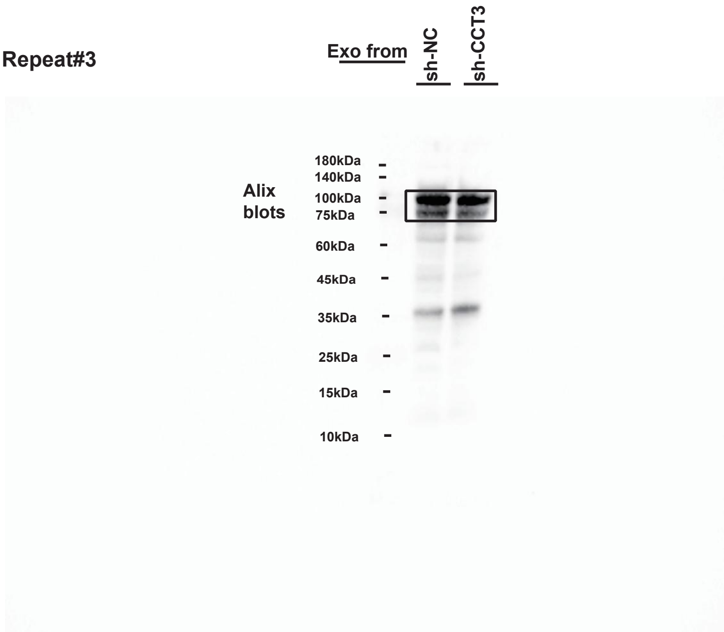


AsPC-1

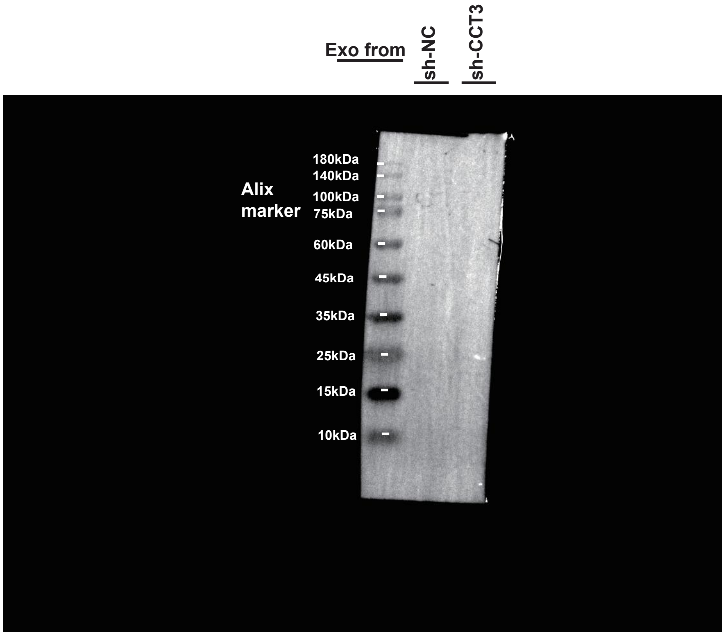


AsPC-1

**Repeat#1**

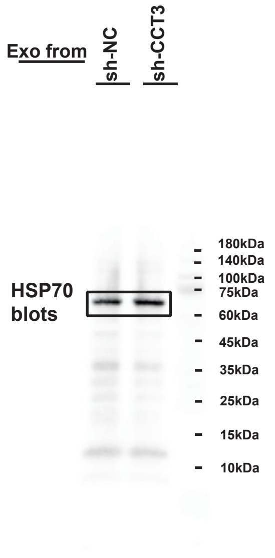


AsPC-1

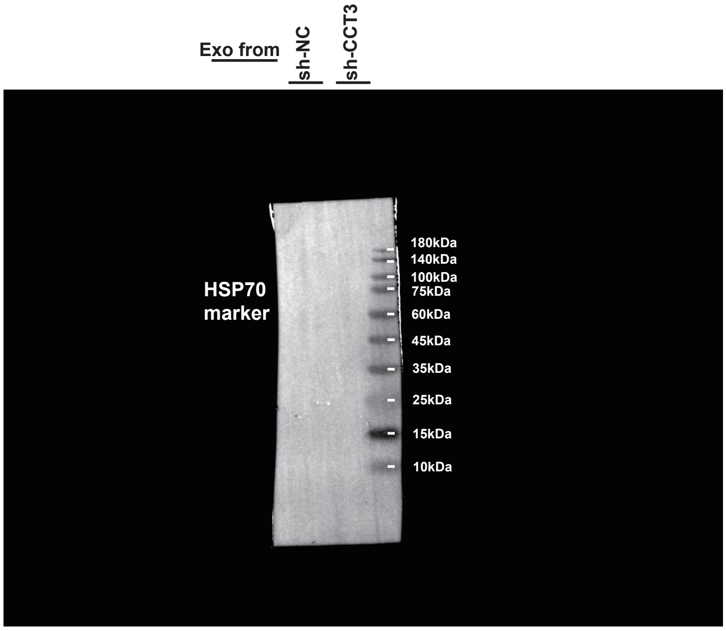


AsPC-1

**Repeat#2**

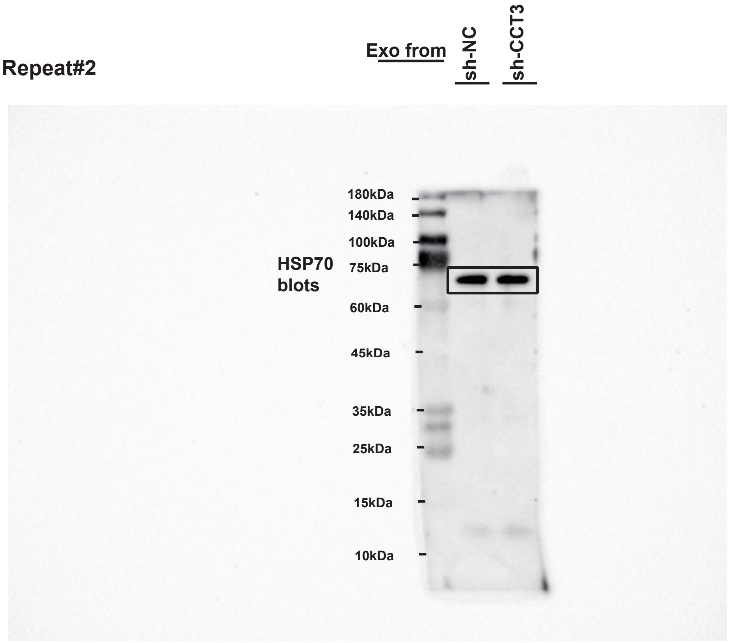


AsPC-1

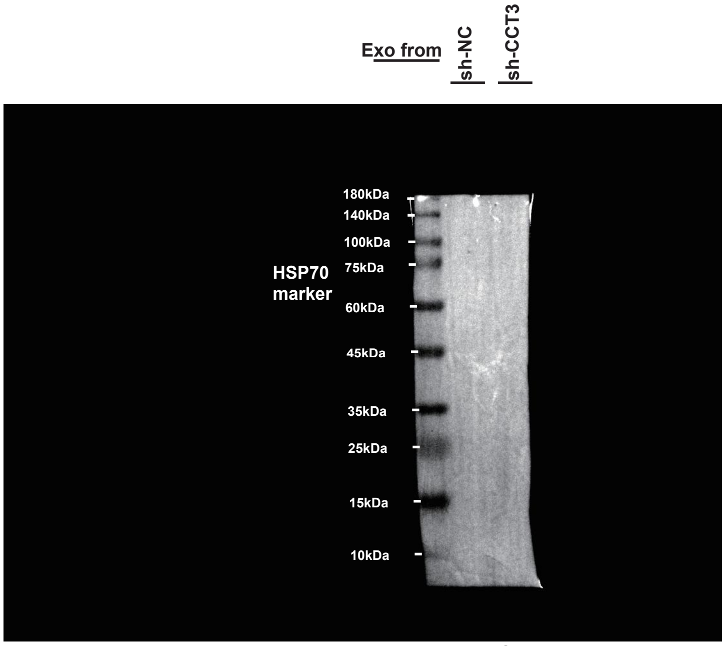


AsPC-1

**Repeat#3**

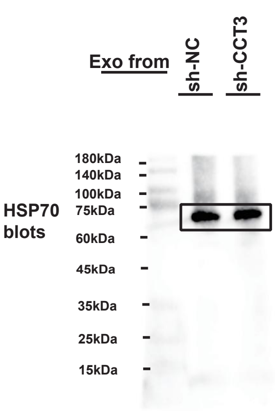


AsPC-1

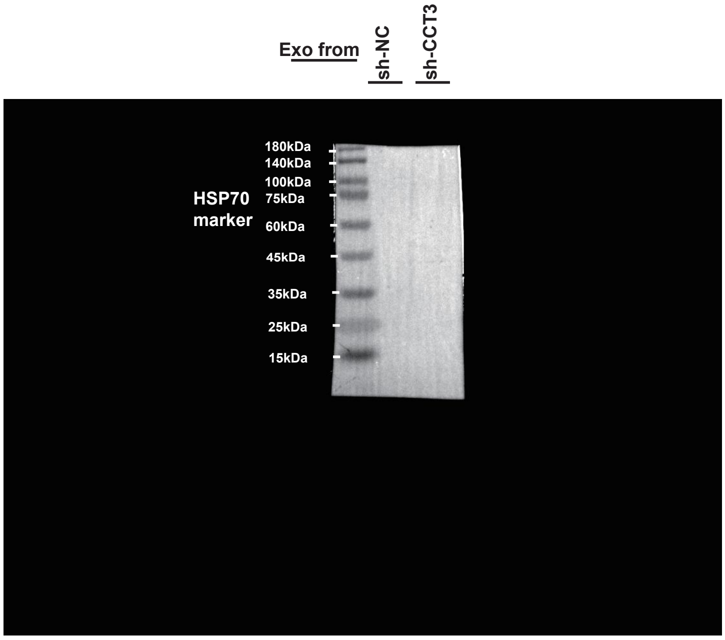


AsPC-1

Repeat#1

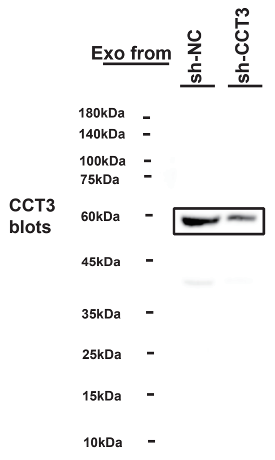


AsPC-1

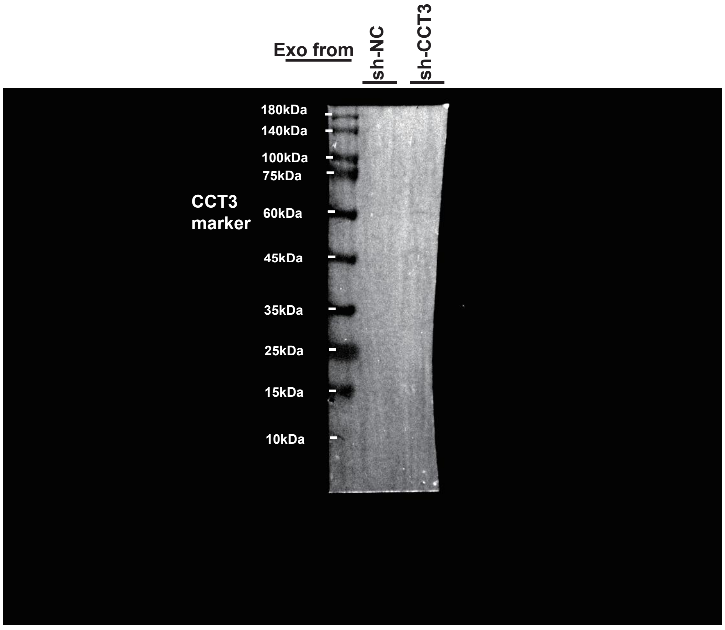


AsPC-1

Repeat#2

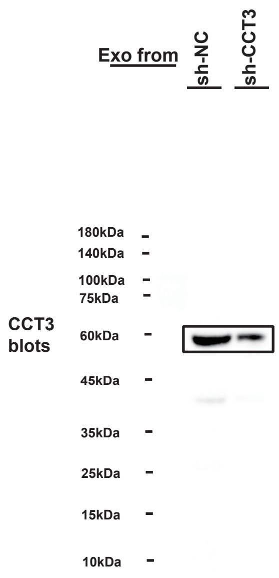


AsPC-1

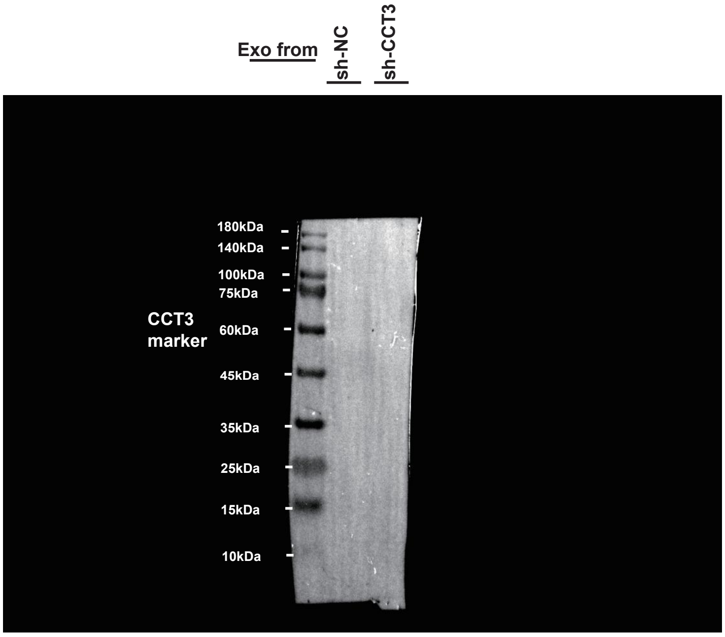


AsPC-1

Repeat#3

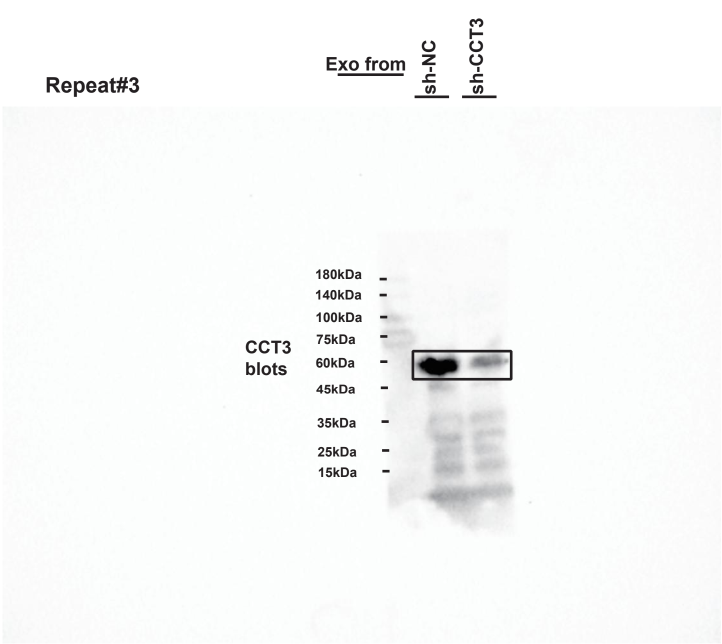


AsPC-1

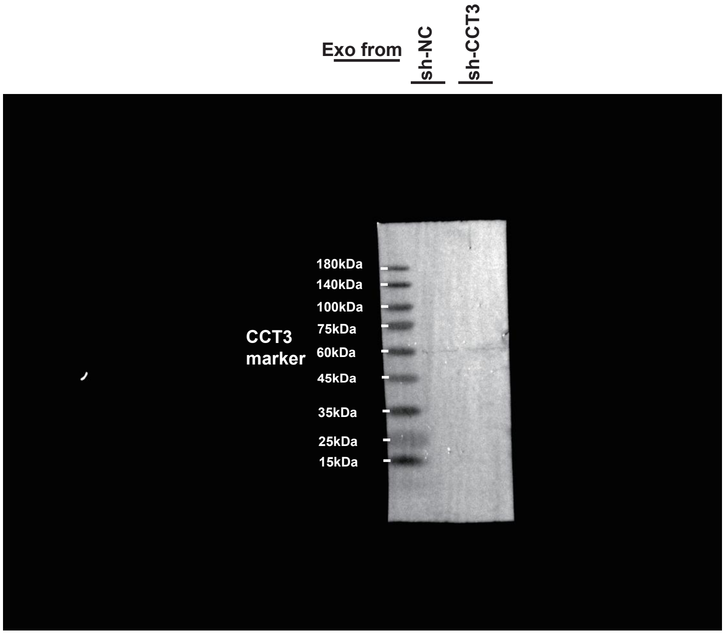


AsPC-1

Repeat#1

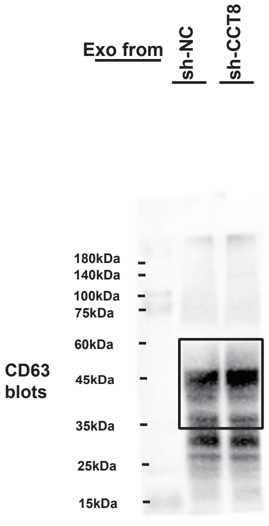


AsPC-1

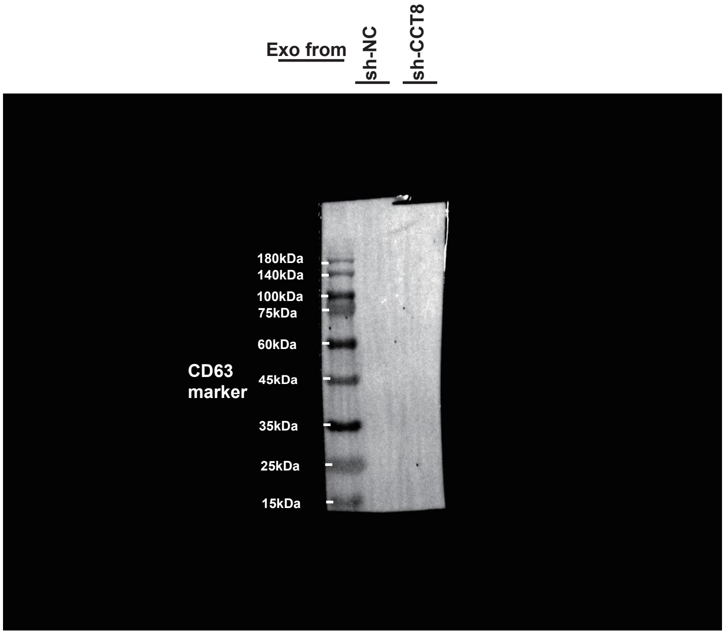


AsPC-1

Repeat#2

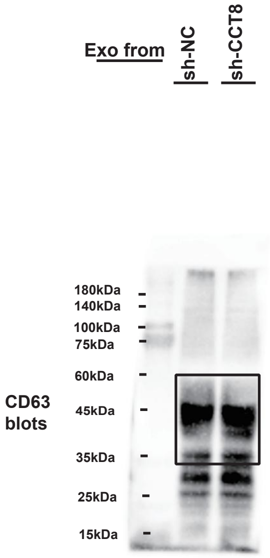


AsPC-1

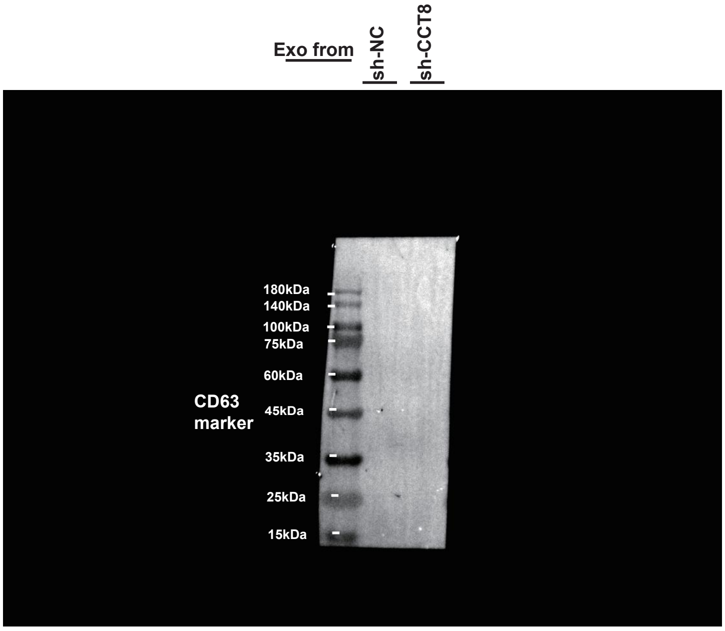


AsPC-1

Repeat#3

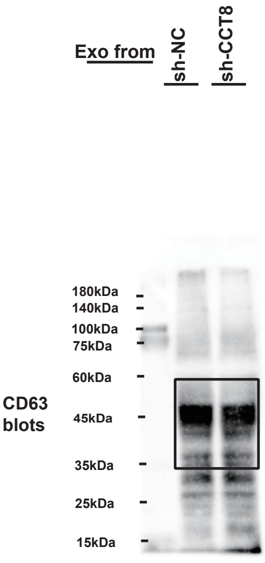


AsPC-1

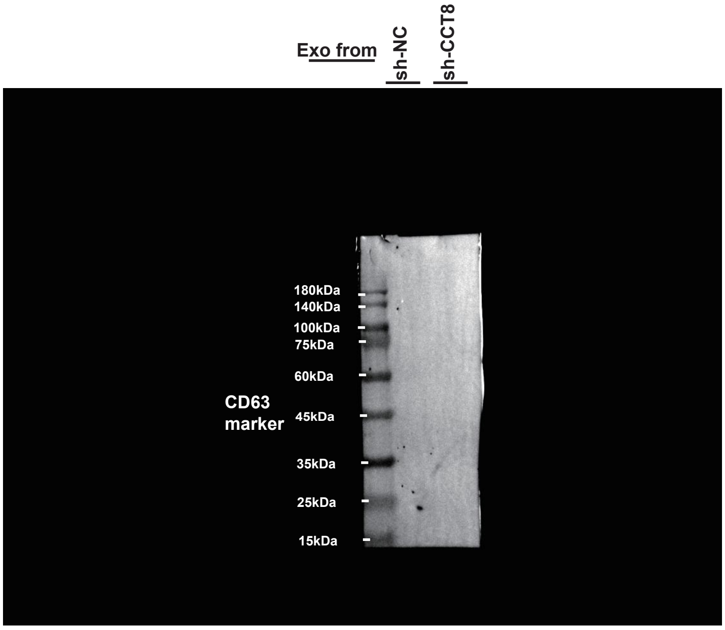


AsPC-1

Repeat#1

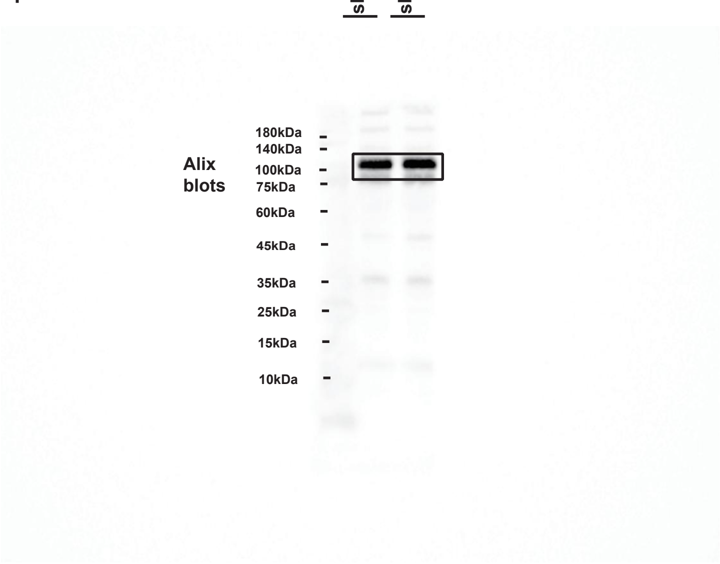


AsPC-1

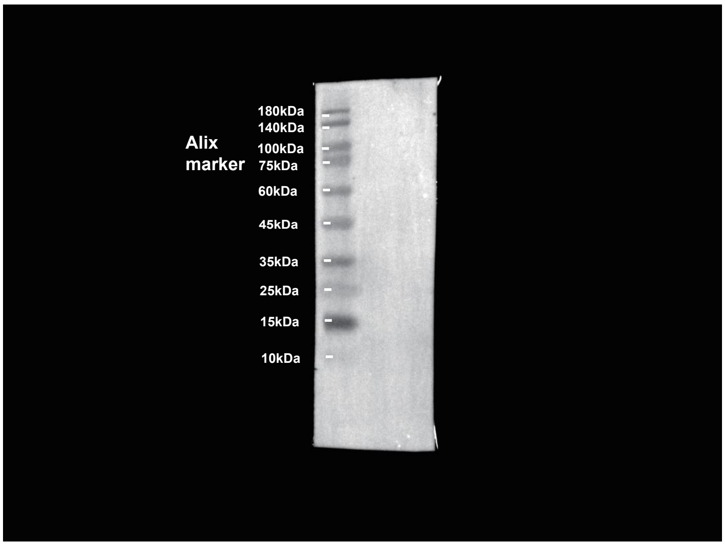


AsPC-1

Repeat#2

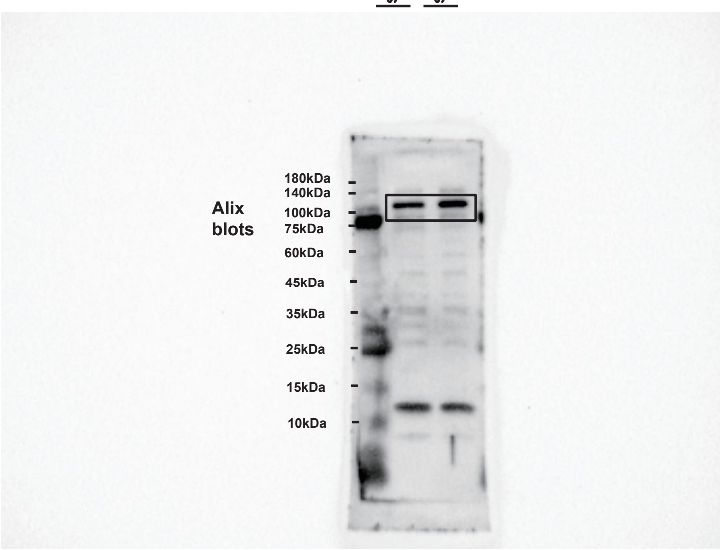


AsPC-1

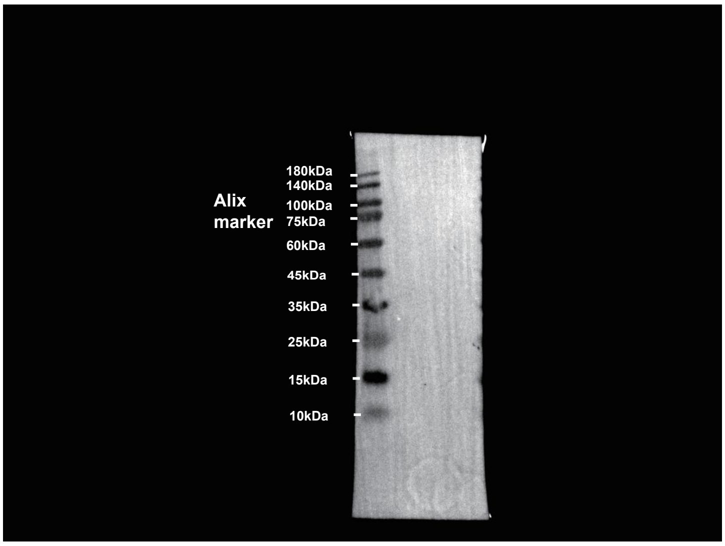


AsPC-1

Repeat#3

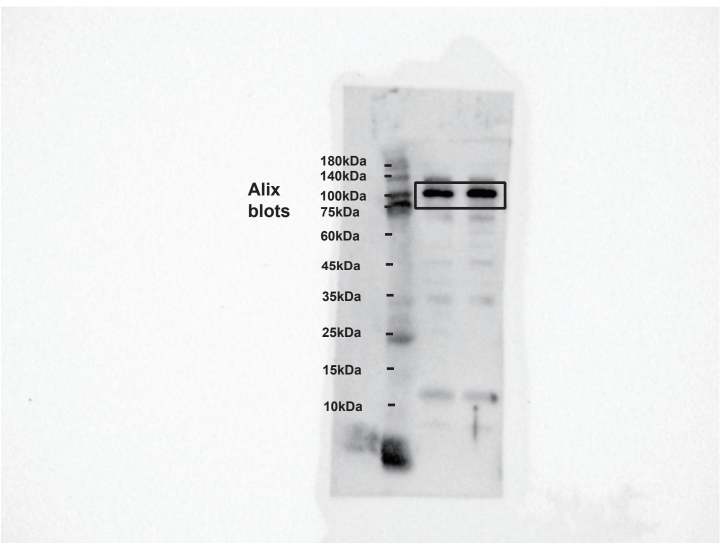


AsPC-1

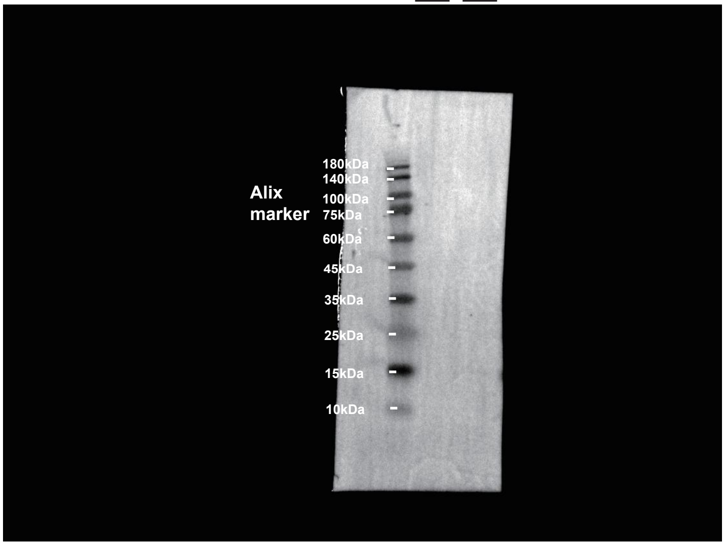


AsPC-1

## Repeat#1

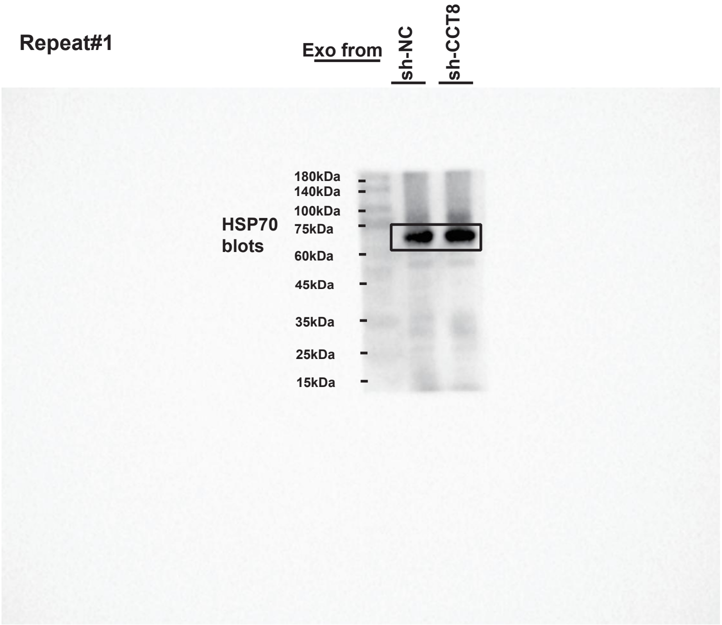


AsPC-1

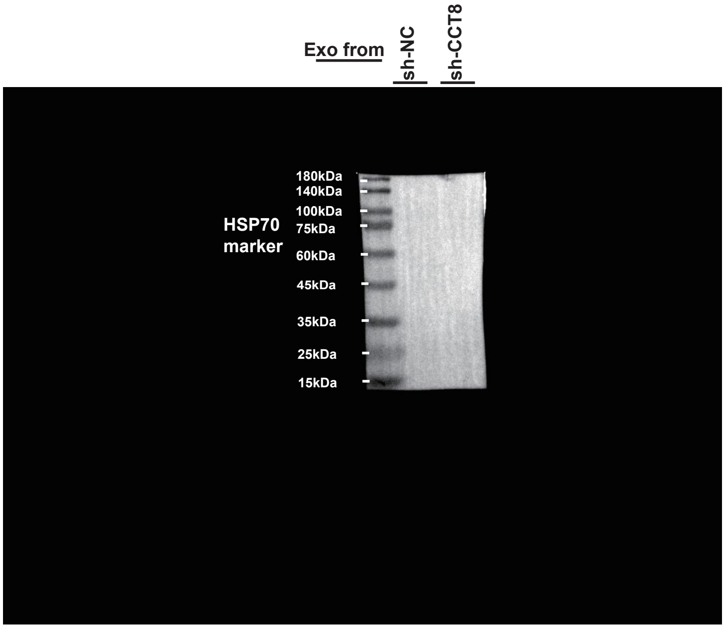


AsPC-1

## Repeat#2

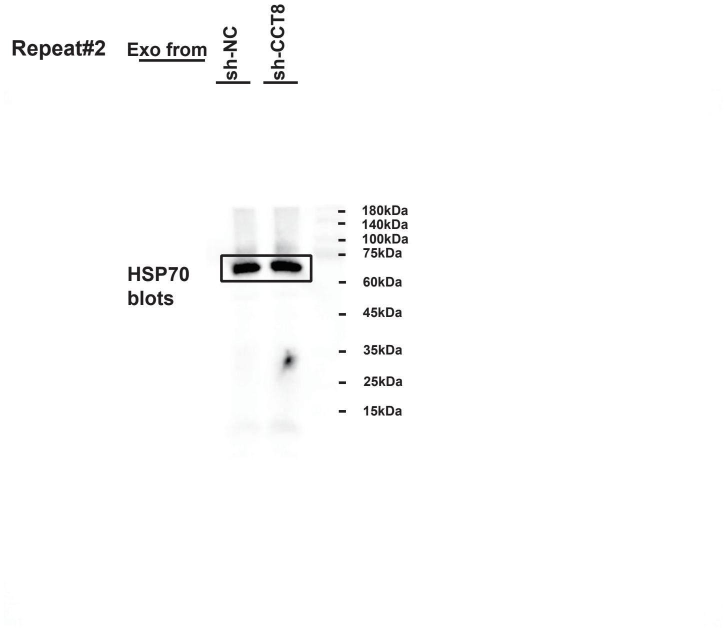


AsPC-1

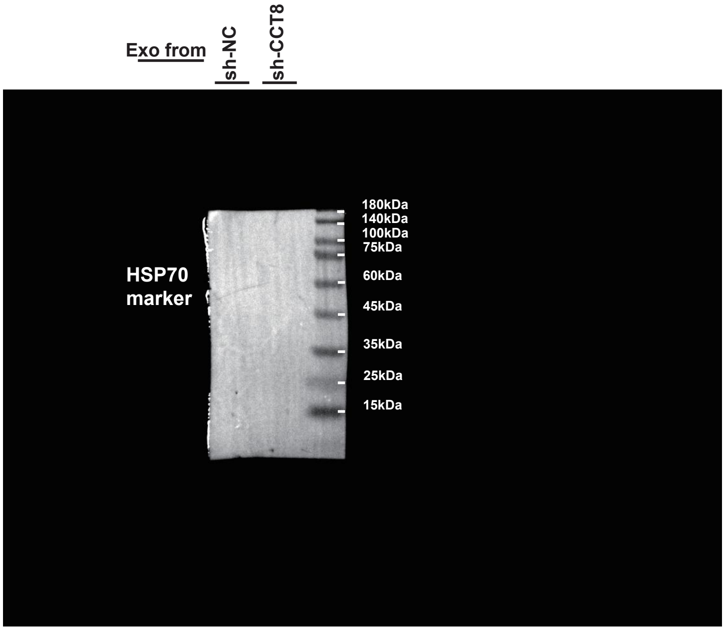


AsPC-1

## Repeat#3

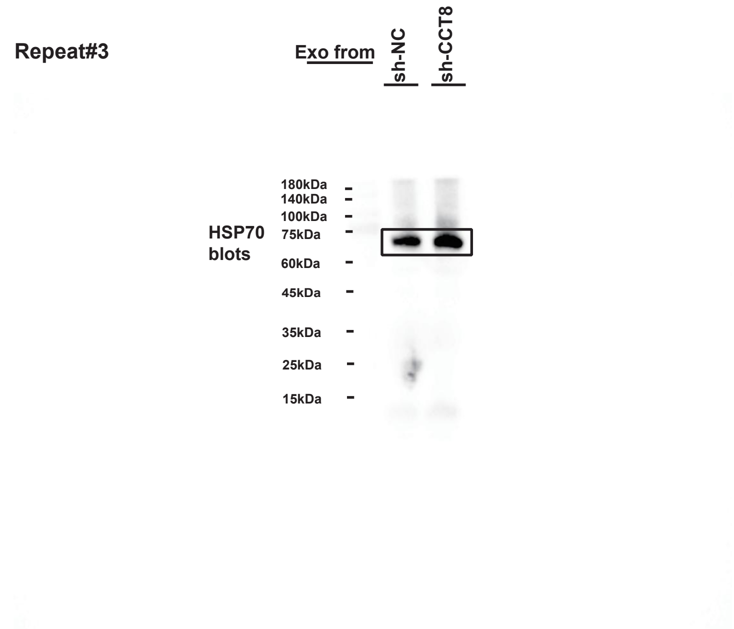


AsPC-1

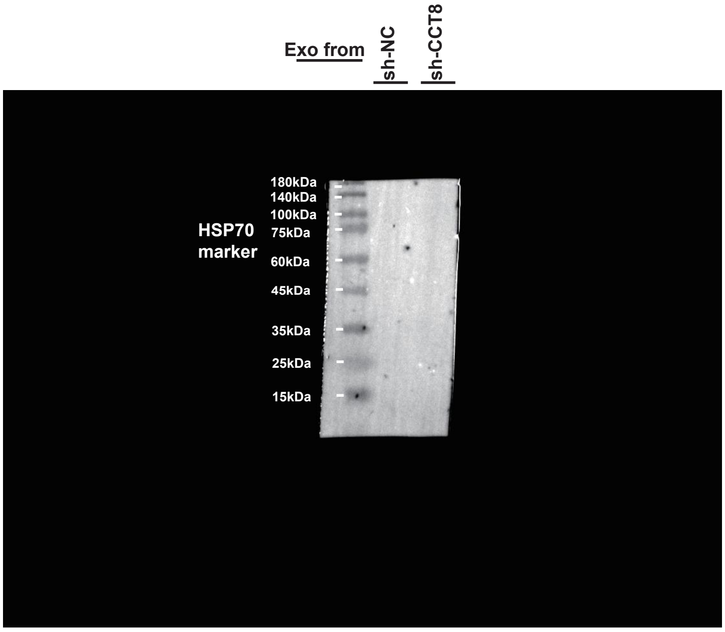


AsPC-1

Repeat#1

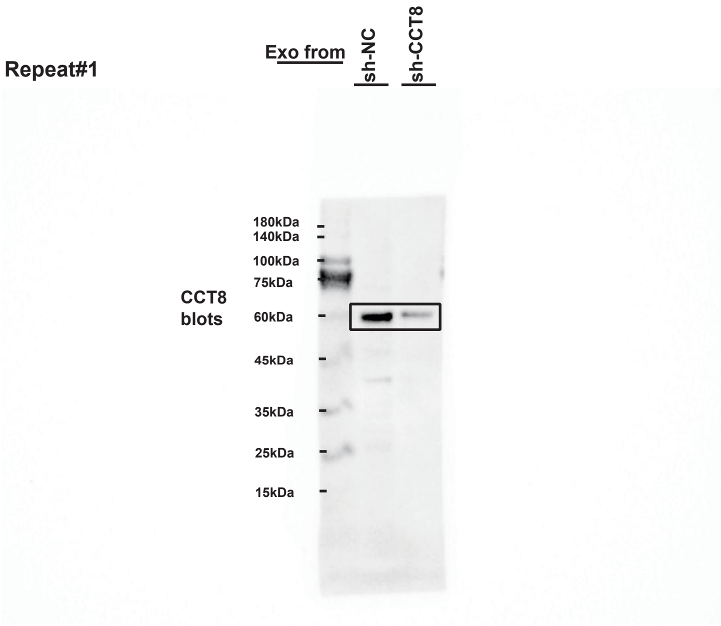


AsPC-1

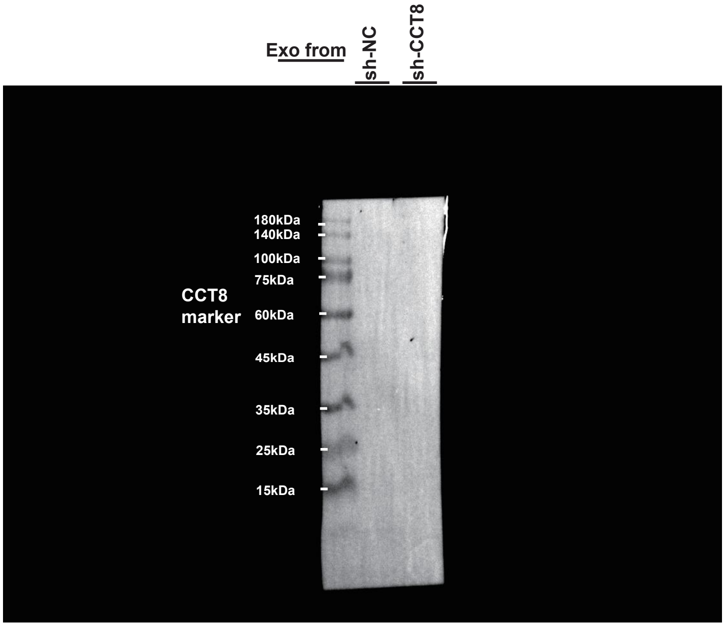


AsPC-1

Repeat#2

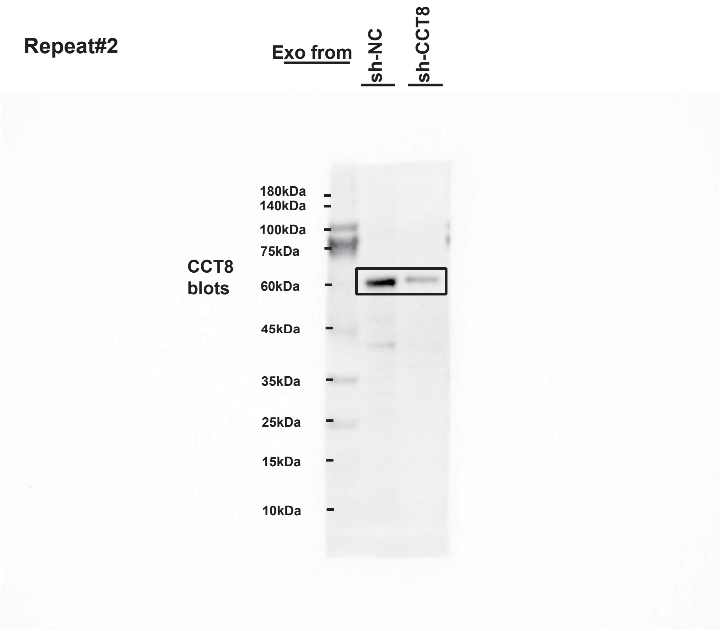


AsPC-1

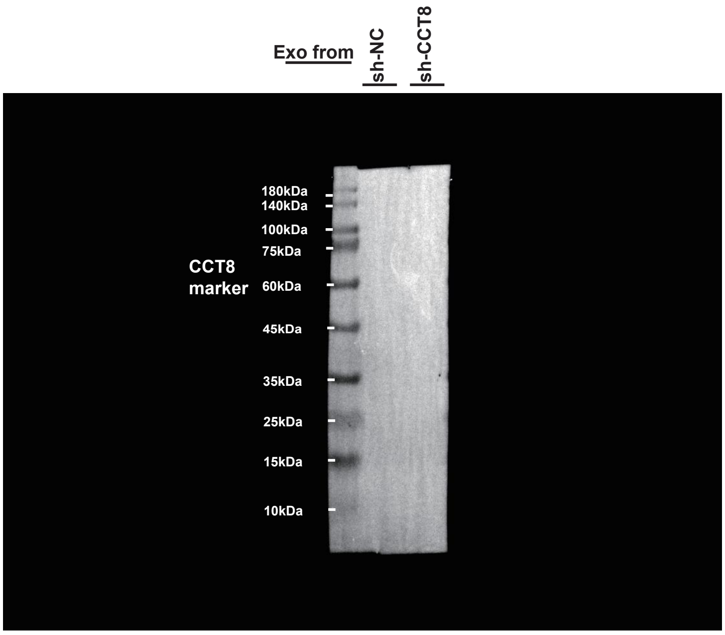


AsPC-1

Repeat#3

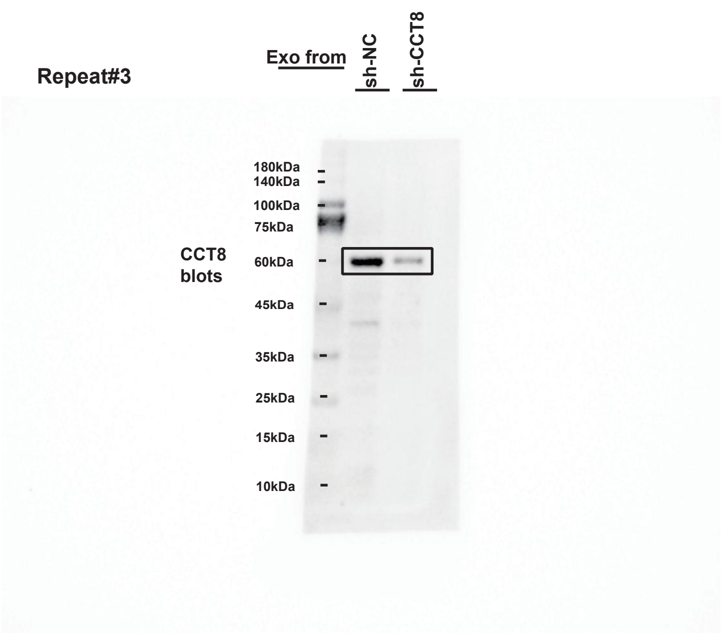


AsPC-1

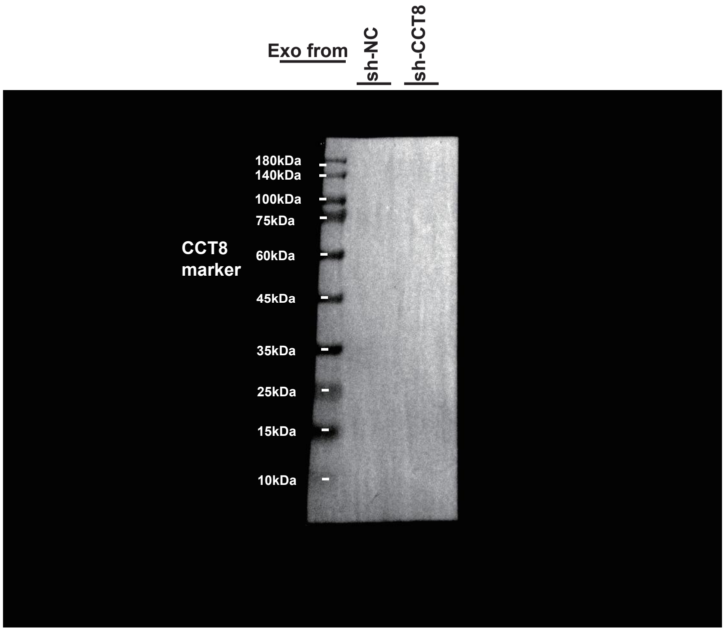


AsPC-1

Fig. S10E

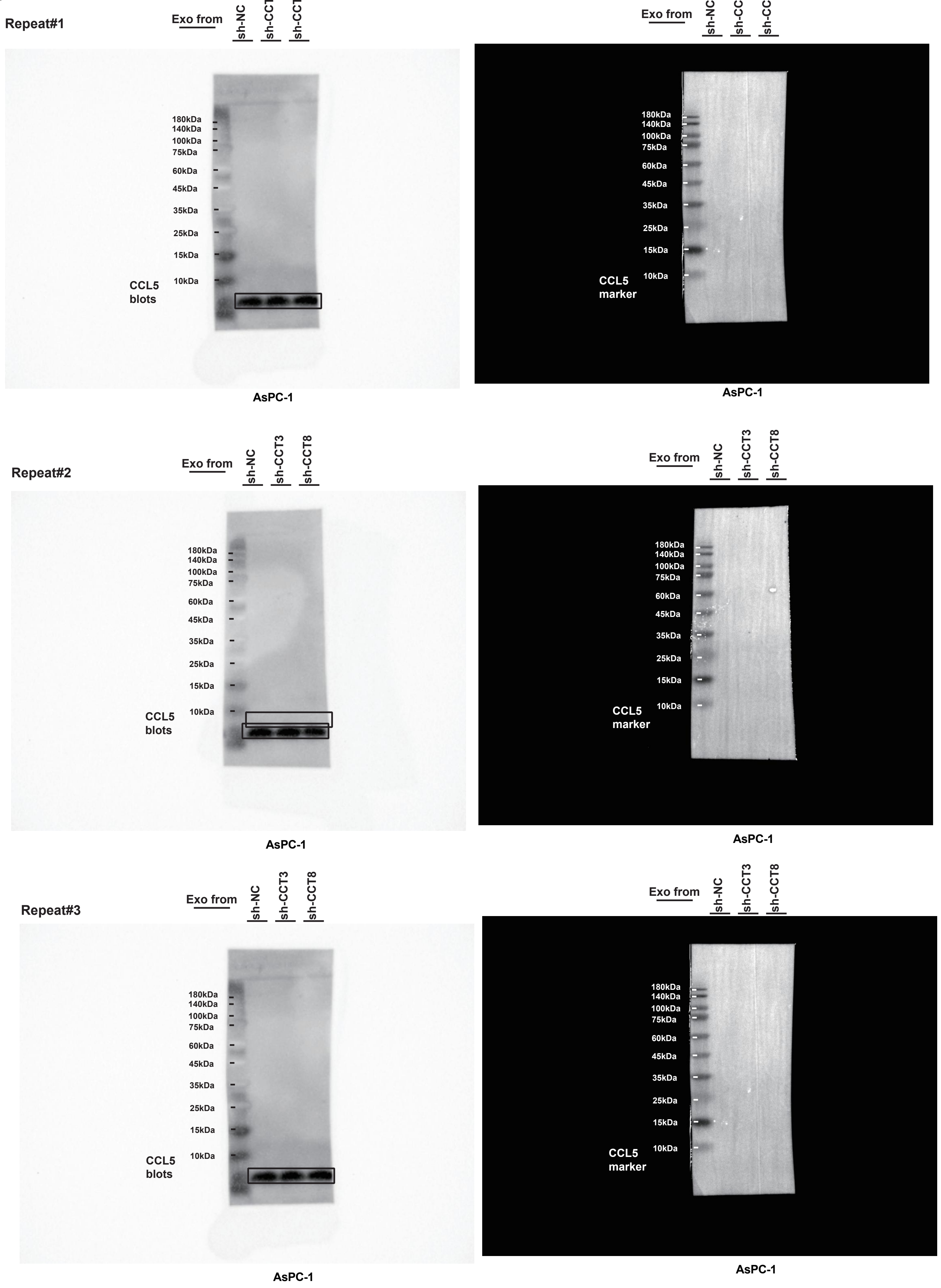
Repeat#1
Exo from
sh-NC
sh-CCT3
sh-CCT8
180kDa
140kDa
100kDa
75kDa
60kDa
45kDa
35kDa
25kDa
15kDa
10kDa
CCL5
blots
AsPC-1
Exo from
sh-NC
sh-CCT3
sh-CCT8
180kDa
140kDa
100kDa
75kDa
60kDa
45kDa
35kDa
25kDa
15kDa
10kDa
CCL5
marker
AsPC-1
Repeat#2
Exo from
sh-NC
sh-CCT3
sh-CCT8
180kDa
140kDa
100kDa
75kDa
60kDa
45kDa
35kDa
25kDa
15kDa
10kDa
CCL5
blots
AsPC-1
Exo from
sh-NC
sh-CCT3
sh-CCT8
180kDa
140kDa
100kDa
75kDa
60kDa
45kDa
35kDa
25kDa
15kDa
10kDa
CCL5
marker
AsPC-1
Repeat#3
Exo from
sh-NC
sh-CCT3
sh-CCT8
180kDa
140kDa
100kDa
75kDa
60kDa
45kDa
35kDa
25kDa
15kDa
10kDa
CCL5
blots
AsPC-1
Exo from
sh-NC
sh-CCT3
sh-CCT8
180kDa
140kDa
100kDa
75kDa
60kDa
45kDa
35kDa
25kDa
15kDa
10kDa
CCL5
marker
AsPC-1

Repeat#1

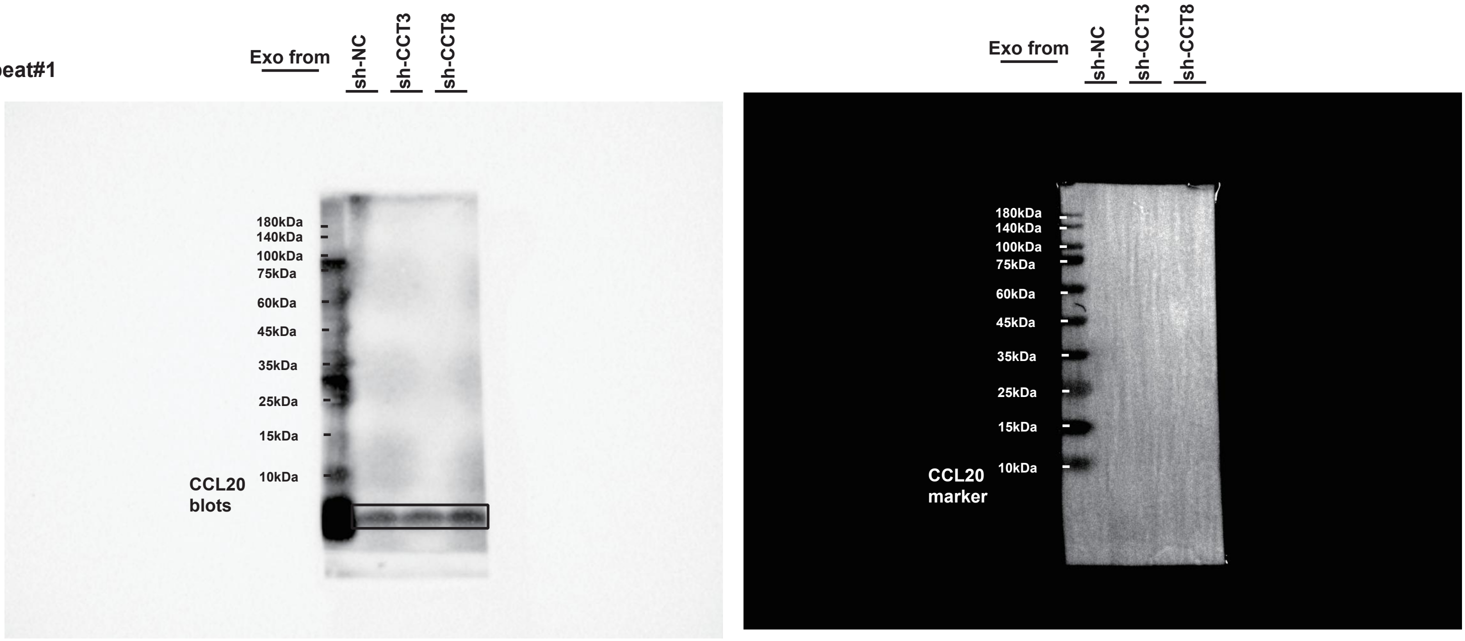


AsPC-1

AsPC-1

Repeat#2

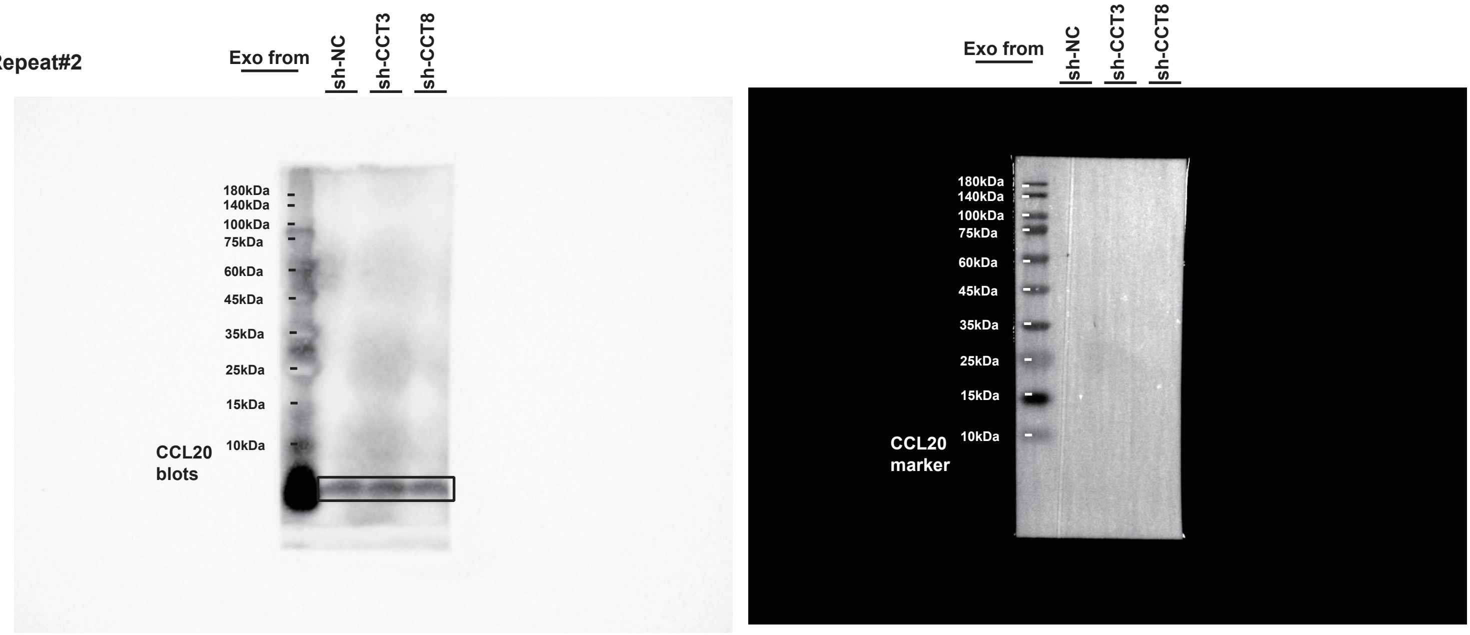


AsPC-1

AsPC-1

Repeat#3

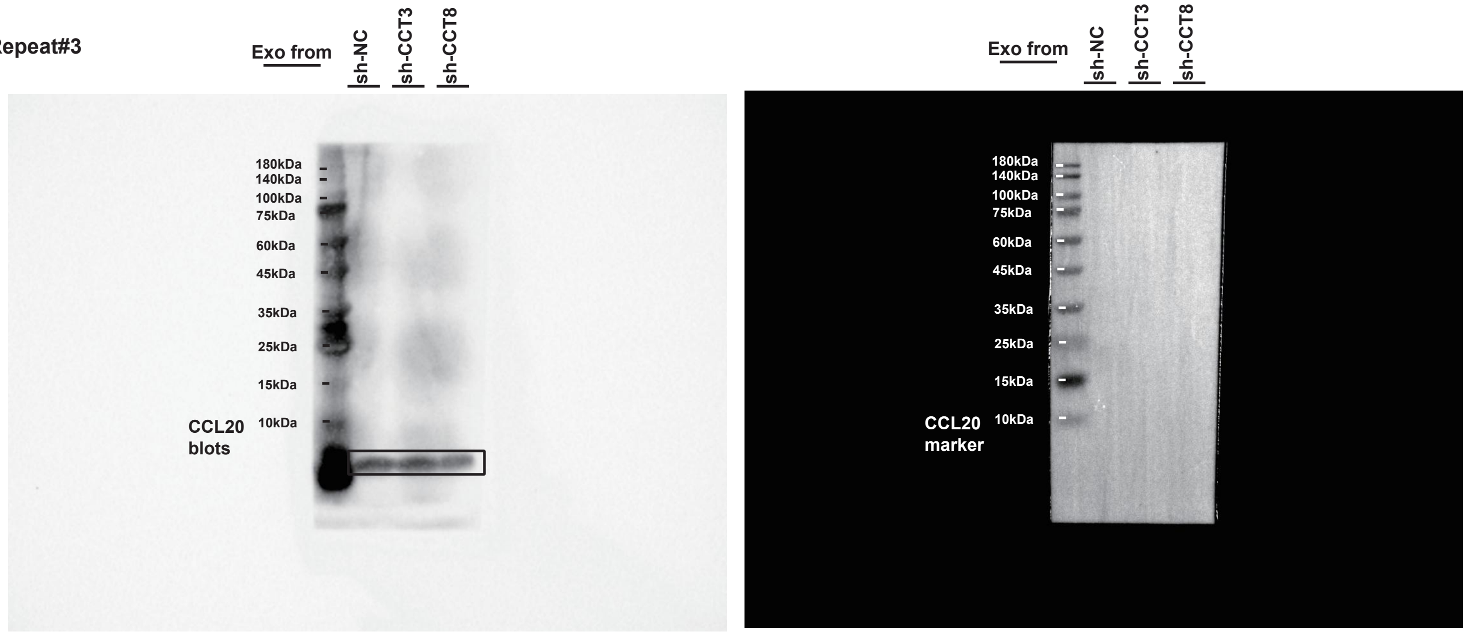


AsPC-1

AsPC-1

Repeat#1

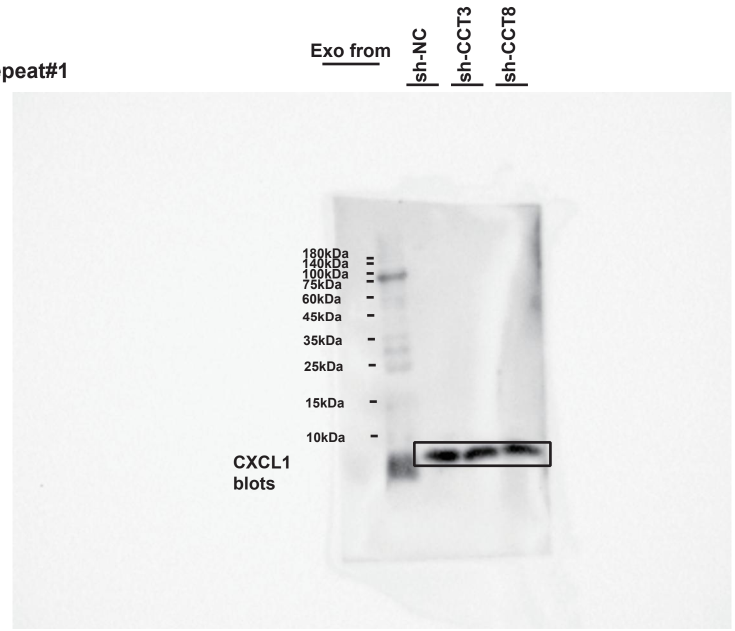


AsPC-1

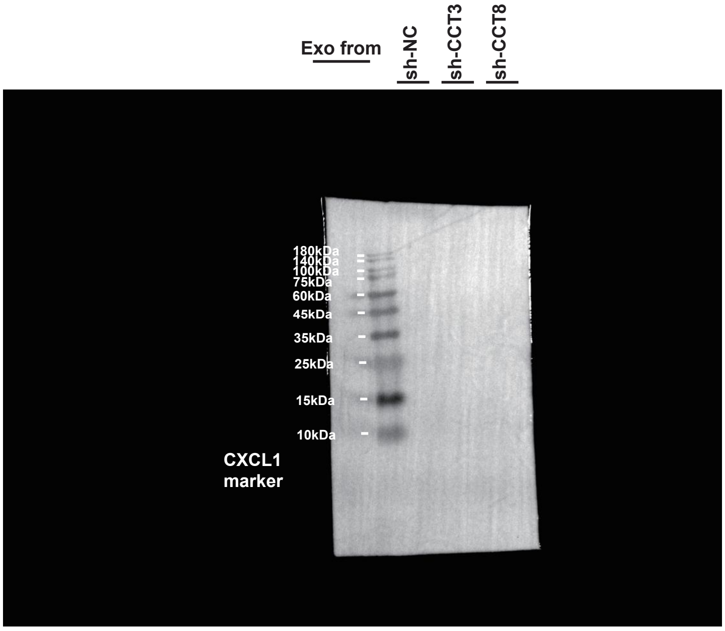


AsPC-1

Repeat#2

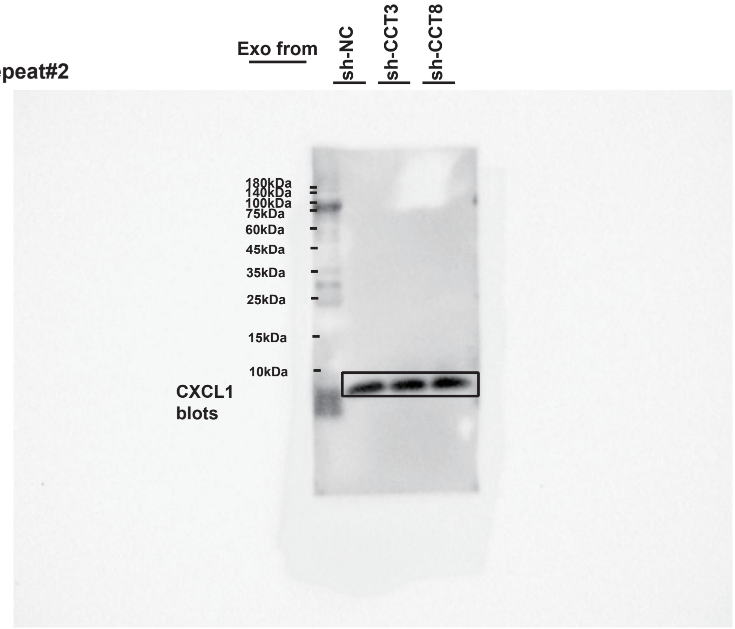


AsPC-1

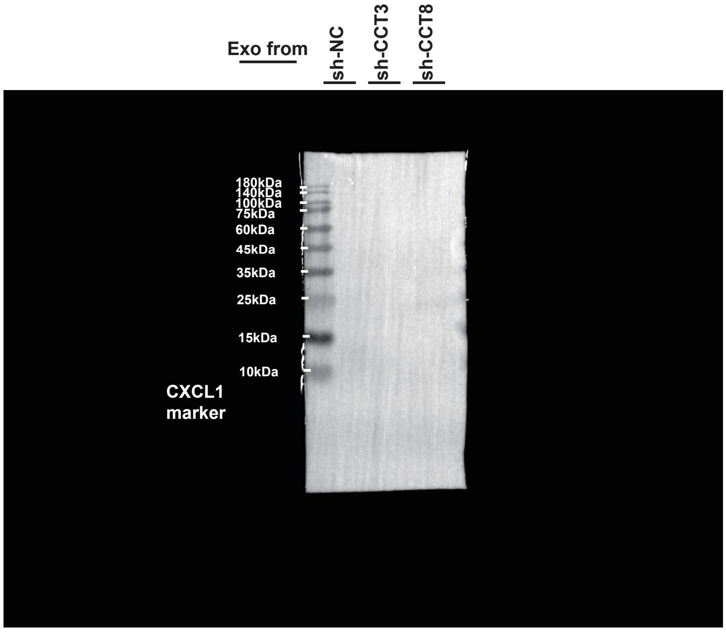


AsPC-1

Repeat#3

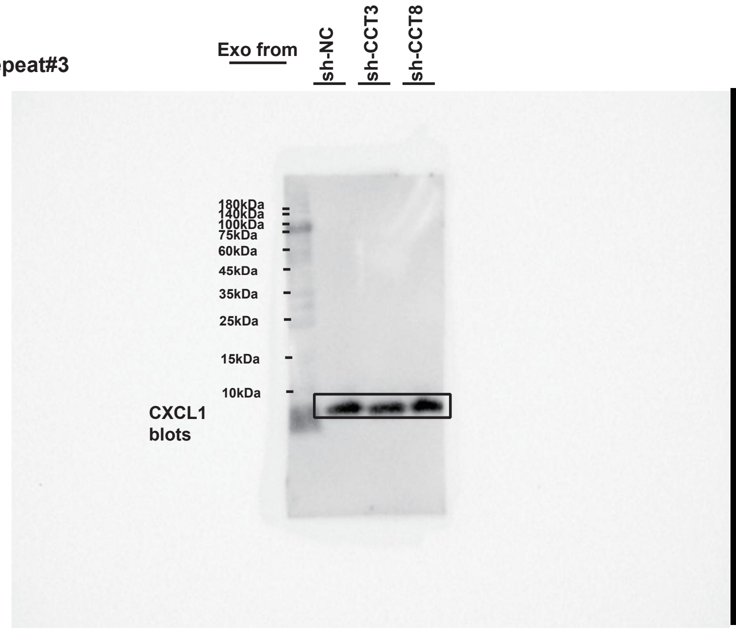


AsPC-1

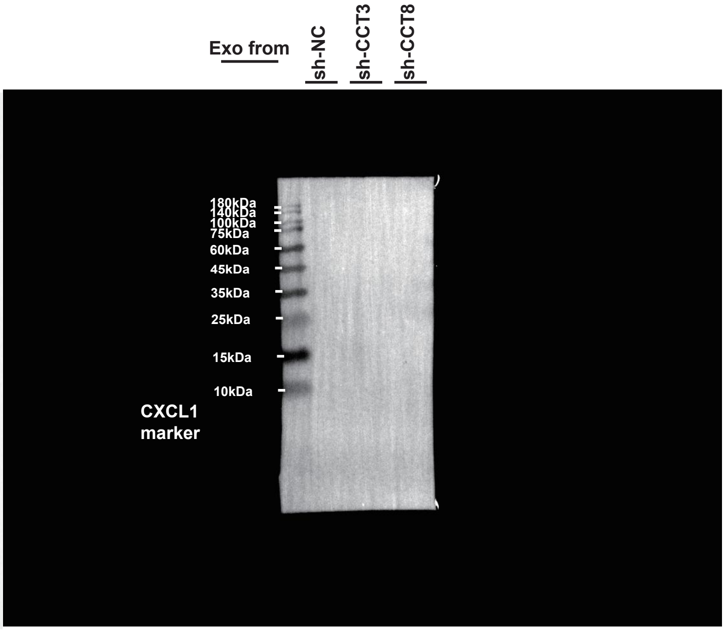


AsPC-1

Repeat#1

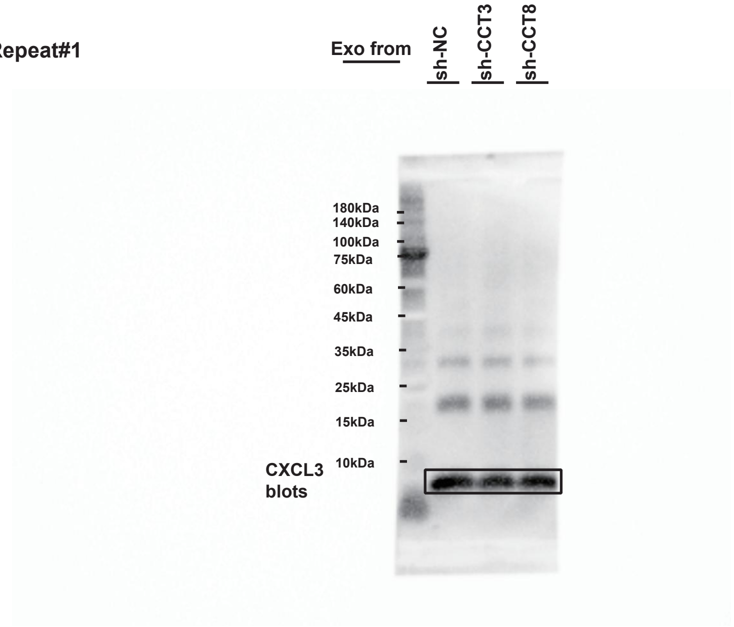


AsPC-1

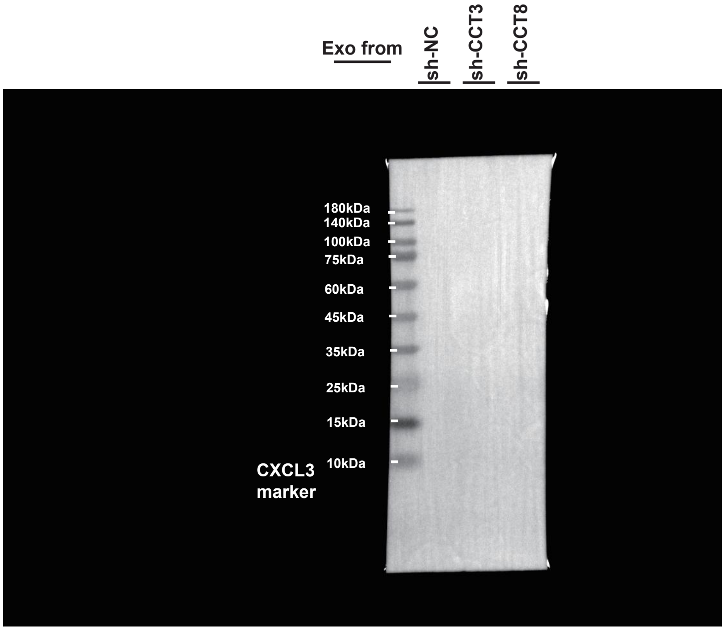


AsPC-1

Repeat#2

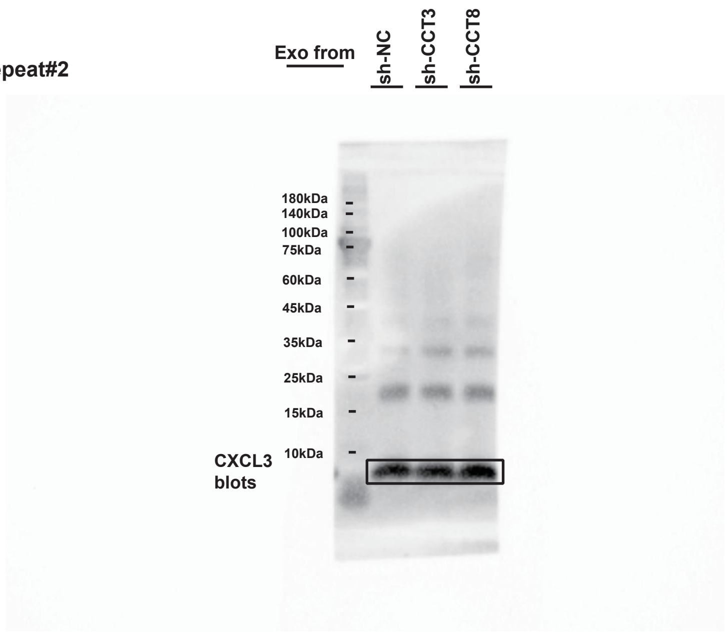


AsPC-1

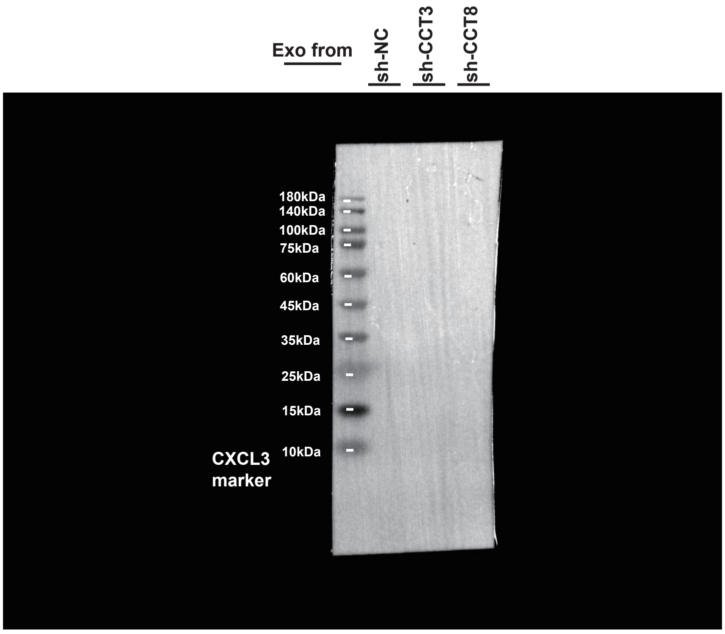


AsPC-1

Repeat#3

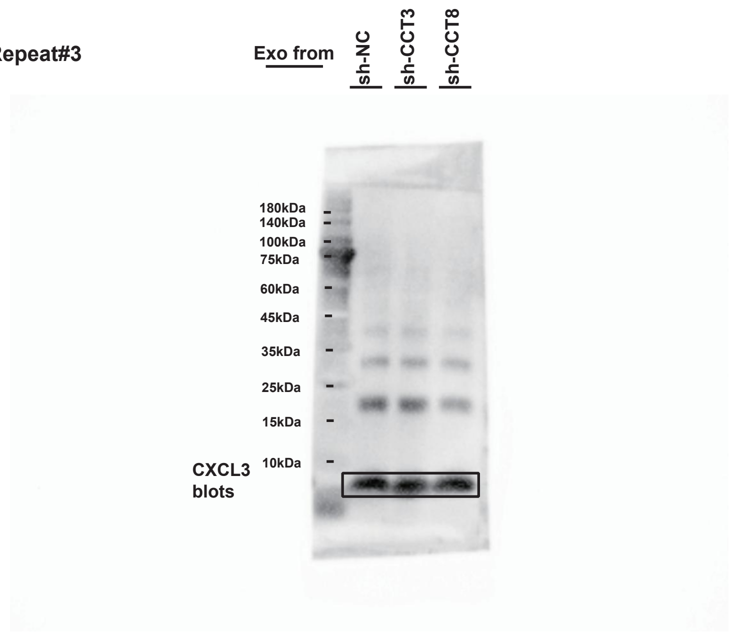


AsPC-1

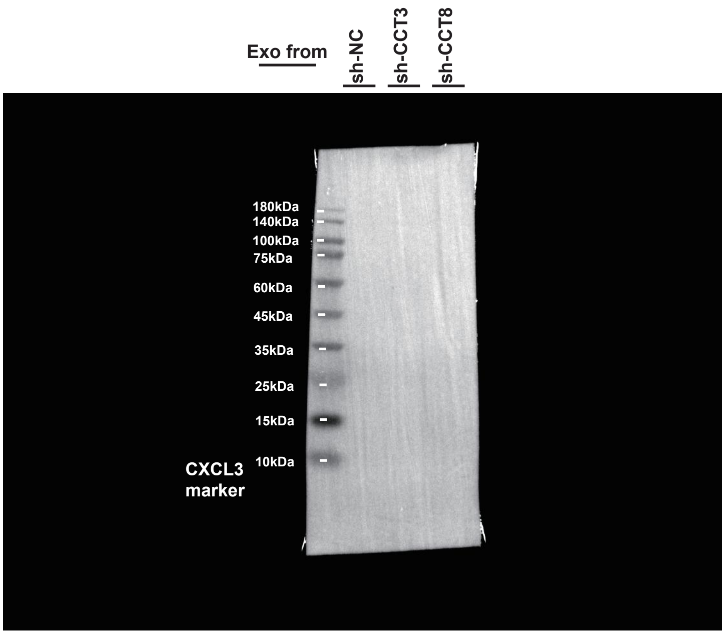


AsPC-1

**Fig. S10F**

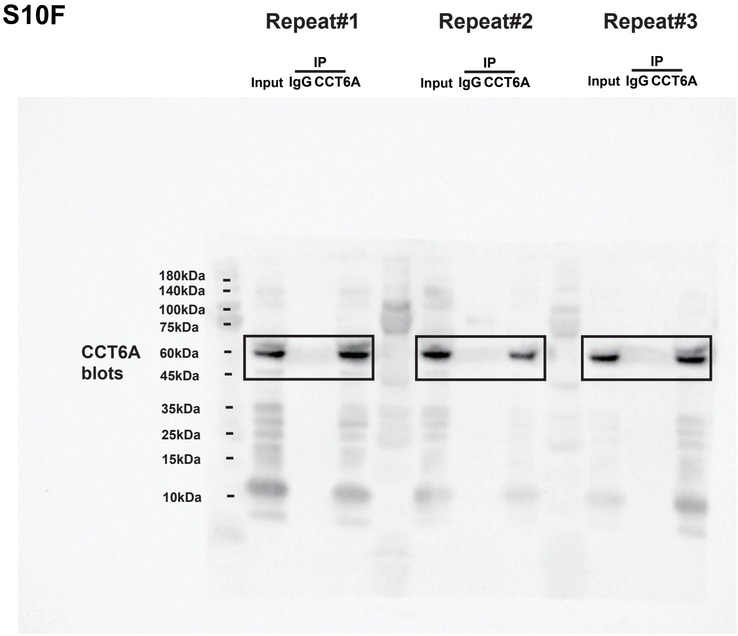


AsPC-1-Exo

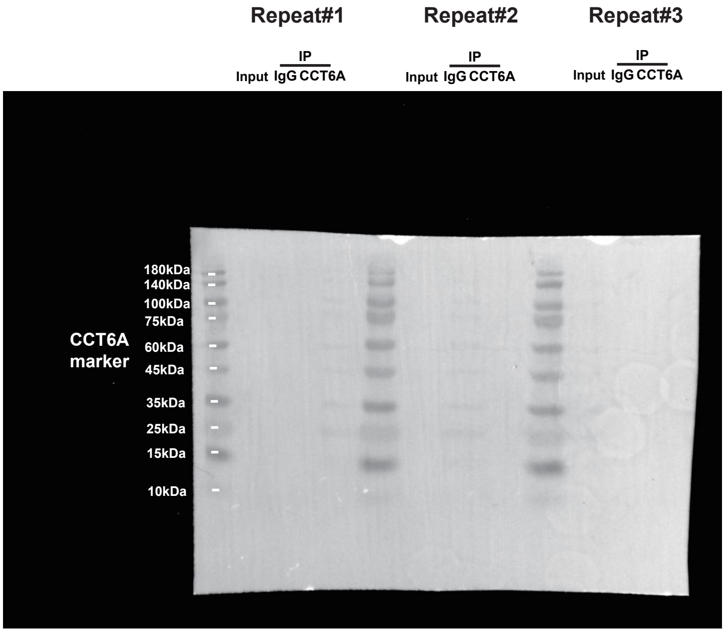


AsPC-1-Exo

**Repeat#1**

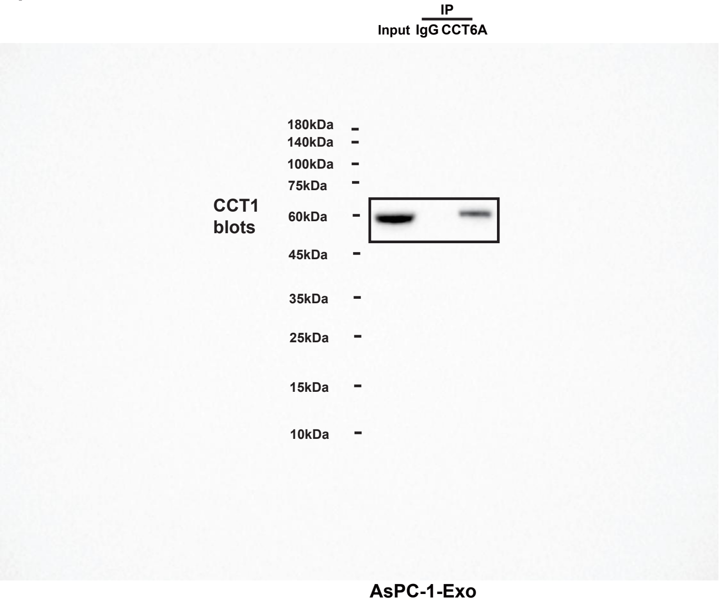


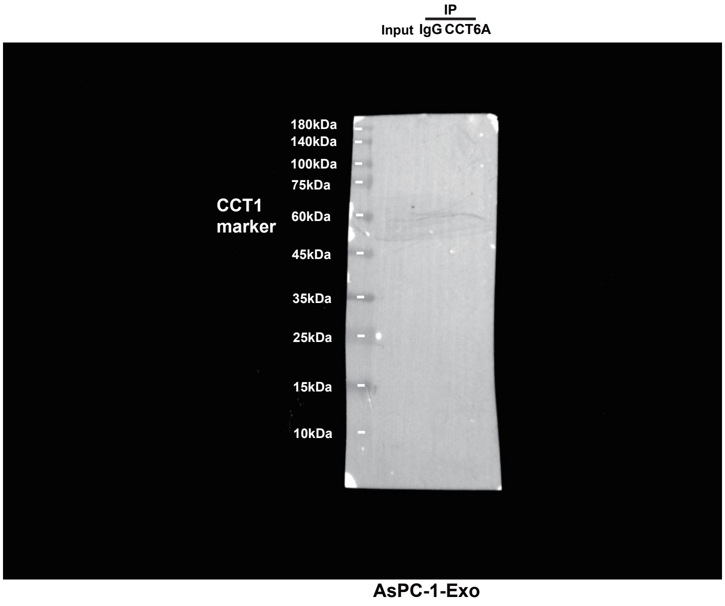


**Repeat#2**

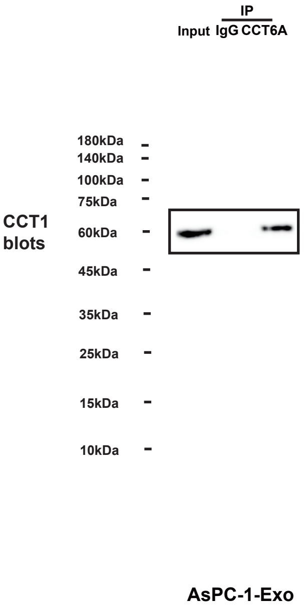


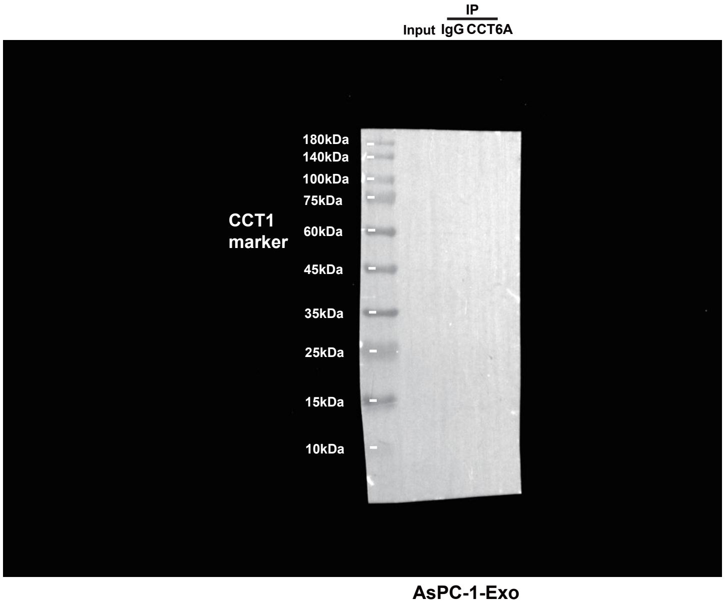


**Repeat#3**

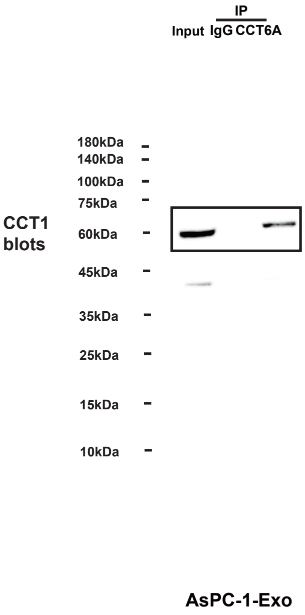


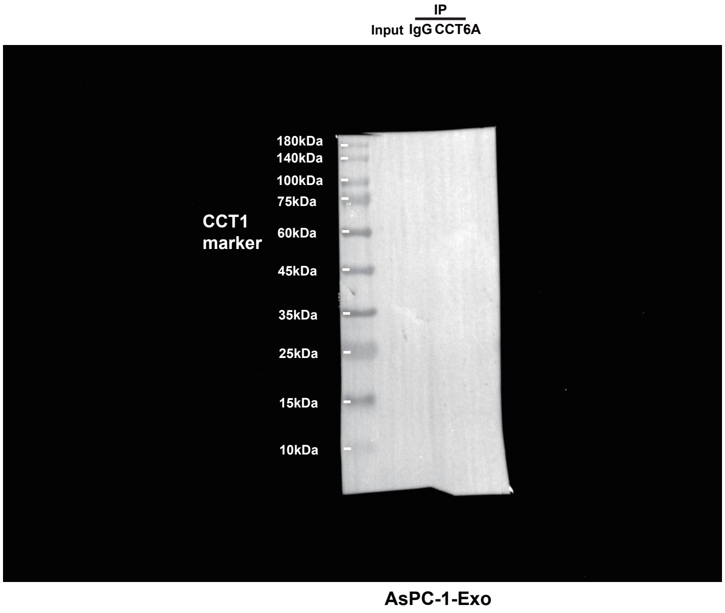

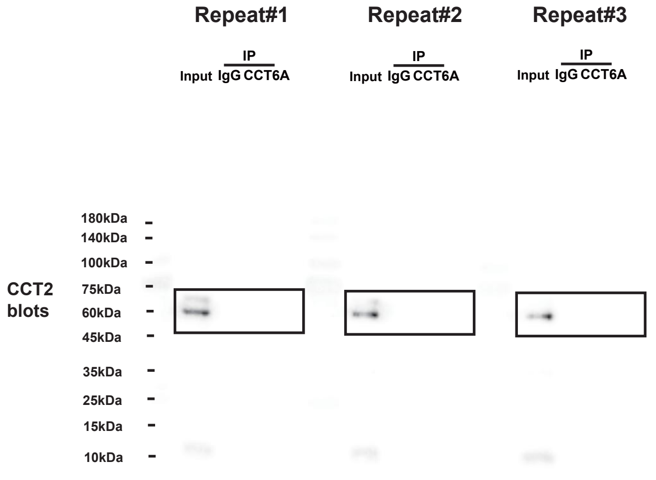


AsPC-1-Exo

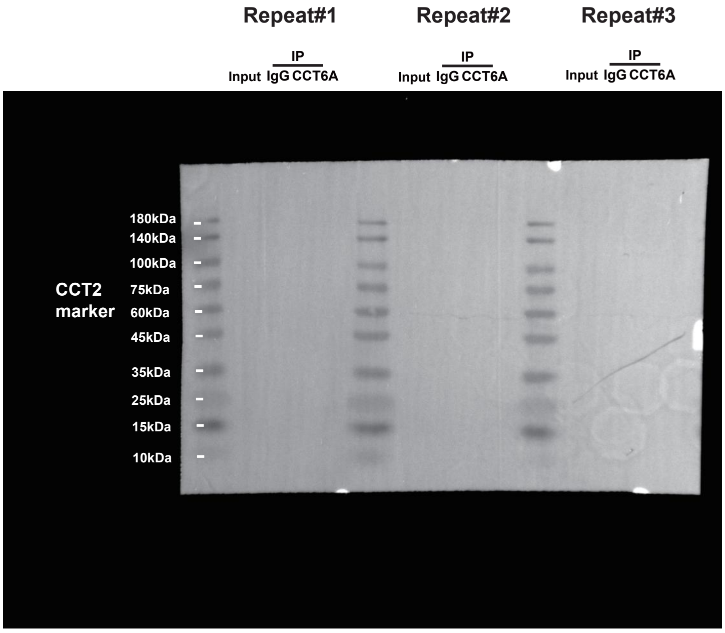


AsPC-1-Exo

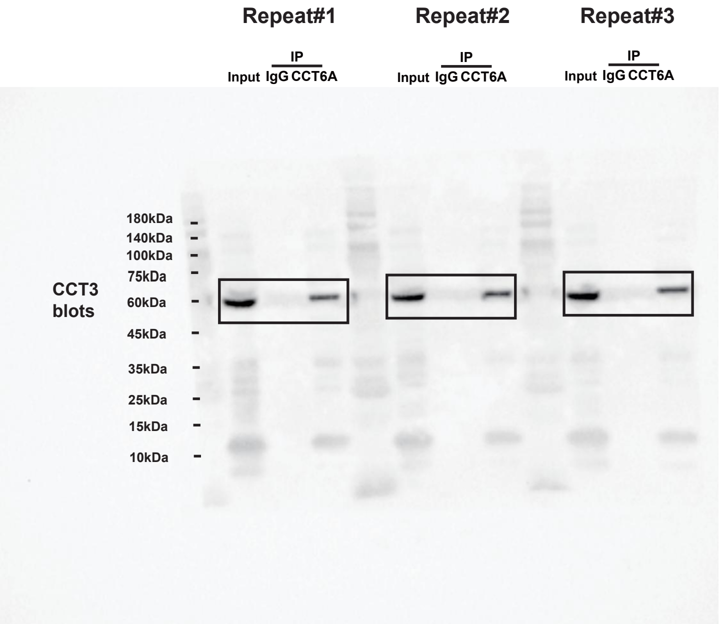


AsPC-1-Exo

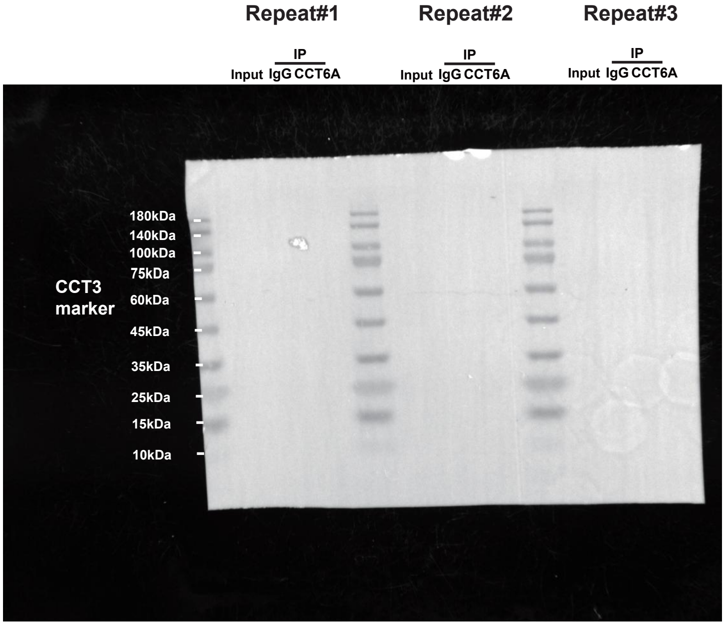


AsPC-1-Exo

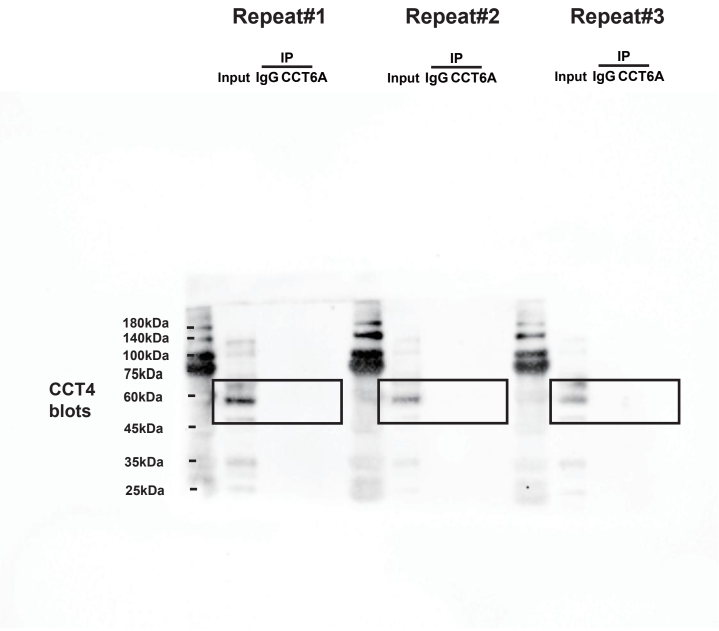


AsPC-1-Exo

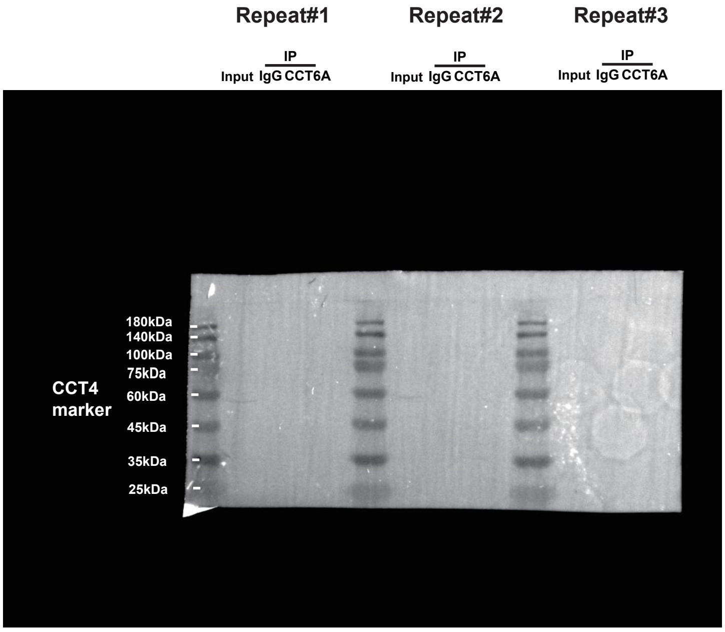


AsPC-1-Exo

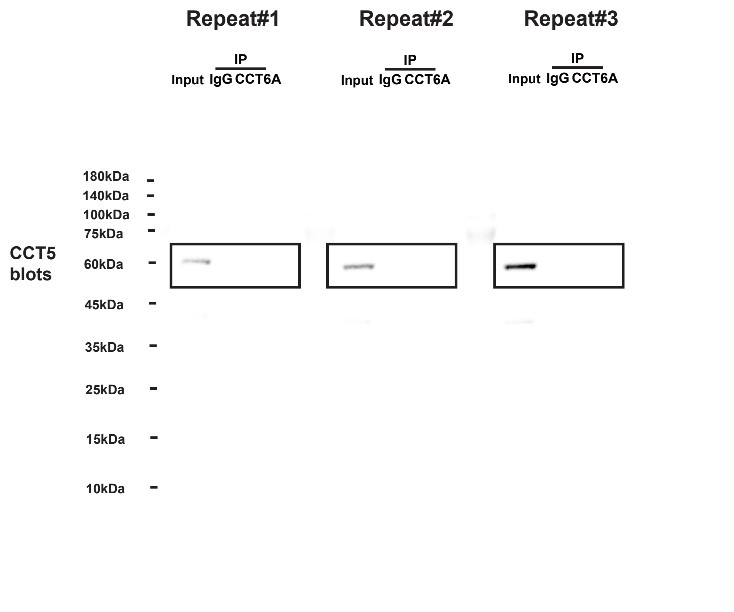


AsPC-1-Exo

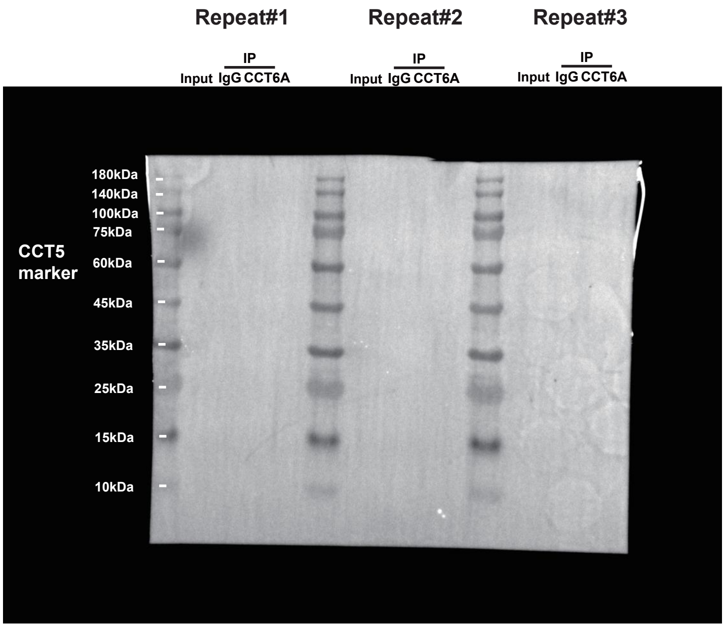


AsPC-1-Exo

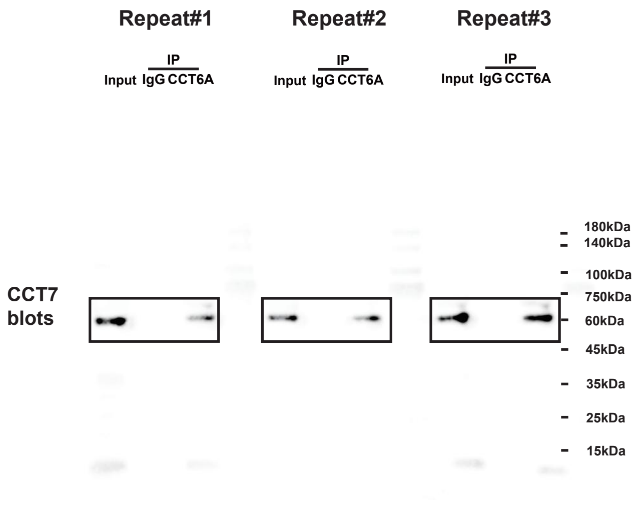


AsPC-1-Exo

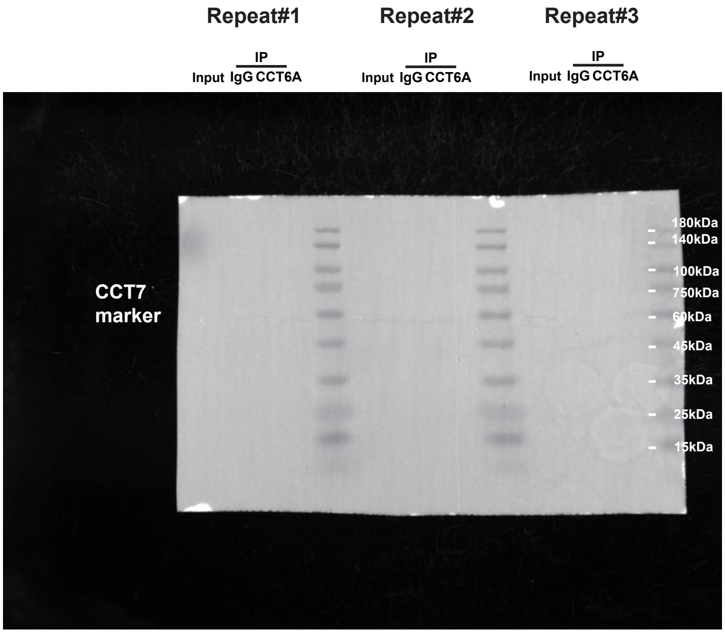


AsPC-1-Exo

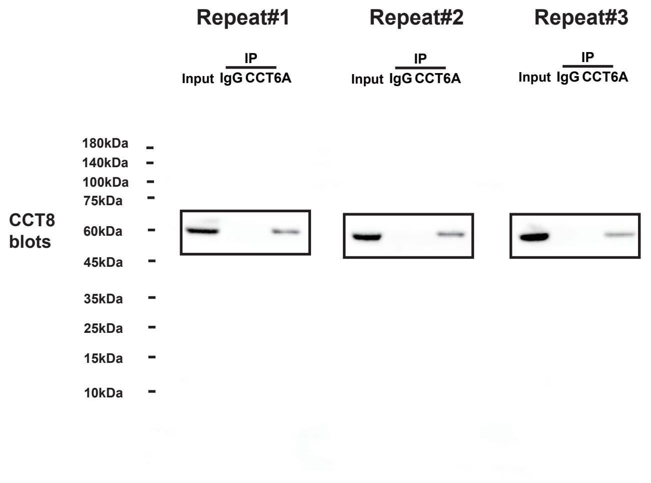


AsPC-1-Exo

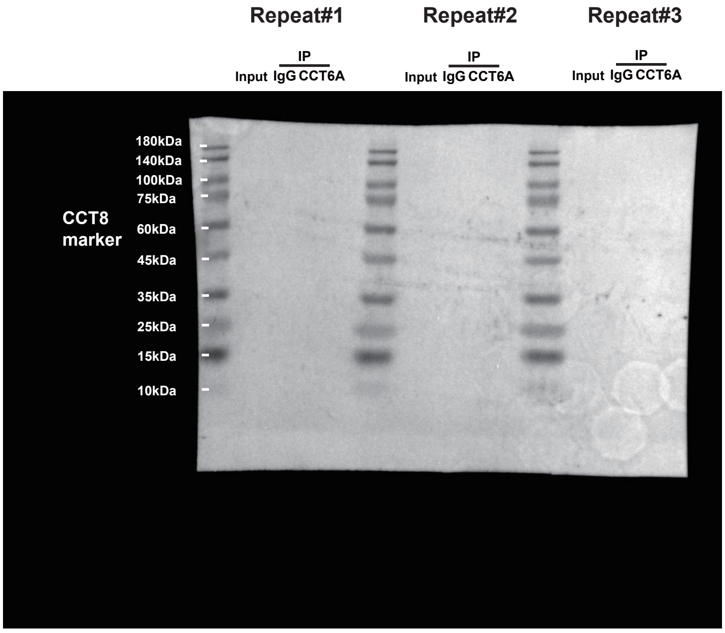


AsPC-1-Exo

**Fig. S10G**

Repeat#1

BxPC-3-Exo

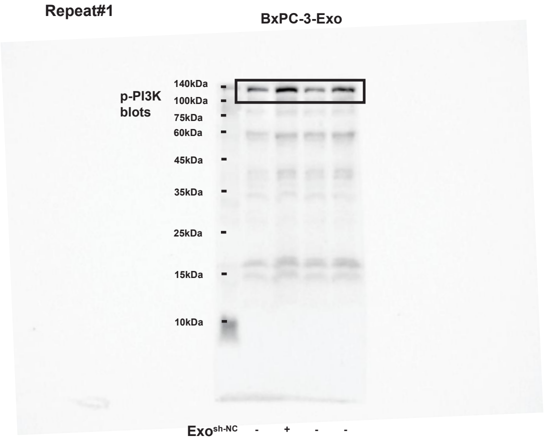


| | | | | |
|---|---|---|---|---|
| $Exo^{sh-NC}$ | - | + | - | - |
| $Exo^{sh-CCT6A}$ | - | - | + | - |
| $Exo^{sh-CCT6A+OE-CCL5}$ | - | - | - | + |

BxPC-3-Exo

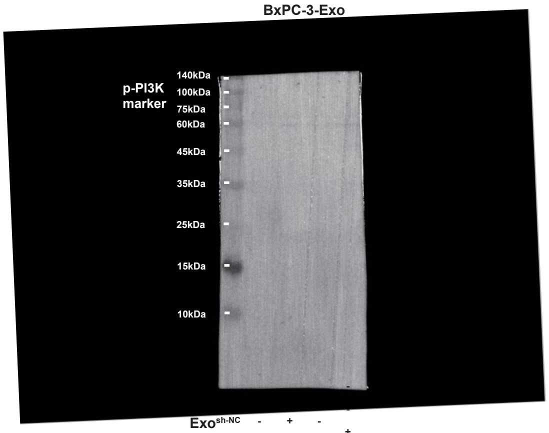


| | | | | |
|---|---|---|---|---|
| $Exo^{sh-NC}$ | - | + | - | + |
| $Exo^{sh-CCT6A}$ | - | - | + | |
| $Exo^{sh-CCT6A+OE-CCL5}$ | - | - | - | |

Repeat#2

BxPC-3-Exo

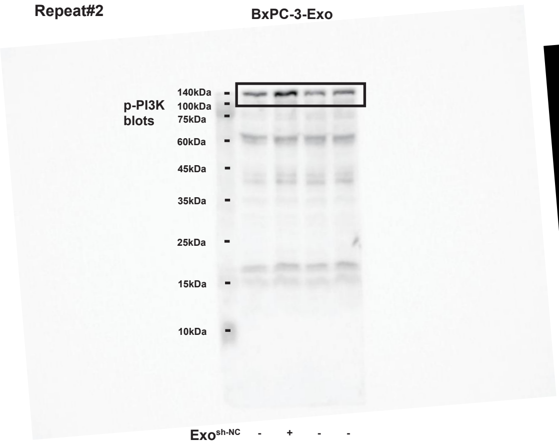


| | | | | |
|---|---|---|---|---|
| $Exo^{sh-NC}$ | - | + | - | - |
| $Exo^{sh-CCT6A}$ | - | - | + | - |
| $Exo^{sh-CCT6A+OE-CCL5}$ | - | - | - | + |

BxPC-3-Exo

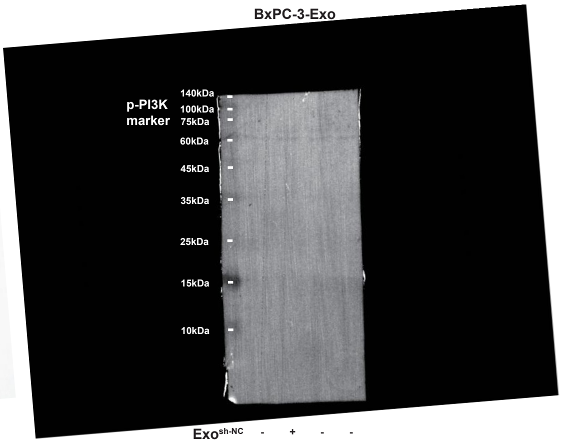


| | | | | |
|---|---|---|---|---|
| $Exo^{sh-NC}$ | - | + | - | - |
| $Exo^{sh-CCT6A}$ | - | - | + | - |
| $Exo^{sh-CCT6A+OE-CCL5}$ | - | - | - | + |

Repeat#3

BxPC-3-Exo

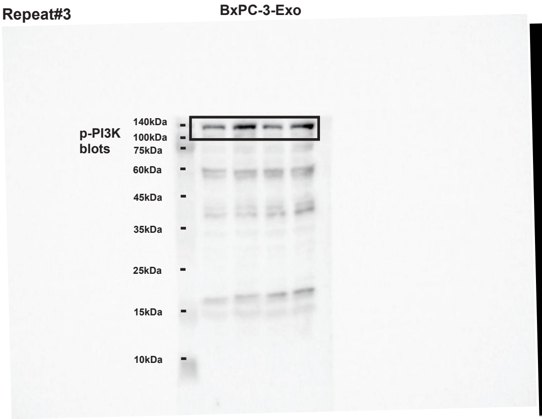


| | | | | |
|---|---|---|---|---|
| $Exo^{sh-NC}$ | - | + | - | - |
| $Exo^{sh-CCT6A}$ | - | - | + | - |
| $Exo^{sh-CCT6A+OE-CCL5}$ | - | - | - | + |

BxPC-3-Exo

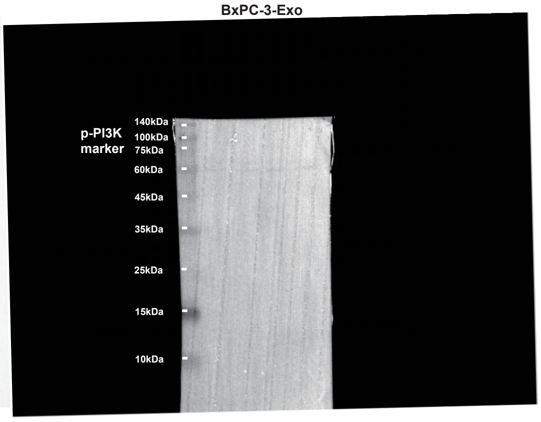


| | | | | |
|---|---|---|---|---|
| $Exo^{sh-NC}$ | - | + | - | - |
| $Exo^{sh-CCT6A}$ | - | - | + | - |
| $Exo^{sh-CCT6A+OE-CCL5}$ | - | - | - | + |

| | p-PI3K | PI3K |
|---|---|---|
| Control | 10,826.04 | 29,690.84 |
| sh-nc | 18,832.82 | 26,690.58 |
| sh-cct6a | 8,621.31 | 29,418.43 |
| oe-ccl5 | 18,912.53 | 27,593.54 |
| | | |
| Control | 3486.098 | 10,987.95 |
| sh-nc | 6506.0172 | 10,188.08 |
| sh-cct6a | 2632.6838 | 11,363.37 |
| oe-ccl5 | 5131.3824 | 10,021.73 |
| | | |
| Control | 6,509.32 | 20,854.39 |
| sh-nc | 12,477.44 | 20,011.24 |
| sh-cct6a | 5,968.34 | 20,711.54 |
| oe-ccl5 | 12,412.65 | 20,753.08 |

Repeat#1

BxPC-3-Exo

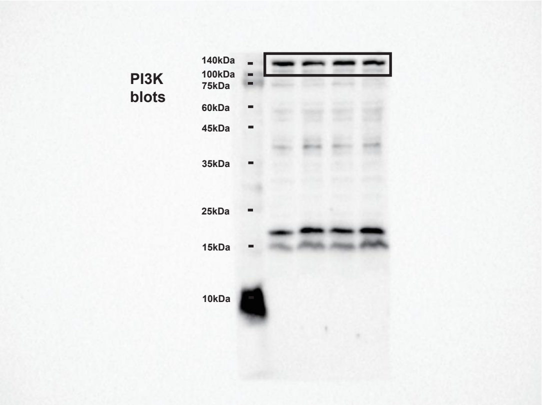


| | | | | |
|---|---|---|---|---|
| $Exo^{sh-NC}$ | - | + | - | - |
| $Exo^{sh-CCT6A}$ | - | - | + | - |
| $Exo^{sh-CCT6A+OE-CCL5}$ | - | - | - | + |

BxPC-3-Exo

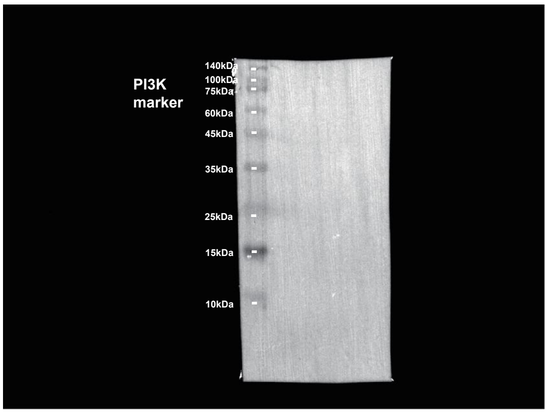


| | | | | |
|---|---|---|---|---|
| $Exo^{sh-NC}$ | - | + | - | - |
| $Exo^{sh-CCT6A}$ | - | - | + | - |
| $Exo^{sh-CCT6A+OE-CCL5}$ | - | - | - | + |

Repeat#2

BxPC-3-Exo

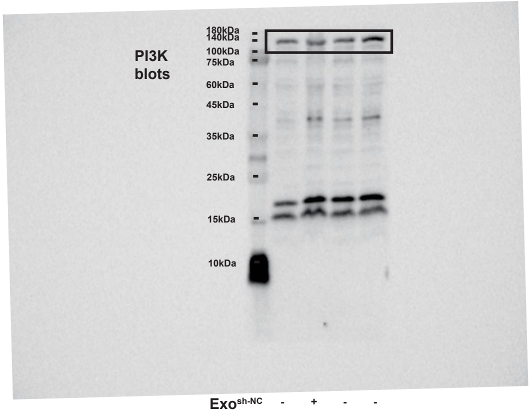


| | | | | |
|---|---|---|---|---|
| $Exo^{sh-NC}$ | - | + | - | - |
| $Exo^{sh-CCT6A}$ | - | - | + | - |
| $Exo^{sh-CCT6A+OE-CCL5}$ | - | - | - | + |

BxPC-3-Exo

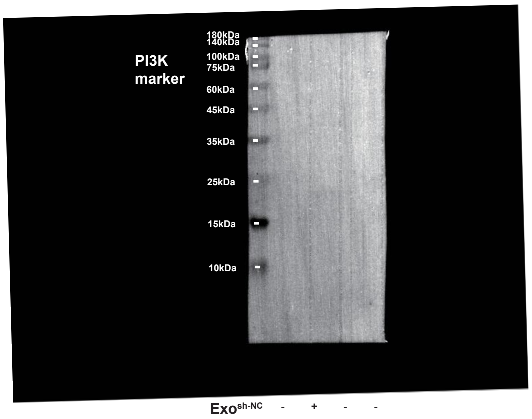


| | | | | |
|---|---|---|---|---|
| $Exo^{sh-NC}$ | - | + | - | - |
| $Exo^{sh-CCT6A}$ | - | - | + | - |
| $Exo^{sh-CCT6A+OE-CCL5}$ | - | - | - | + |

Repeat#3

BxPC-3-Exo

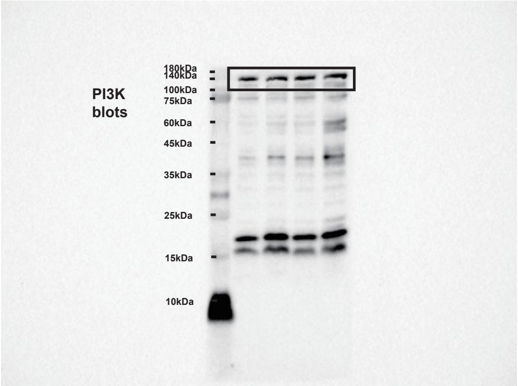


| | | | | |
|---|---|---|---|---|
| $Exo^{sh-NC}$ | - | + | - | - |
| $Exo^{sh-CCT6A}$ | - | - | + | - |
| $Exo^{sh-CCT6A+OE-CCL5}$ | - | - | - | + |

BxPC-3-Exo

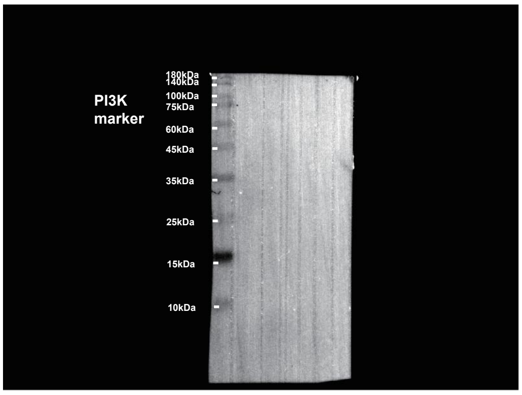


| | | | | |
|---|---|---|---|---|
| $Exo^{sh-NC}$ | - | + | - | - |
| $Exo^{sh-CCT6A}$ | - | - | + | - |
| $Exo^{sh-CCT6A+OE-CCL5}$ | - | - | - | + |

| | p-PI3K | PI3K |
|---|---|---|
| Control | 10,826.04 | 29,690.84 |
| sh-nc | 18,832.82 | 26,690.58 |
| sh-cct6a | 8,621.31 | 29,418.43 |
| oe-ccl5 | 18,912.53 | 27,593.54 |
| | | |
| Control | 3486.098 | 10,987.95 |
| sh-nc | 6506.0172 | 10,188.08 |
| sh-cct6a | 2632.6838 | 11,363.37 |
| oe-ccl5 | 5131.3824 | 10,021.73 |
| | | |
| Control | 6,509.32 | 20,854.39 |
| sh-nc | 12,477.44 | 20,011.24 |
| sh-cct6a | 5,968.34 | 20,711.54 |
| oe-ccl5 | 12,412.65 | 20,753.08 |

Repeat#1

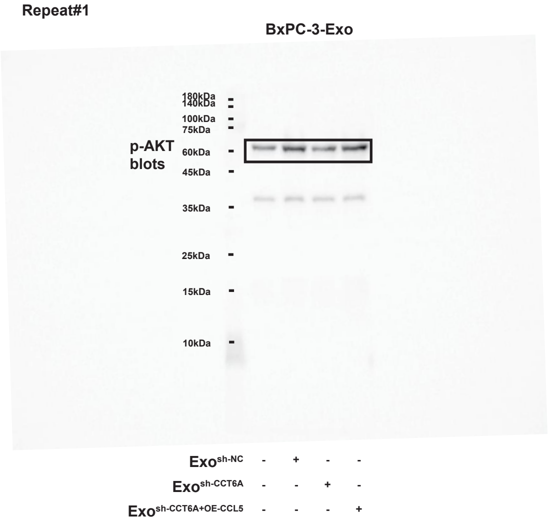


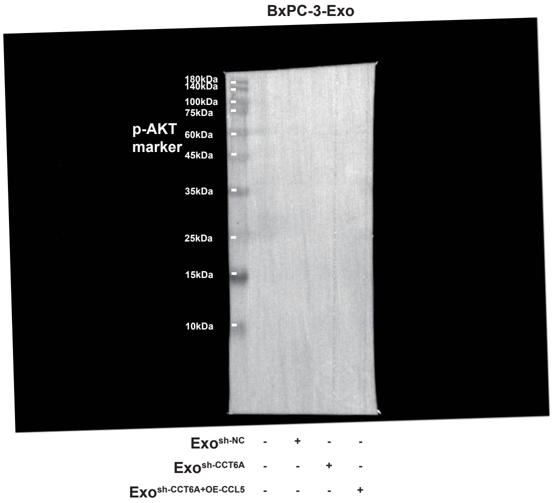


Repeat#2

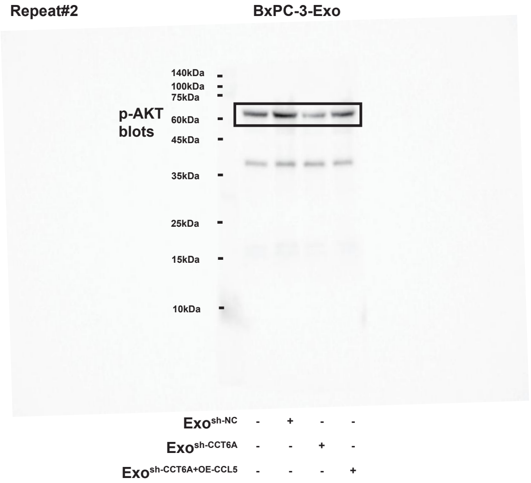


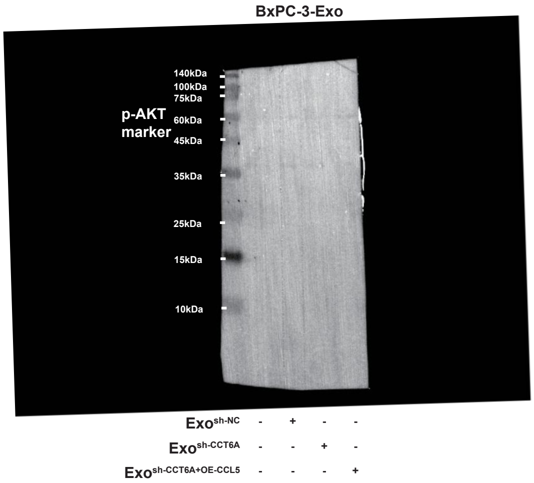


Repeat#3

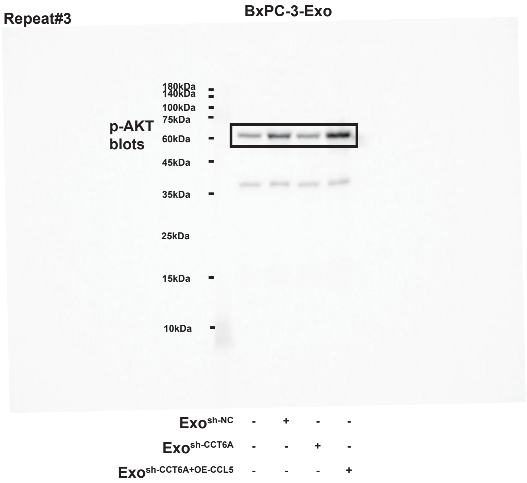


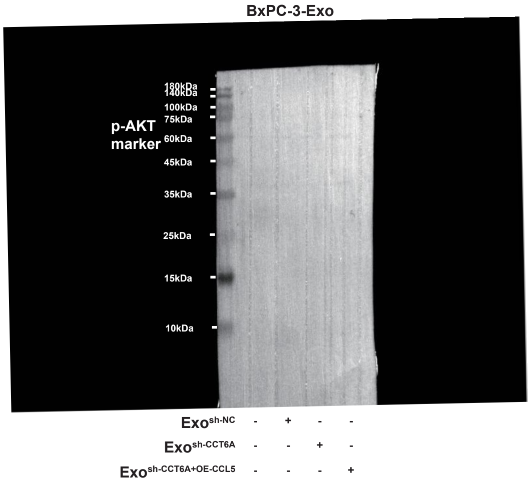


| | p-Akt | Akt |
|---|---|---|
| Control | 7,386.40 | 15,825.24 |
| sh-nc | 13,934.54 | 15,487.43 |
| sh-cct6a | 8,676.65 | 20,551.97 |
| oe-ccl5 | 17,961.87 | 19,573.81 |
| | | |
| Control | 8,371.30 | 20,263.37 |
| sh-cct6a | 16,600.68 | 17,120.57 |
| sh-cct6a | 11,516.85 | 21,469.07 |
| oe-ccl5 | 21,028.89 | 18,858.18 |
| | | |
| Control | 11,672.93 | 17,246.90 |
| sh-nc | 17,713.96 | 16,514.82 |
| sh-cct6a | 10,938.94 | 17,256.69 |
| oe-ccl5 | 19,437.87 | 16,755.98 |

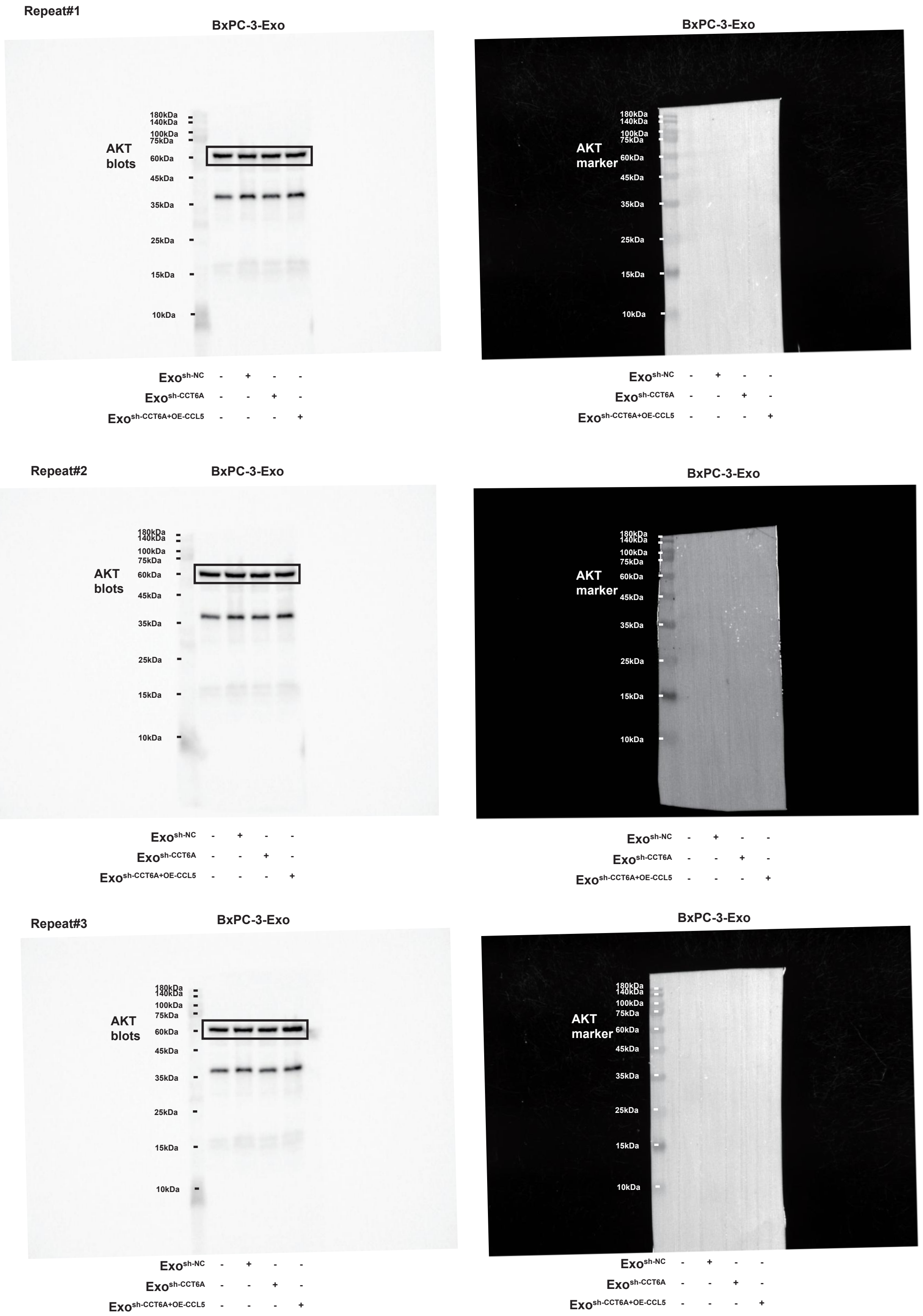


| | p-Akt | Akt |
|---|---|---|
| Control | 7,386.40 | 15,825.24 |
| sh-nc | 13,934.54 | 15,487.43 |
| sh-cct6a | 8,676.65 | 20,551.97 |
| oe-ccl5 | 17,961.87 | 19,573.81 |
| | | |
| Control | 8,371.30 | 20,263.37 |
| sh-cct6a | 16,600.68 | 17,120.57 |
| sh-cct6a | 11,516.85 | 21,469.07 |
| oe-ccl5 | 21,028.89 | 18,858.18 |
| | | |
| Control | 11,672.93 | 17,246.90 |
| sh-nc | 17,713.96 | 16,514.82 |
| sh-cct6a | 10,938.94 | 17,256.69 |
| oe-ccl5 | 19,437.87 | 16,755.98 |

Repeat#1

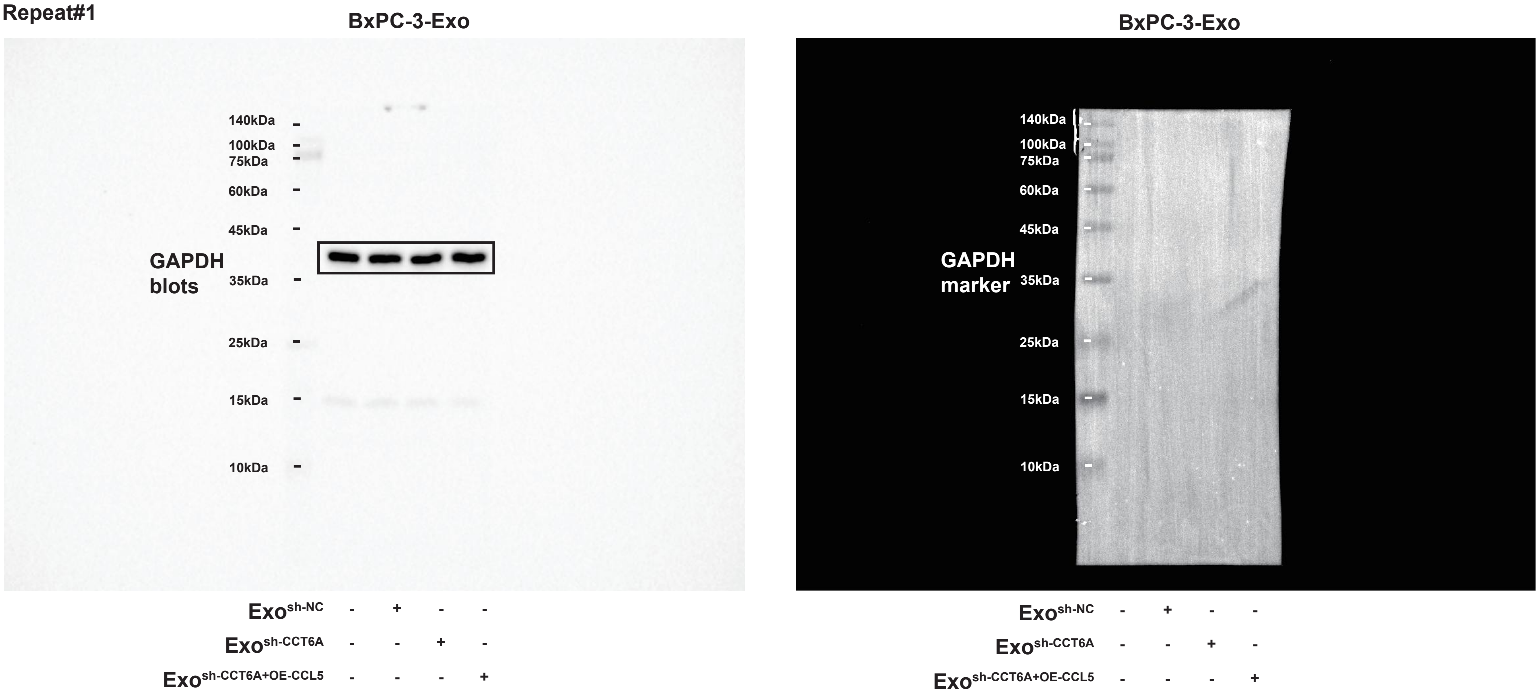


Repeat#2

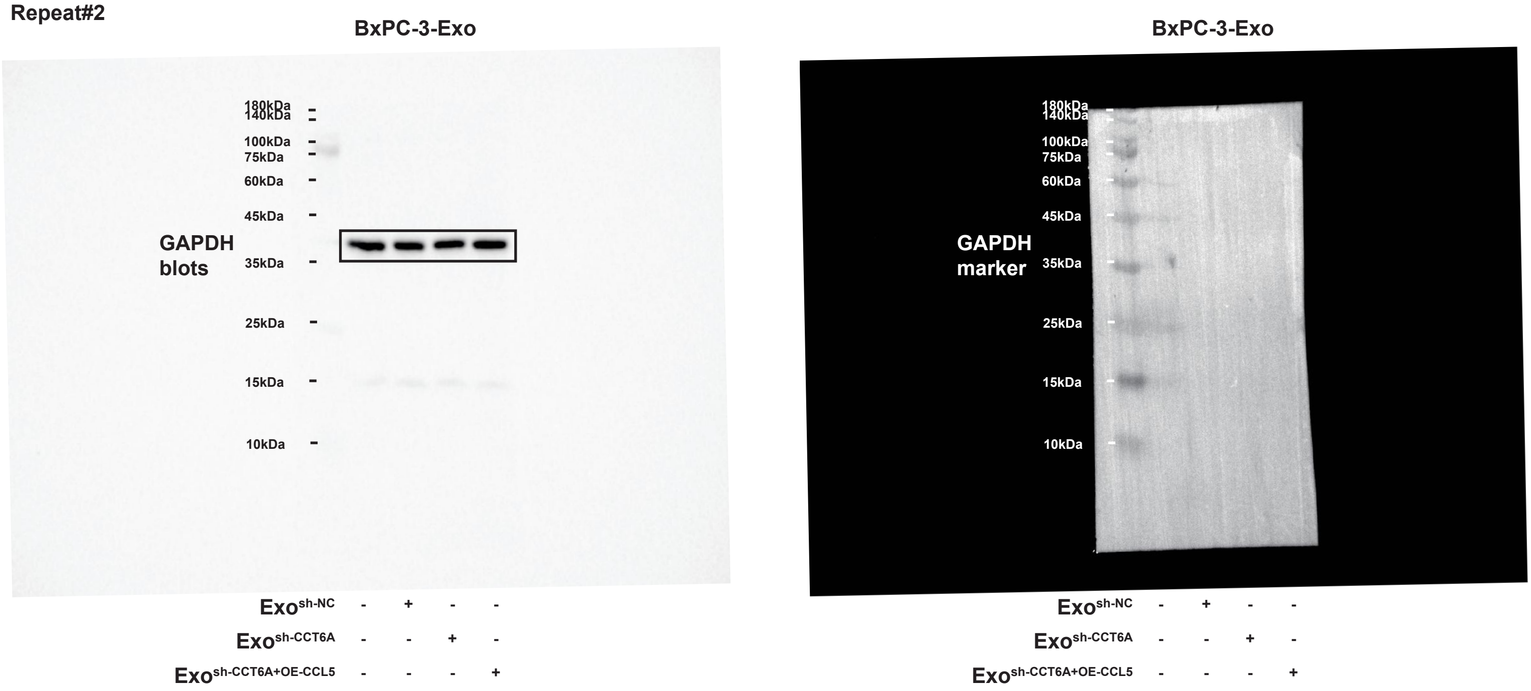


Repeat#3

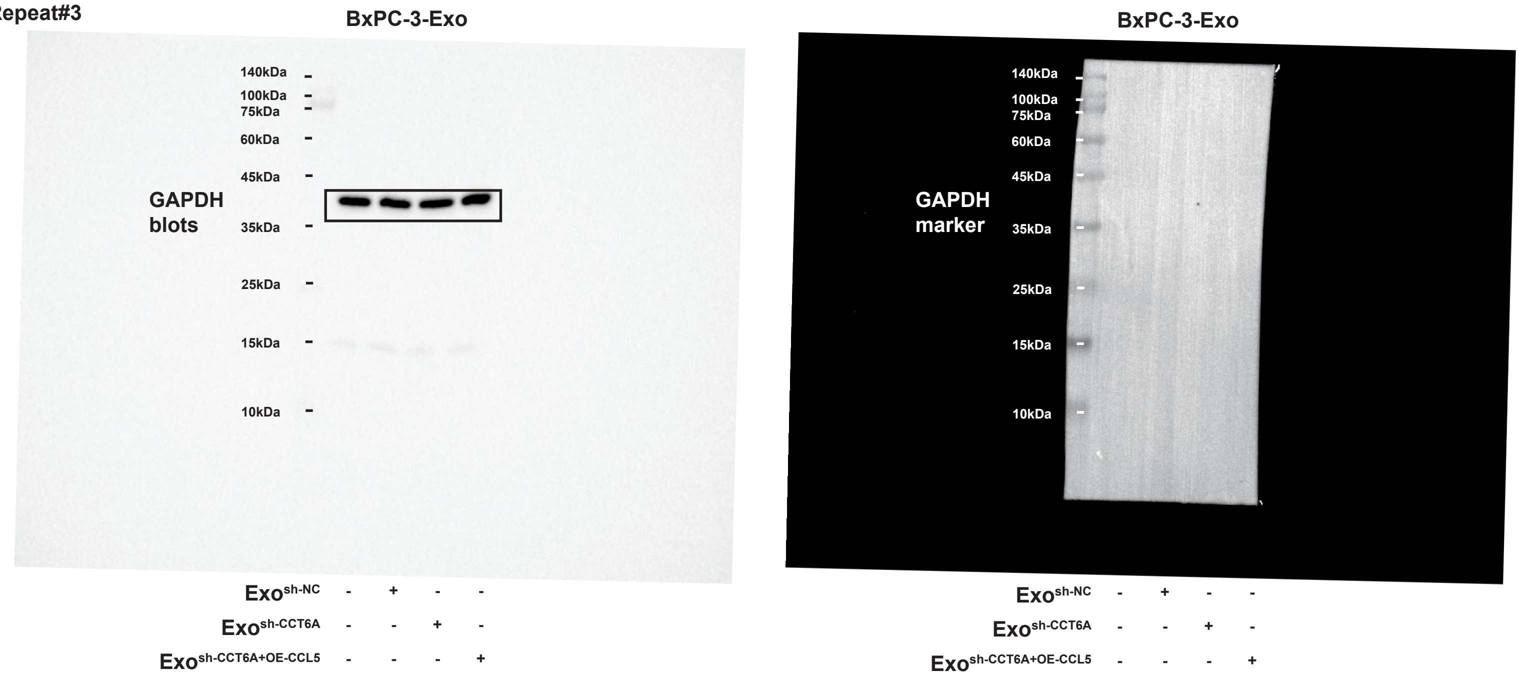

Fig. S11B

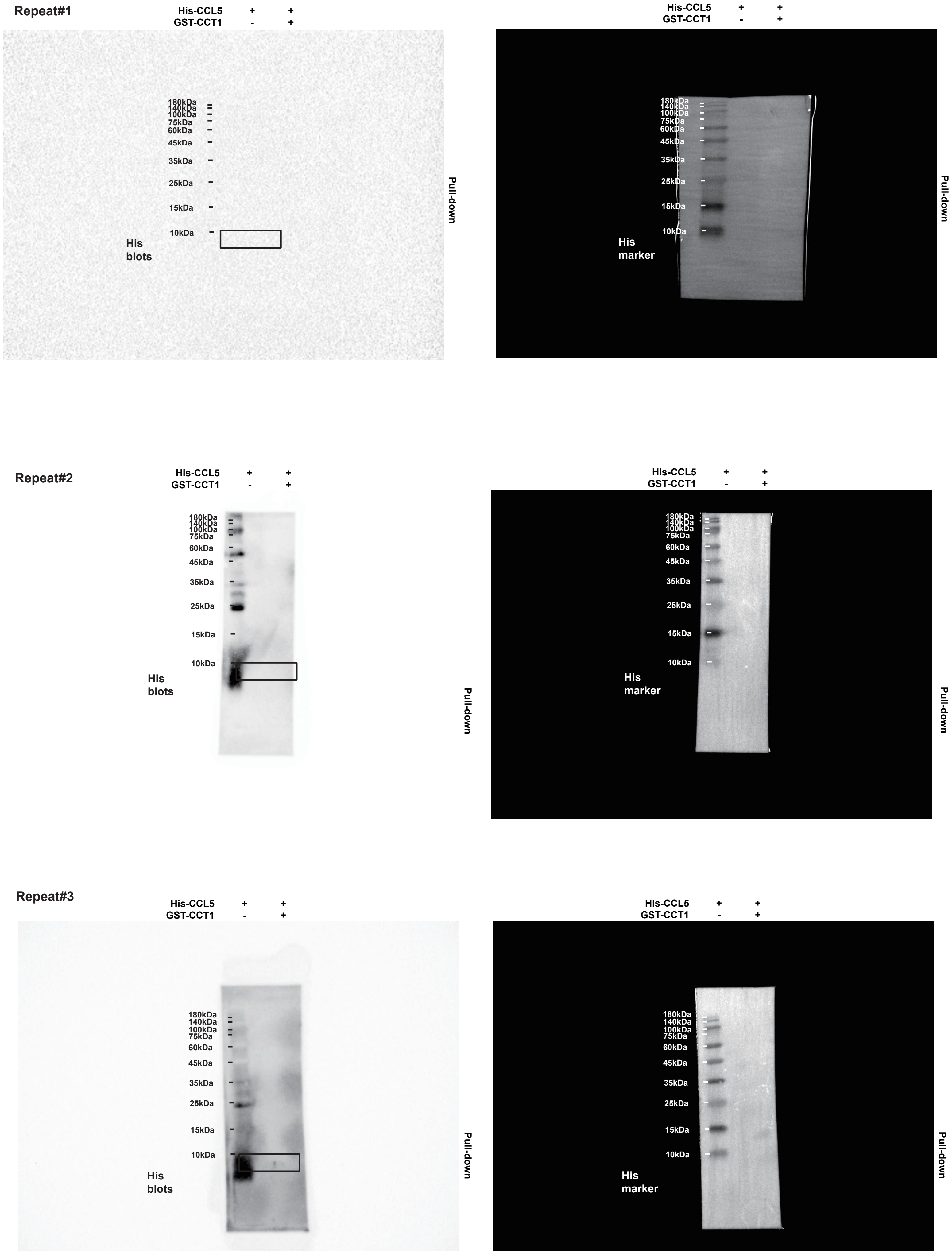
Repeat#1
His-CCL5 + +
GST-CCT1 - +
180kDa
140kDa
100kDa
75kDa
60kDa
45kDa
35kDa
25kDa
15kDa
10kDa
His blots
Pull-down
His-CCL5 + +
GST-CCT1 - +
180kDa
140kDa
100kDa
75kDa
60kDa
45kDa
35kDa
25kDa
15kDa
10kDa
His marker
Pull-down
Repeat#2
His-CCL5 + +
GST-CCT1 - +
180kDa
140kDa
100kDa
75kDa
60kDa
45kDa
35kDa
25kDa
15kDa
10kDa
His blots
Pull-down
His-CCL5 + +
GST-CCT1 - +
180kDa
140kDa
100kDa
75kDa
60kDa
45kDa
35kDa
25kDa
15kDa
10kDa
His marker
Pull-down
Repeat#3
His-CCL5 + +
GST-CCT1 - +
180kDa
140kDa
100kDa
75kDa
60kDa
45kDa
35kDa
25kDa
15kDa
10kDa
His blots
Pull-down
His-CCL5 + +
GST-CCT1 - +
180kDa
140kDa
100kDa
75kDa
60kDa
45kDa
35kDa
25kDa
15kDa
10kDa
His marker
Pull-down

Repeat#1

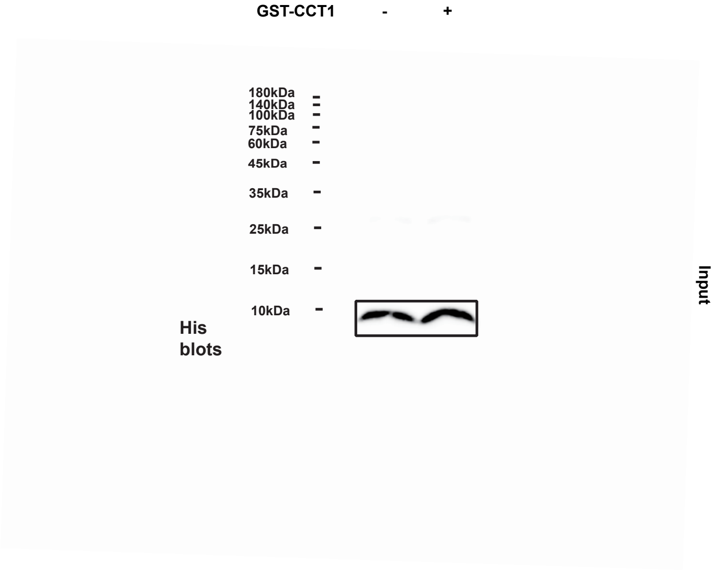


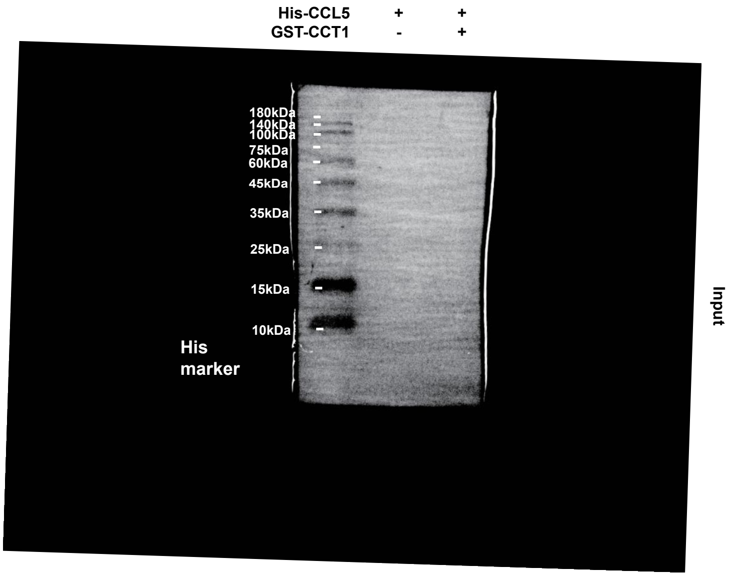


Repeat#2

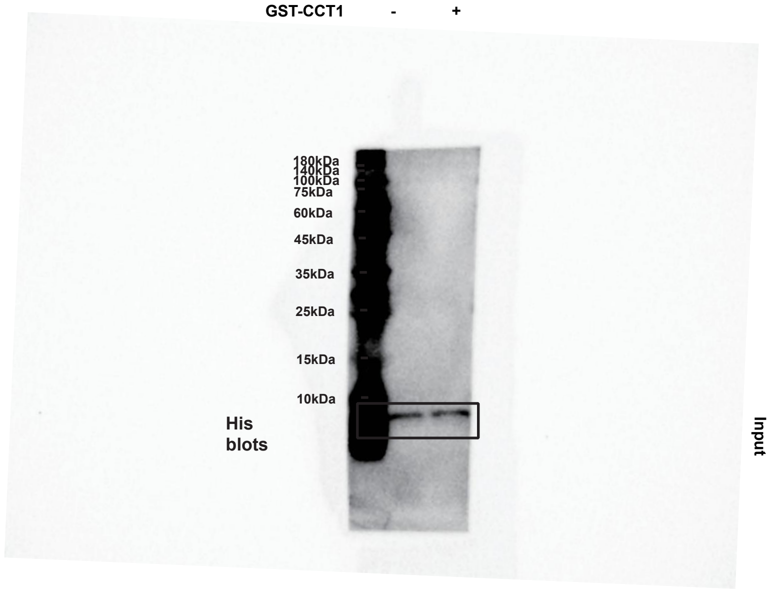


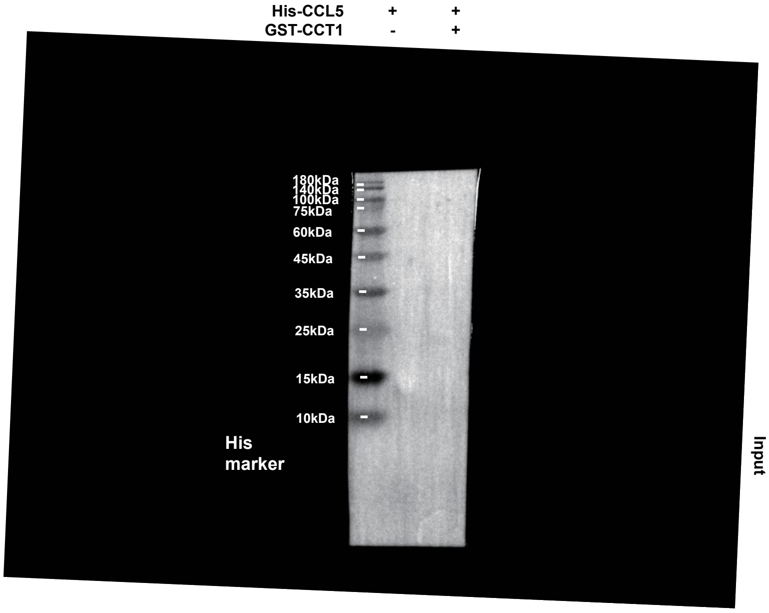


Repeat#3

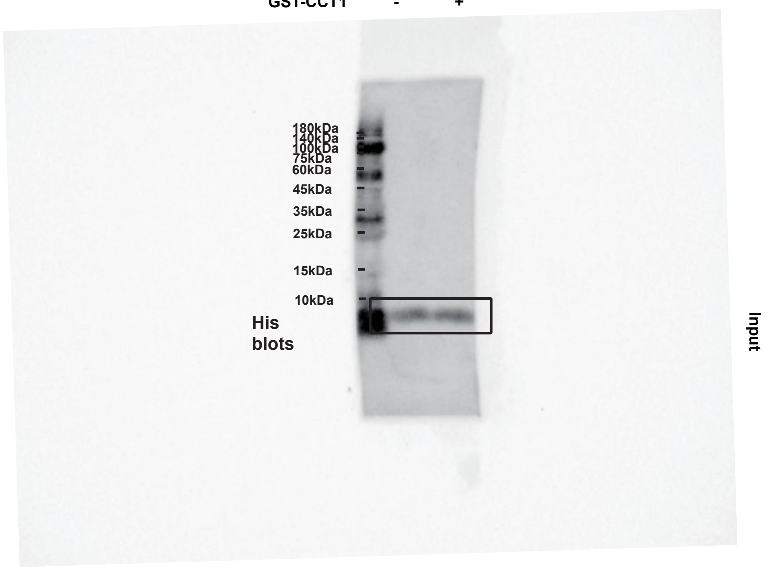


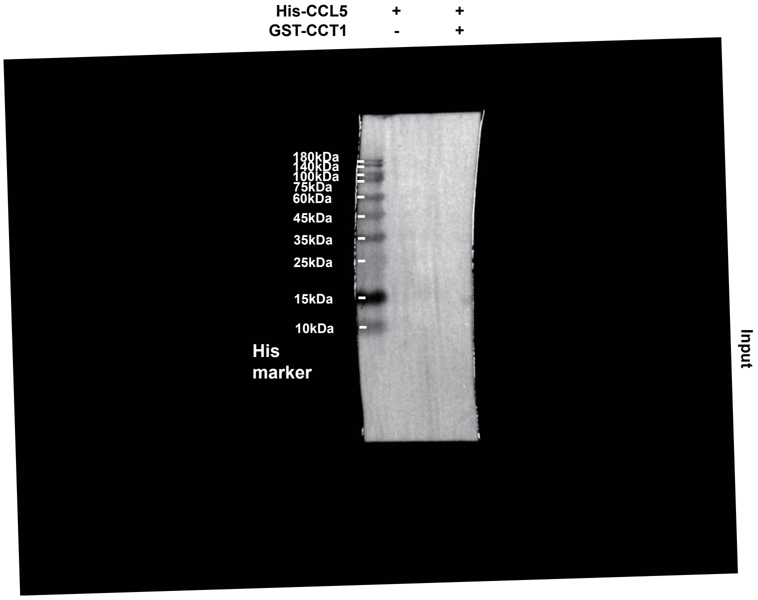

**Repeat#1**

His-CCL5 + +
GST-CCT8 - +

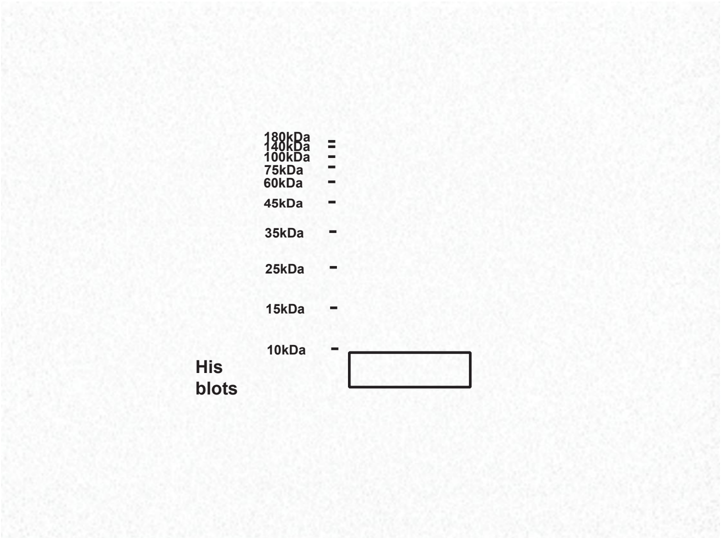


Pull-down

His-CCL5 + +
GST-CCT8 - +

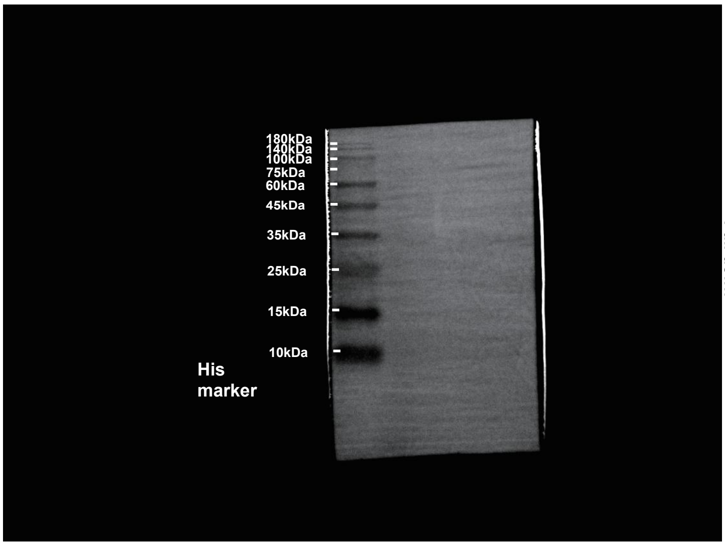


Pull-down

**Repeat#2**

His-CCL5 + +
GST-CCT8 - +

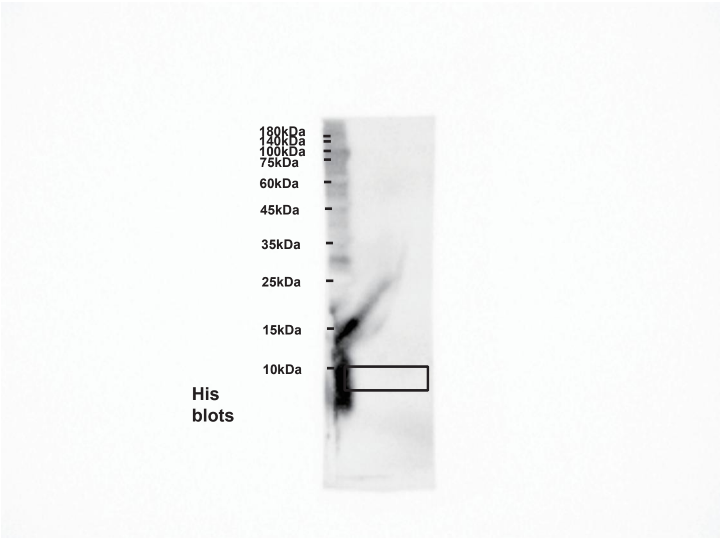


Pull-down

His-CCL5 + +
GST-CCT8 - +

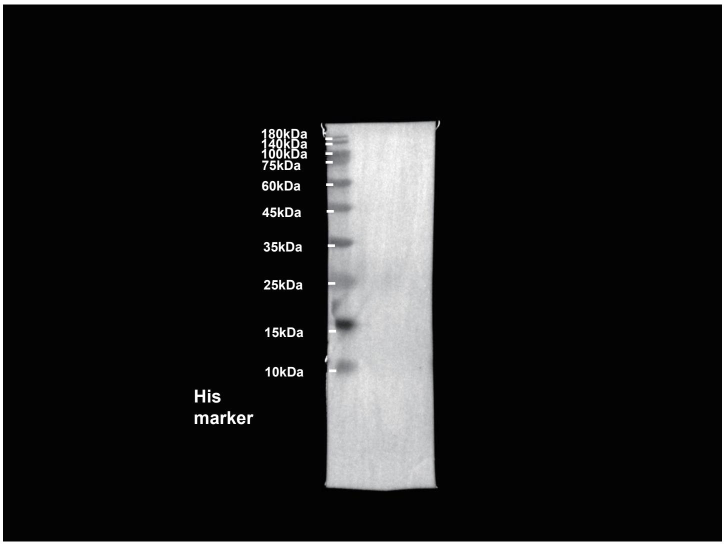


Pull-down

**Repeat#3**

His-CCL5 + +
GST-CCT8 - +

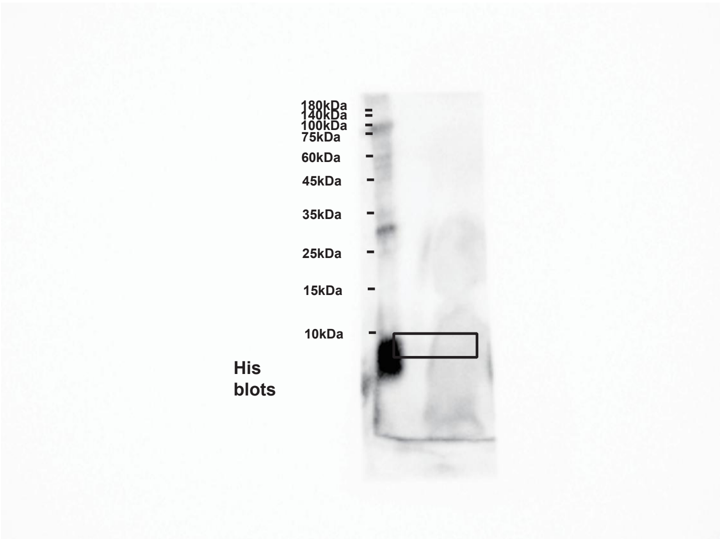


Pull-down

His-CCL5 + +
GST-CCT8 - +

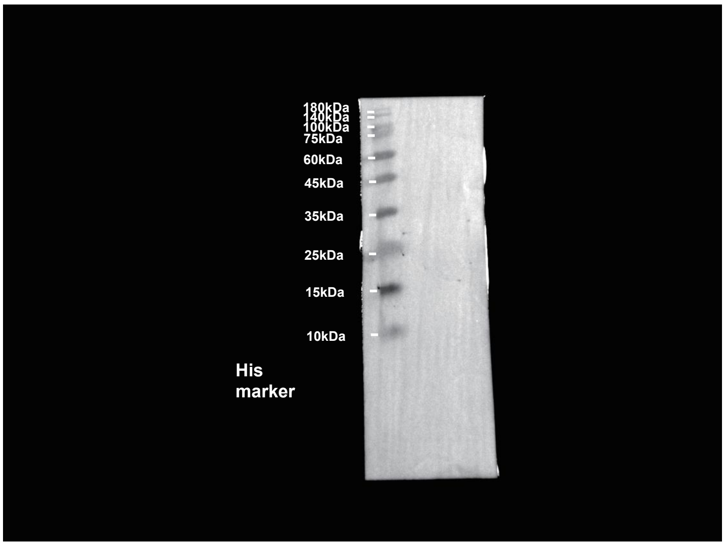


Pull-down

Repeat#1

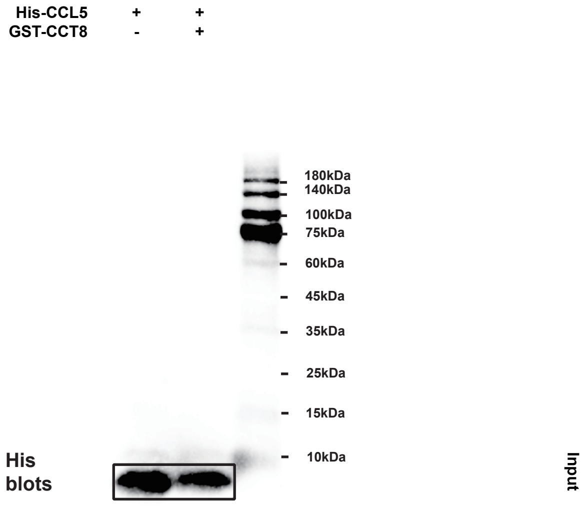


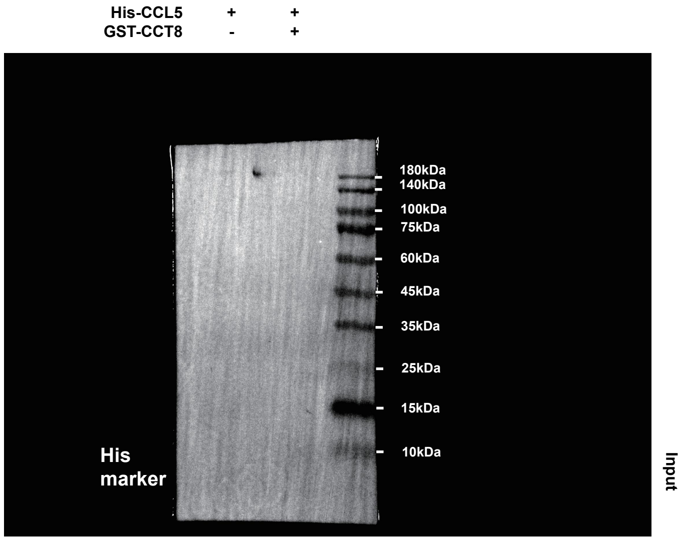


Repeat#2

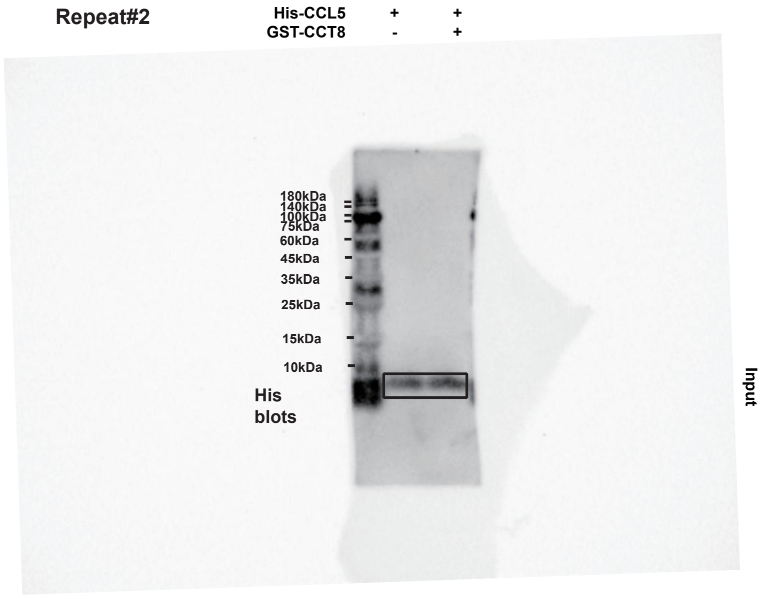


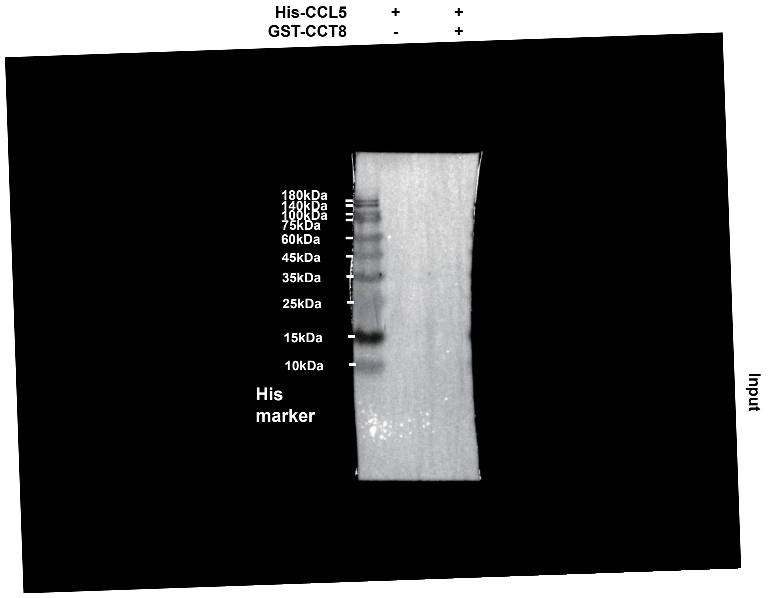


Repeat#3

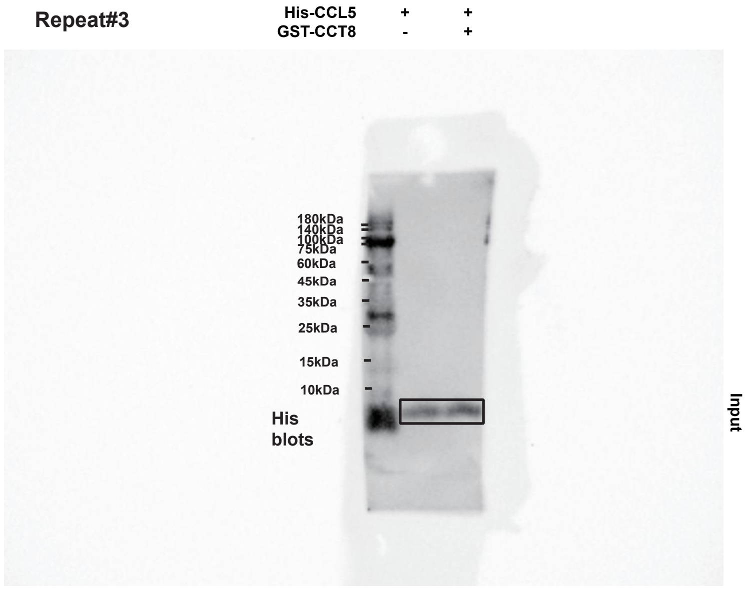


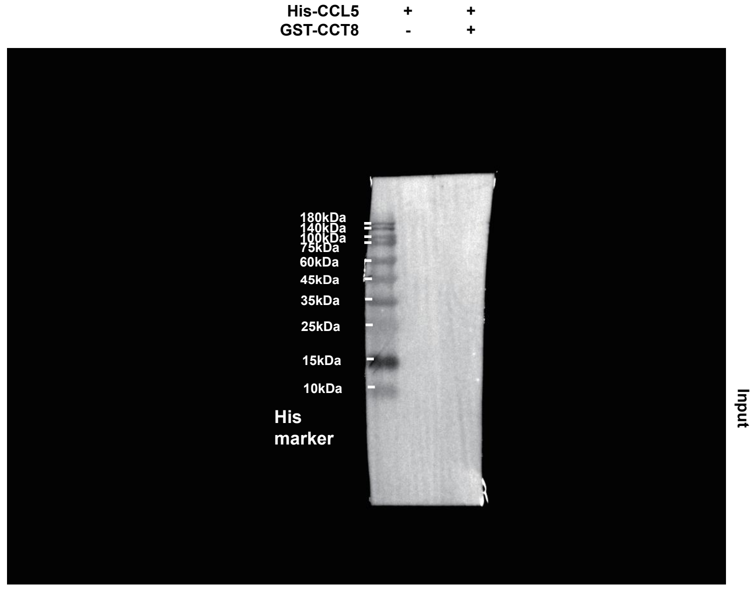

**Fig. S12A**

Repeat#1

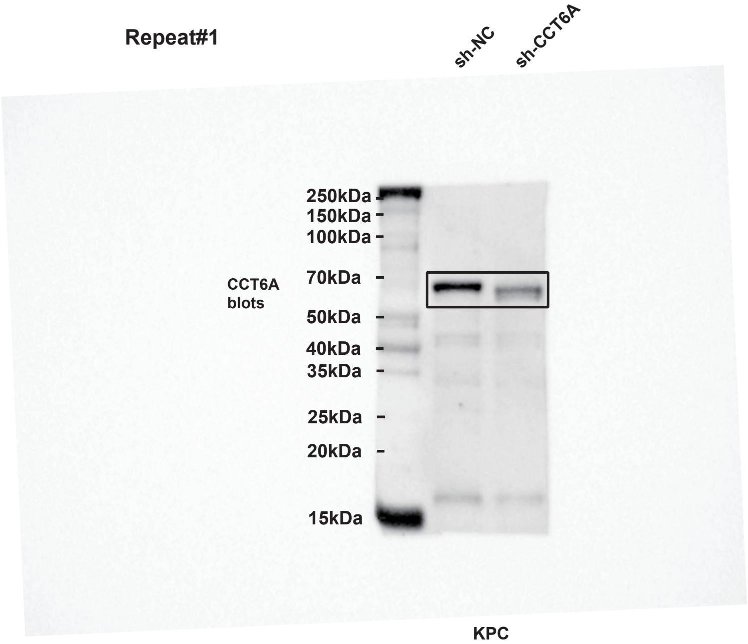


KPC

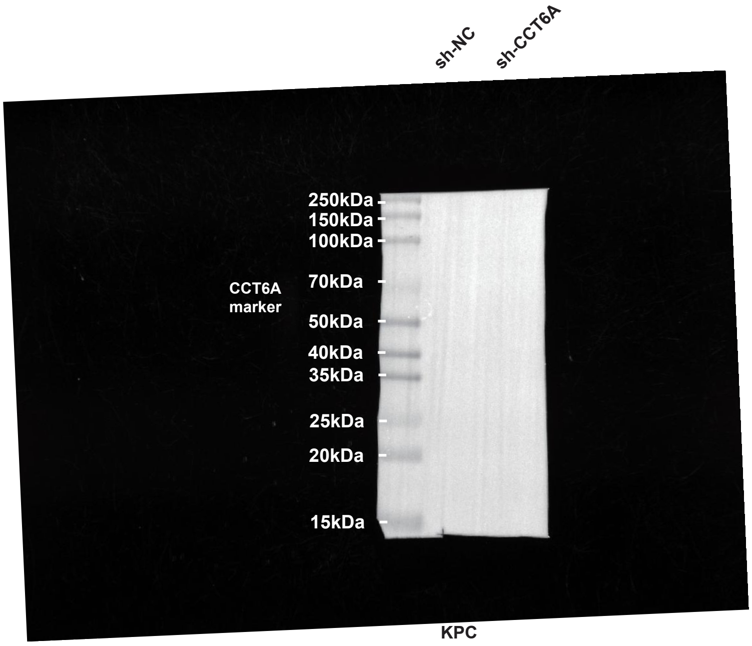


KPC

Repeat#2

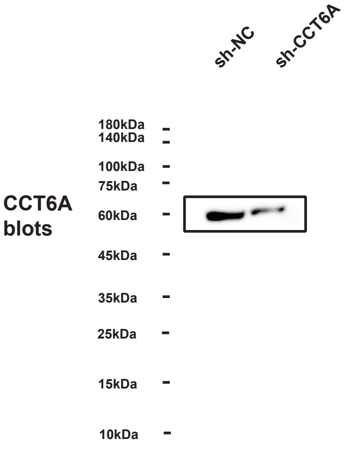


KPC

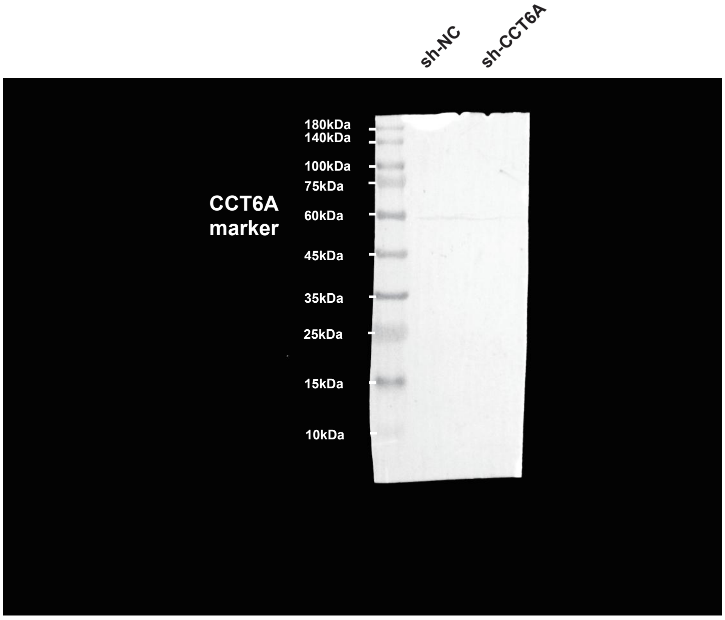


KPC

Repeat#3

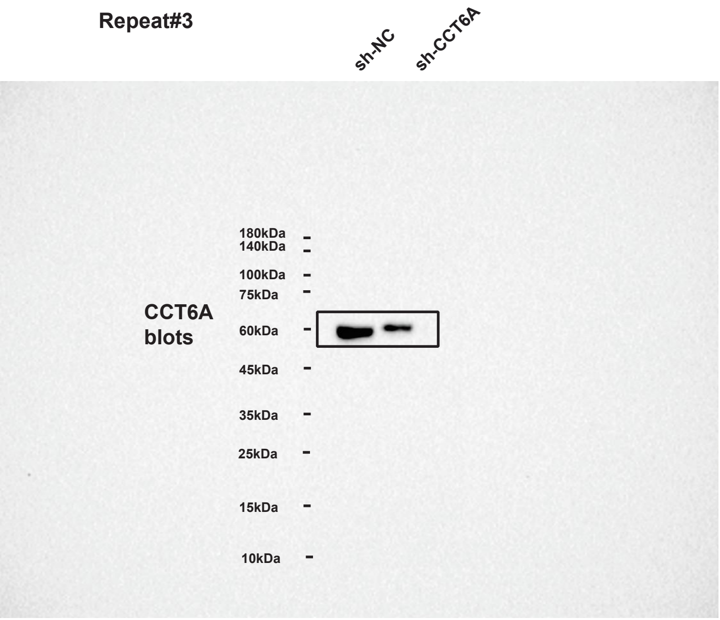


KPC

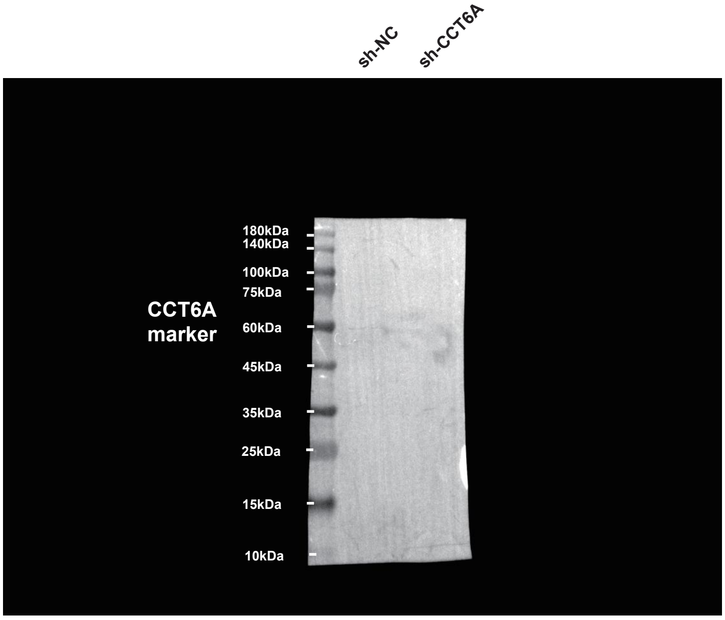


KPC

Repeat#1

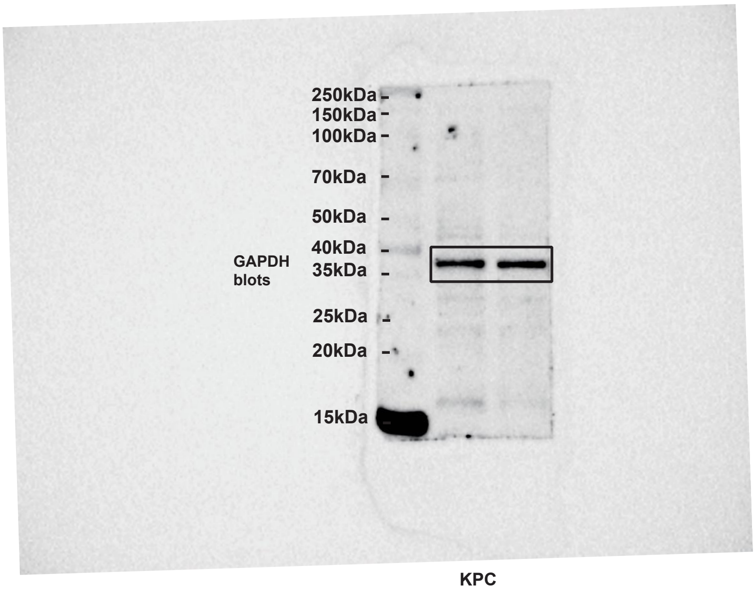


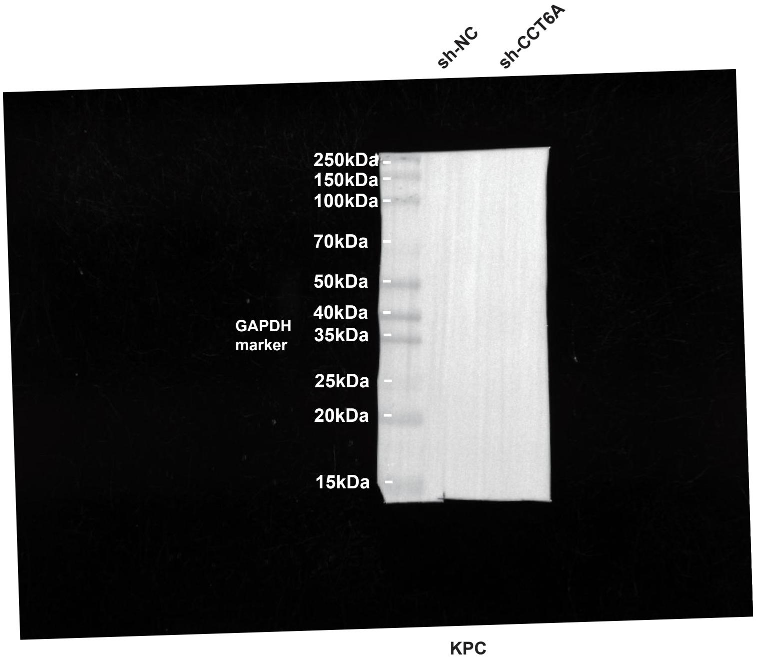


Repeat#2

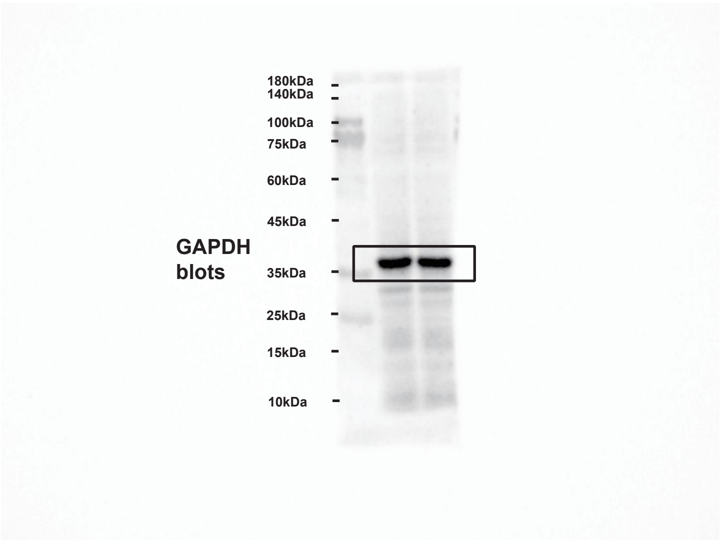


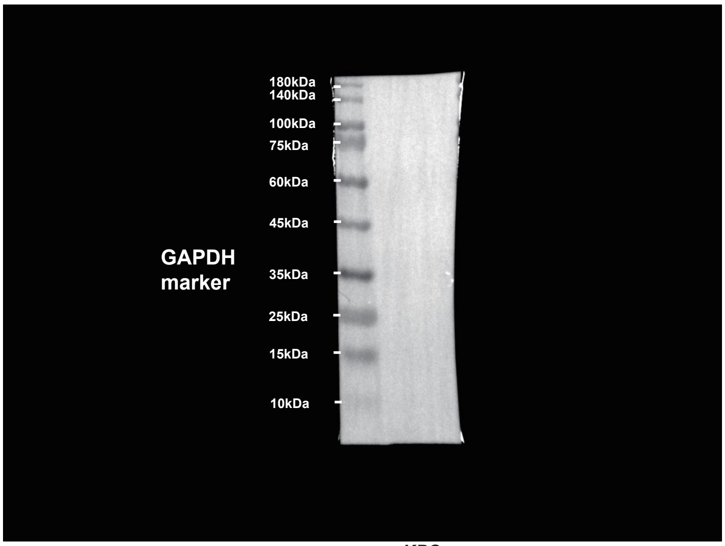


Repeat#3

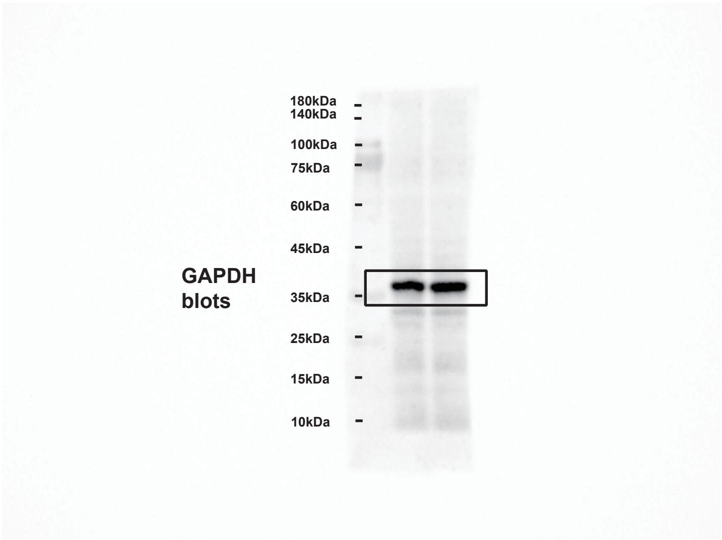


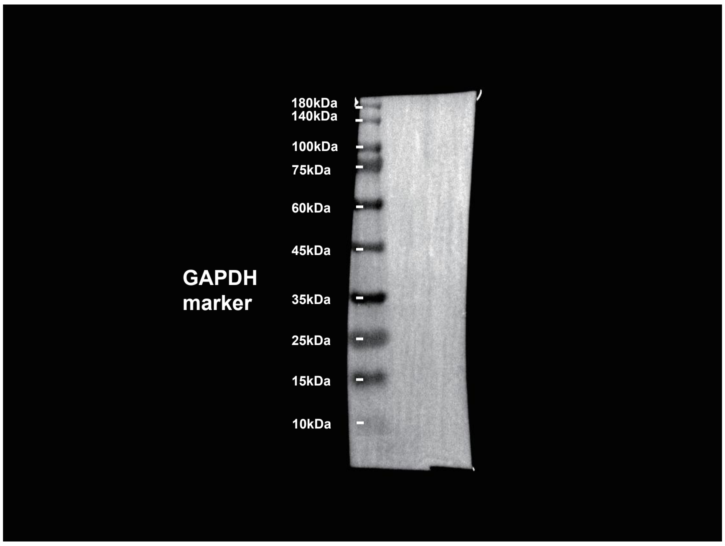

Fig. S12B

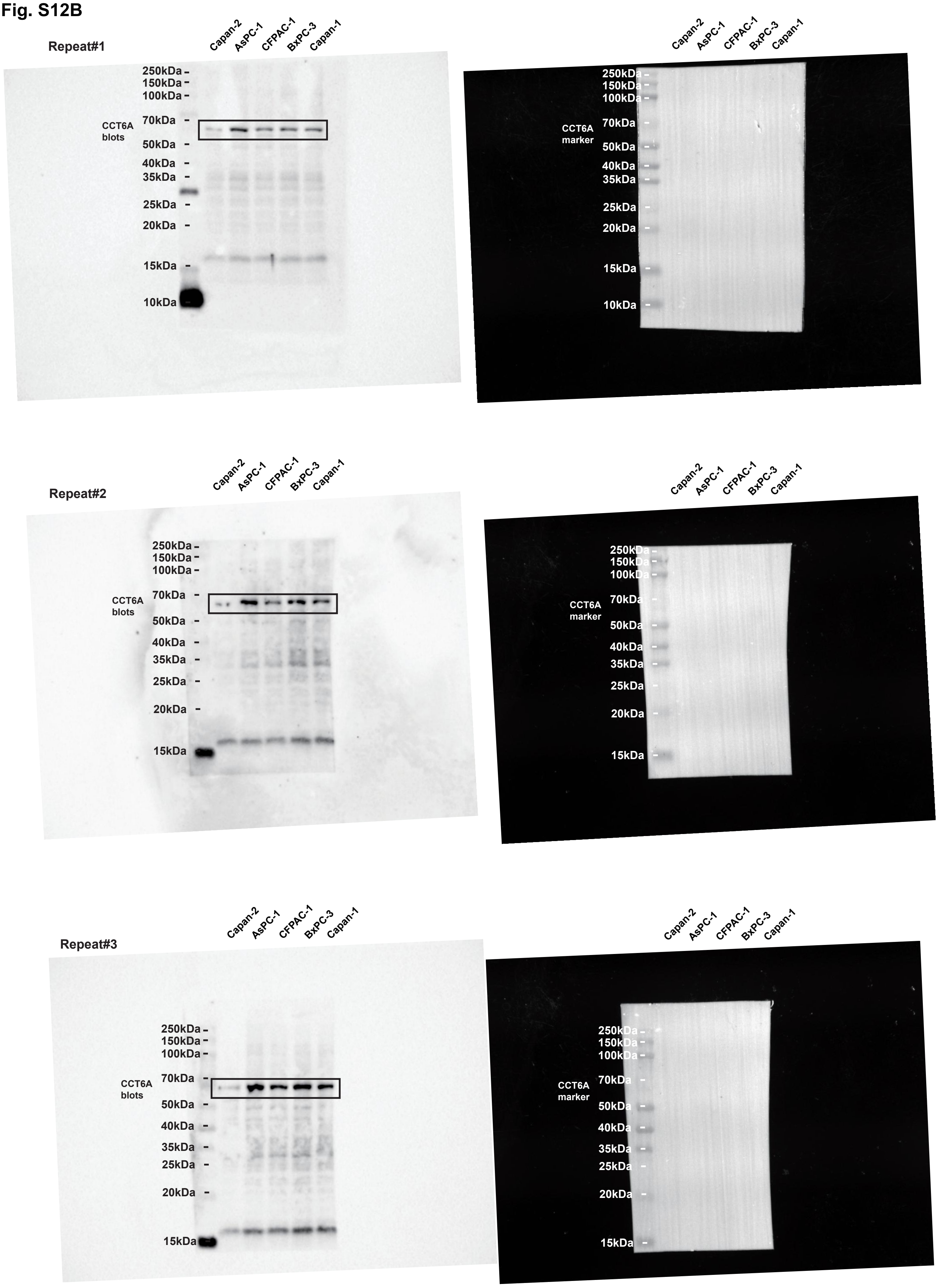


| | CCT6A | | | GAPDH | | |
|---|---|---|---|---|---|---|
| Capan-2 | 375448.71 | 331525.57 | 282865.36 | 4341811.3 | 4169351.1 | 3428166.6 |
| AsPC-1 | 2035825.3 | 2038331.7 | 2132990.5 | 3871852.2 | 3702277.5 | 3616034.5 |
| CFPAC-1 | 1071501.9 | 902268.43 | 1038751 | 3580500.9 | 3456202.8 | 3643916.5 |
| BxPC-3 | 1272025.7 | 1660169 | 1644622.9 | 3405418.1 | 3412159.3 | 3352888.3 |
| Capan-1 | 1185893.1 | 1167122.6 | 1127667.5 | 3574347.3 | 3741846.4 | 3607992.2 |

Repeat#1

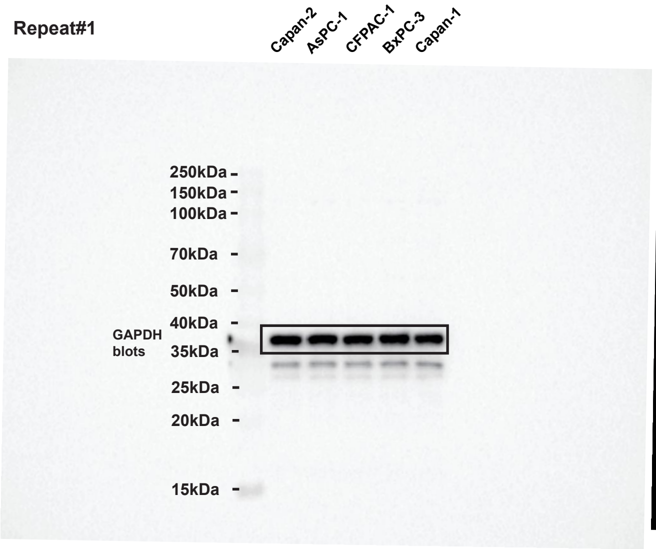


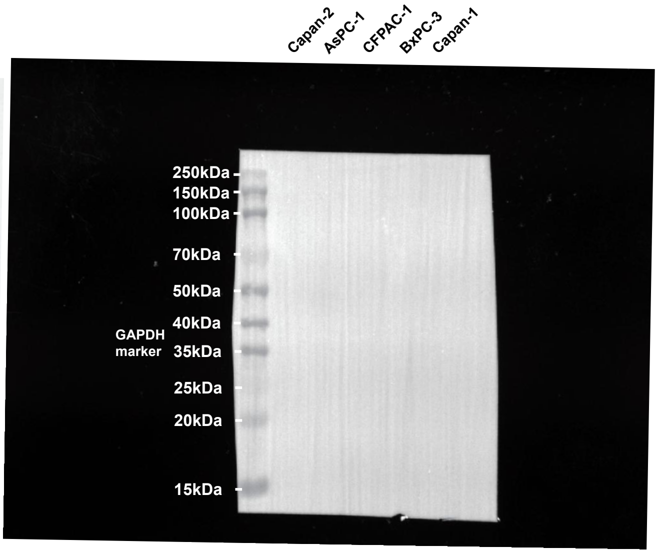


Repeat#2

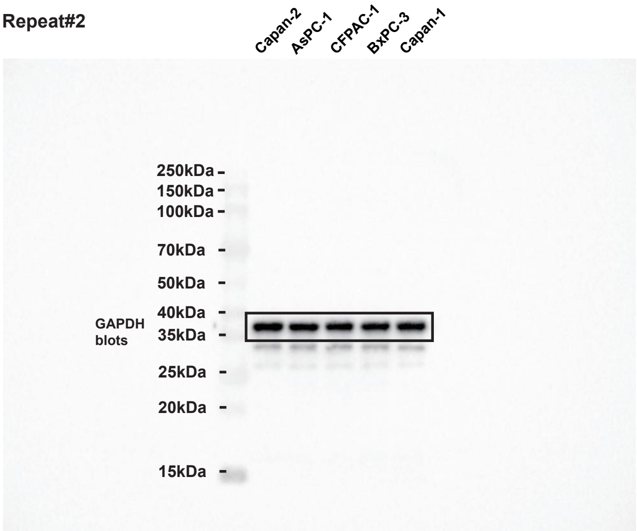


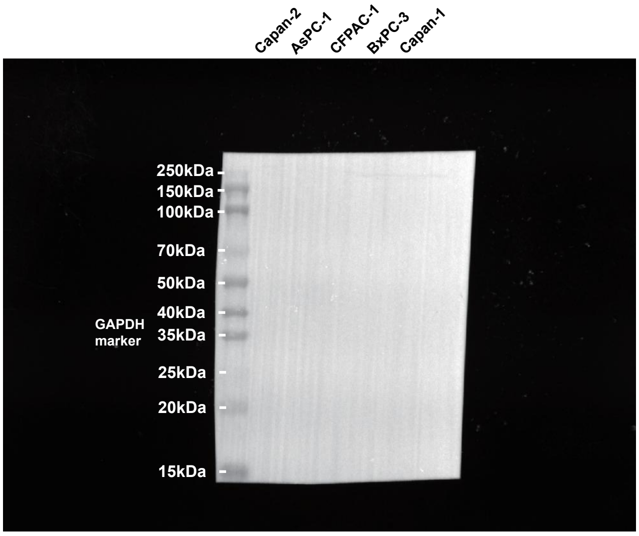


Repeat#3

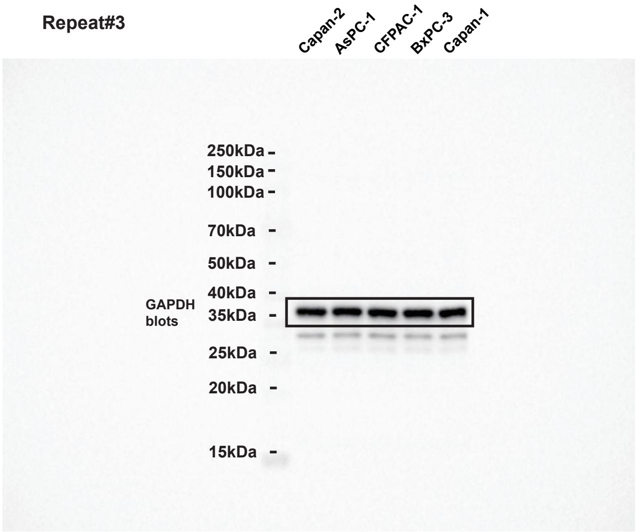


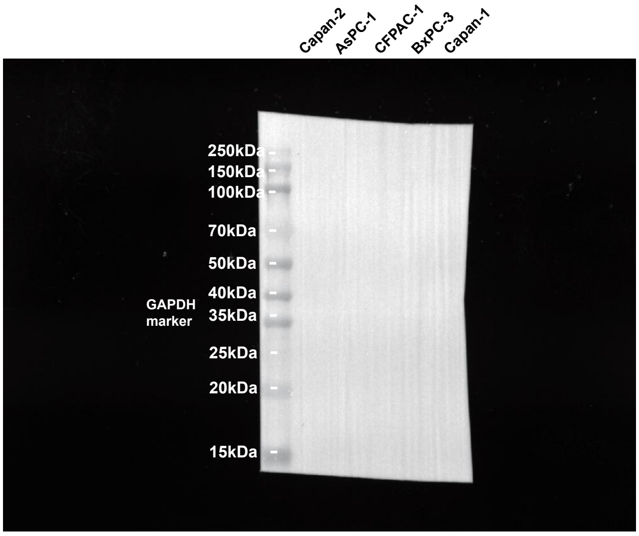


| | CCT6A | | | GAPDH | | |
|---|---|---|---|---|---|---|
| Capan-2 | 375448.71 | 331525.57 | 282865.36 | 4341811.3 | 4169351.1 | 3428166.6 |
| AsPC-1 | 2035825.3 | 2038331.7 | 2132990.5 | 3871852.2 | 3702277.5 | 3616034.5 |
| CFPAC-1 | 1071501.9 | 902268.43 | 1038751 | 3580500.9 | 3456202.8 | 3643916.5 |
| BxPC-3 | 1272025.7 | 1660169 | 1644622.9 | 3405418.1 | 3412159.3 | 3352888.3 |
| Capan-1 | 1185893.1 | 1167122.6 | 1127667.5 | 3574347.3 | 3741846.4 | 3607992.2 |
